# Mind the governance gap: a one health scoping review of national AMR performance indicators

**DOI:** 10.1038/s44259-026-00213-8

**Published:** 2026-05-13

**Authors:** Emily T. O’Neill, Uswa Shafaque, Vassilis Karadimitris, Brandon L. Hill, Sruthi Ranganathan, Danilo Lo Fo Wong, Ketevan Kandelaki, Elias Mossialos, Michael Anderson

**Affiliations:** 1 LSE Health, The London School of Economics and Political Science, London, UK; 2 Department of Medicine, University of Cambridge, Cambridge, UK; 3 World Health Organization (WHO) Regional Office for Europe, Copenhagen, Denmark; 4 Department of Health Policy, London School of Economics and Political Science, London, UK; 5 Health Organisation, Policy, Economics (HOPE), Centre for Primary Care & Health Services Research, The University of Manchester, Manchester, UK; 6 Present Address: International Centre for Antimicrobial Resistance Solutions (ICARS), Copenhagen, Denmark

**Keywords:** Health care, Medical research

## Abstract

Despite several initiatives to benchmark national action in responding to antimicrobial resistance (AMR), no comprehensive inventory exists of indicators used across these efforts. We present a scoping review that maps and synthesises existing indicators used to assess national performance in addressing AMR. We conducted a review of academic and grey literature to identify quantitative and qualitative indicators used to assess national AMR performance across animal, environmental, and human health sectors. The academic search was conducted across MEDLINE, EMBASE, and Global Health. The grey literature search was conducted across multilateral organisations (e.g., WHO, FAO, WOAH, UNEP, OECD) and think-tanks. We categorised indicators into a thematic framework spanning 19 subdomains within three overarching domains: Governance & Leadership, Action Areas, and Monitoring & Evaluation. From 184 academic studies, we identified 2311 indicators, and 1406 from 48 grey literature sources. In total, 2101 were in human health, 675 in animal health, 232 in environmental health, and 709 were multisectoral. Within the three overarching domains, 281 were Governance & Leadership, 1907 were Action Areas, and 1671 were Monitoring & Evaluation indicators. When grouping indicators by subdomain, Surveillance & Laboratory (1427), Stewardship (765), and Prevention & Control (365) accounted for the largest volumes, followed by Social Determinants (232), Reporting (141), and Workforce (140), in contrast to Accountability (22), Transparency (22), and Equity (29). This comprehensive scoping review provides a One Health inventory of 3717 indicators used to assess national performance in responding to AMR. While revealing robust Surveillance & Laboratory, Prevention & Control, and Stewardship metrics, we identify critical gaps in Accountability, Transparency, and Equity. These measures comprise the Governance & Leadership domain, which is essential for policy implementation.

## Introduction

Antimicrobial resistance (AMR), leading to an estimated 1.14 million deaths worldwide in 2021 alone^1^, is a critical, complex public health issue. By 2035, the global economy is projected to incur additional healthcare costs of $412 billion, and losses of $443 billion from reduced workforce productivity^2^.

International efforts to combat AMR have intensified since 2011, when the WHO Regional Office for Europe (WHO Europe) and the European Commission adopted regional action plans for AMR^3,4^. This was followed by the WHO 2015 Global Action Plan (GAP) and the 2016 and 2024 United Nations (UN) political declaration on AMR^5^. In 2023, the WHO Europe member states endorsed an AMR roadmap to identify, prioritise, implement, and monitor high-impact interventions to tackle AMR^6^. Despite these initiatives, countries are inconsistent when translating global and regional commitments into practice due to differing political priorities, economic constraints, and healthcare infrastructure capacities^7^.

In response, stakeholders have advocated for target-based approaches, such as the global 10–20–30 targets for 2030, the targets within the 2024 UN political declaration on AMR (UNGA), and the EU’s One Health Action Plan goals^5,8,9^. Targets provide specific, measurable objectives that can unify and accelerate country-level efforts, but reliance on targets alone risks oversimplifying the multifactorial drivers of and policy responses to AMR^10^. Several initiatives exist that monitor different aspects of AMR national policy, such as the Global Database for Tracking AMR Country Self-Assessment Survey (TrACSS)^11^, IHR States Parties Self-Assessment Annual Report (SPAR)^12^, WHO Infection Prevention and Control Assessment Tool 2 (IPCAT2)^13^ the Joint External Evaluation (JEE)^14^, the Global Coalition on Aging and Infectious Disease Society of America AMR Preparedness Index^15^, the AMR Governance Framework^16^, FAO’s Assessment Tool for Laboratories and AMR Surveillance Systems (ATLASS)^17^, and the WOAH Observatory Monitoring Reports^18^. However, these initiatives are sometimes conducted and interpreted in isolation, resulting in a fragmented evidence base that limits the ability of policymakers to form a comprehensive, cross-sectoral view of national AMR policy responses.

We conducted a literature review to identify and consolidate existing quantitative and qualitative indicators currently used, or proposed, to compare national performance in addressing AMR. We aimed to highlight best practices, uncover gaps in indicator coverage, and identify candidates for inclusion within a novel proposed One-Health AMR Accountability Index^19^. While previous reviews examine selected aspects of AMR policy implementation, this study encompasses academic and grey literature to map indicators across the full One Health spectrum and, to our knowledge, constitutes the most comprehensive resource to date that identifies gaps, reduces duplication, and helps develop balanced, multisectoral indicators to measure national performance in addressing AMR.

## Results

### Scoping review

**Fig. 1:**
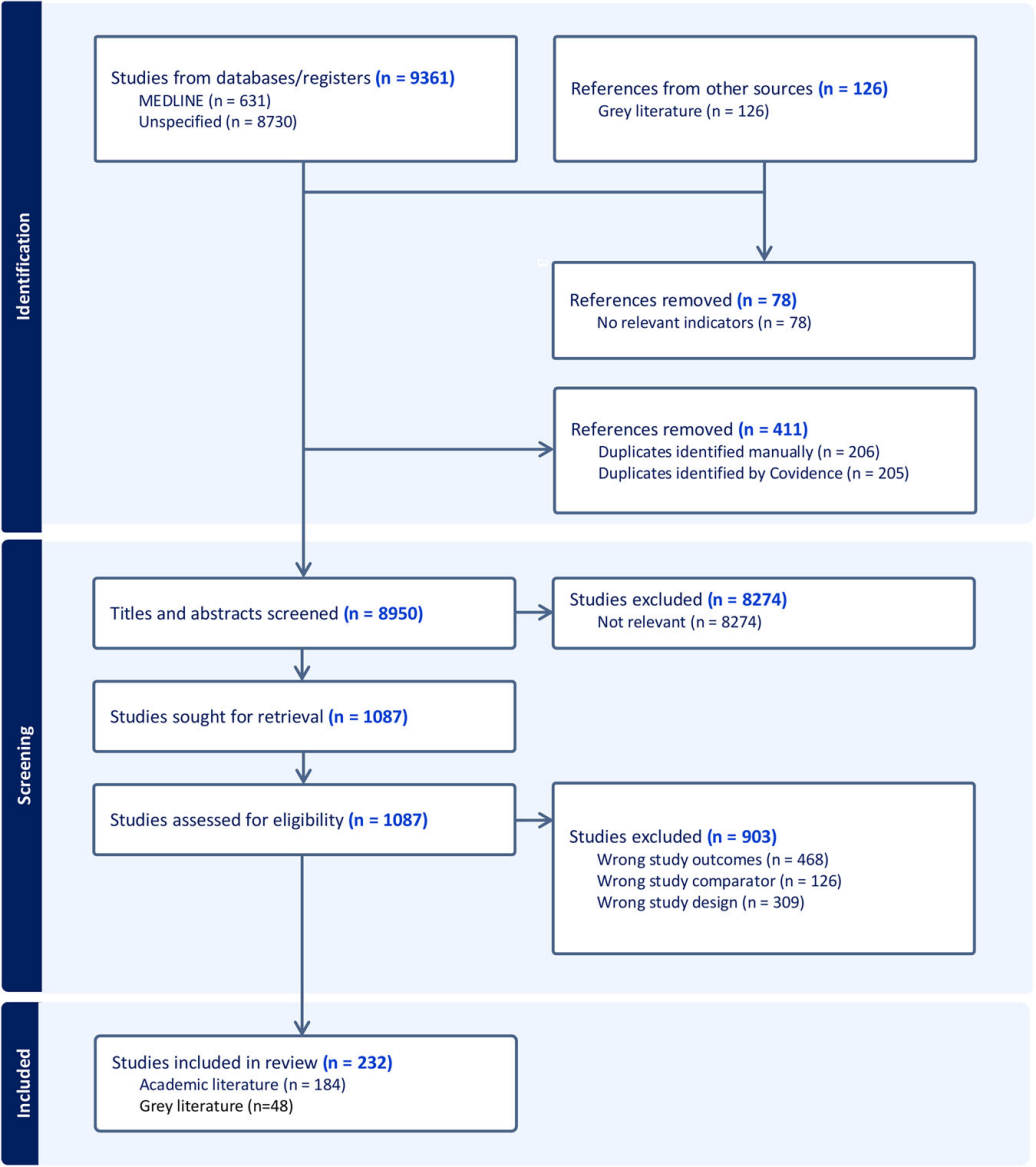
PRISMA flow diagram of studies included in scoping review. Authors’ creation.

Our academic search identified 9361 results (Fig. 1). Following title and abstract screening, 1087 results remained. We further excluded 468 (wrong outcomes), 126 (wrong comparator), and 309 (wrong study design). This resulted in 184 academic studies and 48 grey literature sources. Levels of interrater-reliability observed were substantial, for abstract and title screening (0.78), and fair, for full text screening (0.30). The lower agreement at the full-text stage is consistent with the greater complexity of eligibility decisions required at this level, where a more nuanced judgement was required regarding the inclusion and exclusion criteria. All disagreements were resolved through adjudication by a third independent reviewer (MA).

**Fig. 2:**
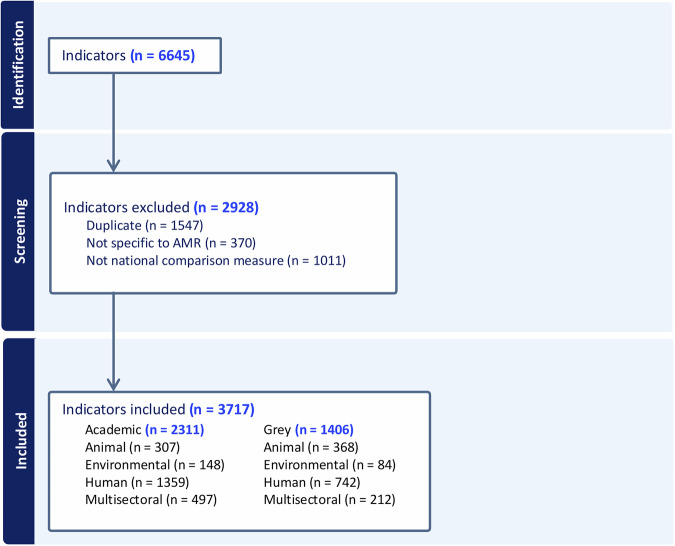
PRISMA flow diagram of indicators included in scoping review. Authors’ creation.

Supplementary Table 1 lists grey literature and source links, while Supplementary Tables 2 and 3 summarise the literature included. From these sources, 6645 indicators were initially extracted (Fig. 2). During screening, 2928 indicators were excluded: 1011 (not considered cross-national measures of performance in addressing AMR); 1547 (duplicates); 370 (not specific to AMR). This resulted in 3717 indicators eligible for inclusion.

**Fig. 3:**
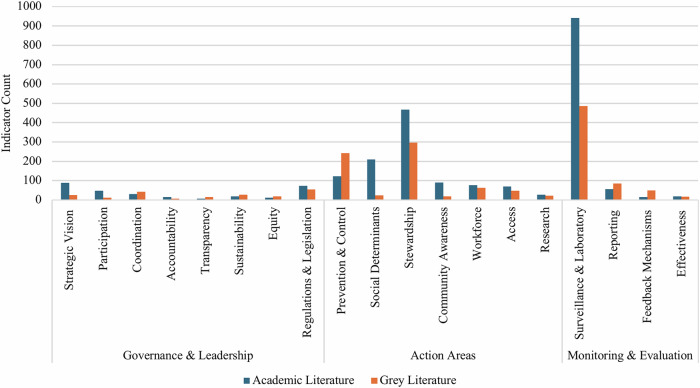
Indicators within 19 government framework subdomains for academic and grey literature. Figure Notes: Some indicators and studies appear in multiple subdomains; subdomain sums may exceed the overall total. Access to Medicines & Health Services (Access); Community Awareness & Enabling Behaviours (Community Awareness); Research, Innovation, & Digital Technology (Research).

We identified 2311 unique indicators from academic studies and 1406 from grey literature sources (outlined in Supplementary Data 1 and 2). Table 1 provides the number of academic studies, grey literature sources, indicators identified, and example indicators within each AMR policy subdomain. A summary visualisation of indicator density across the 19 subdomains illustrates substantial concentration in Surveillance & Laboratory, Stewardship, Prevention & Control, and comparatively few indicators for Accountability, Transparency, and Equity subdomains (Fig. 3). Since indicators could be grouped under multiple subdomains, in some instances the totals exceed the number of unique indicators identified (*n* = 3717). Supplementary Figure 1 visualises indicator groupings by sector (animal, environmental, human health, multisectoral). We provide below an in-depth narrative synthesis of indicators grouped under their subdomains, with notable findings. Supplementary Figure 2 displays the distribution of academic studies by publication year; most appeared within the past five years.

**Table 1:** Overview of subdomains showing the number of academic studies and grey literature sources reviewed

Domain	Sub-domain	Definition	Academic indicators	Academic studies	Grey literature indicators	Grey literature studies	Example indicator
*Monitoring & Evaluation*	Surveillance & Laboratory	Indicators that assess the comprehensiveness of surveillance programmes and the capacity and quality of laboratory systems across all sectors.	941	118	486	32	What is the status of the national surveillance system for antimicrobial resistance in live aquatic animals?^11^
	Reporting	Mechanisms for communicating progress on the national AMR strategy to external stakeholders, including annual progress reports and formal submissions to international and regional surveillance initiatives (e.g., GLASS, ECDC, CAESER, EARS-Net)	56	18	85	12	Does the country report use of antibiotics (ATC classes J01, A07AA, P01AB) and do they report route of administration (oral, parenteral, inhalation, rectal)?^63^
	Feedback Mechanisms	Indicators that capture the existence and quality of processes for relaying surveillance data and performance information back to regional, organizational, and individual stakeholders	16	6	50	13	Select all that apply: 1) Quality audits of long-term care facilities (LTCFs) include AMR. 2) There is monitoring and evaluation of AMR in LTCFs^44^.
	Effectiveness	Indicators examine whether structured mechanisms exist at the national level to evaluate if policies or interventions achieve their intended health and economic outcomes.	19	9	17	8	Estimation of the incidence of patients acquiring at least one hospital-acquired infection (HAI) in acute care hospitals per 1000 hospital admissions. Estimation of all types of HAIs per year in acute care hospitals per 1000 hospital admissions^13^.
*Governance & Leadership*	Regulations & Legislation	Covers all forms of legislation, incentives, and penalties in place for the containment of AMR.	74	13	54	14	Does the country have legislation in place that limits or prohibits the over-the-counter sale of antibiotics without a prescription?^49^
	Strategic Vision	Indicators that measure the extent to which there exists a national long-term strategy that has been informed by a situational analysis, with specific, measurable, actionable, relevant and time-bound (SMART) aims for the containment of AMR.	88	26	25	13	Is there a national strategic plan for hospital-acquired infection surveillance (with a focus on priority infections based on the local context) developed by the multidisciplinary technical group?^48^
	Participation	Indicators that evaluate the extent of inclusive involvement of all relevant stakeholders in development, implementation, monitoring and evaluation of national strategies to address AMR.	47	19	11	7	Who is involved & what is the role for priority setting regarding AMR action and standards?^20^
	Coordination	Indicators that measure the comprehensiveness of mechanisms in place for coordination across One Health sectors.	30	14	42	16	What is the level of effective integration of laboratories in AMR surveillance in the animal health and food safety sectors?^48^
	Accountability	Indicators that measure whether responsibility is allocated to deliver objectives and activities listed within the NAP, and whether agreements exist if actions are not undertaken or targets not met.	15	9	7	6	What is the number of focal persons in the network who are reference persons for AMR activities?^23^
	Transparency	Indicators that measure the extent to which NAPs, progress reports, funding information and other data are publicly available and accessible.	6	6	16	7	Is the information on the national action plan, including progress reports, AMR surveillance data, funding allocation, responsible body, and responsible person/sector within the country, publicly available?^75^
	Sustainability	Assesses the commitment of relevant stakeholders, the degree to which adequate budgets for present and future activities are allocated, and the consistency with which support is provided for AMR policy development, implementation and monitoring.	18	9	28	14	Is the country’s funding for One Health initiatives in government budget? If Yes, what is the amount of funding? Describe the extent of technical support provided to build capacities to address AMR through support for implementation of international standards and action plans, trainings, and R&D^80^.
	Equity	Indicators that measure the extent to which AMR policy facilitates equitable access to antimicrobials or mitigates the impact of AMR on disadvantaged and vulnerable populations.	11	3	18	3	Does the country promote equal participation of women, men and other vulnerable groups and/or groups facing discrimination in the multisectoral AMR coordination mechanism and technical working groups?^85^
*Action Areas*	Prevention & Control	Measures of the comprehensiveness of IPC programmes, biosecurity mechanisms, and access to clean water, sanitation, and hygiene between countries.	122	35	243	22	Are a minimal set of core indicators for infection, prevention, and control (IPC) within healthcare facilities in the country defined?^108^
	Social Determinants	Encompass the structural and contextual factors that shape AMR risk between countries, including educational level, climate change, pollution, conflict, nutrition, housing, finance status, demographic trends, technological environments, and individual characteristics.	209	19	23	4	What are the behavioural barriers, including structural barriers, to tackling AMR?^32^
	Stewardship	Indicators that measure the comprehensiveness of efforts to reduce inappropriate antimicrobial use such as stewardship programmes and guidelines for antibiotic use, and consumption rates and sales of antibiotics	468	80	297	31	Report antimicrobial use for ATC class J01, A07AA, P01AB by AWaRe (Access, Watch, and Reserve) classification (relative use % of Access class antibiotics; relative use % of Watch class antibiotics; relative use % of Reserve class antibiotics)^63^.
	Community Awareness & Enabling Behaviors	Indicators designed to improve knowledge, shape attitudes, and foster practices that support responsible antimicrobial use.	91	18	18	11	What is the amount of funding mobilised for communication actions on AMR and antimicrobial stewardship per 1000 population?^80^
	Workforce	Indicators referring to the development, education, and training of teams and individuals across the One Health sectors.	77	22	63	10	Does the country provide training and professional education on AMR to the agriculture (animal and plant), food production, food safety, and environment sectors?^11^
	Access to Medicines & Health Services	To what extent the population experiences barriers when accessing healthcare services and essential medicines.	70	20	48	11	What is the unmet need for each of the following due to cost, distance, and waiting times? 1) Health care 2) Dental care 3) Prescribed medicines^64^.
	Research, Innovation, & Digital Technology	Assess whether there is investment in research to examine the drivers of AMR, effectiveness of interventions to mitigate AMR, and whether there are incentives in place that aim to foster innovation.	28	16	22	6	Amount of funding allocated for translational research and late-stage development of AMR medical countermeasures, including clinical trials for antimicrobials^80^.

The total indicators extracted, and representative example indicators per subdomain. Some indicators and studies appear in multiple subdomains; subdomain sums may exceed the overall total.

### Domain: governance & leadership; subdomain 1: strategic vision

Strategic Vision refers to indicators relating to the adoption of a national long-term strategy informed by a situational analysis, with specific, measurable, achievable, relevant, and time-bound (SMART) aims for addressing AMR. Indicators also measure whether there is a high-level commitment to tackling AMR, e.g., AMR appears in political manifestos or on national risk registers. 88 indicators were extracted from 23 academic studies^20–42^; 25 from 12 grey literature sources^11,13,15,17,43–50^ (in particular, from Implementation of WOAH Standards: The Observatory Annual Report 2022)^18^.

Indicators include measurements of operational plan comprehensiveness^34^, zoonotic disease governance^21^, overall National Action Plan (NAP) design^22^, and specific NAP characteristics (e.g. shared leadership^27,33^, advocacy facilitators, functionality, preparedness and response to public health events, and incorporation of the primary healthcare sector)^28^. While some indicators measure whether countries have adopted a NAP^21,32^, and progress with NAP development^29,30,45^, most gauge whether specific elements are included, e.g. the implementation of a One Water stewardship framework^36^, antimicrobial use (AMU) reduction across One Health sectors^41^, the integration of long-term care facilities^44^, and sections dedicated to environmental, food and agricultural issues^17,43,46^. Certain indicators from the academic literature evaluate the level of political recognition and support that strategies and targets receive^21,25,31^.

Indicators also evaluate the degree to which NAPs incorporate international bodies’ priorities. This especially concerns alignment between NAPs and the GAP^24,35,37,39,42^, on infection prevention control (IPC), AMU and surveillance^34^, best practices^26,38^, support for IPC programmes^13^, and governance training programmes (e.g. provided by Medicines, Technologies, and Pharmaceutical Services (MTaPS)^23^. Other indicators consider the standards and criteria for implementation of targets^40^, the existence of a focal point or technical group to lead NAP implementation and monitoring and evaluation (M&E)^11^, and whether the setting of priorities is based on empirical evidence^20^.

### Domain: governance & leadership; subdomain 2: participation

Participation refers to indicators that evaluate the extent to which the development, implementation, and M&E of national AMR strategies involve stakeholders, e.g., civil society, healthcare professionals, veterinarians, the food and pharmaceutical industries, and subject matter experts. Forty-seven indicators were extracted from 19 academic studies^20,21,23,27,29,31–33,39,51–60^; 11 from 7 grey literature sources^15,18,49,61–64^.

Indicators assess ‘participatory’, inclusive and consensus-oriented decision-making at the governmental and inter-governmental level^21^. They also evaluate stakeholders’ level of contribution and participation across One Health sectors^52^ and map the type of persons associated with AMR national-level policy-making^20,54^. Other indicators gauge how many stakeholder support activities involve non-state actors^31^, the participation of private-sector entities in AMR-related decision-making, and the safeguards against potential conflicts of interest^62^. Indicators consider participation in international AMR-related initiatives^55^, e.g. campaigns^56^, laboratory training programmes^60^, and surveillance data repositories^15,29,32,57–60^; and stakeholder participation in facilitating mechanisms, e.g. regional and national-level AMR meetings^51^ and joint programmes involving agricultural and veterinarian actors^61,65^.

### Domain: governance & leadership; subdomain 3: coordination

Coordination refers to indicators that measure the comprehensiveness of mechanisms linking up One Health sectors, e.g. multisectoral committees. Thirty indicators were extracted from 14 academic studies^21–30,52,53,66,67^; 42 from 15 grey literature sources^11,13–15,17,43,50,61,62,68–73^.

Multiple indicators, both from the academic and grey literature, assess the extent to which national-level coordination includes all One Health sectors. Studies employ ordinal^11,14,27,30^, quantitative^22^, qualitative^53^, and multiple-choice^72^ metrics for overall assessments of One Health intersectoral coordination capacity. Other indicators record stakeholder contribution to coordinated initiatives^11,43,52^.

One Health initiatives are often included within NAPs^29^, in addition to collaborative research activities^66^. Other studies consider the level of coordination within sectors (e.g., between agricultural and veterinary stakeholders^52^ or intra-sectoral chains of command)^61^. For the human sector, indicators ordinally measure coordination between governments, NGOs and patient groups^15^; and institutional linkages between IPC and water, sanitation, and hygiene (WASH) programmes, patient associations, civil society bodies, and medical societies^11,13,30,66^.

Nationally and internationally, indicators also evaluate institutional alignment and collaboration, e.g. whether a country’s NAP is linked to or integrated with its other national strategies^11,21^; the extent to which national reference laboratories support the integration of peripheral laboratories across the veterinary and agricultural sector^70^; and how decision-making competences are shared between centralised levels of AMR governance^62^. Internationally, indicators measure integration of addressing AMR into other global agendas^24–26^, and countries’ collaboration with international research^15^ and programmes such as MTaPS^23^.

Several indicators assess national mechanisms for facilitating coordinated activity, e.g. whether countries have national, multisectoral coordinating committees^17,26,28,53,71,72^ and the extent to which local and national IPC monitoring, audit and feedback mechanisms are aligned^13^. Other indicators evaluate coordinating mechanisms, e.g. operational documents^43^, integrated laboratory data-sharing networks^69^, emergency logistics and supply chain management systems^69^, International Food Safety Authorities Network contact points^72^, and information-sharing processes for regulatory bodies^43^.

### Domain: governance & leadership; subdomain 4: accountability

Accountability refers to indicators that assess whether responsibility is allocated to certain individuals or organisations to deliver the NAP’s objectives, and whether sanctions exist if any are unmet. Fifteen indicators were extracted from nine academic studies^20–23,27,51,66,74,75^; 7 from 5 grey literature documents^11,13,61,76,77^. In both sets of sources, indicators measure accountability at the national level through overall, quantitative scores^21,22^.

Indicators demonstrate the existence of relevant authorities to which responsibility for AMR-related initiatives can be allocated, e.g., focal persons acting as reference persons for AMR activities^51^, or focal working groups responsible for M&E and NAP implementation^11^. Other indicators map the role and scope of authorities and stakeholders, outlining mechanisms for delegating, accrediting, and authorising AMR-related responsibilities^13,61^. These mechanisms are evaluated according to their linkage to broader health system governance structures^20^, and their compliance with national-level policies and regulatory frameworks, concerning, e.g. food policy^21^. Lastly, indicators consider facilitating practices, e.g., institutional audits^51^; MTaPS-supported activities for entities addressing AMR^23^; the consistent and reliable reporting of information to national or international health authorities^74,77^.

### Domain: governance & leadership; subdomain 5: transparency

Transparency encompasses indicators that measure the public availability and accessibility of NAPs, progress reports, funding information, and the extent to what degree do AMR policy development, implementation, and evaluation occur in an accessible and open environment. Six indicators were extracted from 6 academic studies^21,22,27,75,78,79^; 16 from 7 grey literature sources^17,18,61–63,71,77^. In both sets of sources, indicators measure AMR governance transparency through overall, quantitative scores^21,22^ and respondent awareness of AMR risks and response^79^.

Indicators consider whether information (e.g., NAPs^21,75,78^, progress reports, surveillance data^17^, funding allocations, a log of responsible entities^75^, audits of AMR treatment guidelines^71^, risk assessment outcomes)^17^ is published and accessible^17,62,63^. Other indicators assess whether stakeholders fulfil their obligations to facilitate transparency, either by sharing information^77^ or by consistently and punctually informing other AMR-related stakeholders about sector-specific developments and activities^61^.

### Domain: governance & leadership; subdomain 6: sustainability

Sustainability encompasses the commitment of relevant stakeholders, the allocation of adequate budgets for present and future activities, and the consistency with which support is provided for AMR policy development, implementation and monitoring through the input of subject matter experts through technical advisory groups. Eighteen indicators were extracted from 9 academic studies^21–23,26–28,31,34,80^; 28 indicators from 14 grey literature sources^11,13,15,17,43,44,46,61,69,72,80–83^. Two studies measure sustainability through overall scores^22^, while another qualitatively assesses the overall sustainability of national veterinary laboratory systems^61^.

Indicators assess whether AMR activities are financially sustainable, e.g. ensuring accessibility of funds^61^; the appropriate costing of activities^34^; the identification, mobilisation, and diversification of funding^15,26,34,43,72,80^; the protection of allocated funds for operating budgets and resources^13,28,81^. Other indicators gauge AMR funding and investments’ level of government backing^15,21,31,44,46,72^. Lastly, indicators consider the existence of other, non-financial support that facilitates sustainable practices, e.g. advocacy mechanisms for International Health Regulation (IHR) implementation^72^; experts’ participation in a NAP implementation campaign^28^; MTaPS-supported activities for building technical capacity^23^; active Drug Safety Monitoring expert committees^69^.

### Domain: governance & leadership; subdomain 7: equity

Equity refers to indicators that measure the extent to which AMR policy facilitates equitable access to antimicrobials or mitigates the impact of AMR on disadvantaged and vulnerable populations. Eleven indicators were extracted from three academic studies^21,22,84^; 18 from 3 grey literature sources^11,72,85^, in particular from the WHO guidance: addressing gender inequalities in national action plans on AMR^85^.

Indicators include geographic factors that impact access^86^, economic inequality and development measures^21^. Another measures the impact of a country’s poverty gap and public sanitation services on AMR^84^. Other indicators inquire whether national-level data are captured and categorised according to social stratifiers^11,49,87^. Grey-literature indicators assess if inclusion of vulnerable groups is promoted across all AMR-related national mechanisms^49^. Indicators also include retrospective reviews of services, policies, and education to identify and reduce gender biases or inequalities^21,49,72^.

### Domain: action areas; subdomain 8: regulations & legislation

Regulations & Legislation covers legislation, incentives, and penalties in place for the containment of AMR. Seventy-four indicators were extracted from 10 academic studies^21,22,34,66,88–93^; and 54 from 14 grey literature sources^11,15,17,44,46,48,49,61,62,69,71,72,77,85^. Three studies generated 49 indicators^18,66,88^. Within the grey literature, over half came from WHO’s TrACSS^11^.

Many indicators focus on oversight of AMU and enforcement of guidelines, laws, and regulatory instruments. To regulate AMU, indicators consider whether legislative frameworks exist to prohibit or severely restrict highly critical antibiotics^89^ in animal health^34,48,66,77,89^. Indicators also include regulations for antibiotic use in human health, establishing whether over-the-counter (OTC) sales without prescription are banned^71^ and OTC prescriptions are written by legally authorised persons^49,77,89^. Other indicators focus on elements of antimicrobial supply legislation, e.g. controlling the marketing of pesticides; country-level coverage of drug regulations in the supply chain^21,92^. Indicators also consider the potential contribution of regulations that might reduce AMU^91^, e.g. taxing antibiotics for livestock use; restricting broad-spectrum antibiotics’ use in growth promotion^15,90^.

Several indicators evaluate the existence and adequacy of legislation for safe wastewater treatment and disposal. TrACSS, for example, inquires whether national legislation is in place and enforceable for preventing environmental antibiotic contamination^11,88^. Indicators also quantify antibiotic residue according to environmental protection criteria^49^. Other indicators gauge regulation concerning the detection and prevention of substandard and falsified medical products in human^49^ and animal health^61,62^. Finally, certain indicators assess other forms of legal or regulatory instruments, e.g. nutrition guidelines^21^, legislation for AMR surveillance^17,80^, transnational transport of food, goods, animals and people^11^, IHR implementation^72^, and contextual factors including rule of law and corruption^21^.

### Domain: action areas; subdomain 9: prevention & control

Prevention and control refer to IPC programmes, biosecurity mechanisms, access to clean water and sanitation, and hygiene. 122 indicators were extracted from 33 academic studies^21,22,24,26,28,30,32,36,40,51,53,55,57,60,66,74,80,84,86,90,93–105^; 243 from 21 grey literature studies^11–13,15,18,43,44,46,49,61,68,69,71,77,83,106–112^, including 100-plus from IPCAT2^13^.

Indicators for WASH include the proportion of the population with basic or safely managed drinking water and sanitation facilities; access to hygiene installations in communities and healthcare facilities; hand hygiene infrastructure; waste management services; environmental cleaning protocols; and WASH adherence^21,84^. Human health IPC indicators cover the existence of national IPC programmes^11^; focal points^108^; budgets^44^; updated and implemented national and facility-level guidelines^13,108^; adaptation and implementation of multimodal strategies such as the States Parties Self-Assessment Annual Report^12^; integrated monitoring and audit frameworks linked to AMR and WASH surveillance, microbiological support for healthcare-associated infection (HAI) surveillance, clear reporting lines, timely feedback mechanisms^13^; defined surveillance objectives and benchmarking^69^; capacity assessments such as Joint External Evaluation (JEE) IPC scores^26,55^; and institutional policies and training for IPC personnel^13^. Animal health indicators include farm biosecurity measures^90^; livestock density and diversity metrics^21^; animal–human contact rates^95^; inspection and hygiene practices along the farm-to-fork food chain^21^; good manufacturing practices^30^; manure management^21^; traceability and movement control, zoning and compartmentalisation^61^; preparedness plans for containment of resistant pathogens^68^; vaccination strategies for zoonoses^61,68^; vector control measures^69^; and monitoring of antibiotic residues and environmental discharges^11,49^. Policy and planning indicators encompass the percentage of NAPs addressing WASH^98^; environmental contamination, inclusion of One Health environment sector scorecards, the existence of national strategic plans for HAI and AMR surveillance that are aligned with IHR^12,36,98^; vaccination coverage metrics for key pathogens^68^; AMU reduction interventions on farms^71^; and stakeholder perceptions of biosecurity improvements^13^.

We observed crossover between indicators for IPC and other domains, including Coordination, Feedback Mechanisms, and Accountability. Multi-domain indicators include presence of IPC M&E plans for NAPs^13,108^; consistency and quality of surveillance platform data, national platforms for IPC data-sharing, feedback mechanism for IPC scores, and clarity on responsible entities for IPC performance monitoring^13^; cross-sector linkage of IPC with AMR, WASH, and environmental programmes, regular audits and guideline reviews^99^; training programmes for IPC professionals^13^; outbreak preparedness and response capacity^68^; and health outcome monitoring, e.g., HAI incidence^40^, *Clostridioides difficile* infections surveillance, sepsis critical care admissions, and measures of population-level intervention impact on infection rates^57^.

### Domain: action areas; subdomain 10: social determinants

Social determinants encompass the structural and contextual factors that shape AMR risk within different populations across countries, including educational level, climate change, pollution, conflict, nutrition, housing, financial status, demographic trends, technological environments, and individual characteristics such as gender, age, and ethnicity. A total of 209 indicators were extracted from 19 academic studies^21,32,36,42,84,86,91,92,94–96,98,101,102,113–117^, with over half of the indicators coming from one study^21^; 23 from 4 grey literature studies^69,77,109,110^, including 15 from the Joint Monitoring Programme for Water Supply, Sanitation and Hygiene (JMP)^110^.

Indicators for WASH appeared in both IPC and Social Determinants^21,94–96,98^. Economic and financial indicators comprise GNI per capita, GDP, public social and education expenditure, unemployment, labour force participation, income inequality, and measures of political stability, corruption perception, and rule of law^21,94,96^. Demographic indicators cover urbanisation rates, age distribution, gender and ethnic composition, population growth, migration or refugee ratios, and population density or proximity to water bodies, all of which affect transmission dynamics and healthcare demands^21,77,96,110,113^. Nutrition and food security metrics such as undernourishment prevalence, stunting in children, anaemia among women of reproductive age, dietary energy and protein supply adequacy, food price indices, and food insecurity ratios highlight vulnerability to infection and influence AMU in agriculture and human health^21^.

Many environmental indicators were grouped within the Social Determinants subdomain (89). These include climate indicators, e.g. average temperature and precipitation, air quality, CO_2_ and greenhouse gas emissions, climate risk indices, water stress and renewable water resources, land use factors, pollution scores, and indicators of ecosystem health, all of which affect pathogen emergence, spread, and AMR selection pressures^21,94,96^. Housing and living conditions, e.g., quality of housing, overcrowding, and access to electricity, influence infection risk and healthcare-seeking behaviour^110^. Technological and infrastructure indicators include internet penetration, mobile subscriptions, logistics performance, transport accessibility, electricity access, and technology adoption rates, which shape health communication, surveillance capacity, and distribution of medical and veterinary interventions^21,96^. Conflict and governance contexts are captured by political stability, violence and conflict indices, proportions of refugees and internally displaced people, justice system metrics (unsentenced detainees, property rights, press freedom), and governance indicators (public spending, affordability of justice), reflecting how crises and instability disrupt health services, surveillance, and stewardship efforts^21^.

### Domain: action areas; subdomain 11: stewardship

Stewardship refers both to indicators that measure the comprehensiveness of efforts to reduce inappropriate AMU, such as stewardship programmes and guidelines for antibiotic use in human, animal, and environmental (aquaculture) health, and consumption rates and sales of antibiotics. A total of 468 indicators were extracted from 78 academic studies^21,22,24,26,30,32,34,36,40,41,51,53,55,56,60,65,66,79,80,86,90–95,97,100,101,103,105,113,114,116,118–160^; 297 from 31 grey literature studies^11,14,18,44,46,48,49,63,68,69,71,77,83,107,109,161–170^, with 163 indicators originating from the 9th Annual Report on Antimicrobial Agents Intended for Use in Animals (ANIMUSE)^48^.

Key indicators for Stewardship assess whether NAPs include One Health stewardship frameworks, guidelines, and dedicated governance structures (e.g., antimicrobial stewardship (AMS) committees, AMS focal points, drug and therapeutics committees, auditing and feedback mechanisms)^28,36,103,129,147^. Human-sector consumption metrics encompass defined daily dose (DDD) and DDD/1000/day (DID) in hospital and community settings, stratified by antibiotic class, access, watch, reserve (AWaRe) categories, age groups, syndromes, and seasonal or regional variation^63,86,94,101,103,125,127,128,130,143,155,164^. Additional indicators include antibiotic treatment courses per country population,^103^, percentage of hospitals in the country with early switch/discharge protocols^97^, prescribing rates per consultation or population^103^, percentage of prescriptions aligned with guidelines^146^, and metrics of outpatient vs. inpatient antibiotic use^130^.

Animal-sector indicators cover antimicrobial consumption^68,165^, antimicrobial consumption by Antimicrobial Expert Group (AMEG) categorisation^170^, treatment incidence per flock or per production round^100,128,150^, proportions of classes used^121^, routes of administration^171^, traceability of medicated feed^119^, classification of veterinary antimicrobials per WOAH/WHO importance lists^18,119^, targets for AMU reduction^168^, and projections of future consumption^139,140^. Environmental stewardship indicators include monitoring and reduction of antibiotic discharges^97,143^, biosecurity practices to optimise AMU^11^ and minimisation of antimicrobial pesticide use^11,14,71^. Broader metrics capture national monitoring systems for sales/use in human and animal sectors^11,77^, adoption of AWaRe classification in essential medicines lists^28,79,103,136,151^, capacity for AMU monitoring^30^, interaction of livestock antibiotic consumption with GDP^113^, and perceived contributions or achievable reductions in sector-specific AMU^90^. Surveillance and evaluation indicators include variation in antibiotic use (DDD/1000/day), trends in consumption rates and costs (EUR/DDD), benchmarking of antibiotic use, and correlation analyses over time^90,127,131^. Stewardship implementation metrics also cover rapid diagnostic test availability, feedback on prescriptions, AMS resource presence, training and behaviour-change methodologies, audit and quality improvement activities, integration of AMS into facility accreditation, and feedback loops for prescribers^103,142,147^.

### Domain: action areas; subdomain 12: community awareness & enabling behaviours

Community awareness and enabling behaviours encompass indicators designed to improve knowledge, shape attitudes, and foster practices that support responsible AMU, including the comprehensiveness and consistency of public awareness campaigns, including educational programmes at school. Ninety-one indicators were extracted from 18 academic studies^21,22,24,27,28,30,31,40,51,56,66,91,104,117,138,147,172,173^, with 38 indicators originating from a single study^31^; 18 across 11 grey literature studies^11,15,17,43,49,62,71,77,111,164,168^.

Indicators focused on educational metrics (e.g., students’ participation rates in prudent antibiotic use campaigns and the core concepts of curriculum used^11,173^). Others measured public awareness campaign output (e.g. counts of education activities, and the variety of materials produced^24,31^). Knowledge and attitude assessments are reflected in government- or survey-derived awareness and understanding scores, personal and political knowledge, attitude, and practice scores, perceived awareness of interconnected issues (climate change, food security, AMR), and perception of priority areas for action^24,56,117,172^. Indicators also track engagement in national and international campaigns and the existence of formal communication strategies^12,30,147^.

Behavioural change metrics include self-reported antibiotic use patterns, public perception of benefits and worries about AMR, and adoption of recommended practices such as obtaining antibiotics by prescription^12,49,111^. Capacity-building indicators assess countries’ ability to raise AMR awareness, measured by Global One Health Index (GOHI)-AMR improved awareness and understanding scores and the presence of incentives to promote and monitor public engagement^21,138^. Finally, stakeholder mapping and community engagement metrics gauge the number of identified and engaged actors, jointly developed risk communication activities, and the proportion of activities adopted by communities^12^.

### Domain: action areas; subdomain 13: workforce

Workforce encompasses indicators referring to the development, education, and training of teams and individuals across the One Health sectors. Indicators in this subdomain include staffing and the existence of an overarching, coherent workforce strategy. A total of 77 indicators were extracted from 22 academic studies^21,22,27,28,30–32,40,53,60,66,90,91,95,99,102,115,119,133,141,174,175^; 63 from 10 grey literature studies^11–13,15,17,43,49,69,77,108^, with over half originating from IPCAT2^13^.

Academic measures include participation in laboratory training and national training modules, the existence of pre-service and in-service pharmacovigilance curricula, and mandatory IPC training for healthcare workers^99,104^. Other staffing-level indicators capture the proportion of the employed population working in agriculture or animal husbandry, ratios of IPC personnel, and estimated numbers of pharmacists and technicians per country^32,95,115^. Some indicators measure competency and preparedness via stakeholder knowledge, perceptions, and practices toward AMS^90,174,175^; education quality is also tracked^11,21,31^. Performance analyses such as correlations between hospital transmission rates and numbers of healthcare workers or practising nurses per 100,000 population that highlight the impact of staffing levels on infection control are also included^141^. National assessment tools such as GOHI education scores benchmark country progress in workforce development and continuous professional education^23,45,69^. These indicators further track the number of professionals trained in joint risk assessment and the integration of AMR content into medical and veterinary curricula^13,43,49^. Lastly, indicators encompass IHR workforce readiness during public health emergencies^12^.

### Domain: action areas; subdomain 14: access to medicines & health services

Access to Medicines & Healthcare Services captures the extent to which the population experiences barriers when accessing healthcare services and essential medicines. Seventy indicators were extracted from 19 academic studies^21,51,84,86,92,96,101,103,115,120,134,141,176–182^; 48 from 11 grey literature studies^11,15,44,49,61,64,72,83,106,112,167^. The majority of grey literature indicators (38) were from Universal Health Coverage (UHC) Watch^64^. Academic indicators include both financial metrics and utilisation measures.

Financial metrics include total health expenditure as a percentage of GDP, the ratio of public to private health spending, out-of-pocket payments as a share of national income, and cost estimates per infection^13,76^. Utilisation measures here include the proportion of children with cough or fever who visit formal versus informal healthcare providers and the estimated cumulative number of facility visits before age five^120^. Service capacity within this subdomain is gauged by hospital bed density, ICU and IMCU beds per 100,000 inhabitants, hospital admissions per year, numbers of registered medicine outlets, and days antibiotics are out of stock^51,84,86,134,179,180^. Essential medicines access is tracked via the count of countries approving the WHO essential medicines list ‘Access’, ‘Forgotten’, and pediatric formulations, while GOHI scores assess domestic health expenditure, health service coverage, and UHC service indices for infectious diseases^21,182^. Grey literature indicators complement these with measures of financial protection, e.g., households facing catastrophic or impoverishing health spending by consumption quintile; unmet need for healthcare and dental care due to cost, distance, or waiting time; and system resilience metrics (e.g., public spending on health as a share of government and GDP, occupancy rates of acute-care beds, and veterinary service strength)^64^. Trusted and utilised health services under this subdomain are gauged by outpatient contact rates and the continuity of essential health services^12^.

### Domain: action areas; subdomain 15: research, innovation & digital technology

Research, Innovation & Digital Technology includes indicators that assess whether there is investment in research to examine the drivers of AMR and the effectiveness of interventions to mitigate AMR. This subdomain includes indicators that measure whether incentives exist, in the public and private sectors, that aim to foster innovation with respect to the development of diagnostics and treatments for AMR. Twenty-eight indicators were extracted from 14 academic studies^21,22,27,31,34,36,51,53,60,66,80,93,183,184^; 22 from 6 grey literature studies^15,49,62,69,77,112^.

In the case of AMR-related research, different academic studies generated AMR research scores through assessment of countries’ commitment to research and development (R&D) through the development of novel agents, presence of a research budget, and reference to R&D within the NAP. These scores were variously presented (e.g. quantitative^22^, qualitative^53^, ordinal, and binary measures)^27,34^. Certain indicators evaluate the provenance^15,80^, and amount^80^, of funding made available for AMR-related research while others assess the type (e.g. push incentives^15^) and scope of this funding^68,82^. Studies also consider the adequacy of AMR-related research by measuring the national number of researchers in the AMR field^21^, the number of publications affiliated with a given country^31,51^, whether these publications address issues such as irrational AMU^183^, and whether relevant scientific or professional conferences are held^31^. The grey literature in particular focuses on structural factors related to AMR research, e.g. the extent to which national research agendas are informed by global AMR agendas and tailored to national needs^49^ and the degree to which capacity building for the implementation of such research is the product of the collaboration between academia, the private sector, and civil society^49^.

### Domain: monitoring & evaluation; subdomain 16: surveillance & laboratory

Surveillance & Laboratory encompasses indicators that assess the comprehensiveness of surveillance programmes and the capacity and quality of laboratory systems across all sectors. A total of 941 indicators were extracted from 115 academic studies^21,22,26,27,29,30,40,51,53,55,57–60,65,74,80,84,87,91,92,94–96,99,101–105,113,121,124–126,132–134,138,141,144,145,147,148,153,154,160,176–180,185–243^; 486 from 29 grey literature studies^11–15,17,18,43,44,46,47,49,61,63,69–71,73,77,106,107,109,112,161–165,244^. Over half of the grey literature indicators were from the WHO Global AMR and Use Surveillance System (GLASS)^63^ (207) and IPCAT 2^13^ (111).

Academic indicators related to surveillance^21,74,99,138,153^, the representativeness and coverage of surveillance platforms^104^, prioritised pathogens or infections^214^, data completeness and consistency, standardised case definitions^104^, sampling schemes^53,216^, data collection intervals^193,230^, data verification and validation processes^27,102,190^, and the establishment and integration of surveillance across One Health sectors^21^. Measures of laboratory capacity and quality span assessments of infrastructure and operations (e.g., availability of national reference laboratories, continuity of services, referral pathways, sample transport)^21,53,74^, diagnostic methods and techniques (e.g., culture, antimicrobial susceptibility testing (AST) for bacteria and fungi, molecular/genomic testing, pathogen-specific assays)^86^, standardisation of procedures (e.g., adherence to Clinical and Laboratory Standards Institute and European Committee on Antimicrobial Susceptibility Testing guidelines, participation in external quality assurance/proficiency testing schemes)^53,57,224,233^, data management, and analysis capabilities (e.g., electronic reporting platforms, and interoperability)^92,113,153,197,214,234^. Many indicators quantify network attributes such as the number and distribution of surveillance sites^194,202,217,235^, coverage of sentinel sites or industry monitoring systems^58,104^, integration of peripheral and reference labs^53,74^, and the establishment of national coordinating centres or integrated surveillance strategies^21,138^. Quality-related indicators include completeness and timeliness of AST data, frequency and methods of data analysis and dissemination, and metrics on external quality assurance participation^27,53,197^.

Surveillance output indicators encompass pathogen-specific resistance rates and incidence densities^134,194,223,232^, bloodstream infection incidence by age, sex, and clinical setting^104,187,202,205^, genotype prevalence and molecular epidemiology (e.g., sequence types, resistance genes, virulence determinants)^203,206^, environmental surveillance metrics (e.g., antimicrobial resistance gene (ARG) abundance in water, wastewater, food chain)^21,30,228^, and outcome measures (e.g. deaths and disability-adjusted life years (DALYs) attributable to AMR)^103,104,205^. Further indicators capture temporal trends^201,206,235^, correlations between resistance prevalence and antimicrobial consumption^96,141^, blood culture positivity rates^218^, and burden estimates^163^. Grey-literature indicators emphasise the establishment of integrated AMR surveillance systems^11,69,70^, laboratory network robustness^11,12,17,70,77^, essential diagnostics lists^11,69^, external quality assessment implementation^11,73^, AST capacity for critical pathogens^11,13^, and reporting to GLASS^63^. Additional measures assess the presence of sustainable specimen referral and transport systems, biosafety/biosecurity frameworks, laboratory quality management systems, tiered diagnostic network implementation, and early warning surveillance protocols^12,69^.

### Domain: monitoring & evaluation; subdomain 17: reporting

Reporting refers to mechanisms for communicating progress and data on the national AMR strategy to stakeholders, including annual progress reports and formal submissions to international and regional AMR surveillance initiatives. Fifty-six indicators were extracted from 18 academic studies^22,27,29,34,53,66,67,75,78,99,104,128,135,153,190,194,197,204^; 85 from 12 grey literature studies^11,13,15,18,43,45,48,63,71,77,107,244^, with 39 indicators originating from ANIMUSE^48^.

Indicators grouped under Reporting address the existence of governance structures or processes for sharing AMR and AMU data with global and regional databases and regulatory bodies (e.g., evidence of protocols for submission to GLASS, Central Asian and European Surveillance of Antimicrobial Resistance network (CAESAR), European Centre of Disease Control (ECDC), European AMR Surveillance Network (EARS-Net), WHO reports, WOAH AMU/AMR databases, European Food Safety Authority (EFSA), European Surveillance of Veterinary Antimicrobial Consumption (ESVAC))^67,78^. Animal health indicators cover registration and reporting of antimicrobial products, submission of AMU and residue data, and integration within One Health reporting frameworks, bacteria–drug combinations, and core data elements^11,18,48,53,77,128^. Laboratory reporting measures include counts of culture-positive/negative results, proportions of commonly reported pathogens, and frequency of AST result submission^197,204^. Grey-literature indicators emphasise reporting NAP status updates^11,15,44,49^, annual animal-sector AMU reports to WOAH^11,18,61^, participation in GLASS^15,45,63,71,77^, and the establishment of clear reporting pathways for AMR and AMU information^13^.

### Domain: monitoring & evaluation; subdomain 18: feedback mechanisms

Feedback mechanisms refer to indicators that capture the existence and quality of processes for relaying surveillance data and performance information to regional, organisational, and individual stakeholders. Sixteen indicators were extracted from 6 academic studies^21,22,53,59,80,104^; 50 from 13 grey literature studies^11,13,17,43,44,49,61,69–71,77,81,108^.

Indicators sought to measure scoping themes from national performance to regional- and hospital-level IPC and stewardship. Sector-specific feedback processes include the establishment of national platforms for sharing pathogen-specific data^13,104^. Multisector coordination indicators examine whether data analysed are used for policy advocacy and resource allocation, whether data reviews occur, and whether AMU and AMR surveillance data inform decision-making and policy^11,13,69,71^. Metrics of zoonotic events (infection transmission between animal and human species) include the proportion of events evaluated with timeliness metrics or decision tools, joint investigations, and activities implemented based on joint risk assessments^43^. Key indicators examine the documented use of surveillance data for decision-making, inclusion of monitoring mechanisms in NAPs, and governance responsiveness scores^11,21^.

Monitoring and audit frameworks are evaluated for integration of IPC indicators within national systems, tools for systematic data collection (e.g., WHO hand hygiene self-assessment), alignment of national and local monitoring activities, linkage with WASH monitoring, regular collection of IPC monitoring and audit data, facility-level self- or peer- evaluation against standards, and dissemination of monitoring results to drive improvement^13,43^. Laboratory feedback is measured by participation in external quality assurance/proficiency testing schemes and whether alternate bodies perform equivalent functions^70^. Indicators also assess whether treatment guidelines undergo regular audit, review, and dissemination, and whether collected data guide action and guideline updates^49,71^. Other indicators measure whether surveillance results are systematically distributed to field actors via organised channels^17^. Indicators also gauge whether veterinary laboratory quality management systems and risk-analysis capacity routinely inform risk management and communication^61^. Finally, the presence of national bodies that review surveillance findings and translate them into recommendations and implemented actions captures the end-to-end feedback process^77^.

Several IPC-related indicators were jointly grouped as feedback mechanism indicators, including the existence of strategic plans for monitoring and feedback, schedules for guideline review, national systems for monitoring adherence with periodic audits, and timely feedback reports to stakeholders on HAI trends, outbreak control, and multidrug-resistant pathogen occurrences^13,43^.

### Domain: monitoring & evaluation; subdomain 19: effectiveness

Effectiveness indicators examine whether structured mechanisms exist at the national level to evaluate if policies or interventions achieve their intended health and economic outcomes. Nineteen indicators were extracted from nine academic studies^20–22,27,28,34,36,91,133^; 17 from 8 grey literature studies^11,13,18,77,109,110,165,245^.

Indicators include metrics, e.g., M&E plan quality by country, aggregate M&E scores, and national evaluation frameworks for IPC and AMR strategies^13,22,34^. Governance effectiveness and efficiency are measured via global governance indices; food safety evaluation scores capture the impact of interventions in related sectors across countries^21,22^. Perception-based metrics include respondents’ satisfaction with the functioning of AMR multisector coordination committees and perceived impact from policy measures^28,91^. Outcome indicators quantify health and economic burdens attributable to AMR, e.g. loss of life expectancy, excess health expenditure, reduced labour participation and productivity due to AMR and broader risk indices^245^. Burden-of-disease measures such as DALYs attributed to AMR serve to assess the ultimate impact of interventions^77^. Certain indicators also attempt to measure the cost-effectiveness of policies through comparisons between intervention costs and observed reductions in AMR-related morbidity, mortality, or economic losses^13,245^.

## Discussion

Our scoping review offers a comprehensive, cross‑sectoral inventory of AMR indicators, drawing from quantitative and qualitative sources across the One Health sectors. By systematically cataloguing over 3000 indicators from academic and grey literature, we fill a critical gap in the AMR evidence landscape and provide a foundation for harmonised, One Health-oriented monitoring.

Our findings demonstrate the structural imbalance in indicator development, with limited coverage of Accountability, Transparency, and Equity subdomains, despite their central importance to the Governance & Leadership domain. Strengthening measurement within these areas is essential for effective policy governance, to ensure AMR strategies are properly developed, then implemented, evaluated, and iteratively improved. The paucity of equity-focused indicators is particularly concerning, given recognition that the burden of AMR falls disproportionately on marginalised, disadvantaged populations^246^. This imbalance extends across One Health sectors: far more indicators are available for human than for animal and environmental health (Supplementary Fig. 1). This lack likely contributes to animal and environmental health’s underrepresentation in national AMR agendas, weakening the multisectoral coordination required for effective AMR control. These findings suggest two priority actions for international organisations such as the Quadripartite and partners: first, develop standardised indicators for underrepresented domains—particularly Accountability, Equity, and Transparency; second, develop indicators for the animal and environmental health sectors comparable to those for human health.

Our work has several limitations. First, we focused our attention on collating existing qualitative and quantitative indicators, but a critical appraisal of the indicators identified fell outside the remit of this review. Further work is planned to use these indicators as input for a consensus process to develop an index that will benchmark national performance in responding to AMR. Second, the comparative, cross‑country inclusion criteria that we used necessarily deprioritised subnational and local indicators, e.g. regional biosecurity protocols and community‑level stewardship initiatives. However, many regional indicators may be relevant to a given country’s specific context, though deemed irrelevant to comparing cross-national performance. Third, restricting our inclusion criteria to studies published between 2014 and 2024 may have excluded earlier efforts to measure national AMR response. However, we deemed studies published pre-2014 as less policy-relevant as they precede the publication of the GAP on AMR. Fourth, our initial focus on English-language publications, subsequently supplemented by non-English studies, may have introduced bias: non-English literature may emphasise different aspects of AMR policy. Fifth, the academic evidence base disproportionately reflects European and high-income country contexts, with 37% of included studies situated in Europe and only 11% in the WHO African Region. This may mean we have captured indicators predominantly from countries with more developed surveillance systems, as these are more likely to report findings publicly, and that the resulting indicator set reflects structural biases in the literature rather than a balanced global perspective. This should be considered when interpreting the indicators and the planned One Health AMR Accountability Index.

Building on the inventory compiled in the current study, we plan to use these indicators to build a novel One-Health AMR Accountability Index to measure national performance in addressing AMR^247^. Our next step involves screening the indicators identified for inclusion in a Delphi survey. Indicators will be formally screened and prioritised based on feasibility, data availability, and subdomain balancing. Indicators will be removed if the overall theme is duplicated or collinearity is identified. After indicator refinement, we will undertake a multi-stage consensus process with international AMR experts to select and weight indicators for inclusion into a unified AMR Accountability Index. The One Health AMR Accountability Index will be piloted in different international regions to reflect regional differences and different country income classifications.

As a separate exercise, we have created a human-health-focused AMR Accountability Index in conjunction with WHO Europe, anchored in the Roadmap on Antimicrobial Resistance for the WHO European Region 2023–2030. This index will provide composite indicators that cover the Roadmap’s five action areas and six enablers spanning both multisectoral and human health domains. Currently, the human-health-focused AMR Accountability Index is undergoing a piloting process in six WHO European region Member States to ensure the Index reflects structural differences and country contexts. Ultimately, One Health AMR Accountability Index seeks to fill a current gap in global AMR policy efforts by benchmarking national performance, identifying countries requiring technical support, and tracking global progress toward AMR containment goals.

## Methods

### Literature review

A scoping approach was employed to review and synthesise existing literature to identify and consolidate quantitative and qualitative measures used to assess and compare national performance in addressing AMR. This review encompassed academic and grey literature searches. The academic literature search was conducted between 26 November 2024, and March 2025. Three electronic databases, Medline, EMBASE, and Global Health, were systematically searched to capture peer-reviewed articles in English from 2014 through 2024. This was supplemented by an additional search using the same search terms for non-English studies in January 2025. Where necessary, non-English studies were translated using Google Translate to assess eligibility and extract relevant data. The starting year of 2014 was chosen to coincide with the release of key global policy instruments (e.g., the World Health Assembly resolution that paved the way for the 2015 WHO GAP on AMR), ensuring that pivotal developments in AMR policy and measurement were captured. In parallel with the academic literature search, a thorough grey literature review was undertaken to capture indicators that might not appear in peer-reviewed journals. Grey literature was identified through Google Scholar and targeted searches of major international organisations. Our methods are further outlined within a previously published PROSPERO protocol (CRD42024625477)^248^.

### Academic literature search terms

The search strategy combined terms related to antimicrobials (e.g., “antibiotic*,” “antimicrobial*,” “antifungal*,” “antiinfective*”), resistance (e.g., “resistance,” “resistant”), and policy or performance measures (e.g., “performance,” “benchmark,” “indicator*,” “evaluation,” “governance,” “framework,” “policy,” “rate*”). Further terms specifying national and governmental contexts (e.g., “national,” “country,” “government*”) were included. Boolean operators (AND, OR) were used to generate comprehensive results while minimising irrelevant studies.

### Grey literature search details

For Google Scholar, we screened the first 300 results sorted by relevance for each search string. The 300-result cap was used to balance sensitivity and feasibility, as relevance markedly declines beyond this range in Google Scholar indexing^1^. Second, relevant websites of major multilateral organisations involved in AMR policy, such as the World Health Organization (WHO)^2^, the World Organisation for Animal Health (WOAH)^3^, the Food and Agriculture Organization (FAO)^4^, the United Nations Environment Programme (UNEP)^5^, the Organisation for Economic Co-operation and Development (OECD)^6^, and the World Bank^7^, were examined to identify policy documents, reports, and white papers listing or discussing AMR indicators. Additionally, websites of prominent think tanks and professional societies (e.g., the Centre for Global Development, the Alliance for Reducing Microbial Resistance, and the European Society of Clinical Microbiology and Infectious Diseases) were searched for relevant non-peer-reviewed materials. The full list of all grey literature sources with links is listed in Supplemental Table 1. All grey literature records were screened and extracted by two reviewers (BH and EO) manually using the same inclusion criteria and data charting procedures applied to the academic literature. All identified documents were saved in a shared repository, and duplicates were removed.

### Inclusion and exclusion criteria

Inclusion criteria were deliberately broad to capture any cross-country comparison of AMR policy or performance that presented either quantitative or qualitative indicators. Inclusion criteria included any article using indicators to measure national performance in addressing AMR. Exclusion criteria included: 1) Wrong study outcome (articles relevant to antivirals, tuberculosis, or vaccines). Tuberculosis was excluded on the basis that TB-specific AMR operates within a largely separate policy, surveillance, and programmatic infrastructure with dedicated international frameworks and accountability structures, and its inclusion risked conflating two distinct policy domains; 2) wrong study design (systematic reviews that identify individual studies in multiple countries focused on developing understanding of AMR, systematic reviews, organisational or regional-level studies of prevalence of AMR or AMU, cost-effectiveness studies, or editorials or conference abstracts); and 3) wrong study comparator (articles measuring regional, organisational, or individual performance and articles used to measure performance in one country in isolation). As the primary purpose of this review was to identify indicators suitable for cross-national benchmarking, single-country studies may reflect idiosyncratic national priorities or data systems that cannot be meaningfully standardised or compared across diverse country contexts.

Two reviewers (US, SR) screened titles and abstracts for relevance, each working separately to apply inclusion and exclusion criteria independently. Any discrepancies were resolved through iterative discussions or by consulting a third reviewer (MA) if consensus could not be reached. Studies that met inclusion criteria at the abstract level were retained for full-text review, after which the same reviewers independently confirmed eligibility with discrepancies again resolved through iterative discussions or by consulting a third reviewer (MA) if consensus could not be reached. Cohen’s Kappa was reported to assess inter-rater reliability for both title and abstract screening as well as full text screening.

### Data extraction and thematic synthesis

All peer-reviewed articles and grey literature documents included were collated into a database. Basic bibliographic information (author(s), publication date, title) and details about the identified indicators (description, measurement approach, data sources used, and One Health domain coverage, which included animal health, environmental health, human health, and multisectoral coordination) were recorded for each. Indicators with sectoral overlap were categorised across animal health, environmental health, and human health, depending on the nature of the indicator. For example, indicators relating to meat production, livestock, or animal products were captured under animal health, while indicators relating to food safety, foodborne disease, or human dietary exposure were captured under human health. Indicators relating to soil contamination, agricultural runoff, or AMR in environmental systems were captured under environmental health. If an indicator had multiple metrics or definitions, each variant was noted separately to ensure a comprehensive initial pool of potential measures.

**Fig. 4:**
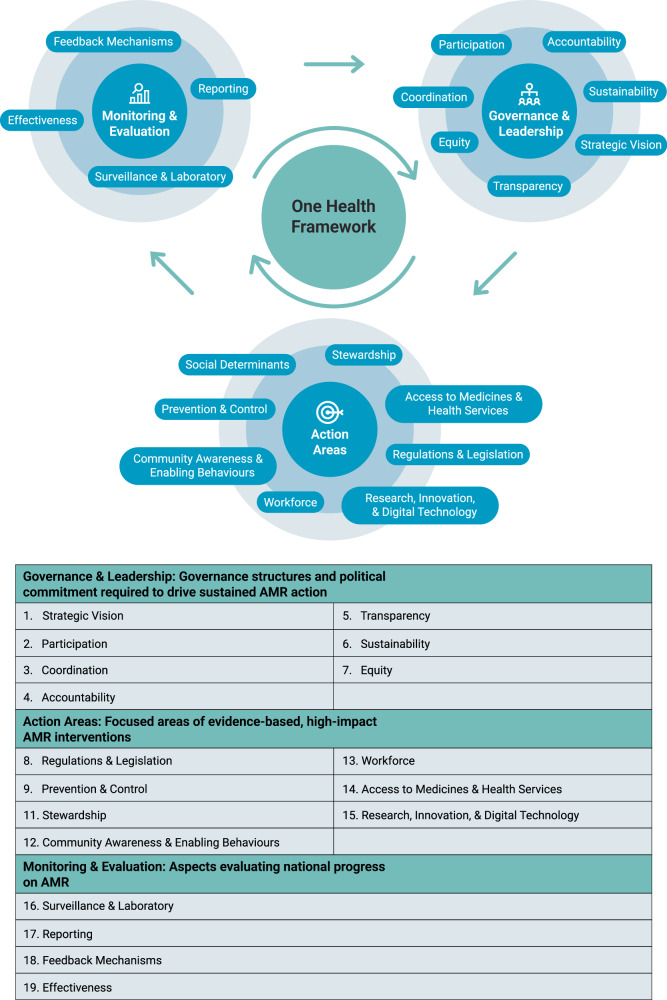
Thematic synthesis of AMR policy domains. Authors’ creation.

A narrative thematic synthesis was then performed. We employed a framework-based approach, adapting existing frameworks (Roadmap on Antimicrobial Resistance for the WHO European Region 2023–2030^6^ and Anderson et al.^16^) to identify relevant domains a priori. We then inductively refined these categories based on the indicators identified. We merged these frameworks to identify 19 AMR subdomain groups under three domains: Governance & Leadership, Action Areas, and Monitoring & Evaluation (Fig. 4).

### Indicator screening and coding

Indicators were excluded if not considered cross-national measures of performance in AMR response, were duplicates (i.e., were identical or had near-identical constructs and measures); not specific to AMR. Four reviewers (US, VK, BH, and SR) independently conducted initial indicator exclusions; two reviewers (MA and EO) evaluated their accuracy and consistency.

Two reviewers coded each indicator according to the framework’s policy domains (Fig. 4); MA and EO reviewed the coding to ensure concordance in approach. Indicators were then examined within each thematic category to highlight commonalities and identify overlap. Finalised extracted indicators must: (1) measure national performance in addressing AMR in two or more countries; (2) be quantitative or qualitative within the scope of AMR; (3) measure performance in any sector across the One Health spectrum; (4) be relevant to AMR.

## Supplementary information

Supplementary Information

## Data Availability

All data generated or analysed during this study are included in this published article and its supplementary information files.

## References

[1] Naghavi, M. et al. Global burden of bacterial antimicrobial resistance 1990–2021: a systematic analysis with forecasts to 2050. *Lancet* **404**, 1199–1226 (2024). 39299261 10.1016/S0140-6736(24)01867-1 PMC11718157

[2] Global Leaders Group on Antimicrobial Resistance. *Towards specific commitments and action in the response to antimicrobial resistance* https://www.amrleaders.org/about-us/what-we-do/glg-report (2024).

[3] World Health Organization. *European Strategic Action Plan on Antibiotic Resistance* (WHO Regional Office for Europe, 2011).

[4] European Commission. *Action Plan Against the Rising Threats from Antimicrobial Resistance* (European Commission, 2011).

[5] Health Emergency Preparedness and Response Authority. *UNGA Political Declaration: A global commitment to combat antimicrobial resistance (AMR)* (European Commission, 2024).

[6] World Health Organization. *Roadmap on antimicrobial resistance for the WHO European Region 2023–2030* (WHO Regional Office for Europe, 2023).

[7] Tejpar, S., Rogers Van Katwyk, S., Wilson, L. & Hoffman, S. J. Taking stock of global commitments on antimicrobial resistance. *BMJ Glob. Health* **7**, e008159 (2022). 35589150 10.1136/bmjgh-2021-008159 PMC9121412

[8] European Commission. *A European One Health Action Plan against Antimicrobial Resistance (AMR)* (European Commission, 2017).

[9] Mendelson, M. et al. Ensuring progress on sustainable access to effective antibiotics at the 2024 UN General Assembly: a target-based approach. *Lancet* **403**, 2551–2564 (2024). 38797179 10.1016/S0140-6736(24)01019-5

[10] Chatterjee, A. et al. Quantifying drivers of antibiotic resistance in humans: a systematic review. *Lancet Infect. Dis.* **18**, e368–e378 (2018). 30172580 10.1016/S1473-3099(18)30296-2

[11] World Health Organization. *Tracking AMR Country Self-assessment Survey - TrACSS* (World Health Organization, 2025).

[12] World Health Organization. *IHR (2005) States Parties self-assessment annual reporting tool* 2nd ed. (World Health Organization, 2021).

[13] World Health Organization. *Instructions for the national infection prevention and control assessment tool 2 (IPCAT 2)* (World Health Organization, 2017).

[14] World Health Organization. *Joint External Evaluation Tool: International Health Regulations (2005)* 3rd ed. (World Health Organization, 2022).

[15] Global Coalition on Aging & Infectious Diseases Society of America. *2024 AMR Preparedness Index Progress Report* (Global Coalition on Aging, 2024).

[16] Anderson, M., Schulze, K., Cassini, A., Plachouras, D. & Mossialos, E. A governance framework for development and assessment of national action plans on antimicrobial resistance. *Lancet Infect. Dis.* **19**, e371–e384 (2019). 10.1016/S1473-3099(19)30415-3 31588040

[17] LLatornico, F., Keck, N., Treilles, M. & Kabali, E. *FAO Assessment Tool for Laboratories and AMR Surveillance Systems (ATLASS) for the food and agriculture sectors* (Food and Agriculture Organization of the United Nations, 2018).

[18] World Organisation for Animal Health. *Implementation of WOAH Standards: The Observatory Annual Report*. https://www.woah.org/en/what-we-do/standards/observatory/implementation-of-standards-the-observatory-monitoring-report/ (2022).

[19] Anderson, M., Kluge, H. H. P., Wong, D. L. F., Butler, R. & Mossialos, E. Promoting sustainable national action to tackle antimicrobial resistance: a proposal to develop an antimicrobial resistance accountability index. *Lancet Microbe* **5**, e304–e311 (2024). 10.1016/j.lanmic.2024.100997 39341218

[20] Birgand, G. et al. Comparison of governance approaches for the control of antimicrobial resistance: Analysis of three European countries. *Antimicrob. Resist. Infect. Control* **7**, 28 (2018). 29468055 10.1186/s13756-018-0321-5 PMC5819189

[21] Zhang, Q. et al. How far has the globe gone in achieving One Health? Current evidence and policy implications based on global One Health index. *Sci. One Health* **3**, 100064 (2024). 39077388 10.1016/j.soh.2024.100064 PMC11262257

[22] Patel, J. et al. Measuring the global response to antimicrobial resistance, 2020-21: a systematic governance analysis of 114 countries. *Lancet Infect. Dis.* **23**, 706–718 (2023). 36657475 10.1016/S1473-3099(22)00796-4

[23] Joshi, M. P. et al. Strengthening multisectoral coordination on antimicrobial resistance: a landscape analysis of efforts in 11 countries. *J. Pharm. Policy Pract.* **14**, 27 (2021). 33648589 10.1186/s40545-021-00309-8 PMC7917520

[24] Chua, A. Q., Verma, M., Hsu, L. Y. & Legido-Quigley, H. An analysis of national action plans on antimicrobial resistance in Southeast Asia using a governance framework approach. *Lancet Reg. Health West. Pac.* **7**, 100084 (2021). 34327414 10.1016/j.lanwpc.2020.100084 PMC8315476

[25] Vogeler, C. S. & Parth, A.-M. An elephant in the room? Explaining agenda-setting in antimicrobial resistance policies in 30 European countries. *Soc. Sci. Med.* **356**, 117164 (2024). 39088927 10.1016/j.socscimed.2024.117164

[26] Joshi, M. P. et al. Moving from assessments to implementation: promising practices for strengthening multisectoral antimicrobial resistance containment capacity. *J. Globe Health Rep.* **5**, 7 (2023). 10.1186/s42522-023-00081-6 PMC10101730 37055845

[27] TTegegne H. A. et al. OH-EpiCap: a semi-quantitative tool for the evaluation of One Health epidemiological surveillance capacities and capabilities. *medRxiv*10.1101/2023.01.04.23284159 (2023). 10.3389/fpubh.2023.1053986 PMC10213933 37250092

[28] Yahaya, A. A. et al. Perspectives on the Regional Strategy for Implementation of National Action Plans on Antimicrobial Resistance in the WHO African Region. *Antibiotics* **13**, 202 (2024). 10.3390/antibiotics13100943 PMC11505852 39452210

[29] Okolie, O. J., Igwe, U., Ismail, S. U., Ighodalo, U. L. & Adukwu, E. C. Systematic review of surveillance systems for AMR in Africa. *J. Antimicrob. Chemother.* **78**, 31EP–51EP (2023). 10.1093/jac/dkac342 PMC9780554 36227707

[30] Sabbatucci, M. et al. Tracking progress on antimicrobial resistance by the quadripartite country self-assessment survey (TrACSS) in G7 countries, 2017-2023: opportunities and gaps. *Pharmacol. Res.* **204**, 107188 (2024). 38705262 10.1016/j.phrs.2024.107188 PMC11156590

[31] Earnshaw, S. et al. European antibiotic awareness day: a five-year perspective of Europe-wide actions to promote prudent use of antibiotics. *Eurosurveillance* **19**, 20928 (2014). 25345519 10.2807/1560-7917.es2014.19.41.20928

[32] Kamere, N. et al. Scoping review of national antimicrobial stewardship activities in eight African countries and adaptable recommendations. *Antibiotics* **11**, 1149 (2022). 10.3390/antibiotics11091149 PMC9495160 36139929

[33] Carelli, D. E., Mitsouli, E. T., Ogne, J. B. & Pierre, J. The best laid plans? international governance perspectives in AMR national action plans in Europe. *Eur. J. Public Health* **33**, 682–686 (2023). 37196335 10.1093/eurpub/ckad080 PMC10393504

[34] Samuel, F., Orubu, E., Sutradhar, I., Zaman, M. H. & Wirtz, V. J. Benchmarking national action plans on antimicrobial resistance in eight selected LMICs: Focus on the veterinary sector strategies. *J. Glob. Health* **10**, 020414 (2020). 33110576 10.7189/jogh.10.020414 PMC7568929

[35] Iwu, C. D. & Patrick, S. M. An insight into the implementation of the global action plan on antimicrobial resistance in the WHO African region: a roadmap for action. *Int. J. Antimicrob. Agents* **58**, 106411 (2021). 34371112 10.1016/j.ijantimicag.2021.106411

[36] Kaiser, R. A., Taing, L. & Bhatia, H. Antimicrobial resistance and environmental health: a water stewardship framework for global and national action. *Antibiotics* **11**, 1014 (2022). 10.3390/antibiotics11010063 PMC8773023 35052940

[37] Carelli, D. E., Ogne, J. B. & Pierre, J. Coming of age: governance challenges in updated AMR national action plans in the EU. *Eur. J. Public Health* **34**, 885–889 (2024). 38578613 10.1093/eurpub/ckae067 PMC11430912

[38] Rubin, O. & Munkholm, L. Isomorphic dynamics in national action plans on antimicrobial resistance. *Public Adm. Dev.* **42**, 142–153 (2021). 10.1057/s41271-021-00277-y 33597731

[39] Avello, P. et al. National action plans on antimicrobial resistance in Latin America: an analysis via a governance framework. *Health policy Plan.* **39**, 188–197 (2024). 38179856 10.1093/heapol/czad118 PMC10883663

[40] Bravo, G. et al. SPiNCAR: A systematic model to evaluate and guide actions for tackling AMR. *PloS ONE* **17**, e0265010 (2022). 35271635 10.1371/journal.pone.0265010 PMC8912127

[41] D’Atri, F. et al. Targets for the reduction of antibiotic use in humans in the Transatlantic Taskforce on Antimicrobial Resistance (TATFAR) partner countries. *Eurosurveillance* **24**, 1800339 (2019). 31311620 10.2807/1560-7917.ES.2019.24.28.1800339 PMC6636213

[42] Munkholm, L. & Rubin, O. The global governance of antimicrobial resistance: a cross-country study of alignment between the global action plan and national action plans. *Globaliz. Health* **16**, 109 (2020). 10.1186/s12992-020-00639-3 PMC7656753 33176810

[43] Food and Agriculture Organization of the United Nations, World Health Organization & World Organisation for Animal Health. *Monitoring and Evaluation for Effective Management of Zoonotic Diseases - An operational tool of the Tripartite Zoonoses Guide* (World Health Organization, 2024).

[44] Organisation for Economic Co-operation and Development. *Embracing a One Health Framework to Fight Antimicrobial Resistance* (OECD Publishing, 2023).

[45] United Nations General Assembly. *Political declaration of the high-level meeting on antimicrobial resistance* (United Nations, 2024).

[46] Global Leaders Group on Antimicrobial Resistance. *Priorities of the Global Leaders Group on AMR* (Global Leaders Group, 2023).

[47] World Health Organization. *Surveillance of health care-associated infections at national and facility levels - Practical handbook* (World Health Organization, 2024).

[48] World Organisation for Animal Health. *Annual Report on Antimicrobial Agents Intended for Use in Animals* https://www.woah.org/app/uploads/2024/05/woah-amu-report-2024-final.pdf (World Organisation for Animal Health, 2024).

[49] World Health Organization. *People-centred approach to addressing antimicrobial resistance in human health: WHO core package of interventions to support national action plans* (World Health Organization, 2023).

[50] Food and Agriculture Organization of the United Nations. *Food and Agriculture Organization of the United Nations*. https://www.fao.org/home/en (2026).

[51] Joshi, M. P. et al. Multidisciplinary and multisectoral coalitions as catalysts for action against antimicrobial resistance: Implementation experiences at national and regional levels. *Glob. Public Health* **13**, 1781–1795 (2018). 29557288 10.1080/17441692.2018.1449230

[52] Willemsen, A., Reid, S. & Assefa, Y. A review of national action plans on antimicrobial resistance: strengths and weaknesses. *Antimicrob. Resist. Infect. Control* **11**, 90 (2022). 35739564 10.1186/s13756-022-01130-x PMC9229779

[53] Ferdinand, A. S. et al. Development of a cross-sectoral antimicrobial resistance capability assessment framework. *BMJ Glob. Health* **9**, e014282 (2024). 10.1136/bmjgh-2023-013280 PMC10806917 38232993

[54] Chan, O. S. K. et al. Antimicrobial resistance policy protagonists and processes—a qualitative study of policy advocacy and implementation. *Antibiotics* **11**, 1029 (2022). 10.3390/antibiotics11101434 PMC9598113 36290091

[55] Elton, L. et al. Antimicrobial resistance preparedness in sub-Saharan African countries. *Antimicrob. Resist. Infect. Control* **9**, 145 (2020). 32859252 10.1186/s13756-020-00800-y PMC7456056

[56] Bajalan, A. et al. Awareness regarding antimicrobial resistance and confidence to prescribe antibiotics in dentistry: a cross-continental student survey. *Antimicrob. Resist. Infect. Control* **11**, 158 (2022). 36503570 10.1186/s13756-022-01192-x PMC9741920

[57] Viprey, V. F. et al. European survey on the current surveillance practices, management guidelines, treatment pathways and heterogeneity of testing of Clostridioides difficile, 2018-2019: results from the Combatting Bacterial Resistance in Europe CDI (COMBACTE. *J. Hosp. Infect.* **131**, 213–220 (2023). 36462673 10.1016/j.jhin.2022.11.011

[58] Rahbe, E., Kovacevic, A., Opatowski, L. & Leclerc, Q. J. Investigating the feasibility and potential of combining industry AMR monitoring systems: a comparison with WHO GLASS. *medRxiv*10.1101/2024.03.27.24303768 (2024). 10.12688/wellcomeopenres.21181.2 PMC11452768 39372841

[59] Pallett, S. J. et al. National action plans for antimicrobial resistance and variations in surveillance data platforms. *Bull. World Health Organ.* **101**, 501–512F (2023). 37529028 10.2471/BLT.22.289403 PMC10388141

[60] Cole, M. J. et al. The European response to control and manage multi- and extensively drug-resistant *Neisseria gonorrhoeae*. *Eurosurveillance* **27**, 2100482 (2022). 10.2807/1560-7917.ES.2022.27.18.2100611 PMC9074391 35514307

[61] World Organisation for Animal Health. *Evaluation of the Performance of Veterinary Services PVS Tool – Terrestrial 2019* (World Organisation for Animal Health, 2023).

[62] Food and Agriculture Organization of the United Nations. *Methodology to Analyse AMR-Relevant Legislation in the Food and Agriculture Sector - Guidance Document for Regulators* (Food and Agriculture Organization of the United Nations, 2020).

[63] World Health Organization. *Global Antimicrobial Resistance and Use Surveillance System (GLASS)* (World Health Organization, 2023).

[64] World Health Organization Barcelona Office for Health Systems Financing. *UHC Watch* (World Health Organization, 2024).

[65] Kinoshita, T., Tokumasu, H., Tanaka, S., Kramer, A. & Kawakami, K. Policy implementation for methicillin-resistant Staphylococcus aureus in seven European countries: a comparative analysis from 1999 to 2015. *J. Mark. Access Health Policy* **5**, 1351293 (2017). 28804601 10.1080/20016689.2017.1351293 PMC5533128

[66] Balkhy, H. H. et al. The strategic plan for combating antimicrobial resistance in Gulf Cooperation Council States. *J. Infect. Public Health* **9**, 375–385 (2016). 27106389 10.1016/j.jiph.2016.03.003

[67] Mesa Varona, O. et al. Monitoring antimicrobial resistance and drug usage in the human and livestock sector and foodborne antimicrobial resistance in six European countries. *Infect. Drug Resist.* **13**, 957–993 (2020). 32308439 10.2147/IDR.S237038 PMC7140725

[68] Center for Global Development. *The Commitment to Development Index* (Center for Global Development, 2023).

[69] World Health Organization. *WHO benchmarks for strengthening health emergency capacities* (World Health Organization, 2023).

[70] Food and Agriculture Organization of the United Nations. *The International FAO Antimicrobial Resistance Monitoring (InFARM) system: Manual for implementation – Annex 3* (Food and Agriculture Organization of the United Nations, 2024).

[71] World Health Organization. *WHO implementation handbook for national action plans on antimicrobial resistance: guidance for the human health sector* (World Health Organization, 2022).

[72] World Health Organization. *International Health Regulations (2005): State Party Self-Assessment Annual Reporting Tool* (World Health Organization, 2021).

[73] World Health Organization. *Central Asian and European Surveillance of Antimicrobial Resistance – External quality assessment results 2020* (World Health Organization, 2022).

[74] Albiger, B., Glasner, C., Struelens, M. J., Grundmann, H. & Monnet, D. L. Carbapenemase-producing Enterobacteriaceae in Europe: assessment by national experts from 38 countries, May 2015. *Eurosurveillance* **20**, 21300 (2015). 10.2807/1560-7917.ES.2015.20.45.30062 26675038

[75] Harant, A. Assessing transparency and accountability of national action plans on antimicrobial resistance in 15 African countries. *Antimicrob. Resist. Infect. Control* **11**, 15 (2022). 35073967 10.1186/s13756-021-01040-4 PMC8785006

[76] World Health Organization Eastern Mediterranean Region. *Operational approach to antimicrobial stewardship in the WHO Eastern Mediterranean Region* (World Health Organization, 2024).

[77] World Health Organization, World Organisation for Animal Health & Food and Agriculture Organization of the United Nations. *Monitoring and evaluation of the global action plan on antimicrobial resistance* (World Health Organization, 2019).

[78] Charani, E. et al. An analysis of existing national action plans for antimicrobial resistance-gaps and opportunities in strategies optimising antibiotic use in human populations. *Lancet Glob. Health* **11**, e466–e474 (2023). 36739875 10.1016/S2214-109X(23)00019-0

[79] Gahimbare, L. et al. Monitoring progress on Antimicrobial Resistance (AMR) response in the World Health Organization African region: insights from the Tracking AMR Country Self-Assessment Survey (TrACSS) 2021 results for the human health sector. *J. Public Health Afr.* **14**, 2392 (2023). 38500695 10.4081/jphia.2023.2392 PMC10946299

[80] European Health and Digital Executive Agency, Intellera Consulting & Tetra Tech International Development. *Study on the Design of a Monitoring Framework of the EU One Health Action Plans against AMR and Council Recommendation on Stepping up EU Actions to Combat Antimicrobial Resistance in a One Health Approach*. https://op.europa.eu/en/publication-detail/-/publication/e872ae01-e50d-11ef-bc1c-01aa75ed71a1 (European Health and Digital Executive Agency, 2024).

[81] World Bank & Food and Agriculture Organization of the United Nations. *From reacting to preventing pandemics: Building Animal Health and Wildlife Systems for One Health in East Asia and Pacific* (World Bank, 2022).

[82] Alliance for Reducing Microbial Resistance & G7 Research Group. *G7 Compliance Report on Antimicrobial Resistance, 2021–2023* (Alliance for Reducing Microbial Resistance, 2024).

[83] Council of the European Union. *Council Recommendation on stepping up EU actions to combat antimicrobial resistance in a One Health approach* (Council of the European Union, 2023).

[84] Alsan, M. et al. Out-of-pocket health expenditures and antimicrobial resistance in low-income and middle-income countries: an economic analysis. *Lancet Infect. Dis.* **15**, 1203–1210 (2015). 26164481 10.1016/S1473-3099(15)00149-8 PMC4609169

[85] World Health Organization. *Addressing gender inequalities in national action plans on antimicrobial resistance – Guidance to complement the people-centred approach* (World Health Organization, 2024).

[86] Browne, A. J. et al. Global antibiotic consumption and usage in humans, 2000-18: a spatial modelling study. *Lancet Planet. Health* **5**, e893–e904 (2021). 34774223 10.1016/S2542-5196(21)00280-1 PMC8654683

[87] Medland, N. A. et al. Surveillance systems to monitor antimicrobial resistance in Neisseria gonorrhoeae: a global, systematic review, 1 January 2012 to 27 September 2020. *Eurosurveillance* **27**, 2100917 (2022). 35514308 10.2807/1560-7917.ES.2022.27.18.2100917 PMC9074396

[88] Weets, C. M. & Katz, R. Global approaches to tackling antimicrobial resistance: a comprehensive analysis of water, sanitation and hygiene policies. *BMJ Glob. Health* **9**, e014224 (2024). 10.1136/bmjgh-2023-013855 PMC10900347 38413102

[89] Luthman, O., Robb, D. H. F., Henriksson, P. J. G., Jorgensen, P. S. & Troell, M. Global overview of national regulations for antibiotic use in aquaculture production. *Aquac. Int.* **32**, 9253–9270 (2024).

[90] Postma, M. et al. Opinions of veterinarians on antimicrobial use in farm animals in Flanders and the Netherlands. *Vet. Rec.* **179**, 68 (2016). 27313178 10.1136/vr.103618

[91] Visschers, V. H. M. et al. Perceptions of antimicrobial usage, antimicrobial resistance and policy measures to reduce antimicrobial usage in convenient samples of Belgian, French, German, Swedish and Swiss pig farmers. *Prev. Vet. Med.* **119**, 10–20 (2015). 25684036 10.1016/j.prevetmed.2015.01.018

[92] Ahmad, R., Zhu, N. J., Leather, A. J. M., Holmes, A. & Ferlie, E. Strengthening strategic management approaches to address antimicrobial resistance in global human health: a scoping review. *BMJ Glob. Health* **4**, e001730 (2019). 31565417 10.1136/bmjgh-2019-001730 PMC6747904

[93] Jensen, C. S. While we are waiting for the Superbug: constitutional asymmetry and EU governmental policies to combat antimicrobial resistance. *J. Common Mark. Stud.* **58**, 1361–1376 (2020).

[94] Booth, A. & Wester, A. L. A multivariable analysis of the contribution of socioeconomic and environmental factors to blood culture Escherichia Coli resistant to fluoroquinolones in high- and middle-income countries. *BMC Public Health* **22**, 354 (2022). 35183144 10.1186/s12889-022-12776-y PMC8857829

[95] Opatowski, L., Opatowski, M., Vong, S. & Temime, L. A one-health quantitative model to assess the risk of antibiotic resistance acquisition in asian populations: impact of exposure through food, water, livestock and humans. *Risk Anal.* **41**, 1427–1446 (2021). 33128307 10.1111/risa.13618

[96] Collignon, P., Beggs, J. J., Walsh, T. R., Sumanth Gandra, S. G. & Ramanan Laxminarayan, R. L. Anthropological and socioeconomic factors contributing to global antimicrobial resistance: a univariate and multivariable analysis. *Lancet Planet. Health* **2**, e398 (2018). 30177008 10.1016/S2542-5196(18)30186-4

[97] Eckmann, C. et al. Antibiotic treatment patterns across Europe in patients with complicated skin and soft-tissue infections due to meticillin-resistant Staphylococcus aureus: a plea for implementation of early switch and early discharge criteria. *Int. J. Antimicrob. Agents* **44**, 56–64 (2014). 24928311 10.1016/j.ijantimicag.2014.04.007

[98] Munkholm, L., Rubin, O., Baekkeskov, E. & Humboldt-Dachroeden, S. Attention to the Tripartite’s one health measures in national action plans on antimicrobial resistance. *J. Public Health Policy* **42**, 236–248 (2021). 33597731 10.1057/s41271-021-00277-y

[99] Leong, M., Picton, R., Wratten, M., Mahe, A. & Zimmerman, P.-A. Baseline evaluation of the World Health Organization (WHO) infection prevention and control (IPC) core components in Pacific Island Countries and Territories (PICTs). *Antimicrob. Resist. Infect. control* **13**, 108 (2024). 39334478 10.1186/s13756-024-01447-9 PMC11437787

[100] Caekebeke, N. et al. Comparing farm biosecurity and antimicrobial use in high-antimicrobial-consuming broiler and pig farms in the Belgian-Dutch border region. *Front. Vet. Sci.* **7**, 558455 (2020). 33330687 10.3389/fvets.2020.558455 PMC7673451

[101] Ardakani, Z. et al. Evaluating the contribution of antimicrobial use in farmed animals to global antimicrobial resistance in humans. *One Health* **17**, 100600 (2023). 10.1016/j.onehlt.2023.100647 PMC10665205 38024271

[102] Chiang, C.-H. et al. Healthcare-associated infections in intensive care units in Taiwan, South Korea, and Japan: recent trends based on national surveillance reports. *Antimicrob. Resist. Infect. Control* **7**, 129 (2018). 30455867 10.1186/s13756-018-0422-1 PMC6223041

[103] Funiciello, E. et al. Identifying AWaRe indicators for appropriate antibiotic use: a narrative review. *J. Antimicrob. Chemother.* **79**, 3063–3077 (2024). 39422368 10.1093/jac/dkae370 PMC11638856

[104] Takaya, S. et al. Surveillance systems for healthcare-associated infection in high and upper-middle income countries: a scoping review. *J. Infect. Chemother.* **26**, 429–437 (2020). 32081645 10.1016/j.jiac.2020.01.001

[105] Carmo, L. P. et al. Veterinary expert opinion on potential drivers and opportunities for changing antimicrobial usage practices in livestock in Denmark, Portugal, and Switzerland. *Front. Vet. Sci.* **5**, 29 (2018). 29546044 10.3389/fvets.2018.00029 PMC5837977

[106] United Nations Department of Economic and Social Affairs. *Global indicator framework for the Sustainable Development Goals and targets of the 2030 Agenda for Sustainable Development* (United Nations, 2024).

[107] European Centre for Disease Prevention and Control et al. *Point prevalence survey of healthcare-associated infections and antimicrobial use in European long-term care facilities* (European Centre for Disease Prevention and Control, 2014).

[108] World Health Organization. *Assessment tool of the minimum requirements for infection prevention and control programmes at the national level* (World Health Organization, 2021).

[109] Vivideconomics. *The costs and risks of AMR water pollution* (Vivideconomics, 2020).

[110] World Health Organization & United Nations International Children’s Emergency Fund. *WHO/UNICEF Joint Monitoring Programme for Water Supply, Sanitation and Hygiene (JMP)* (World Health Organization, 2023).

[111] World Health Organization. *Worldwide country situation analysis: response to antimicrobial resistance* (World Health Organization, 2015).

[112] World Health Organization. *Monitoring framework for the WHO Strategic and operational priorities to address drug-resistant bacterial infections in the human health sector, 2025–2035* (World Health Organization, 2024).

[113] Mendelsohn, E. et al. Global patterns and correlates in the emergence of antimicrobial resistance in humans. *Proc. Biol. Sci.* **290**, 20231085 (2023). 37727084 10.1098/rspb.2023.1085 PMC10509571

[114] Zavaleta, E. et al. Antibiotic consumption in primary care in Costa Rica and Italy: a retrospective cross-country analysis. *Cureus* **15**, e41414 (2023). 37546059 10.7759/cureus.41414 PMC10403152

[115] Collignon, P., Athukorala, P.-C., Senanayake, S. & Khan, F. Antimicrobial resistance: the major contribution of poor governance and corruption to this growing problem. *PloS ONE* **10**, e0116746 (2015). 25786027 10.1371/journal.pone.0116746 PMC4364737

[116] Holloway, K. A., Rosella, L. & Henry, D. The impact of WHO essential medicines policies on inappropriate use of antibiotics. *PloS ONE* **11**, e0152020 (2016). 27002977 10.1371/journal.pone.0152020 PMC4803297

[117] Naing, S., van Wijk, M., Vila, J. & Balleste-Delpierre, C. *Understanding antimicrobial resistance from the perspective of public policy: a multinational knowledge, attitude, and perception survey to determine global awareness* (Antibiotics, 2021). 10.3390/antibiotics10121486 PMC8698787 34943698

[118] Padget, M. et al. A community survey of antibiotic consumption among children in Madagascar and Senegal: the importance of healthcare access and care quality. *J. Antimicrob. Chemother.* **72**, 564–573 (2017). 28115503 10.1093/jac/dkw446

[119] Caipo, M., Gatica, M., de, L. A., Rojas, H. & Del Barrio, L. A qualitative approach for a situation analysis of AMR risks in the food animal production sector. *Front. Vet. Sci.* **10**, 1045276 (2023). 36876011 10.3389/fvets.2023.1045276 PMC9978409

[120] Fink, G., D’Acremont, V., Leslie, H. H. & Cohen, J. Antibiotic exposure among children younger than 5 years in low-income and middle-income countries: a cross-sectional study of nationally representative facility-based and household-based surveys. *Lancet Infect. Dis.* **20**, 179–187 (2020). 31843383 10.1016/S1473-3099(19)30572-9

[121] van Bijnen, E. M. E. et al. Antibiotic exposure and other risk factors for antimicrobial resistance in nasal commensal staphylococcus aureus: an ecological study in 8 European Countries. *PloS One* **10**, e0135094 (2015). 26262679 10.1371/journal.pone.0135094 PMC4532423

[122] Andersson, K., van Driel, M., Hedin, K., Hollingworth, S. & Merlo, G. Antibiotic use in Australian and Swedish primary care: a cross-country comparison. *Scand. J. Prim. Health Care* **40**, 95–103 (2022). 35166180 10.1080/02813432.2022.2036494 PMC9090355

[123] Robertson, J. et al. Antimicrobial medicines consumption in eastern europeand central asia - an updated cross-national study and assessment of quantitative metrics for policy action. *Front. Pharmacol.* **9**, 1156 (2018). 30890943 10.3389/fphar.2018.01156 PMC6411709

[124] Ceccarelli, D. et al. Antimicrobial resistance prevalence in commensal Escherichia coli from broilers, fattening turkeys, fattening pigs and veal calves in European countries and association with antimicrobial usage at country level. *J. Med. Microbiol.* **69**, 537EP–547EP (2020). 32186483 10.1099/jmm.0.001176

[125] Gladstone, B. P. et al. Antimicrobial resistance rates in gram-positive bacteria do not drive glycopeptides use. *PloS ONE* **12**, e0181358 (2017). 28727741 10.1371/journal.pone.0181358 PMC5519079

[126] Tomic, T. et al. Antimicrobial utilization and resistance in Pseudomonas aeruginosa using segmented regression analysis: a comparative study between Serbia and eight European Countries. *Int. J. Clin. Pharm.* **45**, 989–998 (2023). 37284904 10.1007/s11096-023-01603-y PMC10246517

[127] Horvat, O. et al. Are there striking differences in outpatient use of antibiotics between South Backa District, Serbia, and Some Scandinavian Countries? *Front. Public Health* **6**, 91 (2018). 29651413 10.3389/fpubh.2018.00091 PMC5884880

[128] Joosten, P. et al. *Assigning Defined Daily/Course Doses for Antimicrobials in Turkeys to Enable a Cross-Country Quantification and Comparison of Antimicrobial Use* (Antibiotics, 2021). 10.3390/antibiotics10080971 PMC8388960 34439021

[129] Suzuki, H. G., Dewez, J. E., Nijman, R. G. & Yeung, S. Clinical practice guidelines for acute otitis media in children: a systematic review and appraisal of European national guidelines. *BMJ Open* **10**, e035343 (2020). 32371515 10.1136/bmjopen-2019-035343 PMC7228535

[130] Scholle, O., Rasmussen, L., Reilev, M., Viebrock, J. & Haug, U. Comparative analysis of outpatient antibiotic prescribing in early life: a population-based study across birth cohorts in Denmark and Germany. *Infect. Dis. Ther.* **13**, 299–312 (2024). 38261237 10.1007/s40121-024-00916-3 PMC10904695

[131] Carmo, L. P. et al. Comparison of antimicrobial consumption patterns in the swiss and danish cattle and swine production (2007-2013). *Front. Vet. Sci.* **4**, 26 (2017). 28303244 10.3389/fvets.2017.00026 PMC5332391

[132] Chantziaras, I., Boyen, F., Callens, B. & Dewulf, J. Correlation between veterinary antimicrobial use and antimicrobial resistance in food-producing animals: a report on seven countries. *J. Antimicrob. Chemother.* **69**, 827–834 (2014). 24216767 10.1093/jac/dkt443

[133] van Dorst, P. W. M. et al. Cost-effectiveness of test-and-treat strategies to reduce the antibiotic prescription rate for acute febrile illness in primary healthcare clinics in Africa. *Appl. Health Econ. Health policy* **22**, 701–715 (2024). 38796659 10.1007/s40258-024-00889-x PMC11338971

[134] Hesstvedt, L. et al. Differences in epidemiology of candidaemia in the Nordic countries - what is to blame? *Mycoses* **60**, 11–19 (2017). 27464892 10.1111/myc.12535

[135] Babu Rajendran, N. et al. EPI-Net One Health reporting guideline for antimicrobial consumption and resistance surveillance data: a Delphi approach. *Lancet Reg. Health Eur.* **26**, 100563 (2023). 36895445 10.1016/j.lanepe.2022.100563 PMC9989632

[136] Jackson, C. et al. Estimating global trends in total and childhood antibiotic consumption, 2011-2015. *BMJ Glob. Health* **4**, e001241 (2019). 30899565 10.1136/bmjgh-2018-001241 PMC6407570

[137] Van Boeckel, T. P. et al. Global antibiotic consumption 2000 to 2010: an analysis of national pharmaceutical sales data. *Lancet Infect. Dis.* **14**, 742–750 (2014). 25022435 10.1016/S1473-3099(14)70780-7

[138] Zhou, N. et al. Global antimicrobial resistance: a system-wide comprehensive investigation using the Global One Health Index. *Infect. Dis. Poverty* **11**, 92 (2022). 35996187 10.1186/s40249-022-01016-5 PMC9395850

[139] Schar, D., Klein, E. Y., Laxminarayan, R., Gilbert, M. & Van Boeckel, T. P. Global trends in antimicrobial use in aquaculture. *Sci. Rep.* **10**, 21878 (2020). 33318576 10.1038/s41598-020-78849-3 PMC7736322

[140] Mulchandani, R., Wang, Y., Gilbert, M. & Van Boeckel, T. P. Global trends in antimicrobial use in food-producing animals: 2020 to 2030. *PLOS Glob. Public Health* **3**, e0001305 (2023). 36963007 10.1371/journal.pgph.0001305 PMC10021213

[141] Kachalov, V. N. et al. Identifying the drivers of multidrug-resistant Klebsiella pneumoniae at a European level. *PLoS Comput. Biol.* **17**, e1008446 (2021). 33513129 10.1371/journal.pcbi.1008446 PMC7888642

[142] Wang, S., Pulcini, C., Rabaud, C., Boivin, J.-M. & Birge, J. Inventory of antibiotic stewardship programs in general practice in France and abroad. *Med. Mal. Infect.* **45**, 111–123 (2015). 25747501 10.1016/j.medmal.2015.01.011

[143] Rubinic, I. et al. Measuring hospital antibiotic consumption in EU/EEA countries: comparison of different metrics, 2017 to 2021. *Euro Surveill.* **29**, 2300433 (2024). 10.2807/1560-7917.ES.2024.29.46.2400317 PMC11565654 39544143

[144] Pontinen, A. K. et al. Modulation of multidrug-resistant clone success in Escherichia coli populations: a longitudinal, multi-country, genomic and antibiotic usage cohort study. *Lancet Microbe* **5**, e142–e150 (2024). 38219757 10.1016/S2666-5247(23)00292-6

[145] McDonnell, L. et al. National disparities in the relationship between antimicrobial resistance and antimicrobial consumption in Europe: an observational study in 29 countries. *J. Antimicrob. Chemother.* **72**, 3199–3204 (2017). 28961862 10.1093/jac/dkx248

[146] Thompson, W. et al. Patterns of dental antibiotic prescribing in 2017: Australia, England, United States, and British Columbia (Canada). *Infect. Control Hosp. Epidemiol.* **43**, 191–198 (2022). 33818323 10.1017/ice.2021.87 PMC9044466

[147] Kerr, F. et al. *Practical Pharmacist-Led Interventions to Improve Antimicrobial Stewardship in Ghana, Tanzania, Uganda and Zambia* (Pharmacy, 2021). 10.3390/pharmacy9030124 PMC8293468 34287350

[148] Cong, W. et al. Prevalence of antibiotic prescribing in COVID-19 patients in China and other low- and middle-income countries during the pandemic (December 2019-March 2021): a systematic review and meta-analysis. *J. Antimicrobial Chemother.* **78**, 2787EP–2794EP (2023). 10.1093/jac/dkad302 PMC10689912 37883697

[149] Bijnen, E. et al. *Primary care treatment guidelines for skin infections in Europe: congruence with antimicrobial resistance found in commensal Staphylococcus aureus in the community* (BMC Family Practice, 2014). 10.1186/s12875-014-0175-8 PMC4220054 25413920

[150] Joosten, P. et al. Quantitative and qualitative analysis of antimicrobial usage at farm and flock level on 181 broiler farms in nine European countries. *J. Antimicrob. Chemother.* **74**, 798–806 (2019). 30649428 10.1093/jac/dky498

[151] Wieters, I. et al. Reported antibiotic use among patients in the multicenter ANDEMIA infectious diseases surveillance study in sub-saharan Africa. *Antimicrob. Resist. Infect. control* **13**, 9 (2024). 38273333 10.1186/s13756-024-01365-w PMC10809765

[152] Ferrer, P. et al. Sales of macrolides, lincosamides, streptogramins, and amoxicillin/clavulanate in the in- and outpatient setting in 10 European countries, 2007-2010. *SpringerPlus* **4**, 612 (2015). 26543747 10.1186/s40064-015-1398-4 PMC4628133

[153] Galia, L. et al. *Surveillance of Antifungal Resistance in Candidemia Fails to Inform Antifungal Stewardship in European Countries* (Journal of Fungi, 2022). 10.3390/jof8030249 PMC8950249 35330251

[154] Klein, E. Y., Tseng, K. K., Pant, S. & Laxminarayan, R. Tracking global trends in the effectiveness of antibiotic therapy using the Drug Resistance Index. *BMJ Glob. Health* **4**, e001315 (2019). 31139449 10.1136/bmjgh-2018-001315 PMC6509601

[155] Benko, R. et al. *Trends in the hospital-sector consumption of the WHO AWaRe Reserve group antibiotics in EU/EEA countries and the United Kingdom, 2010 to 2018* (Eurosurveillance, 2022). 10.2807/1560-7917.ES.2022.27.41.2101058 PMC9562808 36239173

[156] Hillerton, J. E., Irvine, C. R., Bryan, M. A., Scott, D. & Merchant, S. C. Use of antimicrobials for animals in New Zealand, and in comparison with other countries. *N. Z. Vet. J.* **65**, 71–77 (2017). 27030313 10.1080/00480169.2016.1171736

[157] Hsia, Y. et al. Use of the WHO Access, Watch, and Reserve classification to define patterns of hospital antibiotic use (AWaRe): an analysis of paediatric survey data from 56 countries. *Lancet Glob. Health* **7**, e861–e871 (2019). 31200888 10.1016/S2214-109X(19)30071-3

[158] Moura P. et al. Users’ perception of the OH-EpiCap evaluation tool based on its application to nine national antimicrobial resistance surveillance systems. *medRxiv*10.1101/2023.03.15.23287323 (2023). 10.3389/fpubh.2023.1138645 PMC10315896 37404278

[159] Sanchez, M. L. et al. Variability in the community consumption of antibiotics: a problem in Europe, Spain and Asturias. *Le. Infez. Med.* **27**, 134–140 (2019). 31205035

[160] Hagedoorn, N. N. et al. Variation in antibiotic prescription rates in febrile children presenting to emergency departments across Europe (MOFICHE): a multicentre observational study. *PLoS Med.* **17**, e1003208 (2020). 32813708 10.1371/journal.pmed.1003208 PMC7444592

[161] European Food Safety Authority. *Antimicrobial consumption and resistance in bacteria from humans and food-producing animals* (European Food Safety Authority, 2024). 10.2903/j.efsa.2024.8589 PMC10885775 38405113

[162] European Centre for Disease Prevention and Control. *Antimicrobial resistance in the EU/EEA (EARS-Net)* - (Annual Epidemiological Report 2023, 2024).

[163] Anderson, M. et al. Averting the AMR crisis - what are the avenues for policy action for countries in Europe? *Eur. Obs. Health Syst. Policies* (2019). 31287637

[164] One Health Trust. ResistanceMap. *One Health Trust* https://resistancemap.onehealthtrust.org/ (2025).

[165] European Centre for Disease Prevention and Control, European Food Safety Authority & European Medicines Agency. Third joint inter-agency report on integrated analysis of consumption of antimicrobial agents and occurrence of antimicrobial resistance in bacteria from humans and food-producing animals in the EU/EEA. *EFSA J.* **19**, e06712 (2021). 10.2903/j.efsa.2021.6712 PMC8243991 34221148

[166] World Health Organization. Antimicrobial stewardship programmes in health-care facilities in low- and middle-income countries: a WHO practical toolkit. *World Health Organization*. https://www.who.int/publications/i/item/9789241515481 (2019). 10.1093/jacamr/dlz072 PMC8210188 34222945

[167] Anderson, M., Panteli, D. & Mossialos, E. How can the EU support sustainable innovation and access to effective antibiotics? *Policy Brief, No. 51* https://www.ncbi.nlm.nih.gov/books/NBK594073/ (2023). 37582187

[168] Sultanate of Oman Ministry of Health & Sultanate of Oman Ministry of Agriculture, Fisheries Wealth and Water Resources. *Report of the Third High-Level Ministerial Conference on Antimicrobial Resistance* (2022).

[169] Eurostat. Consumption of antibiotics in the community and hospital sectors - defined daily doses (DDD) per day. *Eurostat Database (sdg_03_70)* https://ec.europa.eu/eurostat/databrowser/view/sdg_03_70/default/table (2025).

[170] European Medicines Agency Science Medicines Health. Guideline on the reporting of antimicrobial sales and use in animals at the EU level – denominators and indicators. *EMA/CVMP/882931/2022* (2023).

[171] Robertson, J. et al. Antimicrobial medicines consumption in Eastern Europe and Central Asia – an updated cross-national study and assessment of quantitative metrics for policy action. *Front. Pharmacol.* **9**, 1156 (2019). 10.3389/fphar.2018.01156 PMC6411709 30890943

[172] Farrell, S. et al. A multinational survey of companion animal veterinary clinicians: How can antimicrobial stewardship guidelines be optimised for the target stakeholder? *Vet. J.* **303**, 106045 (2024). 10.1016/j.tvjl.2023.106045 38000694

[173] OOgunnigbo, O. et al. Exploring the antimicrobial stewardship educational needs of healthcare students and the potential of an antimicrobial prescribing app as an educational tool in selected African countries. *Antibiotics* **11**, 832 (2022). 10.3390/antibiotics11050691 PMC9137764 35625335

[174] Bedekelabou, A. P., Oyetola, D. W., Coulibaly, Z. L., Akinsola, O. & Bada-Alambedji, R. First assessment of the knowledge, attitudes, and practices of health actors in Togo and Ivory Coast in regard to antibiotic resistance. *Int. J. One Health* **8**, 108–123 (2022).

[175] Dyar, O. J., Lund, M., Lindsjo, C., Stalsby Lundborg, C. & Pulcini, C. Preparedness to prescribe antibiotics responsibly: a comparison between final year medical students in France and Sweden. *Eur. J. Clin. Microbiol. Infect. Dis.* **38**, 711–717 (2019). 30771121 10.1007/s10096-019-03494-2 PMC6425071

[176] Serra-Burriel, M., Campillo-Artero, C., Agodi, A., Barchitta, M. & Lopez-Casasnovas, G. Association between antibiotic resistance in intensive care unit (ICU)-acquired infections and excess resource utilization: evidence from Spain, Italy, and Portugal. *Infect. Control Hosp. Epidemiol.* **43**, 1360–1367 (2022). 34657648 10.1017/ice.2021.429

[177] Carrara, E. et al. Clinical management of severe infections caused by carbapenem-resistant gram-negative bacteria: a worldwide cross-sectional survey addressing the use of antibiotic combinations. *Clin. Microbiol. Infect.* **28**, 66–72 (2022). 33975010 10.1016/j.cmi.2021.05.002

[178] Balasubramanian, R., Van Boeckel, T. P., Carmeli, Y., Cosgrove, S. & Laxminarayan, R. Global incidence in hospital-associated infections resistant to antibiotics: an analysis of point prevalence surveys from 99 countries. *PLoS Med.* **20**, e1004178 (2023). 37310933 10.1371/journal.pmed.1004178 PMC10263350

[179] Allel, K. et al. The impact of inpatient bloodstream infections caused by antibiotic-resistant bacteria in low- and middle-income countries: a systematic review and meta-analysis. *PLoS Med.* **20**, e1004199 (2023). 37347726 10.1371/journal.pmed.1004199 PMC10287017

[180] Li, G. et al. Towards understanding global patterns of antimicrobial use and resistance in neonatal sepsis: insights from the NeoAMR network. *Arch. Dis. Child.* **105**, 26–31 (2020). 31446393 10.1136/archdischild-2019-316816 PMC6951234

[181] Monedero-Recuero, I., Gegia, M., Wares, D. F., Chadha, S. S. & Mirzayev, F. Situational analysis of 10 countries with a high burden of drug-resistant tuberculosis 2 years post-UNHLM declaration: progress and setbacks in a changing landscape. *Int. J. Infect. Dis.* **108**, 557–567 (2021). 34139370 10.1016/j.ijid.2021.06.022

[182] Tebano, G. et al. Essential and forgotten antibiotics: an inventory in low- and middle-income countries. *Int. J. Antimicrob. Agents* **54**, 273–282 (2019). 31260741 10.1016/j.ijantimicag.2019.06.017

[183] Sweileh, W. M. Global research publications on irrational use of antimicrobials: call for more research to contain antimicrobial resistance. *Global. Health* **17**, 94 (2021). 34429128 10.1186/s12992-021-00754-9 PMC8383246

[184] Outterson, K., Orubu, E. S. F., Rex, J., Ardal, C. & Zaman, M. H. Patient access in 14 high-income countries to new antibacterials approved by the US Food and Drug Administration, European medicines agency, Japanese pharmaceuticals and medical devices agency, or health Canada, 2010-2020. *Clin. Infect. Dis.* **74**, 1183–1190 (2021). 10.1093/cid/ciab612 PMC8994582 34251436

[185] Stedt, J. et al. Antibiotic resistance patterns in *Escherichia coli* from gulls in nine European countries. *Infect. Ecol. Epidemiol.* **4**, 23203 (2014). 10.3402/iee.v4.21565 PMC3889177 24427451

[186] Yang, D. et al. Antimicrobial resistance genes aph(3’)-III, erm(B), sul2 and tet(W) abundance in animal faeces, meat, production environments and human faeces in Europe. *J. Antimicrob. Chemother.* **77**, 1883–1893 (2022). 35466367 10.1093/jac/dkac133 PMC9244224

[187] Waterlow, N. R., Cooper, B. S., Robotham, J. V. & Knight, G. M. Antimicrobial resistance prevalence in bloodstream infection in 29 European countries by age and sex: an observational study. *PLoS Med.* **21**, e1004301 (2024). 38484006 10.1371/journal.pmed.1004301 PMC10939247

[188] Kariuki, S., Kering, K., Wairimu, C., Onsare, R. & Mbae, C. Antimicrobial resistance rates and surveillance in sub-Saharan Africa: where are we now? *Infect. Drug Resist.* **15**, 3589–3609 (2022). 35837538 10.2147/IDR.S342753 PMC9273632

[189] Veloo, A. C. M. et al. Antimicrobial susceptibility profiles of anaerobic bacteria, isolated from human clinical specimens, within different European and surrounding countries. A joint ESGAI study. *Anaerobe* **61**, 102111 (2020). 31634565 10.1016/j.anaerobe.2019.102111

[190] Holm, M. et al. Capturing data on antimicrobial resistance patterns and trends in use in regions of Asia (CAPTURA). *Clin. Infect. Dis.* **77**, S500–S506 (2023). 38118015 10.1093/cid/ciad567 PMC10732560

[191] Dossouvi, K. M. & Ametepe, A. S. Carbapenem resistance in animal-environment-food from africa: a systematic review, recommendations and perspectives. *Infect. Drug Resist.* **17**, 1699–1728 (2024). 38715963 10.2147/IDR.S458317 PMC11075763

[192] Wang, M. et al. Clinical outcomes and bacterial characteristics of carbapenem-resistant Klebsiella pneumoniae complex among patients from different global regions (CRACKLE-2): a prospective, multicentre, cohort study. *Lancet Infect. Dis.* **22**, 401–412 (2022). 34767753 10.1016/S1473-3099(21)00399-6 PMC8882129

[193] Mizuno, S. et al. Comparison of national strategies to reduce meticillin-resistant Staphylococcus aureus infections in Japan and England. *J. Hosp. Infect.* **100**, 280–298 (2018). 30369423 10.1016/j.jhin.2018.06.026

[194] Rahbe, E., Watier, L., Guillemot, D., Glaser, P. & Opatowski, L. Determinants of worldwide antibiotic resistance dynamics across drug-bacterium pairs: a multivariable spatial-temporal analysis using ATLAS. *Lancet Planet. Health* **7**, e547–e557 (2023). 37437996 10.1016/S2542-5196(23)00127-4

[195] Sebastian, S. et al. Different microbial and resistance patterns in primary total knee arthroplasty infections - a report on 283 patients from Lithuania and Sweden. *BMC Musculoskelet. Disord.* **22**, 800 (2021). 34535109 10.1186/s12891-021-04689-5 PMC8449428

[196] Kenyon, C. R., De Baetselier, I. & Crucitti, T. Does gonorrhoea screening intensity play a role in the early selection of antimicrobial resistance in men who have sex with men (MSM)? a comparative study of Belgium and the United Kingdom. *F1000Research* **7**, 569 (2018). 30364212 10.12688/f1000research.14869.1 PMC6192441

[197] Poudyal, N. et al. Effective stakeholder engagement for collation, analysis and expansion of antimicrobial resistance (AMR) data: a CAPTURA experience. *Clin. Infect. Dis.* **77**, S519–S527 (2023). 38118005 10.1093/cid/ciad585 PMC10732561

[198] GRAM Typhoid Collaborators. Estimating the subnational prevalence of antimicrobial resistant Salmonella enterica serovars Typhi and Paratyphi A infections in 75 endemic countries, 1990–2019: a modelling study. *Lancet. Glob. Health* **12**, e406–e418 (2024). 10.1016/S2214-109X(23)00585-5 PMC10882211 38365414

[199] De Jong, A. et al. European-wide antimicrobial resistance monitoring in commensal Escherichia coli isolated from healthy food animals between 2004 and 2018. *J. Antimicrob. Chemother.* **77**, 3301–3311 (2022). 36203261 10.1093/jac/dkac318

[200] Handley, B. L. et al. Evaluating the yaws diagnostic gap: a survey to determine the capacity of and barriers to improving diagnostics in all yaws-endemic countries. *medRxiv*10.1101/2022.05.30.22275669 (2022).

[201] Guzman-Blanco, M., Labarca, J. A., Villegas, M. V. & Gotuzzo, E. Extended spectrum beta-lactamase producers among nosocomial Enterobacteriaceae in Latin America. *Braz. J. Infect. Dis.* **18**, 421–433 (2014). 24389277 10.1016/j.bjid.2013.10.005 PMC9427466

[202] Freeman, J. et al. Five-year Pan-European, longitudinal surveillance of Clostridium difficile ribotype prevalence and antimicrobial resistance: the extended ClosER study. *Eur. J. Clin. Microbiol. Infect. Dis.* **39**, 169–177 (2020). 31811507 10.1007/s10096-019-03708-7 PMC6962284

[203] Kakooza, F. et al. Genomic surveillance and antimicrobial resistance determinants in Neisseria gonorrhoeae isolates from Uganda, Malawi and South Africa, 2015-20. *J. Antimicrob. Chemother.* **78**, 1982–1991 (2023). 37352017 10.1093/jac/dkad193

[204] Tornimbene, B. et al. Global antimicrobial resistance and use surveillance system on the African continent: early implementation 2017–2019. *Afr. J. Lab. Med.* **11**, 1594 (2022). 36091353 10.4102/ajlm.v11i1.1594 PMC9453120

[205] GBD 2021 Antimicrobial Resistance Collaborators. Global burden of bacterial antimicrobial resistance 1990–2021: a systematic analysis with forecasts to 2050. *Lancet* **404**, 1199–1226 (2024). 10.1016/S0140-6736(24)01867-1 PMC11718157 39299261

[206] Carey, M. E. et al. Global diversity and antimicrobial resistance of typhoid fever pathogens: insights from a meta-analysis of 13,000 *Salmonella* Typhi genomes. *eLife* **12**, e85861 (2023). 10.7554/eLife.85867 PMC10506625 37697804

[207] Hou, J. et al. Global trend of antimicrobial resistance in common bacterial pathogens in response to antibiotic consumption. *J. Hazard. Mater.* **442**, 130042 (2023). 36182890 10.1016/j.jhazmat.2022.130042

[208] Karlowsky, J. A. et al. In vitro activity of ceftazidime-avibactam against clinical isolates of Enterobacteriaceae and Pseudomonas aeruginosa collected in Asia-Pacific countries: results from the INFORM global surveillance progr. *Antimicrob. Agents Chemother.* **62**, e02569 (2018). 29760124 10.1128/AAC.02569-17 PMC6021687

[209] Karlowsky, J. A. et al. In vitro activity of ceftazidime-avibactam against clinical isolates of Enterobacteriaceae and Pseudomonas aeruginosa collected in Latin American countries: results from the INFORM global surveillance pro. *Antimicrob. Agents Chemother.* **63**, e01814 (2019). 30670424 10.1128/AAC.01814-18 PMC6437529

[210] Karlowsky, J. A. et al. In vitro activity of imipenem/relebactam against non-*Morganellaceae**Enterobacterales* and *Pseudomonas aeruginosa* in Latin America: SMART 2018–2020. *Braz. J. Infect. Dis.* **27**, 102766 (2023). 10.1016/j.bjid.2023.102775 PMC10192529 37169345

[211] Karlowsky, J. A. et al. In vitro activity of imipenem/relebactam against piperacillin/tazobactam-resistant and meropenem-resistant non-Morganellaceae *Enterobacterales* and *Pseudomonas aeruginosa* collected from patients with lower respiratory tract infections: SMART 2018–2020. *JAC-Antimicrob. Resist.* **5**, dlad059 (2023). 10.1093/jacamr/dlad003 PMC9856267 36694850

[212] Rutz, J. et al. Individual and institutional predisposing factors of MRSA surgical site infection and outcomes—a retrospective case-control-study in 14 European high-volume surgical centres. *JAC-Antimicrob. Resist.* **6**, dlad142 (2024). 10.1093/jacamr/dlae046 PMC10993902 38577701

[213] Kinross, P. et al. Livestock-associated meticillin-resistant Staphylococcus aureus (MRSA) among human MRSA isolates, European Union/European Economic Area countries, 2013. *Eurosurveillance* **22**, 00696 (2017). 10.2807/1560-7917.ES.2017.22.44.16-00696 PMC5710135 29113628

[214] Babu Rajendran, N. et al. Mandatory surveillance and outbreaks reporting of the WHO priority pathogens for research & discovery of new antibiotics in European countries. *Clin. Microbiol. Infect.* **26**, 943.e1–943.e6 (2020). 31812771 10.1016/j.cmi.2019.11.020

[215] Baede, V. O. et al. Markers of epidemiological success of methicillin-resistant Staphylococcus aureus isolates in European populations. *Clin. Microbiol. Infect.* **29**, 1166–1173 (2023). 37207981 10.1016/j.cmi.2023.05.015 PMC10775016

[216] Beeton, M. L. et al. Mycoplasma pneumoniae infections, 11 countries in Europe and Israel, 2011 to 2016. *Eurosurveillance* **25**, 1900112 (2020). 10.2807/1560-7917.ES.2020.25.2.1900112 PMC6976882 31964459

[217] Dyson, Z. A. et al. Pathogen diversity and antimicrobial resistance transmission of Salmonella enterica serovars Typhi and Paratyphi A in Bangladesh, Nepal, and Malawi: a genomic epidemiological study. *Lancet Microbe* **5**, 100841 (2024). 38996496 10.1016/S2666-5247(24)00047-8 PMC11300424

[218] Barkume, C. et al. Phase I of the surveillance for enteric fever in Asia Project (SEAP): an overview and lessons learned. *J. Infect. Dis.* **218**, S188–S194 (2018). 30304505 10.1093/infdis/jiy522 PMC6226726

[219] Tosas Auguet, O. et al. Population-level faecal metagenomic profiling as a tool to predict antimicrobial resistance in *Enterobacterales* isolates causing invasive infections: an exploratory study across Cambodia, Kenya, and the UK. *EClinicalMedicine* **36**, 100914 (2021). 10.1016/j.eclinm.2021.100910 PMC8173267 34124634

[220] Stefaniak, K., Kiedrzynski, M., Korzeniewska, E., Kiedrzynska, E. & Harnisz, M. Preliminary insights on carbapenem resistance in Enterobacteriaceae in high-income and low-/middle-income countries. *Sci. Total Environ.* **957**, 177593 (2024). 39551200 10.1016/j.scitotenv.2024.177593

[221] Savoldi, A., Carrara, E., Graham, D. Y., Conti, M. & Tacconelli, E. Prevalence of antibiotic resistance in Helicobacter pylori: a systematic review and meta-analysis in World Health Organization regions. *Gastroenterology* **155**, 1372–e17 (2018). 29990487 10.1053/j.gastro.2018.07.007 PMC6905086

[222] Argimon, S. et al. Rapid genomic characterization and global surveillance of Klebsiella using pathogenwatch. *Clin. Infect. Dis.* **73**, S325–S335 (2021). 34850838 10.1093/cid/ciab784 PMC8634497

[223] Criscuolo, N. G., Pires, J., Zhao, C. & Van Boeckel, T. P. resistancebank.org, an open-access repository for surveys of antimicrobial resistance in animals. *Sci. Data* **8**, 189 (2021). 34294731 10.1038/s41597-021-00978-9 PMC8298417

[224] Torumkuney, D. et al. Results from the survey of antibiotic resistance (SOAR) 2014-16 in Bulgaria, Romania, Serbia and Croatia. *J. Antimicrob. Chemother.* **73**, v2–v13 (2018). 29659882 10.1093/jac/dky066

[225] Malik, H. et al. Review of antibiotic use and resistance in food animal production in WHO South-East Asia Region. *J. Infect. Public Health* **16**, 172–182 (2023). 37977981 10.1016/j.jiph.2023.11.002

[226] Yang, D. et al. Risk factors for the abundance of antimicrobial resistance genes aph(3’)-III, erm(B), sul2 and tet(W) in pig and broiler faeces in nine European countries. *J. Antimicrob. Chemother.* **77**, 969–978 (2022). 35061866 10.1093/jac/dkac002 PMC8969523

[227] Jarlier, V. et al. Strong correlation between the rates of intrinsically antibiotic-resistant species and the rates of acquired resistance in Gram-negative species causing bacteraemia, EU/EEA, 2016. *Eurosurveillance* **24**, 1800538 (2019). 31431208 10.2807/1560-7917.ES.2019.24.33.1800538 PMC6702794

[228] Huijbers, P. M. C., Larsson, D. G. J. & Flach, C.-F. Surveillance of antibiotic resistant *Escherichia coli* in human populations through urban wastewater in ten European countries. *Environ. Pollut.* **261**, 114200 (2020). 10.1016/j.envpol.2020.114200 32220750

[229] Piovani, D., Figlioli, G., Nikolopoulos, G. K. & Bonovas, S. The global burden of enteric fever, 2017–2021: a systematic analysis from the global burden of disease study 2021. *EClinicalMedicine* **77**, 102883 (2024). 39469533 10.1016/j.eclinm.2024.102883 PMC11513656

[230] Odoj, K. *et al*. Tracking candidemia trends and antifungal resistance patterns across Europe: an in-depth analysis of surveillance systems and surveillance studies. *J. Fungi* **10**, 685 (2024). 10.3390/jof10100685 PMC11514733 39452637

[231] Al-Saleh, A., Shahid, M., Farid, E. & Bindayna, K. Trends in methicillin-resistant Staphylococcus aureus in the Gulf Cooperation Council countries: antibiotic resistance, virulence factors and emerging strains. *East. Mediterr. Health J.* **28**, 434–443 (2022). 35815875 10.26719/emhj.22.042

[232] Rossolini, G. M., Bochenska, M., Fumagalli, L. & Dowzicky, M. Trends of major antimicrobial resistance phenotypes in enterobacterales and gram-negative non-fermenters from ATLAS and EARS-net surveillance systems: Italian vs. European and global data, 2008-2018. *Diagn. Microbiol. Infect. Dis.* **101**, 115512 (2021). 34419741 10.1016/j.diagmicrobio.2021.115512

[233] Eyre, D. W. et al. WGS to predict antibiotic MICs for Neisseria gonorrhoeae. *J. Antimicrob. Chemother.* **72**, 1937–1947 (2017). 28333355 10.1093/jac/dkx067 PMC5890716

[234] Unemo, M. et al. WHO global antimicrobial resistance surveillance for Neisseria gonorrhoeae 2017-18: a retrospective observational study. *Lancet Microbe* **2**, e627–e636 (2021). 35544082 10.1016/S2666-5247(21)00171-3

[235] Rahbe E., Watier L., Guillemot D., Glaser P., & Opatowski L. Worldwide antibiotic resistance dynamics: how different is it from one drug-bug pair to another? *medRxiv*10.1101/2022.02.09.22270726 (2022).

[236] Maugeri, A., Barchitta, M., Magnano San Lio, R. & Agodi, A. Socioeconomic and governance factors disentangle the relationship between temperature and antimicrobial resistance: a 10-year ecological analysis of European countries. *Antibiotics* **12**, 861 (2023). 10.3390/antibiotics12040777 PMC10135271 37107139

[237] Petrovski, K. R. et al. Susceptibility to antimicrobials of mastitis-causing Staphylococcus aureus, Streptococcus uberis and Str. dysgalactiae from New Zealand and the USA as assessed by the disk diffusion test. *Aust. Vet. J.* **93**, 227–233 (2015). 26113347 10.1111/avj.12340

[238] George, C. R. R. et al. Systematic review and survey of Neisseria gonorrhoeae ceftriaxone and azithromycin susceptibility data in the Asia Pacific, 2011 to 2016. *PloS ONE* **14**, e0213312 (2019). 30943199 10.1371/journal.pone.0213312 PMC6447224

[239] Himanshu et al. Systematic surveillance and meta-analysis of antimicrobial resistance and food sources from China and the USA. *Antibiotics* **11**, 1471 (2022). 36358126 10.3390/antibiotics11111471 PMC9686904

[240] Arieti, F. et al. The antimicrobial resistance travel tool, an interactive evidence-based educational tool to limit antimicrobial resistance spread. *J. Travel Med.* **29**, taac045 (2022). 10.1093/jtm/taac045 PMC9282094 35348740

[241] Anonymous The burden of bacterial antimicrobial resistance in the WHO African region in 2019: a cross-country systematic analysis. *Lancet Glob. health* **12**, e201–e216 (2024). 38134946 10.1016/S2214-109X(23)00539-9 PMC10805005

[242] Aguilar, G. R. et al. The burden of antimicrobial resistance in the Americas in 2019: a cross-country systematic analysis. *Lancet Reg. Health Am.* **25**, 100561 (2023). 10.1016/j.lana.2023.100561 PMC10505822 37727594

[243] Cole, M. J. et al. The European gonococcal antimicrobial surveillance programme (Euro-GASP) appropriately reflects the antimicrobial resistance situation for Neisseria gonorrhoeae in the European Union/European Economic Area. *BMC Infect. Dis.* **19**, 1040 (2019). 31822275 10.1186/s12879-019-4631-x PMC6902330

[244] World Health Organization. *Antimicrobial resistance: global report on surveillance* (WHO, 2014).

[245] Food and Agriculture Organization of the United Nations. *Annex to the GLG Report: Towards specific commitments and action in response to antimicrobial resistance* (FAO, 2024).

[246] Anderson, M. et al. *The Socioeconomic Drivers and Impacts of Antimicrobial Resistance**(**AMR**):**implications for Policy and Research*. https://eurohealthobservatory.who.int/publications/i/the-socioeconomic-drivers-and-impacts-of-antimicrobial-resistance-(amr)-implications-for-policy-and-research (2024). 39656899

[247] Anderson, M., Kluge, H. H. P., Lo Fo Wong, D., Butler, R. & Mossialos, E. Promoting sustainable national action to tackle antimicrobial resistance: a proposal to develop an antimicrobial resistance accountability index. *Lancet Microbe* **5**, 100997 (2024). 39341218 10.1016/j.lanmic.2024.100997

[248] Anderson M., Mossialos E., Shafaque U., Ranganathan S. A systematic review and critical appraisal of indicators to comparatively measure national performance in addressing antimicrobial resistance (AMR). PROSPERO 2024 CRD42024625477. https://www.crd.york.ac.uk/PROSPERO/view/CRD42024625477 (2024).

