# Supplementary material for: Mind the governance gap: a one health scoping review of national AMR performance indicators
Source: NPJ Antimicrob Resist. 2026 May 13;4:40. doi: 10.1038/s44259-026-00213-8 (PMC13190849; doi:10.1038/s44259-026-00213-8)
Supplement: Supplementary file 1 — Supplementary Information [file 44259_2026_213_MOESM1_ESM.pdf]

Supplemental Materials for:

Mind the Governance Gap: A One Health Scoping Review of National AMR Performance Indicators

Table of Contents

Supplemental Tables 4
Supplemental Table 1. Grey literature source links 4
Supplemental Table 2. Summary of academic studies 9
Supplemental Table 3. Summary of grey literature 44

Supplemental Figures 52
Supplemental Figure 1. Distribution of indicators by sector for academic and grey literature 52
Supplemental Figure 2. Distribution of academic study publications by year 53

Preferred Reporting Items for Systematic reviews and Meta-Analyses extension for Scoping Reviews (PRISMA-ScR) Checklist 54

References 57

Supplemental Data 1 & 2: Summary of Academic and Grey Literature Indicators 85

Supplemental Tables

Supplemental Table 1. Grey literature source links.

	Study	*Institution*	*Year of publication*	*Link*
1	8th Annual Report on Antimicrobial Agents Intended for Use in Animals (ANIMUSE)	WOAH	2024	https://www.woah.org/app/uploads/2024/05/woah-amu-report-2024-final.pdf
2	Addressing gender inequalities in national action plans on AMR	WHO	2024	https://www.who.int/publications/i/item/9789240097278
3	Antimicrobial consumption and resistance in bacteria from humans and food-producing animals	ECDC; EMA; EFSA	2024	https://www.efsa.europa.eu/en/efsajournal/pub/8589
4	Antimicrobial resistance in the EU/EAA (EARS-Net): Annual Epidemiological Report for 2023	ECDC	2024	https://www.ecdc.europa.eu/en/publications-data/antimicrobial-resistance-eueea-ears-net-annual-epidemiological-report-2023
5	Antimicrobial resistance: global report on surveillance	WHO	2014	https://www.who.int/publications/i/item/9789241564748
6	Antimicrobial stewardship programmes in health-care facilities in LMICs: a WHO practical toolkit	WHO	2019	https://www.who.int/publications/i/item/9789241515481
7	Assessment tool of the minimum requirements for infection prevention and control programmes at the national level	WHO	2021	https://www.who.int/publications/m/item/assessment-tool-of-the-minimum-requirements-for-infection-prevention-and-control-programmes-at-the-national-level
8	Averting the AMR Crisis: What are the Avenues for policy action for countries in Europe?	Adapted from Cassini et al. 2019	2019	https://www.ncbi.nlm.nih.gov/books/NBK543406/
9	Building the investment case for action against antimicrobial resistance (Annex to the GLG report)	Global Leaders Group on AMR	2024	https://www.amrleaders.org/docs/librariesprovider20/glg/annex-to-the-glg-report.pdf
10	CAESAR External Quality	WHO	2022	https://www.who.int/europe/publications/i/item/9789289058391

	Assessment			
11	Commitment to Development index (Health)	Centre for Global Development	2023	https://www.cgdev.org/
12	EU Council recommendation on stepping up EU actions to combat AMR in a One Health approach	EU Council	2023	https://health.ec.europa.eu/publications/council-recommendation-stepping-eu-actions-combat-antimicrobial-resistance-one-health-approach_en
13	Guideline on the reporting of antimicrobial sales and use in animals at the EU level – denominators and indicators	European Commission		
14	Eurostat	Eurostat	N/A	https://ec.europa.eu/eurostat
15	Evaluation of the Performance of Veterinary Services: PVS Tool (Terrestrial 2019)	WOAH	2019	https://www.woah.org/app/uploads/2021/03/af-book-ang-pvstool-trr.pdf
16	FAO-ATLASS "SET AMR" (Surveillance Evaluation Tool for AMR)	FAO	First piloted in 2016	https://www.fao.org/antimicrobial-resistance/resources/tools/fao-atlass/en/
17	From reacting to preventing pandemics: building animal health and wildlife systems for One Health in East Asia and Pacific	FAO; World Bank	2022	https://openknowledge.fao.org/items/b5333fcb-5bf8-4ffc-8ee1-ff574850b655
18	G7 Compliance Report on Antimicrobial Resistance, 2021-2023	ARMoR; G7	2024	https://www.g7.utoronto.ca/compliance/AMR-compliance-2021-2023.pdf
19	GLASS-AMR	WHO	Published in 5-year cycles	https://www.who.int/initiatives/glass
20	Global Coalition on Aging AMR Preparedness Index Progress Report	Global Coalition on Ageing	2024	https://globalcoalitiononaging.com/wp-content/uploads/2024/01/2024-AMR-Index-Progress-Report.pdf
21	Global Indicator Framework for SDGs	UNSD	Adopted in 2017	https://unstats.un.org/sdgs/indicators/indicators-list/
22	How can the EU support sustainable innovation and access to effective antibiotics?	European Health Observatory on Health Systems and Policies	2023	https://eurohealthobservatory.who.int/publications/i/how-can-the-eu-support-sustainable-innovation-and-access-to-effective-antibiotics-policy-options-for-existing-and-new-medicines
23	Implementation of WOAH	WOAH	2022	https://www.woah.org/app/uploads/2022/12/annual-report-observatory-2022.pdf

	standards: the observatory annual report 2022			
24	InFARM surveillance components and implementation questionnaire	FAO		https://infarm.fao.org/api/download/annex-3_infarm-animals_surveillance-questionnare.docx
25	International Health Regulations States Parties Self-assessment annual reporting tool	WHO	Published annually	https://www.who.int/emergencies/operations/international-health-regulations-monitoring-evaluation-framework/states-parties-self-assessment-annual-reporting
26	IPCAT 2	WHO	2017	https://www.who.int/publications/i/item/WHO-HIS-SDS-2017.13
27	Joint Monitoring Programme for Water Supply, Sanitation and Hygiene (JMP)	WHO; UNICEF	Published periodically	https://www.who.int/teams/environment-climate-change-and-health/water-sanitation-and-health/monitoring-and-evidence/wash-monitoring
28	Methodology to analyze AMR-relevant legislation in the food and agriculture sector	WHO	2020	https://openknowledge.fao.org/server/api/core/bitstreams/270bb661-6608-4e67-982a-de331754e930/content
29	Minimizing risk of developing antibiotic resistance and aquatic ecotoxicity in the environment resulting from the manufacturing of human antibiotics	AMRIA	2022; updated 2025	https://www.amrindustryalliance.org/antibiotic-manufacturing-standard/
30	Monitoring and evaluation for effective management of zoonotic diseases	WHO	2024	https://www.woah.org/app/uploads/2024/12/monitoring-and-evaluation-for-effective-disease-management--web.pdf
31	Monitoring and evaluation of the global action plan on antimicrobial resistance: framework and recommended indicators	WHO; WOAH; FAO	2019	https://www.woah.org/app/uploads/2021/03/en-mande-gap-amr.pdf
32	Monitoring Framework for the WHO Strategic and Operational priorities to address drug-resistant bacterial infections	WHO	2024	https://www.who.int/news-room/articles-detail/consultation-monitoring-framework-strategic-and-operational-priorities-to-address-drug-resistant-bacterial-infections
33	Muscat Ministerial Manifesto on AMR	UN Multi-partner Trust Fund	2022	http://amrconference2022.om/assets/images/Third_High-Level_Report.pdf
34	OECD Embracing a One Health Framework to Fight Antimicrobial Resistance	OECD	2023	https://www.oecd.org/en/publications/embracing-a-one-health-framework-to-fight-antimicrobial-resistance_ce44c755-en.html

35	One Health Trust Resistance Map	IQVIA	N/A	https://resistancemap.onehealthtrust.org/
36	Operational approach to antimicrobial stewardship in the WHO Eastern Mediterranean Region	WHO	2024	https://applications.emro.who.int/docs/WHOEMCSR801E-eng.pdf?ua=1
37	People-centred approach to addressing antimicrobial resistance in human health: WHO core package of interventions to support national action plans	WHO	2023	https://www.who.int/publications/i/item/9789240082496
38	Point prevalence survey of healthcare-associated infections and antimicrobial use in European long-term care facilities	ECDC	2025	https://www.ecdc.europa.eu/en/publications-data/point-prevalence-survey-healthcare-associated-infections-and-antimicrobial-use-6
39	Priorities of the Global Leaders Group on AMR	Global Leaders Group on AMR	2025	https://www.amrleaders.org/resources/m/item/priorities-of-the-global-leaders-group-on-amr-for-2025---2027
40	Surveillance of health care-associated infections at national and facility levels: practical handbook (WHO)	WHO	2024	https://www.who.int/publications/i/item/9789240101456
41	The costs and risks of AMR water pollution	WEF	2021	https://www.weforum.org/publications/the-costs-and-risks-of-amr-water-pollution/
42	Third joint inter-agency report on integrated analysis of consumption of antimicrobial agents and occurrence of antimicrobial resistance in bacteria from humans and food-producing animals in the EU/EEA (with disaggregation by antimicrobial class)	EFSA	2021	https://www.efsa.europa.eu/en/efsajournal/pub/6712
43	TrACSS 2023	WHO	2023	https://www.who.int/publications/m/item/tracking-amr-country-self-assessment-survey-tracss-(7.0)-2023
44	UHC Watch	WHO	2024	https://apps.who.int/dhis2/uhcwatch/#/
45	WHO benchmarks for strengthening health emergency capacities	WHO	2019	https://iris.who.int/server/api/core/bitstreams/8883ae92-ecbe-4cc6-8ad4-500297ef1df9/content

46	WHO implementation handbook for NAPs on AMR: guidance for the human health sector (annex 7)	WHO	2022	https://www.who.int/publications/i/item/9789240041981
47	WHO Joint External Evaluation - AMR	WHO	N/A	https://www.who.int/emergencies/operations/international-health-regulations-monitoring-evaluation-framework/joint-external-evaluations
48	Worldwide country situation analysis: response to antimicrobial resistance	WHO	2015	https://www.paho.org/sites/default/files/2019-09/9789241564946_eng.pdf

Supplemental Table 2. Summary of academic studies.

Study	*Year*	*OH Sector*	*Data Source*	*Unit of Observation*	*Setting*	*Time Frame*	*Key Findings*

A community survey of antibiotic consumption among children in Madagascar and Senegal: the importance of healthcare access and care quality[8]	2016	Human	Cross-sectional population-based survey	2 countries (Madagascar & Senegal)	WHO AFR (LIC)	2014-2015	Elevated levels of antibiotic consumption often for symptoms linked to viral infection; density of healthcare structures, national antibiotic payment programmes and prescriber training all important drivers of antibiotic consumption
A multinational survey of companion animal veterinary clinicians: How can antimicrobial stewardship guidelines be optimised for the target stakeholder?[9]	2024	Animal	21-question survey distributed online	43 countries	WHO EUR + Morocco and Tunisia	2022	Correlation between agreement with recommended AM stewardship practice and awareness of ASGs; awareness of companion animal (pets) ASGs was greatest among countries that have national guidelines
A multivariable analysis of the contribution of socioeconomic and environmental factors to blood culture Escherichia Coli resistant to fluoroquinolones in high- and middle-income countries[10]	2022	Multisectoral	CDDEP, GLASS, World Bank	71 countries	Global	2022	Corruption and antibiotic use strong predictors in HIC, MIC; sanitation services also play a role in MIC; improvement of AMR surveillance in LIC critical
A One-Health Quantitative Model to Assess the Risk of Antibiotic Resistance Acquisition in Asian Populations: Impact of Exposure Through Food, Water, Livestock and Humans[11]	2020	Human, Animal, Environmental and Multisectoral	Author-produced model	3 countries	WHO SEAR/WPR	2021	After careful parameterization for a specific country setting, it will also allow a quantitative assessment of the impact of potential control measures and provide better support for their prioritization; This illustrates the need for collecting high-quality data on antimicrobial use, hygiene practices and ARB prevalence, as model predictions are bound to be dependent on the quality of the available data
A qualitative approach for a situation analysis of AMR risks in the food animal production sector[12]	2023	Human, Animal and Multisectoral	Codex Alimentarius; WOAH; Survey	33 countries	FAO RLC members	2017	The tool allows the identification and collection of information from the different sectors of animal production; The analysis highlights, lists and prioritizes the challenges to be addressed for the management of AMR at the technical level, but also at the

							level of authorities and decision makers, based on country priorities and resources
A review of national action plans on antimicrobial resistance: strengths and weaknesses[13]	2022	Human, Animal and Multisectoral	WHO library of NAPs	70 countries	Global	2021	The lack of acknowledgement and definition of One Health in the WHO Gap may inhibit other countries in adopting a One Health approach; It is vital to motivate and support the 12 non-WHO approved countries to get their NAP approved, as well as the 120 countries that have not submitted a NAP to date is required.
An analysis of existing national action plans for antimicrobial resistance-gaps and opportunities in strategies optimising antibiotic use in human populations[14]	2023	Human and Multisectoral	WHO library of NAPs; Global Database for Antimicrobial Resistance Country Self-Assessment	108 countries	Global	2008-2022	Foster in-country development of NAP policy through political commitment, identify deliverable NAP objectives, understand and invest in the human resource capacity needed, and develop and measure indicators that are specific to AMR in human population.
An analysis of national action plans on antimicrobial resistance in Southeast Asia using a governance framework approach[15]	2021	Multisectoral	WHO library of NAPs	10 countries	ASEAN	2021	Participation during NAP development emphasised, but fewer NAPs discussed sustained engagement (most forms of public engagement were top-down); Gender considerations not mentioned at all; Behavioural change campaigns needed; Plans regarding the environmental sector inadequate
An elephant in the room? Explaining agenda-setting in antimicrobial resistance policies in 30 European countries[16]	2024	Multisectoral	Political party websites; Manifesto Project Database	30 countries	EU/EFTA/UK	2015-2020	Complex, intersectoral policy issues are often not considered as such by political parties
An insight into the implementation of the global action plan on antimicrobial resistance in the WHO African region: A roadmap for action[17]	2021	Multisectoral	WHO, FAO and OIE global tripartite database	25 countries	WHO AFR	NA	Overall GAP implementation performance in WHO AFR is inadequate, no significant difference in GAP implementation performance between African sub-regions and between the income groups, roadmap for

							action is recommended to realign country-specific AMR plans to the Global AMR agenda
Anthropological and socioeconomic factors contributing to global antimicrobial resistance: a univariate and multivariable analysis[18]	2018	Human	ResistanceMap, WHO 2014 AMR report; IQVIA MIDAS (consumption); World Bank DataBank (GDP, healthcare spending, infrastructure); Transparency International (corruption index)	103 countries	Global	2008-2013, 2008-2014 for 69 countries	Poorer infrastructure and governance associated with higher levels of AM resistance; Intervention in antibiotic consumption insufficient, as contagion is likely the main factor affecting AM resistance levels, especially in LMIC.
Antibiotic Consumption in Primary Care in Costa Rica and Italy: A Retrospective Cross-Country Analysis[19]	2023	Human	Tessera Sanitaria	1 country (Italy)	WHO EUR	2021-2022	The trend of antibiotic consumption in 2022 in both countries exhibited a steady rise, resulting in increased pharmaceutical expenditure and potentially contributing to the antibiotic resistance phenomenon.
Antibiotic exposure among children younger than 5 years in low-income and middle-income countries: a cross-sectional study of nationally representative facility-based and household-based surveys[20]	2019	Human	DHS; SPA surveys	8 countries	Global (LMIC)	2006-2016	Antibiotic exposure of children in LMICs is extremely high; antibiotics prescriptions issued inappropriately; up-to-date clinical guidelines, appropriate point-of-care diagnostic tests, e-support all necessary
Antibiotic Exposure and Other Risk Factors for Antimicrobial Resistance in Nasal Commensal Staphylococcus aureus: An Ecological Study in 8 European Countries[21]	2015	Human	APRES	8 countries	EU (HIC)	2010-2011	In a population with no recent antibiotic use, the prescription behaviour of the general practitioner affects the odds for carriage of a resistant S. aureus, highlighting the need for cautious prescribing in primary care
Antibiotic resistance patterns in Escherichia coli from gulls in nine European countries[22]	2013	Animal	Samples collected on-site	9 countries	EU/UK	2009	Gulls may serve as a sentinel of environmental levels of antibiotic-resistant E. coli to complement studies of human-associated microbiota
Antibiotic treatment patterns across Europe in patients with complicated skin and soft-tissue infections due to	2014	Human	Patients for data collection identified by study investigators (ID specialists, internists with ID subspecialty, medical microbiologists)	12 countries	EU	2010-2011	Correlation between availability of local antibiotic stewardship activity (ES, ED protocols) and increased likelihood of reduced hospital LOS; significant

meticillin-resistant Staphylococcus aureus: a plea for implementation of early switch and early discharge criteria[23]							opportunities for certain patients to be switched from i.v. to oral antibiotic therapy and discharged from the hospital sooner were identified
Antibiotic use in Australian and Swedish primary care: a cross-country comparison[24]	2021	Human	National Swedish Drug Registry; PBS, ASM, Date of Supply Reports (AU)	2 countries (Australia & Sweden)	Global (HIC)	2006-2018	The observed differences could be explained by antibiotic choice recommended in guidelines, models of primary care funding, and the presence and duration of national antimicrobial stewardship programmes
Antimicrobial Medicines Consumption in Eastern Europe and Central Asia - An Updated Cross-National Study and Assessment of Quantitative Metrics for Policy Action[25]	2019	Human	Import, sales, wholesaler, certification records; IQVIA; VIORTIS	15 countries & 1 territory	WHO EUR (AMC Network)	2015	The impact of locally produced antibiotics on treatment choices, pharmaceutical industry promotion, perverse incentives to prescribe and dispense antibiotics, availability and use of up-to-date guidelines all need to be considered in developing interventions to improve antibiotic use
Antimicrobial Resistance and Environmental Health: A Water Stewardship Framework for Global and National Action[26]	2022	Environmental	WHO library of NAPs; Literature review	25 countries	Global	2019-2020	The human and animal sectors receive the most attention while the environment sector, food safety and security, and antimicrobial plant use have limited focus in current guidance and policy
Antimicrobial resistance genes aph(3')-III, erm(B), sul2 and tet(W) abundance in animal faeces, meat, production environments and human faeces in Europe[27]	2022	Human	Sample collection as part of EFFORT	9 countries	EU	2014-2017	High variation of ARG abundance assessed using qPCR was found across animal species, environmental samples and humans. A ‘farm to fork’ decreasing trend in ARG abundance was found for both pigs and broilers. The between-country and between-farm variation could be partially attributed to AMU and farm biosecurity levels. Occupational livestock AMR exposure is related to the ARG abundance in human faeces
Antimicrobial Resistance	2022	Multisectoral	Interviews;	5 countries	Global (HMIC)	2018-2020	Free markets play an ambivalent

Policy Protagonists and Processes-A Qualitative Study of Policy Advocacy and Implementation[28]			AMR-IMPACT/Intervene				role in AMR policymaking while social norms are important to enable AMR policy implementation
Antimicrobial resistance preparedness in sub-Saharan African countries[29]	2020	Multisectoral	Joint External Evaluations	44 countries	WHO AFR (Sub-Saharan Africa)	2018-2020	SSA countries ought to approach building robust One Health AMR response networks through multisectoral standardised coordination
Antimicrobial resistance prevalence in bloodstream infection in 29 European countries by age and sex: An observational study[30]	2024	Human	EARS-Net	29 countries	EEA/UK	2019	The prevalence of resistance in BSIs in Europe varies substantially by bacteria and antibiotic over the age and sex of the patient shedding new light on gaps in our understanding of AMR epidemiology
Antimicrobial resistance prevalence in commensal Escherichia coli from broilers, fattening turkeys, fattening pigs and veal calves in European countries and association with antimicrobial usage at country level[31]	2020	Animal	Faecal samples	9 countries	EU	2014-2016	Resistance proportions varied between antimicrobials and animal species, and correlation with usage was not always significant
Antimicrobial Resistance Rates and Surveillance in Sub-Saharan Africa: Where Are We Now?[32]	2022	Human	PubMed	Sub-Saharan regions (NESW)	WHO AFR (Sub-Saharan Africa)	2019	The few available data indicate an increase in reports of AMR rates and resistance genes, which could be resulting in treatment failure and increasing mortalities; Therefore, there is a need for SSA countries to ensure continuous data generation, which will be key in informing strategies to be implemented at the country level as different countries have varying resistance levels
Antimicrobial resistance rates in gram-positive bacteria do not drive glycopeptides use[33]	2017	Human	EARS-Net; ESAC	31 countries	EEA	1997-2015	Increasing trends in glycopeptides use despite decreases in rates of BSI due to MRSA, MRCoNS, VSE are worrisome; increasing local vancocymin use should be carefully evaluated when planning and implementing AM

							stewardship systems
Antimicrobial resistance: the major contribution of poor governance and corruption to this growing problem[34]	2015	Human	EARS-Net (AMR); ESAC (antibiotic use); International Country Risk Guide (corruption); World Development Indicators-World Bank (other indicators)	28 countries	EU	1998-2010	Improved governance at the national level could be fundamental in confronting AMR; results suggest governance is an even more important determinant of AMR than antibiotic use in people
Antimicrobial susceptibility profiles of anaerobic bacteria, isolated from human clinical specimens, within different European and surrounding countries. A joint ESGAI study[35]	2020	Human	Participating laboratories	3 countries	WHO EUR & Kuwait	2017	The antimicrobial susceptibility profile of anaerobic bacteria differs remarkably between different countries and that unexpected resistance patterns can be observed; This data set confirms that the antimicrobial resistance rates are highest among gram-negative anaerobic bacteria
Antimicrobial utilization and resistance in Pseudomonas aeruginosa using segmented regression analysis: a comparative study between Serbia and eight European Countries[36]	2023	Human	MMDAS; CAESA; ECDC; National databases	9 countries	WHO EUR	2015-2020	Even though decreasing or stabilising utilization trends have been noted for most of the studied antibiotics for Serbia, the level of utilization is still high compared to the other European countries
Are There Striking Differences in Outpatient Use of Antibiotics Between South Backa District, Serbia, and Some Scandinavian Countries?[37]	2018	Human	Data obtained from state-owned and private pharmacies	2 countries, 1 region	WHO EUR (HMIC)	2008	Interventions to improve use and education on rational antibiotic use is essential
Assessing transparency and accountability of national action plans on antimicrobial resistance in 15 African countries[38]	2022	Multisectoral	Government websites; Google; Anderson et al. (2019)	15 countries	WHO AFR (LMIC)	2020	Biggest challenges in LMIC are implementation not formulation of NAPs; a detailed NAP is not a measure of success in itself; transparency and accountability important for bridging planning-action gap
Assigning Defined Daily/Course Doses for Antimicrobials in Turkeys to Enable a Cross-Country Quantification and Comparison of	2021	Animal	EU-FP7 EFFORT	3 countries	EU (HIC)	2014-2016	Need for a unified European antimicrobial drug portfolio and harmonization of recommended doses across the SPCs of similar antimicrobial products authorized for use in turkeys, at the least

Antimicrobial Use[39]							when treating identical diseases
Association between antibiotic resistance in intensive care unit (ICU)-acquired infections and excess resource utilization: evidence from Spain, Italy, and Portugal[40]	2021	Human	TESSy	3 countries	EU (HIC)	2008-2016	A significant overall excess LOS and a substantial economic burden of resistant ICU-acquired infections compared to their susceptible counterparts
Attention to the Tripartite's one health measures in national action plans on antimicrobial resistance[41]	2021	Human, Environmental and Multisectoral	Global Database for Antimicrobial Resistance Country Self-Assessment	77 countries	Global	-2020	We have shown how the findings can be triangulated with other indicators, in this case with self-reported data on OH collaboration, to reveal new insights. This exercise has helped us identify a discrepancy between attention to OH measures in the national policy document vis-à-vis in OH collaboration
Awareness regarding antimicrobial resistance and confidence to prescribe antibiotics in dentistry: a cross-continental student survey[42]	2022	Multisectoral	Derived from questionnaire posited to participants (dental students)	3 countries	Global (HMIC)	2022	Despite high awareness of antibiotic resistance among dental students, there is limited understanding of how human health and the environment are interlinked (WHO One Health concept); more education needed
Baseline evaluation of the World Health Organization (WHO) infection prevention and control (IPC) core components in Pacific Island Countries and Territories (PICTs)[43]	2024	Multisectoral	Cross-sectional survey study	15 countries and territories	PICTs	2022-2023	Place greater emphasis on reducing IPC gaps through enforcing national and facility level IPC programmes and guidelines, dedicating adequate budgets to IPC improvement, providing mandatory IPC education for healthcare workers, and conducting HAI surveillance within healthcare facilities
Benchmarking national action plans on antimicrobial resistance in eight selected LMICs: Focus on the veterinary sector strategies[44]	2020	Animal and Multisectoral	WHO library of NAPs; WHO Manual for designing NAPs	8 countries	Global (LMIC)	2015-2018	There are gaps in key areas such as finance, targets and legislation for reducing antimicrobial use in the veterinary sector and medicine quality assurance
Capturing data on antimicrobial resistance patterns and trends in use	2023	Multisectoral	CAPTURA	12 countries	WHO SEAR/WPR/EMR (Pakistan)	2019-2022	The project has paved the way for expansion of surveillance networks to include both the

in regions of Asia (CAPTURA)[45]							academic and private sector in several countries and has actively engaged in discussions to promote data sharing at the local, national, and regional levels
Carbapenem Resistance in Animal-Environment-Food from Africa: A Systematic Review, Recommendations and Perspectives[46]	2024	Multisectoral	Google Scholar; African Journals Online; ResearchGate; PubMed; Embase; Scopus	25 countries and territories	WHO EMR/AFR	2009-2023	Animal-environment-food ecosystems would constitute reservoirs of CRBs involved in human infections; Public health authorities should focus their efforts on preventing the transmission of CRBs from animal-environment-food ecosystems to humans and invest more in the acquisition and use of genomics platforms in Africa
Carbapenemase-producing Enterobacteriaceae in Europe: assessment by national experts from 38 countries, May 2015[47]	2015	Human	EuSCAPE, EARS-net	38 countries	Participants of EuSCAPE (EU, IS, NO, 7 EU enlargement states and IL)	2013-2015	EuSCAPE project confirm the need to develop an EU-wide system for surveillance of high-risk CPE clones and mobile genetic vectors across European healthcare systems.
Clinical management of severe infections caused by carbapenem-resistant gram-negative bacteria: a worldwide cross-sectional survey addressing the use of antibiotic combinations[48]	2022	Human and Multisectoral	Cross-sectional survey questionnaire	95 countries	Global	2019	Unequal access to diagnostic and therapeutic resources and the unavailability of evidence-based recommendations were two strong determinants contributing to this heterogeneity; Additionally, the lack of a universally accepted definition of 'combination therapy' might have further impaired the confidence in results from available clinical studies
Clinical outcomes and bacterial characteristics of carbapenem-resistant Klebsiella pneumoniae complex among patients from different global regions (CRACKLE-2): a prospective, multicentre, cohort study[49]	2022	Human	CRACKLE-2	8 countries	Global	2017-2018	Clinical outcomes in patients with CRKP infections are probably driven by acute and chronic levels of illness and vary according to region; These findings raise questions about the external generalisability of clinical studies on CRKP done in any specific global region
Clinical practice guidelines for acute otitis media in children: a	2020	Human	Medline; Embase; Cochrane library; guidelines International Network; Trip Medical Database; European	17 countries	EU/EFTA/UK & USA	2000-2018	Existing European guidelines scored poorly in most AGREE II domains, including items related

systematic review and appraisal of European national guidelines[50]			Paediatric Association; American Association of Paediatrics; WHO guidelines				to how evidence was gathered and appraised; Consideration of country-specific antibiotic resistance patterns appears to be limited
Coming of age: governance challenges in updated AMR national action plans in the EU[51]	2024	Multisectoral	WHO library of NAPs	13 countries	EU	2024	EU states becoming increasingly knowledgeable of AMR causes, consequences and methods of addressing it; Countries becoming more aware of One Health approach; Noticeable development relating to governance issues; international cooperation in Europe could be more active
Comparative Analysis of Outpatient Antibiotic Prescribing in Early Life: A Population-Based Study Across Birth Cohorts in Denmark and Germany[52]	2024	Human	Danish National Prescription Registry; GePaRD	2 countries (Denmark & Germany)	EU (HIC)	2004-2018	Over time, positive changes occurred towards more rational antibiotic prescribing in early life in both countries, particularly for children born in 2010 and later
Comparing Farm Biosecurity and Antimicrobial Use in High-Antimicrobial-Consuming Broiler and Pig Farms in the Belgian-Dutch Border Region[53]	2020	Animal	Data collected from farm visits (total n=60)	2 countries (Belgium and Netherlands)	EU	2017-2018	Reduction targets for AMU on a national level can drive reduction of AMU on farm level; improved biosecurity a component of many different management, housing and feeding measures
Comparison of Antimicrobial Consumption Patterns in the Swiss and Danish Cattle and Swine Production (2007-2013)[54]	2017	Animal	DANMAP, Swiss data obtained from Regula et al. (2009) and FSVO, animal pop data from ESVAC	2 countries (Denmark and Switzerland)	EU/EFTA (HIC)	2007-2013	AM consumption in CH cattle population was more than twice as high as observed in DK; also higher for swine; introduction of the 2010 'Yellow Card' system in DK led to a big drop in AM consumption in pigs and cattle in 2010 and 2011
Comparison of governance approaches for the control of antimicrobial resistance: Analysis of three European countries[55]	2018	Multisectoral	Archival data of previous research conducted by the research team; other published and grey literature	3 countries	EU/UK (HIC)	2018	Effective implementation of governance approaches may require the extensive involvement of various actors from the national to the frontline level and a level of engagement with the issue of AMR by the public
Comparison of national	2018	Human	Public Health England; JANIS	2 countries (Japan &	Global (HIC)	2000-2017	Policy interventions need to be

strategies to reduce meticillin-resistant Staphylococcus aureus infections in Japan and England[56]				England)			relevant to local epidemiological trends, while acceptable within the health system, culture, and public expectations
Correlation between veterinary antimicrobial use and antimicrobial resistance in food-producing animals: a report on seven countries[57]	2013	Animal	ESVAC	7 countries	EU/EFTA	2010	High correlations for all the antimicrobial classes studied
Cost-Effectiveness of Test-and-Treat Strategies to Reduce the Antibiotic Prescription Rate for Acute Febrile Illness in Primary Healthcare Clinics in Africa[58]	2024	Human	ADIP accelerator trial	3 countries	WHO AFR	2020-2022	The implementation of diagnostic tests for more appropriate antibiotic prescriptions is effective, but requires a WTP per ppt reduction in the APR to decide on the most cost-effective strategy, and should be part of an integrated stewardship program to realise its full potential
Determinants of worldwide antibiotic resistance dynamics across drug-bacterium pairs: a multivariable spatial-temporal analysis using ATLAS[59]	2023	Human, Environmental and Multisectoral	ATLAS	51 countries	Global	2006-2019	ABR rates are highly dependent on the country and, most importantly, on the drug–bacterium pair considered; Results from our statistical analysis suggested that factors associated with ABR rates were different across drug–bacterium pairs but more similar within a bacterial species, reflecting different underlying ecological behaviour
Development of a cross-sectoral antimicrobial resistance capability assessment framework[60]	2024	Human, Animal and Multisectoral	Reviewed/adapted from existing tools sourced from Google; Google Scholar; SCOPUS; Medline; CABI Global Health	4 countries	PICTS	2020-2021	The Framework is flexible to meet the needs of implementers, as tools can be used separately to assess the capacity of individual institutions or as a whole to align priority-setting and capacity-building with National AMR Action Plans or national policies
Differences in epidemiology of candidaemia in the Nordic countries - what is to blame?[61]	2016	Human	National Reference Laboratories for Medical Mycology; NIDR-NIHW; Surveillance programmes (DEN, NOR)	4 countries	WHO EUR (Nordic)	2010-2011	Differences between the Nordic countries in the incidence of haematological malignancies and the use of certain antimicrobial drugs may contribute to the

							changing Candida epidemiology.
Different microbial and resistance patterns in primary total knee arthroplasty infections - a report on 283 patients from Lithuania and Sweden[62]	2021	Human	SKAR; LAR	2 countries (Lithuania & Sweden)	EU (HIC)	2011-2020	Various factors such as patient related, societal and local hospital factors might be related to the observed differences but needs to be confirmed in future longer studies with larger comparable cohorts
Does gonorrhoea screening intensity play a role in the early selection of antimicrobial resistance in men who have sex with men (MSM)? a comparative study of Belgium and the United Kingdom[63]	2018	Human	NRC-STI; GRASP	2 countries (Belgium & UK)	WHO EUR (HIC)	2010-2014	NG spends proportionately more time in the oropharynx and rectum in MSM and may offer it more opportunities for acquisition of resistance genes and mutations.
Effective stakeholder engagement for collation, analysis and expansion of antimicrobial resistance (AMR) data: a CAPTURA experience[64]	2023	Multisectoral	CAPTURA	7 countries	WHO SEAR/WPR	2019-2022	Close collaboration with the existing national mechanisms for identifying AMR data sources was crucial for the project's success; There remain critical gaps in data generation/management practice and analysis capacity for AMR data at most facilities
EPI-Net One Health reporting guideline for antimicrobial consumption and resistance surveillance data: a Delphi approach[65]	2022	Human, Animal and Environmental	Delphi study	20 countries	Global	2021-2022	We believe the document can support discussion among major international and national stakeholders for coordinated One Health surveillance reporting from the human, animal, and environmental sectors within national and regional plans for antibiotic policy to reduce the burden of AMR
Essential and forgotten antibiotics: an inventory in low- and middle-income countries[66]	2019	Human	Internet-based cross-sectional survey	28 countries	Global (LMIC)	2019	Most of WHO-EML 'access' antibiotics are approved in the 28 surveyed LMICs, including the most relevant paediatric formulations; Many 'forgotten' antibiotics are not approved in these countries, despite their important role in some specific clinical conditions, particularly in areas with high prevalence of

							MDR bacteria
Estimating global trends in total and childhood antibiotic consumption, 2011-2015[67]	2019	Human	IQVIA MIDAS	75 countries	Global	2011-2015	Global antibiotic consumption changed relatively little between 2011 and 2015
Estimating the subnational prevalence of antimicrobial resistant Salmonella enterica serovars Typhi and Paratyphi A infections in 75 endemic countries, 1990-2019: a modelling study[68]	2024	Human	TSAP, SEAP, STRATAA, GBD, literature	75 countries	Global (LMIC endemic countries - incidence rate of at least 10 cases per 100,000 a year)	1990-2019	Control of enteric fever will ultimately hinge on improvements in WASH; Current pace of WASH progress is projected to be too slow to achieve 2030 SDG targets
European antibiotic awareness day: a five-year perspective of Europe-wide actions to promote prudent use of antibiotics[69]	2014	Multisectoral	EAAD	43 countries	WHO EUR	2008-2012	Looking to the future, self-medication with antibiotics has been identified as a new focus for EAAD 2014. Concerns about AMR and the need for more prudent use of antibiotics are of global significance and on political agendas.
European survey on the current surveillance practices, management guidelines, treatment pathways and heterogeneity of testing of Clostridioides difficile, 2018-2019: results from the Combatting Bacterial Resistance in Europe CDI (COMBACTE)[70]	2023	Human	Survey questionnaire	12 countries	EU/UK	2018-2019	Data from this survey show good awareness of CDI testing and treatment guidelines in hospital sites across Europe; However, there are still disparities between countries and insufficient awareness of infection control measures for suspected CDI
European-wide antimicrobial resistance monitoring in commensal Escherichia coli isolated from healthy food animals between 2004 and 2018[71]	2022	Animal	EASSA studies (2004-05; 2008-09; 2013-14; 2017-18)	10 countries	EU	2004-2018	Studying AMR in commensal indicator E. coli from intestinal content of healthy food-producing animals provides information on the reservoirs of resistant bacteria that can potentially be transferred between animals and between animals and humans
Evaluating the contribution of antimicrobial use in farmed animals to global	2023	Human	FAO, CDDEP	30 countries	HIC and LMIC, large animal producer countries	2010-2020	Positive association between AMU in farmed animals and AMR in humans; importance of AMR spread between countries

antimicrobial resistance in humans[72]							(cross-border effects); many countries need to improve enforcement of laws and regulations regarding AMU for farm animals
Evaluating the yaws diagnostic gap: a survey to determine the capacity of and barriers to improving diagnostics in all yaws-endemic countries[73]	2022	Human	2-part Diagnostic Gap Survey	14 countries	Yaws-endemic countries	2021-2022	Countries in need of a sustainable supply of serological tests, development of molecular testing facilities
Exploring the antimicrobial stewardship educational needs of healthcare students and the potential of an antimicrobial prescribing app as an educational tool in selected African countries[74]	2022	Human	59-item questionnaire	10 countries	WHO AFR	2021	Poor knowledge of AMS and limited access to relevant info shows there could be value in educational tools in mobile application form (e.g., CwPAMS app)
Extended spectrum beta -lactamase producers among nosocomial Enterobacteriaceae in Latin America[75]	2014	Human	Literature review (PubMed)	17 countries	WHO AMR (Latin and Central America)	2005-2013	Patients have survival advantage when correct AM agent is administered as initial therapy; patient-to-patient transmission could play an important role in L. American healthcare.
First assessment of the knowledge, attitudes, and practices of health actors in Togo and Ivory Coast in regard to antibiotic resistance[76]	2022	Human	Cross-sectional descriptive questionnaire	2 countries (Togo & Ivory Coast)	WHO AFR (LMIC)	2020-2021	Health actors in Togo and Ivory Coast have a good knowledge and perception in regard to antibiotics and ABR, but also follow inadequate practices
Five-year Pan-European, longitudinal surveillance of Clostridium difficile ribotype prevalence and antimicrobial resistance: the extended ClosER study[77]	2019	Human	ClosER study	28 countries	EU/UK	2011-2016	Overall ribotype prevalence across Europe remained stable between 2011 and 2016, and a lack of ribotype diversity in an individual country was associated with greater AMR
Genomic surveillance and antimicrobial resistance determinants in Neisseria gonorrhoeae isolates from Uganda, Malawi	2023	Human	Isolates obtained from sites in each country	3 countries	WHO AFR (LMIC)	2015-2020	High prevalence of resistance to ciprofloxacin (and empirical use continues), tetracycline and benzylpenicillin, and the emerging resistance determinants for

and South Africa, 2015-20[78]							azithromycin show that it is imperative to strengthen gonococcal AMR surveillance, ideally including genomics, in African countries
Global antibiotic consumption 2000 to 2010: an analysis of national pharmaceutical sales data[79]	2014	Human	MIDAS	71 countries	Global	2000; 2010	Although HIC consume more antibiotics per person, consumption rates are rising rapidly in emerging economies and represent the bulk of the increase in consumption over the decade 2000–10
Global antibiotic consumption and usage in humans, 2000-18: a spatial modelling study[80]	2021	Human	IQVIA, WHO, ESAC-Net, USAID (DHS), UNICEF (MICS)	204 countries (consumption) 101 countries (usage)	Global (consumption) LMIC (usage)	2000-2018	Lack of access to antibiotics as well as their inappropriate use need addressing
Global Antimicrobial Resistance and Use Surveillance System on the African continent: Early implementation 2017-2019[81]	2022	Human	GLASS	23 countries	WHO AFR/EMR	2017-2019	The evidence generated is supporting the identification of areas for further research – AMR burden in healthcare settings, improvement of diagnostic stewardship, and AMR in the human animal environment interface – and it is advocating for the continuous support of actions directed to AMR monitoring and control
Global antimicrobial resistance: a system-wide comprehensive investigation using the Global One Health Index[82]	2022	Human and Multisectoral	GLASS; GHS; TrACCS; ECDC; CDC; CARSS	146 countries	Global	2022	Establishing laboratory infrastructure and multidisciplinary platforms rapidly will be critical to addressing the enormous burden of AMR; Simultaneously, this will urgently require more extensive surveillance of AMR in humans, animals, and the surrounding environment
Global approaches to tackling antimicrobial resistance: a comprehensive analysis of water, sanitation and hygiene policies[83]	2024	Environmental	FAO LEX	193 countries	Global	2024	AMR is a threat that must be addressed through a broad continuum of action, with a critical reliance on policy, laws and regulations at the national and subnational levels
Global burden of bacterial antimicrobial	2022	Human	GBD; pharma companies running surveillance networks, diagnostic	204 countries and territories	Global	1990-2021	AMR burden forecasted to increase by 2050, continued

resistance 1990-2021: a systematic analysis with forecasts to 2050[84]			labs and clinical trials; researchers; smaller studies; research institutes based in LMICs; public and private hospitals and public health institutes; global and national surveillance networks				reductions in AMR mortality for children >5 will be outpaced by increase in mortality in other age groups, overall improvements to health-care systems and access to existing antibiotics could be far more impactful than drug development alone
Global diversity and antimicrobial resistance of typhoid fever pathogens: Insights from a meta-analysis of 13,000 Salmonella Typhi genomes[85]	2023	Human	ENA; NCBI-SRA;GenBank; Enterobase; GTGC	21 world regions	Global	2010-2020	Widespread use of TCVs can help to further reduce global incidence of typhoid fever; Vaccines are an even more important tool to mitigate the public health burden of AMR Typhi.
Global incidence in hospital-associated infections resistant to antibiotics: An analysis of point prevalence surveys from 99 countries[86]	2023	Human	Hospitalisation rates: OECD for 78 countries, national registries for others, inferred for 26 countries from the OECD78; HARIs per year estimated as the product of population size, proportion of pop hospitalised each year, probability that a patient was hospitalised for an AMR infection	195 countries	Global	2010-2020	MIC have the largest burden of HARIs - lack of antibiotic stewardship, lack of personal accountability from medical personnel might lead to erratic prescribing behaviour - overuse of antibiotics also might be a result of their ready availability; hospital hygiene associated with infection rates, esp. in LMICs; overuse of antibiotics in HIC
Global overview of national regulations for antibiotic use in aquaculture production[87]	2024	Animal	National steering documents; research from IGOs & NGOs; Elsevier ScienceDirect; JSTOR; PubMed; Google Scholar; Taylor & Francis Online; Wiley Online Library	17 countries and regions	Global	N/A	One important steppingstone in minimizing antibiotic use is to have a thorough prescriptive management system in place with continuous analysis of animal and environmental health in the farm. This includes but is not limited to, using pre- and probiotics, re-assessing and optimizing feed composition, optimizing stocking density, and limiting human interaction (handling)
Global patterns and correlates in the emergence of antimicrobial resistance in humans[88]	2023	Human	https://zenodo.org/record/4924992	95 countries	Global	2006-2017	The relationship between AMR emergence and antibiotic consumption in livestock is modified by GDP, with only higher GDP countries showing a slight positive association, a finding that differs from previous

							studies on the drivers of AMR prevalence
Global research publications on irrational use of antimicrobials: call for more research to contain antimicrobial resistance[89]	2021	Multisectoral	Scopus	105 countries	Global	1980-2020	The bulk of research on the irrational use of antimicrobials originated from a limited number of countries and the bulk of research publications focused on misuse and self-medication with antibiotics in the context of community pharmacy practice; The remaining bulk of research publications focused on hospital settings and the role of antimicrobial stewardship
Global trend of antimicrobial resistance in common bacterial pathogens in response to antibiotic consumption[90]	2023	Human	Antimicrobial resistance: global report on surveillance (WHO, 2014)	61 countries	Global	2009-2012	LMICs presented relatively higher resistance rates in common bacterial pathogens but lower antibiotic consumption rates, as well as the potentially inappropriate antibiotic consumption compared with HICs
Global trends in antimicrobial use in aquaculture[91]	2020	Animal	FAO; FishStat	32 countries	Global	2017; 2030	Increases in use may be particularly significant in geographies with nascent antimicrobial consumption surveillance, regulatory and enforcement capacities
Global trends in antimicrobial use in food-producing animals: 2020 to 2030[92]	2023	Animal	Literature review	10 countries	Global	2020-2030	Availability of national data would enable comparisons within regions that have large discrepancies between countries, and perhaps more importantly, relate antibiotic stewardship policies (or absence thereof) with accurate AMU levels; Until these data are publicly available, for any national level analysis of the impact of AMU we continue to rely on modelling estimates.
Healthcare-associated infections in intensive care units in Taiwan, South Korea, and Japan: recent trends based on national surveillance	2018	Human	PubMed; Google Scholar; TNIS; KONIS; JANIS; World Bank	3 countries	WHO WPR (HIC)	2008-2015	The overall decrease in HAI appears to be due to improved surveillance coupled with a series of interventions in each country.

reports[93]							
How far has the globe gone in achieving One Health? Current evidence and policy implications based on global One Health index[94]	2024	Human, Animal, Environmental and Multisectoral	FAO	160 countries & territories	Global	2022	Overall, global capacity building of One Health requires an international cooperation scheme to foster extensive information sharing and experience exchange among countries/territories on common issues. On key issues, leading countries/territories should increase aid to struggling areas and accelerate the development of a joint global response to One Health by dispatching experts and developing pilot projects
Identifying AWaRe indicators for appropriate antibiotic use: a narrative review[95]	2024	Human	Medline	N/A	Global	1996-2023	Lack of discrete and dedicated QIs based on the WHO AWaRe system highlights the need to develop and test indicators based directly on the AWaRe system focused on feasible integration and implementation into both local and national ASPs
Identifying the drivers of multidrug-resistant Klebsiella pneumoniae at a European level[96]	2021	Human	ECDC; WHO	11 countries	EU	2005-2015	Both consumption and transmission rates in the hospital are critical drivers for the spread of resistance
In Vitro Activity of Ceftazidime-Avibactam against Clinical Isolates of Enterobacteriaceae and Pseudomonas aeruginosa Collected in Asia-Pacific Countries: Results from the INFORM Global Surveillance Program, 2012 to 2015[97]	2018	Human	INFORM global surveillance programme	9 countries	WHO SEAR/WPR	2012-2015	The differences in antimicrobial susceptibilities observed across countries in a specific geographic region, such as the Asia-Pacific region, demonstrate the importance of performing regional surveillance.
In Vitro Activity of Ceftazidime-Avibactam against Clinical Isolates of Enterobacteriaceae and Pseudomonas aeruginosa Collected in Latin American Countries: Results from	2019	Human	International Network for Optimal Resistance Monitoring (INFORM) global surveillance program	6 countries	WHO AMR (Latin America)	2012-2015	Clinical isolates of Enterobacteriaceae from six WHO AMR (Latin America)n countries in 2012 to 2015 were highly susceptible to ceftazidime-avibactam (99.0% susceptible) and that ceftazidime-avibactam was more active than currently

the INFORM Global Surveillance Program, 2012 to 2015[98]							available antimicrobial agents of last resort (e.g., amikacin, colistin, and tigecycline). Only 17 isolates of Enterobacteriaceae (0.2% of all isolates tested) carried an MBL. The current study demonstrated ceftazidime-avibactam to be a potent agent against ceftazidime-non-susceptible, meropenem-non-susceptible, colistin-resistant, and MDR isolates of Enterobacteriaceae. Ceftazidime-avibactam (87.4% susceptible) was the second most potent agent tested against isolates of P. aeruginosa from six WHO AMR (Latin American) countries in 2012 to 2015, after colistin (94.9% susceptible).
In vitro activity of ceftazidime-avibactam against enterobacterales and Pseudomonas aeruginosa isolates collected in Latin America as part of the ATLAS global surveillance program, 2017-2019[99]	2021	Human	ATLAS	10 countries	WHO AMR (Latin & Central America)	2017-2019	Ceftazidime-avibactam has retained its in vitro potency against clinical isolates of Enterobacterales and P. aeruginosa collected from hospitalized patients in Latin American countries since 2012; Regional and country prevalence of different carbapenem-resistance mechanisms do exist and must be considered when evaluating treatment options
In vitro activity of imipenem/relebactam against non-Morganellaceae Enterobacterales and Pseudomonas aeruginosa in Latin America: SMART 2018–2020[100]	2023	Human	SMART global surveillance programme	9 countries	WHO AMR (Central & Latin America)	2018-2020	Increases in the prevalence of MBL-positive isolates of Gram-negative bacilli may be occurring in Latin America and will pose treatment challenges for all newer b-lactam/b-lactamase inhibitor combinations, including IMR
In vitro activity of imipenem/relebactam against piperacillin/tazobactam-resistant and meropenem-resistant non-	2023	Human	SMART global surveillance programme	6 countries	WHO EUR (HIC)	2018-2020	Imipenem/relebactam appears to be a potential treatment option for lower respiratory tract infections caused by piperacillin/tazobactam- and meropenem-resistant NME and P.

Morganellaceae Enterobacterales and Pseudomonas aeruginosa collected from patients with lower respiratory tract infections in Western Europe: SMART 2018-20[101]							aeruginosa
In Vitro Susceptibility of Global Surveillance Isolates of Pseudomonas aeruginosa to Ceftazidime-Avibactam (INFORM 2012 to 2014)[102]	2016	Human	INFORM	39 countries	Global	2012-2014	The in vitro behaviour of ceftazidime-avibactam against P. aeruginosa, yielding percentages of susceptibility higher than those of other -lactams tested, similar to those of amikacin, but lower than those of colistin, supports an ongoing clinical trial of this agent in patients with nosocomial pneumonia, including ventilated patients
Individual and institutional predisposing factors of MRSA surgical site infection and outcomes-a retrospective case-control-study in 14 European high-volume surgical centres[103]	2024	Human	SALT	5 countries	WHO EUR (HIC)	2016	Most concerning is the incomplete implementation of best practices approaches including ABS programmes and ID consultancy
Inventory of antibiotic stewardship programs in general practice in France and abroad[104]	2015	Multisectoral	Survey questionnaire; Medline; Embase; Cochrane Library; Google Scholar	18 countries	Global (HIC)	2000-2014	Educational and persuasive measures are certainly needed, but the use of restrictive and organizational measures appear mandatory if the number of antibiotic prescriptions is to be decreased in the near future
Investigating the feasibility and potential of combining industry AMR monitoring systems: a comparison with WHO GLASS[105]	2024	Human and Multisectoral	WHO GLASS; ATLAS; GEARS; SIDEROWT; KEYSTONE; DREAM; SOAR	85 countries	Global	2017	High agreement values for available comparisons with GLASS suggest that data for other bacteria-antibiotic-country-year combinations only present in industry systems could complement GLASS
Isomorphic dynamics in national action plans on antimicrobial resistance[106]	2021	Multisectoral	WHO library of NAPs	59 countries	Global	2019	We found isomorphic mimicry to be a pronounced dynamic in AMR NAPs produced by several LICs and LMICs displaying alignment

							with the GAP guidelines in form but not in function
Livestock-associated meticillin-resistant Staphylococcus aureus (MRSA) among human MRSA isolates, European Union/European Economic Area countries, 2013[107]	2018	Human	National or regional reference laboratories	5 countries (Belgium, Denmark, Spain, the Netherlands and Slovenia).	EEA	2013	LA-MRSA represented 3.9% of 13,756 typed MRSA human isolates, but it represented ≥ 10% in five countries
Mandatory surveillance and outbreaks reporting of the WHO priority pathogens for research & discovery of new antibiotics in European countries[108]	2020	Human	National databases	32 countries	EU/EFTA	2019	Few countries have implemented mandatory surveillance and outbreak reporting of the WHO priority pathogens; standardisation of data collection and harmonisation of surveillance results publication frequency are necessary
Markers of epidemiological success of methicillin-resistant Staphylococcus aureus isolates in European populations[109]	2023	Human	MRSA infection incidence (Cassini et al.); data on antibiotic use extracted from ECDC database	29 countries	EU/EFTA	2015	Significant correlation between community usage of beta lactam and quinolone, and MRSA incidence; a standardised framework for MRSA sampling badly needed to establish cohesive sample and data collection, such as: common reasons for collecting isolates and source of isolates, harmonised typing and accessible strains
Measuring hospital antibiotic consumption in EU/EEA countries: comparison of different metrics, 2017 to 2021[110]	2024	Human	TESSy; Eurostat	24 countries	EEA	2017-2021	The study underscores the importance of using hospital activity-based denominators such as ‘bed-days’ and ‘discharges’ when evaluating hospital antibiotic consumption
Measuring the global response to antimicrobial resistance, 2020-21: a systematic governance analysis of 114 countries[111]	2023	Multisectoral	TrACCS; WHO library of NAPs; FDI WDF; ECDC; FAO; AAR; Anderson et al. (2019); GLASS; WHO 2018 SEAR SA; Global AMR R&D Hub	114 countries	Global	2020-2021	In LMICs activities identified in NAPs often lack sustainable domestic financing for operationalisation, instead relying on funds from foreign donors.
Modulation of multidrug-resistant clone success in Escherichia coli populations: a	2024	Human	BSAC	1 country (UK)	WHO EUR (HIC)	2001-2017	Benefits gained by bacteria from AMR do not manifest themselves uniformly across different clones or classes of antibiotics

longitudinal, multi-country, genomic and antibiotic usage cohort study[112]							
Monitoring Antimicrobial Resistance and Drug Usage in the Human and Livestock Sector and Foodborne Antimicrobial Resistance in Six European Countries[113]	2020	Human, Animal, Environmental and Multisectoral	PubMed; questionnaire	6 countries	EEA/UK	2018	Efforts should be made to improve standardization and harmonization and allow more meaningful analyses of AMR and AMU surveillance data under a One Health approach
Monitoring progress on Antimicrobial Resistance (AMR) response in the World Health Organization African region: Insights from the Tracking AMR Country Self-Assessment Survey (TrACSS) 2021 results for the human health sector[114]	2023	Multisectoral	TrACSS	41 countries	WHO AFR	2021	Sharing experiences and best practices stories ought to be encouraged for cross-country learning and collective regional action and coordination
Moving from assessments to implementation: promising practices for strengthening multisectoral antimicrobial resistance containment capacity[115]	2023	Multisectoral	Literature review	13 countries	WHO AFR/SEAR (Bangladesh)	2014-2022	As LMICs continue to tackle the growing threat of AMR using the WHO benchmarks and other tools, sharing lessons learned and best practices and highlighting critical gaps will continually improve the technical resources available to strengthen their AMR containment capacity
Multidisciplinary and multisectoral coalitions as catalysts for action against antimicrobial resistance: Implementation experiences at national and regional levels[116]	2018	Multisectoral	Programme developed by study authors	3 countries	WHO AFR (LIC)	2004 (Zambia); 2006 (Ethiopia); 2013 (Namibia)	Additional stakeholders from different disciplines and sectors who were then able to generate a shared vision, foster the coalition's development, and mount organised local and regional advocacy and action against AMR
Mycoplasma pneumoniae infections, 11 countries in Europe and Israel, 2011 to 2016[117]	2020	Human	ESGMI survey (May 2016)	12 countries	WHO EUR	2011-2016	No standardised method for detecting M. pneumoniae infection; macrolide resistance screening is sporadic
National action plans for antimicrobial resistance and variations in	2023	Multisectoral	Diallo et al., 2020; WHO library of NAPs; Google	N/A	Global	2022	The considerable heterogeneity of nationally submitted data to commonly used antimicrobial

surveillance data platforms[118]							resistance surveillance platforms compromises their validity, thus undermining local and global antimicrobial resistance strategies
National action plans on antimicrobial resistance in Latin America: an analysis via a governance framework[119]	2024	Multisectoral	WHO AMR Library, governmental websites by time of publication of TrACSS 2021, Governance framework from Anderson et al. (2019)	11 countries	WHO AMR (Latin America)	2015-2021	Strategic objectives and many governance aspects (coordination, participation, equity) addressed by all NAPs; monitoring and evaluation, activities in the environment sector need addressing in future NAPs
National disparities in the relationship between antimicrobial resistance and antimicrobial consumption in Europe: an observational study in 29 countries[120]	2017	Human	ESAC-Net	29 countries	EEA	2013	This study has highlighted the strength of association between total and specific community consumption rates of antibiotics and resistance rates in up to 20 strains of resistant bacteria across 29 European countries
OH-EpiCap: A semi-quantitative tool for the evaluation of One Health epidemiological surveillance capacities and capabilities[121]	2023	Multisectoral	OH EpiCap tool	6 countries	EU (HIC)	2022	The tool supports the diagnostic of strengths and weaknesses in multi-sectoral collaborations and helps to identify concrete and direct actions to improve collaborative activities at all steps of surveillance
Opinions of veterinarians on antimicrobial use in farm animals in Flanders and the Netherlands[122]	2016	Animal	Questionnaire	1 country (NED) & 1 region (Flanders)	EU	2012	Cultural differences (level of risk averseness, characteristics of the veterinarian-client relationship) in combination with diverging policies towards veterinary AMU in Belgium and the Netherlands seem to be the basis for these differences
Out-of-pocket health expenditures and antimicrobial resistance in low-income and middle-income countries: an economic analysis[123]	2015	Human, Animal and Multisectoral	WHO 2014 Antibacterial Resistance Global Surveillance Report; World Bank	47 countries	Global (LMIC)	2014	Our data suggest cost-sharing of antimicrobials in the public sector might drive demand to the private sector in which supply-side incentives to overprescribe are probably heightened and quality assurance less standardised
Pathogen diversity and antimicrobial resistance transmission of Salmonella enterica serovars Typhi and	2024	Human	STRATAA surveillance sites	3 countries	Global (LMIC)	2016-2019	The burden of drug-resistant enteric fever at the study sites is currently caused mainly by transmission of locally established variants, and transmits across age

Paratyphi A in Bangladesh, Nepal, and Malawi: a genomic epidemiological study[124]							groups; Childhood immunisation programmes can be expected to reduce the overall burden of resistant infections in endemic settings
Patient access in 14 high-income countries to new antibacterials approved by the US Food and Drug Administration, European medicines agency, Japanese pharmaceuticals and medical devices agency, or health Canada, 2010-2020[125]	2021	Human	Government websites; WHO EML/AWaRe; Pew Charitable Trusts	14 countries	Global (HIC)	2010-2020	Patient access to new antibacterials also limited in HIC; new economic incentives that are delinked from unit sales are necessary
Patterns of dental antibiotic prescribing in 2017: Australia, England, United States, and British Columbia (Canada)[126]	2021	Human	Department of Health (AU); NHS Digital Prescription Cost Analysis (GB); IQVIA LRx (US); BC Ministry of Health (CA)	3 countries & 1 territory	Commonwealth (HIC) & USA	2017	Significant opportunities exist for the global dental community to contribute to international efforts to tackle antibiotic resistance, including by changing from broad-spectrum antibiotics to narrower-spectrum antibiotics and by reducing the use of WHO 'Watch' antibiotics
Perceptions of antimicrobial usage, antimicrobial resistance and policy measures to reduce antimicrobial usage in convenient samples of Belgian, French, German, Swedish and Swiss pig farmers[127]	2015	Animal	Survey part of MINAPIG	5 countries	EU/EFTA	2012-2013	Financial policy measures are most likely to influence the antimicrobial usage in the pig sector
Perspectives on the Regional Strategy for Implementation of National Action Plans on Antimicrobial Resistance in the WHO African Region[128]	2024	Human and Multisectoral	Cross-sectional online survey	36 countries	WHO AFR	2023-2024	Need to enhance awareness campaigns, support the establishment and functioning of AMR evaluation and monitoring systems, and build the capacity of AMR staff with cost-benefit analysis and budgeting skills.
Phase I of the Surveillance for Enteric Fever in Asia Project (SEAP): An Overview and	2018	Human	Available data from the selected hospitals	4 countries	South Asia	2012-2016	Combination of WASH interventions and vaccination may eliminate typhoid in settings where it is endemic

Lessons Learned[129]							
Policy implementation for methicillin-resistant Staphylococcus aureus in seven European countries: a comparative analysis from 1999 to 2015[130]	2017	Human and Animal	Europe-wide surveillance data on infectious diseases (EARS-Net), antibiotic consumption (ESAC-Net), and veterinary medicine (ESVAC).	7 countries	EU	1999-2015	There were similar but different health policy implications in the seven countries regarding LA- and HA-MRSA. Although causation could not be defined, some policies such as mandatory surveillance may be helpful for countries that have yet to implement an MRSA policy.
Population-level faecal metagenomic profiling as a tool to predict antimicrobial resistance in Enterobacterales isolates causing invasive infections: an exploratory study across Cambodia, Kenya, and the UK[131]	2021	Human	Sample collection	3 countries	Global (HMIC)	2010-2017	Surveillance based on population colonisation metagenomics and taxonomy-adjusted AMR metrics presented here is a potentially valuable public health opportunity; This approach could theoretically be used to rapidly overcome the current paucity of quality AMR surveillance data and inform setting and population-tailored rationalization of empirical antibiotic use and treatment guidelines, develop measures to prevent and/or mitigate AMR, and ultimately improve public health decision-making in conjunction with relevant stakeholders, especially in LMICs
Practical Pharmacist-Led Interventions to Improve Antimicrobial Stewardship in Ghana, Tanzania, Uganda and Zambia[132]	2021	Human and Multisectoral	CwPAMS	4 countries	WHO AFR	2019-2020	Every country and hospital had a different focus for improvement based on local needs, from improving prescription developing guidelines and establishing new roles for pharmacists to a renewed focus on IPC and novel approaches to resolving local problems with local solution
Preliminary insights on carbapenem resistance in Enterobacteriaceae in high-income and low-/middle-income countries[133]	2024	Human, Environment and Multisectoral	PubMed; Google Scholar	46 countries	Global (HMIC)	2010-2020	The use of molecular methods, specifically whole genome sequencing, enables researchers to trace the sources of CRE and model the evolution and transmission of CRGs. However, the high costs of the method pose

							a limitation in comprehensive analyses of carbapenem resistance in HICs and LMICs
Preparedness to prescribe antibiotics responsibly: a comparison between final year medical students in France and Sweden[134]	2019	Human	Cross-sectional online survey	2 countries (France & Sweden)	EU (HIC)	2015	Disparities might reflect differences in national priorities, which translate into consistent emphases placed on certain topics at individual medical schools
Prevalence of antibiotic prescribing in COVID-19 patients in China and other low- and middle-income countries during the pandemic (December 2019-March 2021): A systematic review and meta-analysis[135]	2023	Human	Web of Science; EMBASE; PubMed; CNKI	19 countries	Global (LMIC)	2019-2021	Antibiotic prescribing rates in LMICs were generally high in hospitalized COVID-19 patients, regardless of severity of illness, in the first 15 months of the pandemic
Prevalence of antibiotic resistance in Helicobacter pylori: a systematic review and meta-analysis in World Health Organization regions[136]	2018	Human	PubMed	45 countries	Global	2007-2017	Worrisome levels of resistance rates in several areas of the world, suggesting that the development of resistance is associated with an increased risk of treatment failure.
Primary care treatment guidelines for skin infections in Europe: congruence with antimicrobial resistance found in commensal Staphylococcus aureus in the community[137]	2014	Human	200 nasal swabs from 20 primary care practices in each of the 9 countries; national primary-care guidelines for antibiotic use	9 countries	EU	2014	Evidence-based guidelines are an important step in controlling the development of AMR; not all European countries have developed national guidance for the treatment of common skin infections in primary care.
Quantitative and qualitative analysis of antimicrobial usage at farm and flock level on 181 broiler farms in nine European countries[138]	2019	Animal	Sample collection conducted by study authors	20 farms (21 in country A) in each of the nine participating countries: Belgium, Bulgaria, Denmark, France, Germany, Italy, Poland, Spain and the Netherlands	EU	2014-2016	Rearing broilers without AMU is feasible. However, a huge variation in AMU in terms of amount, moment of administration and antimicrobial classes was observed. This shows that there is still ground to be covered when it comes to AMU on broiler farms.
Rapid genomic characterization and global surveillance of Klebsiella using pathogenwatch[139]	2021	Multisectoral	NIHR GHRU; ENA	84 countries	Global	2021	Whole-genome sequencing empowers AMR surveillance laboratories to make public health decisions by providing a high-resolution view of the circulating bacterial strains and aiding

							outbreak investigations
Reported antibiotic use among patients in the multicenter ANDEMIA infectious diseases surveillance study in sub-Saharan Africa[140]	2024	Human	ANDEMIA	4 countries	WHO AFR	2018-2022	Relatively high levels of Watch group antibiotic use, particularly in acute febrile disease of unknown cause and for gastrointestinal infections, pose a challenge to antibiotic use interventions to address the burden of AMR
resistancebank.org, an open-access repository for surveys of antimicrobial resistance in animals[141]	2021	Animal	PubMed; Scopus; Web of Science; AGISAR	72 countries	Global (LMIC)	2000-2019	Areas identified as hotspots of resistance (P50>0.4) could be used to investigate the effects of stewardship campaigns, and alternatives to antimicrobials, such as vaccines and probiotics
Results from the Survey of Antibiotic Resistance (SOAR) 2014-16 in Bulgaria, Romania, Serbia and Croatia[142]	2018	Human	PubMed	45 countries	Global	2007-2017	Worrisome levels of resistance rates in several areas of the world, suggesting that the development of resistance is associated with an increased risk of treatment failure.
Review of antibiotic use and resistance in food animal production in WHO South-East Asia Region[143]	2014	Human	200 nasal swabs from 20 primary care practices in each of the 9 countries; national primary-care guidelines for antibiotic use	9 countries	EU	2014	Evidence-based guidelines are an important step in controlling the development of AMR; not all European countries have developed national guidance for the treatment of common skin infections in primary care.
Risk factors for the abundance of antimicrobial resistance genes aph(3')-III, erm(B), sul2 and tet(W) in pig and broiler faeces in nine European countries[144]	2019	Animal	Sample collection conducted by study authors	20 farms (21 in country A) in each of the nine participating countries: Belgium, Bulgaria, Denmark, France, Germany, Italy, Poland, Spain and the Netherlands	EU	2014-2016	Rearing broilers without AMU is feasible. However, a huge variation in AMU in terms of amount, moment of administration and antimicrobial classes was observed. This shows that there is still ground to be covered when it comes to AMU on broiler farms.
Sales of macrolides, lincosamides, streptogramins, and amoxicillin/clavulanate in the in- and outpatient setting in 10 European countries, 2007-2010[145]	2021	Multisectoral	NIHR GHRU; ENA	84 countries	Global	2021	Whole-genome sequencing empowers AMR surveillance laboratories to make public health decisions by providing a high-resolution view of the circulating bacterial strains and aiding outbreak investigations
Scoping review of national antimicrobial stewardship activities in	2024	Human	ANDEMIA	4 countries	WHO AFR	2018-2022	Relatively high levels of Watch group antibiotic use, particularly in acute febrile disease of

eight African countries and adaptable recommendations[146]							unknown cause and for gastrointestinal infections, pose a challenge to antibiotic use interventions to address the burden of AMR
Socioeconomic and Governance Factors Disentangle the Relationship between Temperature and Antimicrobial Resistance: A 10-Year Ecological Analysis of European Countries[147]	2023	Human, Environmental and Multisectoral	EARS-Net	30 countries	EEA/UK	2010-2019	Evidence of a positive linear association between temperature change and AMR proportion across all countries, years, pathogens, and antibiotics, adjusting for the effect of covariates.
SPiNCAR: A systematic model to evaluate and guide actions for tackling AMR[148]	2022	Multisectoral	Literature review	1 country (Italy)	EU (HIC)	2017-2020	Although drawn up on the basis of international evidence and recommendations, the framework was developed with great attention to the Italian specific reality and therefore represents a tailor-made tool; Nevertheless, the whole method could be adapted to other countries after a few necessary adjustments, cuts, and additions
Strengthening multisectoral coordination on antimicrobial resistance: a landscape analysis of efforts in 11 countries[149]	2021	Multisectoral	Desk review & scoping visit to each site	11 countries	WHO AFR/SEAR (Bangladesh)	2018-2019	The JEE tool and WHO benchmarks were highly useful guides to achieving key milestones in AMR containment —finalisation of NAPs-AMR and tools to guide their implementation strengthening the leadership, governance, and oversight capabilities of multisectoral governance structures, including establishment or revitalisation of IPC and AMS TWGs
Strengthening strategic management approaches to address antimicrobial resistance in global human health: a scoping review[150]	2019	Human, Animal and Multisectoral	Embase; Scopus; EconLit; HMIC; PsychInfo; IEEE; GARP	8 countries and global regions	Global	2000-2019	By using a consistent and comprehensive framework such as the PESTELI framework, important facilitators and inhibitors can be identified and leveraged
Strong correlation	2019	Human	EARS-Net	28 countries	EU	2016	strong correlation in bloodstream

between the rates of intrinsically antibiotic-resistant species and the rates of acquired resistance in Gram-negative species causing bacteraemia, EU/EEA, 2016[151]							infections between on the one hand the countries with most intrinsically resistant Gram-negative species, indicating the burden of intrinsic resistance, and on the other hand the percentage of acquired non-susceptibility in these species. This important information adds to the already well-established arguments for a strong reduction in the consumption of antibiotics, particularly those with broad-spectrum activity, which exert a selective pressure on all types of resistant bacteria. It also reinforces the crucial importance of measures to prevent host-to-host cross-transmission of antibiotic-resistant microorganisms, not only to control acquired resistance in every bacterial species but also to limit the burden of infections caused by species such as P. aeruginosa and Acinetobacter spp., in which intrinsic resistance per se represents a therapeutic problem.
Study on the design of a monitoring framework of the EU One Health Action Plans against AMR and Council Recommendation on stepping up EU actions to combat antimicrobial resistance in a One Health approach[152]	2025	Multisectoral	Indicators, consultations, interviews, and frameworks	29 countries	EU	2017-2023	Monitoring framework offering a foundation for tracking AMR efforts across EU, recommending periodical review, shared responsibility, visibility of results, and automation
Surveillance of antibiotic-resistant Escherichia coli in human populations through urban wastewater in ten European countries[153]	2020	Multisectoral	Sample collection conducted by study authors	10 countries	WHO EUR (HIC)	2016-2017	Significant relationships between proportion of resistant E. coli from wastewater samples and proportion of resistant E. coli from clinical samples; wastewater monitoring can predict clinical

							resistance levels
Surveillance of Antifungal Resistance in Candidemia Fails to Inform Antifungal Stewardship in European Countries[154]	2022	Multisectoral	EARS-Net; FWD-Net; AURES (AT); WIV-ISP(BE); ISKRA (HR); DANMAP (DK); NAKISe (EE); FIRE (FI); ONERBA (FR); ARS, SARI, GERMAP (DE); GSSAR (GR); NNSR (HU) Directorate of Health (IS); HPSC (IE); AR-ISS (IT); NVSPL (LT); ISIS-AR, MARAN, NETHMAP (NL); NORM, NORM-VET (NO); ARSIP (PT); CARMIN-ROM (RO); JIACARA (ES); SKUOPZ, NLZOH (SI); SWEDRES (SE); ANRESIS (CH); HPS, PHE, PHA (UK)	32 countries	EU/EFTA/UK	2005-2020	To facilitate comparisons at a global level, standardization of surveillance system reports, including disease definition and susceptibility patterns, reporting should be prioritized; These efforts would foster the interconnection between surveillance and AFS; Improving surveillance data in the field of fungal disease is of critical importance for the appropriate definition of burden, assessment of R&D priorities, and antifungal stewardship recommendations
Surveillance systems for healthcare-associated infection in high and upper-middle income countries: A scoping review[155]	2020	Human	PubMed; Google; online questionnaire	41 countries	Global (HIC; UMIC)	2017	Majority of the systems were operated on a voluntary basis and monitored disease incidence using CDC's definitions; MRSA, SSI, and CLABSI were commonly monitored; Comparability across countries and accessibility to the surveillance system should be subject to improvement
Surveillance systems to monitor antimicrobial resistance in Neisseria gonorrhoeae: a global, systematic review, 1 January 2012 to 27 September 2020[156]	2022	Human	Medline; PubMed; Global Health; EMBASE; CINAHL; Web of Science; ProQuest	47 countries	Global	2012-2020	Too few countries have surveillance systems, and too few systems are adequate
Susceptibility to antimicrobials of mastitis-causing Staphylococcus aureus, Streptococcus uberis and Str. dysgalactiae from New Zealand and the USA as assessed by the disk diffusion test[157]	2015	Human	5 diagnostic labs (NZ); Laboratory for Udder Health at the University of Minnesota, Veterinary Diagnostic Laboratory, Saint Paul (USA)	2 countries (NZ & USA)	Global (HIC)	2006-2007	Because of susceptibility to many available antimicrobials, routine use of later-generation cephalosporins and fluoroquinolones to treat mastitis caused by *S. aureus* and streptococci should be discouraged
Systematic review and survey of Neisseria gonorrhoeae ceftriaxone and azithromycin	2019	Human	WHO Centre for Sexually Transmitted Infections and Antimicrobial Resistance - GASP survey; literature; Gonococcal	24 countries and territories	WHO SEAR/WPR	2011-2016	The emergence of ceftriaxone and azithromycin resistance in N. gonnorrhoeae is a major global concern; the WHO 5% resistance

susceptibility data in the Asia Pacific, 2011 to 2016[158]			Antimicrobial Surveillance Programme				threshold of AMR has been breached in many sites in the Asia Pacific region
Systematic review of surveillance systems for AMR in Africa[159]	2022	Human and Multisectoral	PubMed; Scopus; Embase; AJOL; Cochrane	23 countries	WHO AFR	2018-2021	Although AMR surveillance has been implemented in 23 out of the 47 countries in the region, limitations exist in the surveillance methods and reporting protocols that can impair the usefulness, validity and trustworthiness of data.
Systematic Surveillance and Meta-Analysis of Antimicrobial Resistance and Food Sources from China and the USA[160]	2022	Multisectoral	literature (PubMed, Google Scholar, Web of Science)	2 countries (PRC, USA)	Global (HMIC)	2012-2021	Design and implementation of systematic phenotypic and genotypic surveillance of AMR in retail food is recommended
Targets for the reduction of antibiotic use in humans in the Transatlantic Taskforce on Antimicrobial Resistance (TATFAR) partner countries[161]	2019	Human	ECDC-developed questionnaire	9 countries	TATFAR partners	2017	Monitoring of countries' progress towards existing targets, possible barriers and facilitators, as well as the assessment of these countries' need to revise their targets, should provide additional key information and may be the objective of a future survey
The antimicrobial resistance travel tool, an interactive evidence-based educational tool to limit antimicrobial resistance spread[162]	2022	Human	COMBACTE-MAGNET EPI-Net; CDDEP; GLASS; EARS-Net; FWD-Net; Euro-GASP; various national surveillance systems	86 countries	Global	2014 (earliest)-2018 (latest)	From the perspective of the traveller, limiting travel-related spread can be effectively achieved by preventing colonisation through active advice from HCPs - the tool helps in this regard
The best laid plans?: international governance perspectives in AMR national action plans in Europe[163]	2023	Multisectoral	WHO library of NAPs	27 countries	EU	2023	Recognition of the challenge AMR poses for national and international governance strategies varies among NAPs in Europe, which might make coordinated action more difficult
The burden of antimicrobial resistance in the Americas in 2019: a cross-country systematic analysis[164]	2023	Human	GBD; mortality registries, surveillance systems, hospital systems, systematic literature reviews	34 countries	WHO AMR	2019	Countries facing limited access to antibiotics and basic healthcare services, or high ASMR rates of AMR, infection prevention the most cost-effective solution; for countries with widespread access to basic healthcare services, vaccination, antenatal care and antibiotic treatment, largest gains

							may be made through emphasis on antimicrobial stewardship
The burden of bacterial antimicrobial resistance in the WHO African Region in 2019: a cross-country systematic analysis[165]	2023	Human	GBD; research hospitals; surveillance networks; infection databases maintained by private labs and medical tech companies	47 countries	WHO AFR	2019	High infection rates are the primary driver of AMR mortality and morbidity, investment in vaccine development and distribution of critical importance, development of NAPs associated with decreases in AMR burden, but they are an expensive, multi-stakeholder undertaking
The European gonococcal antimicrobial surveillance programme (Euro-GASP) appropriately reflects the antimicrobial resistance situation for Neisseria gonorrhoeae in the European Union/European Economic Area[166]	2019	Human	Euro-GASP 2014; PubMed; Google Scholar, TESSy	15 countries	EEA	2009-2013	The prevalence of antimicrobial resistance reported by the Euro-GASP sentinel surveillance system appropriately reflects the AMR situation in the EEA
The European response to control and manage multi- and extensively drug-resistant Neisseria gonorrhoeae[167]	2022	Human	ECDC (Euro-GASP)	22 countries	Euro-GASP participants	2019	2019 ECDC response plan needs strong support from comprehensive management and control strategies nationally and internationally, including appropriate STI prevention (e.g. promotion of condom use), diagnostic and testing algorithms (e.g. triple-site testing in men who have sex with men), treatment, test of cure, notification and treatment of partners and robust epidemiological surveillance to identify key groups at risk of gonorrhoea and gonococcal AMR
The global burden of enteric fever, 2017-2021: a systematic analysis from the global burden of disease study 2021[168]	2024	Human	GBD 2021	75 countries	Global	2017-2021	The findings underscore the need for enhanced vaccination programmes, particularly the deployment of typhoid conjugate vaccines, which are effective in young children and provide long-term protection
The global governance of	2020	Multisectoral	WHO library of NAPs	59 countries	Global	2019	NAPs are more likely to align

antimicrobial resistance: a cross-country study of alignment between the global action plan and national action plans[169]							with the GAP in poorer member states, both when it comes to syntax and content
The impact of inpatient bloodstream infections caused by antibiotic-resistant bacteria in low- and middle-income countries: A systematic review and meta-analysis[170]	2023	Human	Systematic review and meta-analysis	18 countries	Global (LMIC)	1998 (earliest study) - 2022 (latest study)	Excess morbidity, mortality and costs associated with ARB BSIs are significant. Economic and disease burden in LMICs must be reduced through prevention, detection and treatment
The Impact of WHO Essential Medicines Policies on Inappropriate Use of Antibiotics[171]	2016	Human	WHO medicines use, policy databases; World Bank	55 countries	Global (LMIC)	2002-2008	Countries implementing the hypothesised policies have less inappropriate use of antibiotics; implementation of essential medicine policies is critical for LMIC
The strategic plan for combating antimicrobial resistance in Gulf Cooperation Council States[172]	2016	Human, Animal and Multisectoral	GCC-IC workshop	6 countries	Gulf Cooperation Council	2015	Networking has taken place to identify key players. They have acknowledged the importance of AMR and the immediate need for a national/regional strategic action plan; The plan is now at the stage of full adaptation at the highest national level and for countries to identify an accountable body to oversee it's implementation. This will take time to establish, allocate resources, and identify KPIs as well as to train the involved personnel
Towards understanding global patterns of antimicrobial use and resistance in neonatal sepsis: insights from the NeoAMR network[173]	2020	Human	Online survey of 39 NNUs	12 countries	Global	2017	Our data provide an important snapshot of how differences in NNU organisation and empirical antibiotic regimens may contribute to different clinical outcomes from AMR sepsis in different country settings
Tracking Candidemia Trends and Antifungal Resistance Patterns across Europe: An In-Depth Analysis of	2024	Human	EPI-Net	13 countries	EEA	2005-2024	The surveillance of C. glabrata and C. parapsilosis is intensifying globally, reflecting the rising incidence of candidemia caused by non-albicans species

Surveillance Systems and Surveillance Studies[174]							
Tracking global trends in the effectiveness of antibiotic therapy using the Drug Resistance Index[175]	2019	Human	IQVIA MIDAS	41 countries	Global	2000-2015	Here we used the DRI to demonstrate how the problems of resistance vary widely by geography, reflect underlying trends in antibiotic use and infer potential factors driving the differences among countries
Tracking progress on antimicrobial resistance by the quadripartite country self-assessment survey (TrACSS) in G7 countries, 2017-2023: opportunities and gaps[176]	2024	Multisectoral	TrACCS	7 countries	G7	2017-2023	Low implementation was described for the integration in AMR surveillance in the animal and food sectors, as well as training and education and good manufacturing and hygiene practices in the food sector, and optimising antimicrobial pesticide use
Trends in methicillin-resistant Staphylococcus aureus in the Gulf Cooperation Council countries: antibiotic resistance, virulence factors and emerging strains[177]	2022	Human	Literature (PubMed)	6 countries	Gulf Cooperation Council	2011-2021	Preventive guidelines needed to avoid a repeat of what caused the emergence of fusidic acid resistance; special attention to regional novel clones and newly adapted variants
Trends in the hospital-sector consumption of the WHO AWaRe Reserve group antibiotics in EU/EEA countries and the United Kingdom, 2010 to 2018[178]	2022	Human	EARS-Net; 2011-2012 ECDC PPS	23 countries	EEA/UK	2010-2018	Significantly increasing trends in the consumption of reserve antibiotics in most of countries raises concerns;
Trends of major antimicrobial resistance phenotypes in enterobacterales and gram-negative non-fermenters from ATLAS and EARS-net surveillance systems: Italian vs. European and global data, 2008-2018[179]	2021	Human	ATLAS; EARS-Net	76 countries	Global	2008-2018	A. baumannii had the highest resistance rates for carbapenems and DTR, amongst all resistance phenotypes, followed by 3GC resistance in K. pneumoniae in Italy; These resistance rates are higher than those observed in Europe and globally
Understanding antimicrobial resistance	2021	Multisectoral	Digital survey	15 countries	Global	2020-2021	Although both LMICs and HICs showed sufficient knowledge

from the perspective of public policy: a multinational knowledge, attitude, and perception survey to determine global awareness[180]							levels of AMR in this study, the perceptions and attitudes towards antimicrobial use are associated with the country of origin (i.e., LMIC or HIC)
Use of antimicrobials for animals in New Zealand, and in comparison with other countries[181]	2016	Animal	ESVAC; Department of Agriculture and industry levy-body databases (AUS); Land grant university websites (USA/CAN); Industry levy-body websites, publications and veterinarians (NZ)	30 countries	Global (HMIC)	2012	New Zealand was the third lowest user of antimicrobials in animal production and used much less than in human medicine
Use of the WHO Access, Watch, and Reserve classification to define patterns of hospital antibiotic use (AWaRe): an analysis of paediatric survey data from 56 countries[182]	2019	Human	GARPEC, Global-PPS	56 countries	Global	2015-2017	AWaRe classification could be used as a traffic light metric of appropriate antibiotic use; development and evaluation of paediatric antibiotic stewardship programmes on the basis for the AWaRe index are important future steps
Users' perception of the OH-EpiCap evaluation tool based on its application to nine national antimicrobial resistance surveillance systems[183]	2023	Multisectoral	SWOT analysis	N/A	N/A	2022	The tool provides a brief macro-overview of relevant OH topics, such as the perceived added value of establishing a OH team as a governance structure
Variability in the community consumption of antibiotics: a problem in Europe, Spain and Asturias[184]	2019	Human	ESAC-Net; Health Service of Asturias	30 countries & 1 region (Asturias)	EEA	2015	In those areas of greatest consumption, interventions should be implemented to establish criteria for a more rational use of antibiotics as well as to document the local prevalence of resistance
Variation in antibiotic prescription rates in febrile children presenting to emergency departments across Europe (MOFICHE): A multicentre observational study[185]	2020	Human	MOFICHE	8 countries	EU/UK (HIC)	2017-2018	Until better diagnostics are available to accurately differentiate between bacterial and viral aetiologies, implementation of antimicrobial stewardship guidelines across Europe is necessary to limit antimicrobial resistance
Veterinary Expert Opinion on Potential Drivers and Opportunities for	2018	Animal	Derived from questionnaire posited to participants (67 veterinarians - DK:18 PT:25 CH:24)	3 countries (Denmark, Portugal, Switzerland)	EU/EFTA (HIC)	2005-2012	Agreement that mandatory official interventions are very likely to positively impact AMU rates in livestock; improvement of on-

Changing Antimicrobial Usage Practices in Livestock in Denmark, Portugal, and Switzerland[186]							farm biosecurity, education on prevention measures for vets, early disease detection, rational AMU, vaccination and treatment plans all considered as important.
WGS to predict antibiotic MICs for Neisseria gonorrhoeae[187]	2017	Human	Sample collections	3 countries (US, UK & CAN)	Global (HIC)	1989-2014	If WGS becomes a widely used diagnostic tool, large amounts of surveillance data may become available from routine clinical activity. WGS may therefore provide reproducible and readily exchangeable data on the spread of antimicrobial resistance, alongside providing informative data on the strains that are carrying it and how resistance is being transmitted
While we are waiting for the Superbug: constitutional asymmetry and EU governmental policies to combat antimicrobial resistance[188]	2020	Multisectoral	ECDC; EFSA; EMA	31 countries	EEA/UK	2014	Constitutional asymmetry leads to differences in the way AMR initiatives are implemented through EU governmental instruments; We may expect AMR to rise quite high on the EU agenda, despite the weak embeddedness of health policy in the Treaty
WHO global antimicrobial resistance surveillance for Neisseria gonorrhoeae 2017-18: a retrospective observational study[189]	2021	Human	WHO GASP; WHO GLASS	73 countries	Global	2017-2018	The development of new antimicrobials to treat urogenital and extragenital gonorrhoea (in conjunction with strategies to conserve these novel and current antimicrobials), gonococcal vaccines, and detection tests for diagnosis and surveillance (including rapid point-of-care tests for detecting gonococci and AMR), is crucial for future disease control
Worldwide antibiotic resistance dynamics: how different is it from one drug-bug pair to another?[190]	2023	Human	ATLAS surveillance system	51 countries	Global	2019	Factors associated with ABR rates were different across drug-bug pairs but more similar within a bacterial species, reflecting different underlying ecological behaviours

Supplemental Table 3. Summary of grey literature.

Study	*Institution*	*Regional or National*	*Implemented or Proposed*	*Data Source Available*	*Units of Observation*	*Time Frame Examined*	*Ongoing*	*Setting*
8th Annual Report on Antimicrobial Agents Intended for Use in Animals (ANIMUSE)[191]	WOAH	National	Implemented	Yes	152 countries	2015-2024 (most recent data from 2021)	Yes	Global
Addressing gender inequalities in national action plans on AMR[192]	WHO	National	Proposed	NAPs yes, literature review no	N/A	2000-2024 (literature review)	N/A	Global
Antimicrobial consumption and resistance in bacteria from humans and food-producing animals[193]	ECDC, European Medicines Agency, EFSA	Regional	Implemented	No	Aggregate data from many European countries	2021	No	Europe
Antimicrobial resistance in the EU/EAA (EARS-Net): Annual Epidemiological Report for 2023[194]	ECDC	National	Implemented	Yes	30 countries	2023	Yes	Europe
Antimicrobial resistance: global report on surveillance[195]	WHO	Regional	Implemented	UNK	6 WHO Regions	2014	No	Global
Antimicrobial stewardship programmes in health-care facilities in LMICs: a WHO practical toolkit[196]	N/A	National	Proposed	No	N/A	N/A	N/A	Global
Assessment tool of the	National evaluation	National	Implemented	No	N/A	N/A	Yes	Global

minimum requirements for infection prevention and control programmes at the national level[197]	tool							
Averting the AMR Crisis: What are the Avenues for policy action for countries in Europe?[198]	Adapted from Cassini et al. 2019	National	Implemented	No	EU/EEA	2015	No	Global
Building the investment case for action against antimicrobial resistance (Annex to the GLG report)[199]	Calculated	National	Proposed	Yes	6 Region	2020-2050 projections	No	Global
CAESAR External Quality Assessment[200]	UKNEQAS	National	Implemented	Yes	11 countries	2020	No	Europe/Asia
Commitment to Development index (Health)[201]	PVS pathways	National	Implemented	Yes	40 countries	2003-2023	Yes	OECD, G20, BRICS, G7, MICs, EU
EU Council recommendation on stepping up EU actions to combat AMR in a One Health approach[202]	N/A	Regional	Proposed	No	EU	2023	Yes	EU
Guideline on the reporting of antimicrobial sales and use in animals at the EU level – denominators and indicators[203]	European Commission	National	Implemented	Yes	27 countries	2022	No	EU
Eurostat[204]	Eurostat	National	Implemented	Yes	27 countries	2013-present	Yes	EU
Evaluation of the Performance of Veterinary Services: PVS Tool (Terrestrial 2019)[205]	Country-measured	National	Proposed	No	N/A	N/A	N/A	Global
FAO-ATLASS "SET AMR" (Surveillance Evaluation Tool for AMR)[206]	Country-level	National	Proposed	No	N/A	N/A	No	Global

From reacting to preventing pandemics: building animal health and wildlife systems for One Health in East Asia and Pacific[207]	FAO, World Bank	National	Implemented	UNK	10 countries	2022	No	East Asia and Pacific
G7 Compliance Report on Antimicrobial Resistance, 2021-2023[208]	ARMoR and G7 Research Group	National	Implemented	Yes	G7 and EU	2021-2023	No	G7 and EU
GLASS-AMR[209]	WHO	National	Implemented	Yes	129 countries	5-year cycles since 2016	Yes	Global
Global Coalition on Aging AMR Preparedness Index Progress Report[210]	Global Coalition on Aging (through policy review, lit review, and expert interviews)	National	Implemented	Yes - but detailed indicators not publicly available	11 countries (Brazil, Canada, China, France, Germany, India, Italy, Japan, South Korea, the UK and the US)	2021, 2024	No	HIC
Global Indicator Framework for SDGs[211]	WHO	National	Implemented	Yes	200+ Countries	2016-2024	Yes	Global, with breakdowns by region or income
How can the EU support sustainable innovation and access to effective antibiotics?[212]	Adapted from BCG & Wellcome Trust, 2021	Regional	Implemented	UNK	5 regions	2021	No	Global
Implementation of WOAH standards: the observatory annual report 2022[213]	WOAH	National	Implemented	Yes	183 WOAH member countries	2022	Yes	Global
InFARM surveillance components and implementation questionnaire[214]	FAO	National	Proposed	No	N/A	N/A	N/A	Global
International Health Regulations States Parties Self-assessment annual	WHO	National	Implemented	Yes	195 countries	2010-2024	Yes	Global

reporting tool[215]								
IPCAT 2[216]	National evaluation tool - comparative scores do not seem to be compiled anywhere	National	Implemented	No	N/A	Published 2017	Yes	Global
Joint Monitoring Programme for Water Supply, Sanitation and Hygiene (JMP)[217]	WHO and UNICEF	National	Implemented	Yes	234 countries	1990 - Present	Yes	Global, with regional breakdowns
Methodology to analyse AMR-relevant legislation in the food and agriculture sector[218]	N/A	National	Proposed	No	N/A	N/A	No	Global
Minimizing risk of developing antibiotic resistance and aquatic ecotoxicity in the environment resulting from the manufacturing of human antibiotics[219]	AMRIA	N/A	Implemented	No	N/A	2022, updated in 2025	Yes	Global
Monitoring and evaluation for effective management of zoonotic diseases[220]	Individual countries	National	Proposed	No	N/A	Published 2024	Yes	Global
Monitoring and evaluation of the global action plan on antimicrobial resistance: framework and recommended indicators[221]	Global burden of disease (key bacterial infections plus HIV, TB and malaria)	National	Proposed	No	N/A	N/A	N/A	Global
Monitoring Framework for the WHO Strategic and Operational priorities to address drug-resistant bacterial infections[222]	WHO	National	Proposed	No	N/A	2025–2035	N/A	Global
Muscat Ministerial	N/A	National	Proposed	No	Endorsed by ~50	Proposed 2024	N/A	Global

Manifesto on AMR[223]					countries			
OECD Embracing a One Health Framework to Fight Antimicrobial Resistance[224]	OECD	National	Implemented	Yes	34 countries	2021-22	No	EU/EEA and OECD
One Health Trust Resistance Map[225]	IQVIA	National	Implemented	Yes	77 countries	2000-2023	Yes	Global
Operational approach to antimicrobial stewardship in the WHO Eastern Mediterranean Region[226]	WHO regional data on Member State Mechanism to address substandard and falsified medical products	National	Implemented	Yes	All countries	2024	Yes	Global
People-centred approach to addressing antimicrobial resistance in human health: WHO core package of interventions to support national action plans[227]	N/A	National	Proposed	No	N/A	N/A	N/A	Global
Point prevalence survey of healthcare-associated infections and antimicrobial use in European long-term care facilities[228]	ECDC	National	Implemented	Yes	LTCFs within 17 Countries	April - May 2013	UNK	Global
Priorities of the Global Leaders Group on AMR[229]	N/A	National	Proposed	No	N/A	N/A	N/A	Global
Surveillance of health care-associated infections at national and facility levels: practical handbook (WHO)[230]	WHO guide for national work	National	Proposed	No	N/A	N/A	Yes	Global
The costs and risks of AMR water pollution[231]	CCDEP	National	Implemented	No	Almost every country	2020	No	Global
Third joint inter-agency report on integrated analysis of consumption of	JIACRA III	National	Implemented	Yes	29 countries	2016-2018	No	Europe

antimicrobial agents and occurrence of antimicrobial resistance in bacteria from humans and food-producing animals in the EU/EEA (with disaggregation by antimicrobial class)[232]								
TrACSS 2023[233]	WHO	National	Implemented	Yes	177 Countries (with regional and income breakdowns)	2017 - 2023	Yes	Global, with breakdowns by region or income
UHC Watch[234]	WHO	National, regional	Implemented	Yes	56 countries/regions assessed	2000-2024	Yes	WHO EURO region
WHO benchmarks for strengthening health emergency capacities[235]	WHO	National	Implemented	Yes	199 countries	2023-Present	Yes	Global
WHO implementation handbook for NAPs on AMR: guidance for the human health sector (annex 7)[236]	N/A	National	Proposed	No	N/A	N/A	No	Global
WHO Joint External Evaluation - AMR[237]	WHO	National	Implemented	Yes	125 countries	2016 - 2024 (not annual)	Yes	Global
Worldwide country situation analysis: response to antimicrobial resistance[238]	WHO	Regional	Implemented	No	6 WHO regions	2015	No	Global

Supplemental Table 4. Summary of academic literature by setting (n = 183).

Setting	*Frequency (%)*
Europe	68 (37%)

Global (Income-level unspecified)	48 (26%)
African Region	21 (11%)
Global (HIC focused)	14 (8%)
South-East Asia/Western Pacific Region	12 (7%)
Global (LMIC focused)	12 (7%)
Region of the Americas	7 (4%)
Eastern Mediterranean Region	5 (3%)
Not Reported	1 (1%)

Table Notes: Income classifications (HIC/LMIC) reflect the scope as described by the original study authors and were not independently assigned. Totals exceed 100% due to multiple counting of entries spanning more than one category. HIC: High-income country; LMIC: Low-and middle-income country.

Supplemental Table 5. Summary of grey literature by setting (n = 49).

Setting	*Frequency (%)*
Global	36 (73%)
Europe	8 (16%)
Named Economic/Political Groupings[†]	4 (8%)
South-East Asia/Western Pacific Region	2 (4%)

Table Notes: [†]Named economic/political groupings (e.g. G7, OECD, HIC) refer to documents scoped to specific multi-country economic or political groupings that do not map directly onto WHO geographic regions. Some of these groupings span multiple continents and could be considered global in scope; they are presented separately here to reflect the heterogeneity of geographic descriptors used across the included literature. Totals exceed 100% due to multiple counting of entries spanning more than one

category.

Supplemental Figures

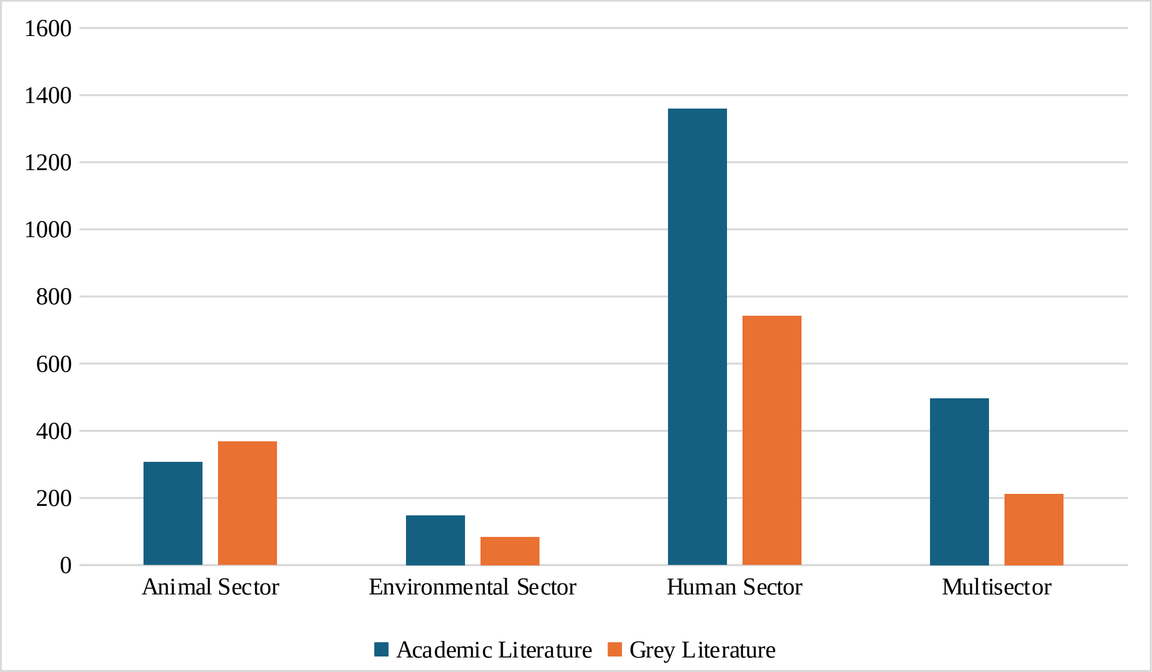

Supplemental Figure 1. Distribution of indicators by sector for academic and grey literature.

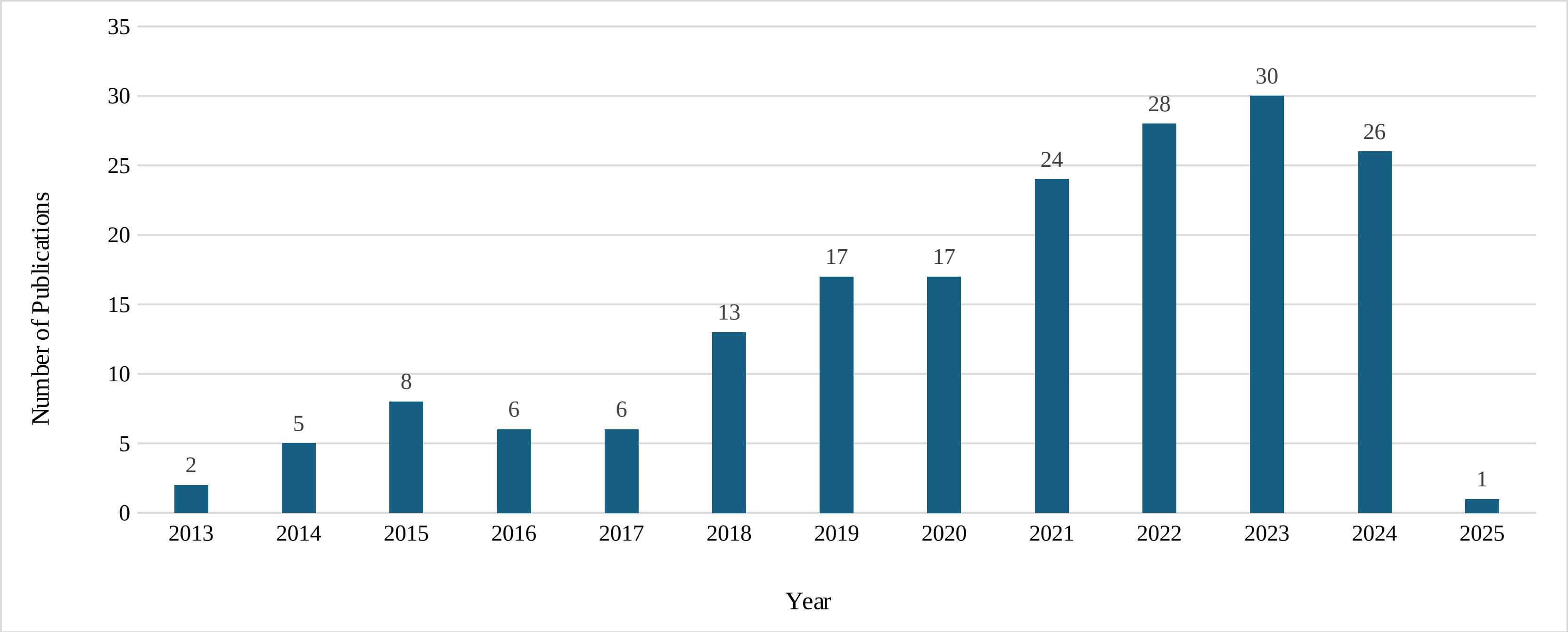

Supplemental Figure 2. Distribution of academic study publications by year.

Figure Notes: Although the primary search window spanned 2014–2024, one 2025 academic study identified during the grey-literature search met the inclusion criteria and was retained.

Preferred Reporting Items for Systematic reviews and Meta-Analyses extension for Scoping Reviews (PRISMA-ScR) Checklist

SECTION	ITEM	PRISMA-ScR CHECKLIST ITEM	REPORTED ON PAGE #
**TITLE**			
Title	1	Identify the report as a scoping review.	Page 1
**ABSTRACT**			
Structured summary	2	Provide a structured summary that includes (as applicable): background, objectives, eligibility criteria, sources of evidence, charting methods, results, and conclusions that relate to the review questions and objectives.	Lines 62 - 67
**INTRODUCTION**			
Rationale	3	Describe the rationale for the review in the context of what is already known. Explain why the review questions/objectives lend themselves to a scoping review approach.	Lines 65 - 67
Objectives	4	Provide an explicit statement of the questions and objectives being addressed with reference to their key elements (e.g., population or participants, concepts, and context) or other relevant key elements used to conceptualize the review questions and/or objectives.	Lines 62 - 64
**METHODS**			
Protocol and registration	5	Indicate whether a review protocol exists; state if and where it can be accessed (e.g., a Web address); and if available, provide registration information, including the registration number.	Appendix 1
Eligibility criteria	6	Specify characteristics of the sources of evidence used as eligibility criteria (e.g., years considered, language, and publication status), and provide a rationale.	Appendix 1
Information sources*	7	Describe all information sources in the search (e.g., databases with dates of coverage and contact with authors to identify additional sources), as well as the date the most recent search was executed.	Appendix 1
Search	8	Present the full electronic search strategy for at least 1 database, including any limits used, such that it could be repeated.	Appendix 1
Selection of sources of evidence†	9	State the process for selecting sources of evidence (i.e., screening and eligibility) included in the scoping review.	Appendix 1
Data charting process‡	10	Describe the methods of charting data from the included sources of evidence (e.g., calibrated forms or forms that have been tested by the team before their use, and	Appendix 1

SECTION	ITEM	PRISMA-ScR CHECKLIST ITEM	REPORTED ON PAGE #
		whether data charting was done independently or in duplicate) and any processes for obtaining and confirming data from investigators.	
Data items	11	List and define all variables for which data were sought and any assumptions and simplifications made.	Appendix 1
Critical appraisal of individual sources of evidence§	12	If done, provide a rationale for conducting a critical appraisal of included sources of evidence; describe the methods used and how this information was used in any data synthesis (if appropriate).	Appendix 1
Synthesis of results	13	Describe the methods of handling and summarizing the data that were charted.	Appendix 1
**RESULTS**			
Selection of sources of evidence	14	Give numbers of sources of evidence screened, assessed for eligibility, and included in the review, with reasons for exclusions at each stage, ideally using a flow diagram.	Lines 112 - 122
Characteristics of sources of evidence	15	For each source of evidence, present characteristics for which data were charted and provide the citations.	Supplemental Tables 1, 2, and 3
Critical appraisal within sources of evidence	16	If done, present data on critical appraisal of included sources of evidence (see item 12).	Supplemental Tables 2 and 3
Results of individual sources of evidence	17	For each included source of evidence, present the relevant data that were charted that relate to the review questions and objectives.	Supplemental Data 1 and 2
Synthesis of results	18	Summarize and/or present the charting results as they relate to the review questions and objectives.	Lines 671 - 673
**DISCUSSION**			
Summary of evidence	19	Summarize the main results (including an overview of concepts, themes, and types of evidence available), link to the review questions and objectives, and consider the relevance to key groups.	Lines 687 - 691
Limitations	20	Discuss the limitations of the scoping review process.	Lines 682 - 696
Conclusions	21	Provide a general pretation of the results with respect to the review questions and objectives, as well as potential implications and/or next steps.	Lines 736 - 742
**FUNDING**			
Funding	22	Describe sources of funding for the included sources of evidence, as well as sources of funding for the scoping review. Describe the role of the funders of the scoping review.	Lines 728 - 730

JBI = Joanna Briggs Institute; PRISMA-ScR = Preferred Reporting Items for Systematic reviews and Meta-Analyses extension for Scoping Reviews.
* Where *sources of evidence* (see second footnote) are compiled from, such as bibliographic databases, social media platforms, and Web sites.

† A more inclusive/heterogeneous term used to account for the different types of evidence or data sources (e.g., quantitative and/or qualitative research, expert opinion, and policy documents) that may be eligible in a scoping review as opposed to only studies. This is not to be confused with *information sources* (see first footnote).
‡ The frameworks by Arksey and O'Malley (6) and Levac and colleagues (7) and the JBI guidance (4, 5) refer to the process of data extraction in a scoping review as data charting.
§ The process of systematically examining research evidence to assess its validity, results, and relevance before using it to inform a decision. This term is used for items 12 and 19 instead of "risk of bias" (which is more applicable to systematic reviews of interventions) to include and acknowledge the various sources of evidence that may be used in a scoping review (e.g., quantitative and/or qualitative research, expert opinion, and policy document).

From: Tricco AC, Lillie E, Zarin W, O'Brien KK, Colquhoun H, Levac D, et al. PRISMA Extension for Scoping Reviews (PRISMAScR): Checklist and Explanation. Ann Intern Med. 2018;169:467–473. doi: 10.7326/M18-0850

References

1. Haddaway, N. R., Collins, A. M., Coughlin, D. & Kirk, S. The Role of Google Scholar in Evidence Reviews and Its Applicability to Grey Literature Searching. *PLOS ONE* **10**, e0138237 (2015).
2. Home | World Health Organization (WHO). *WHO- World Health Organization* https://www.who.int.
3. Home | World Organisation for Animal Health. *WOAH - World Organisation for Animal Health* https://www.woah.org/en/home/.
4. Food and Agriculture Organization of the United Nations. Home | Food and Agriculture Organization of the United Nations. *FAOHome* https://www.fao.org/home/en.
5. Environment, U. N. Home | UNEP - UN Environment Programme. *UNEP - UN Environment Programme* https://www.unep.org/node.
6. Home | The Organisation for Economic Co-operation and Development. *OECD* https://www.oecd.org/en.html.
7. Home | World Bank Group - International Development, Poverty and Sustainability. *World Bank Group* https://www.worldbank.org/ext/en/home.
8. Padget, M. *et al.* A community survey of antibiotic consumption among children in Madagascar and Senegal: the importance of healthcare access and care quality. *The Journal of antimicrobial chemotherapy* **72**, 564–573 (2017).
9. Farrell, S. *et al.* A multinational survey of companion animal veterinary clinicians: How can antimicrobial stewardship guidelines be optimised for the target stakeholder?. *Veterinary journal (London, England : 1997)* **303**, 106045 (2024).
10. Booth, A. & Wester, A. L. A multivariable analysis of the contribution of socioeconomic and environmental factors to blood culture Escherichia Coli resistant to fluoroquinolones in high- and middle-income countries. *BMC public health* **22**, 354 (2022).

11. Opatowski, L., Opatowski, M., Vong, S. & Temime, L. A One-Health Quantitative Model to Assess the Risk of Antibiotic Resistance Acquisition in Asian Populations: Impact of Exposure Through Food, Water, Livestock and Humans. *Risk analysis : an official publication of the Society for Risk Analysis* **41**, 1427–1446 (2021).

12. Caipo, M., Gatica, M. de L. A., Rojas, H. & Del Barrio, L. A qualitative approach for a situation analysis of AMR risks in the food animal production sector. *Frontiers in veterinary science* **10**, 1045276 (2023).

13. Willemsen, A., Reid, S. & Assefa, Y. A review of national action plans on antimicrobial resistance: strengths and weaknesses. *Antimicrobial resistance and infection control* **11**, 90 (2022).

14. Charani, E. *et al.* An analysis of existing national action plans for antimicrobial resistance-gaps and opportunities in strategies optimising antibiotic use in human populations. *The Lancet. Global health* **11**, e466–e474 (2023).

15. Chua, A. Q., Verma, M., Hsu, L. Y. & Legido-Quigley, H. An analysis of national action plans on antimicrobial resistance in Southeast Asia using a governance framework approach. *The Lancet regional health. Western Pacific* **7**, 100084 (2021).

16. Vogeler, C. S. & Parth, A.-M. An elephant in the room? Explaining agenda-setting in antimicrobial resistance policies in 30 European countries. *Social science & medicine (1982)* **356**, 117164 (2024).

17. Iwu, C. D. & Patrick, S. M. An insight into the implementation of the global action plan on antimicrobial resistance in the WHO African region: A roadmap for action. *International journal of antimicrobial agents* **58**, 106411 (2021).

18. Collignon, P., Beggs, J. J., Walsh, T. R., Sumanth Gandra, S. G. & Ramanan Laxminarayan, R. L. Anthropological and socioeconomic factors contributing to global antimicrobial resistance: a univariate and multivariable analysis. *Lancet Planetary Health* **2**, e398 (2018).

19. Zavaleta, E. *et al.* Antibiotic Consumption in Primary Care in Costa Rica and Italy: A Retrospective Cross-Country Analysis. *Cureus* **15**, e41414 (2023).
20. Fink, G., D'Acremont, V., Leslie, H. H. & Cohen, J. Antibiotic exposure among children younger than 5 years in low-income and middle-income countries: a cross-sectional study of nationally representative facility-based and household-based surveys. *Lancet Infectious Diseases* **20**, 179–187 (2020).
21. van Bijnen, E. M. E. *et al.* Antibiotic Exposure and Other Risk Factors for Antimicrobial Resistance in Nasal Commensal Staphylococcus aureus: An Ecological Study in 8 European Countries. *PloS one* **10**, e0135094 (2015).
22. Stedt, J. *et al.* Antibiotic resistance patterns in Escherichia coli from gulls in nine European countries. *Infection ecology & epidemiology* **4**, (2014).
23. Eckmann, C. *et al.* Antibiotic treatment patterns across Europe in patients with complicated skin and soft-tissue infections due to meticillin-resistant Staphylococcus aureus: a plea for implementation of early switch and early discharge criteria. *International Journal of Antimicrobial Agents* **44**, 56–64 (2014).
24. Andersson, K., van Driel, M., Hedin, K., Hollingworth, S. & Merlo, G. Antibiotic use in Australian and Swedish primary care: a cross-country comparison. *Scandinavian journal of primary health care* **40**, 95–103 (2022).
25. Robertson, J. *et al.* Antimicrobial medicines consumption in Eastern Europe and Central Asia - an updated cross-national study and assessment of quantitative metrics for policy action. *Frontiers in Pharmacology* **9**, (2019).
26. Kaiser, R. A., Taing, L. & Bhatia, H. Antimicrobial Resistance and Environmental Health: A Water Stewardship Framework for Global and National Action. *Antibiotics (Basel, Switzerland)* **11**, (2022).

27. Yang, D. *et al.* Antimicrobial resistance genes aph(3')-III, erm(B), sul2 and tet(W) abundance in animal faeces, meat, production environments and human faeces in Europe. *The Journal of antimicrobial chemotherapy* **77**, 1883–1893 (2022).

28. Chan, O. S. K. *et al.* Antimicrobial Resistance Policy Protagonists and Processes-A Qualitative Study of Policy Advocacy and Implementation. *Antibiotics (Basel, Switzerland)* **11**, (2022).

29. Elton, L. *et al.* Antimicrobial resistance preparedness in sub-Saharan African countries. *Antimicrobial resistance and infection control* **9**, 145 (2020).

30. Waterlow, N. R., Cooper, B. S., Robotham, J. V. & Knight, G. M. Antimicrobial resistance prevalence in bloodstream infection in 29 European countries by age and sex: An observational study. *PLoS medicine* **21**, e1004301 (2024).

31. Ceccarelli D. *et al.* Antimicrobial resistance prevalence in commensal Escherichia coli from broilers, fattening turkeys, fattening pigs and veal calves in European countries and association with antimicrobial usage at country level. *Journal of Medical Microbiology* **69**, 537EP – 547 (2020).

32. Kariuki, S., Kering, K., Wairimu, C., Onsare, R. & Mbae, C. Antimicrobial Resistance Rates and Surveillance in Sub-Saharan Africa: Where Are We Now?. *Infection and drug resistance* **15**, 3589–3609 (2022).

33. Gladstone, B. P. *et al.* Antimicrobial resistance rates in gram-positive bacteria do not drive glycopeptides use. *PloS one* **12**, e0181358 (2017).

34. Collignon, P., Athukorala, P.-C., Senanayake, S. & Khan, F. Antimicrobial resistance: the major contribution of poor governance and corruption to this growing problem. *PloS one* **10**, e0116746 (2015).

35. Veloo, A. C. M. *et al.* Antimicrobial susceptibility profiles of anaerobic bacteria, isolated from human clinical specimens, within different European and surrounding countries. A joint ESGAI study. *Anaerobe* **61**, 102111 (2020).

36. Tomic, T. *et al.* Antimicrobial utilization and resistance in Pseudomonas aeruginosa using segmented regression analysis: a comparative study between Serbia and eight European Countries. *International journal of clinical pharmacy* **45**, 989–998 (2023).

37. Horvat, O. *et al.* Are There Striking Differences in Outpatient Use of Antibiotics Between South Backa District, Serbia, and Some Scandinavian Countries?. *Frontiers in public health* **6**, 91 (2018).

38. Harant, A. Assessing transparency and accountability of national action plans on antimicrobial resistance in 15 African countries. *Antimicrobial resistance and infection control* **11**, 15 (2022).

39. Joosten, P. *et al.* Assigning Defined Daily/Course Doses for Antimicrobials in Turkeys to Enable a Cross-Country Quantification and Comparison of Antimicrobial Use. *Antibiotics (Basel, Switzerland)* **10**, (2021).

40. Serra-Burriel, M., Campillo-Artero, C., Agodi, A., Barchitta, M. & Lopez-Casasnovas, G. Association between antibiotic resistance in intensive care unit (ICU)-acquired infections and excess resource utilization: evidence from Spain, Italy, and Portugal. *Infection Control and Hospital Epidemiology* **43**, 1360–1367 (2022).

41. Munkholm, L., Rubin, O., Baekkeskov, E. & Humboldt-Dachroeden, S. Attention to the Tripartite's one health measures in national action plans on antimicrobial resistance. *Journal of public health policy* **42**, 236–248 (2021).

42. Bajalan, A. *et al.* Awareness regarding antimicrobial resistance and confidence to prescribe antibiotics in dentistry: a cross-continental student survey. *Antimicrobial resistance and infection control* **11**, 158 (2022).

43. Leong, M., Picton, R., Wratten, M., Mahe, A. & Zimmerman, P.-A. Baseline evaluation of the World Health Organization (WHO) infection prevention and control (IPC) core components in Pacific Island Countries and Territories (PICTs). *Antimicrobial resistance and infection control* **13**, 108 (2024).

44. Samuel F Orubu, E., Sutradhar, I., Zaman, M. H. & Wirtz, V. J. Benchmarking national action plans on antimicrobial resistance in eight selected LMICs: Focus on the veterinary sector strategies. *Journal of global health* **10**, 020414 (2020).

45. Holm, M. *et al.* Capturing data on antimicrobial resistance patterns and trends in use in regions of Asia (CAPTURA). *Clinical Infectious Diseases* **77**, S500–S506 (2023).

46. Dossouvi, K. M. & Ametepe, A. S. Carbapenem Resistance in Animal-Environment-Food from Africa: A Systematic Review, Recommendations and Perspectives. *Infection and drug resistance* **17**, 1699–1728 (2024).

47. Albiger, B., Glasner, C., Struelens, M. J., Grundmann, H. & Monnet, D. L. Carbapenemase-producing Enterobacteriaceae in Europe: assessment by national experts from 38 countries, May 2015. *Eurosurveillance* **20**, 21300 (2015).

48. Carrara, E. *et al.* Clinical management of severe infections caused by carbapenem-resistant gram-negative bacteria: a worldwide cross-sectional survey addressing the use of antibiotic combinations. *Clinical microbiology and infection : the official publication of the European Society of Clinical Microbiology and Infectious Diseases* **28**, 66–72 (2022).

49. Wang, M. *et al.* Clinical outcomes and bacterial characteristics of carbapenem-resistant Klebsiella pneumoniae complex among patients from different global regions (CRACKLE-2): a prospective, multicentre, cohort study. *Lancet Infectious Diseases* **22**, 401–412 (2022).

50. Suzuki, H. G., Dewez, J. E., Nijman, R. G. & Yeung, S. Clinical practice guidelines for acute otitis media in children: a systematic review and appraisal of European national guidelines. *BMJ open* **10**, e035343 (2020).

51. Carelli, D. E., Ogne, J. B. & Pierre, J. Coming of age: governance challenges in updated AMR national action plans in the EU. *European journal of public health* **34**, 885–889 (2024).

52. Scholle, O., Rasmussen, L., Reilev, M., Viebrock, J. & Haug, U. Comparative Analysis of Outpatient Antibiotic Prescribing in Early Life: A Population-Based Study Across Birth Cohorts in Denmark and Germany. *Infectious diseases and therapy* **13**, 299–312 (2024).

53. Caekebeke, N. *et al.* Comparing Farm Biosecurity and Antimicrobial Use in High-Antimicrobial-Consuming Broiler and Pig Farms in the Belgian-Dutch Border Region. *Frontiers in veterinary science* **7**, 558455 (2020).

54. Carmo, L. P. *et al.* Comparison of Antimicrobial Consumption Patterns in the Swiss and Danish Cattle and Swine Production (2007-2013). *Frontiers in veterinary science* **4**, 26 (2017).

55. Birgand, G. *et al.* Comparison of governance approaches for the control of antimicrobial resistance: Analysis of three European countries. *Antimicrobial resistance and infection control* **7**, 28 (2018).

56. Mizuno, S. *et al.* Comparison of national strategies to reduce meticillin-resistant Staphylococcus aureus infections in Japan and England. *The Journal of hospital infection* **100**, 280–298 (2018).

57. Chantziaras, I., Boyen, F., Callens, B. & Dewulf, J. Correlation between veterinary antimicrobial use and antimicrobial resistance in food-producing animals: a report on seven countries. *The Journal of antimicrobial chemotherapy* **69**, 827–34 (2014).

58. van Dorst, P. W. M. *et al.* Cost-Effectiveness of Test-and-Treat Strategies to Reduce the Antibiotic Prescription Rate for Acute Febrile Illness in Primary Healthcare Clinics in Africa. *Applied health economics and health policy* **22**, 701–715 (2024).

59. Rahbe, E., Watier, L., Guillemot, D., Glaser, P. & Opatowski, L. Determinants of worldwide antibiotic resistance dynamics across drug-bacterium pairs: a multivariable spatial-temporal analysis using ATLAS. *The Lancet. Planetary health* **7**, e547–e557 (2023).

60. Ferdinand, A. S. *et al.* Development of a cross-sectoral antimicrobial resistance capability assessment framework. *BMJ global health* **9**, (2024).

61. Hesstvedt, L. *et al.* Differences in epidemiology of candidaemia in the Nordic countries - what is to blame? *Mycoses* **60**, 11–19 (2017).

62. Sebastian, S. *et al.* Different microbial and resistance patterns in primary total knee arthroplasty infections - a report on 283 patients from Lithuania and Sweden. *BMC musculoskeletal disorders* **22**, 800 (2021).

63. Kenyon C.R., De Baetselier I., & Crucitti T. Does gonorrhoea screening intensity play a role in the early selection of antimicrobial resistance in men who have sex with men (MSM)? a comparative study of Belgium and the United Kingdom. *F1000Research* **7**, 569 (2018).

64. Poudyal, N. *et al.* Effective stakeholder engagement for collation, analysis and expansion of antimicrobial resistance (AMR) data: a CAPTURA experience. *Clinical Infectious Diseases* **77**, S519–S527 (2023).

65. Babu Rajendran, N. *et al.* EPI-Net One Health reporting guideline for antimicrobial consumption and resistance surveillance data: a Delphi approach. *The Lancet regional health. Europe* **26**, 100563 (2023).

66. Tebano, G. *et al.* Essential and forgotten antibiotics: an inventory in low- and middle-income countries. *International Journal of Antimicrobial Agents* **54**, 273–282 (2019).

67. Jackson, C. *et al.* Estimating global trends in total and childhood antibiotic consumption, 2011-2015. *BMJ global health* **4**, e001241 (2019).

68. Anonymous. Estimating the subnational prevalence of antimicrobial resistant Salmonella enterica serovars Typhi and Paratyphi A infections in 75 endemic countries, 1990-2019: a modelling study. *The Lancet. Global health* **12**, e406–e418 (2024).

69. Earnshaw, S. *et al.* European antibiotic awareness day: a five-year perspective of Europe-wide actions to promote prudent use of antibiotics. *Eurosurveillance* **19**, 20928 (2014).

70. Viprey, V. F. *et al.* European survey on the current surveillance practices, management guidelines, treatment pathways and heterogeneity of testing of Clostridioides difficile, 2018-2019: results from the Combatting Bacterial Resistance in Europe CDI (COMBACTE. *Journal of Hospital Infection* **131**, 213–220 (2023).

71. De Jong, A. *et al.* European-wide antimicrobial resistance monitoring in commensal Escherichia coli isolated from healthy food animals between 2004 and 2018. *The Journal of antimicrobial chemotherapy* **77**, 3301–3311 (2022).

72. Ardakani, Z. *et al.* Evaluating the contribution of antimicrobial use in farmed animals to global antimicrobial resistance in humans. *One Health* **17**, (2023).

73. Handley B.L. *et al.* Evaluating the yaws diagnostic gap: a survey to determine the capacity of and barriers to improving diagnostics in all yaws-endemic countries. *medRxiv* https://doi.org/10.1101/2022.05.30.22275669 (2022) doi:10.1101/2022.05.30.22275669.

74. Ogunnigbo, O. *et al.* Exploring the antimicrobial stewardship educational needs of healthcare students and the potential of an antimicrobial prescribing app as an educational tool in selected African countries. *Antibiotics* **11**, (2022).

75. Guzman-Blanco, M., Labarca, J. A., Villegas, M. V. & Gotuzzo, E. Extended spectrum beta -lactamase producers among nosocomial Enterobacteriaceae in Latin America. *Brazilian Journal of Infectious Diseases* **18**, 421–433 (2014).

76. Bedekelabou, A. P., Oyetola, D. W., Coulibaly, Z. L., Akinsola, O. & Bada-Alambedji, R. First assessment of the knowledge, attitudes, and practices of health actors in Togo and Ivory Coast in regard to antibiotic resistance. *International Journal of One Health* **8**, 108–123 (2022).

77. Freeman, J. *et al.* Five-year Pan-European, longitudinal surveillance of Clostridium difficile ribotype prevalence and antimicrobial resistance: the extended ClosER study. *European journal of clinical microbiology & infectious diseases : official publication of the European Society of Clinical Microbiology* **39**, 169–177 (2020).

78. Kakooza, F. *et al.* Genomic surveillance and antimicrobial resistance determinants in Neisseria gonorrhoeae isolates from Uganda, Malawi and South Africa, 2015-20. *The Journal of antimicrobial chemotherapy* **78**, 1982–1991 (2023).

79. Van Boeckel, T. P. *et al.* Global antibiotic consumption 2000 to 2010: an analysis of national pharmaceutical sales data. *The Lancet. Infectious diseases* **14**, 742–750 (2014).

80. Browne, A. J. *et al.* Global antibiotic consumption and usage in humans, 2000-18: a spatial modelling study. *The Lancet. Planetary health* **5**, e893–e904 (2021).

81. Tornimbene, B. *et al.* Global Antimicrobial Resistance and Use Surveillance System on the African continent: Early implementation 2017-2019. *African journal of laboratory medicine* **11**, 1594 (2022).

82. Zhou, N. *et al.* Global antimicrobial resistance: a system-wide comprehensive investigation using the Global One Health Index. *Infectious diseases of poverty* **11**, 92 (2022).

83. Weets, C. M. & Katz, R. Global approaches to tackling antimicrobial resistance: a comprehensive analysis of water, sanitation and hygiene policies. *BMJ global health* **9**, (2024).

84. Anonymous. Global burden of bacterial antimicrobial resistance 1990-2021: a systematic analysis with forecasts to 2050. *Lancet (London, England)* **404**, 1199–1226 (2024).

85. Carey, M. E. *et al.* Global diversity and antimicrobial resistance of typhoid fever pathogens: Insights from a meta-analysis of 13,000 Salmonella Typhi genomes. *eLife* **12**, (2023).

86. Balasubramanian, R., Van Boeckel, T. P., Carmeli, Y., Cosgrove, S. & Laxminarayan, R. Global incidence in hospital-associated infections resistant to antibiotics: An analysis of point prevalence surveys from 99 countries. *PLoS medicine* **20**, e1004178 (2023).

87. Luthman, O., Robb, D. H. F., Henriksson, P. J. G., Jorgensen, P. S. & Troell, M. Global overview of national regulations for antibiotic use in aquaculture production. *Aquaculture International* **32**, 9253–9270 (2024).

88. Mendelsohn, E. *et al.* Global patterns and correlates in the emergence of antimicrobial resistance in humans. *Proceedings. Biological sciences* **290**, 20231085 (2023).

89. Sweileh, W. M. Global research publications on irrational use of antimicrobials: call for more research to contain antimicrobial resistance. *Globalization and health* **17**, 94 (2021).

90. Hou, J. *et al.* Global trend of antimicrobial resistance in common bacterial pathogens in response to antibiotic consumption. *Journal of hazardous materials* **442**, 130042 (2023).

91. Schar, D., Klein, E. Y., Laxminarayan, R., Gilbert, M. & Van Boeckel, T. P. Global trends in antimicrobial use in aquaculture. *Scientific reports* **10**, 21878 (2020).

92. Mulchandani, R., Wang, Y., Gilbert, M. & Van Boeckel, T. P. Global trends in antimicrobial use in food-producing animals: 2020 to 2030. *PLOS global public health* **3**, e0001305 (2023).

93. Chiang, C.-H. *et al.* Healthcare-associated infections in intensive care units in Taiwan, South Korea, and Japan: recent trends based on national surveillance reports. *Antimicrobial resistance and infection control* **7**, 129 (2018).

94. Zhang, Q. *et al.* How far has the globe gone in achieving One Health? Current evidence and policy implications based on global One Health index. *Science in One Health* **3**, 100064 (2024).

95. Funiciello, E. *et al.* Identifying AWaRe indicators for appropriate antibiotic use: a narrative review. *Journal of Antimicrobial Chemotherapy* **79**, 3063–3077 (2024).

96. Kachalov V.N. *et al.* Identifying the drivers of multidrug-resistant Klebsiella pneumoniae at a European level. *PLoS Computational Biology* **17**, e1008446 (2021).

97. Karlowsky, J. A. *et al.* In Vitro Activity of Ceftazidime-Avibactam against Clinical Isolates of Enterobacteriaceae and Pseudomonas aeruginosa Collected in Asia-Pacific Countries: Results from the INFORM Global Surveillance Program, 2012 to 2015. *Antimicrobial agents and chemotherapy* **62**, (2018).

98. Karlowsky, J. A. *et al.* In vitro activity of ceftazidime-avibactam against clinical isolates of Enterobacteriaceae and Pseudomonas aeruginosa collected in Latin American countries: results from the INFORM global surveillance pro. *Antimicrobial Agents and Chemotherapy* **63**, e01814 (2019).

99. Karlowsky, J. A. *et al.* In vitro activity of ceftazidime-avibactam against enterobacterales and Pseudomonas aeruginosa isolates collected in Latin America as part of the atlas global surveillance program, 2017-2019. *Brazilian Journal of Infectious Diseases* **25**, (2021).

100. Karlowsky, J. A. *et al.* In vitro activity of imipenem/relebactam against non-MorganellaceaeEnterobacterales and Pseudomonas aeruginosa in Latin America: SMART 2018-2020. *Brazilian Journal of Infectious Diseases* **27**, (2023).

101. Karlowsky, J. A. *et al.* In vitro activity of imipenem/relebactam against piperacillin/tazobactam-resistant and meropenem-resistant non-Morganellaceae Enterobacterales and Pseudomonas aeruginosa collected from patients with lower. *JAC-Antimicrobial Resistance* **5**, (2023).

102. Nichols, W. W., de Jonge, B. L. M., Kazmierczak, K. M., Karlowsky, J. A. & Sahm, D. F. In Vitro Susceptibility of Global Surveillance Isolates of Pseudomonas aeruginosa to Ceftazidime-Avibactam (INFORM 2012 to 2014). *Antimicrobial agents and chemotherapy* **60**, 4743–9 (2016).

103. Rutz, J. *et al.* Individual and institutional predisposing factors of MRSA surgical site infection and outcomes-a retrospective case-control-study in 14 European high-volume surgical centres. *JAC-Antimicrobial Resistance* **6**, (2024).

104. Wang, S., Pulcini, C., Rabaud, C., Boivin, J.-M. & Birge, J. Inventory of antibiotic stewardship programs in general practice in France and abroad. *Medecine et maladies infectieuses* **45**, 111–23 (2015).

105. Rahbe E., Kovacevic A., Opatowski L., & Leclerc Q.J. Investigating the feasibility and potential of combining industry AMR monitoring systems: a comparison with WHO GLASS. *medRxiv* https://doi.org/10.1101/2024.03.27.24303768 (2024) doi:10.1101/2024.03.27.24303768.

106. Rubin, O. & Munkholm, L. Isomorphic dynamics in national action plans on antimicrobial resistance. *Public Administration and Development* **42**, 142–153 (2021).

107. Kinross, P. *et al.* Livestock-associated meticillin-resistant Staphylococcus aureus (MRSA) among human MRSA isolates, European Union/European Economic Area countries, 2013. *Eurosurveillance* **22**, 00696 (2017).

108. Babu Rajendran, N. *et al.* Mandatory surveillance and outbreaks reporting of the WHO priority pathogens for research & discovery of new antibiotics in European countries. *Clinical microbiology and infection : the official publication of the European Society of Clinical Microbiology and Infectious Diseases* **26**, 943.e1-943.e6 (2020).

109. Baede, V. O. *et al.* Markers of epidemiological success of methicillin-resistant Staphylococcus aureus isolates in European populations. *Clinical Microbiology and Infection* **29**, 1166–1173 (2023).

110. Rubinic, I., Leung, V. H., Hogberg, L. D., Monnet, D. L. & Vlahovic-Palcevski, V. Measuring hospital antibiotic consumption in EU/EEA countries: comparison of different metrics, 2017 to 2021. *Euro surveillance : bulletin Europeen sur les maladies transmissibles = European communicable disease bulletin* **29**, (2024).

111. Patel, J. *et al.* Measuring the global response to antimicrobial resistance, 2020-21: a systematic governance analysis of 114 countries. *The Lancet. Infectious diseases* **23**, 706–718 (2023).

112. Pontinen, A. K. *et al.* Modulation of multidrug-resistant clone success in Escherichia coli populations: a longitudinal, multi-country, genomic and antibiotic usage cohort study. *The Lancet. Microbe* **5**, e142–e150 (2024).

113. Mesa Varona, O. *et al.* Monitoring Antimicrobial Resistance and Drug Usage in the Human and Livestock Sector and Foodborne Antimicrobial Resistance in Six European Countries. *Infection and drug resistance* **13**, 957–993 (2020).

114. Gahimbare, L. *et al.* Monitoring progress on Antimicrobial Resistance (AMR) response in the World Health Organization African region: Insights from the Tracking AMR Country Self-Assessment Survey (TrACSS) 2021 results for the human health sector. *Journal of public health in Africa* **14**, 2392 (2023).

115. Joshi, M. P. *et al.* Moving from assessments to implementation: promising practices for strengthening multisectoral antimicrobial resistance containment capacity. *One health outlook* **5**, 7 (2023).

116. Joshi, M. P. *et al.* Multidisciplinary and multisectoral coalitions as catalysts for action against antimicrobial resistance: Implementation experiences at national and regional levels. *Global public health* **13**, 1781–1795 (2018).

117. Beeton, M. L. *et al.* Mycoplasma pneumoniae infections, 11 countries in Europe and Israel, 2011 to 2016. *Eurosurveillance* **25**, (2020).

118. Pallett, S. J. *et al.* National action plans for antimicrobial resistance and variations in surveillance data platforms. *Bulletin of the World Health Organization* **101**, 501-512F (2023).

119. Avello, P. *et al.* National action plans on antimicrobial resistance in Latin America: an analysis via a governance framework. *Health policy and planning* **39**, 188–197 (2024).

120. McDonnell, L. *et al.* National disparities in the relationship between antimicrobial resistance and antimicrobial consumption in Europe: an observational study in 29 countries. *The Journal of antimicrobial chemotherapy* **72**, 3199–3204 (2017).

121. Tegegne H.A. *et al.* OH-EpiCap: A semi-quantitative tool for the evaluation of One Health epidemiological surveillance capacities and capabilities. *medRxiv* https://doi.org/10.1101/2023.01.04.23284159 (2023) doi:10.1101/2023.01.04.23284159.

122. Postma, M. *et al.* Opinions of veterinarians on antimicrobial use in farm animals in Flanders and the Netherlands. *The Veterinary record* **179**, 68 (2016).

123. Alsan, M. *et al.* Out-of-pocket health expenditures and antimicrobial resistance in low-income and middle-income countries: an economic analysis. *The Lancet. Infectious diseases* **15**, 1203–1210 (2015).

124. Dyson, Z. A. *et al.* Pathogen diversity and antimicrobial resistance transmission of Salmonella enterica serovars Typhi and Paratyphi A in Bangladesh, Nepal, and Malawi: a genomic epidemiological study. *The Lancet. Microbe* **5**, 100841 (2024).

125. Outterson, K., Orubu, E. S. F., Rex, J., Ardal, C. & Zaman, M. H. Patient access in 14 high-income countries to new antibacterials approved by the US Food and Drug Administration, European medicines agency, Japanese pharmaceuticals and medical devices agency, or health Canada, 2010-2020. *Clinical Infectious Diseases* **74**, 1183–1190 (2021).

126. Thompson, W. *et al.* Patterns of dental antibiotic prescribing in 2017: Australia, England, United States, and British Columbia (Canada). *Infection control and hospital epidemiology* **43**, 191–198 (2022).

127. Visschers, V. H. M. *et al.* Perceptions of antimicrobial usage, antimicrobial resistance and policy measures to reduce antimicrobial usage in convenient samples of Belgian, French, German, Swedish and Swiss pig farmers. *Preventive veterinary medicine* **119**, 10–20 (2015).

128. Yahaya, A. A. *et al.* Perspectives on the Regional Strategy for Implementation of National Action Plans on Antimicrobial Resistance in the WHO African Region. *Antibiotics (Basel, Switzerland)* **13**, (2024).

129. Barkume, C. *et al.* Phase I of the Surveillance for Enteric Fever in Asia Project (SEAP): An Overview and Lessons Learned. *The Journal of infectious diseases* **218**, S188–S194 (2018).

130. Kinoshita, T., Tokumasu, H., Tanaka, S., Kramer, A. & Kawakami, K. Policy implementation for methicillin-resistant Staphylococcus aureus in seven European countries: a comparative analysis from 1999 to 2015. *Journal of market access & health policy* **5**, 1351293 (2017).

131. Tosasauguet, O. *et al.* Population-level faecal metagenomic profiling as a tool to predict antimicrobial resistance in Enterobacterales isolates causing invasive infections: an exploratory study across Cambodia, Kenya, and the UK. *EClinicalMedicine* **36**, (2021).

132. Kerr, F. *et al.* Practical Pharmacist-Led Interventions to Improve Antimicrobial Stewardship in Ghana, Tanzania, Uganda and Zambia. *Pharmacy (Basel, Switzerland)* **9**, (2021).

133. Stefaniak, K., Kiedrzynski, M., Korzeniewska, E., Kiedrzynska, E. & Harnisz, M. Preliminary insights on carbapenem resistance in Enterobacteriaceae in high-income and low-/middle-income countries. *The Science of the total environment* **957**, 177593 (2024).

134. Dyar, O. J., Lund, M., Lindsjo, C., Stalsby Lundborg, C. & Pulcini, C. Preparedness to prescribe antibiotics responsibly: a comparison between final year medical students in France and Sweden. *European journal of clinical microbiology & infectious diseases : official publication of the European Society of Clinical Microbiology* **38**, 711–717 (2019).

135. Cong W. *et al.* Prevalence of antibiotic prescribing in COVID-19 patients in China and other low- and middle-income countries during the pandemic (December 2019-March 2021): A systematic review and meta-analysis. *Journal of Antimicrobial Chemotherapy* **78**, 2787EP – 2794 (2023).

136. Savoldi, A., Carrara, E., Graham, D. Y., Conti, M. & Tacconelli, E. Prevalence of Antibiotic Resistance in Helicobacter pylori: A Systematic Review and Meta-analysis in World Health Organization Regions. *Gastroenterology* **155**, 1372-1382.e17 (2018).

137. Bijnen, E. M. van *et al.* Primary care treatment guidelines for skin infections in Europe: congruence with antimicrobial resistance found in commensal Staphylococcus aureus in the community. *BMC Family Practice* **15**, (2014).

138. Joosten, P. *et al.* Quantitative and qualitative analysis of antimicrobial usage at farm and flock level on 181 broiler farms in nine European countries. *The Journal of antimicrobial chemotherapy* **74**, 798–806 (2019).

139. Argimon, S. *et al.* Rapid genomic characterization and global surveillance of Klebsiella using pathogenwatch. *Clinical Infectious Diseases* **73**, S325–S335 (2021).

140. Wieters, I. *et al.* Reported antibiotic use among patients in the multicenter ANDEMIA infectious diseases surveillance study in sub-saharan Africa. *Antimicrobial resistance and infection control* **13**, 9 (2024).

141. Criscuolo, N. G., Pires, J., Zhao, C. & Van Boeckel, T. P. resistancebank.org, an open-access repository for surveys of antimicrobial resistance in animals. *Scientific data* **8**, 189 (2021).

142. Torumkuney, D. *et al.* Results from the Survey of Antibiotic Resistance (SOAR) 2014-16 in Bulgaria, Romania, Serbia and Croatia. *The Journal of antimicrobial chemotherapy* **73**, v2–v13 (2018).

143. Malik, H. *et al.* Review of antibiotic use and resistance in food animal production in WHO South-East Asia Region. *Journal of infection and public health* **16 Suppl 1**, 172–182 (2023).

144. Yang, D. *et al.* Risk factors for the abundance of antimicrobial resistance genes aph(3')-III, erm(B), sul2 and tet(W) in pig and broiler faeces in nine European countries. *The Journal of antimicrobial chemotherapy* **77**, 969–978 (2022).

145. Ferrer, P. *et al.* Sales of macrolides, lincosamides, streptogramins, and amoxicillin/clavulanate in the in- and outpatient setting in 10 European countries, 2007-2010. *SpringerPlus* **4**, 612 (2015).

146. Kamere, N. *et al.* Scoping review of national antimicrobial stewardship activities in eight African countries and adaptable recommendations. *Antibiotics* **11**, (2022).

147. Maugeri, A., Barchitta, M., Magnano San Lio, R. & Agodi, A. Socioeconomic and Governance Factors Disentangle the Relationship between Temperature and Antimicrobial Resistance: A 10-Year Ecological Analysis of European Countries. *Antibiotics (Basel, Switzerland)* **12**, (2023).

148. Bravo, G. *et al.* SPiNCAR: A systematic model to evaluate and guide actions for tackling AMR. *PloS one* **17**, e0265010 (2022).

149. Joshi, M. P. *et al.* Strengthening multisectoral coordination on antimicrobial resistance: a landscape analysis of efforts in 11 countries. *Journal of pharmaceutical policy and practice* **14**, 27 (2021).

150. Ahmad, R., Zhu, N. J., Leather, A. J. M., Holmes, A. & Ferlie, E. Strengthening strategic management approaches to address antimicrobial resistance in global human health: a scoping review. *BMJ global health* **4**, e001730 (2019).

151. Jarlier, V. *et al.* Strong correlation between the rates of intrinsically antibiotic-resistant species and the rates of acquired resistance in Gram-negative species causing bacteraemia, EU/EEA, 2016. *Eurosurveillance* **24**, 1800538 (2019).

152. European Health and Digital Executive Agency, Intellera Consulting & Tetra Tech International Development. *Study on the Design of a Monitoring Framework of the EU One Health Action Plans against AMR and Council Recommendation on Stepping up EU Actions to Combat Antimicrobial Resistance in a One Health Approach*. https://op.europa.eu/en/publication-detail/-/publication/e872ae01-e50d-11ef-bc1c-01aa75ed71a1.

153. Huijbers, P. M. C., Larsson, D. G. J. & Flach, C.-F. Surveillance of antibiotic resistant Escherichia coli in human populations through urban wastewater in ten European countries. *Environmental pollution (Barking, Essex : 1987)* **261**, 114200 (2020).

154. Galia, L. *et al.* Surveillance of Antifungal Resistance in Candidemia Fails to Inform Antifungal Stewardship in European Countries. *Journal of fungi (Basel, Switzerland)* **8**, (2022).

155. Takaya, S. *et al.* Surveillance systems for healthcare-associated infection in high and upper-middle income countries: A scoping review. *Journal of infection and chemotherapy : official journal of the Japan Society of Chemotherapy* **26**, 429–437 (2020).

156. Medland N.A. *et al.* Surveillance systems to monitor antimicrobial resistance in Neisseria gonorrhoeae: a global, systematic review, 1 January 2012 to 27 September 2020. *Eurosurveillance* **27**, 2100917 (2022).

157. Petrovski, K. R. *et al.* Susceptibility to antimicrobials of mastitis-causing Staphylococcus aureus, Streptococcus uberis and Str. dysgalactiae from New Zealand and the USA as assessed by the disk diffusion test. *Australian veterinary journal* **93**, 227–33 (2015).

158. George, C. R. R. *et al.* Systematic review and survey of Neisseria gonorrhoeae ceftriaxone and azithromycin susceptibility data in the Asia Pacific, 2011 to 2016. *PloS one* **14**, e0213312 (2019).

159. Okolie O.J., Igwe U., Ismail S.U., Ighodalo U.L., & Adukwu E.C. Systematic review of surveillance systems for AMR in Africa. *Journal of Antimicrobial Chemotherapy* **78**, 31EP – 51 (2023).

160. Himanshu *et al.* Systematic Surveillance and Meta-Analysis of Antimicrobial Resistance and Food Sources from China and the USA. *Antibiotics* **11**, 1471 (2022).

161. D'Atri, F. *et al.* Targets for the reduction of antibiotic use in humans in the Transatlantic Taskforce on Antimicrobial Resistance (TATFAR) partner countries. *Eurosurveillance* **24**, 1800339 (2019).

162. Arieti, F. *et al.* The antimicrobial resistance travel tool, an interactive evidence-based educational tool to limit antimicrobial resistance spread. *Journal of travel medicine* **29**, (2022).

163. Carelli, D. E., Mitsouli, E. T., Ogne, J. B. & Pierre, J. The best laid plans?: international governance perspectives in AMR national action plans in Europe. *European journal of public health* **33**, 682–686 (2023).

164. Anonymous. The burden of antimicrobial resistance in the Americas in 2019: a cross-country systematic analysis. *Lancet regional health. Americas* **25**, 100561 (2023).

165. Anonymous. The burden of bacterial antimicrobial resistance in the WHO African region in 2019: a cross-country systematic analysis. *The Lancet. Global health* **12**, e201–e216 (2024).

166. Cole, M. J. *et al.* The European gonococcal antimicrobial surveillance programme (Euro-GASP) appropriately reflects the antimicrobial resistance situation for Neisseria gonorrhoeae in the European Union/European Economic Area. *BMC infectious diseases* **19**, 1040 (2019).

167. Cole, M. J. *et al.* The European response to control and manage multi- and extensively drug-resistant Neisseria gonorrhoeae. *Eurosurveillance* **27**, (2022).

168. Piovani, D., Figlioli, G., Nikolopoulos, G. K. & Bonovas, S. The global burden of enteric fever, 2017-2021: a systematic analysis from the global burden of disease study 2021. *EClinicalMedicine* **77**, 102883 (2024).

169. Munkholm, L. & Rubin, O. The global governance of antimicrobial resistance: a cross-country study of alignment between the global action plan and national action plans. *Globalization and health* **16**, 109 (2020).

170. Allel, K. *et al.* The impact of inpatient bloodstream infections caused by antibiotic-resistant bacteria in low- and middle-income countries: A systematic review and meta-analysis. *PLoS medicine* **20**, e1004199 (2023).

171. Holloway, K. A., Rosella, L. & Henry, D. The Impact of WHO Essential Medicines Policies on Inappropriate Use of Antibiotics. *PloS one* **11**, e0152020 (2016).

172. Balkhy, H. H. *et al.* The strategic plan for combating antimicrobial resistance in Gulf Cooperation Council States. *Journal of infection and public health* **9**, 375–85 (2016).

173. Li, G. *et al.* Towards understanding global patterns of antimicrobial use and resistance in neonatal sepsis: insights from the NeoAMR network. *Archives of disease in childhood* **105**, 26–31 (2020).

174. Odoj, K. *et al.* Tracking Candidemia Trends and Antifungal Resistance Patterns across Europe: An In-Depth Analysis of Surveillance Systems and Surveillance Studies. *Journal of fungi (Basel, Switzerland)* **10**, (2024).

175. Klein, E. Y., Tseng, K. K., Pant, S. & Laxminarayan, R. Tracking global trends in the effectiveness of antibiotic therapy using the Drug Resistance Index. *BMJ global health* **4**, e001315 (2019).

176. Sabbatucci, M. *et al.* Tracking progress on antimicrobial resistance by the quadripartite country self-assessment survey (TrACSS) in G7 countries, 2017-2023: opportunities and gaps. *Pharmacological research* **204**, 107188 (2024).

177. Al-Saleh, A., Shahid, M., Farid, E. & Bindayna, K. Trends in methicillin-resistant Staphylococcus aureus in the Gulf Cooperation Council countries: antibiotic resistance, virulence factors and emerging strains. *Eastern Mediterranean Health Journal* **28**, 434–443 (2022).

178. Benko, R. *et al.* Trends in the hospital-sector consumption of the WHO AWaRe Reserve group antibiotics in EU/EEA countries and the United Kingdom, 2010 to 2018. *Eurosurveillance* **27**, (2022).

179. Rossolini, G. M., Bochenska, M., Fumagalli, L. & Dowzicky, M. Trends of major antimicrobial resistance phenotypes in enterobacterales and gram-negative non-fermenters from ATLAS and EARS-net surveillance systems: Italian vs. European and global data, 2008-2018. *Diagnostic microbiology and infectious disease* **101**, 115512 (2021).

180. Naing, S., van Wijk, M., Vila, J. & Balleste-Delpierre, C. Understanding antimicrobial resistance from the perspective of public policy: a multinational knowledge, attitude, and perception survey to determine global awareness. *Antibiotics* **10**, (2021).

181. Hillerton, J. E., Irvine, C. R., Bryan, M. A., Scott, D. & Merchant, S. C. Use of antimicrobials for animals in New Zealand, and in comparison with other countries. *New Zealand veterinary journal* **65**, 71–77 (2017).

182. Hsia, Y. *et al.* Use of the WHO Access, Watch, and Reserve classification to define patterns of hospital antibiotic use (AWaRe): an analysis of paediatric survey data from 56 countries. *The Lancet. Global health* **7**, e861–e871 (2019).

183. Moura P. *et al.* Users' perception of the OH-EpiCap evaluation tool based on its application to nine national antimicrobial resistance surveillance systems. *medRxiv* https://doi.org/10.1101/2023.03.15.23287323 (2023) doi:10.1101/2023.03.15.23287323.

184. Sanchez, M. L. *et al.* Variability in the community consumption of antibiotics: a problem in Europe, Spain and Asturias. *Le infezioni in medicina* **27**, 134–140 (2019).

185. Hagedoorn N.N. *et al.* Variation in antibiotic prescription rates in febrile children presenting to emergency departments across Europe (MOFICHE): A multicentre observational study. *PLoS Medicine* **17**, e1003208 (2020).

186. Carmo, L. P. *et al.* Veterinary Expert Opinion on Potential Drivers and Opportunities for Changing Antimicrobial Usage Practices in Livestock in Denmark, Portugal, and Switzerland. *Frontiers in veterinary science* **5**, 29 (2018).

187. Eyre, D. W. *et al.* WGS to predict antibiotic MICs for Neisseria gonorrhoeae. *The Journal of antimicrobial chemotherapy* **72**, 1937–1947 (2017).

188. Jensen, C. S. While we are waiting for the Superbug: constitutional asymmetry and EU governmental policies to combat antimicrobial resistance. *Journal of Common Market Studies* **58**, 1361–1376 (2020).

189. Unemo, M. *et al.* WHO global antimicrobial resistance surveillance for Neisseria gonorrhoeae 2017-18: a retrospective observational study. *The Lancet. Microbe* **2**, e627–e636 (2021).

190. Rahbe E., Watier L., Guillemot D., Glaser P., & Opatowski L. Worldwide antibiotic resistance dynamics: how different is it from one drug-bug pair to another? *medRxiv* https://doi.org/10.1101/2022.02.09.22270726 (2022) doi:10.1101/2022.02.09.22270726.

191. World Organisation for Animal Health. *Annual Report on Antimicrobial Agents Intended for Use in Animals*. https://www.woah.org/app/uploads/2024/05/woah-amu-report-2024-final.pdf (2024).

192. World Health Organization. Addressing gender inequalities in national action plans on antimicrobial resistance - Guidance to complement the people-centred approach. (2024).

193. European Food Safety Authority. Antimicrobial consumption and resistance in bacteria from humans and food-producing animals. (2024).

194. European Centre for Disease Prevention and Control. Antimicrobial resistance in the EU/EEA (EARS-Net) - Annual Epidemiological Report 2023. (2024).

195. World Health Organization. Antimicrobial resistance: global report on surveillance. (2014).

196. World Health Organization. Antimicrobial stewardship programmes in health-care facilities in low- and middle-income countries: a WHO practical toolkit. (2019).

197. World Health Organization. Assessment tool of the minimum requirements for infection prevention and control programmes at the national level. (2021).

198. Anderson, M. *et al.* Averting the AMR crisis - What are the avenues for policy action for countries in Europe? (2019).

199. Food and Agriculture Organization of the United Nations *et al.* Annex to the GLG Report: Towards specific commitments and action in response to antimicrobial resistance. (2024).

200. World Health Organization. Central Asian and European Surveillance of Antimicrobial Resistance - External quality assessment results 2020. (2022).

201. Center for Global Development. The Commitment to Development Index. (2023).

202. The Council of the European Union. Council Recommendation on stepping up EU actions to combat antimicrobial resistance in a One Health approach. (2023).

203. European Medicines Agency Science Medicines Health. Guideline on the reporting of antimicrobial sales and use in animals at the EU level – denominators and indicators. (2023).

204. Eurostat. Consumption of antibiotics in the community and hospital sectors - defined daily doses (DDD) per day. (2025).

205. World Organisation for Animal Health. Evaluation of the Performance of Veterinary Services PVS Tool - Terrestrial 2019. (2023).

206. Latornico, F., Keck, N., Treilles, M. & Kabali, E. FAO Assessment Tool for Laboratories and AMR Surveillance Systems (ATLASS) for the food and agriculture sectors. (2018).

207. World Bank & Food and Agriculture Organization of the United Nations. From reacting to preventing pandemics: Building Animal Health and Wildlife Systems for One Health in East Asia and Pacific. (2022).

208. Alliance for Reducing Microbial Resistance & G7 Research Group. G7 Compliance Report on Antimicrobial Resistance, 2021-2023. (2024).

209. World Health Organization. Global Antimicrobial Resistance and Use Surveillance System (GLASS). (2023).

210. Global Coalition on Aging & Infectious Diseases Society of America. 2024 AMR Preparedness Index Progress Report. (2024).

211. United Nations Department of Economic and Social Affiars. Global indicator framework for the Sustainable Development Goals and targets of the 2030 Agenda for Sustainable Development. (2024).

212. Anderson, M., Panteli, D. & Mossialos, E. How can the EU support sustainable innovation and access to effective antibiotics? (2023).

213. World Organisation for Animal Health. *Implementation of WOAH Standards: The Observatory Annual Report*. https://www.woah.org/en/what-we-do/standards/observatory/implementation-of-standards-the-observatory-monitoring-report/ (2022).

214. Food and Agriculture Organization of the United Nations. The International FAO Antimicrobial Resistance Monitoring (InFARM) system: Manual for implementation - Annex 3. (2024).

215. World Health Organization. IHR (2005) States Parties self-assessment annual reporting tool, 2nd ed. (2021).

216. World Health Organization. Instructions for the national infection prevention and control assesment tool 2 (IPCAT 2). (2017).

217. World Health Organization & United Nations International Children's Emergency Fund. WHO/UNICEF Joint Monitoring Programme for Water Supply, Sanitation and Hygiene (JMP). (2023).
218. Food and Agriculture Organization of the United Nations. Methodology to Analyse AMR-Relevant Legislation in the Food and Agriculture Sector - Guidance Document for Regulators. (2020).
219. AMR Industry Alliance. Minimizing risk of developing antibiotic resistance and aquatic ecotoxicity in the environment resulting from the manufacturing of human antibiotics. (2022).
220. Food and Agriculture Organization of the United Nations, World Health Organization & World Organisation for Animal Health. Monitoring and Evaluation for Effective Management of Zoonotic Diseases - An operational tool of the Tripartite Zoonoses Guide. (2024).
221. World Health Organization, World Organisation for Animal Health & Food and Agriculture Organization of the United Nations. Monitoring and evaluation of the global action plan on antimicrobial resistance. (2019).
222. World Health Organization. Monitoring framework for the WHO Strategic and operational priorities to address drug-resistant bacterial infections in the human health sector, 2025–2035. (2024).
223. Sultanate of Oman Ministry of Health & Sultanate of Oman Ministry of Agriculture, Fisheries Wealth and Water Resources. Report of the Third High-Level Ministerial Conference on Antimicrobial Resistance. (2022).
224. Organisation for Economic Co-operation and Development Health Policy Studies. Embracing a One Health Framework to Fight Antimicrobial Resistance. (2023).
225. One Health Trust. ResistanceMap. (2025).

226. World Health Organization Eastern Mediterranean Region. Operational approach to antimicrobial stewardship in the WHO Eastern Mediterranean Region. (2024).
227. World Health Organization. People-centred approach to addressing antimicrobial resistance in human health: WHO core package of interventions to support national action plans. (2023).
228. European Centre for Disease Prevention and Control *et al.* Point prevalence survey of healthcare-associated infections and antimicrobial use in European long-term care facilities. (2014).
229. Global Leaders Group on Antimicrobial Resistance. Priorities of the Global Leaders Group on AMR. (2023).
230. World Health Organization. Surveillance of health care-associated infections at national and facility levels - Practical handbook. (2024).
231. Vivideconomics. The costs and risks of AMR water pollution. (2020).
232. European Centre for Disease Prevention and Control, European Food Safety Authority & European Medicines Agency. Third joint inter-agency report on integrated analysis of consumption of antimicrobial agents and occurrence of antimicrobial resistance in bacteria from humans and food-producing animals in the EU/EEA. (2021).
233. World Health Organization. Tracking AMR Country Self-assessment Survey - TrACSS. (2025).
234. World Health Organization Barcelona Office for Health Systems Financing. UHC Watch. (2024).
235. World Health Organization. WHO benchmarks for strengthening health emergency capacities. (2023).
236. World Health Organization. WHO implementation handbook for national action plans on antimicrobial resistance: guidance for the human health sector. (2022).
237. World Health Organization. Joint External Evaluation Tool: International Health Regulations (2005), third edition. (2022).

238. World Health Organization. Worldwide country situation analysis: response to antimicrobial resistance. (2015).

Supplemental Data 1 & 2: Summary of Academic and Grey Literature Indicators

Supplemental Data 1. Summary of Academic Literature Indicators

	Study	*Indicator*	*Measurement*	*One Health Sector*	*Subdomain*
1	A community survey of antibiotic consumption among children in Madagascar and Senegal: the importance of healthcare access and care quality	Antibiotic consumption (%) per public health structures and pharmacies (/10,000 pop)	quantitative	Human	Stewardship
2	A multinational survey of companion animal veterinary clinicians: How can antimicrobial stewardship guidelines be optimised for the target stakeholder?	Country-level median awareness and understanding scores for antimicrobial stewardship guidelines	quantitative	Multisector	Community Awareness & Enabling Behaviours
3	A multivariable analysis of the contribution of socioeconomic and environmental factors to blood culture Escherichia Coli resistant to fluoroquinolones in high- and middle-income countries	DDD/1000/y of fluoroquinolones consumed	quantitative	Human	Stewardship
		DDD/1000/y of all antibiotics consumed	quantitative	Human	Stewardship
		GNI US$	quantitative	Multisector	Social Determinants
		% of E. coli that is fluoroquinolone resistant	quantitative	Multisector	Surveillance & Laboratory
		Education level in years	quantitative	Multisector	Social Determinants
		Average annual °C	quantitative	Multisector	Social Determinants
		% of a country's pop using unsafely managed sanitation	quantitative	Environment	Prevention & Control/ Social Determinants
		% of a country's pop using unsafely managed water	quantitative	Environment	Prevention & Control/ Social Determinants
		Mg/PCU/y of total antimicrobial consumption by animals	quantitative	Animal	Stewardship
		Livestock, crop, aquaculture production indices (metric tonnes)	quantitative	Animal	Stewardship
4	A One-Health Quantitative Model to Assess the Risk of Antibiotic Resistance Acquisition in Asian Populations: Impact of Exposure Through Food, Water, Livestock and Humans	Community model: frequency of antibiotics misuse in the community	quantitative	Human	Stewardship
		Water model: % of population with access to improved water on premises	quantitative	Environment	Prevention & Control/ Social Determinants
		Community model: % of population with access to hygiene installation	quantitative	Environment	Prevention & Control/ Social Determinants
		Community model: % of population with access to private sanitation	quantitative	Environment	Prevention & Control/ Social Determinants
		Food model: level of antibiotic use for production in farms	quantitative	Animal	Stewardship
		Food model: prevalence of natural ARB in animals	quantitative	Animal	Surveillance & Laboratory

		Food model: probability of acquisition of bacteria when consuming contaminated product	quantitative	Animal	Prevention & Control
		Livestock model: portion of individuals involved in husbandry	quantitative	Animal	Workforce
		Livestock model: average n of animals per family/industrial-type farm	quantitative	Animal	Prevention & Control
		Livestock model: n of animal-human contacts per animal and per year for family/industrial-type farms	quantitative	Animal	Prevention & Control
5	A qualitative approach for a situation analysis of AMR risks in the food animal production sector	AMR introduction risk factor: Existence of a traceability system for medicated concentrated feed from production to the farm of destination	qualitative	Animal	Stewardship
		AMR introduction risk factor: Auditable registry of feed used in animal production	qualitative	Animal	Stewardship
		AMR introduction risk factor: Classification of antibiotics used within the WOAH List of Antimicrobial Agents of Veterinary Importance (22) and WHO List of Critically Important Antimicrobials for Human Medicine	qualitative	Animal	Stewardship
		AMR exposure risk factor: n of people at national level employed in animal production and animal products processing plants	qualitative	Animal	Workforce
6	A review of national action plans on antimicrobial resistance: strengths and weaknesses	Multisectoral collaboration & involvement of stakeholders by country: contributors listed	Likert scale (fully addressed or located, partially addressed or all responses not located, not addressed or cannot be located)	Multisector	Coordination/ Participation
		Multisectoral collaboration & involvement of stakeholders by country: human health	Likert scale (fully addressed or located, partially addressed or all responses not located, not	Multisector	Coordination/ Participation

			addressed or cannot be located)		
		Multisectoral collaboration & involvement of stakeholders by country: agriculture	Likert scale (fully addressed or located, partially addressed or all responses not located, not addressed or cannot be located)	Environment	Coordination/ Participation
		Multisectoral collaboration & involvement of stakeholders by country: veterinary health	Likert scale (fully addressed or located, partially addressed or all responses not located, not addressed or cannot be located)	Animal	Coordination/ Participation
7	An analysis of existing national action plans for antimicrobial resistance-gaps and opportunities in strategies optimising antibiotic use in human populations	n and % of NAPs (by income group) that mentioned submission of AMR data to GLASS database	quantitative	Human	Reporting
		n and % of NAPs (by income group) with AMR data available in GLASS database	quantitative	Human	Reporting
		n and % of NAPs (by income group) with available data for antibiotic use or consumption	quantitative	Human	Reporting
		n and % of NAPs (by income group) with antibiotic consumption data available in WHO Report	quantitative	Human	Reporting
		Availability of NAPs by WHO Region (% of region)	quantitative	Multisector	Transparency
8	An analysis of national action plans on antimicrobial resistance in Southeast Asia using a governance framework approach	Comparison of objectives in each NAP against objectives in the GAP	qualitative	Multisector	Strategic Vision
		Implementation tools: optimising AMU (description of each NAP)	qualitative	Multisector	Stewardship
		Implementation tools: IPC (description of each NAP)	qualitative	Multisector	Prevention & Control

		Implementation tools: education (description of each NAP)	qualitative	Multisector	Community Awareness & Enabling Behaviours
		Implementation tools: international collaboration (description of each NAP)	qualitative	Multisector	Coordination
9	An elephant in the room? Explaining agenda-setting in antimicrobial resistance policies in 30 European countries	n of parties addressing AMR in their manifesto by country	quantitative	Multisector	Strategic Vision
		Sector in which AMR is addressed in manifesto by country	qualitative	Multisector	Strategic Vision
		Association between presence of AMR statement in manifesto and whether it was a Green party	quantitative	Multisector	Strategic Vision
		Association between presence of AMR statement in manifesto and whether it was a peasant/pro-agrarian party	quantitative	Multisector	Strategic Vision
		Association between presence of AMR statement in manifesto and vote share (%)	quantitative	Multisector	Strategic Vision
		Association between presence of AMR statement in manifesto and EU membership	quantitative	Multisector	Coordination/ Strategic Vision
		Association between presence of AMR statement in manifesto and region (E,W)	quantitative	Multisector	Strategic Vision
		Association between presence of AMR statement in manifesto and country average antibiotic consumption (%)	quantitative	Multisector	Strategic Vision
		Association between presence of AMR statement in manifesto and country average antibiotic knowledge (%)	quantitative	Multisector	Strategic Vision
		Association between presence of AMR statement in manifesto and country average in tackling resistance at the national level (%)	quantitative	Multisector	Strategic Vision
		AMR problem identification and proposed measures/instruments by manifesto	qualitative	Multisector	Strategic Vision
10	An insight into the implementation of the global action plan on antimicrobial resistance in the WHO AFR: A roadmap for action	Global Action Plan (GAP) performance score	quantitative	Multisector	Strategic Vision

11	Anthropological and socioeconomic factors contributing to global antimicrobial resistance: a univariate and multivariable analysis	Ratio of public to private health expenditure	quantitative	Human	Access to Medicines & Health Services
		E. coli resistance levels (%) for fluoroquinolones and 3G cephalosporins compared with antibiotic consumption (DDD)	quantitative	Human	Surveillance & Laboratory
		Average °C	quantitative	Multisector	Social Determinants
		Urbanisation (% of population)	quantitative	Multisector	Social Determinants
		Health expenditure, total (% of GDP)	quantitative	Multisector	Access to Medicines & Health Services
		Corruption	Ordinal (1-100)	Multisector	Social Determinants
		GDPpc, 2015 (PPP in 2011, US$)	quantitative	Multisector	Social Determinants
		Literacy (%)	quantitative	Multisector	Social Determinants
		Literacy (average of male and female rates)	quantitative	Multisector	Social Determinants
		Ratio of female to male literacy	quantitative	Multisector	Social Determinants
		Female literacy (%)	quantitative	Multisector	Social Determinants
		Male literacy (%)	quantitative	Multisector	Social Determinants
		% finishing secondary education	quantitative	Multisector	Social Determinants
		Primary school completion rate	quantitative	Multisector	Social Determinants
		Average precipitation (mm per year)	quantitative	Multisector	Social Determinants
		Access to electricity (% of population)	quantitative	Multisector	Social Determinants
		Internet penetration (% of population)	quantitative	Multisector	Social Determinants
		Political stability and absence of violence	quantitative	Multisector	Social Determinants
		Rule of law	quantitative	Multisector	Social Determinants
		Adequate sanitation, 2015 (% of population)	quantitative	Environment	Prevention & Control/ Social Determinants
		Access to improved water source (% of population)	quantitative	Environment	Prevention & Control/ Social Determinants
12	Antibiotic Consumption in Primary Care in Costa Rica and Italy: A Retrospective Cross-Country Analysis	ATC J01 DID in Italy	quantitative	Human	Stewardship
		ATC J01 DID in Campania	quantitative	Human	Stewardship
		ATC J01 DID in LHANS	quantitative	Human	Stewardship

		ATC J01 DID in Costa Rican private sector	quantitative	Human	Stewardship
		ATC J01 gross expenditure (€) per 100,000 inhabitant/day for Campania	quantitative	Human	Social Determinants
		ATC J01 gross expenditure (€) per 100,000 inhabitant/day for Italy	quantitative	Human	Social Determinants
		ATC J01 gross expenditure (€) per 100,000 inhabitant/day for LHANS	quantitative	Human	Social Determinants
		ATC J01 gross expenditure (€) per 100,000 inhabitant/day for Costa Rican private sector	quantitative	Human	Social Determinants
13	Antibiotic exposure among children younger than 5 years in low-income and middle-income countries: a cross-sectional study of nationally representative facility-based and household-based surveys	Proportion of children <5 with respiratory infection, diarrhoea, malaria who were prescribed antibiotic (by country)	quantitative	Human	Stewardship
		Estimated age-specific and cumulative antibiotic exposure of children from birth to age 5	quantitative	Human	Stewardship
		Proportion of children with cough/fever who visited a formal sector HC facility (%) by country average	quantitative	Human	Access to Medicines & Health Services
		Proportion of children with cough/fever who visited an informal sector HC provider (%) by country average	quantitative	Human	Access to Medicines & Health Services
		Proportion of formal HC facility visits unrelated to cough/fever (%) by country average	quantitative	Human	Access to Medicines & Health Services
		Estimated cumulative mean n of visits to a HC facility between birth and age 5 by country average	quantitative	Human	Access to Medicines & Health Services
		Mean n of antibiotics prescribed/sick-child visit to a HC facility	quantitative	Human	Stewardship
		Proportion of children prescribed an antibiotic (%) (by antibiotic type, age (y), sex, type of illness, HC provider qualification, type of facility, type of ownership)	quantitative	Human	Stewardship
14	Antibiotic Exposure and Other Risk Factors for Antimicrobial Resistance in Nasal Commensal Staphylococcus	AMR % by country	quantitative	Multisector	Surveillance & Laboratory
		MDR % by country	quantitative	Multisector	Surveillance & Laboratory
		MRSA % by country	quantitative	Multisector	Surveillance & Laboratory

	aureus: An Ecological Study in 8 European Countries	Antibiotic prescriptions per 100 active patients by country	quantitative	Human	Stewardship
		Proportional prescription patterns (%) of antibiotic classes by country	quantitative	Human	Stewardship
15	Antibiotic resistance patterns in Escherichia coli from gulls in nine European countries	MDR (≥3 antibiotics) phenotype (all combinations) distribution by country	quantitative	Human	Surveillance & Laboratory
16	Antibiotic treatment patterns across Europe in patients with complicated skin and soft-tissue infections due to meticillin-resistant Staphylococcus aureus: a plea for implementation of early switch and early discharge criteria	% of hospitals with antibiotic subcommittees and early switch and early discharge protocols (antibiotic/steering subcommittee, i.v.-to-oral antibiotic switch protocol, ED protocol for MRSA cSSTI)	quantitative	Human	Stewardship
		MRSA-active antibiotic treatment patterns by country, n and % (therapies administered in hospital, prescribed at discharge)	quantitative	Human	Prevention & Control
		Actual length of i.v. therapy and hospital LOS in days	quantitative	Human	Stewardship
17	Antibiotic use in Australian and Swedish primary care: a cross-country comparison	Dispensed use of antibiotics (DID) by country	quantitative	Human	Stewardship
		Dispensed use of lincosamides (DID) by country	quantitative	Human	Stewardship
		Dispensed use of penicillins & beta-lactamase inhibitors (DID) by country	quantitative	Human	Stewardship
		Dispensed use of beta-lactamase-sensitive penicillins (DID) by country	quantitative	Human	Stewardship
		Dispensed use of cephalosporins (DID) by country	quantitative	Human	Stewardship
		Dispensed use of beta-lactamase-resistant penicillins (DID) by country	quantitative	Human	Stewardship
		Dispensed use of penicillins with extended spectrum (DID) by country	quantitative	Human	Stewardship
		Dispensed use of tetracyclines (DID) by country	quantitative	Human	Stewardship
		Dispensed use of macrolides (DID) by country	quantitative	Human	Stewardship
		Dispensed use of sulphonamides & trimethoprim (DID) by country	quantitative	Human	Stewardship

		Dispensed use of fluoroquinolones (DID) by country	quantitative	Human	Stewardship
18	Antimicrobial Medicines Consumption in Eastern Europe and Central Asia - An Updated Cross-National Study and Assessment of Quantitative Metrics for Policy Action	Antibiotic consumption (DID) by country (tetracyclines)	quantitative	Human	Stewardship
		Antibiotic consumption (DID) by country (beta-lactams)	quantitative	Human	Stewardship
		Antibiotic consumption (DID) by country (sulfonamides & trimethoprim)	quantitative	Human	Stewardship
		Antibiotic consumption (DID) by country (macrolides, lincosamides & streptogramins)	quantitative	Human	Stewardship
		Consumption of cephalosporins and quinolones (as % of total consumption) by country	quantitative	Human	Stewardship
		Relative consumption of cephalosporins (as % of total cephalosporin consumption) by generation of agents , by country	quantitative	Human	Stewardship
		Consumption of "Watch" group of antibiotics classes (as % of total consumption) by country	quantitative	Human	Stewardship
		Total antibiotic consumption (DID) by country	quantitative	Human	Stewardship
		Total antibacterial consumption (DID) for systemic used by route of administration (oral, parenteral), by country	quantitative	Human	Stewardship
		Antibiotic consumption (DID) by country (quinolone)	quantitative	Human	Stewardship
		Antibiotic consumption (DID) by country (amphenicols)	quantitative	Human	Stewardship
		Antibiotic consumption (DID) by country (cephalosporins)	quantitative	Human	Stewardship
		Antibiotic consumption (DID) by country (other)	quantitative	Human	Stewardship
19	Antimicrobial Resistance and Environmental Health: A Water Stewardship Framework for Global and National Action	NAP evaluation: Human consumption scorecard by country and income level	quantitative	Human	Stewardship
		NAP evaluation: Human IPC scorecard by country and income level	quantitative	Human	Prevention & Control
		NAP evaluation: Human One Health sector scorecard by country and income level	quantitative	Human	Effectiveness

		One Health-One Water AMR stewardship framework scoring criteria (not included in NAP, acknowledged but no plan of action in NAP, plan of action outlined in NAP, implementation in NAP, established plan of action/stewardship programme in NAP)	ordinal (0, 0.25, 0.5, 0.75, 1)	Multisector	Strategic Vision/ Stewardship
		NAP evaluation: Food safety and security scorecard by country and income level	quantitative	Environment	Prevention & Control
		NAP evaluation: AMU in plants scorecard by country and income level	quantitative	Environment	Stewardship
		NAP evaluation: Environmental contamination scorecard by country and income level	quantitative	Environment	Prevention & Control
		NAP evaluation: Clean water & sanitation scorecard by country and income level	quantitative	Environment	Prevention & Control/ Social Determinants
		NAP evaluation: One Health environment sector scorecard by country and income level	quantitative	Environment	Prevention & Control
		Proportion of pop using safely managed drinking water service by country	quantitative	Environment	Prevention & Control
		NAP evaluation: Stewardship pillar - prevention scorecard by country and income	quantitative	Multisector	Prevention & Control
		NAP evaluation: Stewardship pillar - mitigation scorecard by country and income	quantitative	Multisector	Prevention & Control
		NAP evaluation: Stewardship pillar - surveillance scorecard by country and income	quantitative	Multisector	Surveillance & Laboratory
		NAP evaluation: Stewardship pillar - innovation scorecard by country and income	quantitative	Multisector	Research, Innovation, & Digital Technology
		NAP evaluation: Animal One Health sector scorecard by country and income level	quantitative	Animal	Prevention & Control, and Stewardship
		NAP evaluation: AMU in animals scorecard by country and income level	quantitative	Animal	Stewardship
20	Antimicrobial resistance genes aph(3')-III, erm(B), sul2 and tet(W) abundance in animal faeces, meat, production	Relative abundance (ARG copies/16S copies) of four target genes in retail veal	quantitative	Animal	Surveillance & Laboratory
		Relative abundance (ARG copies/16S copies) of four target genes in retail turkey	quantitative	Animal	Surveillance & Laboratory

environments and human faeces in Europe	Relative abundance (ARG copies/16S copies) of four target genes in retail pork	quantitative	Animal	Surveillance & Laboratory
	Relative abundance (ARG copies/16S copies) of four target genes in retail fish	quantitative	Animal	Surveillance & Laboratory
	Relative abundance (ARG copies/16S copies) of four target genes in retail chicken	quantitative	Animal	Surveillance & Laboratory
	Relative abundance (ARG copies/16S copies) of four target genes in gloves (pig slaughterhouses)	quantitative	Animal	Surveillance & Laboratory
	Relative abundance (ARG copies/16S copies) of four target genes in gloves (broiler slaughterhouses)	quantitative	Animal	Surveillance & Laboratory
	Relative abundance (ARG copies/16S copies) of four target genes in EDC (pig farms)	quantitative	Animal	Surveillance & Laboratory
	Relative abundance (ARG copies/16S copies) of four target genes in EDC (broiler farms)	quantitative	Animal	Surveillance & Laboratory
	Relative abundance (ARG copies/16S copies) of four target genes in wild boar faeces	quantitative	Animal	Surveillance & Laboratory
	Relative abundance (ARG copies/16S copies) of four target genes in veal calf faeces (farms)	quantitative	Animal	Surveillance & Laboratory
	Relative abundance (ARG copies/16S copies) of four target genes in turkey faeces (farms)	quantitative	Animal	Surveillance & Laboratory
	Relative abundance (ARG copies/16S copies) of four target genes in pig faeces (farms)	quantitative	Animal	Surveillance & Laboratory
	Relative abundance (ARG copies/16S copies) of four target genes in pig faeces (slaughterhouse)	quantitative	Animal	Surveillance & Laboratory
	Relative abundance (ARG copies/16S copies) of four target genes in pig carcass (slaughterhouse)	quantitative	Animal	Surveillance & Laboratory
	Relative abundance (ARG copies/16S copies) of four target genes in pig meat (slaughterhouse)	quantitative	Animal	Surveillance & Laboratory
	Relative abundance (ARG copies/16S copies) of four target genes in fish faeces (farms)	quantitative	Animal	Surveillance & Laboratory
	Relative abundance (ARG copies/16S copies) of four target genes in dog faeces	quantitative	Animal	Surveillance & Laboratory
	Relative abundance (ARG copies/16S copies) of four target genes in cat faeces	quantitative	Animal	Surveillance & Laboratory

		Relative abundance (ARG copies/16S copies) of four target genes in broiler faeces (farms)	quantitative	Animal	Surveillance & Laboratory
		Relative abundance (ARG copies/16S copies) of four target genes in broiler faeces (slaughterhouse)	quantitative	Animal	Surveillance & Laboratory
		Relative abundance (ARG copies/16S copies) of four target genes in broiler carcass (slaughterhouse)	quantitative	Animal	Surveillance & Laboratory
		Relative abundance (ARG copies/16S copies) of four target genes in broiler meat (slaughterhouse)	quantitative	Animal	Surveillance & Laboratory
		Relative abundance (ARG copies/16S copies) of four target genes in human faeces control	quantitative	Animal	Surveillance & Laboratory
		Relative abundance (ARG copies/16S copies) of four target genes in human faeces (pig slaughterhouses)	quantitative	Animal	Surveillance & Laboratory
		Relative abundance (ARG copies/16S copies) of four target genes in human faeces (pig farms)	quantitative	Animal	Surveillance & Laboratory
		Relative abundance (ARG copies/16S copies) of four target genes in human faeces (broiler slaughterhouses)	quantitative	Animal	Surveillance & Laboratory
		Relative abundance (ARG copies/16S copies) of four target genes in human faeces (broiler farms)	quantitative	Animal	Surveillance & Laboratory
21	Antimicrobial Resistance Policy Protagonists and Processes-A Qualitative Study of Policy Advocacy and Implementation	n of policy makers associated with AMR policymaking by country	quantitative	Multisector	Participation
		n of hospital directors & leaders associated with AMR policymaking by country	quantitative	Multisector	Participation
		n of healthcare & public health officials associated with AMR policymaking by country	quantitative	Multisector	Participation
		n of medical professionals (pharmacists, dentists, doctors & vets) associated with AMR policymaking by country	quantitative	Multisector	Participation
		n of diplomats, bureaucrats and policy administrators associated with AMR policymaking by country	quantitative	Multisector	Participation

		n of policy advisors associated with AMR policymaking by country	quantitative	Multisector	Participation
22	Antimicrobial resistance preparedness in WHO AFR (Sub-Saharan Africa) countries	% of countries reported having AMR indicators in place (NAP, surveillance, IPC, AM stewardship)	quantitative	Multisector	Stewardship
		Mean SSA AMR score	quantitative	Multisector	Surveillance & Laboratory
		JEE (Joint External Evaluation) completion status	Y/N	Multisector	Prevention & Control/ Participation
		Overall JEE score	quantitative	Multisector	Prevention & Control
23	Antimicrobial resistance prevalence in bloodstream infection in 29 European countries by age and sex: An observational study	BSI incidence (infections/100,000 pop/year) for E. coli, by age & sex	quantitative	Human	Surveillance & Laboratory
		BSI incidence (infections/100,000 pop/year) for S. pneumoniae, by age & sex	quantitative	Human	Surveillance & Laboratory
		BSI incidence (infections/100,000 pop/year) for K. pneumoniae, by age & sex	quantitative	Human	Surveillance & Laboratory
		BSI incidence (infections/100,000 pop/year) for Acinetobacter spp, by age & sex	quantitative	Human	Surveillance & Laboratory
		Proportion of Acinetobacter spp isolates resistant to amikacin, by age	quantitative	Human	Surveillance & Laboratory
		Proportion of Acinetobacter spp isolates resistant to aminoglycoside, by age	quantitative	Human	Surveillance & Laboratory
		Proportion of Acinetobacter spp isolates resistant to carbapenem, by age	quantitative	Human	Surveillance & Laboratory
		Proportion of Acinetobacter spp isolates resistant to ceftazidime, by age	quantitative	Human	Surveillance & Laboratory
		Proportion of Acinetobacter spp isolates resistant to fluoroquinolone, by age	quantitative	Human	Surveillance & Laboratory
		Proportion of Acinetobacter spp isolates resistant to ureidopenicillin, by age	quantitative	Human	Surveillance & Laboratory
		Proportion of P. aeruginosa isolates resistant to amikacin, by age	quantitative	Human	Surveillance & Laboratory
		Proportion of P. aeruginosa isolates resistant to aminoglycoside, by age	quantitative	Human	Surveillance & Laboratory
		Proportion of P. aeruginosa isolates resistant to carbapenem, by age	quantitative	Human	Surveillance & Laboratory

Proportion of P. aeruginosa isolates resistant to ceftazidime, by age	quantitative	Human	Surveillance & Laboratory
Proportion of P. aeruginosa isolates resistant to fluoroquinolone, by age	quantitative	Human	Surveillance & Laboratory
Proportion of P. aeruginosa isolates resistant to ureidopenicillin, by age	quantitative	Human	Surveillance & Laboratory
Proportion of E. coli isolates resistant to 3G cephalosporins, by age	quantitative	Human	Surveillance & Laboratory
Proportion of E. coli isolates resistant to amikacin, by age	quantitative	Human	Surveillance & Laboratory
Proportion of E. coli isolates resistant to aminoglycoside, by age	quantitative	Human	Surveillance & Laboratory
Proportion of E. coli isolates resistant to aminopenicillins, by age	quantitative	Human	Surveillance & Laboratory
Proportion of E. coli isolates resistant to carbapenem, by age	quantitative	Human	Surveillance & Laboratory
Proportion of E. coli isolates resistant to ertapenem, by age	quantitative	Human	Surveillance & Laboratory
Proportion of E. coli isolates resistant to fluoroquinolone, by age	quantitative	Human	Surveillance & Laboratory
Proportion of E. coli isolates resistant to ureidopenicillin, by age	quantitative	Human	Surveillance & Laboratory
Proportion of K. pneumoniae isolates resistant to 3G cephalosporins, by age	quantitative	Human	Surveillance & Laboratory
Proportion of K. pneumoniae isolates resistant to amikacin, by age	quantitative	Human	Surveillance & Laboratory
Proportion of K. pneumoniae isolates resistant to aminoglycoside, by age	quantitative	Human	Surveillance & Laboratory
Proportion of K. pneumoniae isolates resistant to aminopenicillins, by age	quantitative	Human	Surveillance & Laboratory
Proportion of K. pneumoniae isolates resistant to carbapenem, by age	quantitative	Human	Surveillance & Laboratory
Proportion of K. pneumoniae isolates resistant to ertapenem, by age	quantitative	Human	Surveillance & Laboratory

	Proportion of K. pneumoniae isolates resistant to fluoroquinolone, by age	quantitative	Human	Surveillance & Laboratory
	Proportion of K. pneumoniae isolates resistant to ureidopenicillin, by age	quantitative	Human	Surveillance & Laboratory
	Proportion of E. faecalis isolates resistant to aminopenicillins, by age	quantitative	Human	Surveillance & Laboratory
	Proportion of E. faecalis isolates resistant to gentamicin, by age	quantitative	Human	Surveillance & Laboratory
	Proportion of E. faecalis isolates resistant to vancomycin, by age	quantitative	Human	Surveillance & Laboratory
	Proportion of E. faecium isolates resistant to aminopenicillins, by age	quantitative	Human	Surveillance & Laboratory
	Proportion of E. faecium isolates resistant to gentamicin, by age	quantitative	Human	Surveillance & Laboratory
	Proportion of E. faecium isolates resistant to vancomycin, by age	quantitative	Human	Surveillance & Laboratory
	Proportion of S. aureus isolates resistant to 3G cephalosporins, by age	quantitative	Human	Surveillance & Laboratory
	Proportion of S. aureus isolates resistant to fluoroquinolone, by age	quantitative	Human	Surveillance & Laboratory
	Proportion of S. aureus isolates resistant to macrolides, by age	quantitative	Human	Surveillance & Laboratory
	Proportion of S. aureus isolates resistant to methicillin, by age	quantitative	Human	Surveillance & Laboratory
	Proportion of S. aureus isolates resistant to penicillins, by age	quantitative	Human	Surveillance & Laboratory
	Proportion of S. aureus isolates resistant to rifampicin, by age	quantitative	Human	Surveillance & Laboratory
	Proportion of S. pneumoniae isolates resistant to 3G cephalosporins, by age	quantitative	Human	Surveillance & Laboratory
	Proportion of S. pneumoniae isolates resistant to fluoroquinolone, by age	quantitative	Human	Surveillance & Laboratory
	Proportion of S. pneumoniae isolates resistant to macrolides, by age	quantitative	Human	Surveillance & Laboratory

Proportion of S. pneumoniae isolates resistant to methicillin, by age	quantitative	Human	Surveillance & Laboratory
Proportion of S. pneumoniae isolates resistant to penicillins, by age	quantitative	Human	Surveillance & Laboratory
Proportion of S. pneumoniae isolates resistant to rifampicin, by age	quantitative	Human	Surveillance & Laboratory
BSI incidence (infections/100,000 pop/year) for E. faecium, by age & sex	quantitative	Human	Surveillance & Laboratory
BSI incidence (infections/100,000 pop/year) for E. faecalis, by age & sex	quantitative	Human	Surveillance & Laboratory
BSI incidence (infections/100,000 pop/year) for S. aureus, by age & sex	quantitative	Human	Surveillance & Laboratory
BSI incidence (infections/100,000 pop/year) for P. aeruginosa, by age & sex	quantitative	Human	Surveillance & Laboratory
BSI incidence (total) in ED, by bacteria type, sex & age	quantitative	Human	Surveillance & Laboratory
BSI incidence (total) in INTMED, by bacteria type, sex & age	quantitative	Human	Surveillance & Laboratory
BSI incidence (total) in ICU, by bacteria type, sex & age	quantitative	Human	Surveillance & Laboratory
BSI incidence (total) in URO, by bacteria type, sex & age	quantitative	Human	Surveillance & Laboratory
BSI incidence (total) in SURG, by bacteria type, sex & age	quantitative	Human	Surveillance & Laboratory
BSI incidence (total) in ONCOL, by bacteria type, sex & age	quantitative	Human	Surveillance & Laboratory
BSI incidence (total) in OBGYN, by bacteria type, sex & age	quantitative	Human	Surveillance & Laboratory
BSI incidence (total) in INFECT, by bacteria type, sex & age	quantitative	Human	Surveillance & Laboratory
BSI incidence (total) in PEDS, by bacteria type, sex & age	quantitative	Human	Surveillance & Laboratory
BSI incidence (total) in PEDS ICU, by bacteria type, sex & age	quantitative	Human	Surveillance & Laboratory

		BSI incidence (total) in Other hospital unit, by bacteria type, sex & age	quantitative	Human	Surveillance & Laboratory
24	Antimicrobial resistance prevalence in commensal Escherichia coli from broilers, fattening turkeys, fattening pigs and veal calves in European countries and association with antimicrobial usage at country level	Fattening pigs: resistance (%) of E. coli isolates by antimicrobial and country	quantitative	Animal	Surveillance & Laboratory
		Broilers: resistance (%) of E. coli isolates by antimicrobial and country	quantitative	Animal	Surveillance & Laboratory
		Veal calves: resistance (%) of E. coli isolates by antimicrobial and country	quantitative	Animal	Surveillance & Laboratory
		Fattening turkeys: resistance (%) of E. coli isolates by antimicrobial and country	quantitative	Animal	Surveillance & Laboratory
		Fully susceptible E. coli (%) by country and animal species	quantitative	Animal	Surveillance & Laboratory
		MDR E. coli (%) by country and animal species	quantitative	Animal	Surveillance & Laboratory
		Cefotaxime and/or ceftazidime-resistant E. coli (%) by country and animal species	quantitative	Animal	Surveillance & Laboratory
		Ciprofloxacin-resistant E. coli (%) by country and animal species	quantitative	Animal	Surveillance & Laboratory
		Fattening pigs: AMU (TI/1000) by antimicrobial and country	quantitative	Animal	Stewardship
		Broilers: AMU (TI/1000) by antimicrobial and country	quantitative	Animal	Stewardship
		Veal calves: AMU (TI/1000) by antimicrobial and country	quantitative	Animal	Stewardship
		Fattening turkeys: AMU (TI/1000) by antimicrobial and country	quantitative	Animal	Stewardship
25	Antimicrobial Resistance Rates and Surveillance in Sub-Saharan Africa: Where Are We Now?	Meropenem-resistant clinical A. baumannii isolates (%) by country	quantitative	Human	Surveillance & Laboratory
		Trimethoprim/sulfamethoxazole-resistant clinical A. baumannii isolates (%) by country	quantitative	Human	Surveillance & Laboratory
		Imipenem-resistant clinical A. baumannii isolates (%) by country	quantitative	Human	Surveillance & Laboratory
		Levofloxacin-resistant clinical A. baumannii isolates (%) by country	quantitative	Human	Surveillance & Laboratory
		Ceftazidime-resistant clinical A. baumannii isolates (%) by country	quantitative	Human	Surveillance & Laboratory

	Cefepime-resistant clinical A. baumannii isolates (%) by country	quantitative	Human	Surveillance & Laboratory
	Ciprofloxacin-resistant clinical A. baumannii isolates (%) by country	quantitative	Human	Surveillance & Laboratory
	Gentamicin-resistant clinical A. baumannii isolates (%) by country	quantitative	Human	Surveillance & Laboratory
	MRSA isolates (%) by country	quantitative	Human	Surveillance & Laboratory
	Clindamycin-resistant S. aureus isolates (%) by country	quantitative	Human	Surveillance & Laboratory
	Vancomycin-resistant S. aureus isolates (%) by country	quantitative	Human	Surveillance & Laboratory
	Penicillin-resistant S. aureus isolates (%) by country	quantitative	Human	Surveillance & Laboratory
	Trimethoprim/sulfamethoxazole-resistant S. aureus isolates (%) by country	quantitative	Human	Surveillance & Laboratory
	Tetracycline-resistant S. aureus isolates (%) by country	quantitative	Human	Surveillance & Laboratory
	Ciprofloxacin-resistant S. aureus isolates (%) by country	quantitative	Human	Surveillance & Laboratory
	Erythromycin-resistant S. aureus isolates (%) by country	quantitative	Human	Surveillance & Laboratory
	Chloramphenicol-resistant S. aureus isolates (%) by country	quantitative	Human	Surveillance & Laboratory
	Gentamicin-resistant S. aureus isolates (%) by country	quantitative	Human	Surveillance & Laboratory
	Ciprofloxacin-resistant clinical S. Typhi isolates (%) by country	quantitative	Human	Surveillance & Laboratory
	Ceftriaxone-resistant clinical S. Typhi isolates (%) by country	quantitative	Human	Surveillance & Laboratory
	Tetracycline-resistant DEC isolates (%) by country	quantitative	Human	Surveillance & Laboratory
	Cotrimoxazole-resistant DEC isolates (%) by country	quantitative	Human	Surveillance & Laboratory
	Chloramphenicol-resistant DEC isolates (%) by country	quantitative	Human	Surveillance & Laboratory

	Ceftazidime-resistant DEC isolates (%) by country	quantitative	Human	Surveillance & Laboratory
	Cefotaxime-resistant DEC isolates (%) by country	quantitative	Human	Surveillance & Laboratory
	Ciprofloxacin-resistant DEC isolates (%) by country	quantitative	Human	Surveillance & Laboratory
	Nalidixic acid-resistant DEC isolates (%) by country	quantitative	Human	Surveillance & Laboratory
	Ceftriaxone-resistant DEC isolates (%) by country	quantitative	Human	Surveillance & Laboratory
	Ampicillin-resistant DEC isolates (%) by country	quantitative	Human	Surveillance & Laboratory
	Amoxicillin/clavulanic acid-resistant DEC isolates (%) by country	quantitative	Human	Surveillance & Laboratory
	Gentamicin-resistant DEC isolates (%) by country	quantitative	Human	Surveillance & Laboratory
	Imipenem-resistant DEC isolates (%) by country	quantitative	Human	Surveillance & Laboratory
	Doxycycline-resistant V. cholerae isolates (%) by country	quantitative	Human	Surveillance & Laboratory
	Azithromycin-resistant V. cholerae isolates (%) by country	quantitative	Human	Surveillance & Laboratory
	Ciprofloxacin-resistant V. cholerae isolates (%) by country	quantitative	Human	Surveillance & Laboratory
	Nalidixic acid-resistant V. cholerae isolates (%) by country	quantitative	Human	Surveillance & Laboratory
	Sulfamethoxazole–trimethoprim-resistant V. cholerae isolates (%) by country	quantitative	Human	Surveillance & Laboratory
	Ampicillin-resistant V. cholerae isolates (%) by country	quantitative	Human	Surveillance & Laboratory
	Gentamicin-resistant V. cholerae isolates (%) by country	quantitative	Human	Surveillance & Laboratory
	Tetracycline-resistant V. cholerae isolates (%) by country	quantitative	Human	Surveillance & Laboratory
	Tetracycline-resistant clinical S. Typhi isolates (%) by country	quantitative	Human	Surveillance & Laboratory

	Cotrimoxazole-resistant clinical S. Typhi isolates (%) by country	quantitative	Human	Surveillance & Laboratory
	Chloramphenicol-resistant clinical S. Typhi isolates (%) by country	quantitative	Human	Surveillance & Laboratory
	Ceftazidime-resistant clinical S. Typhi isolates (%) by country	quantitative	Human	Surveillance & Laboratory
	Cefotaxime-resistant clinical S. Typhi isolates (%) by country	quantitative	Human	Surveillance & Laboratory
	Nalidixic acid-resistant clinical S. Typhi isolates (%) by country	quantitative	Human	Surveillance & Laboratory
	Ampicillin-resistant clinical S. Typhi isolates (%) by country	quantitative	Human	Surveillance & Laboratory
	Tetracycline-resistant clinical S. Typhimurium isolates (%) by country	quantitative	Human	Surveillance & Laboratory
	Cotrimoxazole-resistant clinical S. Typhimurium isolates (%) by country	quantitative	Human	Surveillance & Laboratory
	Chloramphenicol-resistant clinical S. Typhimurium isolates (%) by country	quantitative	Human	Surveillance & Laboratory
	Ceftazidime-resistant clinical S. Typhimurium isolates (%) by country	quantitative	Human	Surveillance & Laboratory
	Cefotaxime-resistant clinical S. Typhimurium isolates (%) by country	quantitative	Human	Surveillance & Laboratory
	Ciprofloxacin-resistant clinical S. Typhimurium isolates (%) by country	quantitative	Human	Surveillance & Laboratory
	Nalidixic acid-resistant clinical S. Typhimurium isolates (%) by country	quantitative	Human	Surveillance & Laboratory
	Ceftriaxone-resistant clinical S. Typhimurium isolates (%) by country	quantitative	Human	Surveillance & Laboratory
	Azithromycin-resistant clinical S. Typhimurium isolates (%) by country	quantitative	Human	Surveillance & Laboratory
	Ampicillin-resistant clinical S. Typhimurium isolates (%) by country	quantitative	Human	Surveillance & Laboratory
	Tetracycline-resistant clinical S. Enteritidis isolates (%) by country	quantitative	Human	Surveillance & Laboratory

		Cotrimoxazole-resistant clinical S. Enteritidis isolates (%) by country	quantitative	Human	Surveillance & Laboratory
		Chloramphenicol-resistant clinical S. Enteritidis isolates (%) by country	quantitative	Human	Surveillance & Laboratory
		Ceftazidime-resistant clinical S. Enteritidis isolates (%) by country	quantitative	Human	Surveillance & Laboratory
		Cefotaxime-resistant clinical S. Enteritidis isolates (%) by country	quantitative	Human	Surveillance & Laboratory
		Ciprofloxacin-resistant clinical S. Enteritidis isolates (%) by country	quantitative	Human	Surveillance & Laboratory
		Nalidixic acid-resistant clinical S. Enteritidis isolates (%) by country	quantitative	Human	Surveillance & Laboratory
		Ceftriaxone-resistant clinical S. Enteritidis isolates (%) by country	quantitative	Human	Surveillance & Laboratory
		Azithromycin-resistant clinical S. Enteritidis isolates (%) by country	quantitative	Human	Surveillance & Laboratory
		Ampicillin-resistant clinical S. Enteritidis isolates (%) by country	quantitative	Human	Surveillance & Laboratory
26	Antimicrobial resistance rates in gram-positive bacteria do not drive glycopeptides use	Time trends of rates (%) of MRSA and VRE resistance among enterococci isolates from BSI by country	quantitative	Human	Surveillance & Laboratory
		Time trend of rate (%) of glycopeptide use expressed as DDD/1000 inhabitants/day by country	quantitative	Human	Stewardship
27	Antimicrobial resistance: the major contribution of poor governance and corruption to this growing problem	Private health expenditure as % of national income	quantitative	Human	Access to Medicines & Health Services
		GDPpc in constant price	quantitative	Multisector	Social Determinants
		% of pop that is tertiary educated	quantitative	Multisector	Social Determinants
		Control of corruption in society	Ordinal (0-6)	Multisector	Social Determinants
		% of employed pop employed in agricultural sector	quantitative	Environment	Workforce
28	Antimicrobial susceptibility profiles of anaerobic bacteria, isolated from	Resistance (%) per antibiotic of gram-negative anaerobic bacteria: cefoxitin	quantitative	Human	Surveillance & Laboratory

	human clinical specimens, within different European and surrounding countries. A joint ESGAI study	Resistance (%) per antibiotic of gram-negative anaerobic bacteria: penicillin	quantitative	Human	Surveillance & Laboratory
		Resistance (%) per antibiotic of gram-negative anaerobic bacteria: clindamycin	quantitative	Human	Surveillance & Laboratory
		Resistance (%) per antibiotic of gram-negative anaerobic bacteria: piperacillin-tazobactam	quantitative	Human	Surveillance & Laboratory
		Resistance (%) per antibiotic of gram-negative anaerobic bacteria: metronidazole	quantitative	Human	Surveillance & Laboratory
		Resistance (%) per antibiotic of gram-negative anaerobic bacteria: amoxicillin-clavulanic acid	quantitative	Human	Surveillance & Laboratory
		Resistance (%) per antibiotic of gram-negative anaerobic bacteria: imipenem	quantitative	Human	Surveillance & Laboratory
		Resistance (%) per antibiotic of gram-positive anaerobic genera: cefoxitin	quantitative	Human	Surveillance & Laboratory
		Resistance (%) per antibiotic of gram-positive anaerobic genera: penicillin	quantitative	Human	Surveillance & Laboratory
		Resistance (%) per antibiotic of gram-positive anaerobic genera: clindamycin	quantitative	Human	Surveillance & Laboratory
		Resistance (%) per antibiotic of gram-positive anaerobic genera: amoxicillin-clavulanic acid	quantitative	Human	Surveillance & Laboratory
		Resistance (%) per antibiotic of gram-positive anaerobic genera: metronidazole	quantitative	Human	Surveillance & Laboratory
29	Antimicrobial utilization and resistance in Pseudomonas aeruginosa using segmented regression analysis: a comparative study between Serbia and eight European Countries	Annual DID for aminoglycosides by country	quantitative	Human	Stewardship
		Annual DID for fluoroquinolones by country	quantitative	Human	Stewardship
		Annual resistance prevalence (%) in piperacillin-tazobactam of P. aeruginosa by country	quantitative	Human	Surveillance & Laboratory
		Annual resistance prevalence (%) in ceftazidime of P. aeruginosa by country	quantitative	Human	Surveillance & Laboratory
		Annual resistance prevalence (%) in carbapenem of P. aeruginosa by country	quantitative	Human	Surveillance & Laboratory
		Annual resistance prevalence (%) in aminoglycosides of P. aeruginosa by country	quantitative	Human	Surveillance & Laboratory
		Annual resistance prevalence (%) in fluoroquinolones of P. aeruginosa by country	quantitative	Human	Surveillance & Laboratory

30	Are There Striking Differences in Outpatient Use of Antibiotics Between South Backa District, Serbia, and Some Scandinavian Countries?	Cost (EUR/DDD) and DDD/TID by antibiotic	quantitative	Human	Stewardship
		DDD/TID (%) by therapeutic subgroup and country	quantitative	Human	Stewardship
		DDD/TID (%) by penicillin subgroup and country	quantitative	Human	Stewardship
		DDD/TID (%) by cephalosporin subgroup and country	quantitative	Human	Stewardship
		DDD/TID (%) by tetracyclines subgroup and country	quantitative	Human	Stewardship
		DDD/TID (%) by macrolides & lincosamides subgroup and country	quantitative	Human	Stewardship
		DDD/TID (%) by quinolones subgroup and country	quantitative	Human	Stewardship
		DDD/TID (%) by sulfonamides & trimethoprim subgroup and country	quantitative	Human	Stewardship
31	Assessing transparency and accountability of national action plans on antimicrobial resistance in 15 African countries	Public availability of information (NAP, progress reports, AMR surveillance data, funding allocation, responsible body, responsible person/sector)	qualitative	Multisector	Transparency
		Responsible bodies (name of body, responsibilities) and persons (responsibility, affiliation) by country	qualitative	Multisector	Accountability
		Countries responding to item 5.1 of tripartite survey by response option (C-National AMR action plan developed, D-National AMR action plan approved by gov that reflects GAP objectives, + operational plan and monitoring arrangements, E-National AMR action plan has funding sources identified, is being implemented and hs relevant sectors involved with a defined monitoring and evaluation process in place)	qualitative	Multisector	Reporting
32	Assigning Defined Daily/Course Doses for Antimicrobials in Turkeys to Enable a Cross-Country Quantification and Comparison of Antimicrobial Use	DDD (turkeys) administered through feed by antibiotic	quantitative	Animal	Stewardship
		DDD (turkeys) administered through injection by antibiotic	quantitative	Animal	Stewardship

DDD (turkeys) administered through water by antibiotic	quantitative	Animal	Stewardship
DCD (turkeys) administered through feed by antibiotic	quantitative	Animal	Stewardship
DCD (turkeys) administered through injection by antibiotic	quantitative	Animal	Stewardship
DCD (turkeys) administered through water by antibiotic	quantitative	Animal	Stewardship
DDD (broilers) administered through feed by antibiotic	quantitative	Animal	Stewardship
DDD (broilers) administered through injection by antibiotic	quantitative	Animal	Stewardship
DDD (broilers) administered through water by antibiotic	quantitative	Animal	Stewardship
DCD (broilers) administered through feed by antibiotic	quantitative	Animal	Stewardship
DCD (broilers) administered through injection by antibiotic	quantitative	Animal	Stewardship
DCD (broilers) administered through water by antibiotic	quantitative	Animal	Stewardship
Deviation of DDD (turkeys) from corresponding DDD (broilers) value by antibiotic (administered through feed)	quantitative	Animal	Stewardship
Deviation of DDD (turkeys) from corresponding DDD (broilers) value by antibiotic (administered through water)	quantitative	Animal	Stewardship
Top 20 deviating antibiotic products from the mean DDD (turkeys) by administration route and country	quantitative	Animal	Stewardship
Median AMU expressed as TIDDD (turkeys)	quantitative	Animal	Stewardship
Median AMU expressed as TIDCD (turkeys)	quantitative	Animal	Stewardship
Median AMU expressed as TIDDD (broilers)	quantitative	Animal	Stewardship
Median AMU expressed as TIDCD (broilers)	quantitative	Animal	Stewardship

	AMU at each flock level (TIDDD turkeys) by country	quantitative	Animal	Stewardship
	Distribution of AMU over different antimicrobial classes by country	quantitative	Animal	Stewardship
	Running treatment incidence (%) in studied flocks by week of production	quantitative	Animal	Stewardship
	Proportion (%) of turkey flocks where treatments with antimicrobials were initiated by week of production	quantitative	Animal	Stewardship
	Proportion (%) of turkey flocks being treated with antimicrobials by week of production	quantitative	Animal	Stewardship
	n of products registered for use in turkeys by antimicrobial class, administration route and country	quantitative	Animal	Reporting
	Proportion (%) of total amount of antimicrobials used for turkeys by antimicrobial class, administration route and country	quantitative	Animal	Stewardship
	% of total n of treatments for intestinal disorders, by country	quantitative	Animal	Stewardship
	% of total n of treatments for respiratory disorders, by country	quantitative	Animal	Stewardship
	% of total n of treatments for colibacillosis, by country	quantitative	Animal	Stewardship
	% of total n of treatments for locomotive disorders, by country	quantitative	Animal	Stewardship
	% of total n of treatments for general disorders, by country	quantitative	Animal	Stewardship
	% of total n of treatments for non-specific disorders, by country	quantitative	Animal	Stewardship
	% of total n of treatments for higher mortality rate, by country	quantitative	Animal	Stewardship
	Proportion (%) of total amount of antimicrobials used for each disorder by week of production	quantitative	Animal	Stewardship
	Proportion (%) of each antimicrobial used by week of production	quantitative	Animal	Stewardship

33	Association between antibiotic resistance in intensive care unit (ICU)-acquired infections and excess resource utilization: evidence from Spain, Italy, and Portugal	Cost estimate (€) per resistant infection by country	quantitative	Human	Access to Medicines & Health Services
		Cost estimate (€) per susceptible infection by country	quantitative	Human	Access to Medicines & Health Services
		Cumulative discharge incidence by excess length of stay (resistant isolates) by country	quantitative	Human	Surveillance & Laboratory
		Cumulative discharge incidence by excess length of stay (susceptible isolates) by country	quantitative	Human	Surveillance & Laboratory
34	Attention to the Tripartite's one health measures in national action plans on antimicrobial resistance	% of NAPs addressing hygiene	quantitative	Human	Prevention & Control/ Social Determinants
		% of NAPs addressing waste management	quantitative	Environment	Prevention & Control/ Social Determinants
		% of NAPs addressing sanitation	quantitative	Environment	Prevention & Control/ Social Determinants
		% of NAPs addressing clean water	quantitative	Environment	Prevention & Control/ Social Determinants
35	Awareness regarding antimicrobial resistance and confidence to prescribe antibiotics in dentistry: a cross-continental student survey	Perceived awareness of climate change, food security, antibiotic resistance, gender inequality, obesity	Ordinal (1-10)	Multisector	Community Awareness & Enabling Behaviours
		Knowledge of the concept of One Health	quantitative (proportion of Y/N/not sure)	Multisector	Community Awareness & Enabling Behaviours
		Perception of which areas should be addressed to slow down the development of antibiotic resistance (AMU in humans and in animals, IPC practices, public awareness)	Ordinal (1-10)	Multisector	Community Awareness & Enabling Behaviours
		Participants' level of confidence concerning antibiotic prescriptions for treatment and prevention of infections (%)	quantitative	Multisector	Stewardship
		Interest of final year dental students in receiving further education and information on selected topics related to antibiotic resistance (%)	quantitative	Multisector	Community Awareness & Enabling Behaviours
		Engagement in national and international campaigns promoting awareness on antibiotic resistance	quantitative (proportion of Y/N/not sure)	Multisector	Community Awareness & Enabling Behaviours/ Participation

36	Baseline evaluation of the World Health Organization (WHO) infection prevention and control (IPC) core components in Pacific Island Countries and Territories (PICTs)	HAI surveillance: HAI defined component of national IPC programme (% of countries)	quantitative	Human	Surveillance & Laboratory
		IPC Programmes: National IPC Programme (% of countries)	quantitative	Multisector	Prevention & Control
		IPC Programmes: IPC focal point (% of countries)	quantitative	Multisector	Prevention & Control
		IPC Programmes: Dedicated budget (% of countries)	quantitative	Multisector	Prevention & Control
		National and Facility level IPC guidelines: Updated national IPC guidelines (% of countries)	quantitative	Multisector	Prevention & Control
		National and Facility level IPC guidelines: Guidelines adapted and implemented (% of countries)	quantitative	Multisector	Prevention & Control
		IPC education and training: Mandatory in-service training provided to healthcare workers (% of countries)	quantitative	Multisector	Workforce
		Multimodal strategies for IPC activities: Multimodal strategies being implemented (% of countries)	quantitative	Multisector	Prevention & Control
		IPC monitoring and feedback: Monitoring/audit of IPC practices (% of countries)	quantitative	Multisector	Prevention & Control
		Overall progress (%) by country (Not present, in progress, present)	quantitative	Multisector	Prevention & Control
		Built environment, materials and equipment: b.Waste management plan (% of countries)	quantitative	Environment	Prevention & Control
		Built environment, materials and equipment: Accessible records of daily environment cleaning (% of countries)	quantitative	Environment	Reporting
37	Benchmarking national action plans on antimicrobial resistance in eight selected LMICs: Focus on the veterinary sector strategies	Is each proposed activity in each country NAP costed?	Y/N	Multisector	Sustainability
		Is funding source in each country NAP indicated?	Y/N	Multisector	Sustainability
		Score for overall concordance with WHO GAP by country	quantitative	Multisector	Strategic Vision

		Population livestock holding (% households) by country	quantitative	Animal	Reporting
		Size of livestock (million) by country	quantitative	Animal	Reporting
		Concordance with WHO GAP by country: IPC	Y/N	Multisector	Strategic Vision
		Concordance with WHO GAP by country: Optimised use of antimicrobials	Y/N	Multisector	Strategic Vision
		Concordance with WHO GAP by country: Surveillance & research	Y/N	Multisector	Strategic Vision/ Research, Innovation, & Digital Technology
		Concordance with WHO GAP by country: Improve awareness & understanding of AMR	Y/N	Multisector	Strategic Vision
		Target for AMU reduction by country	Y/N	Animal	Stewardship
		Legislation restricting AMU for growth promotion or disease prevention in livestock, by country	Y/N	Animal	Regulations & Legislation
		AM quality assurance strategies by country	Y/N	Animal	Stewardship
		Operational plan quality by country	Y/N	Animal	Strategic Vision
		M&E plan quality by country	Y/N	Animal	Effectiveness
38	Capturing data on antimicrobial resistance patterns and trends in use in regions of Asia (CAPTURA)	Dissemination: Data analysis - report generation (facility and country-level)	qualitative	Multisector	Reporting
		Implementation: Data collection - capacity building activities in data management and analysis	qualitative	Multisector	Surveillance & Laboratory
		Dissemination: Data analysis - data analysis and interpretation	qualitative	Multisector	Surveillance & Laboratory
39	Carbapenem Resistance in Animal-Environment-Food from Africa: A Systematic Review, Recommendations and Perspectives	Carbapenemase-encoding gene families detected in Africa in animals, the environment, and foods	qualitative	Animal	Surveillance & Laboratory
		CRB's virulence genes reported in animals, the environment and foods in Africa by virulence factor	quantitative	Animal	Surveillance & Laboratory
40	Carbapenemase-producing Enterobacteriaceae in Europe:	Officially nominated national reference laboratory, or expert laboratory	qualitative	Multisector	Surveillance & Laboratory
		Epidemiological stage by country and CPE type	qualitative	Multisector	Surveillance & Laboratory

	assessment by national experts from 38 countries, May 2015	National recommendation or obligation for reporting (notification) to health authorities	qualitative	Multisector	Accountability
		National plan for containment of (or preparedness to contain) CPE	qualitative	Multisector	Prevention & Control
		National recommendation or guideline on infection control measures	qualitative	Multisector	Prevention & Control
		National capacity for surveillance and containment of CPE by country	qualitative	Multisector	Surveillance & Laboratory
		National system for surveillance	qualitative	Multisector	Surveillance & Laboratory
41	Clinical management of severe infections caused by carbapenem-resistant gram-negative bacteria: a worldwide cross-sectional survey addressing the use of antibiotic combinations	Prevalence of CR Acinetobacter spp., Klebsiella pneumoniae and Pseudomonas spp. in invasive isolates reported by respondents and clustered by country	quantitative	Human	Surveillance & Laboratory
		Availability of antibiotic agents by country, clustered by income group	quantitative	Human	Access to Medicines & Health Services
42	Clinical outcomes and bacterial characteristics of carbapenem-resistant Klebsiella pneumoniae complex among patients from different global regions (CRACKLE-2): a prospective, multicentre, cohort study	Bacterial population structure by country	quantitative	Human	Surveillance & Laboratory
43	Clinical practice guidelines for acute otitis media in children: a systematic review and appraisal of European national guidelines	Do the above guidelines provide diagnostic criteria?	Y/N	Human	Stewardship
		Do the guidelines specify when to initiate antibiotics?	Y/N	Human	Stewardship
		Do the guidelines specify the route of antibiotic administration?	Y/N	Human	Stewardship
		Do the guidelines specify duration of antibiotic regimens?	Y/N	Human	Stewardship
		Existence of national guideline by country	Y/N	Human	Stewardship
		Intiation, choice, duration and strength of recommendation for routine 1st-line antibiotics by country	qualitative	Human	Stewardship
		Aimed audience of AOM guidelines by country	qualitative	Human	Stewardship

		AMS & AOM guidelines: do guidelines provide diagnostic criteria?	Y/N	Human	Stewardship
		AMS & AOM guidelines: do guidelines specify duration of antibiotic regimens?	Y/N	Human	Stewardship
		AMS & AOM guidelines: do guidelines specify route of administration?	Y/N	Human	Stewardship
		AMS & AOM guidelines: do guidelines specify when to initiate antibiotics?	Y/N	Human	Stewardship
		AMS & AOM guidelines: % of antibiotic recommendations that refer to country-specific AMR patterns	quantitative	Human	Stewardship
		AMS & AOM guidelines: amoxicillin dosage that refers to country-specific AMR patterns	Y/N	Human	Stewardship
		AMS & AOM guidelines: amoxicillin-clavulanic acid dosage that refers to country-specific AMR patterns	Y/N	Human	Stewardship
44	Coming of age: governance challenges in updated AMR national action plans in the EU	NAP general structure: extent to which NAP mirrors GAP	3-point scale (no mention, some, extensively)	Multisector	Strategic Vision
45	Comparative Analysis of Outpatient Antibiotic Prescribing in Early Life: A Population-Based Study Across Birth Cohorts in Denmark and Germany	Most frequently prescribed outpatient antibiotics (%) first in early life for birth cohorts 2004 & 2016, by country	quantitative	Human	Stewardship
		Access % of outpatient antibiotics prescribed in first 2 yrs of life, by birth cohort and country	quantitative	Human	Stewardship
		Watch % of outpatient antibiotics prescribed in first 2 yrs of life, by birth cohort and country	quantitative	Human	Stewardship
		Median time (months) to first outpatient antibiotic prescription by birth cohort and country	quantitative	Human	Stewardship
		Rate of outpatient antibiotic treatment episodes per 1,000 person-years in the first & second year of life, by birth cohort and country	quantitative	Human	Stewardship
		n of outpatient antibiotic prescriptions per 1,000 person-years (first two years of life combined) by birth cohort and country	quantitative	Human	Stewardship

		Prescription prevalence of outpatient antibiotics per 100 children (first two years of life combined) by birth cohort and country	quantitative	Human	Stewardship
		n of DDDs of outpatient antibiotics per 1,000 children per day (first two years of life combined) by birth cohort and country	quantitative	Human	Stewardship
		Distribution of n of outpatient antibiotic treatment episodes per person in first 2 yrs of life, by birth cohort and country	quantitative	Human	Stewardship
46	Comparing Farm Biosecurity and Antimicrobial Use in High-Antimicrobial-Consuming Broiler and Pig Farms in the Belgian-Dutch Border Region	Biosecurity for pigs and broilers by country (internal and external biosecurity)	Ordinal (/100)	Animal	Prevention & Control
		AMU per production round for broilers by country (expressed as Treatment Incidence)	quantitative	Animal	Stewardship
		Proportion of each AM class prescribed by country for pigs and broilers	quantitative	Animal	Stewardship
		AMU by pig type by country (expressed as Treatment Incidence)	quantitative	Animal	Stewardship
47	Comparison of Antimicrobial Consumption Patterns in the Swiss and Danish Cattle and Swine Production (2007-2013)	Correlation coefficients between years (2007–2013) and antimicrobial consumption for each combination of species/country/antimicrobial class	quantitative	Animal	Stewardship
		AM consumption in mg/BM/y in cattle	quantitative	Animal	Stewardship
		AM consumption in mg/BM/y in pigs	quantitative	Animal	Stewardship
		AM consumption in mg/BM/y by antimicrobial class and livestock sector	quantitative	Animal	Stewardship
		Relative consumption of antimicrobial classes by country (%) and livestock sector	quantitative	Animal	Stewardship
48	Comparison of governance approaches for the control of antimicrobial resistance: Analysis of three European countries	How priorities are set for improving actions and standards by country: Who is involved & what is the role?	qualitative	Multisector	Participation
		How priorities are set for improving actions and standards by country: What is the evidence base for decision-making?	qualitative	Multisector	Strategic Vision

		How priorities are set for improving actions and standards by country: c.What are the main strengths	qualitative	Multisector	Strategic Vision
		How priorities are set for improving actions and standards by country: d.What are the main weaknesses?	qualitative	Multisector	Strategic Vision
		How is performance monitored by country: By whom?	qualitative	Multisector	Effectiveness
		How is accountability for performance ensured by country: How are the accountability mechanisms in place linked to the health system’s broader governance structures?	qualitative	Multisector	Accountability
		How is accountability for performance ensured by country: Are the mechanisms effective?	qualitative	Multisector	Accountability
		To what extent are the three components aligned by country?	qualitative	Multisector	Strategic Vision
		Drivers and mechanisms for change for revising future governance for AMR prevention, including new perspectives, objectives and suggested actions to achieve the former two	qualitative	Multisector	Strategic Vision
49	Comparison of national strategies to reduce meticillin-resistant Staphylococcus aureus infections in Japan and England	Infection rate (/100,000 hospitalised patients) for MRSA and total n of interventions per year, Japan	quantitative	Human	Surveillance & Laboratory
		Infection rate (/100,000 bed-day) for MRSA BSI and total n of interventions per year, England	quantitative	Human	Surveillance & Laboratory
		Infection rate (/100,000 hospitalised patients) for MRSA and intervention types (mandatory, recommendation, campaign), Japan	quantitative	Human	Surveillance & Laboratory
		Infection rate (/100,000 bed-day) for MRSA BSI and intervention types (mandatory, recommendation, campaign), England	quantitative	Human	Surveillance & Laboratory
		Infection rate (/100,000 hospitalised patients) for MRSA and nature of intervention (restrictive, persuasive, structural), Japan	quantitative	Human	Surveillance & Laboratory

	Infection rate (/100,000 bed-day) for MRSA BSI and nature of intervention (restrictive, persuasive, structural), England	quantitative	Human	Surveillance & Laboratory
	Infection rate (/100,000 hospitalised patients) for MRSA and level of intervention implementation (macro, meso, micro), Japan	quantitative	Human	Surveillance & Laboratory
	Infection rate (/100,000 bed-day) for MRSA BSI and level of intervention implementation (macro, meso, micro), England	quantitative	Human	Surveillance & Laboratory
	Infection rate (/100,000 hospitalised patients) for MRSA, Japan	quantitative	Human	Surveillance & Laboratory
	Infection rate (/100,000 bed-day) for E. coli BSI, England	quantitative	Human	Surveillance & Laboratory
	Infection rate (/100,000 bed-day) for CDI, England	quantitative	Human	Surveillance & Laboratory
	Infection rate (/100,000 hospitalised patients) for PRSP, Japan	quantitative	Human	Surveillance & Laboratory
	Infection rate (/100,000 hospitalised patients) for VRE, Japan	quantitative	Human	Surveillance & Laboratory
	Infection rate (/100,000 hospitalised patients) for MDRP, Japan	quantitative	Human	Surveillance & Laboratory
	Infection rate (/100,000 hospitalised patients) for MDRA, Japan	quantitative	Human	Surveillance & Laboratory
	Infection rate (/100,000 hospitalised patients) for CRE, Japan	quantitative	Human	Surveillance & Laboratory
	Infection rate (/100,000 hospitalised patients) for VRSA, Japan	quantitative	Human	Surveillance & Laboratory
	Infection rate (/100,000 bed-day) for MRSA BSI, England	quantitative	Human	Surveillance & Laboratory
	Infection rate (/100,000 bed-day) for MSSA BSI, England	quantitative	Human	Surveillance & Laboratory
50 Correlation between veterinary antimicrobial use and antimicrobial resistance in food-producing animals: a report on seven countries	Ampicillin-resistant E. coli (%) by animal species (poultry, veal calves, pigs, cattle)	quantitative	Animal	Surveillance & Laboratory
	Sulphonamides-resistant E. coli (%) by animal species (poultry, veal calves, pigs, cattle)	quantitative	Animal	Surveillance & Laboratory

		Ciprofloxacin-resistant E. coli (%) by animal species (poultry, veal calves, pigs, cattle)	quantitative	Animal	Surveillance & Laboratory
		Streptomycin-resistant E. coli (%) by animal species (poultry, veal calves, pigs, cattle)	quantitative	Animal	Surveillance & Laboratory
		Tetracycline-resistant E. coli (%) by animal species (poultry, veal calves, pigs, cattle)	quantitative	Animal	Surveillance & Laboratory
		Chloramphenicol-resistant E. coli (%) by animal species (poultry, veal calves, pigs, cattle)	quantitative	Animal	Surveillance & Laboratory
		Cefotaxime-resistant E. coli (%) by animal species (poultry, veal calves, pigs, cattle)	quantitative	Animal	Surveillance & Laboratory
		Gentamicin-resistant E. coli (%) by animal species (poultry, veal calves, pigs, cattle)	quantitative	Animal	Surveillance & Laboratory
		Tetracyclines use (mg/PCU) by country	quantitative	Animal	Stewardship
		Amphenicols use (mg/PCU) by country	quantitative	Animal	Stewardship
		Penicillins use (mg/PCU) by country	quantitative	Animal	Stewardship
		3G cephalosporins use (mg/PCU) by country	quantitative	Animal	Stewardship
		Sulphonamides use (mg/PCU) by country	quantitative	Animal	Stewardship
		Fluoroquinolones use (mg/PCU) by country	quantitative	Animal	Stewardship
		Aminoglycosides use (mg/PCU) by country	quantitative	Animal	Stewardship
51	Cost-Effectiveness of Test-and-Treat Strategies to Reduce the Antibiotic Prescription Rate for Acute Febrile Illness in Primary Healthcare Clinics in Africa	n of antibiotic regimens consumed by strategy and country	quantitative	Human	Stewardship
		Peak month for antibiotic consumption by country	qualitative	Human	Stewardship
		Cost (I$) of hourly salary (nurse, doctor, lab technician) by country	quantitative	Human	Workforce
		Cost (I$) of hospitalisation (1 inpatient day) by country	quantitative	Human	Access to Medicines & Healthcare Services
		Cost (I$) of training by country	quantitative	Human	Workforce
		Cost (I$) of tests by country	quantitative	Human	Access to Medicines & Healthcare Services
		Cost (I$) of time spent per patient (mins) by country	quantitative	Human	Access to Medicines & Healthcare Services

	Cost (I$) of standard-of-care (nurse, doctor, lab technician) by country	quantitative	Human	Workforce
	Cost (I$) of alternative strategies (mins) by country	quantitative	Human	Access to Medicines & Healthcare Services
	Cost (I$) of health effect (DALY infectious disease moderate, DALY infectious disease severe) by country	quantitative	Human	Effectiveness
	Costs (I$) per consultation per cost variable by country	quantitative	Human	Access to Medicines & Healthcare Services
	Cost (I$) of drugs by country	quantitative	Human	Access to Medicines & Healthcare Services
	Availability and coverage of antibiotics consumption audit by sector (retail, hospital) and country	qualitative	Human	Stewardship
	DALYs by strategy and country	quantitative	Human	Effectiveness
	DDDs by strategy and country	quantitative	Human	Stewardship
	Days-of-therapy by strategy and country	quantitative	Human	Stewardship
	ICER (pairwise to SoC, fully incremental) by strategy and country	quantitative	Human	Access to Medicines & Healthcare Services
	Incremental % point difference in antibiotic prescription rate against incremental cost (I$) by strategy and country	quantitative	Human	Access to Medicines & Healthcare Services
	Willingness-to-pay (I$) per consultation for 1 ppt reducton in antibiotic prescription rate against the probability it is cost-effective (%) by strategy and country	quantitative	Human	Access to Medicines & Healthcare Services
	Non-respiratory patient pop: antibiotic prescription rate (%) by strategy and country	quantitative	Human	Stewardship
	Non-respiratory patient pop: cost (I$) by strategy and country	quantitative	Human	Access to Medicines & Healthcare Services
	Non-respiratory patient pop: ppt reduction in APR by strategy and country	quantitative	Human	Effectiveness
	Non-respiratory patient pop: cost difference compared to SoC (I$) by strategy and country	quantitative	Human	Access to Medicines & Healthcare Services

		Respiratory patient pop: antibiotic prescription rate (%) by strategy and country	quantitative	Human	Stewardship
		Respiratory patient pop: cost (I$) by strategy and country	quantitative	Human	Access to Medicines & Healthcare Services
		Respiratory patient pop: ppt reduction in APR by strategy and country	quantitative	Human	Effectiveness
		Respiratory patient pop: cost difference compared to SoC (I$) by strategy and country	quantitative	Human	Access to Medicines & Healthcare Services
		Positivity rates per test for non-pathogen-specific CRP by country	quantitative	Human	Surveillance & Laboratory
		Positivity rates per test for streptococcus pneumoniae by country	quantitative	Human	Surveillance & Laboratory
		Positivity rates per test for typhoid fever by country	quantitative	Human	Surveillance & Laboratory
		Positivity rates per test for urine nitrite by country	quantitative	Human	Surveillance & Laboratory
		Positivity rates per test for urine esterase by country	quantitative	Human	Surveillance & Laboratory
		Positivity rates per test for CBC by country	quantitative	Human	Surveillance & Laboratory
		Positivity rates per test for WBC ≥ 11,000 by country	quantitative	Human	Surveillance & Laboratory
		Positivity rates per test for Neutrophils ≥ 75% by country	quantitative	Human	Surveillance & Laboratory
		Positivity rates per test for group A streptococcus by country	quantitative	Human	Surveillance & Laboratory
52	Determinants of worldwide antibiotic resistance dynamics across drug-bacterium pairs: a multivariable spatial-temporal analysis using ATLAS	n of isolates by bacterial species from sputum & medicine general	quantitative	Human	Surveillance & Laboratory
		n of isolates by bacterial species from sputum & emergency room	quantitative	Human	Surveillance & Laboratory
		n of isolates by bacterial species from sputum & surgery general	quantitative	Human	Surveillance & Laboratory
		n of isolates by bacterial species from sputum & medicine ICU	quantitative	Human	Surveillance & Laboratory
		n of isolates by bacterial species from blood & medicine general	quantitative	Human	Surveillance & Laboratory

	n of isolates by bacterial species from blood & emergency room	quantitative	Human	Surveillance & Laboratory
	n of isolates by bacterial species from blood & surgery general	quantitative	Human	Surveillance & Laboratory
	n of isolates by bacterial species from blood & medicine ICU	quantitative	Human	Surveillance & Laboratory
	n of isolates by bacterial species from abscess & medicine general	quantitative	Human	Surveillance & Laboratory
	n of isolates by bacterial species from abscess & emergency room	quantitative	Human	Surveillance & Laboratory
	n of isolates by bacterial species from abscess & surgery general	quantitative	Human	Surveillance & Laboratory
	n of isolates by bacterial species from abscess & medicine ICU	quantitative	Human	Surveillance & Laboratory
	n of isolates by bacterial species from urine & medicine general	quantitative	Human	Surveillance & Laboratory
	n of isolates by bacterial species from urine & emergency room	quantitative	Human	Surveillance & Laboratory
	n of isolates by bacterial species from urine & surgery general	quantitative	Human	Surveillance & Laboratory
	n of isolates by bacterial species from urine & medicine ICU	quantitative	Human	Surveillance & Laboratory
	n of isolates by bacterial species from wound & medicine general	quantitative	Human	Surveillance & Laboratory
	n of isolates by bacterial species from wound & emergency room	quantitative	Human	Surveillance & Laboratory
	n of isolates by bacterial species from wound & surgery general	quantitative	Human	Surveillance & Laboratory
	n of isolates by bacterial species from wound & medicine ICU	quantitative	Human	Surveillance & Laboratory
	Distribution of ABR rates across countries by infection source (all, blood, sputum, urine) & drug-bacterium pair	quantitative	Human	Surveillance & Laboratory
	Aminopenicillin-resistant E. coli resistance rate (%) by country	quantitative	Human	Surveillance & Laboratory

		Carbapenem-resistant A. baumannii resistance rate (%) by country	quantitative	Human	Surveillance & Laboratory
		n of isolates by infection sources and countries	quantitative	Human	Reporting
53	Development of a cross-sectoral antimicrobial resistance capability assessment framework	National assessment: national coordination of AMR	qualitative	Multisector	Coordination
		National assessment: national AMR coordinating committee	qualitative	Multisector	Coordination
		National assessment: stakeholders in AMR	qualitative	Multisector	Participation
		National assessment: intersectoral coordination	qualitative	Multisector	Coordination
		National assessment: AMR policy	qualitative	Multisector	Regulations & Legislation
		National assessment: regulation of the supply of antimicrobials	qualitative	Multisector	Regulations & Legislation
		National assessment: AMS	qualitative	Multisector	Stewardship
		AMS assessment: facility characteristics	qualitative	Multisector	Stewardship
		AMS assessment: education and guidelines	qualitative	Multisector	Stewardship/Workforce
		AMS assessment: governance and leadership	qualitative	Multisector	Stewardship
		AMS assessment: AMS and antimicrobial prescribing processes	qualitative	Multisector	Stewardship
		AMS assessment: clinical patient review	qualitative	Multisector	Stewardship
		AMS assessment: monitoring antimicrobial prescribing, use and resistance	qualitative	Multisector	Stewardship
		AMS assessment: reporting and feedback	qualitative	Multisector	Stewardship
		National assessment: AMR research	qualitative	Multisector	Research, Innovation, & Digital Technology
		National assessment: use of surveillance data	qualitative	Multisector	Feedback Mechanisms
		National assessment: antimicrobial surveillance in animal health/agriculture settings	qualitative	Environment	Surveillance & Laboratory
		National assessment: organisation of animal health laboratory network	qualitative	Animal	Surveillance & Laboratory/ Coordination
		National assessment: antimicrobial surveillance in human health		Human	Surveillance & Laboratory

	National assessment: major AMR activities and issues		Multisector	Strategic Vision
	National assessment: programme planning		Multisector	Strategic Vision
	IPC WASH assessment: IPC programme	qualitative	Human	Prevention & Control
	IPC WASH assessment: IPC guidelines	qualitative	Human	Prevention & Control
	IPC WASH assessment: IPC education and training	qualitative	Human	Prevention & Control
	IPC WASH assessment: HAI surveillance	qualitative	Human	Prevention & Control
	IPC WASH assessment: multimodal strategies for implemenation of IPC interventions	qualitative	Human	Prevention & Control
	IPC WASH assessment: monitoring/audit of IPC practices and feedback	qualitative	Human	Prevention & Control
	IPC WASH assessment: workload, staffing and bed occupancy	qualitative	Human	Prevention & Control/Workforce
	IPC WASH assessment: built environment, material and equipment for IPC at the facility level	qualitative	Human	Prevention & Control
	Human health lab assessment: AST	qualitative	Human	Surveillance & Laboratory
	Human health lab assessment: pathogens vs antibiotic resistance combination	qualitative	Human	Surveillance & Laboratory
	Human health lab assessment: AMR diagnostic capabilities	qualitative	Human	Surveillance & Laboratory
	Human health lab assessment: reporting	qualitative	Human	Surveillance & Laboratory/Reporting
	Human health lab assessment: referral pathways and sample transport	qualitative	Human	Surveillance & Laboratory
	Human health lab assessment: data collection and management	qualitative	Human	Surveillance & Laboratory
	Human health lab assessment: operations and infrastructure	qualitative	Human	Surveillance & Laboratory
	Human health lab assessment: biosafety and biosecurity	qualitative	Human	Surveillance & Laboratory
	Human health lab assessment: quality management system and quality assurance	qualitative	Human	Surveillance & Laboratory

		Animal health lab assessment: AST	qualitative	Animal	Surveillance & Laboratory
		Animal health lab assessment: quality management system and quality assurance	qualitative	Animal	Surveillance & Laboratory
		Animal health lab assessment: bacteria vs antibiotic combinations for AST	qualitative	Animal	Surveillance & Laboratory
		Animal health lab assessment: equipment	qualitative	Animal	Surveillance & Laboratory
		Animal health lab assessment: bacteriology sample details	qualitative	Animal	Surveillance & Laboratory
		Animal health lab assessment: referral pathways and sample transport	qualitative	Animal	Surveillance & Laboratory
		Animal health lab assessment: data collection and management	qualitative	Animal	Surveillance & Laboratory
		Animal health lab assessment: operations and infrastructure	qualitative	Animal	Surveillance & Laboratory
		Animal health lab assessment: biosafety and biosecurity	qualitative	Animal	Surveillance & Laboratory
		National assessment: livestock industry and production	qualitative	Animal	Prevention & Control
		Animal health lab assessment: bacteria the lab is able to grow and identify	qualitative	Animal	Surveillance & Laboratory
		Animal health lab assessment: reporting	qualitative	Animal	Reporting
54	Differences in epidemiology of candidaemia in the Nordic countries - what is to blame?	Consumption (DID) by country: Metronidazole	quantitative	Human	Stewardship
		Fluconazole-resistance rate (n,%) by country: C. albicans	quantitative	Human	Surveillance & Laboratory
		Fluconazole-resistance rate (n,%) by country: C. glabrata	quantitative	Human	Surveillance & Laboratory
		Fluconazole-resistance rate (n,%) by country: C. tropicalis	quantitative	Human	Surveillance & Laboratory
		Fluconazole-resistance rate (n,%) by country: C. parapsilosis	quantitative	Human	Surveillance & Laboratory
		Anidulafungin-resistance rate (n,%) by country: C. albicans	quantitative	Human	Surveillance & Laboratory
		Anidulafungin-resistance rate (n,%) by country: C. glabrata	quantitative	Human	Surveillance & Laboratory

	Anidulafungin-resistance rate (n,%) by country: C. tropicalis	quantitative	Human	Surveillance & Laboratory
	Anidulafungin-resistance rate (n,%) by country: C. parapsilosis	quantitative	Human	Surveillance & Laboratory
	ICU+IMCU beds (/100,000 inhabitants) by country	quantitative	Human	Access to Medicines & Health Services
	Consumption (DID) by country: Total antibacterial consumption	quantitative	Human	Stewardship
	Consumption (DID) by country: Tetracyclines	quantitative	Human	Stewardship
	Consumption (DID) by country: Penicillins	quantitative	Human	Stewardship
	Consumption (DID) by country: 1st, 2nd, 3G cephalosporins	quantitative	Human	Stewardship
	Consumption (DID) by country: Aminoglycosides	quantitative	Human	Stewardship
	Consumption (DID) by country: Trimethoprim and sulphonamides	quantitative	Human	Stewardship
	Consumption (DID) by country: Piperacillin-tazobactam	quantitative	Human	Stewardship
	Consumption (DID) by country: Vancomycin	quantitative	Human	Stewardship
	Consumption (DID) by country: Ciprofloxacin	quantitative	Human	Stewardship
	Consumption (DID) by country: Carbapenems	quantitative	Human	Stewardship
	Consumption (DID) by country: Colistin	quantitative	Human	Stewardship
	Consumption (DID) by country: Clindamycin	quantitative	Human	Stewardship
	DID of antifungal drugs for systemic use (/100,000 inhabitants) by country)	quantitative	Human	Stewardship
	Use of systemic antifungals, fluconazole in primary care and hospital care sector by country	quantitative	Human	Stewardship
	Candidaemia incidence (/100,000 inhabitants) by country	quantitative	Human	Surveillance & Laboratory
	Species distribution (n, %) by country: C. albicans	quantitative	Human	Surveillance & Laboratory
	Species distribution (n, %) by country: C. glabrata	quantitative	Human	Surveillance & Laboratory

		Species distribution (n, %) by country: C. tropicalis	quantitative	Human	Surveillance & Laboratory
		Species distribution (n, %) by country: C. parapsilosis	quantitative	Human	Surveillance & Laboratory
55	Different microbial and resistance patterns in primary total knee arthroplasty infections - a report on 283 patients from Lithuania and Sweden	Antibiotic susceptibility pattern of selected antibiotics by country	quantitative	Human	Surveillance & Laboratory
		n of pathogens tested for and susceptibility to selected antimicrobial agents in patients with PJI by country	quantitative	Human	Surveillance & Laboratory
		Pathogens found in PJIs (n, %) by country	quantitative	Human	Surveillance & Laboratory
		Pathogens found in early, delayed, late & acute haematogenous infections by country	quantitative	Human	Surveillance & Laboratory
		Distribution of pathogens in polymicrobial infections by country	quantitative	Human	Surveillance & Laboratory
		Distribution of bacterial combinations in polymicrobial infections	quantitative	Human	Surveillance & Laboratory
56	Does gonorrhoea screening intensity play a role in the early selection of antimicrobial resistance in men who have sex with men (MSM)? a comparative study of Belgium and the United Kingdom	MIC distributions for MSM and women by country (azithromycin)	quantitative	Human	Surveillance & Laboratory
		MIC distributions for MSM and women by country (cefixime)	quantitative	Human	Surveillance & Laboratory
		MIC distributions for MSM and women by country (ceftriaxone)	quantitative	Human	Surveillance & Laboratory
57	Effective stakeholder engagement for collation, analysis and expansion of antimicrobial resistance (AMR) data: a CAPTURA experience	n of culture positive and culture negative test results reported by country	quantitative	Multisector	Reporting
		Proportion of 5 most commonly reported microorganisms by country	quantitative	Multisector	Reporting
		Data completeness (%) by country (age, microorganism type, id number, sex, specimen type and date, location, location type, reporting microorganisms to species level)	quantitative	Multisector	Surveillance & Laboratory
		n of public, private & public-private facilities sharing AST data by country	quantitative	Multisector	Surveillance & Laboratory
58	EPI-Net One Health reporting guideline for antimicrobial consumption and	One Health approach for AMC and residue surveillance reporting: inclusion of information on falsified and substandard antimicrobials	qualitative	Multisector	Reporting

	resistance surveillance data: a Delphi approach	One Health approach for AMC and residue surveillance reporting: inclusion of AMC and antimicrobial residues data	qualitative	Multisector	Reporting
		One Health approach for AMC and residue surveillance reporting: target antimicrobials for reporting	qualitative	Multisector	Reporting
		One Health approach for AMC and residue surveillance reporting: core elements for data reporting	qualitative	Multisector	Reporting
		One Health approach for AMR surveillance reporting: inclusion of AMR data	qualitative	Multisector	Reporting
		One Health approach for AMR surveillance reporting: target bacteria-drug combination for reporting	qualitative	Multisector	Reporting
		One Health approach for AMR surveillance reporting: core elements for data reporting	qualitative	Multisector	Reporting
		One Health approach for common definitions and strategies within surveillance reporting: inclusion of private (industry-funded) sector surveillance data	qualitative	Multisector	Reporting
		One Health approach for common definitions and strategies within surveillance reporting: frequency of reporting	qualitative	Multisector	Reporting
		AMC (animal) core element: sales or prescriptions	qualitative	Animal	Stewardship
59	Essential and forgotten antibiotics: an inventory in low- and middle-income countries	n of countries with WHO-EML 'Access' antibiotics approval	quantitative	Human	Access to Medicines & Health Services
		n of countries with WHO-EML 'Forgotten' antibiotics approval	quantitative	Human	Access to Medicines & Health Services
		n of countries with WHO-EML 'Access' & 'Forgotten' antibiotics approval	quantitative	Human	Access to Medicines & Health Services
		n of WHO-EML ‘Access’ & ‘Forgotten’ antibiotics approved by country	quantitative	Human	Access to Medicines & Health Services
		n of countries with WHO-EML 'Access' antibiotics approval intended for paediatric use	quantitative	Human	Access to Medicines & Health Services

		n of countries with WHO-EML 'Access' & 'Forgotten' antibiotics approval intended for paediatric use	quantitative	Human	Access to Medicines & Health Services
		n of countries with WHO-EML 'Forgotten' antibiotics approval intended for paediatric use	quantitative	Human	Access to Medicines & Health Services
		n of formulations intended specifically for paediatric use approved by country	quantitative	Human	Access to Medicines & Health Services
60	Estimating global trends in total and childhood antibiotic consumption, 2011-2015	Total antimicrobial consumption (SU) (X10 -10) for all formulations (HIC, LMIC)	quantitative	Human	Stewardship
		Total antimicrobial consumption (SU) per person for all formulations(HIC, LMIC)	quantitative	Human	Stewardship
		Total antimicrobial consumption (SU) (X10 -10) for child-appropriate formulations (HIC, LMIC)	quantitative	Human	Stewardship
		Total antimicrobial consumption (SU) per child aged 0-4 for child-appropriate formulations(HIC, LMIC)	quantitative	Human	Stewardship
		Consumption per person of AWaRe and Unclassified antibiotics for all formulations (HIC, LMIC)	quantitative	Human	Stewardship
		Consumption per child of AWaRe and Unclassified antibiotics for child-appropriate formulations (HIC, LMIC)	quantitative	Human	Stewardship
		Compound annual growth rates in consumption of Access and Watch antibiotics for all formulations (HIC, LMIC)	quantitative	Human	Stewardship
		Compound annual growth rates in consumption of Access and Watch antibiotics for child-appropriate formulations (HIC, LMIC)	quantitative	Human	Stewardship
		Consumption (SU) of amoxicillin and amoxicillin with clavulanic acid per child aged 0-4 (HIC, LMIC)	quantitative	Human	Stewardship
		Compound annual growth rate (%) for all formulations (HIC, LMIC) of consumption per person	quantitative	Human	Stewardship

		Compound annual growth rate (%) for child-appropriate formulations (HIC, LMIC) of consumption per child	quantitative	Human	Stewardship
61	Estimating the subnational prevalence of antimicrobial resistant Salmonella enterica serovars Typhi and Paratyphi A infections in 75 endemic countries, 1990-2019: a modelling study	MDR prevalence in S Typhi isolates (%, for 1990,2000,2010,2019 at the administrative division level 1 resolution)	quantitative	Human	Surveillance & Laboratory
		Prevalence and total n of drug-resistant Salmonella enterica Typhi and Paratyphi A infections by GBD super-region (% and n, 1990 and 2019)	quantitative	Human	Surveillance & Laboratory
		n of MDR and non-MDR Salmonella enterica Typhi infections and prevalence of MDR S Typhi isolates (%) by GBD super-region and year	quantitative	Human	Surveillance & Laboratory
		FQNS prevalence in S Typhi (%, for 1990,2000,2010,2019 at the administrative division level 1 resolution)	quantitative	Human	Surveillance & Laboratory
		n of FQNS and fluoroquinolone susceptible Salmonella enterica Typhi infections, and prevalence of FQNS S Typhi isolates (%) by GBD super-region and year	quantitative	Human	Surveillance & Laboratory
		Prevalence of MDR in S Paratyphi A (%, for 1990,2000,2010,2019 at the administrative division level 1 resolution)	quantitative	Human	Surveillance & Laboratory
62	European antibiotic awareness day: a five-year perspective of Europe-wide actions to promote prudent use of antibiotics	Countries participating in European Antibiotic Awareness Day	quantitative (n of countries)	Human	Participation/ Community Awareness & Enabling Behaviours
		n of national activities/year by country: Training	quantitative	Human	Workforce
		n of national activities/year by country: Distribution of brochures or other materials	quantitative	Human	Community Awareness & Enabling Behaviours
		n of national activities/year by country: Mailing	quantitative	Human	Community Awareness & Enabling Behaviours
		n of national activities/year by country: Communications on treatment recommendations	quantitative	Human	Community Awareness & Enabling Behaviours
		n of national activities/year by country: Advertisements	quantitative	Human	Community Awareness & Enabling Behaviours

	n of national activities/year by country: Press conference	quantitative	Human	Community Awareness & Enabling Behaviours
	n of national activities/year by country: Press release	quantitative	Human	Community Awareness & Enabling Behaviours
	n of national activities/year by country: PR activities	quantitative	Human	Community Awareness & Enabling Behaviours
	n of national activities/year by country: Activities targeting schools	quantitative	Human	Community Awareness & Enabling Behaviours
	n of national activities/year by country: Exhibition	quantitative	Human	Community Awareness & Enabling Behaviours
	n of national activities/year by country: Gimmicks	quantitative	Human	Community Awareness & Enabling Behaviours
	n of national activities/year by country: Other	quantitative	Human	Community Awareness & Enabling Behaviours
	n of campaign materials/year by country: Factsheet	quantitative	Human	Community Awareness & Enabling Behaviours
	n of campaign materials/year by country: Checklist	quantitative	Human	Community Awareness & Enabling Behaviours
	n of campaign materials/year by country: Advertorial	quantitative	Human	Community Awareness & Enabling Behaviours
	n of campaign materials/year by country: Online banner	quantitative	Human	Community Awareness & Enabling Behaviours
	n of campaign materials/year by country: Presentation	quantitative	Human	Community Awareness & Enabling Behaviours
	n of campaign materials/year by country: Screensaver	quantitative	Human	Community Awareness & Enabling Behaviours
	n of campaign materials/year by country: Gimmicks	quantitative	Human	Community Awareness & Enabling Behaviours
	n of campaign materials/year by country: Posters	quantitative	Human	Community Awareness & Enabling Behaviours
	n of campaign materials/year by country: Leaflets	quantitative	Human	Community Awareness & Enabling Behaviours
	n of campaign materials/year by country: Patient dialogues	quantitative	Human	Community Awareness & Enabling Behaviours

n of campaign materials/year by country: Letters to stakeholders	quantitative	Human	Community Awareness & Enabling Behaviours
n of campaign materials/year by country: Web-based materials	quantitative	Human	Community Awareness & Enabling Behaviours
n of campaign materials/year by country: Brochures	quantitative	Human	Community Awareness & Enabling Behaviours
n of campaign materials/year by country: Television or web spots	quantitative	Human	Community Awareness & Enabling Behaviours
n of campaign materials/year by country: Advertisements in print media	quantitative	Human	Community Awareness & Enabling Behaviours
n of government support activities/year by country: Political support	quantitative	Human	Strategic Vision/ Community Awareness & Enabling Behaviours
n of government support activities/year by country: Financial support	quantitative	Human	Sustainability/ Community Awareness & Enabling Behaviours
n of stakeholder support activities/year by country, from: Health professionals	quantitative	Human	Participation/ Community Awareness & Enabling Behaviours
n of stakeholder support activities/year by country, from: Pharmacies	quantitative	Human	Participation/ Community Awareness & Enabling Behaviours
n of stakeholder support activities/year by country, from: Patient groups	quantitative	Human	Participation/ Community Awareness & Enabling Behaviours
n of stakeholder support activities/year by country, from: NGOs	quantitative	Human	Participation/ Community Awareness & Enabling Behaviours
n of stakeholder support activities/year by country, from: Non-pharmaceutical companies	quantitative	Human	Participation/ Community Awareness & Enabling Behaviours
n of stakeholder support activities/year by country, from: Pharmaceutical companies	quantitative	Human	Participation/ Community Awareness & Enabling Behaviours
n of stakeholder support activities/year by country, from: Professional societies	quantitative	Human	Participation/ Community Awareness & Enabling Behaviours
n of stakeholder support activities/year by country, from: Insurance system	quantitative	Human	Participation/ Community Awareness & Enabling Behaviours
n of stakeholder support activities/year by country, from: WHO EUR/WHO country office	quantitative	Human	Participation/ Community Awareness & Enabling Behaviours
n of national activities/year by country: Scientific/professional conference	quantitative	Human	Research, Innovation, & Digital Technology/Workforce

		n of national activities/year by country: Publication of articles in medical journals	quantitative	Human	Research, Innovation, & Digital Technology
63	European survey on the current surveillance practices, management guidelines, treatment pathways and heterogeneity of testing of Clostridioides difficile, 2018-2019: results from the Combatting Bacterial Resistance in Europe CDI (COMBACTE)	Single assays used for CDI testing (n, %) by country	quantitative	Human	Surveillance & Laboratory
		At least 1 toxin detection assay used for CDI testing (n, %) by country	quantitative	Human	Surveillance & Laboratory
		ESCMID-recommended assays used for CDI testing (n, %) by country	quantitative	Human	Surveillance & Laboratory
		Adherence to contact isolation precautions (% of hospitals) for confirmed CDI patients, by country	quantitative	Human	Prevention & Control
		Adherence to contact isolation precautions (% of hospitals) for suspected CDI patients, by country	quantitative	Human	Prevention & Control
		Participation of hospital sites in international CDI surveillance (n, %) by country	quantitative	Human	Participation
		Participation of hospital sites in national CDI surveillance (n, %) by country	quantitative	Human	Surveillance & Laboratory
		Median testing frequency for CDI (/10,000 bed-days) by country	quantitative	Human	Surveillance & Laboratory
		Median CDI cases (/10,000 bed-days) by country	quantitative	Human	Surveillance & Laboratory
		Hospital-diagnosed CDI (n) included in national surveillance by hospitals, by country	quantitative	Human	Prevention & Control
		Hospital-acquired CDI (n) included in national surveillance by hospitals, by country	quantitative	Human	Prevention & Control
		Outpatient CDI (n) included in national surveillance by hospitals, by country	quantitative	Human	Prevention & Control
		Hospital sites' awareness of national CDI testing guidelines (n, %) by country	quantitative	Human	Prevention & Control
		Hospital sites' awareness of European CDI treatment guidelines (n, %) by country	quantitative	Human	Prevention & Control
		CDI in LCTF (n) included in national surveillance by hospitals, by country	quantitative	Human	Prevention & Control
		CDI in residual care (n) included in national surveillance by hospitals, by country	quantitative	Human	Prevention & Control

		CDI in community (n) included in national surveillance by hospitals, by country	quantitative	Human	Prevention & Control
		Notification of CDI cases from hospital sites (n, %) by country	quantitative	Human	Prevention & Control
64	European-wide antimicrobial resistance monitoring in commensal Escherichia coli isolated from healthy food animals between 2004 and 2018	Non-wild type and clinical resistance to both ciprofloxacin and cefotaxime based on ECOFFs and clinical breakpoints by animal group	quantitative	Animal	Surveillance & Laboratory
		Antimicrobial susceptibility of E. coli isolates by antimicrobial (n=15), by animal type (cattle, pigs, broilers) and by year group (the 4 stated in 'DATA SOURCE/S' column);	quantitative	Animal	Surveillance & Laboratory
		Proportions of multi-drug resistance of E. coli isolates of cattle, pigs and broilers by resistance phenotype	quantitative	Animal	Surveillance & Laboratory
		Occurrence of ESBL- and/or AmpC-producing E. coli in food animals	quantitative	Animal	Surveillance & Laboratory
		AMR % of E. coli by antimicrobial, by animal and by year group	quantitative	Animal	Surveillance & Laboratory
65	Evaluating the contribution of antimicrobial use in farmed animals to global antimicrobial resistance in humans	AMR in humans (%) from E. coli and S. aureus 2010-2020	quantitative	Human	Surveillance & Laboratory
		AMU in humans (DDD)	quantitative	Human	Stewardship
		Health expenditure (% of GDP)	quantitative	Multisector	Access to Medicines & Health Services
		WASH adherence	Binary	Environment	Prevention & Control/ Social Determinants
		AMU in farmed animals (tons)	quantitative	Animal	Stewardship
66	Evaluating the yaws diagnostic gap: a survey to determine the capacity of and barriers to improving diagnostics in all yaws-endemic countries	Serological testing capacity by country	qualitative	Multisector	Surveillance & Laboratory
		Molecular testing capacity by country	qualitative	Multisector	Surveillance & Laboratory
67	Exploring the antimicrobial stewardship educational needs of healthcare students and the potential of	Students' participation and means thereof in public health campaigns on prudent antibiotic use (n and %)	quantitative	Multisector	Community Awareness & Enabling Behaviours

	an antimicrobial prescribing app as an educational tool in selected African countries	Teaching methods used to teach students about prudent use of antibiotics (Y/N/UNSURE n and %)	quantitative	Multisector	Community Awareness & Enabling Behaviours
		Students' perspectives on AMR & AMS (n and %)	Likert scale	Multisector	Community Awareness & Enabling Behaviours
		Topics students would like to receive more info about (%)	quantitative	Multisector	Community Awareness & Enabling Behaviours
		Educational tools that would be part of their regular learning (if available) (%)	quantitative	Multisector	Community Awareness & Enabling Behaviours
68	Extended spectrum beta -lactamase producers among nosocomial Enterobacteriaceae in WHO AMR (Latin America)	Frequency of ESBL producers among E. coli & K. pneumoniae isolates in SMART and TEST AM surveillance programmes (% of isolates producing ESBLs by global region and programme year)	quantitative	Human	Surveillance & Laboratory
		Temporal prevalences of healthcare-acquired ESBL-positive E. coli & Klebsiella spp. (by surveillance programme, collection period, anatomical collection site and % of total n of isolates)	quantitative	Human	Surveillance & Laboratory
		Frequency of ESBL producers among E. coli & K. pneumoniae isolates in L.American hospitals (% by country and programme)	quantitative	Human	Surveillance & Laboratory
		Proportion of healthcare-acquired Enterobacteriaceae isolates susceptible to 3G cephalosporins and cefepime in L.American hospitals (by pathogen, country and programme)	quantitative	Human	Surveillance & Laboratory
69	First assessment of the knowledge, attitudes, and practices of health actors in Togo and Ivory Coast in regard to antibiotic resistance	Perception of health actors on antibiotic resistance by occupation and country	Likert scale	Human	Workforce
		Practices of health actors in relation to antibiotic resistance by occupation and country	Y/N	Human	Workforce
		Knowledge of health actors on antibiotic resistance by country	Likert scale	Human	Workforce
		Perception of the fight against antibiotic resistance by health actors, by occupation and country	Y/N; Likert scale	Human	Workforce

		Stakeholder scores on knowledge, Perceptions, and practices on antibiotics and antibiotic resistance by occupation and country	ordinal (0-11 for knowledge, 0-5 for perception, 0-4 for practice)	Multisector	Workforce
70	Five-year Pan-European, longitudinal surveillance of Clostridium difficile ribotype prevalence and antimicrobial resistance: the extended ClosER study	Proportions of the most prevalent ribotypes (n > 70) isolated over years 1–5 of the study, by gender, age group, source and previous CDI history	quantitative	Human	Surveillance & Laboratory
		Percentage prevalence (> 1%) of C. difficile PCR ribotypes in years 1–5 of the study	quantitative	Human	Surveillance & Laboratory
		Percentage prevalence by country of C. difficile PCR ribotypes in each year of the study	quantitative	Human	Surveillance & Laboratory
		Ribotype diversity by country during years 1–5 of the study	quantitative	Human	Surveillance & Laboratory
		Proportions of sensitive, intermediate and resistant C. difficile isolates from years 1–5 of the study	quantitative	Human	Surveillance & Laboratory
		MICs (mg/L) of prevalent C. difficile PCR ribotypes over Years 1–5 of the study	quantitative	Human	Surveillance & Laboratory
		Fidaxomicin MIC (mg/L) distributions for the most prevalent (n>30 isolates) ribotypes across all years of the study	quantitative	Human	Surveillance & Laboratory
		MICs of the 9 antimicrobials tested for all RT344 isolates	quantitative	Human	Surveillance & Laboratory
		Cumulative resistance score versus ribotype diversity score for each country, years 1–5 of the study	quantitative	Human	Surveillance & Laboratory
		Distribution of mean cumulative antimicrobial resistance scores for Clostridium difficile isolates across Europe in each year of the study	quantitative	Human	Surveillance & Laboratory
71	Genomic surveillance and antimicrobial resistance determinants in Neisseria	Antimicrobial susceptibility profiles (susceptible, susceptible increased exposure, resistant) of isolates (ceftriaxone) by country	quantitative	Multisector	Surveillance & Laboratory

	gonorrhoeae isolates from Uganda, Malawi and South Africa, 2015-20	Antimicrobial susceptibility profiles (susceptible, susceptible increased exposure, resistant) of isolates (cefixime) by country	quantitative	Multisector	Surveillance & Laboratory
		Antimicrobial susceptibility profiles (susceptible, susceptible increased exposure, resistant) of isolates (azithromycin) by country	quantitative	Multisector	Surveillance & Laboratory
		Antimicrobial susceptibility profiles (susceptible, susceptible increased exposure, resistant) of isolates (spectinomycin) by country	quantitative	Multisector	Surveillance & Laboratory
		Antimicrobial susceptibility profiles (susceptible, susceptible increased exposure, resistant) of isolates (ciprofloxacin) by country	quantitative	Multisector	Surveillance & Laboratory
		Antimicrobial susceptibility profiles (susceptible, susceptible increased exposure, resistant) of isolates (gentamicin MIC range mg/L) by country	quantitative	Multisector	Surveillance & Laboratory
		3 most common MLSTs STs (%) in isolates by country	quantitative	Multisector	Surveillance & Laboratory
		3 most common NG-STAR STs (%) in isolates by country	quantitative	Multisector	Surveillance & Laboratory
		3 most common NG-STAR CCs (%) in isolates by country	quantitative	Multisector	Surveillance & Laboratory
		10 most common NG-STAR CCs and their proportion in each country	quantitative	Multisector	Surveillance & Laboratory
		Country of origin, molecular typing schemes & phenotypic susceptibility and associated resistance determinants for different classes of antimicrobials	quantitative	Multisector	Surveillance & Laboratory
		AMR determinants (%) in isolates by country	quantitative	Multisector	Surveillance & Laboratory
72	Global antibiotic consumption 2000 to 2010: an analysis of national pharmaceutical sales data	Global antibiotic consumption by class	quantitative	Human	Stewardship
		Antibiotic consumption (standard units) per person, by country	quantitative	Human	Stewardship
		Compound annual growth rate (%) of antibiotic consumption by country	quantitative	Human	Stewardship

73	Global antibiotic consumption and usage in humans, 2000-18: a spatial modelling study	Hospital beds/1000 pop	quantitative	Human	Access to Medicines & Health Services
		Antibiotic consumption DDD/1000/day	quantitative	Human	Stewardship
		Travel time to nearest settlement	quantitative	Human	Access to Medicines & Health Services
		Ratio of dependants: working-age adults	quantitative	Human	Access to Medicines & Health Services
		y of maternal education	quantitative	Human	Access to Medicines & Health Services
		DPT3 vaccine coverage	quantitative	Human	Prevention & Control
74	Global Antimicrobial Resistance and Use Surveillance System on the African continent: Early implementation 2017-2019	n of patients with confirmed bacterial infection in bloodstream: Acinetobacter spp	quantitative	Human	Surveillance & Laboratory
		n of patients with confirmed bacterial infection in bloodstream: Escherichia coli	quantitative	Human	Surveillance & Laboratory
		n of patients with confirmed bacterial infection in bloodstream: Klebsiella pneumoniae	quantitative	Human	Surveillance & Laboratory
		n of patients with confirmed bacterial infection in bloodstream: Salmonella spp.	quantitative	Human	Surveillance & Laboratory
		n of patients with confirmed bacterial infection in bloodstream: Staphylococcus aureus	quantitative	Human	Surveillance & Laboratory
		n of patients with confirmed bacterial infection in bloodstream: Streptococcus pneumoniae	quantitative	Human	Surveillance & Laboratory
		n of patients with confirmed bacterial infection in bloodstream: Total	quantitative	Human	Surveillance & Laboratory
		n of patients with confirmed bacterial infection in urinary tract: Escherichia coli	quantitative	Human	Surveillance & Laboratory
		n of patients with confirmed bacterial infection in urinary tract: K. pneumoniae	quantitative	Human	Surveillance & Laboratory
		n of patients with confirmed bacterial infection in urinary tract: Total	quantitative	Human	Surveillance & Laboratory
		n of patients with confirmed bacterial infection in gastroenteric: Salmonella spp.	quantitative	Human	Surveillance & Laboratory
		n of patients with confirmed bacterial infection in gastroenteric: Shigella spp.	quantitative	Human	Surveillance & Laboratory

	n of patients with confirmed bacterial infection in gastroenteric: Total	quantitative	Human	Surveillance & Laboratory
	n of patients with confirmed bacterial infection in genital: Neisseria gonorrhoeae	quantitative	Human	Surveillance & Laboratory
	n of patients with confirmed bacterial infection in genital: Total	quantitative	Human	Surveillance & Laboratory
	n of patients with AST results in bloodstream: Acinetobacter spp	quantitative	Human	Surveillance & Laboratory
	n of patients with AST results in bloodstream: Escherichia coli	quantitative	Human	Surveillance & Laboratory
	n of patients with AST results in bloodstream: Klebsiella pneumoniae	quantitative	Human	Surveillance & Laboratory
	n of patients with AST results in bloodstream: Salmonella spp.	quantitative	Human	Surveillance & Laboratory
	n of patients with AST results in bloodstream: Staphylococcus aureus	quantitative	Human	Surveillance & Laboratory
	n of patients with AST results in bloodstream: Streptococcus pneumoniae	quantitative	Human	Surveillance & Laboratory
	n of patients with AST results in bloodstream: Total	quantitative	Human	Surveillance & Laboratory
	n of patients with AST results in urinary tract: Escherichia coli	quantitative	Human	Surveillance & Laboratory
	n of patients with AST results in urinary tract: K. pneumoniae	quantitative	Human	Surveillance & Laboratory
	n of patients with AST results in urinary tract: Total	quantitative	Human	Surveillance & Laboratory
	n of patients with AST results in gastroenteric: Salmonella spp.	quantitative	Human	Surveillance & Laboratory
	n of patients with AST results in gastroenteric: Shigella spp.	quantitative	Human	Surveillance & Laboratory
	n of patients with AST results in gastroenteric: Total	quantitative	Human	Surveillance & Laboratory
	n of patients with AST results in genital: Neisseria gonorrhoeae	quantitative	Human	Surveillance & Laboratory
	n of patients with AST results in genital: Total	quantitative	Human	Surveillance & Laboratory

		Countries reporting information on national surveillance system (n, %) per year	quantitative	Human	Reporting
		Countries reporting AMR data (n, %) per year	quantitative	Human	Reporting
		n of countries reporting AST results per year	quantitative	Human	Reporting
		Countries reporting age (n, %) per year	quantitative	Human	Reporting
		Countries reporting gender (n, %) per year	quantitative	Human	Reporting
		Countries reporting infection origin (n, %) per year	quantitative	Human	Reporting
		Countries reporting n of tested patients (n, %) per year	quantitative	Human	Reporting
75	Global antimicrobial resistance: a system-wide comprehensive investigation using the Global One Health Index	CR-AB rates by country	quantitative	Human	Surveillance & Laboratory
		VR-EF rates by country	quantitative	Human	Surveillance & Laboratory
		MRSA rates by country	quantitative	Human	Surveillance & Laboratory
		CR-KP rates by country	quantitative	Human	Surveillance & Laboratory
		CR-PA rates by country	quantitative	Human	Surveillance & Laboratory
		3GCR-EC rates by country	quantitative	Human	Surveillance & Laboratory
		MRSA positive rates by country	quantitative	Human	Surveillance & Laboratory
		Spatial distribution of MRSA by country	quantitative	Human	Surveillance & Laboratory
		CRKP positive rates by country	quantitative	Human	Surveillance & Laboratory
		Spatial distribution of CRKP by country	quantitative	Human	Surveillance & Laboratory
		GOHI-AMR scores for antimicrobial control & optimisation indicator by country	quantitative	Human	Stewardship
		GOHI-AMR scores for improve awareness & understanding indicator by country	quantitative	Human	Community Awareness & Enabling Behaviours
		GOHI-AMR scores for AMR rate for important antibiotics indicator by country	quantitative	Human	Surveillance & Laboratory
		GOHI-AMR scores for AMR surveillance system indicator by country	quantitative	Human	Surveillance & Laboratory
		GOHI-AMR scores for AMR lab network & coordination capacity indicator by country	quantitative	Multisector	Surveillance & Laboratory
76	Global approaches to tackling antimicrobial resistance: a	% of countries within WHO regions with enforceable wastewater disposal policy	quantitative	Environment	Regulations & Legislation

	comprehensive analysis of water, sanitation and hygiene policies	% of countries within WHO regions with enforceable medical waste disposal policy	quantitative	Environment	Regulations & Legislation
		Presence of water monitoring policy by country	Y/N	Environment	Regulations & Legislation
		Presence of sewerage policy by country	Y/N	Environment	Regulations & Legislation
		Presence of water quality policy by country	Y/N	Environment	Regulations & Legislation
		Presence of wastewater disposal policy by country	Y/N	Environment	Regulations & Legislation
		Presence of pollutant disposal policy by country	Y/N	Environment	Regulations & Legislation
		Presence of medical waste disposal policy by country	Y/N	Environment	Regulations & Legislation
		% of countries within WHO regions with enforceable water quality policy	quantitative	Environment	Regulations & Legislation
		% of countries within WHO regions with enforceable water monitoring policy	quantitative	Environment	Regulations & Legislation
		% of countries within WHO regions with enforceable sewerage policy	quantitative	Environment	Regulations & Legislation
		% of countries within WHO regions with enforceable pollutant disposal policy	quantitative	Environment	Regulations & Legislation
77	Global burden of bacterial antimicrobial resistance 1990-2021: a systematic analysis with forecasts to 2050	Deaths associated with and attributable to bacterial AMR (counts and all-age rates/100,000)	quantitative	Human	Surveillance & Laboratory
		DALYs associated with and attributable to bacterial AMR (counts and all-age rates/100,000)	quantitative	Human	Surveillance & Laboratory
78	Global diversity and antimicrobial resistance of typhoid fever pathogens: Insights from a meta-analysis of 13,000 Salmonella Typhi genomes	Annual genotype prevalence for countries with at least 50 genomes	quantitative	Multisector	Surveillance & Laboratory
		Genotype frequency by country (annual & aggregated)	quantitative	Multisector	Surveillance & Laboratory
		Prevalence of key AMR genotype profiles for countries with at least 50 genomes	quantitative	Multisector	Surveillance & Laboratory
		Annual prevalence of key AMR profiles for countries with ≥3 years with ≥10 representative genomes	quantitative	Multisector	Surveillance & Laboratory
		ESBL genes detected in Typhi genomes	quantitative	Multisector	Surveillance & Laboratory

		Temporal distribution of acrB mutants (genome count) by country	quantitative	Multisector	Surveillance & Laboratory
		Distribution of acrB mutants by genotype and mutation	quantitative	Multisector	Surveillance & Laboratory
79	Global incidence in hospital-associated infections resistant to antibiotics: An analysis of point prevalence surveys from 99 countries	Hospitalisations per year, as % of population, in countries with more than 5 million inhabitants	quantitative	Human	Access to Medicines & Health Services
		Hospital-associated antibiotic-resistant infections per 1,000 population per year in countries with at least 2 surveys reporting drug resistance proportions	quantitative	Human	Surveillance & Laboratory
		Hospital-associated antibiotic-resistant infections per year by income group and bacterial pathogen type	quantitative	Human	Surveillance & Laboratory
80	Global overview of national regulations for antibiotic use in aquaculture production	Are there limitations to the number of antibiotic treatments during each production cycle?	Y/N	Animal	Regulations & Legislation
		Is prophylactic use of antibiotics banned?	Y/N	Animal	Regulations & Legislation
		Can antibiotics only be used if prescribed by a veterinarian or fish health expert?	Y/N	Animal	Regulations & Legislation
		Is using antibiotics as growth enhancers banned?	Y/N	Animal	Regulations & Legislation
		Are highest-priority antibiotics prohibited?	Y/N	Animal	Regulations & Legislation
81	Global patterns and correlates in the emergence of antimicrobial resistance in humans	Mean predicted AMR emergence counts relative to ProMed mentions (log per cap)	quantitative	Multisector	Social Determinants
		Probability of zero AMR emergence reporting relative to GDP (log dollars per cap)	quantitative	Multisector	Social Determinants
		Probability of zero AMR emergence reporting relative to English spoken (yes/no)	quantitative	Multisector	Social Determinants
		Probability of zero AMR emergence reporting relative to publication bias index (log per cap)	quantitative	Multisector	Social Determinants
		Probability of zero AMR emergence reporting relative to ProMed mentions (log per cap)	quantitative	Multisector	Social Determinants
		Probability of zero AMR emergence reporting relative to population (log)	quantitative	Multisector	Social Determinants
		Mean predicted AMR emergence counts relative to tourism-inbound (log per cap)	quantitative	Multisector	Social Determinants

		Mean predicted AMR emergence counts relative to AB exports (log dollars per cap)	quantitative	Multisector	Social Determinants
		Mean predicted AMR emergence counts relative to health expenditure (% GDP)	quantitative	Multisector	Social Determinants
		Mean predicted AMR emergence counts relative to human AB consumption (DOD)	quantitative	Multisector	Stewardship
		Mean predicted AMR emergence counts relative to English spoken (yes/no)	quantitative	Multisector	Social Determinants
		Mean predicted AMR emergence counts relative to publication bias index (log per cap)	quantitative	Multisector	Social Determinants
		Reported and median predicted AMR emergence event counts by country	quantitative	Multisector	Surveillance & Laboratory
		Difference between predicted and reported AMR emergence event counts	quantitative	Multisector	Surveillance & Laboratory
		10 countries with the largest absolute difference between reported and median predicted counts (95th percentile predicted range in parentheses)	quantitative	Multisector	Surveillance & Laboratory
		Mean predicted AMR emergence counts relative to GDP (log dollars per cap)	quantitative	Multisector	Social Determinants
		Mean predicted AMR emergence counts relative to migrant population (log per cap)	quantitative	Multisector	Social Determinants
		Mean predicted AMR emergence counts relative to livestock AB consumption (log kg per cap)	quantitative	Animal	Surveillance & Laboratory
		Interaction between livestock antibiotic consumption and GDP	quantitative	Animal	Social Determinants/ Stewardship
82	Global research publications on irrational use of antimicrobials: call for more research to contain antimicrobial resistance	n of research publications on irrational AM use by decade	quantitative	Human	Research, Innovation, & Digital Technology
		n of countries contributing to research publications on irrational AM use per decade	quantitative	Human	Research, Innovation, & Digital Technology
		n of publications on irrational AM use & proportion of total for countries with at least 10 publications	quantitative	Human	Research, Innovation, & Digital Technology
83	Global trend of antimicrobial resistance in common bacterial pathogens in response to antibiotic consumption	Average NARCI value of target combinations for each country	quantitative	Human	Surveillance & Laboratory, and Stewardship
		DIDs for all penicillins	quantitative	Human	Stewardship

		DIDs for broad-spectrum penicillins	quantitative	Human	Stewardship
		DIDs for cephalosporins	quantitative	Human	Stewardship
		DIDs for fluoroquinolones	quantitative	Human	Stewardship
		DIDs for narrow-spectrum penicillins	quantitative	Human	Stewardship
		DIDs for carbapenems	quantitative	Human	Stewardship
		Resistance proportions (%) in MRSA	quantitative	Human	Surveillance & Laboratory
		Resistance proportions (%) in 3G cephalosporin-resistant E. coli	quantitative	Human	Surveillance & Laboratory
		Resistance proportions (%) in fluoroquinolone-resistant E. coli	quantitative	Human	Surveillance & Laboratory
		Resistance proportions (%) in 3G cephalosporin-resistant K. pneumoniae	quantitative	Human	Surveillance & Laboratory
		Resistance proportions (%) in carbapenem-resistant K. pneumoniae	quantitative	Human	Surveillance & Laboratory
		Resistance proportions (%) in penicillin-resistant S. pneumoniae	quantitative	Human	Surveillance & Laboratory
		Resistance proportions (%) in fluoroquinolone-resistant nontyphoidal Salmonella	quantitative	Human	Surveillance & Laboratory
84	Global trends in antimicrobial use in aquaculture	Antimicrobial consumption in aquaculture (2017, 2030) in tons	quantitative	Animal	Stewardship
		Projected AMU for pooled fish by country (top-5 consumers) in tons	quantitative	Animal	Stewardship
		Projected AMU for salmon by country (top-5 consumers) in tons	quantitative	Animal	Stewardship
		Projected AMU for shrimp by country (top-5 consumers) in tons	quantitative	Animal	Stewardship
		Projected AMU for tilapia by country (top-5 consumers) in tons	quantitative	Animal	Stewardship
		Projected AMU for trout by country (top-5 consumers) in tons	quantitative	Animal	Stewardship
		AMU by antimicrobial class (mg/kg)	quantitative	Animal	Stewardship
		AMU by active pharmaceutical ingredient (freq of use - n)	quantitative	Animal	Stewardship

		AMU (mg.kg-1) by aquaculture production system (intensive, intensive & semi-intensive, semi-intensive)	quantitative	Animal	Stewardship
		Projected AMU for catfish by country (top-5 consumers) in tons	quantitative	Animal	Stewardship
		Growth (%) in AMU by country	quantitative	Animal	Stewardship
		Annual sales by antimicrobial class (mg.kg-1)	quantitative	Animal	Stewardship
85	Global trends in antimicrobial use in food-producing animals: 2020 to 2030	Share of tetracycline in a country (%) vs AMU (mg/PCU)	quantitative	Animal	Stewardship
		Veterinary AMU (mg/PCU) by animal species (cattle, chicken, pigs, sheep)	quantitative	Animal	Stewardship
		Veterinary antimicrobial consumption (mg/PCU) & 2030 projection by country (top 10)	quantitative	Animal	Stewardship
		Veterinary antimicrobial consumption (mg/PCU) & 2030 projection by antimicrobial class (tetracyclines, amphenicols, penicillins, 1-4G cephalosporins, sulfonamides, macrolides, lincosamides, aminoglycosides, quinolones, pleuromutilins, polymyxins, other)	quantitative	Animal	Stewardship
		Antimicrobial consumption per country in 2020 & 2030	quantitative	Animal	Stewardship
86	Healthcare-associated infections in intensive care units in Taiwan, South Korea, and Japan: recent trends based on national surveillance reports	Mandated standard ratio of infection control personnel	quantitative	Human	Workforce
		Healthcare-associated infection data provided for: Causative pathogens	quantitative	Human	Surveillance & Laboratory
		Healthcare-associated infection data provided for: Antimicrobial-resistant pathogens	quantitative	Human	Surveillance & Laboratory
		n of isolates and resistance rate for MRSA	quantitative	Human	Surveillance & Laboratory
		n of isolates and resistance rate for Carbapenem-resistant Pseudomonas aeruginosa	quantitative	Human	Surveillance & Laboratory
		n of isolates and resistance rate for Carbapenem-resistant Acinetobacter baumannii	quantitative	Human	Surveillance & Laboratory

		Site-specific HAI (per 1000 patient-days) & Device-associated HAI (per 1000 device-days) across different geographic regions	quantitative	Human	Surveillance & Laboratory
		Type of healthcare-associated infection data provided for: Site-specific HAIs	qualitative	Human	Prevention & Control
		Type of healthcare-associated infection data provided for: Device-associated HAIs	qualitative	Human	Prevention & Control
		Incidence densities of healthcare-associated infections in ICUs by country and year (important landmarks related to surveillance, infection control and acts/legislation superimposed on the graph)	quantitative	Human	Surveillance & Laboratory
		Incidence density of ventilator-associated pneumonia by country and year	quantitative	Human	Surveillance & Laboratory
		Incidence density of central line-associated BSIs by country and year	quantitative	Human	Surveillance & Laboratory
		Incidence density of catheter-associated UTIs by country and year	quantitative	Human	Surveillance & Laboratory
		Common causative pathogens of HAIs by organism & proportion	quantitative	Human	Surveillance & Laboratory
		Year surveillance system established	quantitative	Multisector	Surveillance & Laboratory
		Demographics by country	mixed	Multisector	Social Determinants
87	How far has the globe gone in achieving One Health? Current evidence and policy implications based on global One Health index	GOHI - Government support & response (investment & financial support score): Research and development (R&D) expenses	quantitative	Human	Research, Innovation, & Digital Technology
		GOHI - Nutrition (food balance): Average dietary energy supply adequacy	quantitative	Human	Social Determinants
		GOHI - Nutrition (food balance): Average protein supply (g/cap/day) (3-year average)	quantitative	Human	Social Determinants
		GOHI - Nutrition (food balance): Per capita food supply variability (kcal/cap/day)	quantitative	Human	Social Determinants
		GOHI - Nutrition (nutrition promoting capacity): Nutrition labeling	quantitative	Human	Regulations & Legislation
		GOHI - Nutrition (nutrition promoting capacity): Nutrition guideline: an assessment of whether	quantitative	Human	Regulations & Legislation

	the government has a published nutrition guideline			
	GOHI - Nutrition (nutrition promoting capacity): Nutrition education programme	quantitative	Human	Community Awareness & Enabling Behaviours
	GOHI - Nutrition (nutrition score): Undernourishment	quantitative	Human	Social Determinants
	GOHI - Nutrition (nutrition score): Stunting in children under 5	quantitative	Human	Social Determinants
	GOHI - Nutrition (nutrition score): Anemia among women of reproductive age	quantitative	Human	Social Determinants
	GOHI - Nutrition (nutrition score): Ratio of overweight children	quantitative	Human	Social Determinants
	GOHI - Natural & social circumstances (food price indicators): Consumer Prices Food Indices	quantitative	Human	Social Determinants
	GOHI - Natural & social circumstances (food price indicators): Food price inflation	quantitative	Human	Equity
	GOHI - Food demand & supply (food demand score): Ratio of pop growth	quantitative	Human	Social Determinants
	GOHI - Food demand & supply (food demand score): Ratio of refugees and internally displaced people	quantitative	Human	Social Determinants
	GOHI - Food demand & supply (food demand score): Ratio of moderately or severely food insecure people	quantitative	Human	Social Determinants
	GOHI - Institutional system (justice): Unsentenced detainees	quantitative	Human	Social Determinants
	GOHI - Institutional system (justice): Property rights	quantitative	Human	Social Determinants
	GOHI - Institutional system (justice): Corruption perception index	quantitative	Human	Social Determinants
	GOHI - Institutional system (justice): Press freedom index	quantitative	Human	Social Determinants
	GOHI - Institutional system (justice): Affordability of justice	quantitative	Human	Social Determinants

GOHI - Institutional system (governance): Voice and accountability	quantitative	Human	Accountability
GOHI - Institutional system (governance): Gov spending	quantitative	Human	Social Determinants
GOHI - Institutional system (governance): Public social expenditure	quantitative	Human	Social Determinants
GOHI - Institutional system (governance): Public education expenditure	quantitative	Human	Social Determinants
GOHI - Institutional system (governance): Public health expenditure	quantitative	Human	Access to Medicines & Health Services
GOHI - Institutional system (governance): Political stability	quantitative	Human	Social Determinants
GOHI - Institutional system (governance): Gov effectiveness	quantitative	Human	Strategic Vision
GOHI - Institutional system (governance): Regulatory quality	quantitative	Human	Regulations & Legislation
GOHI - Institutional system (governance): Rule of law	quantitative	Human	Regulations & Legislation
GOHI - Institutional system (governance): Control of corruption	quantitative	Human	Regulations & Legislation
GOHI - Economical system (finance): GDP	quantitative	Human	Social Determinants
GOHI - Economical system (finance): GDP deflator	quantitative	Human	Social Determinants
GOHI - Economical system (finance): Revenue excluding grants	quantitative	Human	Social Determinants
GOHI - Economical system (finance): Grants and other revenue	quantitative	Human	Social Determinants
GOHI - Economical system (finance): Adjusted GDP growth	quantitative	Human	Social Determinants
GOHI - Economical system (work): Labour force participation	quantitative	Human	Social Determinants
GOHI - Economical system (work): Unemployment	quantitative	Human	Social Determinants
GOHI - Economical system (work): Annual working hours	quantitative	Human	Social Determinants

GOHI - Economical system (work): Youth condition	quantitative	Human	Social Determinants
GOHI - Economical system (housing): Own outright	quantitative	Human	Social Determinants
GOHI - Economical system (housing): Rent at reduced/subsidised price	quantitative	Human	Social Determinants
GOHI - Sociological system (demographic): Natural pop growth	quantitative	Human	Social Determinants
GOHI - Sociological system (demographic): Life expectancy	quantitative	Human	Social Determinants
GOHI - Sociological system (demographic): Child/infant mortality	quantitative	Human	Social Determinants
GOHI - Sociological system (demographic): Total fertility rate	quantitative	Human	Social Determinants
GOHI - Sociological system (demographic): Urbanisation	quantitative	Human	Social Determinants
GOHI - Sociological system (education): Education enrollment	quantitative	Human	Social Determinants
GOHI - Sociological system (education): Literacy	quantitative	Human	Social Determinants
GOHI - Sociological system (education): PISA score	quantitative	Human	Social Determinants
GOHI - Sociological system (education): Science performance	quantitative	Human	Social Determinants
GOHI - Sociological system (education): Higher education	quantitative	Human	Social Determinants
GOHI - Sociological system (education): Expenditure on research	quantitative	Human	Research, Innovation, & Digital Technology
GOHI - Sociological system (education): Female graduates	quantitative	Human	Equity
GOHI - Sociological system (education): Researchers population	quantitative	Human	Research, Innovation, & Digital Technology
GOHI - Sociological system (inequalities): Gini coefficient	quantitative	Human	Equity

	GOHI - Sociological system (inequalities): Palma ratio	quantitative	Human	Equity
	GOHI - Sociological system (inequalities): HDI	quantitative	Human	Equity
	GOHI - Sociological system (inequalities): Poverty rate	quantitative	Human	Equity
	GOHI - Sociological system (inequalities): Gender inequality index	quantitative	Human	Equity
	GOHI- Technological system (transport): Railway travel	quantitative	Human	Social Determinants
	GOHI- Technological system (transport): Air travel	quantitative	Human	Social Determinants
	GOHI- Technological system (tech adoption): Population using internet	quantitative	Human	Social Determinants
	GOHI- Technological system (tech adoption): Motor vehicle ownership	quantitative	Human	Social Determinants
	GOHI- Technological system (tech adoption): Mobile cellular subscriptions	quantitative	Human	Social Determinants
	GOHI- Technological system (tech adoption): Logistics performance index	quantitative	Human	Social Determinants
	GOHI- Technological system (tech adoption): Access to electricity	quantitative	Human	Social Determinants
	GOHI- Technological system (tech adoption): Share of renewable energy	quantitative	Human	Social Determinants
	GOHI- Technological system (consumption & production): Energy consumption	quantitative	Human	Social Determinants
	GOHI- Technological system (consumption & production): Electricity consumption	quantitative	Human	Social Determinants
	GOHI- Technological system (consumption & production): Solid waste	quantitative	Human	Social Determinants
	GOHI- Technological system (consumption & production): Electronic waste	quantitative	Human	Social Determinants
	GOHI- Technological system (consumption & production): SO2 emissions	quantitative	Human	Social Determinants
	GOHI- Technological system (consumption & production): Nitrogen emissions	quantitative	Human	Social Determinants

	GOHI- Technological system (consumption & production): Non-recycled waste	quantitative	Human	Social Determinants
	GOHI - Human health (health coverage): Life expectancy	quantitative	Human	Social Determinants
	GOHI - Human health (health coverage): Health service coverage	quantitative	Human	Access to Medicines & Health Services
	GOHI - Human health (health coverage): Domestic health expenditure	quantitative	Human	Access to Medicines & Health Services
	GOHI - Human health (health coverage): Health risks	quantitative	Human	Social Determinants
	GOHI - Human health (diseases burden): Infectious diseases	quantitative	Human	Social Determinants
	GOHI - Human health (diseases burden): NCDs	quantitative	Human	Social Determinants
	GOHI - Human health (diseases burden): Mental health	quantitative	Human	Social Determinants
	GOHI - Human health (injury & violence): Homicide	quantitative	Human	Social Determinants
	GOHI - Human health (injury & violence): Road traffic	quantitative	Human	Social Determinants
	GOHI - AMR surveillance system (consumption surveillance): Consumption in humans	quantitative	Human	Surveillance & Laboratory
	GOHI - Improve awareness & understanding (raising awareness & understanding): Raising awareness & understanding	quantitative	Human	Community Awareness & Enabling Behaviours
	GOHI - Improve awareness & understanding (professional multisector training activities): Training in human health sector	quantitative	Human	Workforce
	GOHI - AMR rate for antibiotics (carbapenems): CRKP	quantitative	Human	Surveillance & Laboratory
	GOHI - AMR rate for antibiotics (carbapenems): CRAB	quantitative	Human	Surveillance & Laboratory
	GOHI - AMR rate for antibiotics (carbapenems): CREC	quantitative	Human	Surveillance & Laboratory
	GOHI - AMR rate for antibiotics (carbapenems): CRPA	quantitative	Human	Surveillance & Laboratory

GOHI - AMR rate for antibiotics (glycopeptide): VREF (E. faecium)	quantitative	Human	Surveillance & Laboratory
GOHI - AMR rate for antibiotics (glycopeptide): VREF (E. faecalis)	quantitative	Human	Surveillance & Laboratory
GOHI - AMR rate for antibiotics (beta-lactams): MRSA	quantitative	Human	Surveillance & Laboratory
GOHI - AMR rate for antibiotics (beta-lactams): 3G beta-lactam-resistant KP	quantitative	Human	Surveillance & Laboratory
GOHI - AMR rate for antibiotics (beta-lactams): 3G beta-lactam-resistant EC	quantitative	Human	Surveillance & Laboratory
GOHI - AMR rate for antibiotics (beta-lactams): 3G beta-lactam-resistant SP	quantitative	Human	Surveillance & Laboratory
GOHI - AMR rate for antibiotics (beta-lactams): 3G beta-lactam-resistant PA	quantitative	Human	Surveillance & Laboratory
GOHI - AMR rate for antibiotics (macrolides): MRSP	quantitative	Human	Surveillance & Laboratory
GOHI - AMR rate for antibiotics (aminoglycosides): ARKP	quantitative	Human	Surveillance & Laboratory
GOHI - AMR rate for antibiotics (aminoglycosides): ARAB	quantitative	Human	Surveillance & Laboratory
GOHI - AMR rate for antibiotics (quinolones): QRKP	quantitative	Human	Surveillance & Laboratory
GOHI - AMR rate for antibiotics (quinolones): QREC	quantitative	Human	Surveillance & Laboratory
GOHI - AMR rate for antibiotics (quinolones): QRAB	quantitative	Human	Surveillance & Laboratory
GOHI - Source of infection (strategy & regulation): National guideline for surveillance/control	quantitative	Human	Prevention & Control/ Surveillance & Laboratory
GOHI - Outcomes (Yellow fever): Yellow fever vaccination	quantitative	Human	Prevention & Control
GOHI - Targeted population (vaccine coverage): Vaccination strategy and regulation of vaccination	quantitative	Human	Prevention & Control

GOHI - Targeted population (population coverage & intervention costs): Costs directed to chemotherapy/vaccination of humans	quantitative	Human	Prevention & Control
GOHI - Outcomes (Echinococcosis): Echinococcosis DALYs	quantitative	Human	Surveillance & Laboratory
GOHI - Outcomes (Leishmaniasis): Leishmaniasis DALYs	quantitative	Human	Surveillance & Laboratory
GOHI - Outcomes (Rabies): Rabies DALYs	quantitative	Human	Surveillance & Laboratory
GOHI - Food safety (foodborne illness burden): DALY of diarrhoea	quantitative	Human	Surveillance & Laboratory
GOHI - Outcomes (Yellow fever): Yellow fever DALYs	quantitative	Human	Surveillance & Laboratory
GOHI - Good governance (equity & inclusiveness): Social inclusion	quantitative	Multisector	Participation/ Equity
GOHI - AMR lab network & coordination capacity (national AMR capacity): Lab for bacteria	quantitative	Multisector	Surveillance & Laboratory
GOHI - AMR lab network & coordination capacity (national AMR capacity): Lab for fungi	quantitative	Multisector	Surveillance & Laboratory
GOHI - AMR lab network & coordination capacity (national AMR capacity): Effective integration of labs	quantitative	Multisector	Surveillance & Laboratory
GOHI - AMR lab network & coordination capacity (technical level): Multisector working on AMR	quantitative	Multisector	Surveillance & Laboratory
GOHI - AMR lab network & coordination capacity (technical level): Standardisation and harmonisation of labs	quantitative	Multisector	Surveillance & Laboratory
GOHI - AMR lab network & coordination capacity (technical level): Relevant diagnostic techniques	quantitative	Multisector	Surveillance & Laboratory
GOHI - AMR lab network & coordination capacity (technical level): Technical level of data management	quantitative	Multisector	Surveillance & Laboratory

GOHI - Good governance (transparency): Data availability of national statistical system	quantitative	Multisector	Surveillance & Laboratory
GOHI - Good governance (participation): Global connectivity	quantitative	Multisector	Participation
GOHI - Good governance (participation): One Health association	quantitative	Multisector	Strategic Vision
GOHI - Good governance (participation): One Health forums	quantitative	Multisector	Participation
GOHI - Good governance (responsiveness): Emergency response operation	quantitative	Multisector	Feedback Mechanisms
GOHI - Good governance (responsiveness): Risk communication	quantitative	Multisector	Feedback Mechanisms
GOHI - Good governance (effectiveness & efficiency): Government effectiveness	quantitative	Multisector	Strategic Vision
GOHI - Good governance (political support): One Health official department	quantitative	Multisector	Strategic Vision
GOHI - Good governance (political support): Financial input	quantitative	Multisector	Sustainability
GOHI - AMR lab network & coordination capacity (national plan): NAP on monitoring & evaluation	quantitative	Multisector	Effectiveness
GOHI - AMR lab network & coordination capacity (national plan): NAP on AMR	quantitative	Multisector	Strategic Vision
GOHI - AMR lab network & coordination capacity (national plan): NAP on AMR linked to any other existing action plans	quantitative	Multisector	Coordination
GOHI - AMR lab network & coordination capacity (national plan): Publishment of action plan	quantitative	Multisector	Transparency
GOHI - Good governance (rule of law): One Health specialised law & regulation	quantitative	Multisector	Regulations & Legislation
GOHI - Food demand & supply (infrastructures score): Logistic performance index	quantitative	Multisector	Social Determinants
GOHI - Food demand & supply (infrastructures score): Net capital stocks	quantitative	Multisector	Social Determinants

GOHI - Good governance (consensus oriented): One Health education	quantitative	Multisector	Workforce
GOHI - Good governance (political support): Regulatory quality	quantitative	Multisector	Regulations & Legislation
GOHI - AMR surveillance system (consumption surveillance): Pesticide use	quantitative	Environment	Surveillance & Laboratory/Stewardship
GOHI - AMR surveillance system (environmental AMR surveillance): Environmental surveillance system	quantitative	Environment	Surveillance & Laboratory
GOHI - AM control & optimisation (AMU optimisation): Optimising AMU in pesticide use	quantitative	Environment	Stewardship
GOHI - AM control & optimisation (national law for antibiotic use): National law for antbiotic use in humans		Human	Regulations & Legislation
GOHI - AM control & optimisation (national law for antibiotic use): National law for prohibition of antibiotic use		Multisector	Regulations & Legislation
GOHI - AM control & optimisation (national law for antibiotic use): National law for marketing of pesticides	quantitative	Environment	Regulations & Legislation
GOHI - Food demand & supply (food loss & waste): Food loss	quantitative	Environment	Social Determinants
GOHI - Food demand & supply (food loss & waste): Food waste	quantitative	Environment	Social Determinants
GOHI - Food demand & supply (food aid): Food aid	quantitative	Environment	Social Determinants
GOHI - Food demand & supply (food production score): Average value of food production	quantitative	Environment	Social Determinants
GOHI - Natural & social circumstances (economic performance index): Trade balance indicators	quantitative	Environment	Social Determinants
GOHI - Natural & social circumstances (economic performance index): Economic vulnerability index	quantitative	Environment	Social Determinants

GOHI - Natural & social circumstances (economic performance index): Cereal import dependency ratio	quantitative	Environment	Social Determinants
GOHI - Natural & social circumstances (economic performance index): Value of food imports over total merchandise exports	quantitative	Environment	Social Determinants
GOHI - Natural & social circumstances (agricultural value per added worker): Agricultural value per added worker	quantitative	Environment	Workforce
GOHI - Natural & social circumstances (food price indicators): Agricultural import tariffs	quantitative	Environment	Social Determinants
GOHI - Earth system (land): Country area	quantitative	Environment	Social Determinants
GOHI - Earth system (land): Cultivated area	quantitative	Environment	Social Determinants
GOHI - Earth system (land): Arable land area	quantitative	Environment	Social Determinants
GOHI - Earth system (land): Terrain Ruggedness Index	quantitative	Environment	Social Determinants
GOHI - Earth system (forest): Forest area	quantitative	Environment	Social Determinants
GOHI - Earth system (forest): Forest Transition Phase	quantitative	Environment	Social Determinants
GOHI - Earth system (forest): Trees per capita	quantitative	Environment	Social Determinants
GOHI - Earth system (forest): Permanent deforestation	quantitative	Environment	Social Determinants
GOHI - Earth system (water): Renewable water resources	quantitative	Environment	Social Determinants
GOHI - Earth system (water): Water dependency ratio	quantitative	Environment	Social Determinants
GOHI - Earth system (water): Water stress	quantitative	Environment	Social Determinants
GOHI - Earth system (air): CO2 emissions	quantitative	Environment	Social Determinants
GOHI - Earth system (air): Air pollution index	quantitative	Environment	Social Determinants
GOHI - Animal health & ecosystem diversity (wildlife & marine life biodiversity): Red list index	quantitative	Environment	Social Determinants

GOHI - Environmental health (air quality & climate change): Air quality	quantitative	Environment	Social Determinants
GOHI - Environmental health (air quality & climate change): Climate risk index	quantitative	Environment	Social Determinants
GOHI - Environmental health (environmental biodiversity): Biodiversity conditions	quantitative	Environment	Social Determinants
GOHI - Environmental health (environmental biodiversity): Ecological services	quantitative	Environment	Surveillance & Laboratory
GOHI - Environmental health (environmental resources): Water resources	quantitative	Environment	Social Determinants
GOHI - Environmental health (environmental resources): Acidification	quantitative	Environment	Social Determinants
GOHI - Environmental health (environmental resources): Clean water	quantitative	Environment	Social Determinants
GOHI - Environmental health (environmental resources): Waste reduction	quantitative	Environment	Social Determinants
GOHI - Environmental health (environmental resources): Heavy metal pollutants	quantitative	Environment	Social Determinants
GOHI - Good governance (equity & inclusiveness): Biodiversity protection	quantitative	Environment	Social Determinants
GOHI - Good governance (effectiveness & efficiency): Climate change governance	quantitative	Environment	Strategic Vision
GOHI - Source of infection (hygiene): Basic sanitation services	quantitative	Environment	Prevention & Control/ Social Determinants
GOHI - Route of transmission (ecological intervention): Policy adoption of insecticide-treated mosquito nets	quantitative	Environment	Regulations & Legislation/ Prevention & Control
GOHI - Route of transmission (ecological intervention): Policy adoption for indoor residual spraying	quantitative	Environment	Regulations & Legislation/ Prevention & Control
GOHI - Targeted population (population coverage & intervention costs): Proportion of population having basic drinking water and sanitation facilities	quantitative	Environment	Prevention & Control/ Social Determinants

	GOHI - Targeted population (inhabitants below 5m above sea level): Proportion of population living in the areas where elevation is below 5 meters)	quantitative	Environment	Social Determinants
	GOHI - Capacity building (nature reserves): Proportion of natural protected areas	quantitative	Environment	Social Determinants
	GOHI - Food demand & supply (infrastructures score): % of arable land equipped for irrigation	quantitative	Environment	Prevention & Control
	GOHI - Natural & social circumstances (famine warning): Food affected by extreme weather conditions, disasters or crisis	quantitative	Environment	Social Determinants
	GOHI - Natural & social circumstances (natural sources sustainability): Per person land under cereal production	quantitative	Environment	Social Determinants
	GOHI - Natural & social circumstances (natural sources sustainability): Agricultural water withdrawal as % of total renewable water resources	quantitative	Environment	Social Determinants
	GOHI - Natural & social circumstances (natural sources sustainability): Agriculture area under organic agric	quantitative	Environment	Social Determinants
	GOHI - Natural & social circumstances (natural sources sustainability): Naturally regenerating forest	quantitative	Environment	Social Determinants
	GOHI - Natural & social circumstances (natural sources sustainability): % of agriculture land area affected by soil erosion	quantitative	Environment	Social Determinants
	GOHI - Natural & social circumstances (natural sources sustainability): Crop diversity	quantitative	Environment	Social Determinants
	GOHI - Natural & social circumstances (natural sources sustainability): Agricultural nitrous oxide emissions	quantitative	Environment	Social Determinants
	GOHI - Government support & response (investment & financial support score): Government Investment on agriculture	quantitative	Environment	Sustainability

	GOHI - Government support & response (investment & financial support score): Credit to Agriculture, Forestry, Fishing	quantitative	Environment	Sustainability
	GOHI - Government support & response (investment & financial support score): Agricultural R&D Investment Intensity	quantitative	Environment	Research, Innovation, & Digital Technology
	GOHI - Government support & response (training & AI agriculture performance score): Training programme	quantitative	Environment	Workforce
	GOHI - Government support & response (training & AI agriculture performance score): Smart & digital agriculture	quantitative	Environment	Research, Innovation, & Digital Technology
	GOHI - Climate change risk (GG emissions): CO2 emissions	quantitative	Environment	Social Determinants
	GOHI - Climate change risk (GG emissions): Methane emissions	quantitative	Environment	Social Determinants
	GOHI - Climate change risk (GG emissions): Nitrous oxide	quantitative	Environment	Social Determinants
	GOHI - Climate change risk (GG emissions): Other GG emissions	quantitative	Environment	Social Determinants
	GOHI - Climate change risk (energy use): Oil	quantitative	Environment	Social Determinants
	GOHI - Climate change risk (energy use): Natural gas	quantitative	Environment	Social Determinants
	GOHI - Climate change risk (energy use): Coal	quantitative	Environment	Social Determinants
	GOHI - Climate change risk (energy use): Energy intensity	quantitative	Environment	Social Determinants
	GOHI - Climate change risk (air quality): Ozone exposure	quantitative	Environment	Social Determinants
	GOHI - Climate change risk (air quality): Inhalable particles	quantitative	Environment	Social Determinants
	GOHI - Climate change risk (natural disaster & extreme weather): Wildfires	quantitative	Environment	Social Determinants
	GOHI - Climate change risk (natural disaster & extreme weather): Droughts, floods & storms	quantitative	Environment	Social Determinants

	GOHI - Climate change risk (natural disaster & extreme weather): Extreme high temperature	quantitative	Environment	Social Determinants
	GOHI - Climate change risk (natural disaster & extreme weather): Extreme low temperature	quantitative	Environment	Social Determinants
	GOHI - Health outcomes (air quality DALYs): Inhalable particles burden	quantitative	Environment	Social Determinants
	GOHI - Health outcomes (climate-related illnesses): Heat-related illness burden	quantitative	Environment	Social Determinants
	GOHI - Health outcomes (climate-related illnesses): Vulnerability to extremes of heat	quantitative	Environment	Social Determinants
	GOHI - Health outcomes (climate-related illnesses): Death from disasters	quantitative	Environment	Social Determinants
	GOHI - Mitigation & adaptation capacity (mitigation & adaptation achievement): Low-carbon energy use	quantitative	Environment	Social Determinants
	GOHI - Mitigation & adaptation capacity (mitigation & adaptation achievement): Hydro energy use	quantitative	Environment	Social Determinants
	GOHI - Mitigation & adaptation capacity (mitigation & adaptation achievement): Solar energy use	quantitative	Environment	Social Determinants
	GOHI - Mitigation & adaptation capacity (mitigation & adaptation achievement): Wind energy use	quantitative	Environment	Social Determinants
	GOHI - Mitigation & adaptation capacity (mitigation & adaptation achievement): Renewable energy capacity	quantitative	Environment	Social Determinants
	GOHI - Improve awareness & understanding (professional multisector training activities): Training in farming sector	quantitative	Environment	Workforce
	GOHI - Mitigation & adaptation capacity (financial support): Climate adaptation spending	quantitative	Environment	Social Determinants
	GOHI - Mitigation & adaptation capacity (financial support): Renewable energy finance flows	quantitative	Environment	Social Determinants

	GOHI - Mitigation & adaptation capacity (propaganda & education): Climate health education	quantitative	Environment	Social Determinants
	GOHI - Mitigation & adaptation capacity (propaganda & education): Climate health research	quantitative	Environment	Social Determinants
	GOHI - Mitigation & adaptation capacity (forestation): Forest change	quantitative	Environment	Social Determinants
	GOHI - Mitigation & adaptation capacity (forestation): Reforestation	quantitative	Environment	Social Determinants
	GOHI - Mitigation & adaptation capacity (forestation): Deforestation	quantitative	Environment	Social Determinants
	GOHI - Source of infection (strategy & regulation): National legislation on animal reservoirs	qualitative	Animal	Regulations & Legislation
	GOHI - Source of infection (monitoring & feedback): General surveillance	qualitative	Animal	Surveillance & Laboratory
	GOHI - Animal health & ecosystem diversity (animal epidemic disease): Diseases of domestic animal	quantitative	Animal	Surveillance & Laboratory
	GOHI - Animal health & ecosystem diversity (animal epidemic disease): Diseases of wild animal	quantitative	Animal	Surveillance & Laboratory
	GOHI - Animal health & ecosystem diversity (wildlife & marine life biodiversity): Fisheries	quantitative	Animal	Prevention & Control
	GOHI - Good governance (effectiveness & efficiency): Zoonotic disease governance	quantitative	Animal	Strategic Vision
	GOHI - Source of infection (strategy & regulation): Zoonosis capacity score	quantitative	Animal	Prevention & Control
	GOHI - Source of infection (monitoring & feedback): Vector control	quantitative	Animal	Prevention & Control
	GOHI - Source of infection (monitoring & feedback): Wildlife reservoirs control	quantitative	Animal	Prevention & Control

	GOHI - Route of transmission (conventional intervention): Lab testing for zoonotic reservoirs (vectors and animals)	quantitative	Animal	Surveillance & Laboratory
	GOHI - Route of transmission (ecological intervention): Prevention chemotherapy coverage of zoonoses	quantitative	Animal	Prevention & Control
	GOHI - Capacity building (guidelines for control & supervision of zoonotic diseases): Legislation of zoonosis educational activities	quantitative	Animal	Regulations & Legislation
	GOHI - Capacity building (guidelines for control & supervision of zoonotic diseases): Zoonosis vaccine national plan	quantitative	Animal	Prevention & Control
	GOHI - Capacity building (guidelines for control & supervision of zoonotic diseases): Zoonotic Events and the Human-animal Interface	quantitative	Animal	Prevention & Control
	GOHI - Capacity building (guidelines for control & supervision of zoonotic diseases): Universal health coverage (UHC) Service Coverage subindex on infectious diseases	quantitative	Animal	Access to Medicines & Health Services
	GOHI - Capacity building (guidelines for control & supervision of zoonotic diseases): NTD control and prevention	quantitative	Animal	Prevention & Control
	GOHI - Capacity building (guidelines for control & supervision of zoonotic diseases): Surveillance	quantitative	Animal	Surveillance & Laboratory
	GOHI - AMR surveillance system (AMR status surveillance): AMR in terrestrial animals	quantitative	Animal	Surveillance & Laboratory
	GOHI - AMR surveillance system (AMR status surveillance): AMR in aquatic animals	quantitative	Animal	Surveillance & Laboratory
	GOHI - AMR surveillance system (AMR status surveillance): AMR in food	quantitative	Animal	Surveillance & Laboratory
	GOHI - AM control & optimisation (national law for antibiotic use): National law for antibiotic use in terrestrial animals	quantitative	Animal	Regulations & Legislation

	GOHI - AM control & optimisation (national law for antibiotic use): National law for antibiotic use in aquatic animals	quantitative	Animal	Regulations & Legislation
	GOHI - AM control & optimisation (AMU optimisation): Optimising AMU in terrestrial animal health	quantitative	Animal	Regulations & Legislation
	GOHI - AM control & optimisation (AMU optimisation): Optimising AMU in aquatic animal health	quantitative	Animal	Regulations & Legislation
	GOHI - AM control & optimisation (AMU control): Reduce transmission of AMR in terrestrial animal production	quantitative	Animal	Regulations & Legislation
	GOHI - AM control & optimisation (AMU control): Reduce transmission of AMR in aquatic animal production	quantitative	Animal	Regulations & Legislation
	GOHI - AM control & optimisation (AMU control): Reduce transmission of AMR in food processing	quantitative	Animal	Prevention & Control
	GOHI - Improve awareness & understanding (professional multisector training activities): Training in vet sector	quantitative	Animal	Workforce
	GOHI - Improve awareness & understanding (professional multisector training activities): Progress with strengthening vet services	quantitative	Animal	Strategic Vision
	GOHI - Food demand & supply (food production score): Food production viability	quantitative	Animal	Social Determinants
	GOHI - Food demand & supply (food production score): Livestock production index	quantitative	Animal	Social Determinants
	GOHI - Food safety (food safety governance): Food safety agency	quantitative	Animal	Regulations & Legislation
	GOHI - Food safety (food safety governance): Food policy, legal and regulatory framework	quantitative	Animal	Regulations & Legislation/ Accountability
	GOHI - Food safety (food safety control & surveillance): Inspections in farm-to-fork food chain	quantitative	Animal	Prevention & Control

		GOHI - Food safety (food safety control & surveillance): Food recalls	quantitative	Animal	Prevention & Control
		GOHI - Food safety (food safety evaluation): Food safety score	quantitative	Animal	Effectiveness
		GOHI - Food safety (safety of livestock production): Livestock density	quantitative	Animal	Prevention & Control
		GOHI - Food safety (safety of livestock production): Domestic animal disease outbreak	quantitative	Animal	Prevention & Control
		GOHI - Food safety (safety of livestock production): Manure management	quantitative	Animal	Prevention & Control
		GOHI - Natural & social circumstances (natural sources sustainability): Livestock diversity	quantitative	Animal	Prevention & Control
		GOHI - AMR surveillance system (consumption surveillance): Consumption in animals	quantitative	Animal	Stewardship
88	Identifying AWaRe indicators for appropriate antibiotic use: a narrative review	Within the guidelines, are there standardized criteria for changing from intravenous to oral antimicrobial therapy in appropriate situations?	Y/N	Human	Stewardship
		Percentage of acute health care facilities with an antimicrobial stewardship programme in place	quantitative	Human	Stewardship
		No. of patients with specific drug-resistant organisms	quantitative	Human	Surveillance & Laboratory
		Proportion of total antibiotic prescribing from the "Access" category of the WHO Essential Medicines List AWaRe index; by quarter and acute trust	quantitative	Human	Stewardship
		Percentage of adult and paediatric hospital patients receiving an antibiotic according to AWaRe categories	quantitative	Human	Stewardship
		Proportion of total antibiotic prescribing from the “Watch” category of the WHO Essential Medicines List AWaRe index	quantitative	Human	Stewardship
		Proportion of total antibiotic prescribing from the “Reserve” category of the WHO Essential Medicines List AWaRe index	quantitative	Human	Stewardship

List of “Reserve” antibiotics with authorisation system for delivery available	qualitative	Human	Stewardship
Community Acquired Pneumonia Admission Rate	quantitative	Human	Access to Medicines & Health Services
Percentage of penicillin prescriptions in dental treatments	quantitative	Human	Stewardship
Percentage of clindamycin prescriptions in dental treatments	quantitative	Human	Stewardship
Percentage of tetracycline prescriptions in dental treatments	quantitative	Human	Stewardship
Percentage of all primary care consultations in which an antibiotic is prescribed or dispensed	quantitative	Human	Stewardship
Initial therapy (drugs) according to local / national guideline	quantitative	Human	Stewardship
Indications for a disease based on clinical guidelines x 100 / total number of indications for that disease	quantitative	Human	Stewardship
The proportion of prescriptions for restricted antimicrobials that are in accordance with the locally endorsed approval policy	quantitative	Human	Stewardship
Number of deaths by type of infection / Total number of patients with that infection	quantitative	Human	Surveillance & Laboratory
Days of hospitalization by type of infection / Total number of patients with that infection	quantitative	Human	Surveillance & Laboratory
% of drugs prescribed from essential drug list or formulary	quantitative	Human	Stewardship
Antibiotic generic prescribing rate (%)	quantitative	Human	Stewardship
Consumption of cephalosporins expressed in DID	quantitative	Human	Stewardship
Consumption of third and fourth generation of cephalosporins expressed as percentage	quantitative	Human	Stewardship
Number of patients treated with cephalosporins	quantitative	Human	Stewardship
Consumption of quinolones expressed in DID	quantitative	Human	Stewardship

	Selective use of fluoroquinolones (only as oral or in beta-lactam allergy/anaphylaxis)	quantitative	Human	Stewardship
	Ciprofloxacin use	quantitative	Human	Stewardship
	Seasonal variation of quinolone consumption	quantitative	Human	Stewardship
	Prescriptions of moxifloxacin expressed as a percentage of the total prescriptions of antibacterials for systemic use	quantitative	Human	Stewardship
	Prescriptions of levofloxacin, ofloxacin or ciprofloxacin among patients having been prescribed a quinolone (J01M) in the preceding 6 months, expressed as a percentage of the total prescriptions of quinolones	quantitative	Human	Stewardship
	Prescriptions of first-generation quinolones, expressed as a percentage of the total prescriptions of antibacterials for systemic use	quantitative	Human	Stewardship
	Prescriptions of moxifloxacin or levofloxacin associated on the same day with another antibiotic, expressed as a percentage of the total prescriptions of antibacterials for systemic use	quantitative	Human	Stewardship
	Prescriptions of nitrofurantoine (J01XE01), first-generation quinolones or fosfomycin–trometamol prescriptions in male patients, expressed as a percentage of the total prescriptions of antibacterials for systemic use in male patients	quantitative	Human	Stewardship
	Consumption of penicillins expressed in DID	quantitative	Human	Stewardship
	Consumption of b-lactamase sensitive penicillins expressed as percentage	quantitative	Human	Stewardship
	Amoxicillin use	quantitative	Human	Stewardship
	Flucloxacillin use	quantitative	Human	Stewardship
	Phenoxymethylpenicillin use	quantitative	Human	Stewardship
	Co-amoxiclav use	quantitative	Human	Stewardship

	Consumption of combination of penicillins, including b-lactamase inhibitor expressed as percentage	quantitative	Human	Stewardship
	Proportion of amoxicillin users (amoxicillin index)	quantitative	Human	Stewardship
	Relative number of amoxicillin prescriptions versus amoxicillin/clavulanate prescriptions	quantitative	Human	Stewardship
	Number of patients treated with narrow-spectrum penicillin	quantitative	Human	Stewardship
	Percentage of prescribed antibiotic items from cephalosporin, quinolone and co-amoxiclav class by quarter	quantitative	Human	Stewardship
	Twelve-month rolling percentage of prescribed antibiotic items from cephalosporin, quinolone and co-amoxiclav class	quantitative	Human	Stewardship
	Number of patients treated with broad-spectrum penicillin + /– clavulanic acid	quantitative	Human	Stewardship
	Ratio between broad-spectrum beta-lactam versus non-broad-spectrum beta-lactams per discipline	quantitative	Human	Stewardship
	Ratio between users of amoxicillin to broad-spectrum penicillins, cephalosporins and macrolides (A/B ratio)	quantitative	Human	Stewardship
	Ratio of the consumption of broad to the consumption of narrow spectrum penicillins, cephalosporins and macrolide	quantitative	Human	Stewardship
	Percentage of broad-spectrum prescribed antibiotic items (cephalosporin, quinolone and co-amoxiclav class) by quarter	quantitative	Human	Stewardship
	Proportion of cephalosporins and quinolones, of total antibiotic drugs used	quantitative	Human	Stewardship
	Volume of cephalosporins and quinolones as a proportion of all systemic antibiotics prescribed (DDD)	quantitative	Human	Stewardship

Consumption of macrolides, lincosamides and streptogramins expressed in DID	quantitative	Human	Stewardship
Total tetracycline use	quantitative	Human	Stewardship
Doxycycline use	quantitative	Human	Stewardship
Total macrolide use	quantitative	Human	Stewardship
Percentage of erythromycin among total macrolides prescribed	quantitative	Human	Stewardship
Number of patients treated with macrolides	quantitative	Human	Stewardship
Ratio of co-trimoxazole items to trimethoprim items	quantitative	Human	Stewardship
Trimethoprim use	quantitative	Human	Stewardship
Co-trimoxazole use	quantitative	Human	Stewardship
Twelve-month rolling proportion of trimethoprim class prescribed antibiotic items as a ratio of trimethoprim to nitrofurantoin	quantitative	Human	Stewardship
Nitrofurantoin use	quantitative	Human	Stewardship
Carbapenem prescribing DDDs per 1000 admissions by quarter and acute trust	quantitative	Human	Stewardship
% of prescriptions with an antibiotic	quantitative	Human	Stewardship
Clindamycin use	quantitative	Human	Stewardship
Proportion of Access antibiotics for systemic use, relative to total antibiotic consumption in DDD	quantitative	Human	Stewardship
Proportion of DDD in AWaRe and OTHER groups	quantitative	Human	Stewardship
Antimicrobial prescribing rates	quantitative	Human	Stewardship
Total number of antibiotic prescriptions	quantitative	Human	Stewardship
Consumption of antibacterials for systemic use expressed in DID (Defined daily doses per 1000 inhabitants per day)	quantitative	Human	Stewardship
Total antibacterial use	quantitative	Human	Stewardship
Total antibiotic use per 1000 persons per day	quantitative	Human	Stewardship

	Overall volume of antibiotics for systemic use prescribed (DDD)	quantitative	Human	Stewardship
	DDDs per defined population	quantitative	Human	Stewardship
	Number of healthcare-associated infections (MDR organisms (e.g.MRSA, ESBL-E/CPE,MDR Pseudomonas and Acinetobacter spp., vancomycin-resistant enterococci) in a period of time / Total number of patient-days within that period x 100 000	quantitative	Human	Stewardship
	E. coli bacteraemia hospital-onset counts and rates by NHS acute trust and financial year	quantitative	Human	Stewardship
	Antimicrobial prescribing rates in hospitals	quantitative	Human	Stewardship
	Antimicrobial prescribing rates in primary care	quantitative	Human	Stewardship
	Antibiotic prescribing rates (primary and secondary care)	quantitative	Human	Stewardship
	Treatments/courses per defined population	quantitative	Human	Stewardship
	Prescriptions per defined population	quantitative	Human	Stewardship
	Treatments/courses per defined number of physician contacts	quantitative	Human	Stewardship
	Prescriptions per defined number of physician contacts	quantitative	Human	Stewardship
	Relative proportion of AWaRe (Access, Watch and Reserve) antibiotics for paediatric formulations	quantitative	Human	Stewardship
	C. difficile hospital-onset rates by reporting acute trust and financial year	quantitative	Human	Surveillance & Laboratory
	Mortality related to antimicrobial-resistant organisms	quantitative	Human	Surveillance & Laboratory
	Incidence of vascular access device-related bloodstream infection	quantitative	Human	Prevention & Control
	Number of deaths during hospitalization / Total number of hospitalizations	quantitative	Human	Prevention & Control
	Rates of admission to critical care for people with sepsis	quantitative	Human	Prevention & Control

		Antibiotic resistance data regarding MRSA analysed and written report provided at least 1×/year	Y/N	Human	Surveillance & Laboratory
		Antibiotic resistance data regarding ESBL analysed at least 1×/year	Y/N	Human	Surveillance & Laboratory
		Antibiotic resistance data (other than MRSA and ESBL) analysed at least 1×/year	Y/N	Human	Surveillance & Laboratory
		Countries that conduct regular and risk-based post-market surveillance on antimicrobials a / For humans b / For animals (terrestrial, aquatic)	qualitative	Animal	Surveillance & Laboratory
89	Identifying the drivers of multidrug-resistant Klebsiella pneumoniae at a European level	Correlation between hospital transmission rate and n of HCW employed in hospital (/100,000)	quantitative	Human	Workforce
		Correlation between hospital transmission rate and mean yearly expenditures on healthcare ($PPPpc)	quantitative	Human	Access to Medicines & Health Services
		Correlation between hospital transmission rate and n of practising nurses (/100,000)	quantitative	Human	Workforce
		Prevalence of resistant ESBL strains (%) by country	quantitative	Human	Surveillance & Laboratory
		Prevalence of resistant CRK strains (%) by country	quantitative	Human	Surveillance & Laboratory
		Prevalence of resistance (%) to 3G cephalosporins by country	quantitative	Human	Surveillance & Laboratory
		Prevalence of resistance (%) to carbapenems by country	quantitative	Human	Surveillance & Laboratory
		Outpatient consumption of 3rd & 4th generation cephalosporins (DID) by country	quantitative	Human	Stewardship
		Outpatient consumption of carbapenems (DID) by country	quantitative	Human	Stewardship
		Inpatient consumption of 3rd & 4th generation cephalosporins (DID) by country	quantitative	Human	Stewardship
		Inpatient consumption of carbapenems (DID) by country	quantitative	Human	Stewardship

		Correlation between resistance prevalence (%) and inpatient consumption of 3rd & 4th generation cephalosporins (DDD)	quantitative	Human	Surveillance & Laboratory
		Correlation between resistance prevalence (%) and total consumption of 3rd & 4th generation cephalosporins (DDD)	quantitative	Human	Surveillance & Laboratory
		Correlation between resistance prevalence (%) and outpatient consumption of 3rd & 4th generation cephalosporins (DDD)	quantitative	Human	Surveillance & Laboratory
		Correlation between resistance prevalence (%) and inpatient consumption of 3rd & 4th generation cephalosporins and carbapenems (DDD)	quantitative	Human	Surveillance & Laboratory
		Correlation between resistance prevalence (%) and total consumption of 3rd & 4th generation cephalosporins and carbapenems (DDD)	quantitative	Human	Surveillance & Laboratory
90	In Vitro Activity of Ceftazidime-Avibactam against Clinical Isolates of Enterobacteriaceae and Pseudomonas aeruginosa Collected in Asia-Pacific Countries: Results from the INFORM Global Surveillance Program, 2012 to 2015	MIC (mg/ml) and susceptibility (%) of Enterobacteriaceae isolates against 12 antimicrobial agents	quantitative	Human	Surveillance & Laboratory
		MIC (mg/ml) and susceptibility (%) of P. aeruginosa isolates against 12 antimicrobial agents	quantitative	Human	Surveillance & Laboratory
		Susceptibility (%) to ceftazidime-avibactam for isolates of Enterobacteriaceae by country	quantitative	Human	Surveillance & Laboratory
		MIC (mg/ml) and susceptibility (%) of beta-lactamase positive Enterobacteriaceae isolates against 12 antimicrobial agents	quantitative	Human	Surveillance & Laboratory
		MIC (mg/ml) and susceptibility (%) of ceftazidime-nonsusceptible Enterobacteriaceae isolates against 12 antimicrobial agents	quantitative	Human	Surveillance & Laboratory
		MIC (mg/ml) and susceptibility (%) of ceftazidime-nonsusceptible P. aeruginosa isolates against 12 antimicrobial agents	quantitative	Human	Surveillance & Laboratory

		MIC (mg/ml) and susceptibility (%) of meropenem-nonsusceptible Enterobacteriaceae isolates against 12 antimicrobial agents	quantitative	Human	Surveillance & Laboratory
		MIC (mg/ml) and susceptibility (%) of meropenem-nonsusceptible P. aeruginosa isolates against 12 antimicrobial agents	quantitative	Human	Surveillance & Laboratory
		MIC (mg/ml) and susceptibility (%) of colistin-resistant Enterobacteriaceae isolates against 12 antimicrobial agents	quantitative	Human	Surveillance & Laboratory
		MIC (mg/ml) and susceptibility (%) of colistin-resistant P. aeruginosa isolates against 12 antimicrobial agents	quantitative	Human	Surveillance & Laboratory
		MIC (mg/ml) and susceptibility (%) of MDR Enterobacteriaceae isolates against 12 antimicrobial agents	quantitative	Human	Surveillance & Laboratory
		MIC (mg/ml) and susceptibility (%) of MDR P. aeruginosa isolates against 12 antimicrobial agents	quantitative	Human	Surveillance & Laboratory
		% of Enterobacteriaceae isolates that were MDR by country	quantitative	Human	Surveillance & Laboratory
		Susceptibility (%) to ceftazidime-avibactam for isolates of P. aeruginosa by country	quantitative	Human	Surveillance & Laboratory
		% of P. aeruginosa isolates that were MDR by country	quantitative	Human	Surveillance & Laboratory
91	In Vitro Activity of Ceftazidime-Avibactam against Clinical Isolates of Enterobacteriaceae and Pseudomonas aeruginosa Collected in WHO AMR (Latin America)n Countries: Results from the INFORM Global Surveillance Program, 2012 to 2015	In vitro susceptibilities to ceftazidime-avibactam and comparators for clinical isolates of Enterobacteriaceae and P. aeruginosa	quantitative	Human	Surveillance & Laboratory
92	In vitro activity of ceftazidime-avibactam against enterobacterales and Pseudomonas aeruginosa isolates collected in Latin America as part of	ESBLs identified in enterobacterales isolates by country	quantitative	Human	Surveillance & Laboratory
		Beta-lactamases identified in MEM-NS enterobacterales isolates by country	quantitative	Human	Surveillance & Laboratory

the ATLAS global surveillance program, 2017-2019	Beta-lactamases identified in MEM-NS P. aeruginosa isolates by country	quantitative	Human	Surveillance & Laboratory
	MIC (mg/ml) & CLSI by antimicrobial agent against all enterobacterales isolates	quantitative	Human	Surveillance & Laboratory
	MIC (mg/ml) & CLSI by antimicrobial agent against meropenem-NS enterobacterales isolates	quantitative	Human	Surveillance & Laboratory
	MIC (mg/ml) & CLSI by antimicrobial agent against meropenem-NS, MBL-, carbapenemase+ enterobacterales isolates	quantitative	Human	Surveillance & Laboratory
	MIC (mg/ml) & CLSI by antimicrobial agent against meropenem-NS, MBL-, carbapenemase- enterobacterales isolates	quantitative	Human	Surveillance & Laboratory
	MIC (mg/ml) & CLSI by antimicrobial agent against MDR enterobacterales isolates	quantitative	Human	Surveillance & Laboratory
	MIC (mg/ml) & CLSI by antimicrobial agent against ESBL+ enterobacterales isolates	quantitative	Human	Surveillance & Laboratory
	MIC (mg/ml) & CLSI by antimicrobial agent against AmpC+ enterobacterales isolates	quantitative	Human	Surveillance & Laboratory
	MIC (mg/ml) & CLSI by antimicrobial agent against ESBL+ & AmpC+ enterobacterales isolates	quantitative	Human	Surveillance & Laboratory
	MIC (mg/ml) & CLSI by antimicrobial agent against KPC+ enterobacterales isolates	quantitative	Human	Surveillance & Laboratory
	MIC (mg/ml) & CLSI by antimicrobial agent against GES-type carbapenemase+ enterobacterales isolates	quantitative	Human	Surveillance & Laboratory
	MIC (mg/ml) & CLSI by antimicrobial agent against OXA-48-like+ enterobacterales isolates	quantitative	Human	Surveillance & Laboratory
	MIC (mg/ml) & CLSI by antimicrobial agent against MBL+ enterobacterales isolates	quantitative	Human	Surveillance & Laboratory
	MIC (mg/ml) & CLSI by antimicrobial agent against all P. aeruginosa isolates	quantitative	Human	Surveillance & Laboratory
	MIC (mg/ml) & CLSI by antimicrobial agent against meropenem-NS P. aeruginosa isolates	quantitative	Human	Surveillance & Laboratory

		MIC (mg/ml) & CLSI by antimicrobial agent against meropenem-NS, MBL-, carbapenemase+ P. aeruginosa isolates	quantitative	Human	Surveillance & Laboratory
		MIC (mg/ml) & CLSI by antimicrobial agent against meropenem-NS, MBL-, carbapenemase- P. aeruginosa isolates	quantitative	Human	Surveillance & Laboratory
		MIC (mg/ml) & CLSI by antimicrobial agent against MDR P. aeruginosa isolates	quantitative	Human	Surveillance & Laboratory
		MIC (mg/ml) & CLSI by antimicrobial agent against KPC+ P. aeruginosa isolates	quantitative	Human	Surveillance & Laboratory
		MIC (mg/ml) & CLSI by antimicrobial agent against GES-type carbapenemase+ P. aeruginosa isolates	quantitative	Human	Surveillance & Laboratory
		MIC (mg/ml) & CLSI by antimicrobial agent against MBL+ P. aeruginosa isolates	quantitative	Human	Surveillance & Laboratory
		MIC (mg/ml) & CLSI by antimicrobial agent against no acquired beta-lactamase P. aeruginosa isolates	quantitative	Human	Surveillance & Laboratory
		MIC (mg/ml) & CLSI by antimicrobial agent against ESBL+ P. aeruginosa isolates	quantitative	Human	Surveillance & Laboratory
93	In vitro activity of imipenem/relebactam against non-Morganellaceae Enterobacterales and Pseudomonas aeruginosa in Latin America: SMART 2018–2020	Susceptibility (%) of all and b-lactam-nonsusceptible isolates of P. aeruginosa by country	quantitative	Human	Surveillance & Laboratory
		Susceptibility (%) of all and b-lactam-nonsusceptible isolates of non-Morganellaceae Enterobacterales by country	quantitative	Human	Surveillance & Laboratory
		Estimated carbapenemase rates among all collected non-Morganellaceae Enterobacterales isolates (% carrying carbapenemase) by country	quantitative	Human	Surveillance & Laboratory
		Beta-lactamase gene carriage of imipenem/relebactam-nonsusceptible non-Morganellaceae Enterobacterales isolates (% of all collected isolates) by country	quantitative	Human	Surveillance & Laboratory
		Beta-lactamase gene carriage of imipenem/relebactam-nonsusceptible P.	quantitative	Human	Surveillance & Laboratory

		aeruginosa isolates (% of all collected isolates) by country			
94	In vitro activity of imipenem/relebactam against piperacillin/tazobactam-resistant and meropenem-resistant non-Morganellaceae Enterobacterales and Pseudomonas aeruginosa collected from patients with lower respiratory tract infections in Western Europe: SMART 2018-20	Susceptibility (%) of all isolates of NME and isolates with β-lactam-resistant phenotypes by country	quantitative	Human	Surveillance & Laboratory
		Susceptibility (%) of all and β-lactam-resistant phenotypes of P. aeruginosa by country	quantitative	Human	Surveillance & Laboratory
		Susceptibility (%) of all and b-lactam-non susceptible isolates of non-Morganellaceae Enterobacterales by country	quantitative	Human	Surveillance & Laboratory
		Susceptibility (%) of all and b-lactam-non susceptible isolates of P. aeruginosa by country	quantitative	Human	Surveillance & Laboratory
		Estimated carbapenemase rates among all collected P. aeruginosa isolates (% of isolates collected) by country	quantitative	Human	Surveillance & Laboratory
		Estimated carbapenemase rates among NME isolates (% carrying carbapenemase) by country	quantitative	Human	Surveillance & Laboratory
		β-Lactamase gene carriage (n, %) among imipenem/relebactam-resistant NME isolates	quantitative	Human	Surveillance & Laboratory
		Estimated carbapenemase rates among P. aeruginosa isolates (% carrying carbapenemase) by country	quantitative	Human	Surveillance & Laboratory
		β-Lactamase gene carriage among imipenem/relebactam (IMR)-resistant P. aeruginosa isolates (% of total isolates collected) by country	quantitative	Human	Surveillance & Laboratory
95	In Vitro Susceptibility of Global Surveillance Isolates of Pseudomonas aeruginosa to Ceftazidime-Avibactam (INFORM 2012 to 2014)	Occurence of beta-lactamase genes in carbapenem-nonsusceptible P. aeruginosa isolates, stratified by phenotype	quantitative	Human	Surveillance & Laboratory
96	Individual and institutional predisposing factors of MRSA surgical site infection and outcomes-a	Overall MRSA SSI incidence (n, %) by country	quantitative	Human	Surveillance & Laboratory
		MRSA SSI incidence rate (vascular surgery)	quantitative	Human	Surveillance & Laboratory
		MRSA SSI incidence rate (orthopaedic and trauma surgery)	quantitative	Human	Surveillance & Laboratory

	retrospective case-control-study in 14 European high-volume surgical centres	MRSA SSI incidence rate (visceral surgery)	quantitative	Human	Surveillance & Laboratory
		MRSA SSI incidence rate (neurosurgery)	quantitative	Human	Surveillance & Laboratory
		MRSA SSI incidence rate (dermatological surgery)	quantitative	Human	Surveillance & Laboratory
		MRSA SSI incidence rate (gynaecological surgery)	quantitative	Human	Surveillance & Laboratory
		MRSA SSI incidence rate (heart and cardiothoracic surgery)	quantitative	Human	Surveillance & Laboratory
		MRSA SSI incidence rate (urological surgery)	quantitative	Human	Surveillance & Laboratory
		MRSA SSI incidence rate (ENT surgery)	quantitative	Human	Surveillance & Laboratory
		MRSA SSI incidence rate (OMS surgery)	quantitative	Human	Surveillance & Laboratory
97	Inventory of antibiotic stewardship programs in general practice in France and abroad	Type of information by country AMS resource	qualitative	Multisector	Stewardship
		Recommendations/training by country AMS resource	qualitative	Multisector	Stewardship
		Feedback on antibiotic prescriptions by country AMS resource	qualitative	Multisector	Stewardship
		Rapid diagnostic tests by country AMS resource	qualitative	Multisector	Stewardship
		Advice for antibiotic therapy by country AMS resource	qualitative	Multisector	Stewardship
		Inciting measures by country AMS resource	qualitative	Multisector	Stewardship
		Restricted antibiotic prescriptions by country AMS resource	qualitative	Multisector	Stewardship
		Biological tests by country AMS resource	qualitative	Multisector	Stewardship
		Sale by unit by country AMS resource	quantitative	Multisector	Stewardship
98	Investigating the feasibility and potential of combining industry AMR monitoring systems: a comparison with WHO GLASS	Combined coverage (n of countries, total isolates, pathogens and antibiotics) of industry monitoring systems	quantitative	Human	Surveillance & Laboratory
		Resistance proportions (%) of bacteria (A. baumannii)-antibiotics combination per year in combined industry dataset	quantitative	Human	Surveillance & Laboratory
		Resistance proportions (%) of bacteria (E. coli)-antibiotics combination per year in combined industry dataset	quantitative	Human	Surveillance & Laboratory

		Resistance proportions (%) of bacteria (K. pneumoniae)-antibiotics combination per year in combined industry dataset	quantitative	Human	Surveillance & Laboratory
		Resistance proportions (%) of bacteria (S. aureus)-antibiotics combination per year in combined industry dataset	quantitative	Human	Surveillance & Laboratory
		Resistance proportions (%) of bacteria (S. pneumoniae)-antibiotics combination per year in combined industry dataset	quantitative	Human	Surveillance & Laboratory
		Coverage (n of countries, total isolates, pathogens and antibiotics) of each industry monitoring system	quantitative	Human	Surveillance & Laboratory
		WHO GLASS coverage (n of countries, total isolates, pathogens and antibiotics)	quantitative	Human	Participation
99	Isomorphic dynamics in national action plans on antimicrobial resistance	Isomorphic posturing, isomorphic mimicry and isomorphism patterns	quantitative	Multisector	Strategic Vision
		Isomorphic behaviour: Alignment with international best practices in both form and function	qualitative	Multisector	Strategic Vision
		Isomorphic mimicry behaviour: Alignment with international best practices in form but not in function	qualitative	Multisector	Strategic Vision
		Posturing behaviour: Alignment with international best practices in function but not in form	qualitative	Multisector	Strategic Vision
100	Livestock-associated meticillin-resistant Staphylococcus aureus (MRSA) among human MRSA isolates, European Union/European Economic Area countries, 2013	Type of sample/body site of MRSA-positive samples reported (n, %)	quantitative	Animal	Surveillance & Laboratory
		Availability of MRSA typing methods	quantitative	Animal	Surveillance & Laboratory
		Number of LA-MRSA isolates reported	quantitative	Animal	Surveillance & Laboratory
101	Mandatory surveillance and outbreaks reporting of the WHO priority pathogens for research & discovery of new antibiotics in European countries	Mandatory surveillance of WHO priority pathogens (n, %)	quantitative	Multisector	Surveillance & Laboratory
		Mandatory antibiotic resistance surveillance in gram-negative and gram-positive bacteria (n, %)	quantitative	Multisector	Surveillance & Laboratory

		Mandatory reporting of outbreaks caused by WHO priority pathogens (n, %)	quantitative	Multisector	Surveillance & Laboratory
102	Markers of epidemiological success of methicillin-resistant Staphylococcus aureus isolates in European populations	Median and total infection incidence because of MRSA for 5 infection types (BSI, UTI, RESP, SSI and others) by country	quantitative	Human	Surveillance & Laboratory
		AMR resistance (%) by successful and sporadic isolates within countries (cefoxitin, ciprofloxacin, chloramphenicol, clindamycin, erythromycin, fusidic acid, gentamicin, tobramycin, mupirocin, rifampicin, tetracycline, trimethoprim)	quantitative	Multisector	Surveillance & Laboratory
103	Measuring hospital antibiotic consumption in EEA countries: comparison of different metrics, 2017 to 2021	Antibiotic consumption rates (DID) by country	quantitative	Human	Stewardship
		Antibiotic consumption rates (DDD per 100 bed-days) by country	quantitative	Human	Stewardship
		Antibiotic consumption rates (DDD per 100 discharges) by country	quantitative	Human	Stewardship
104	Measuring the global response to antimicrobial resistance, 2020-21: a systematic governance analysis of 114 countries	Coordination score by country	quantitative	Multisector	Coordination
		Education score by country	quantitative	Multisector	Workforce
		Accountability score by country	quantitative	Multisector	Accountability
		Participation score by country	quantitative	Multisector	Participation
		Reporting score by country	quantitative	Multisector	Reporting
		Feedback mechanism score by country	quantitative	Multisector	Feedback Mechanisms
		IPC score by country	quantitative	Multisector	Prevention & Control
		Public awareness score by country	quantitative	Multisector	Community Awareness & Enabling Behaviours
		Medicines regulation score by country	quantitative	Multisector	Regulations & Legislation
		AMS score by country	quantitative	Multisector	Stewardship
		Aggregate monitoring and evaluation score by country	quantitative	Multisector	Effectiveness
		Surveillance score by country	quantitative	Multisector	Surveillance & Laboratory
		Overall governance score by country	quantitative	Multisector	Strategic Vision
		Aggregate policy design score by country	quantitative	Multisector	Strategic Vision

		Aggregate implementation tools score by country	quantitative	Multisector	Strategic Vision
		Strategic vision score by country	quantitative	Multisector	Strategic Vision
		Transparency score by country	quantitative	Multisector	Transparency
		Sustainability score by country	quantitative	Multisector	Sustainability
		Effectiveness score by country	quantitative	Multisector	Effectiveness
		R&D for novel products score by country	quantitative	Multisector	Research, Innovation, & Digital Technology
		AMR research score by country	quantitative	Multisector	Research, Innovation, & Digital Technology
		Equity score by country	quantitative	Multisector	Equity
105	Modulation of multidrug-resistant clone success in Escherichia coli populations: a longitudinal, multi-country, genomic and antibiotic usage cohort study	Proportions (% of isolates) of major sequence types (n=10)	quantitative	Human	Surveillance & Laboratory
		Proportion (% of isolates) of ST131 clades (n=6)	quantitative	Human	Surveillance & Laboratory
		Mean relative frequency changes for sequence types over time under a baseline level of DDD (Base DDD) and its multiples	quantitative	Human	Surveillance & Laboratory
		Mean relative frequency changes for ST131 clades A, B, C1, and C2 over time under increasing DDD selection levels	quantitative	Human	Surveillance & Laboratory
		Proportion of isolates with known dfr genes within each major sequence type	quantitative	Human	Surveillance & Laboratory
		Proportion of dfr positive isolates within the four major sequence types (ST69, ST73, ST95, and ST131)	quantitative	Human	Surveillance & Laboratory
		Proportion of isolates that are ciprofloxacin resistant by sequence type (ST) and ST131 clades in the total collection	quantitative	Human	Surveillance & Laboratory
		5 most commonly reported MDR microorganisms by country (E. faecialis, E. coli, K. pneumoniae, P. aeruginosa, S, aureus)	qualitative	Human	Surveillance & Laboratory
		Antimicrobial consumption (DID) for other J01 substances (excl. J01XX)	quantitative	Human	Stewardship

		Antimicrobial consumption (DID) for sulfonamides & trimethoprim	quantitative	Human	Stewardship
		Antimicrobial consumption (DID) for tetracyclines	quantitative	Human	Stewardship
		Antimicrobial consumption (DID) for beta-lactams, penicillins	quantitative	Human	Stewardship
		Antimicrobial consumption (DID) for quinolones	quantitative	Human	Stewardship
		Antimicrobial consumption (DID) for other beta-lactams	quantitative	Human	Stewardship
		Antimicrobial consumption (DID) for macrolides, lincosamides & streptogramins	quantitative	Human	Stewardship
106	Monitoring Antimicrobial Resistance and Drug Usage in the Human and Livestock Sector and Foodborne Antimicrobial Resistance in Six European Countries	Data source by AMU database	qualitative	Human	Reporting
		Does the AMR system (humans) report data to EFSA?	Y/N	Human	Reporting
		Does the AMU database communicate to the EU?	Y/N	Human	Reporting
		Does the AMU system (humans) report data to ESVAC?	Y/N	Human	Reporting
		Data origin by AMR database	qualitative	Multisector	Reporting
		Does the AMU system (livestock) report sales data to ESVAC?	Y/N	Animal	Reporting
		Does the AMU system (livestock) report usage data to ESVAC?	Y/N	Animal	Reporting
		Does the AMR system (livestock) report data to EFSA?	Y/N	Animal	Reporting
		Overlap between country systems for human AMR	quantitative	Human	Coordination
		Overlap between country systems for human AMU	quantitative	Human	Coordination
		Does the AMR database communicate to the EU?	Y/N	Multisector	Reporting
		Does the AMR system (food) report data to EFSA?	Y/N	Environment	Reporting

107	Monitoring progress on Antimicrobial Resistance (AMR) response in the World Health Organization African region: Insights from the Tracking AMR Country Self-Assessment Survey (TrACSS) 2021 results for the human health sector	Awareness and understanding of AMR risks and response	qualitative/ordinal scale answers to the survey analysed and presented quantitatively as n and percentages	Multisector	Transparency
		Adoption of AwaRe categorisation	qualitative/ordinal scale answers to the survey analysed and presented quantitatively as n and percentages	Multisector	Stewardship
		Multisector and One Health coordination	qualitative/ordinal scale answers to the survey analysed and presented quantitatively as n and percentages	Multisector	Stewardship
108	Moving from assessments to implementation: promising practices for strengthening multisectoral antimicrobial resistance containment capacity	ESBL-producing E. coli prevalence (%) by country	quantitative	Human	Surveillance & Laboratory
		Promising practice: implement capacity level-appropriate actions using the WHO Benchmarks for IHR Capacities as an organizing framework	qualitative	Multisector	Strategic Vision
		Promising practice: identify entry points and integrate AMR into other national and global agendas	qualitative	Multisector	Coordination
		Promising practice: improve governance through multisectoral coordination on AMR	qualitative	Multisector	Coordination
		Promising practice: mobilize and diversify funding for AMR containment efforts	qualitative	Multisector	Sustainability
		Capacity for effective multisectoral coordination on AMR by country	quantitative	Multisector	Coordination
		Baseline JEE scores for IPC by country	quantitative	Multisector	Prevention & Control
		Baseline JEE scores for AMS by country	quantitative	Multisector	Stewardship

		Capacity for IPC by country	quantitative	Multisector	Prevention & Control
		Capacity for optimising use of antimicrobial medicines in human, animal health & agriculture	quantitative	Multisector	Stewardship
		Total n of MTaPS-supported action by country	quantitative	Multisector	Stewardship
109	Multidisciplinary and multisectoral coalitions as catalysts for action against antimicrobial resistance: Implementation experiences at national and regional levels	Areas of action and key AMR-related activities/results by country	qualitative	Multisector	Strategic Vision
		Institutional indicators: Number of institutions with active drug and therapeutics committees (DTCs)	quantitative	Human	Stewardship
		National/network indicators: Number of registered medicine outlets	quantitative	Human	Access to Medicines & Health Services
		Institutional indicators: Number of days antibiotics were out of stock	quantitative	Human	Access to Medicines & Health Services
		Institutional indicators: Number of policies on infection control that are displayed in the institution	quantitative	Human	Prevention & Control
		Institutional indicators: Number of audits conducted on AMR	quantitative	Human	Accountability
		Institutional indicators: Number of hospitals with infection control policies and procedures	quantitative	Human	Prevention & Control
		Institutional indicators: d.Number of activities on AMR that are taking place in the institution	quantitative	Multisector	Stewardship
		Institutional indicators: g.Availability of top ten diseases list in the institutions and their treatment guidelines	qualitative	Multisector	Stewardship
		National/network indicators: b.Number of institutions involved in AMR-related activities	quantitative	Multisector	Participation
		National/network indicators: c.Number of hours of media coverage on AMR	quantitative	Multisector	Community Awareness & Enabling Behaviours
		National/network indicators: Number of AMR meetings and activities conducted at regional/national level with relevant people	quantitative	Multisector	Participation
		National/network indicators: Number of focal persons in the network who are reference persons for AMR activities	quantitative	Multisector	Accountability

		National/network indicators: Percentage of functional laboratories that can do culture and sensitivity within the network	quantitative	Multisector	Surveillance & Laboratory
		National/network indicators: Number of information, education, and communication materials that have been distributed	quantitative	Multisector	Community Awareness & Enabling Behaviours
		National/network indicators: Number of research publications on AMR	quantitative	Multisector	Research, Innovation, & Digital Technology
110	Mycoplasma pneumoniae infections, 11 countries in Europe and Israel, 2011 to 2016	Mycoplasma pneumoniae detection methods, % of positive samples and macrolide resistance monitoring by country	quantitative	Human	Surveillance & Laboratory
		Number of Mycoplasma pneumoniae detections by age group and country	quantitative	Human	Surveillance & Laboratory
111	National action plans for antimicrobial resistance and variations in surveillance data platforms	Countries enrolled to GLASS but are inactive	quantitative	Human	Participation
		Countries enrolled and actively reporting to GLASS	quantitative	Human	Participation
		Consistency in presentation and accessibility by surveillance platform	qualitative	Multisector	Feedback Mechanisms/ Surveillance & Laboratory
		Completeness of antimicrobial susceptibility data by surveillance platform	qualitative	Multisector	Feedback Mechanisms/ Surveillance & Laboratory
		Quality of antimicrobial susceptibility data by surveillance platform	qualitative	Multisector	Feedback Mechanisms/ Surveillance & Laboratory
		Consistency of data across key demographic factors by surveillance platform	qualitative	Multisector	Feedback Mechanisms/ Surveillance & Laboratory
		Proposed optimum data set once effective surveillance platform established for demographics of interest	qualitative	Multisector	Feedback Mechanisms/ Surveillance & Laboratory
		Proposed minimum data requirement (to ensure accuracy and consistency) for demographics of interest	qualitative	Multisector	Feedback Mechanisms/ Surveillance & Laboratory
		Proposed minimum data requirement (to ensure accuracy and consistency) for pathogen-antimicrobial combinations	qualitative	Multisector	Feedback Mechanisms/ Surveillance & Laboratory

		Proposed minimum data requirement (to ensure accuracy and consistency) for source of BSI/origin of infection	qualitative	Multisector	Feedback Mechanisms/ Surveillance & Laboratory
		Proposed optimum data set once effective surveillance platform established for pathogen-antimicrobial combinations	qualitative	Multisector	Feedback Mechanisms/ Surveillance & Laboratory
		Proposed optimum data set once effective surveillance platform established for source of BSI/origin of infection	qualitative	Multisector	Feedback Mechanisms/ Surveillance & Laboratory
112	National action plans on antimicrobial resistance in WHO AMR (Latin America): an analysis via a governance framework	% of indicators covered in NAPs by governance area and by country	quantitative	Multisector	Strategic Vision
		Content indicator score of NAP alignment with GAP by country	Ordinal (0-100)	Multisector	Strategic Vision
113	National disparities in the relationship between antimicrobial resistance and antimicrobial consumption in Europe: an observational study in 29 countries	Distribution of antimicrobial types used by country (penicillins, cephalosporins/other beta-lactams, tetracyclines, macrolides/lincosamides/streptogramins, quinolones, sulfonamides/trimethoprim, other)	quantitative	Human	Stewardship
		National antimicrobial consumption rate (DID) by country	quantitative	Human	Stewardship
		National mean AMR rate (%) by country	quantitative	Human	Surveillance & Laboratory
114	OH-EpiCap: A semi-quantitative tool for the evaluation of One Health epidemiological surveillance capacities and capabilities	Dimension 1 (organisation): Supporting documentations	Ordinal (0-4)	Multisector	Strategic Vision
		Dimension 1 (organisation): Shared leadership	Ordinal (0-4)	Multisector	Strategic Vision
		Dimension 1 (organisation): Coordination	Ordinal (0-4)	Multisector	Coordination
		Dimension 1 (organisation): Sectors	Ordinal (0-4)	Multisector	Participation
		Dimension 1 (organisation): Disciplines	Ordinal (0-4)	Multisector	Participation
		Dimension 1 (organisation): Actors	Ordinal (0-4)	Multisector	Participation
		Dimension 1 (organisation): Geographic, populations, hazards	Ordinal (0-4)	Multisector	Social Determinants
		Dimension 1 (organisation): Budget	Ordinal (0-4)	Multisector	Sustainability
		Dimension 1 (organisation): HR	Ordinal (0-4)	Multisector	Sustainability
		Dimension 1 (organisation): Shared resources	Ordinal (0-4)	Multisector	Sustainability

	Dimension 1 (organisation): Training	Ordinal (0-4)	Multisector	Workforce
	Dimension 1 (organisation): Internal evaluation	Ordinal (0-4)	Multisector	Accountability
	Dimension 1 (organisation): External evaluation	Ordinal (0-4)	Multisector	Accountability
	Dimension 1 (organisation): Corrective measures	Ordinal (0-4)	Multisector	Accountability
	Dimension 1 (organisation): Adaptability to changes	Ordinal (0-4)	Multisector	Sustainability
	Dimension 1 (organisation): Common aim	Ordinal (0-4)	Multisector	Coordination
	Dimension 2 (operations): Protocol design	Ordinal (0-4)	Multisector	Strategic Vision
	Dimension 2 (operations): Data collection	Ordinal (0-4)	Multisector	Reporting
	Dimension 2 (operations): Laboratory techniques	Ordinal (0-4)	Multisector	Surveillance & Laboratory
	Dimension 2 (operations): Data wharehouse	Ordinal (0-4)	Multisector	Reporting
	Dimension 2 (operations): Sharing agreement	Ordinal (0-4)	Multisector	Coordination
	Dimension 2 (operations): Data quality	Ordinal (0-4)	Multisector	Reporting
	Dimension 2 (operations): Usefulness	Ordinal (0-4)	Multisector	Effectiveness
	Dimension 2 (operations): FAIR data	Ordinal (0-4)	Multisector	Surveillance & Laboratory
	Dimension 2 (operations): Joint analysis	Ordinal (0-4)	Multisector	Reporting
	Dimension 2 (operations): Sharing techniques	Ordinal (0-4)	Multisector	Coordination
	Dimension 2 (operations): Sharing expertise	Ordinal (0-4)	Multisector	Coordination
	Dimension 2 (operations): Indicators	Ordinal (0-4)	Multisector	Reporting
	Dimension 2 (operations): Internal communication	Ordinal (0-4)	Multisector	Coordination
	Dimension 2 (operations): External communication	Ordinal (0-4)	Multisector	Coordination
	Dimension 2 (operations): Dissemination	Ordinal (0-4)	Multisector	Transparency
	Dimension 2 (operations): Emergence	Ordinal (0-4)	Multisector	Surveillance & Laboratory
	Dimension 3 (impact): Emergence detection	Ordinal (0-4)	Multisector	Surveillance & Laboratory
	Dimension 3 (impact): Improved knowledge	Ordinal (0-4)	Multisector	Effectiveness
	Dimension 3 (impact): Effectiveness	Ordinal (0-4)	Multisector	Effectiveness
	Dimension 3 (impact): Operational cost	Ordinal (0-4)	Multisector	Sustainability

		Dimension 3 (impact): OH team	Ordinal (0-4)	Multisector	Participation
		Dimension 3 (impact): OH network	Ordinal (0-4)	Multisector	Participation
		Dimension 3 (impact): International	Ordinal (0-4)	Multisector	Participation
		Dimension 3 (impact): Strategy	Ordinal (0-4)	Multisector	Strategic Vision
		Dimension 3 (impact): Preparedness	Ordinal (0-4)	Multisector	Strategic Vision
		Dimension 3 (impact): Interventions	Ordinal (0-4)	Multisector	Strategic Vision
		Dimension 3 (impact): Advocacy	Ordinal (0-4)	Multisector	Strategic Vision
		Dimension 3 (impact): Awareness	Ordinal (0-4)	Multisector	Community Awareness & Enabling Behaviours
		Dimension 3 (impact): Research	Ordinal (0-4)	Multisector	Research, Innovation, & Digital Technology
		Dimension 3 (impact): Policy changes	Ordinal (0-4)	Multisector	Effectiveness
		Dimension 3 (impact): Behavioural changes	Ordinal (0-4)	Multisector	Community Awareness & Enabling Behaviours
		Dimension 3 (impact): Health outcomes	Ordinal (0-4)	Multisector	Effectiveness
115	Opinions of veterinarians on antimicrobial use in farm animals in Flanders and the Netherlands	Mean years of respondents' experience in practice by country	quantitative	Animal	Workforce
		Proportion (%) of respondents that are practice owners	quantitative	Animal	Workforce
		Proportion (%) of working time devoted to each animal species (poultry, swine, veal calves, cattle)	quantitative	Animal	Workforce
		Vet AMU attitude: when i think i can prevent diseases with antibiotic treatment, i do not have difficulties with prescribing	5-point Likert scale	Animal	Workforce
		Vet AMU attitude: the possible contribution of veterinary antibiotic use to development of resistance in humans is worrisome	5-point Likert scale	Animal	Workforce
		Vet AMU attitude: last years I have become more aware of the necessity to restrictively prescribe antibiotics	5-point Likert scale	Animal	Workforce
		Vet AMU attitude: it is my goal to reduce antibiotic use in my practice as far as possible	5-point Likert scale	Animal	Workforce

Vet AMU attitude: I (would) support the policy to halve veterinary antibiotic consumption by 2015 compared to 2013	5-point Likert scale	Animal	Workforce
Vet AMU attitude: halving veterinary antibiotic use will be at the expense of animal health and animal welfare	5-point Likert scale	Animal	Workforce
Vet AMU attitude: confronted with a probable bacterial infection, i prefer to immediately start antibiotic treatment to prevent complications	5-point Likert scale	Animal	Workforce
Vet pharmacy attitude: an important motive for me to take up reduction of antimicrobial use is to conserve the privilege to both prescribe and sell veterinary drugs	5-point Likert scale	Animal	Workforce
Vet pharmacy attitude: decoupling prescribing and dispensing veterinary drugs by veterinarians should be executed	5-point Likert scale	Animal	Workforce
Vet pharmacy attitude: farmers will be less motivated to ask for veterinary advices when tariffs for veterinary services will rise	5-point Likert scale	Animal	Workforce
Vet pharmacy attitude: I am able to earn a decent income without earnings from the practice pharmacy	5-point Likert scale	Animal	Workforce
Vet pharmacy attitude: without pharmacy incomes, my hourly tariffs must substantially increase to guarantee a same level of income	5-point Likert scale	Animal	Workforce
Perceived achievable veterinary reduction (%) of AMU in 2015 compared with 2009, by animal species and country	quantitative	Animal	Stewardship
Perceived influence on high AMU: climate in stables is suboptimal, leading to respiratory problems requiring antibiotics	5-point Likert scale	Animal	Workforce
Perceived influence on high AMU: infectious diseases easily spread because biosecurity on farms is unsatisfactorily considered	5-point Likert scale	Animal	Workforce

	Perceived influence on high AMU: for economic reasons, farmers treat sick animals with antibiotics instead of implementing preventative measures	5-point Likert scale	Animal	Workforce
	Perceived influence on high AMU: immunity of the supplied animals is suboptimal, making them susceptible to infectious diseases	5-point Likert scale	Animal	Workforce
	Perceived influence on high AMU: antibiotic treatments are often economically beneficial to treat moderate clinical symptoms and are therefore quickly applied	5-point Likert scale	Animal	Workforce
	Perceived influence on high AMU: the mentality of farmers is to prefer antibiotic treatment instead of accepting small production losses or mortality rates	5-point Likert scale	Animal	Workforce
	Perceived influence on high AMU: general management of farmers fails, leading to infectious diseases	5-point Likert scale	Animal	Workforce
	Perceived influence on high AMU: the very proper registration in each country makes vet AMU seem higher than abroad	5-point Likert scale	Animal	Workforce
	Perceived influence on high AMU: the aim for maximum growth or production make animals sensitive for infections	5-point Likert scale	Animal	Workforce
	Perceived influence on high AMU: feed quality is suboptimal, leading to digestive disorders requiring antibiotics	5-point Likert scale	Animal	Workforce
	Perceived influence on high AMU: financial benefits for vets after prescribing results in higher vet antibiotic prescribing rates	5-point Likert scale	Animal	Workforce
	Perceived contribution to AM reduction: increasing postgraduate education for vets on feed and climate	5-point Likert scale	Animal	Workforce

		Perceived contribution to AM reduction: increasing postgraduate education for vets on infectious diseases prevention	5-point Likert scale	Animal	Workforce
		Perceived contribution to AM reduction: improving biosecurity on farms	5-point Likert scale	Animal	Prevention & Control
		Perceived contribution to AM reduction: increasing efforts to eradicate infectious diseases	5-point Likert scale	Animal	Prevention & Control
		Perceived contribution to AM reduction: increasing education for farmers on prevention of infectious diseases	5-point Likert scale	Animal	Prevention & Control
		Perceived contribution to AM reduction: benchmarking of antibiotic use including sanctioning of high users	5-point Likert scale	Animal	Stewardship
		Perceived contribution to AM reduction: improving the quality of animal feed	5-point Likert scale	Animal	Stewardship
		Perceived contribution to AM reduction: restricting treatments only for diseased animals	5-point Likert scale	Animal	Stewardship
		Perceived contribution to AM reduction: increasing in the use of supportive treatments (like NSAIDS)	5-point Likert scale	Animal	Stewardship
		Perceived contribution to AM reduction: improving communicative skills of vets	5-point Likert scale	Animal	Workforce
		Perceived contribution to AM reduction: introduction of an antibiotic tax	5-point Likert scale	Animal	Regulations & Legislation
		Perceived contribution to AM reduction: increasing the use of complementary treatments (herbs, acids)	5-point Likert scale	Animal	Stewardship
		Perceived contribution to AM reduction: uncoupling the prescribing and dispensing of antibiotics by vets	5-point Likert scale	Animal	Stewardship
116	Out-of-pocket health expenditures and antimicrobial resistance in low-income and middle-income countries: an economic analysis	Hospital bed density when AMR is lower than the median AMR in all isolates	quantitative	Human	Access to Medicines & Health Services
		Hospital bed density when AMR is ≥ the median AMR in all isolates	quantitative	Human	Access to Medicines & Health Services

	Effect of hospital beds /100 pop on AMR predictor	quantitative	Human	Access to Medicines & Health Services
	Proportion of isolates that are resistant predicted for quintiles of out-of-pocket health expenditure in countries that require copayments on medication in the public sector	quantitative	Human	Surveillance & Laboratory
	Proportion of isolates that are resistant predicted for quintiles of out-of-pocket health expenditure in countries that do not require copayments on medication in the public sector	quantitative	Human	Surveillance & Laboratory
	Association between out-of-pocket health expenditure & MRSA	quantitative	Human	Access to Medicines & Health Services
	Association between out-of-pocket health expenditure & E. coli	quantitative	Human	Access to Medicines & Health Services
	Association between out-of-pocket health expenditure & K. pneumoniae	quantitative	Human	Access to Medicines & Health Services
	Association between out-of-pocket health expenditure & Non-typhoidal salmonella	quantitative	Human	Access to Medicines & Health Services
	Association between out-of-pocket health expenditure & Shigella species	quantitative	Human	Access to Medicines & Health Services
	Association between out-of-pocket health expenditure & Fluoroquinolone-resistant N. gonorrhoeae	quantitative	Human	Access to Medicines & Health Services
	Effect of out-of-pocket health expenditure on AMR predictor	quantitative	Multisector	Access to Medicines & Health Services
	Effect of sanitation on AMR predictor	quantitative	Multisector	Equity
	Effect of poverty gap on AMR predictor	quantitative	Multisector	Equity
	Out-of-pocket health expenditure (% of total) when AMR is lower than the median AMR in all isolates	quantitative	Multisector	Access to Medicines & Health Services
	Out-of-pocket health expenditure (% of total) when AMR is ≥ the median AMR in all isolates	quantitative	Multisector	Access to Medicines & Health Services
	Sanitation (% of pop with access to improved facilities) when AMR is lower than the median AMR in all isolates	quantitative	Multisector	Social Determinants/ Prevention & Control

		Sanitation (% of pop with access to improved facilities) when AMR is ≥ the median AMR in all isolates	quantitative	Multisector	Social Determinants/ Prevention & Control
117	Pathogen diversity and antimicrobial resistance transmission of Salmonella enterica serovars Typhi and Paratyphi A in Bangladesh, Nepal, and Malawi: a genomic epidemiological study	Distribution of fluoroquinolone resistance in Salmonella enterica serovars Typhi and Paratyphi A	quantitative	Human	Surveillance & Laboratory
		Distribution of azithromycin resistance in Salmonella enterica serovars Typhi and Paratyphi A	quantitative	Human	Surveillance & Laboratory
118	Patient access in 14 high-income countries to new antibacterials approved by the US Food and Drug Administration, European medicines agency, Japanese pharmaceuticals and medical devices agency, or health Canada, 2010-2020	Median launch lag (days) against number of launches by country	quantitative	Multisector	Research, Innovation, & Digital Technology
		Delay (days) before & after EMA launch approval for 13 recently approved antibacterials with approval from the FDA and EMA by country	quantitative	Multisector	Research, Innovation, & Digital Technology
		US trailing 12-month sales for antibacterials approved 2010-2019 by launch date, sponsor size, n of HIC commercial launches	quantitative	Multisector	Research, Innovation, & Digital Technology
		Approval and commercial launch of new molecular entity antibacterials first approved by FDA, EMA, PMDA, HC by country	qualitative	Multisector	Research, Innovation, & Digital Technology
119	Patterns of dental antibiotic prescribing in 2017: Australia, England, United States, and British Columbia (Canada)	Relative % of total prescription by country: amoxicillin	quantitative	Human	Stewardship
		Relative % of total prescription by country: amoxicillin/clavulanic acid	quantitative	Human	Stewardship
		Relative % of total prescription by country: macrolides	quantitative	Human	Stewardship
		n of antibiotic items dispensed per 1,000 population by country	quantitative	Human	Stewardship
		Relative proportion (n, %) of penicillins dispensed by country	quantitative	Human	Stewardship
		Relative proportion (n, %) of cephalosporins dispensed by country	quantitative	Human	Stewardship
		Relative proportion (n, %) of lincosamides dispensed by country	quantitative	Human	Stewardship

		Relative proportion (n, %) of macrolides dispensed by country	quantitative	Human	Stewardship
		Relative proportion (n, %) of nitroimidazoles dispensed by country	quantitative	Human	Stewardship
		Relative proportion (n, %) of tetracyclines dispensed by country	quantitative	Human	Stewardship
		Relative proportion (n, %) of other antibiotics dispensed by country	quantitative	Human	Stewardship
		Relative % of total prescription by country: cefalexin	quantitative	Human	Stewardship
		Relative % of total prescription by country: clindamycin	quantitative	Human	Stewardship
		Relative % of total prescription by country: metronidazole	quantitative	Human	Stewardship
		Relative % of total prescription by country: phenoxymethylpenicillin	quantitative	Human	Stewardship
		Relative % of total prescription by country: other antibiotics	quantitative	Human	Stewardship
		Rate per 1,000 population of penicillins prescription by country	quantitative	Human	Stewardship
		Rate per 1,000 population of cephalosporins prescription by country	quantitative	Human	Stewardship
		Rate per 1,000 population of lincosamides prescription by country	quantitative	Human	Stewardship
		Rate per 1,000 population of macrolides prescription by country	quantitative	Human	Stewardship
		Rate per 1,000 population of nitroimidazoles prescription by country	quantitative	Human	Stewardship
		Rate per 1,000 population of tetracyclines prescription by country	quantitative	Human	Stewardship
		Rate per 1,000 population of other antibiotics prescription by country	quantitative	Human	Stewardship
120	Perceptions of antimicrobial usage, antimicrobial resistance and policy measures to reduce antimicrobial usage	Mean impact of financial bonus for the use of alternative methods in reducing AMU by country	6-point Likert scale	Multisector	Regulations & Legislation

	in convenient samples of Belgian, French, German, Swedish and Swiss pig farmers	Mean impact of financial compensation for a very large antimicrobial reduction in reducing AMU by country	6-point Likert scale	Multisector	Regulations & Legislation
		Impact from policy measures (respondents' perception)	6-point Likert scale	Multisector	Effectiveness
		Mean level of worries per type of worry and country	quantitative	Multisector	Social Determinants
		Worries about financial/legal issues (respondents' perception)	6-point Likert scale	Multisector	Regulations & Legislation
		Benefits of antimicrobials (respondents' perception)	6-point Likert scale	Multisector	Community Awareness & Enabling Behaviours
		Worries about infectious diseases (respondents' perception)	6-point Likert scale	Multisector	Community Awareness & Enabling Behaviours
		Worries about AMR (respondents' perception)	6-point Likert scale	Multisector	Community Awareness & Enabling Behaviours
		Contribution from veterinarians (respondents' perception)	6-point Likert scale	Animal	Workforce
		Mean years of farming experience by country	quantitative	Animal	Workforce
		Mean impact of less revenue for slaughter pigs with a high antimicrobial level in reducing AMU by country	6-point Likert scale	Animal	Effectiveness
		Mean impact of surveillance of AMU at farms/measures undertaken if AMU is too high in reducing AMU by country	6-point Likert scale	Animal	Surveillance & Laboratory
121	Perspectives on the Regional Strategy for Implementation of National Action Plans on Antimicrobial Resistance in the WHO African Region	Proportion of countries with standalone hospital-based antimicrobial stewardship programmes	quantitative	Human	Stewardship
		Proportion of countries that have integrated AWaRe categorization of antibiotics into National Essential Medicines list or treatment guidelines	quantitative	Human	Stewardship
		Countries that have implemented the campaign strategy on AMR	quantitative (n of countries)	Multisector	Community Awareness & Enabling Behaviours
		Member states with an AMR multisectoral collaboration & coordination committee	Y/N	Multisector	Coordination

n of respondents rating AMR strategy: Satisfied with functioning of AMR MCCC	quantitative (responses originally on a Likert scale - not at all, a little, moderately, quite a bit, extremely)	Multisector	Effectiveness
n of respondents rating AMR strategy: One Health in AMR activities	quantitative (responses originally on a Likert scale - not at all, a little, moderately, quite a bit)	Multisector	Strategic Vision
Rating of AMR strategy by country: AMR committee	Likert scale (0-5: not at all, a little, moderately, quite a bit, extremely)	Multisector	Coordination
Rating of AMR strategy by country: Functional	Likert scale (0-5: not at all, a little, moderately, quite a bit, extremely)	Multisector	Strategic Vision
Rating of AMR strategy by country: Satisfaction	Likert scale (0-5: not at all, a little, moderately, quite a bit, extremely)	Multisector	Strategic Vision
Rating of AMR strategy by country: One Health	Likert scale (0-5: not at all, a little, moderately, quite a bit, extremely)	Multisector	Strategic Vision
Rating of AMR strategy by country: Cognate strategies	Likert scale (0-5: not at all, a little, moderately, quite a bit, extremely)	Multisector	Strategic Vision
Rating of AMR strategy by country: Resources	Likert scale (0-5: not at all, a little,	Multisector	Sustainability

	moderately, quite a bit, extremely)		
Rating of AMR strategy by country: Structures	Likert scale (0-5: not at all, a little, moderately, quite a bit, extremely)	Multisector	Strategic Vision
Rating of AMR strategy by country: Campaign	Likert scale (0-5: not at all, a little, moderately, quite a bit, extremely)	Multisector	Community Awareness & Enabling Behaviours
Rating of AMR strategy by country: AMR prevention	Likert scale (0-5: not at all, a little, moderately, quite a bit, extremely)	Multisector	Prevention & Control
Rating of AMR strategy by country: Learning institutions	Likert scale (0-5: not at all, a little, moderately, quite a bit, extremely)	Multisector	Community Awareness & Enabling Behaviours
Rating of AMR strategy by country: Curricular	Likert scale (0-5: not at all, a little, moderately, quite a bit, extremely)	Multisector	Workforce
Rating of AMR strategy by country: Professional bodies	Likert scale (0-5: not at all, a little, moderately, quite a bit, extremely)	Multisector	Workforce
Rating of AMR strategy by country: Continuous education	Likert scale (0-5: not at all, a little, moderately, quite a bit, extremely)	Multisector	Workforce
Rating of AMR strategy by country: Experts in campaign	Likert scale (0-5: not at all, a little, moderately, quite a bit, extremely)	Multisector	Sustainability

122	Phase I of the Surveillance for Enteric Fever in Asia Project (SEAP): An Overview and Lessons Learned	Blood culture positivity rate (% by country for S Typhi, S Paratyphi, Overall)	quantitative	Human	Surveillance & Laboratory
		Age distribution of enteric fever cases by country for Typhi and Paratyphi	quantitative	Human	Surveillance & Laboratory
		AMR patterns for Typhi and Paratyphi by antibiotic and country (%)	quantitative	Human	Surveillance & Laboratory
123	Policy implementation for methicillin-resistant Staphylococcus aureus in seven European countries: a comparative analysis from 1999 to 2015	MRSA (HA-MRSA) incidence data	quantitative	Human	Surveillance & Laboratory
		Veterinary antibiotic consumption	quantitative	Animal	Stewardship
124	Population-level faecal metagenomic profiling as a tool to predict antimicrobial resistance in Enterobacterales isolates causing invasive infections: an exploratory study across Cambodia, Kenya, and the UK	Relative abundance in metagenome (%) of enterobacterales order by country	quantitative	Human	Surveillance & Laboratory
		Relative abundance in metagenome (%) of enterobacteriaceae family by country	quantitative	Human	Surveillance & Laboratory
		Relative abundance in metagenome (%) of escherichia by country	quantitative	Human	Surveillance & Laboratory
		Relative abundance in metagenome (%) of E. coli by country	quantitative	Human	Surveillance & Laboratory
		Relative abundance in metagenome (%) of klebsiella by country	quantitative	Human	Surveillance & Laboratory
		Relative abundance in metagenome (%) of K. pneumoniae by country	quantitative	Human	Surveillance & Laboratory
		Relative abundance in metagenome (%) of enterobacter by country	quantitative	Human	Surveillance & Laboratory
		Relative abundance in metagenome (%) of salmonella by country	quantitative	Human	Surveillance & Laboratory
		Relative abundance in metagenomic population pools (%) of major antibiotic classes & sub-classes by country	quantitative	Human	Surveillance & Laboratory
		Relative abundance in metagenomic population pools (%) of major antibiotic classes by country	quantitative	Human	Surveillance & Laboratory
		Relative abundance in metagenome (%) of less common enterobacterales families	quantitative	Human	Surveillance & Laboratory

(Morganellaceae, Yersiniaceae, Erwiniaceae) by country			
Relative abundance in metagenome (%) of less common enterobacterales genera and species by country	quantitative	Human	Surveillance & Laboratory
Phenotypic resistance (%) observed in Enterobacterales isolates causing bloodstream and cerebrospinal fluid infection by country	quantitative	Human	Surveillance & Laboratory
Resistant bloodstream and cerebrospinal fluid infections by antibiotic sub-class, by country	quantitative	Human	Surveillance & Laboratory
Proportion of invasive blood & cerebrospinal fluid infections (%) that were enterobacterales order by country	quantitative	Human	Surveillance & Laboratory
Proportion of invasive blood & cerebrospinal fluid infections (%) that were enterobacteriaceae family by country	quantitative	Human	Surveillance & Laboratory
Proportion of invasive blood & cerebrospinal fluid infections (%) that were escherichia by country	quantitative	Human	Surveillance & Laboratory
Proportion of invasive blood & cerebrospinal fluid infections (%) that were E. coli by country	quantitative	Human	Surveillance & Laboratory
Proportion of invasive blood & cerebrospinal fluid infections (%) that were klebsiella by country	quantitative	Human	Surveillance & Laboratory
Proportion of invasive blood & cerebrospinal fluid infections (%) that were K. pneumoniae by country	quantitative	Human	Surveillance & Laboratory
Proportion of invasive blood & cerebrospinal fluid infections (%) that were salmonella by country	quantitative	Human	Surveillance & Laboratory
Proportion of invasive blood & cerebrospinal fluid infections (%) that were enterobacter by country	quantitative	Human	Surveillance & Laboratory
Proportion of invasive blood and cerebrospinal fluid infections (%) that were less common	quantitative	Human	Surveillance & Laboratory

		enterobacterales families (Morganellaceae, Yersiniaceae, Erwiniaceae) by country			
		Proportion of invasive blood and cerebrospinal fluid infections (%) that were less common enterobacterales genera & species by country	quantitative	Human	Surveillance & Laboratory
		Bloodstream & cerebrospinal fluid infections (%) by all genera, by country	quantitative	Human	Surveillance & Laboratory
		Bloodstream & cerebrospinal fluid infections (%) by all species, by country	quantitative	Human	Surveillance & Laboratory
125	Practical Pharmacist-Led Interventions to Improve Antimicrobial Stewardship in Ghana, Tanzania, Uganda and Zambia	Percentage compliance with antimicrobial guidelines in outpatient prescriptions for pneumonia	quantitative	Human	Stewardship
		AMS activity/intervention by country: activities for WAAW 2020	Y/N	Multisector	Community Awareness & Enabling Behaviours
		AMS activity/intervention by country: activities for WAAW 2019	Y/N	Multisector	Community Awareness & Enabling Behaviours
		AMS activity/intervention by country: baseline PPS completed	Y/N	Multisector	Surveillance & Laboratory
		AMS activity/intervention by country: post-intervention PPS completed	Y/N	Multisector	Surveillance & Laboratory
		AMS activity/intervention by country: assessment of institutions' AMS activities using the AMS Checklist	Y/N	Multisector	Stewardship
		AMS activity/intervention by country: CwPAMS app promoted/implemented in institution	Y/N	Multisector	Stewardship
		AMS activity/intervention by country: introduced principles of AWaRe	Y/N	Multisector	Stewardship
		AMS activity/intervention by country: antibiotic guardian pledge-based campaign actively promoted	Y/N	Multisector	Stewardship
		AMS activity/intervention by country: health psychologist input into developing AMS interventions	Y/N	Multisector	Stewardship

		AMS activity/intervention by country: used behaviour change methodology in developing AMS interventions	Y/N	Multisector	Stewardship
		AMS activity/intervention by country: committee established or re-energised responsible for AMS	Y/N	Multisector	Stewardship
		AMS activity/intervention by country: infection management/AMS guidelines developed	Y/N	Multisector	Stewardship
		AMS activity/intervention by country: quality improvement methodology	Y/N	Multisector	Stewardship
		AMS activity/intervention by country: audit training	Y/N	Multisector	Stewardship
		AMS activity/intervention by country: antimicrobial chart development	Y/N	Multisector	Stewardship
126	Preparedness to prescribe antibiotics responsibly: a comparison between final year medical students in France and Sweden	Teaching methods used for antibiotic education	7-point Likert scale (usefulness)	Multisector	Workforce
		Self-reported preparedness on curriculum topics related to prudent antibiotic use by country (% & range)	quantitative	Multisector	Workforce
		Perceived availability and usefulness of teaching methods on antibiotic use by country (% & range)	quantitative	Multisector	Workforce
		Self-reported need for more education on antibiotic use (no more education, more education on prudent antibiotic use, more education on prudent and general antibiotic use, unsure) (%) by country	quantitative	Multisector	Workforce
127	Prevalence of antibiotic prescribing in COVID-19 patients in China and other low- and middle-income countries during the pandemic (December 2019-March 2021): A systematic review and meta-analysis	Prevalence (%) of antibiotic prescribing for China and other LMICs (aggregated)	quantitative	Human	Stewardship
		Prevalence (%) of bacterial infections for China and other LMICs (aggregated)	quantitative	Human	Surveillance & Laboratory
		Prescribing frequency (%) by antibiotic (and WHO AWaRe classification) for China and other LMICs (aggregated)	quantitative	Human	Stewardship

128	Prevalence of antibiotic resistance in Helicobacter pylori: a systematic review and meta-analysis in World Health Organization regions	Pooled resistance prevalence (%) to clarithromycin by country	quantitative	Human	Surveillance & Laboratory
		Pooled resistance prevalence (%) to metronidazole by country	quantitative	Human	Surveillance & Laboratory
		Pooled resistance prevalence (%) to levofloxacin by country	quantitative	Human	Surveillance & Laboratory
129	Preliminary insights on carbapenem resistance in Enterobacteriaceae in high-income and low-/middle-income countries	Prevalence (%) of CRE and CRGs in hospital wastewater	quantitative	Human	Surveillance & Laboratory
		Prevalence (%) of CRE and CRGs in municipal wastewater and WWTP	quantitative	Human	Surveillance & Laboratory
		Prevalence (%) of CRE and CRGs in isolates from patients	quantitative	Human	Surveillance & Laboratory
		Prevalence (%) of CRE and CRGs in hospitals in LMICs	quantitative	Human	Surveillance & Laboratory
		Carbapenem consumption (DID)	quantitative	Human	Surveillance & Laboratory
		Carbapenem resistance genes detected by the sequencing method in wastewater	quantitative	Human	Surveillance & Laboratory
		Co-occurence of CRGS in patients, hospital wastewater and municipal wastewater	Y/N	Human	Surveillance & Laboratory
		Co-occurence of CRE in patients, hospital wastewater and municipal wastewater	Y/N	Human	Surveillance & Laboratory
		Prevalence (%) of CRE and CRGs in hospital wastewater in LMIC	quantitative	Human	Surveillance & Laboratory
		Prevalence (%) of CRE and CRGs in municipal wastewater and WWTPs in LMICs	quantitative	Human	Surveillance & Laboratory
130	Primary care treatment guidelines for skin infections in Europe: congruence with antimicrobial resistance found in commensal Staphylococcus aureus in the community	Congruence of treatment recommendations for skin infections in adults with national commensal S. aureus resistance rate by country (a recommendation is congruent if the resistance rate in S. aureus to that antibiotic is <20%)	qualitative	Human	Stewardship
		Congruence of treatment recommendations for skin infections in children with national commensal S. aureus resistance rate by country (a recommendation is congruent if the resistance rate in S. aureus to that antibiotic is <20%)	qualitative	Human	Stewardship

131	Quantitative and qualitative analysis of antimicrobial usage at farm and flock level on 181 broiler farms in nine European countries	Antimicrobial usage at the flock level	quantitative	Animal	Stewardship
		Indication for treatment	qualitative	Animal	Stewardship
		Treatment incidence (TI) per 100 days at the flock level	quantitative	Animal	Stewardship
		Proportion (%) by antimicrobial class of the amount of antimicrobials used	quantitative	Animal	Stewardship
132	Rapid genomic characterization and global surveillance of Klebsiella using pathogenwatch	Genomes in public collection (n, %) by K. pneumoniae sequence type	quantitative	Human	Surveillance & Laboratory
		Genomes in GHRU collection (n, %) by K. pneumoniae sequence type	quantitative	Human	Surveillance & Laboratory
		Genomes in both collections (n, %) by K. pneumoniae sequence type	quantitative	Human	Surveillance & Laboratory
		n of countries by K. pneumoniae sequence type	quantitative	Human	Surveillance & Laboratory
		Total (%) with a carbapenemase by K. pneumoniae sequence type	quantitative	Human	Surveillance & Laboratory
		Most frequently observed carbapenemases (n, %) by K. pneumoniae sequence type	quantitative	Human	Surveillance & Laboratory
		Total (%) with an ESBL by K. pneumoniae sequence type	quantitative	Human	Surveillance & Laboratory
		Most frequently observed ESBLs (n, %) by K. pneumoniae sequence type	quantitative	Human	Surveillance & Laboratory
		Median n of virulence determinants per genome by K. pneumoniae sequence type	quantitative	Human	Surveillance & Laboratory
		Most frequently observed virulence determinants (n, %) by K. pneumoniae sequence type	quantitative	Human	Surveillance & Laboratory
		Total n of K-Types by K. pneumoniae sequence type	quantitative	Human	Surveillance & Laboratory
		Total n of O-Types by K. pneumoniae sequence type	quantitative	Human	Surveillance & Laboratory
		Klebsiella species distribution (n, %) among public and GHRU genomes	quantitative	Human	Surveillance & Laboratory
		K. pneumoniae genomes (n, %) from the GHRU and public collections with each Kleborate virulence score	quantitative	Human	Surveillance & Laboratory

		Relative frequency (%) of sequence types among K. pneumoniae genomes from the GHRU participant countries	quantitative	Human	Surveillance & Laboratory
		Distribution of carbapenemase genes among 914 K. pneumoniae genomes from the GHRU participant countries	quantitative	Human	Surveillance & Laboratory
		Distribution of O- and K-locus types in K. pneumoniae isolates from the GHRU collection stratified by age	quantitative	Human	Surveillance & Laboratory
133	Reported antibiotic use among patients in the multicenter ANDEMIA infectious diseases surveillance study in sub-saharan Africa	Proportional antibiotic use according to AWaRe classification by country & health facility location for AFDUC syndrome	quantitative	Human	Stewardship
		Proportional antibiotic use according to AWaRe classification by country & health facility location for GI syndrome	quantitative	Human	Stewardship
		Proportional antibiotic use according to AWaRe classification by country & health facility location for RTI syndrome	quantitative	Human	Stewardship
		n of antibiotics (0-4) reported per patient by country	quantitative	Human	Stewardship
		n of different antibiotic substances reported by country and health facility location	quantitative	Human	Stewardship
		n of parenteral antibiotic substances reported by country and health facility location	quantitative	Human	Stewardship
		n of oral antibiotic substances reported by country and health facility location	quantitative	Human	Stewardship
		n of parenteral/oral/other antibiotic substances reported by country and health facility location	quantitative	Human	Stewardship
		Top five most commonly reported antibiotics (%) by country	quantitative	Human	Stewardship
134	resistancebank.org, an open-access repository for surveys of antimicrobial resistance in animals	Resistance rates, n of surveys & total n of isolates by animal type for Campylobacter	quantitative	Animal	Surveillance & Laboratory
		Resistance rates, n of surveys & total n of isolates by animal type for E. coli	quantitative	Animal	Surveillance & Laboratory

		Resistance rates, n of surveys & total n of isolates by animal type for S. aureus	quantitative	Animal	Surveillance & Laboratory
		Resistance rates, n of surveys & total n of isolates by animal type for Salmonella	quantitative	Animal	Surveillance & Laboratory
135	Results from the Survey of Antibiotic Resistance (SOAR) 2014-16 in Bulgaria, Romania, Serbia and Croatia	Susceptibility for S. pneumoniae isolates by country & antimicrobial (CLSI, PK/PD & EUCAST breakpoints)	quantitative	Human	Surveillance & Laboratory
		n of S. pneumoniae isolates at MIC (mg/L) by country & antimicrobial	quantitative	Human	Surveillance & Laboratory
		Susceptibility rates (%) for antimicrobials against all S. pneumoniae according to susceptibility to penicillin	quantitative	Human	Surveillance & Laboratory
		MIC (mg/L) data for H. influenzae isolates by country & antimicrobial	quantitative	Human	Surveillance & Laboratory
		Susceptibility for H. influenzae isolates by country & antimicrobial (CLSI, PK/PD & EUCAST breakpoints)	quantitative	Human	Surveillance & Laboratory
		n of H. influenzae isolates at MIC (mg/L) by country & antimicrobial	quantitative	Human	Surveillance & Laboratory
		MIC breakpoints (mg/L) used for S. pneumoniae (CLSI & EUCAST breakpoints)	quantitative	Human	Surveillance & Laboratory
		MIC breakpoints (mg/L) used for H. influenzae (CLSI & EUCAST breakpoints)	quantitative	Human	Surveillance & Laboratory
		MIC (mg/L) data for S. pneumoniae isolates by country & antimicrobial	quantitative	Human	Surveillance & Laboratory
136	Review of antibiotic use and resistance in food animal production in WHO South-East Asia Region	Antibiotic resistance by antibiotic class in flora, meat and milk by country	Y/N (is data recorded)	Animal	Surveillance & Laboratory
		Antibiotic resistance by antibiotic class in poultry and poultry products by country	Y/N (is data recorded)	Animal	Surveillance & Laboratory
		Antibiotic resistance by antibiotic class in aquaculture by country	Y/N (is data recorded)	Animal	Surveillance & Laboratory
137	Risk factors for the abundance of antimicrobial resistance genes aph(3')-III, erm(B), sul2 and tet(W) in pig and	Relative abundance (ARG copies/16S copies) of aph(3')-III in broilers by country	quantitative	Animal	Surveillance & Laboratory
		Relative abundance (ARG copies/16S copies) of erm(B) in broilers by country	quantitative	Animal	Surveillance & Laboratory

	broiler faeces in nine European countries	Relative abundance (ARG copies/16S copies) of sul2 in broilers by country	quantitative	Animal	Surveillance & Laboratory
		Relative abundance (ARG copies/16S copies) of tet(W) in broilers by country	quantitative	Animal	Surveillance & Laboratory
138	Sales of macrolides, lincosamides, streptogramins, and amoxicillin/clavulanate in the in- and outpatient setting in 10 European countries, 2007-2010	Consumption of erythromycin, clarithromycin, azithromycin and amoxicillin clavulanate by country in DIDs	quantitative	Human	Stewardship
		Use of MLS and amoxicillin clavulanate by country in DIDs	quantitative	Human	Stewardship
		Prescription rates (x1000) of macrolides by diagnostic category and country	quantitative	Human	Stewardship
		Prescription rates (x1000) of lincosamides by diagnostic category and country	quantitative	Human	Stewardship
		Prescription rates (x1000) of amoxicillin clavulanate by diagnostic category and country	quantitative	Human	Stewardship
		Distribution of prescription rates (×1000) of macrolides by country and 10 most frequently assigned diagnostic categories	quantitative	Human	Stewardship
		Distribution of prescription rates (×1000) of AMC by country and 10 most frequently assigned diagnostic categories	quantitative	Human	Stewardship
139	Scoping review of national antimicrobial stewardship activities in eight African countries and adaptable recommendations	Estimated number of pharmacists by country	quantitative	Human	Workforce
		Estimated number of pharmacy technicians by country	quantitative	Human	Workforce
		Key drivers of antimicrobial misuse in community pharmacies	qualitative	Human	Stewardship
		Enrolment to GLASS-AMR by country	Y/N	Human	Participation
		Behavioural barriers (including structural barriers) to tackling AMR	qualitative	Multisector	Social Determinants
		Recommended Priority Actions for AMR	qualitative	Multisector	Stewardship
		NAPs by country	Y/N (is this tool used)	Multisector	Strategic Vision
		Global TrACSS by country	Y/N (is this tool used)	Multisector	Strategic Vision

		WHO WASH FIT by country	Y/N (is this tool used)	Environment	Prevention & Control
		The situation on AMU/AMS in the eight countries for agriculture and animal health	qualitative	Animal	Stewardship
140	Socioeconomic and Governance Factors Disentangle the Relationship between Temperature and Antimicrobial Resistance: A 10-Year Ecological Analysis of European Countries	Weighted mean annual AMR across all countries	quantitative	Human	Surveillance & Laboratory
141	SPiNCAR: A systematic model to evaluate and guide actions for tackling AMR	n of standards & criteria for IPC related to HAI in the framework	quantitative	Human	Prevention & Control
		n of standards & criteria for surveillance & monitoring in the framework	quantitative	Human	Surveillance & Laboratory
		n of standards & criteria for appropriate AMU in the framework	quantitative	Human	Stewardship
		n of standards & criteria for education & training in the framework	quantitative	Human	Workforce
		n of standards & criteria for alliance (public awareness) in the framework	quantitative	Human	Community Awareness & Enabling Behaviours
		n of standards & criteria for implementation in the framework	quantitative	Human	Strategic Vision
		n of standards & criteria for governance in the framework	quantitative	Multisector	Strategic Vision
142	Strengthening multisectoral coordination on antimicrobial resistance: a landscape analysis of efforts in 11 countries	MTaPS-supported activities to finalise and operationalise NAPs-AMR, in collaboration with MSC-AMR bodies (by country)	Y/N	Multisector	Coordination
		MTaPS-supported activities to strengthen leadership, governance and oversight capabilities of MSC-AMR bodies (by country)	Y/N	Multisector	Strategic Vision/ Accountability
		MTaPS-supported activities to build MSC bodies’ technical capacity in AMR, IPC, and AMS (by country)	Y/N	Multisector	Sustainability
		Key stakeholder groups with whom MTaPS has collaborated in country to support actions related	qualitative	Multisector	Participation

		to multisectoral coordination on AMR (by country)			
143	Strengthening strategic management approaches to address antimicrobial resistance in global human health: a scoping review	Coverage of animal disease burden by country GARP report	Y/N	Animal	Surveillance & Laboratory
		Coverage of animal AMU by country GARP report	Y/N	Animal	Stewardship
		Coverage of human disease burden by country GARP report	Y/N	Human	Surveillance & Laboratory
		Coverage of human AMU by country GARP report	Y/N	Human	Stewardship
		Coverage of drug regulation & supply chain by country GARP report	Y/N	Multisector	Regulations & Legislation
		Coverage of demographics by country GARP report	Y/N	Multisector	Social determinants
		Coverage of political context by country GARP report	Y/N	Multisector	Social determinants
		Coverage of economic context by country GARP report	Y/N	Multisector	Social determinants
		Coverage of health systems setting by country GARP report	Y/N	Multisector	Access to Medicines & Health Services
144	Strong correlation between the rates of intrinsically antibiotic-resistant species and the rates of acquired resistance in Gram-negative species causing bacteraemia, EEA, 2016	Proportion of the two most common intrinsically resistant species (P. aeruginosa and Acinetobacter spp.) among the four major Gram-negative species (E. coli, K. pneumoniae, P. aeruginosa and Acinetobacter spp.)	quantitative	Human	Surveillance & Laboratory
		Percentage of selective acquired resistance traits in these species	quantitative	Human	Surveillance & Laboratory
145	Study on the design of a monitoring framework of the EU One Health Action Plans against AMR and Council Recommendation on stepping up EU actions to combat antimicrobial resistance in a One Health approach	Number of Member States whose NAP includes monitoring mechanisms, and the characteristics of their monitoring mechanism	quantitative	Multisector	Feedback Mechanisms
		Amount of funding and extent of technical support provided to third countries to build capacities to address AMR through support for implementation of international standards and action plans, trainings and R&D	quantitative	Multisector	Sustainability

	Number of best practice exchange opportunities in the context of One Health AMR Network meetings or other relevant committees and working groups	quantitative	Multisector	Regulations & Legislation
	Amount of EU funding allocated for translational research and late-stage development of AMR medical countermeasures, including clinical trials for antimicrobials	quantitative	Multisector	Research, Innovation, & Digital Technology
	Extent to which monitoring and reporting of AMR is done in accordance with (Articles 1.4, 3 and 4 of) Commission Implementing Decision (EU) 2020/1729 on the monitoring and reporting of antimicrobial resistance in zoonotic and commensal bacteria	qualitative	Multisector	Regulations & Legislation
	Extent of EU support to the mobilisation of appropriate human and financial resources for the effective implementation of the National Action Plans	mixed	Multisector	Sustainability
	Amount of funds made available (by type of funding instrument) to support research on AMR in the environment	quantitative	Environment	Research, Innovation, & Digital Technology
	Monitoring of the levels of pollution in water caused by antibiotics, antifungal, fungicide and plant protection products is done in accordance with the Watch List under the Water Framework Directive	qualitative	Environment	Prevention & Control
	Levels of AMR in urban waste water as per the recast Urban Wastewater Treatment Directive surveillance obligations for agglomerations of 100,000 population equivalent and above	quantitative	Environment	Surveillance & Laboratory
	Extent to which farms implement measures for good manure and sewage sludge management in each Member State	qualitative	Environment	Stewardship

		Uptake of good evidence-based manure management practices in agriculture in each Member State	qualitative	Environment	Stewardship
		Uptake of good evidence-based sewage sludge management practices in agriculture in each Member State	qualitative	Environment	Stewardship
		Number of Member States whose NAP includes evidence-based measures to prevent, monitor and reduce the spread of AMR in the environment	quantitative	Environment	Prevention & Control
146	Surveillance of antibiotic resistant Escherichia coli in human populations through urban wastewater in ten European countries	Concentration total bacteria (CFU/ml) by country	quantitative	Environment	Surveillance & Laboratory
		Concentration E. coli (CFU/ml) by country	quantitative	Environment	Surveillance & Laboratory
		Growth on cefpodoxime-cloxacillin plates (% of E. coli) by country	quantitative	Environment	Surveillance & Laboratory
		Cefotaxime/ceftazidime (% of isolates) by country	quantitative	Environment	Surveillance & Laboratory
		Total n of E. coli tested by country	quantitative	Environment	Surveillance & Laboratory
		Prevalence of completely susceptible isolates and prevalence of resistance to antibiotics (aminopenicillins, fluoroquinolones, 3G cephalosporins, aminoglycosides) by country	quantitative	Environment	Surveillance & Laboratory
		% of antibiotic resistant E. coli from sampled wastewater compared to antibiotic resistant invasive E. coli reported by hospitals in EARS-Net 2016 (aminopenicillins, fluoroquinolones, 3G cephalosporins, aminoglycosides)	quantitative	Environment	Surveillance & Laboratory
147	Surveillance of Antifungal Resistance in Candidemia Fails to Inform Antifungal Stewardship in European Countries	National reports: Antifungal consumption for C. albicans & non-albicans Candida	Y/N (data availability)	Human	Stewardship
		Epidemiological & observational nationwide studies: Antifungal consumption	Y/N (data availability)	Human	Stewardship
		National reports: S/SDD/R or non-susceptible for C. albicans & non-albicans Candida	Y/N (data availability)	Human	Surveillance & Laboratory
		National reports: MIC distribution for C. albicans & non-albicans Candida	Y/N (data availability)	Human	Surveillance & Laboratory

	Countries providing Candida spp. resistance data in BSIs	quantitative	Human	Reporting
	Countries providing Candida spp. resistance data from mixed samples	quantitative	Human	Reporting
	Countries providing Candida spp. isolates stratified by infection type without resistance data	quantitative	Human	Reporting
	Countries not providing any data on Candida spp. resistance	quantitative	Human	Reporting
	Antifungal & species monitoring (n of national surveillance systems): fluconazole & C. albicans	quantitative	Human	Surveillance & Laboratory
	Antifungal & species monitoring (n of national surveillance systems): fluconazole & C. glabrata	quantitative	Human	Surveillance & Laboratory
	Antifungal & species monitoring (n of national surveillance systems): fluconazole & C. parapsilosis	quantitative	Human	Surveillance & Laboratory
	Antifungal & species monitoring (n of national surveillance systems): fluconazole & C. tropicalis	quantitative	Human	Surveillance & Laboratory
	Antifungal & species monitoring (n of national surveillance systems): itraconazole & C. albicans	quantitative	Human	Surveillance & Laboratory
	Antifungal & species monitoring (n of national surveillance systems): itraconazole & C. glabrata	quantitative	Human	Surveillance & Laboratory
	Antifungal & species monitoring (n of national surveillance systems): itraconazole & C. parapsilosis	quantitative	Human	Surveillance & Laboratory
	Antifungal & species monitoring (n of national surveillance systems): itraconazole & C. tropicalis	quantitative	Human	Surveillance & Laboratory
	Antifungal & species monitoring (n of national surveillance systems): posaconazole & C. albicans	quantitative	Human	Surveillance & Laboratory

Antifungal & species monitoring (n of national surveillance systems): posaconazole & C. glabrata	quantitative	Human	Surveillance & Laboratory
Antifungal & species monitoring (n of national surveillance systems): posaconazole & C. parapsilosis	quantitative	Human	Surveillance & Laboratory
Antifungal & species monitoring (n of national surveillance systems): posaconazole & C. tropicalis	quantitative	Human	Surveillance & Laboratory
Antifungal & species monitoring (n of national surveillance systems): voriconazole & C. albicans	quantitative	Human	Surveillance & Laboratory
Antifungal & species monitoring (n of national surveillance systems): voriconazole & C. glabrata	quantitative	Human	Surveillance & Laboratory
Antifungal & species monitoring (n of national surveillance systems): voriconazole & C. parapsilosis	quantitative	Human	Surveillance & Laboratory
Antifungal & species monitoring (n of national surveillance systems): voriconazole & C. tropicalis	quantitative	Human	Surveillance & Laboratory
Antifungal & species monitoring (n of national surveillance systems): caspofungin & C. albicans	quantitative	Human	Surveillance & Laboratory
Antifungal & species monitoring (n of national surveillance systems): caspofungin & C. glabrata	quantitative	Human	Surveillance & Laboratory
Antifungal & species monitoring (n of national surveillance systems): caspofungin & C. parapsilosis	quantitative	Human	Surveillance & Laboratory
Antifungal & species monitoring (n of national surveillance systems): caspofungin & C. tropicalis	quantitative	Human	Surveillance & Laboratory
Antifungal & species monitoring (n of national surveillance systems): anidulafungin & C. albicans	quantitative	Human	Surveillance & Laboratory

Antifungal & species monitoring (n of national surveillance systems): anidulafungin & C. glabrata	quantitative	Human	Surveillance & Laboratory
Antifungal & species monitoring (n of national surveillance systems): anidulafungin & C. parapsilosis	quantitative	Human	Surveillance & Laboratory
Antifungal & species monitoring (n of national surveillance systems): anidulafungin & C. tropicalis	quantitative	Human	Surveillance & Laboratory
Antifungal & species monitoring (n of national surveillance systems): micafungin & C. albicans	quantitative	Human	Surveillance & Laboratory
Antifungal & species monitoring (n of national surveillance systems): micafungin & C. glabrata	quantitative	Human	Surveillance & Laboratory
Antifungal & species monitoring (n of national surveillance systems): micafungin & C. parapsilosis	quantitative	Human	Surveillance & Laboratory
Antifungal & species monitoring (n of national surveillance systems): micafungin & C. tropicalis	quantitative	Human	Surveillance & Laboratory
Antifungal & species monitoring (n of national surveillance systems): amphotericin & C. albicans	quantitative	Human	Surveillance & Laboratory
Antifungal & species monitoring (n of national surveillance systems): amphotericin & C. glabrata	quantitative	Human	Surveillance & Laboratory
Antifungal & species monitoring (n of national surveillance systems): amphotericin & C. parapsilosis	quantitative	Human	Surveillance & Laboratory
Antifungal & species monitoring (n of national surveillance systems): amphotericin & C. tropicalis	quantitative	Human	Surveillance & Laboratory
National reports: Frequency of reporting (yearly) for C. albicans & non-albicans Candida (national reports)	Y/N (data availability)	Human	Surveillance & Laboratory

		National reports: Data stratification (age, sex, subsetting) for C. albicans & non-albicans Candida	Y/N (data availability)	Human	Surveillance & Laboratory
		National reports: Source of infection for C. albicans & non-albicans Candida	Y/N (data availability)	Human	Surveillance & Laboratory
		National reports: n of patients for C. albicans & non-albicans Candida	Y/N (data availability)	Human	Surveillance & Laboratory
		National reports: BSI incidence (/100 pt, /100 pt/7 days or /100 pt/12-day catheter) for C. albicans & non-albicans Candida	Y/N (data availability)	Human	Surveillance & Laboratory
		National reports: Candidaemia incidence (/100,000 pop) for C. albicans & non-albicans Candida	Y/N (data availability)	Human	Surveillance & Laboratory
		National reports: Total candida isolates for C. albicans & non-albicans Candida	Y/N (data availability)	Human	Surveillance & Laboratory
		National reports: Specific-species identification for C. albicans & non-albicans Candida	Y/N (data availability)	Human	Surveillance & Laboratory
		National reports: Laboratory method for C. albicans & non-albicans Candida	Y/N (data availability)	Multisector	Surveillance & Laboratory
148	Surveillance systems for healthcare-associated infection in high and upper-middle income countries: A scoping review	Target pathogen by country surveillance system: MRSA	Likert scale (mandatory, voluntary, unknown)	Human	Surveillance & Laboratory
		Target pathogen by country surveillance system: MDRP	Likert scale (mandatory, voluntary, unknown)	Human	Surveillance & Laboratory
		Target pathogen by country surveillance system: MDRA	Likert scale (mandatory, voluntary, unknown)	Human	Surveillance & Laboratory
		Target pathogen by country surveillance system: CRE	Likert scale (mandatory, voluntary, unknown)	Human	Surveillance & Laboratory

Target pathogen by country surveillance system: VRE	Likert scale (mandatory, voluntary, unknown)	Human	Surveillance & Laboratory
Frequency of survey used by country HAI national surveillance system	qualitative	Human	Prevention & Control
Target HAI by country surveillance system: CLABSI	Likert scale (mandatory, voluntary, unknown)	Human	Surveillance & Laboratory
Target HAI by country surveillance system: CAUTI	Likert scale (mandatory, voluntary, unknown)	Human	Surveillance & Laboratory
Target HAI by country surveillance system: VAP	Likert scale (mandatory, voluntary, unknown)	Human	Surveillance & Laboratory
Target HAI by country surveillance system: SSI	Likert scale (mandatory, voluntary, unknown)	Human	Surveillance & Laboratory
Target pathogen by country surveillance system: CDI	Likert scale (mandatory, voluntary, unknown)	Human	Surveillance & Laboratory
Target surgery for SSI by country surveillance system: CNS	Y/N	Human	Surveillance & Laboratory
Target surgery for SSI by country surveillance system: CARDIO	Y/N	Human	Surveillance & Laboratory
Target surgery for SSI by country surveillance system: ORTHO	Y/N	Human	Surveillance & Laboratory
Target surgery for SSI by country surveillance system: OBGYN	Y/N	Human	Surveillance & Laboratory

	Target surgery for SSI by country surveillance system: URO	Y/N	Human	Surveillance & Laboratory
	Target surgery for SSI by country surveillance system: ABDO	Y/N	Human	Surveillance & Laboratory
	Definition used for SSI by country surveillance system	qualitative	Human	Surveillance & Laboratory
	Definition used for BSI by country surveillance system	qualitative	Human	Surveillance & Laboratory
	Denominator used for BSI by country surveillance system	quantitative	Human	Surveillance & Laboratory
	Definition used for CAUTI by country surveillance system	qualitative	Human	Surveillance & Laboratory
	Denominator used for CAUTI by country surveillance system	quantitative	Human	Surveillance & Laboratory
	Target population for VAP by country surveillance system	qualitative	Human	Surveillance & Laboratory
	Definition used for VAP by country surveillance system	qualitative	Human	Surveillance & Laboratory
	Parameter used for VAP by country surveillance system	quantitative	Human	Surveillance & Laboratory
	Type of infection for MRSA by country surveillance system	qualitative	Human	Surveillance & Laboratory
	Parameter used for MRSA by country surveillance system	quantitative	Human	Surveillance & Laboratory
	Target population for CDI by country surveillance system	qualitative	Human	Surveillance & Laboratory
	Definition used for CDI by country surveillance system	qualitative	Human	Surveillance & Laboratory
	Denominator used for CDI by country surveillance system	quantitative	Human	Surveillance & Laboratory
	Target population for VRE by country surveillance system	qualitative	Human	Surveillance & Laboratory
	Type of infection for VRE by country surveillance system	qualitative	Human	Surveillance & Laboratory

		Parameter used for VRE by country surveillance system	quantitative	Human	Surveillance & Laboratory
		Target population for CRE by country surveillance system	qualitative	Human	Surveillance & Laboratory
		Type of infection for CRE by country surveillance system	qualitative	Human	Surveillance & Laboratory
		Parameter used for CRE by country surveillance system	quantitative	Human	Surveillance & Laboratory
		Target population for MDRA by country surveillance system	qualitative	Human	Surveillance & Laboratory
		Type of infection for MDRA by country surveillance system	qualitative	Human	Surveillance & Laboratory
		Parameter used for MDRA by country surveillance system	quantitative	Human	Surveillance & Laboratory
		Target population for MDRP by country surveillance system	qualitative	Human	Surveillance & Laboratory
		Type of infection for MDRP by country surveillance system	qualitative	Human	Surveillance & Laboratory
		Parameter used for MDRP by country surveillance system	quantitative	Human	Surveillance & Laboratory
		Follow-up period (days) for SSI by country surveillance system: GENERAL	quantitative	Human	Surveillance & Laboratory
		Follow-up period (days) for SSI by country surveillance system: IMPLANT	quantitative	Human	Surveillance & Laboratory
		Target population for BSI by country surveillance system	qualitative	Human	Surveillance & Laboratory
		Target population for CAUTI by country surveillance system	qualitative	Human	Surveillance & Laboratory
		Operating body of country HAI national surveillance system	qualitative	Human	Surveillance & Laboratory
		Type of survey used by country HAI national surveillance system	qualitative	Human	Surveillance & Laboratory
149	Surveillance systems to monitor antimicrobial resistance in Neisseria gonorrhoeae: a global, systematic	Laboratory procedures used in included surveillance systems	mixed	Multisector	Surveillance & Laboratory

	review, 1 January 2012 to 27 September 2020				
150	Susceptibility to antimicrobials of mastitis-causing Staphylococcus aureus, Streptococcus uberis and Str. dysgalactiae from New Zealand and the USA as assessed by the disk diffusion test	Prevalence of susceptibility of Staphylococcus aureus and Streptococcus species by country and antibiotic	quantitative	Human	Surveillance & Laboratory
		Prevalence of susceptibility of Streptococcus uberis and Str. dysgalactiae by country and antibiotic	quantitative	Human	Surveillance & Laboratory
151	Systematic review and survey of Neisseria gonorrhoeae ceftriaxone and azithromycin susceptibility data in the Asia Pacific, 2011 to 2016	Mapped ranges of alert level MIC (Ceftriaxone), resistance (azithromycin, ciprofloxacin) by country (all as %)	quantitative	Human	Surveillance & Laboratory
		Rates of AMR obtained from Neisseria gonorrhoeae surveillance data by country, year and antibiotic (% rate coded on 1-5 ordinal scale)	quantitative	Human	Surveillance & Laboratory
152	Systematic review of surveillance systems for AMR in Africa	Reporting to GLASS by country	Y/N	Human	Participation
		NRL by country	Y/N	Multisector	Surveillance & Laboratory
		Timeline for NAP development by country NAP	quantitative	Multisector	Strategic Vision
		Multisector/One Health approach by country NAP	qualitative	Multisector	Coordination
		Surveillance activity for AMR by country NAP	qualitative	Multisector	Surveillance & Laboratory
		Frequency of reporting by country AMR surveillance system	quantitative	Multisector	Reporting
		Level of standardisation and harmonisation of procedures among labs included in AMR surveillance system	qualitative	Multisector	Surveillance & Laboratory
		Testing method used by country AMR surveillance system	qualitative	Multisector	Surveillance & Laboratory
		Provision of EQA to local laboratories by country AMR surveillance system	Y/N	Multisector	Surveillance & Laboratory
		Provision of EQA to NRL by country AMR surveillance system	Y/N	Multisector	Surveillance & Laboratory
		Focus/scope by country AMR surveillance system	qualitative	Multisector	Surveillance & Laboratory

		Targeted pop by country AMR surveillance system	qualitative	Multisector	Surveillance & Laboratory
		n of surveillance sites by country AMR surveillance system	quantitative	Multisector	Surveillance & Laboratory
153	Systematic Surveillance and Meta-Analysis of Antimicrobial Resistance and Food Sources from China and the USA	Distribution of n of food samples with AMR isolates in them (%, by isolate, food category and country)	quantitative	Environment	Surveillance & Laboratory
		Individual AMR isolate distributions by country	quantitative	Environment	Surveillance & Laboratory
		Distributions of AMR isolates of foodborne pathogens and indicator bacteria (based on phenotypic AMR detected against the major AM classes in each country)	quantitative	Environment	Surveillance & Laboratory
154	Targets for the reduction of antibiotic use in humans in the Transatlantic Taskforce on Antimicrobial Resistance (TATFAR) partner countries	AMU reduction strategy by country: objective (method(s) for achieving AMU reduction)	qualitative	Multisector	Stewardship
		Are reduction targets for AMU part of a broader national or regional antibiotic resistance strategy or action plan?	Y/N	Multisector	Strategic Vision
		AMU reduction strategy by country: year by which target must be achieved	quantitative	Multisector	Strategic Vision
		Will target(s) be included in a forthcoming NAP?	Y/N	Multisector	Strategic Vision
		Has your country established targets to reduce AMU?	Y/N	Multisector	Strategic Vision
		Does the country have an AMU reduction target for the animal health sector? If so, what is the AMU target (reduction % decrease), and what year must the target be achieved?	Y/N, quantitative	Animal	Strategic Vision
155	The antimicrobial resistance travel tool, an interactive evidence-based educational tool to limit antimicrobial resistance spread	Proportion of AMR worldwide, stratified by country and displayed by ARB type and specimen	quantitative	Human	Surveillance & Laboratory
		Total number of travellers reported visiting WHO regions and corresponding % of AMR bacteria upon return	quantitative	Human	Surveillance & Laboratory

156	The best laid plans?: international governance perspectives in AMR national action plans in Europe	Increase international collaboration	3-point scale (no mention, some, extensively)	Multisector	Participation
		Discussing international collaboration	3-point scale (no mention, some, extensively)	Multisector	Participation
		Structure of NAP (independent of GAP, some GAP recommendations, consistent with GAP)	3-point scale (no mention, some, extensively)	Multisector	Strategic Vision
157	The burden of antimicrobial resistance in the Americas in 2019: a cross-country systematic analysis	DALYs by infectious syndrome associated with AMR (counts, rate/100,000 pop)	quantitative	Human	Surveillance & Laboratory
		Deaths by infectious syndrome attributable to AMR (counts, rate/100,000 pop)	quantitative	Human	Surveillance & Laboratory
		DALYs by infectious syndrome attributable to AMR (counts, rate/100,000 pop)	quantitative	Human	Surveillance & Laboratory
		Deaths by pathogen associated with AMR (counts, rate/100,000 pop)	quantitative	Human	Surveillance & Laboratory
		DALYs by pathogen associated with AMR (counts, rate/100,000 pop)	quantitative	Human	Surveillance & Laboratory
		Deaths by pathogen attributable to AMR (counts, rate/100,000 pop)	quantitative	Human	Surveillance & Laboratory
		DALYs by pathogen attributable to AMR (counts, rate/100,000 pop)	quantitative	Human	Surveillance & Laboratory
		Deaths (count) associated with bacterial antimicrobial resistance by pathogen–drug combination	quantitative	Human	Surveillance & Laboratory
		Deaths (count) attributable to bacterial antimicrobial resistance by pathogen−drug combinations	quantitative	Human	Surveillance & Laboratory
		ASMR per 100,000 person-years for deaths associated with and attributable to AMR	quantitative	Human	Surveillance & Laboratory
		Age–specific mortality rates for deaths attributable to and deaths associated with AMR per country	quantitative	Human	Surveillance & Laboratory

		ASMR associated with and attributable to AMR in relation to the status of NAPs	quantitative	Human	Surveillance & Laboratory
158	The burden of bacterial antimicrobial resistance in the WHO AFR in 2019: a cross-country systematic analysis	Deaths (counts, rate per 100,000) associated with AMR by pathogen	quantitative	Human	Surveillance & Laboratory
		DALYs (counts, per 100,000) associated with AMR by pathogen	quantitative	Human	Surveillance & Laboratory
		Deaths (count) associated with bacterial antimicrobial resistance by pathogen−drug combinations	quantitative	Human	Surveillance & Laboratory
		Deaths that involved infection (counts) by infectious syndrome	quantitative	Human	Surveillance & Laboratory
		Deaths caused by bacteria (counts) by infectious syndrome	quantitative	Human	Surveillance & Laboratory
		Deaths associated with AMR (counts) by infectious syndrome	quantitative	Human	Surveillance & Laboratory
		Deaths associated with AMR (all-age rate per 100000) by infectious syndrome	quantitative	Human	Surveillance & Laboratory
		DALYs associated with AMR (counts) by infectious syndrome	quantitative	Human	Surveillance & Laboratory
		DALYs associated with AMR (all-age rate per 100000) by infectious syndrome	quantitative	Human	Surveillance & Laboratory
		Deaths attributable to AMR (counts) by infectious syndrome	quantitative	Human	Surveillance & Laboratory
		Deaths attributable to AMR (all-age rate per 100000) by infectious syndrome	quantitative	Human	Surveillance & Laboratory
		DALYs attributable to AMR (counts) by infectious syndrome	quantitative	Human	Surveillance & Laboratory
		DALYs attributable to AMR (all-age rate per 100000) by infectious syndrome	quantitative	Human	Surveillance & Laboratory
		Age-standardised mortality associated with and attributable to AMR by country	quantitative	Human	Surveillance & Laboratory
		Deaths (counts, rate per 100,000) attributable to AMR by pathogen	quantitative	Human	Surveillance & Laboratory
		DALYs (counts, per 100,000) attributable to AMR by pathogen	quantitative	Human	Surveillance & Laboratory

		Deaths by infectious syndrome associated with AMR (counts, rate/100,000 pop)	quantitative	Human	Surveillance & Laboratory
		Death counts associated with AMR by pathogen-drug combination	quantitative	Human	Surveillance & Laboratory
		Death counts attributable to AMR by pathogen-drug combination	quantitative	Human	Surveillance & Laboratory
		Age-specific mortality rates for deaths attributable to and deaths associated with AMR per 100,000 person-years by age group and country	quantitative	Human	Surveillance & Laboratory
		Age-standardised mortality rate associated with and attributable to AMR in relation to the status of NAPs	quantitative	Human	Surveillance & Laboratory
159	The European gonococcal antimicrobial surveillance programme (Euro-GASP) appropriately reflects the antimicrobial resistance situation for Neisseria gonorrhoeae in the European Union/European Economic Area	% of isolates with MICs >2 doubling dilutions from the modal MIC (n) against total n of susceptibility tests by country	quantitative	Human	Surveillance & Laboratory
		% of azithromycin resistant isolates and estimates weighted for sexual orientation, gender and age group by country	quantitative	Human	Surveillance & Laboratory
		% of cefixime resistant isolates and estimates weighted for sexual orientation, gender and age group by country	quantitative	Human	Surveillance & Laboratory
		% of ciprofloxacin resistant isolates and estimates weighted for sexual orientation, gender and age group by country	quantitative	Human	Surveillance & Laboratory
160	The European response to control and manage multi- and extensively drug-resistant Neisseria gonorrhoeae	Comms strategy: National publications or communications on MDR N. gonorrhoeae	quantitative (n of countries)	Human	Community Awareness & Enabling Behaviours
		Clinical management: Case definitions for gonorrhoea treatment failure agreed and implemented	quantitative (n of countries)	Human	Prevention & Control
		Clinical management: Gonorrhoea clinical management guidelines reviewed and revised	quantitative (n of countries)	Human	Prevention & Control
		Control strategy & comms: Adoption of national plan to control MDR/XDR gonorrhoea or	quantitative (n of countries)	Human	Prevention & Control

	inclusion in gonorrhoea, STI, sexual health or other relevant strategy			
	Control strategy & comms: n of visits to ECDC Response Plan website	quantitative	Human	Community Awareness & Enabling Behaviours
	Control strategy & comms: n of peer-reviewed publications or other communications on antimicrobial resistant Neisseria gonorrhoeae from Euro-GASP	quantitative	Human	Research, Innovation, & Digital Technology
	National-level surveillance: Proportion of patients who received recommended gonorrhoea treatment	quantitative	Human	Stewardship
	Comms strategy: National communication plan agreed	quantitative (n of countries)	Human	Community Awareness & Enabling Behaviours
	Comms strategy: Fact sheet adjusted and disseminated	quantitative (n of countries)	Human	Community Awareness & Enabling Behaviours
	Clinical management: Recommended culture and AMR testing for cases of suspected treatment failure	mixed	Human	Surveillance & Laboratory
	Clinical management & treatment failure monitoring: n of verified gonorrhoea treatment failures reported to ECDC	quantitative	Human	Surveillance & Laboratory
	Clinical management & treatment failure monitoring: Online reporting template for probable and confirmed gonorrhoea treatment failures developed	Y/N	Human	Reporting
	Clinical management: National gonorrhoea treatment failure reporting/monitoring implemented	Y/N	Human	Reporting
	2012 vs 2017 indicator progress comparison: n of countries participating in Euro-GASP	quantitative (n of countries)	Human	Surveillance & Laboratory
	2012 vs 2017 indicator progress comparison: n of isolates reported through Euro-GASP	quantitative	Human	Surveillance & Laboratory
	2012 vs 2017 indicator progress comparison: n of labs participating in Euro-GASP EQA	quantitative	Human	Surveillance & Laboratory

	2012 vs 2017 indicator progress comparison: n of countries participating in lab training	quantitative (n of countries)	Human	Surveillance & Laboratory
	2012 vs 2017 indicator progress comparison: proportion of countries reporting epidemiological characteristics (mode of transmission) to Euro-GASP	quantitative (n of countries)	Human	Surveillance & Laboratory
	2012 vs 2017 indicator progress comparison: completeness of Euro-GASP data for key epidemiological characteristics	quantitative	Human	Surveillance & Laboratory
	2012 vs 2017 indicator progress comparison: time between Euro-GASP data collection and publication of interim and annual report	quantitative	Human	Surveillance & Laboratory
	2012 vs 2017 indicator progress comparison: n of cases of gonnorrhoea treatment failure reported in EPIS-STI (using the template)	quantitative	Human	Surveillance & Laboratory
	2012 vs 2017 indicator progress comparison: n of publications or communications on MDR-NG	quantitative	Human	Surveillance & Laboratory
	National-level surveillance: Presence of a national representative isolate collection	Y/N	Human	Surveillance & Laboratory
	EEA-level surveillance: Proportion of countries reporting epidemiological characteristics in Euro-GASP	quantitative	Human	Participation
	National-level surveillance: Proportion of all STI clinics (sentinel sites) that have access to culture and antimicrobial susceptibility testing	quantitative	Human	Surveillance & Laboratory
	National-level surveillance: Proportion of all (reported) gonorrhoea cases tested with culture and with antimicrobial susceptibility results available	quantitative	Human	Stewardship
	National-level surveillance: n of countries offering national training modules (laboratory and/or clinical)	quantitative (n of countries)	Human	Workforce
	EEA-level surveillance: n of countries and professionals from these countries participating in the ECDC laboratory training	quantitative	Human	Participation/ Workforce

		Surveillance: National gonococcal antimicrobial surveillance programme in place	quantitative (n of countries)	Multisector	Surveillance & Laboratory
		Surveillance: STI clinic network established (sentinel or other)	quantitative (n of countries)	Multisector	Surveillance & Laboratory
		Surveillance: National platform for sharing of information/data on gonorrhoea AMR established	quantitative (n of countries)	Multisector	Surveillance & Laboratory/Feedback Mechanisms
		Surveillance: Assessment of laboratory capacity performed	quantitative (n of countries)	Multisector	Surveillance & Laboratory
		Surveillance: National training modules (laboratory and/or clinical) available	quantitative (n of countries)	Multisector	Workforce
161	The global burden of enteric fever, 2017-2021: a systematic analysis from the global burden of disease study 2021	ASMR by country		Human	Surveillance & Laboratory
		Age-standardised incidence rate by country		Human	Surveillance & Laboratory
162	The global governance of antimicrobial resistance: a cross-country study of alignment between the global action plan and national action plans	Mean syntactic overlap (%) by region	quantitative	Multisector	Strategic Vision
		Mean content overlap scores (total) by region	quantitative	Multisector	Strategic Vision
		Mean content overlap scores (objectives) by region	quantitative	Multisector	Strategic Vision
		Mean content overlap scores (actions) by region	quantitative	Multisector	Strategic Vision
		Mean syntactic overlap between NAPs in same region (by WHO Region)	quantitative	Multisector	Strategic Vision
		n of observations with syntactic overlap of over 10% between NAPs in same region (by WHO Region)	quantitative	Multisector	Strategic Vision
		n of observations with syntactic overlap of over 50% between NAPs in same region (by WHO Region)	quantitative	Multisector	Strategic Vision
		Mean syntactic overlap between NAPs (by WHO Region)	quantitative	Multisector	Strategic Vision
		n of observations with syntactic overlap of over 10% between NAPs (by WHO Region)	quantitative	Multisector	Strategic Vision
		n of observations with syntactic overlap of over 50% between NAPs (by WHO Region)	quantitative	Multisector	Strategic Vision
		Income per capita by country	quantitative	Multisector	Social Determinants

		Total scores of NAPs based on TSQIs (by country)	quantitative	Multisector	Strategic Vision
		Mean implementation scores (total) by region	quantitative	Multisector	Strategic Vision
163	The impact of inpatient bloodstream infections caused by antibiotic-resistant bacteria in low- and middle-income countries: A systematic review and meta-analysis	ICU admission by bacterial family, gram-positive/negative and most common ABR microbial strains	quantitative	Human	Access to Medicines & Health Services
		LOS by bacterial family, gram-positive/negative and most common ABR microbial strains	quantitative	Human	Access to Medicines & Health Services
		Total excess hospital bed-days cost per ARB BSI patient (USD 2020)	quantitative	Human	Access to Medicines & Health Services
		Total excess ICU admission cost (USD 2020)	quantitative	Human	Access to Medicines & Health Services
		Total excess mortality costs using YPPLL (USD 2020)	quantitative	Human	Access to Medicines & Health Services
		Mortality by bacterial family and gram-positive/negative and most common ABR microbial strains	quantitative	Human	Surveillance & Laboratory
164	The Impact of WHO Essential Medicines Policies on Inappropriate Use of Antibiotics	% of primary care cases receiving antibiotics	quantitative	Human	Stewardship
		% of upper respiratory tract infection cases that received antibiotics	quantitative	Human	Stewardship
		% of acute diarrhoea cases that received antibiotics	quantitative	Human	Stewardship
		% of cases not needing antibiotics that received antibiotics	quantitative	Human	Stewardship
		GNIpc (US$)	quantitative	Multisector	Social Determinants
165	The strategic plan for combating antimicrobial resistance in Gulf Cooperation Council States	Preservation of effective agents for human use: Legislation a. Link the dispensing of all antimicrobial agents to a prescription and unique prescriber/pharmacist identifier	qualitative	Human	Regulations & Legislation
		Preservation of effective agents for human use: Legislation b.Identify a restricted list of antibiotics that will not be available in community pharmacies	qualitative	Human	Regulations & Legislation

Preservation of effective agents for human use: Legislation c. Monitor the trend of the amount of antimicrobial use through a defined daily does/days of therapy (DDD/DOT)	qualitative	Human	Regulations & Legislation
Preservation of effective agents for human use: Legislation d. Develop standards for selecting and using generic antimicrobial agents that meet international standards of quality, safety, and efficacy when granting marketing authorization	qualitative	Human	Regulations & Legislation
Preservation of effective agents for human use: Legislation i. Introduce legal requirements for manufacturers to collect and report data on antimicrobial distribution (including import/export)	qualitative	Human	Regulations & Legislation
Preservation of effective agents for human use: Legislation j. Create economic incentives for the appropriate use of antimicrobials	qualitative	Human	Regulations & Legislation
Preservation of effective agents for human use: Legislation k. Introduce requirements for pharmaceutical companies to comply with national or international codes of practice on promotional activities	qualitative	Human	Regulations & Legislation
Preservation of effective agents for human use: Legislation l. Ensure that national or international codes of practice cover direct-to-consumer advertising, including advertising on the Internet	qualitative	Human	Regulations & Legislation
Preservation of effective agents for human use: Legislation m. Develop and legislate adult and pediatric vaccine programs including, but not limited to, influenza, pneumococcal, and conjugate meningococcal vaccines.	qualitative	Human	Regulations & Legislation
Preservation of effective agents for human use: Legislation n. Restrict the use of the CIA in animals	qualitative	Human	Regulations & Legislation

Preservation of effective agents for human use: Legislation o. Require obligatory prescriptions by licensed veterinarians for all antimicrobials used for disease control in food animals	qualitative	Human	Regulations & Legislation
Preservation of effective agents for human use: Legislation p. Terminate or rapidly phase out the use of antimicrobials for growth promotions	qualitative	Human	Regulations & Legislation
Preservation of effective agents for human use: Legislation q. Create national systems to monitor antimicrobial usage in food animals according to the World Organization for Animal Health (OIE) standards	qualitative	Human	Regulations & Legislation
Preservation of effective agents for human use: Prescriber a. Educate all groups of antimicrobial prescribers and dispensers on the importance of appropriate antimicrobial use and containment of antimicrobial resistance, disease prevention, specifically, immunization programs and infection control, factors that may strongly influence their prescribing habits, such as economic incentives, promotional activities and inducements by the pharmaceutical industry, educating patients on antimicrobial use and the importance of adherence to prescribed treatments	qualitative	Human	Workforce
Preservation of effective agents for human use: Prescriber b. Encourage the development and use of Antimicrobial guidelines and treatment algorithms to foster the appropriate use of antimicrobials	qualitative	Human	Stewardship
Preservation of effective agents for human use: Prescriber c. Improve antimicrobial use by the supervision and support of clinical practices, especially diagnostic and treatment strategies	qualitative	Human	Stewardship

Preservation of effective agents for human use: Prescriber d. Audit prescribing and dispensing practices and utilize peer group or external standard comparisons to provide feedback and endorsements of appropriate antimicrobial prescribing	qualitative	Human	Stewardship
Preservation of effective agents for human use: Prescriber e. Promote targeted undergraduate and postgraduate educational programs on the accurate diagnosis and management of common infections for all health care providers and veterinarians	qualitative	Human	Workforce
Preservation of effective agents for human use: Prescriber f. Establish an Essential Drugs List (EDL) consistent with national and WHO STGs and ensure the accessibility and quality of these drugs	qualitative	Human	Stewardship
Preservation of effective agents for human use: Patient a. Educate patients and the general community on the appropriate use of antimicrobials	qualitative	Human	Community Awareness & Enabling Behaviours
Preservation of effective agents for human use: Patient b. Educate patients on the importance of measures to prevent infection, such as immunization, hygiene, and cough etiquette	qualitative	Human	Community Awareness & Enabling Behaviours
Preservation of effective agents for human use: Patient c. Educate patients on suitable alternatives to antimicrobials for the relief of symptoms, and discourage patient self-initiation of treatment, except in specific circumstances.	qualitative	Human	Community Awareness & Enabling Behaviours
Preservation of effective agents for human use: Patient d. Educate patients on simple measures that may reduce transmission of infection in the household and community, such as hand washing, food hygiene, and so on	qualitative	Human	Community Awareness & Enabling Behaviours

	Preservation of effective agents for human use: Patient e. Encourage appropriate and informed health care seeking behavior	qualitative	Human	Community Awareness & Enabling Behaviours
	Preservation of effective agents for human use: Patient f. Educate students in primary, elementary, and high schools about personal hygiene and how to prevent the spread of infections	qualitative	Human	Community Awareness & Enabling Behaviours
	Early identification of MDR microorganisms: Access to surveillance a. Adopt a unified method to conduct systematic surveillance on MDR organisms from the clinical laboratories through available WHO tools and make the data publicly available for the policy makers and the public	qualitative	Multisector	Surveillance & Laboratory
	Early identification of MDR microorganisms: Access to surveillance b. Adopt a unified method to identify the burden of MDR organisms on HAIs starting by major HAIs such as central line-associatedbloodstream infection (CLABSI), ventilatorassociated pneumonia (VAP), and surgical site infections (SSIs); and make the data publicly available to policy makers and the public	qualitative	Human	Surveillance & Laboratory
	Early identification of MDR microorganisms: Access to surveillance c. Designate or develop reference microbiology laboratory facilities to coordinate effective epidemiologically sound surveillance of antimicrobial resistance among common pathogens in the community, hospitals, and other health care facilities	qualitative	Human	Surveillance & Laboratory
	Early identification of MDR microorganisms: Access to surveillance d. Adopt World Organization for Animal Health (OIE) standards for surveillance in the animal sector	qualitative	Animal	Surveillance & Laboratory

	Early identification of MDR microorganisms: Accountability a. Identify infection prevention and control (IPC) programs in the hospital as separate departments reporting to the highest authority	qualitative	Human	Accountability/Infection, Prevention & Control
	Early identification of MDR microorganisms: Accountability b. Make IPC accountable for leading such activities outlined in (a) and (b) of the previous point	qualitative	Human	Accountability/Infection, Prevention & Control
	Limit spread of resistant pathogens: Humans a. Monitor and improve infection prevention and control practices in healthcare facilities	qualitative	Human	Prevention & Control
	Limit spread of resistant pathogens: Humans b. Establish an inter hospital transfer system that ensures the identification of patients with MDR organisms between hospitals in one country and the transfer of patients between hospitals to and from different GCC countries to encourage proper isolation precautions	qualitative	Human	Prevention & Control
	Limit spread of resistant pathogens: Humans c. Improve the healthcare systems within the GCC countries to ensure minimum infection control requirements while contracting new facilities or renovating old ones	qualitative	Human	Prevention & Control
	Limit spread of resistant pathogens: Animals a. Monitor and improve infection prevention and control practices in animal health, both through the enhanced dissemination and implementation of best practices and the better use of data, diagnostics, and vaccines	qualitative	Animal	Prevention & Control
	Limit spread of resistant pathogens: Animals b. Enhance the availability of veterinarians to prioritize the diagnosis of disease in livestock, poultry, and companion animals and to encourage the early use of appropriate diagnostic	qualitative	Animal	Prevention & Control/Workforce

testing, in particular, bacterial culture and sensitivity testing			
Limit spread of resistant pathogens: Animals c. Make available the use of vaccines in husbandry	qualitative	Animal	Prevention & Control
Limit spread of resistant pathogens: Animals d. Encourage livestock farmers to adhere to government guidance regarding bio-security, animal husbandry and for farm health planning for each of the major farming sectors and to take proactive measures to reduce the risk of disease occurring in their animals under experienced veterinarian supervision	qualitative	Animal	Community Awareness & Enabling Behaviours
Limit spread of resistant pathogens: Animals e. Consider the use of ‘farm assurance schemes' as a mechanism to increase adherence to best husbandry including the isolation of sick animals, testing new stock, and the responsible use of antibiotic by veterinarians only while ensuring animal health and welfare	qualitative	Animal	Strategic Vision
Limit spread of resistant pathogens: Animals f. Encouraging retailers to review their standards for meat and animal products and to set clear specifications concerning biosecurity, antimicrobial stewardship and good husbandry throughout the supply chain for overseas as well as national meat and animal products, working with suppliers and veterinarians to ensure compliance, all in accordance with the World Trade Organization (WTO) guidelines that are set by World Organization for Animal Health (OIE), the Food and Agriculture Organization (FAO), the Codex, and the Intergovernmental Panel on Climate Change (IPPC)	qualitative	Animal	Regulations & Legislation

Limit spread of resistant pathogens: Animals g. Implement standard screening protocols for animal trading	qualitative	Animal	Regulations & Legislation
Understanding magnitude of AMR: Laboratory (human) a. Ensure access to microbiology laboratory services that match the level of those provided by hospitals (e.g., secondary, tertiary)	qualitative	Human	Surveillance & Laboratory
Understanding magnitude of AMR: Laboratory (human) b. Ensure performance and quality assurance of appropriate diagnostic tests, microbial identification, antimicrobial susceptibility tests of key pathogens, and the timely and relevant reporting of results	qualitative	Human	Surveillance & Laboratory
Understanding magnitude of AMR: Laboratory (human) c. Ensure that laboratory data are recorded, preferably in an electronic database, and are used to produce clinically and epidemiologically useful surveillance reports of resistance patterns among common pathogens and infections in a timely manner with feedback to prescribers and to the infection control program	qualitative	Human	Surveillance & Laboratory
Understanding magnitude of AMR: Laboratory (human) d. Adopt an international system of antimicrobial breakpoints interpretation (in microbiology laboratories in the GCC)	qualitative	Human	Surveillance & Laboratory
Understanding magnitude of AMR: IPC (human) a. Characterize the rates of MRSA, vancomycin resistant Enterococci (VRE), Clostridium difficile, and CRE using data from GCC hospitals	qualitative	Human	Surveillance & Laboratory
Understanding magnitude of AMR: IPC (human) b. Characterize and quantify MDR related hospital-acquired infections (HAIs) using	qualitative	Human	Surveillance & Laboratory

	available data on healthcare associated infections routinely collected from GCC hospitals			
	Understanding magnitude of AMR: Laboratory (animals) a. Generate baseline and periodic point prevalence studies on antimicrobial use in animals based on the World Organization for Animal Health (OIE) standards and using data from the Ministry of Agriculture (MoA). In addition to the generation of data, this will ensure compliance with the WHO list of Critically Important Antimicrobials (CIA) for Human Health as described by the WHO Advisory Group on Integrated Surveillance of Antimicrobial Resistance (AGISAR)	qualitative	Animal	Reporting
	Understanding magnitude of AMR: Laboratory (animals) b. Generate baseline and periodic point prevalence data on the presence of antimicrobial residue in food of animal origin with necessary collaboration between the Ministry of Health (MOH), and Drug Authorities	qualitative	Animal	Reporting
	Collaborative research activity: One Health - Identify and provide financial support for research projects in the AMR field as a national priority	qualitative	Multisector	Research, Innovation, & Digital Technology
	Collaborative research activity: One Health - Make academic centers and funding agencies within the GCC countries aware of this priority	qualitative	Multisector	Research, Innovation, & Digital Technology
	Collaborative research activity: One Health - Include all relevant sectors, such as healthcare, food and drug regulatory agencies, veterinarian care, and agriculture and environmental agencies	qualitative	Multisector	Coordination
	Collaborative research activity: One Health - Conduct research that measures the effectiveness of interventions such as drug restriction, guidelines, campaigns, and other strategies	qualitative	Multisector	Research, Innovation, & Digital Technology

166	Towards understanding global patterns of antimicrobial use and resistance in neonatal sepsis: insights from the NeoAMR network	Total n of admissions/year (inborn & outborn) by country	quantitative	Human	Access to Medicines & Health Services
		Mean n of cots by country	quantitative	Human	Access to Medicines & Health Services
		Mean admissions: cots by country	quantitative	Human	Access to Medicines & Health Services
		WTE nurse: cot ratio by country	quantitative	Human	Access to Medicines & Health Services
		Mean % admissions <37 weeks by country	quantitative	Human	Access to Medicines & Health Services
		Mean % admissions <32 weeks by country	quantitative	Human	Access to Medicines & Health Services
		Gram-negative cultures resistant to at least one 3G cephalosporin (n, %) by country	quantitative	Human	Surveillance & Laboratory
		Gram-negative cultures resistant to a carbapenem (n, %) by country	quantitative	Human	Surveillance & Laboratory
		% of Gram-positive cultures resistant to a glycopeptide by country	quantitative	Human	Surveillance & Laboratory
167	Tracking Candidemia Trends and Antifungal Resistance Patterns across Europe: An In-Depth Analysis of Surveillance Systems and Surveillance Studies	Azole resistance (%) by Candida species and country	quantitative	Human	Surveillance & Laboratory
		Echinocandin resistance (%) by Candida species and country	quantitative	Human	Surveillance & Laboratory
		Polyene resistance (%) by Candida species and country	quantitative	Human	Surveillance & Laboratory
		Isolate n growth per year by candida species (Azole)	quantitative	Multisector	Surveillance & Laboratory
		Isolate n growth per year by candida species (Echinocandin)	quantitative	Multisector	Surveillance & Laboratory
		Isolate n growth per year by candida species (Polyene)	quantitative	Multisector	Surveillance & Laboratory
168	Tracking global trends in the effectiveness of antibiotic therapy using the Drug Resistance Index	Frequency of use & resistance (%): aminoglycosides	quantitative	Human	Surveillance & Laboratory, and Stewardship
		Frequency of use & resistance (%): broad-spectrum penicillins	quantitative	Human	Surveillance & Laboratory, and Stewardship

		Frequency of use & resistance (%): carbapenems	quantitative	Human	Surveillance & Laboratory, and Stewardship
		Frequency of use & resistance (%): cephalosporins	quantitative	Human	Surveillance & Laboratory, and Stewardship
		Frequency of use & resistance (%): fluoroquinolones	quantitative	Human	Surveillance & Laboratory, and Stewardship
		Frequency of use & resistance (%): glycopeptides	quantitative	Human	Surveillance & Laboratory, and Stewardship
		Frequency of use & resistance (%): narrow-spectrum penicillins	quantitative	Human	Surveillance & Laboratory, and Stewardship
		Drug Resistance Index by country	Ordinal (0-100)	Human	Surveillance & Laboratory
169	Tracking progress on antimicrobial resistance by the quadripartite country self-assessment survey (TrACSS) in G7 countries, 2017-2023: opportunities and gaps	Capacity to monitor antimicrobial consumption in the human sector by country	Ordinal (0-5)	Human	Stewardship
		Multisector and One Health collaboration/coordination capacity level by country	Ordinal (0-5)	Multisector	Coordination
		Adpotion levels of AWaRe classification	Ordinal (0-5)	Multisector	Stewardship
		Country progress with development of an AMR NAP by country	Ordinal (0-5)	Multisector	Strategic Vision
		Capacity for optimising antimcrobial pesticide such as bactericides and fungicides use in plant production by country	Ordinal (0-5)	Environment	Stewardship
		Capacity to raise awareness & understanding of AMR risks & response by country	Ordinal (0-5)	Multisector	Community Awareness & Enabling Behaviours
		Capacity (to collect data) of national AMR surveillance system by country	Ordinal (0-5)	Multisector	Surveillance & Laboratory
		Capacity level of IPC measures by country	Ordinal (0-5)	Multisector	Prevention & Control
		Adoption levels of AWaRe classification	Ordinal (0-5)	Multisector	Stewardship
		Training & education on AMR in the humans sector by country	Ordinal (0-5)	Human	Workforce
		Training & professional education on AMR for agriculture, food production, food safety and environment sectors by country	Ordinal (0-5)	Environment	Workforce

		Capacity of national AMR surveillance system in food (terrestrial and aquatic animal & plant origin) by country	Ordinal (0-5)	Environment	Surveillance & Laboratory
		Good manufacturing and hygiene practices to reduce development and transmission of AMR in food processing by country	Ordinal (0-5)	Environment	Prevention & Control
		Capacity for optimising antimicrobial pesticide such as bactericides and fungicides use in plant production by country	Ordinal (0-5)	Environment	Stewardship
		Training & education on AMR in the veterinary sector by country	quantitative	Animal	Workforce
170	Trends in methicillin-resistant Staphylococcus aureus in the Gulf Cooperation Council countries: antibiotic resistance, virulence factors and emerging strains	Dominant and novel strains by country	qualitative	Human	Surveillance & Laboratory
		Ranges of virulence elements: prevalence of MRSA (among Staphylococcus aureus isolates, CA-MRSA, SCCmec types, PVL in MRSA, TSST-1, ACME, SCCfus) against prevalence by country (%)	quantitative	Human	Surveillance & Laboratory
		Ranges of AMR resistance rates (%) by country and by antibiotic (n=17)	quantitative	Human	Surveillance & Laboratory
171	Trends in the hospital-sector consumption of the WHO AWaRe Reserve group antibiotics in EEA countries and the United Kingdom, 2010 to 2018	Yearly proportions of Reserve group antibiotics among antibacterials consumed for systemic use, and change over study period by country	quantitative	Human	Stewardship
		Hospital-sector consumption (DDD/1,000 inhabitants/day) of Reserve group antibiotic by country and antibiotic type	quantitative	Human	Stewardship
		Isolates covered by regimen (aminopenicillin, 3-generation cephalosporin, meropenem) and by country (%)	quantitative	Human	Stewardship
		Hospital-sector consumption (DDD/1,000 inhabitants/day) of Reserve group antibiotics and changes in consumption by country and year	quantitative	Human	Stewardship
172	Trends of major antimicrobial resistance phenotypes in enterobacterales and gram-negative	Prevalence of resistance phenotypes (n, %) in bacterial isolates across Italy, Europe, and the World	quantitative	Human	Surveillance & Laboratory

	non-fermenters from ATLAS and EARS-net surveillance systems: Italian vs. European and global data, 2008-2018	Resistance rates (%) in E. coli across Italy, Europe and the World (carbapenem)	quantitative	Human	Surveillance & Laboratory
		Resistance rates (%) in E. coli across Italy, Europe and the World (3GC)	quantitative	Human	Surveillance & Laboratory
		Resistance rates (%) in K. pneumoniae across Italy, Europe and the World (carbapenem)	quantitative	Human	Surveillance & Laboratory
		Resistance rates (%) in K. pneumoniae across Italy, Europe and the World (3GC)	quantitative	Human	Surveillance & Laboratory
		Resistance rates (%) in A. baumannii across Italy, Europe and the World (carbapenem)	quantitative	Human	Surveillance & Laboratory
		Resistance rates (%) in A. baumannii across Italy, Europe and the World (DTR)	quantitative	Human	Surveillance & Laboratory
		Resistance rates (%) in E. cloacae across Italy, Europe and the World (carbapenem)	quantitative	Human	Surveillance & Laboratory
		Resistance rates (%) in P. aeruginosa across Italy, Europe and the World (carbapenem)	quantitative	Human	Surveillance & Laboratory
		Resistance rates (%) in P. aeruginosa across Italy, Europe and the World (MDR)	quantitative	Human	Surveillance & Laboratory
		Resistance rates (%) in P. aeruginosa across Italy, Europe and the World (DTR)	quantitative	Human	Surveillance & Laboratory
173	Understanding antimicrobial resistance from the perspective of public policy: a multinational knowledge, attitude, and perception survey to determine global awareness	Association between demographic variables and 'good personal attitudes and perception'	quantitative	Multisector	Social Determinants
		Association between demographic variables and 'fair personal attitudes and perception'	quantitative	Multisector	Social Determinants
		Cumulative median and mean scores for personal AMR knowledge (HICs)	quantitative	Multisector	Community Awareness & Enabling Behaviours
		Cumulative median and mean scores for personal AMR knowledge (LMICs)	quantitative	Multisector	Community Awareness & Enabling Behaviours
		Cumulative median and mean scores for personal attitude and perception of AMR (HICs)	quantitative	Multisector	Community Awareness & Enabling Behaviours
		Cumulative median and mean scores for personal attitude and perception of AMR (LMICs)	quantitative	Multisector	Community Awareness & Enabling Behaviours
		Cumulative median and mean scores for political KAP (HICs)	quantitative	Multisector	Community Awareness & Enabling Behaviours

		Cumulative median and mean scores for political KAP (LMICs)	quantitative	Multisector	Community Awareness & Enabling Behaviours
		Proportion of questions correctly answered relating to AMR knowledge (HICs)	quantitative	Multisector	Community Awareness & Enabling Behaviours
		Proportion of questions correctly answered relating to AMR knowledge (LMICs)	quantitative	Multisector	Community Awareness & Enabling Behaviours
		Association between demographic variables and 'good knowledge'	quantitative	Multisector	Social Determinants
		Association between demographic variables and 'fair knowledge'	quantitative	Multisector	Social Determinants
		Proportion of personal attitude and perception statements with desired response (HICs)	quantitative	Multisector	Community Awareness & Enabling Behaviours
		Proportion of personal attitude and perception statements with desired response (LMICs)	quantitative	Multisector	Community Awareness & Enabling Behaviours
		Proportion of KAP with desired response (HICs)	quantitative	Multisector	Community Awareness & Enabling Behaviours
		Proportion of KAP with desired response (LMICs)	quantitative	Multisector	Community Awareness & Enabling Behaviours
174	Use of antimicrobials for animals in New Zealand, and in comparison with other countries	Ratio of human: animal use (mg active ingredient/kg biomass)	quantitative	Multisector	Stewardship
		Antimicrobials sold (tonnes), estimated total animal biomass (x10 3 tonnes) and antimicrobial use (mg active ingredient/kg of biomass) for food animals by country	quantitative	Animal	Stewardship
		Antimicrobials sold (tonnes), estimated total animal biomass (x10 3 tonnes) and antimicrobial use (mg active ingredient/kg of biomass) for food animals and horses by country	quantitative	Animal	Stewardship
		Antimicrobials sold (tonnes), estimated total animal biomass (x10 3 tonnes) and antimicrobial use (mg active ingredient/kg of biomass) for all animals by country	quantitative	Animal	Stewardship
		Antimicrobial use (mg active ingredient/kg biomass) for humans by country	quantitative	Animal	Stewardship

		Antimicrobials sold (tonnes), estimated total animal biomass (x10 3 tonnes) and antimicrobial use (mg active ingredient/kg of biomass) by country	quantitative	Animal	Stewardship
175	Use of the WHO Access, Watch, and Reserve classification to define patterns of hospital antibiotic use (AWaRe): an analysis of paediatric survey data from 56 countries	Most frequently (%) reported clinical indications for antibiotic prescribing in children and neonates	quantitative	Human	Stewardship
		% of total antibiotic use in neonates and children by AWaRe classification and country	quantitative	Human	Stewardship
		% of antibiotic use for children with lower respiratory tract infection (by AWaRe classification, WHO Region and country)	quantitative	Human	Stewardship
		% of antibiotic use for neonatal sepsis (by AWaRe classification, WHO Region and country)	quantitative	Human	Stewardship
176	Users' perception of the OH-EpiCap evaluation tool based on its application to nine national antimicrobial resistance surveillance systems	Content theme score (median, min/max): AMU & AMR	quantitative	Multisector	Stewardship
177	Variability in the community consumption of antibiotics: a problem in Europe, Spain and Asturias	Community antibiotic consumption for systemic use by country (DID)	quantitative	Human	Stewardship
178	Variation in antibiotic prescription rates in febrile children presenting to emergency departments across Europe (MOFICHE): A multicentre observational study	Standardised prescription rates by ED for presumed viral, presumed bacterial, and unknown bacterial/viral infections	quantitative	Human	Stewardship
		Antibiotic classes of prescribed antibiotics across EDs (prescription rate)	quantitative	Human	Stewardship
		Inappropriateness of antibiotic prescriptions across EDs (%)	quantitative	Human	Stewardship
		Standardised prescription rates by ED: Antibiotics vs no antibiotics	quantitative	Human	Stewardship
		Standardised prescription rates by ED: Intravenous vs oral	quantitative	Human	Stewardship
		Standardised prescription rates by ED: Broad vs narrow	quantitative	Human	Stewardship

		S. aureus - resistance to methicillin	quantitative	Human	Surveillance & Laboratory
		S. Pneumoniae - resistance to penicillins and macrolides	quantitative	Human	Surveillance & Laboratory
		E.coli - combined resistance to fluoroquinolones, 3G cephalosporins and aminoglycosides	quantitative	Human	Surveillance & Laboratory
		Standardised prescription rates for lower respiratory, otitis media, tonsillitis/pharyngitis and other upper respiratory tract infections	quantitative	Human	Stewardship
		Range of antibiotic prescriptions and broad−spectrum prescriptions per ED for viral, bacterial, and unknown bacterial/viral infections	quantitative	Human	Stewardship
		Hospital antimicrobial resistance rates for S. aureus - resistance to methicillin for invasive isolates	quantitative	Human	Surveillance & Laboratory
179	Veterinary Expert Opinion on Potential Drivers and Opportunities for Changing Antimicrobial Usage Practices in Livestock in Denmark, Portugal, and Switzerland	% of specific diagnoses associated with the application of antimicrobials per country/livestock sector	quantitative	Animal	Stewardship
		% of specific and unspecific diagnosis per organ system/syndrome	quantitative	Animal	Stewardship
		Diseases or syndromes that most frequently lead to the use of antimicrobials, grouped by organ systems, country and livestock sector	quantitative	Animal	Stewardship
		% of reduction of AMU that the veterinary experts believe can be achieved in their country/livestock sector	quantitative	Animal	Prevention & Control
		% of antimicrobial treatments for which an initial sensitivity test was performed in advance by country	quantitative	Animal	Surveillance & Laboratory
		Median and 25–75% interquartile range of reported antimicrobial treatment failures for 2015 by country and species	quantitative	Animal	Surveillance & Laboratory
180	WGS to predict antibiotic MICs for Neisseria gonorrhoeae	Distribution of cefixime MIC (mg/L) with EUCAST breakpoint by country	quantitative	Human	Surveillance & Laboratory
		Distribution of penicillin MIC (mg/L) with EUCAST breakpoint by country	quantitative	Human	Surveillance & Laboratory

		Distribution of azithromycin MIC (mg/L) with EUCAST breakpoint by country	quantitative	Human	Surveillance & Laboratory
		Distribution of ciprofloxacin MIC (mg/L) with EUCAST breakpoint by country	quantitative	Human	Surveillance & Laboratory
		Distribution of tetracycline MIC (mg/L) with EUCAST breakpoint by country	quantitative	Human	Surveillance & Laboratory
181	WHO global antimicrobial resistance surveillance for Neisseria gonorrhoeae 2017-18: a retrospective observational study	Countries reporting ceftriaxone resistance & susceptibility by WHO Region	quantitative	Human	Surveillance & Laboratory
		Countries reporting cefixime resistance & susceptibility by WHO Region	quantitative	Human	Surveillance & Laboratory
		Countries reporting azithromycin resistance & susceptibility by WHO Region	quantitative	Human	Surveillance & Laboratory
		Countries reporting ciprofloxacin resistance & susceptibility by WHO Region	quantitative	Human	Surveillance & Laboratory
		% of isolates with decreased susceptibility or resistance to cefixime reported to WHO GASP/GLASS by country	quantitative	Human	Surveillance & Laboratory
		% of isolates with decreased susceptibility or resistance to ceftriaxone reported to WHO GASP/GLASS by country	quantitative	Human	Surveillance & Laboratory
		% of isolates with decreased susceptibility or resistance to azithromycin reported to WHO GASP/GLASS by country	quantitative	Human	Surveillance & Laboratory
		% of isolates with decreased susceptibility or resistance to ciprofloxacin reported to WHO GASP/GLASS by country	quantitative	Human	Surveillance & Laboratory
182	While we are waiting for the Superbug: constitutional asymmetry and EU governmental policies to combat antimicrobial resistance	Consumption of antimicrobials by humans and food-producing animals (tons), with estimated biomass of the corresponding population in 1,000 tons and consumption (mg/kg biomass)	qualitative, Y/N	Multisector	Stewardship
		Competence (EU/member state) level: a.Use of antimicrobials	qualitative, Y/N	Multisector	Stewardship
		Competence (EU/member state) level: b.Marketisation and selling of antimicrobials	qualitative, Y/N	Multisector	Regulations & Legislation

		Competence (EU/member state) level: c.Innovation and research policy	qualitative, Y/N	Multisector	Research, Innovation, & Digital Technology
		Competence (EU/member state) level: d.Prevention of AMR	qualitative, Y/N	Multisector	Prevention & Control
		Implementation of EU initiatives (hard/soft law): a.Use of antimicrobials	qualitative, Y/N	Multisector	Regulations & Legislation
		Implementation of EU initiatives (hard/soft law): b.Marketisation and selling of antimicrobials	qualitative, Y/N	Multisector	Regulations & Legislation
		Implementation of EU initiatives (hard/soft law): c.Innovation and research policy	qualitative, Y/N	Multisector	Regulations & Legislation
		Implementation of EU initiatives (hard/soft law): d.Prevention of AMR	qualitative, Y/N	Multisector	Regulations & Legislation
		Vet sector - Competence (EU/member state) level: a.Use of antimicrobials	qualitative, Y/N	Animal	Stewardship
		Vet sector - Competence (EU/member state) level: b.Marketisation and selling of antimicrobials	qualitative, Y/N	Animal	Regulations & Legislation
		Vet Sector - Competence (EU/member state) level: c.Prevention of AMR	qualitative, Y/N	Animal	Prevention & Control
		Vet Sector - Implementation of EU initiatives (hard/soft law): a.Use of antimicrobials	qualitative, Y/N	Animal	Regulations & Legislation
		Vet Sector - Implementation of EU initiatives (hard/soft law): b.Marketisation and selling of antimicrobials	qualitative, Y/N	Animal	Regulations & Legislation
		Vet Sector - Implementation of EU initiatives (hard/soft law): c.Prevention of AMR	qualitative, Y/N	Animal	Regulations & Legislation
183	Worldwide antibiotic resistance dynamics: how different is it from one drug-bug pair to another?	Antibiotic resistance rates distribution by country	quantitative	Human	Surveillance & Laboratory
		Antibiotic resistance temporal trends by county and drug-bug pair	quantitative	Human	Surveillance & Laboratory
		Antibiotic resistance rates distribution by infection source	quantitative	Human	Surveillance & Laboratory

Supplemental Data 2. Summary of Grey Literature Indicators

	Study	*Indicator*	*Measurement*	*One Health Sector*	*Subdomain*
1	Global Indicator Framework for SDGs	SDG indicator 3.d.2: median proportion of bloodstream infections due to selected antimicrobial resistant organisms	Quantitative`	Human Health	Surveillance & Laboratory
		SDG indicator 3.b.1: Proportion of the target population covered by all vaccines included in their national program	Quantitative	Human Health	Prevention & Control
		SDG indicator 3.b.3 Proportion of health facilities that have a core set of relevant essential medicines available and affordable on a sustainable basis	Quantitative	Human Health	Access to Medicines and Health Services
2	Implementation of WOAH standards: the observatory annual report 2022	Disease detection, surveillance and diagnosis: Number of diseases that are notifiable at national level per country, amongst WOAH-listed diseases	Quantitative	Animal Health	Reporting
		Disease detection, surveillance and diagnosis: Percentage of WOAH members for which a given disease is notifiable by law at national level	Quantitative	Animal Health	Regulations & Legislation
		Disease detection, surveillance and diagnosis: Percentage of WOAH members for which a given disease is notifiable by law at national level and that have a surveillance system in place for that disease	Quantitative	Animal Health	Regulations & Legislation/Surveillance & Laboratory
		Disease detection, surveillance and diagnosis: Performance of veterinary services regarding surveillance, as assessed by the PVS tool.	Qualitative	Animal Health	Surveillance & Laboratory/Effectiveness
		Emergency preparedness: Number of simulation exercises reported to WOAH	Quantitative	Animal Health	Reporting
		Emergency preparedness: Number of members that reported having a contingency plan	Quantitative	Animal Health	Strategic Vision/Sustainability
		Emergency preparedness: Percentage of members that reported having a contingency plan and a recent simulation exercise for the same diseases	Quantitative	Animal Health	Strategic Vision/Sustainability
		Emergency preparedness: Percentage of members that have an officially recognized disease-free status and that have reported (i) having a contingency plan and (ii) a recent simulation exercise for this disease	Quantitative	Animal Health	Prevention & Control
		Emergency preparedness: Percentage of members	Quantitative	Animal Health	Strategic

	that have a self-declared disease free status and that have reported (i) having a contingency plan and (ii) a recent simulation exercise for this disease (with a focus on ASF, avian influenza and rabies)			Vision/Sustainability/Prevention & Control
	Emergency preparedness: Performance of veterinary services regarding emergency preparedness, as assessed by the PVS tool (emergency funding and emergency response)	Qualitative	Animal Health	Strategic Vision/Effectiveness
	Transparency of Veterinary Services: Time elapsed between the confirmation of a listed disease and the submission of an immediate notification to WOAH (reporting gap)	Quantitative	Animal Health	Transparency/Reporting
	Transparency of Veterinary Services: Confidentiality status of PVS reports	Qualitative	Animal Health	Transparency
	Transparency of Veterinary Services: Performance of Veterinary Services regarding transparency, as assessed by the PVS tool	Qualitative	Animal Health	Transparency/Effectiveness
	Self-declarations of animal health status: Number of self-declarations published by country/compartment/zone	Quantitative	Animal Health	Reporting
	Self-declarations of animal health status: Percentage of members that have self-declared freedom from a disease out of those that reported absence of the same disease in WAHIS	Quantitative	Animal Health	Reporting
	Self-declarations of animal health status: Number of self-declarations that were inactivated and for which recovery was claimed afterwards	Quantitative	Animal Health	Reporting
	Governance and performance of veterinary services (PVS): Percentage of members that have engaged in PVS activities	Quantitative	Animal Health	Participation
	Governance and performance of veterinary services (PVS): Number of requests received for PVS activities	Quantitative	Animal Health	Strategic Vision
	Governance and performance of veterinary services (PVS): Number of PVS activities undertaken	Quantitative	Animal Health	Participation
	Governance and performance of veterinary services (PVS): Number and percentage of members that have had a PVS evaluation/follow-	Quantitative	Animal Health	Strategic Vision

up mission before 2016			
Governance and performance of veterinary services (PVS): Number and percentage of members that have had a PVS evaluation/follow-up mission between 2016 and 2021	Quantitative	Animal Health	Strategic Vision
Governance and performance of veterinary services (PVS): Number and percentage of members that have had only one PVS evaluation between 2006 and 2021	Quantitative	Animal Health	Strategic Vision
Governance and performance of veterinary services (PVS): Distribution of the Levels of Advancement for each Critical competency	Quantitative	Animal Health	Strategic Vision
Governance and performance of veterinary services (PVS): Average levels of advancement for each critical category	Quantitative	Animal Health	Strategic Vision
Governance and performance of veterinary services (PVS): Number of members that have recieved a PVS mission	Quantitative	Animal Health	Participation
Movement control inside countries/territories and precautions at borders: Members declaring that they conduct movement control inside the territory and/or precautions at borders as control measures, in WAHIS six-monthly reports	Y/N	Animal Health	Prevention & Control
Movement control inside countries/territories and precautions at borders: Performance of veterinary services regarding movement controls, assessed by the PVS tool	Y/N	Animal Health	Prevention & Control
Movement control inside countries/territories and precautions at borders: Performance of veterinary services regarding movement control, of members that reported implementing such measures in WAHIS	Y/N	Animal Health	Prevention & Control
WTO notifications: Number of WTO notifications having an effect on trade that involve animal diseases	Quantitative	Animal Health	Effectiveness
WTO notifications: Number of animal-health related disputes filed with the WTO	Quantitative	Animal Health	Reporting
WTO notifications: Performance of Veterinary Services regarding access to trade and interaction	Qualitative	Animal Health	Effectiveness

	with stakeholders, including the TWO, as assessed by the PVS Tool			
	Zoning and compartmentalisation: Number and percentage of members reporting zoning as a control measure in their WAHIS six-monthly reports	Quantitative	Animal Health	Reporting
	Zoning and compartmentalisation: Average number of diseases for which zoning has been reported as a control measure	Quantitative	Animal Health	Reporting
	Zoning and compartmentalisation: Number of members that have reported the presence of a given disease/infection/suspected disease limited to one or more zones in their WAHIS six-monthly reports	Quantitative	Animal Health	Reporting
	Zoning and compartmentalisation: Percentage of members that have reported the presence of a disease/infection/suspected disease limited to one or more zones and that applied zoning as a control measure	Quantitative	Animal Health	Reporting
	Zoning and compartmentalisation: Percentage of members that apply movement control within their territory, of those that apply zoning as a control measure for a given disease	Quantitative	Animal Health	Reporting
	Zoning and compartmentalisation: Number of members that have reported the presence of a given disease/infection/suspected disease limited to one or more zones and that apply the key control measures and that have self-declared freedom or have official recognition for that disease in a zone	Quantitative	Animal Health	Reporting
	Zoning and compartmentalisation: Number of members reporting compartmentalization as part of their disease control measrues in their WAHIS six-monthly reports	Quantitative	Animal Health	Reporting
	Zoning and compartmentalisation: Performance of veterinary services regarding zoning and compartmentalization, as assessed by the PVS tool	Qualitative	Animal Health	Prevention & Control/Effectiveness
	Zoning and compartmentalisation: Number of WOAH members reporting regionalization related to animal health in WTO annual reports on	Quantitative	Animal Health	Reporting

		implementation of regionalization			
		Number of countries in which the use of medically important antimicrobials for growth promotion is eliminated.	Quantitative	Animal Health	Reporting
		Number of outputs promoting concrete commitments to halting non-veterinary medical use of antimicrobial agents.	Quantitative	Animal Health	Reporting
		Number of countries with national targets for responsible and sustainable antimicrobial use in food systems.	Quantitative	Animal Health	Reporting
		Antimicrobial use and antimicrobial resistance: Number of members that submitted their annual report on the use of antimicrobials in animals to WOAH	Quantitative	Animal Health	Reporting
		Antimicrobial use and antimicrobial resistance: Number of members that provided quantitative information on the use of antimicrobials in animals to WOAH in their annual report	Quantitative	Animal Health	Reporting
		Antimicrobial use and antimicrobial resistance: Number of WOAH members using antimicrobial agents as growth promoters	Quantitative	Animal Health	Stewardship
		Antimicrobial use and antimicrobial resistance: Number of WOAH members using growth promoters that are listed by the WHO as critically important for human medicine	Quantitative	Animal Health	Stewardship
		Antimicrobial use and antimicrobial resistance: Number of WOAH members using growth promoters that are listed by WOAH as veterinary critically important antimicrobial agents	Quantitative	Animal Health	Stewardship
3	Joint Monitoring Programme for Water Supply, Sanitation and Hygiene (JMP)	Health care facilities: Coverage of water services in health care facilities	Quantitative	Human Health	Prevention & Control / Social Determinants
		Health care facilities: Population using health care facilities without basic water services	Quantitative	Human Health	Prevention & Control / Social Determinants
		Health care facilities: Improved water on premises in hospitals	Quantitative	Human Health	Prevention & Control / Social Determinants
		Health care facilities: Improved water available when needed in non-hospitals	Quantitative	Human Health	Prevention & Control
		Health care facilities: Coverage of sanitation services in health care facilities	Quantitative	Human Health	Prevention & Control

	Health care facilities: Population using health care facilities with no sanitation services	Quantitative	Human Health	Prevention & Control
	Health care facilities: Improved sanitation facilities accessible to those with limited mobility	Quantitative	Human Health	Prevention & Control
	Health care facilities: Improved sanitation facilities for menstrual hygiene management	Quantitative	Human Health	Prevention & Control
	Health care facilities: Coverage of hygiene services in health care facilities	Quantitative	Human Health	Prevention & Control
	Health care facilities: Population using health care facilities without basic hygiene services	Quantitative	Human Health	Prevention & Control
	Health care facilities: Hand hygiene facilities at points of care in non-hospitals	Quantitative	Human Health	Prevention & Control
	Health care facilities: Handwashing facilities with water and soap at toilets in hospitals	Quantitative	Human Health	Prevention & Control
	Health care facilities: Coverage of waste management services in health care facilities	Quantitative	Human Health	Prevention & Control
	Health care facilities: Population using health care facilities without a basic waste management service in hospitals	Quantitative	Human Health	Prevention & Control
	Health care facilities: Segregation of waste in health care facilities	Quantitative	Human Health	Prevention & Control
	Health care facilities: Treatment and disposal of waste in health care facilities	Quantitative	Human Health	Prevention & Control
	Health care facilities: Coverage of environmental cleaning services in health care facilities	Quantitative	Human Health	Prevention & Control
	Health care facilities: Coverage of basic environmental cleaning services in non-hospitals	Quantitative	Human Health	Prevention & Control
	Health care facilities: Availability of cleaning protocols in health care facilities	Quantitative	Human Health	Prevention & Control
	Health care facilities: Training of staff on environmental cleaning	Quantitative	Human Health	Social Determinants / Prevention & Control
	Households: Rural and urban drinking water service levels	Quantitative	Environment	Social Determinants / Prevention & Control
	Households: Basic safely managed drinking water coverage	Quantitative	Environment	Social Determinants / Prevention & Control
	Households: Subnational inequalities in basic drinking water services	Quantitative	Environment	Social Determinants / Prevention & Control
	Households: Progress towards universal basic water services by 2030	Qualitative	Environment	Social Determinants

		Households: Rural and urban sanitation service levels	Quantitative	Environment	Social Determinants
		Households: Basic and safely managed sanitation coverage by country	Quantitative	Environment	Social Determinants
		Households: Change in open defecation rates by country	Quantitative	Environment	Social Determinants
		Households: Trends in sewer, septic tank and latrine coverage	Quantitative	Environment	Social Determinants
		Households: Population with no hygiene service	Quantitative	Environment	Social Determinants
		Households: Subnational inequalities in hygiene service	Quantitative	Environment	Social Determinants
		Households: Population lacking basic hygiene	Quantitative	Environment	Social Determinants
		Households: hygiene service levels (% coverage)	Quantitative	Environment	Stewardship
4	GLASS-AMR	Antimicrobial use by class: antibiotics (ATC class J01, A07AA, P01AB) (DDD/1,000 inhabitants per day)	Quantitative	Human Health	Stewardship
		Antimicrobial use by class: antifungals (ATC class JO2 'antimycotics for systemic use' and ATC class D01B 'antifungals for systemic use') (DDD/1,000 inhabitants per day)	Quantitative	Human Health	Stewardship
		Use of antibiotics (ATC class J01, A07AA, P01AB) by AWaRe classification (relative use %)	Quantitative	Human Health	Stewardship
		Use of antibiotics (ATC class J01, A07AA, P01AB) by AWaRe classification (DID)	Quantitative	Human Health	Stewardship
		Use of antibiotics (ATC class J01, A07AA, P01AB) by pharmacological subgroups (ATC3) (Relative use %)	Quantitative	Human Health	Stewardship
		Use of antibiotics (ATC class J01, A07AA, P01AB) by pharmacological subgroups (ATC3) (DID)	Quantitative	Human Health	Stewardship
		Use of antibiotics (ATC class J01, A07AA, P01AB) by chemical subgroups (ATC4) (Relative use %)	Quantitative	Human Health	Stewardship
		Use of antibiotics (ATC class J01, A07AA, P01AB) by chemical subgroups (ATC4) (DID)	Quantitative	Human Health	Stewardship
		Use of antibiotics (ATC classes J01, A07AA, P01AB) by route of administration (oral, parenteral, inhalation, rectal)	Qualitative	Human Health	Surveillance & Laboratory

	GLASS-AMR implementation indicator: national coordination centre (available, in progress, not available, no data)	Ordinal	Human Health	Transparency
	GLASS-AMR implementation indicator: national reference library (available, in progress, not available, no data)	Ordinal	Human Health	Transparency
	GLASS-AMR implementation indicator: external quality assurance of national reference library (available, in progress, not available, no data)	Ordinal	Human Health	Transparency
	GLASS-AMR implementation indicator: external quality assurance of all libraries reporting AST to GLASS (available, in progress, not available, no data)	Ordinal	Human Health	Surveillance & Laboratory
	GLASS-AMR implementation indicator: international standards for AST (available, in progress, not available, no data)	Ordinal	Human Health	Reporting
	CTA participation in GLASS-AMU	Y/N	Human Health	Participation
	CTAs reporting data to GLASS-AMR: bacteriologically confirmed infections (overall)	Quantitative	Human Health	Reporting
	CTAs reporting data to GLASS-AMR: AST for 80+% of bacteriologically confirmed infections (overall)	Quantitative and categorical	Human Health	Surveillance & Laboratory
	CTAs reporting data to GLASS-AMR: bacteriologically confirmed infections (bloodstream)	Quantitative	Human Health	Reporting
	CTAs reporting data to GLASS-AMR: AST for 80+% of bacteriologically confirmed infections (bloodstream)	Quantitative	Human Health	Surveillance & Laboratory
	CTAs reporting data to GLASS-AMR: bacteriologically confirmed infections (gastrointestinal)	Quantitative	Human Health	Reporting
	CTAs reporting data to GLASS-AMR: AST for 80+% of bacteriologically confirmed infections (gastrointestinal)	Quantitative	Human Health	Surveillance & Laboratory
	CTAs reporting data to GLASS-AMR: bacteriologically confirmed infections (gonorrhoea)	Quantitative	Human Health	Reporting
	CTAs reporting data to GLASS-AMR: AST for	Quantitative	Human Health	Surveillance & Laboratory

	80+% of bacteriologically confirmed infections (gonorrhoea)			
	CTAs reporting data to GLASS-AMR: bacteriologically confirmed infections (urinary tract)	Quantitative	Human Health	Reporting
	CTAs reporting data to GLASS-AMR: AST for 80+% of bacteriologically confirmed infections (urinary tract)	Quantitative	Human Health	Surveillance & Laboratory
	Data contextualization: type of AMU data	?	Human Health	Surveillance & Laboratory
	Data contextualization: Levels and sectors of health covered	Qualitative	Human Health	Reporting
	Data contextualization: % UN population covered by data	Quantitative	Human Health	Reporting
	Total number of bacteriologically confirmed infections (bloodstream)	Quantitative	Human Health	Surveillance & Laboratory
	Number of bacteriologically confirmed infections with AST (bloodstream)	Quantitative	Human Health	Surveillance & Laboratory
	Total number of bacteriologically confirmed infections (gastrointestinal)	Quantitative	Human Health	Surveillance & Laboratory
	Number of bacteriologically confirmed infections with AST (gastrointestinal)	Quantitative	Human Health	Surveillance & Laboratory
	Total number of bacteriologically confirmed infections (gonorrhoea)	Quantitative	Human Health	Surveillance & Laboratory
	Number of bacteriologically confirmed infections with AST (gonorrhoea)	Quantitative	Human Health	Surveillance & Laboratory
	Total number of bacteriologically confirmed infections (urinary tract)	Quantitative	Human Health	Surveillance & Laboratory
	Number of bacteriologically confirmed infections with AST (urinary tract)	Quantitative	Human Health	Surveillance & Laboratory
	Median (IQR) BCIs with AST per million population (gastrointestinal)	Quantitative	Human Health	Surveillance & Laboratory
	Median (IQR) BCIs with AST per million population (gonorrhoea)	Quantitative	Human Health	Surveillance & Laboratory
	Median (IQR) BCIs with AST per million population (urinary tract)	Quantitative	Human Health	Surveillance & Laboratory
	Median (IQR) BCIs with AST per million population (bloodstream)	Quantitative	Human Health	Surveillance & Laboratory
	Total BCIs per million population	Quantitative	Human Health	Surveillance & Laboratory

	Absolute number of BCIs (total)	Quantitative	Human Health	Surveillance & Laboratory
	Total BCIs with AST per million population (total)	Quantitative	Human Health	Surveillance & Laboratory
	Absolute number of BCIs with AST (total)	Quantitative	Human Health	Surveillance & Laboratory
	% of BCIs with AST (total)	Quantitative	Human Health	Surveillance & Laboratory
	Bloodstream infections: Total Acinetobacter spp. BCIs per million population	Quantitative	Human Health	Surveillance & Laboratory
	Bloodstream infections: Absolute number of Acinetobacter spp. BCIs	Quantitative	Human Health	Surveillance & Laboratory
	Bloodstream infections: Total Acinetobacter spp. BCIs with AST (Carbapenems) per million population	Quantitative	Human Health	Surveillance & Laboratory
	Bloodstream infections: Absolute number of Acinetobacter spp. BCIs with AST (Carbapenems)	Quantitative	Human Health	Surveillance & Laboratory
	Bloodstream infections: % of Acinetobacter spp. BCIs with AST	Quantitative	Human Health	Surveillance & Laboratory
	Bloodstream infections: Total Escherichia coli BCIs per million population	Quantitative	Human Health	Surveillance & Laboratory
	Bloodstream infections: Absolute number of Escherichia coli BCIs	Quantitative	Human Health	Surveillance & Laboratory
	Bloodstream infections: Total Escherichia coli BCIs with AST (Carbapenems) per million population	Quantitative	Human Health	Surveillance & Laboratory
	Bloodstream infections: Absolute number of Escherichia coli BCIs with AST (Carbapenems)	Quantitative	Human Health	Surveillance & Laboratory
	Bloodstream infections: % of Escherichia coli BCIs with AST (Carbapenems)	Quantitative	Human Health	Surveillance & Laboratory
	Bloodstream infections: Total Escherichia coli BCIs with AST (Third-generation cephalosporins) per million population	Quantitative	Human Health	Surveillance & Laboratory
	Bloodstream infections: Absolute number of Escherichia coli BCIs with AST (third-generation cephalosporins)	Quantitative	Human Health	Surveillance & Laboratory
	Bloodstream infections: % Escherichia coli BCIs with AST (third-generation cephalosporins)	Quantitative	Human Health	Surveillance & Laboratory
	Bloodstream infections: Total Klebsiella pneumoniae BCIs per million population	Quantitative	Human Health	Surveillance & Laboratory
	Bloodstream infections: Absolute number of	Quantitative	Human Health	Surveillance & Laboratory

Klebsiella pneumoniae BCIs			
Bloodstream infections: Total Klebsiella pneumoniae BCIs with AST (Carbapenems) per million population	Quantitative	Human Health	Surveillance & Laboratory
Bloodstream infections: Absolute number of Klebsiella pneumoniae BCIs with AST (Carbapenems)	Quantitative	Human Health	Surveillance & Laboratory
Bloodstream infections: % of Klebsiella pneumoniae BCIs with AST (Carbapenems)	Quantitative	Human Health	Surveillance & Laboratory
Bloodstream infections: Total Klebsiella pneumoniae BCIs with AST (third-generation cephalosporins) per million population	Quantitative	Human Health	Surveillance & Laboratory
Bloodstream infections: Absolute number of Klebsiella pneumoniae BCIs with AST (third-generation cephalosporins)	Quantitative	Human Health	Surveillance & Laboratory
Bloodstream infections: % of Klebsiella pneumoniae BCIs with AST (third-generation cephalosporins)	Quantitative	Human Health	Surveillance & Laboratory
Bloodstream infections: Total Salmonella spp. BCIs per million population	Quantitative	Human Health	Surveillance & Laboratory
Bloodstream infections: Absolute number of Salmonella spp. BCIs per million population	Quantitative	Human Health	Surveillance & Laboratory
Bloodstream infections: Total Salmonella spp. BCIs with AST (Fluoroquinolones) per million population	Quantitative	Human Health	Surveillance & Laboratory
Bloodstream infections: Absolute number of Salmonella spp. BCIs with AST (Fluoroquinolones)	Quantitative	Human Health	Surveillance & Laboratory
Bloodstream infections: % of Salmonella spp. BCIs with AST (Fluoroquinolones)	Quantitative	Human Health	Surveillance & Laboratory
Bloodstream infections: Total Staphylococcus aureus BCIs per million population	Quantitative	Human Health	Surveillance & Laboratory
Bloodstream infections: Absolute number of Staphylococcus aureus BCIs per million population	Quantitative	Human Health	Surveillance & Laboratory
Bloodstream infections: Total Staphylococcus aureus BCIs with AST (Methicillin-resistance) per million population	Quantitative	Human Health	Surveillance & Laboratory
Bloodstream infections: Absolute number of	Quantitative	Human Health	Surveillance & Laboratory

	Staphylococcus aureus. BCIs with AST (Methicillin-resistance)			
	Bloodstream infections: % of Staphylococcus aureus BCIs with AST (Methicillin-resistance)	Quantitative	Human Health	Surveillance & Laboratory
	Bloodstream infections: Total Streptococcus pneumoniae aureus BCIs per million population	Quantitative	Human Health	Surveillance & Laboratory
	Bloodstream infections: Absolute number of Streptococcus pneumoniae BCIs per million population	Quantitative	Human Health	Surveillance & Laboratory
	Bloodstream infections: Total Streptococcus pneumoniae s BCIs with AST (Penicillins) per million population	Quantitative	Human Health	Surveillance & Laboratory
	Bloodstream infections: Absolute number of Streptococcus pneumoniae. BCIs with AST (Penicillins)	Quantitative	Human Health	Surveillance & Laboratory
	Bloodstream infections: % of Streptococcus pneumoniae aureus BCIs with AST (Penicillins)	Quantitative	Human Health	Surveillance & Laboratory
	Gastrointestinal infections: Total Salmonella spp. BCIs per million population	Quantitative	Human Health	Surveillance & Laboratory
	Gastrointestinal infections: Absolute number of Salmonella spp. BCIs per million population	Quantitative	Human Health	Surveillance & Laboratory
	Gastrointestinal infections: Total Salmonella spp. BCIs with AST (Fluoroquinolones) per million population	Quantitative	Human Health	Surveillance & Laboratory
	Gastrointestinal infections: Absolute number of Salmonella spp. BCIs with AST (Fluoroquinolones)	Quantitative	Human Health	Surveillance & Laboratory
	Gastrointestinal infections: % of Salmonella spp. BCIs with AST (Fluoroquinolones)	Quantitative	Human Health	Surveillance & Laboratory
	Gastrointestinal infections: Total Shigella spp. BCIs per million population	Quantitative	Human Health	Surveillance & Laboratory
	Gastrointestinal infections: Absolute number of Shigella spp. BCIs per million population	Quantitative	Human Health	Surveillance & Laboratory
	Gastrointestinal infections: Total Shigella spp. BCIs with AST (Third-generation cephalosporins) per million population	Quantitative	Human Health	Surveillance & Laboratory
	Gastrointestinal infections: % Shigella spp. BCIs with AST (third-generation cephalosporins)	Quantitative	Human Health	Surveillance & Laboratory
	Gastrointestinal infections: Absolute number of	Quantitative	Human Health	Surveillance & Laboratory

	Shigella pubis with AST (third-generation cephalosporins)			
	Gastrointestinal infections: Absolute number of Shigella spp. BCIs with AST (third-generation cephalosporins)	Quantitative	Human Health	Surveillance & Laboratory
	Gastrointestinal shigella spp. resistance rate (azithromycin)	Quantitative	Human Health	Surveillance & Laboratory
	Gonorrhoea infection: Total Neisseria gonorrhoeae BCIs per million population	Quantitative	Human Health	Surveillance & Laboratory
	Gonorrhoea infection: Absolute number of Neisseria gonorrhoeae BCIs per million population	Quantitative	Human Health	Surveillance & Laboratory
	Gonorrhoea infection: Total Neisseria gonorrhoeae BCIs with AST (Macrolides) per million population	Quantitative	Human Health	Surveillance & Laboratory
	Gonorrhoea infection: Absolute number of Neisseria gonorrhoeae BCIs with AST (Macrolides)	Quantitative	Human Health	Surveillance & Laboratory
	Gonorrhoea infection: % Neisseria gonorrhoeae BCIs with AST (Macrolides)	Quantitative	Human Health	Surveillance & Laboratory
	Gonorrhoea infection: Total Neisseria gonorrhoeae BCIs with AST (third-generation cephalosporins) per million population	Quantitative	Human Health	Surveillance & Laboratory
	Gonorrhoea infection: Absolute number of Neisseria gonorrhoeae BCIs with AST (third-generation cephalosporins)	Quantitative	Human Health	Surveillance & Laboratory
	Gonorrhoea infection: % Neisseria gonorrhoeae BCIs with AST (third-generation cephalosporins)	Quantitative	Human Health	Surveillance & Laboratory
	Urinary Tract Infections: Total Escherichia coli BCIs per million population	Quantitative	Human Health	Surveillance & Laboratory
	Urinary Tract Infections: Absolute number of Escherichia coli BCIs	Quantitative	Human Health	Surveillance & Laboratory
	Urinary Tract Infections: Total Escherichia coli BCIs with AST (Fluoroquinolones) per million population	Quantitative	Human Health	Surveillance & Laboratory
	Urinary Tract Infections: Absolute number of Escherichia coli BCIs with AST (Fluoroquinolones)	Quantitative	Human Health	Surveillance & Laboratory
	Urinary Tract Infections: % of Escherichia coli BCIs with AST (Fluoroquinolones)	Quantitative	Human Health	Surveillance & Laboratory

Urinary Tract Infections: Total Escherichia coli BCIs with AST (Third-generation cephalosporins) per million population	Quantitative	Human Health	Surveillance & Laboratory
Urinary Tract Infections: Absolute number of Escherichia coli BCIs with AST (third-generation cephalosporins)	Quantitative	Human Health	Surveillance & Laboratory
Urinary Tract Infections: % Escherichia coli BCIs with AST (third-generation cephalosporins)	Quantitative	Human Health	Surveillance & Laboratory
Urinary Tract Infections: Absolute number of Escherichia coli BCIs with AST (Sulphonamides and trimethoprim)	Quantitative	Human Health	Surveillance & Laboratory
Urinary Tract Infections: Total Klebsiella pneumoniae BCIs per million population	Quantitative	Human Health	Surveillance & Laboratory
Urinary Tract Infections: Absolute number of Klebsiella pneumoniae BCIs	Quantitative	Human Health	Surveillance & Laboratory
Urinary Tract Infections: Total Klebsiella pneumoniae BCIs with AST (Fluoroquinolones) per million population	Quantitative	Human Health	Surveillance & Laboratory
Urinary Tract Infections: Absolute number of Klebsiella pneumoniae BCIs with AST (Fluoroquinolones)	Quantitative	Human Health	Surveillance & Laboratory
Urinary Tract Infections: % of Klebsiella pneumoniae BCIs with AST (Fluoroquinolones)	Quantitative	Human Health	Surveillance & Laboratory
Urinary Tract Infections: Total Klebsiella pneumoniae BCIs with AST (Third-generation cephalosporins) per million population	Quantitative	Human Health	Surveillance & Laboratory
Urinary Tract Infections: Absolute number of Klebsiella pneumoniae BCIs with AST (third-generation cephalosporins)	Quantitative	Human Health	Surveillance & Laboratory
Urinary Tract Infections: % Klebsiella pneumoniae BCIs with AST (third-generation cephalosporins)	Quantitative	Human Health	Surveillance & Laboratory
Urinary Tract Infections: % of Escherichia coli BCIs with AST (Sulfonamides and trimethoprim)	Quantitative	Human Health	Surveillance & Laboratory
Urinary Tract Infections: % of Klebsiella pneumoniae BCIs with AST (Sulfonamides and trimethoprim)	Quantitative	Human Health	Surveillance & Laboratory
Urinary Tract Infections: Absolute number of Klebsiella pneumoniae BCIs with AST (Sulfonamides and trimethoprim)	Quantitative	Human Health	Surveillance & Laboratory

	Urinary Tract Infections: Total Escherichia coli BCIs with AST (Sulfonamides and trimethoprim) per million population	Quantitative	Human Health	Surveillance & Laboratory
	Urinary Tract Infections: Total Klebsiella pneumoniae BCIs with AST (Sulfonamides and trimethoprim) per million population	Quantitative	Human Health	Surveillance & Laboratory
	BCIs with AST per million population (bloodstream)	Quantitative	Human Health	Surveillance & Laboratory
	BCIs with AST per million population (gastrointestinal)	Quantitative	Human Health	Surveillance & Laboratory
	BCIs with AST per million population (gonorrhoea)	Quantitative	Human Health	Surveillance & Laboratory
	BCIs with AST per million population (urinary tract)	Quantitative	Human Health	Surveillance & Laboratory
	Percentage resistance to antibiotics by CTA (amikacin)	Quantitative	Human Health	Surveillance & Laboratory
	Percentage resistance to antibiotics by CTA (gentamicin)	Quantitative	Human Health	Surveillance & Laboratory
	Percentage resistance to antibiotics by CTA (doripenem)	Quantitative	Human Health	Surveillance & Laboratory
	Percentage resistance to antibiotics by CTA (imipenem)	Quantitative	Human Health	Surveillance & Laboratory
	Percentage resistance to antibiotics by CTA (meropenem)	Quantitative	Human Health	Surveillance & Laboratory
	Percentage resistance to antibiotics by CTA (colistin)	Quantitative	Human Health	Surveillance & Laboratory
	Percentage resistance to antibiotics by CTA (minocycline)	Quantitative	Human Health	Surveillance & Laboratory
	Percentage resistance to antibiotics by CTA (tigecycline)	Quantitative	Human Health	Surveillance & Laboratory
	Bloodstream Acinetobacter spp. resistance rate (amikacin)	Quantitative	Human Health	Surveillance & Laboratory
	Bloodstream Acinetobacter spp. resistance rate (gentamicin)	Quantitative	Human Health	Surveillance & Laboratory
	Bloodstream Acinetobacter spp. resistance rate (doripenem)	Quantitative	Human Health	Surveillance & Laboratory
	Bloodstream Acinetobacter spp. resistance rate (imipenem)	Quantitative	Human Health	Surveillance & Laboratory
	Bloodstream Acinetobacter spp. resistance rate	Quantitative	Human Health	Surveillance & Laboratory

(meropenem)			
Bloodstream Acinetobacter spp. resistance rate (colistin)	Quantitative	Human Health	Surveillance & Laboratory
Bloodstream Acinetobacter spp. resistance rate (minocycline)	Quantitative	Human Health	Surveillance & Laboratory
Bloodstream Acinetobacter spp. resistance rate (tigecycline)	Quantitative	Human Health	Surveillance & Laboratory
Bloodstream Escherichia coli resistance rate (doripenem)	Quantitative	Human Health	Surveillance & Laboratory
Bloodstream Escherichia coli resistance rate (ertapenem)	Quantitative	Human Health	Surveillance & Laboratory
Bloodstream Escherichia coli resistance rate (imipenem)	Quantitative	Human Health	Surveillance & Laboratory
Bloodstream Escherichia coli resistance rate (meropenem)	Quantitative	Human Health	Surveillance & Laboratory
Bloodstream Escherichia coli resistance rate (cefotaxime)	Quantitative	Human Health	Surveillance & Laboratory
Bloodstream Escherichia coli resistance rate (ceftazidime)	Quantitative	Human Health	Surveillance & Laboratory
Bloodstream Escherichia coli resistance rate (ceftriaxone)	Quantitative	Human Health	Surveillance & Laboratory
Bloodstream Escherichia coli resistance rate (cefepime)	Quantitative	Human Health	Surveillance & Laboratory
Bloodstream Escherichia coli resistance rate (ciprofloxacin)	Quantitative	Human Health	Surveillance & Laboratory
Bloodstream Escherichia coli resistance rate (levofloxacin)	Quantitative	Human Health	Surveillance & Laboratory
Bloodstream Escherichia coli resistance rate (ampicillin)	Quantitative	Human Health	Surveillance & Laboratory
Bloodstream Escherichia coli resistance rate (colistin)	Quantitative	Human Health	Surveillance & Laboratory
Bloodstream Escherichia coli resistance rate (co-trimoxazole)	Quantitative	Human Health	Surveillance & Laboratory
Bloodstream klebsiella pneumoniae resistance rate (doripenem)	Quantitative	Human Health	Surveillance & Laboratory
Bloodstream klebsiella pneumoniae resistance rate (imipenem)	Quantitative	Human Health	Surveillance & Laboratory
Bloodstream klebsiella pneumoniae resistance rate (meropenem)	Quantitative	Human Health	Surveillance & Laboratory

	Bloodstream klebsiella pneumoniae resistance rate (cefotaxime)	Quantitative	Human Health	Surveillance & Laboratory
	Bloodstream klebsiella pneumoniae resistance rate (ceftazidime)	Quantitative	Human Health	Surveillance & Laboratory
	Bloodstream klebisella pneumoniae resistance rate (cefepime)	Quantitative	Human Health	Surveillance & Laboratory
	Bloodstream klebisella pneumoniae resistance rate (ceftriaxone)	Quantitative	Human Health	Surveillance & Laboratory
	Bloodstream klebisella pneumoniae resistance rate (ciprofloxacin)	Quantitative	Human Health	Surveillance & Laboratory
	Bloodstream klebisella pneumoniae resistance rate (co-trimoxazole)	Quantitative	Human Health	Surveillance & Laboratory
	Bloodstream klebisella pneumoniae resistance rate (colistin)	Quantitative	Human Health	Surveillance & Laboratory
	Bloodstream klebisella pneumoniae resistance rate (levofloxacin)	Quantitative	Human Health	Surveillance & Laboratory
	Bloodstream klebsiella pneumoniae resistance rate (ertapenem)	Quantitative	Human Health	Surveillance & Laboratory
	Bloodstream salmonella spp. resistance rate (doripenem)	Quantitative	Human Health	Surveillance & Laboratory
	Bloodstream salmonella spp. resistance rate (ertapenem)	Quantitative	Human Health	Surveillance & Laboratory
	Bloodstream salmonella spp. resistance rate (imipenem)	Quantitative	Human Health	Surveillance & Laboratory
	Bloodstream salmonella spp. resistance rate (meropenem)	Quantitative	Human Health	Surveillance & Laboratory
	Bloodstream salmonella spp. resistance rate (cefotaxime)	Quantitative	Human Health	Surveillance & Laboratory
	Bloodstream salmonella spp. resistance rate (ceftazidime)	Quantitative	Human Health	Surveillance & Laboratory
	Bloodstream salmonella spp. resistance rate (ceftriaxone)	Quantitative	Human Health	Surveillance & Laboratory
	Bloodstream salmonella spp. resistance rate (ciprofloxacin)	Quantitative	Human Health	Surveillance & Laboratory
	Bloodstream salmonella spp. resistance rate (levofloxacin)	Quantitative	Human Health	Surveillance & Laboratory
	Bloodstream streptococcus pneumoniae resistance rate (cefotaxime)	Quantitative	Human Health	Surveillance & Laboratory
	Bloodstream streptococcus pneumoniae resistance	Quantitative	Human Health	Surveillance & Laboratory

	rate (ceftriaxone)			
	Bloodstream streptococcus pneumoniae resistance rate (oxacillin)	Quantitative	Human Health	Surveillance & Laboratory
	Bloodstream streptococcus pneumoniae resistance rate (penicillin G)	Quantitative	Human Health	Surveillance & Laboratory
	Bloodstream streptococcus pneumoniae resistance rate (Co-trimoxazole)	Quantitative	Human Health	Surveillance & Laboratory
	Gastrointestinal shigella spp. resistance rate (cefotaxime)	Quantitative	Human Health	Surveillance & Laboratory
	Gastrointestinal shigella spp. resistance rate (ceftazidime)	Quantitative	Human Health	Surveillance & Laboratory
	Gastrointestinal shigella spp. resistance rate (ceftriaxone)	Quantitative	Human Health	Surveillance & Laboratory
	Gastrointestinal shigella spp. resistance rate (ciprofloxacin)	Quantitative	Human Health	Surveillance & Laboratory
	Gastrointestinal shigella spp. resistance rate (levofloxacin)	Quantitative	Human Health	Surveillance & Laboratory
	Gastrointestinal shigella spp. resistance rate (azithromycin)	Quantitative	Human Health	Surveillance & Laboratory
	Gastrointestinal salmonella spp. resistance rate (doripenem)	Quantitative	Human Health	Surveillance & Laboratory
	Gastrointestinal salmonella spp. resistance rate (ertapenem)	Quantitative	Human Health	Surveillance & Laboratory
	Gastrointestinal salmonella spp. resistance rate (imipenem)	Quantitative	Human Health	Surveillance & Laboratory
	Gastrointestinal salmonella spp. resistance rate (meropenem)	Quantitative	Human Health	Surveillance & Laboratory
	Gastrointestinal salmonella spp. resistance rate (cefotaxime)	Quantitative	Human Health	Surveillance & Laboratory
	Gastrointestinal salmonella spp. resistance rate (ceftazidime)	Quantitative	Human Health	Surveillance & Laboratory
	Gastrointestinal salmonella spp. resistance rate (ceftriaxone)	Quantitative	Human Health	Surveillance & Laboratory
	Gastrointestinal salmonella spp. resistance rate (ciprofloxacin)	Quantitative	Human Health	Surveillance & Laboratory
	Gastrointestinal salmonella spp. resistance rate (levofloxacin)	Quantitative	Human Health	Surveillance & Laboratory
	Neisseria gonorrhoeae resistance rate (gentamicin)	Quantitative	Human Health	Surveillance & Laboratory

Neisseria gonorrhoeae resistance rate (spectinomycin)	Quantitative	Human Health	Surveillance & Laboratory
Neisseria gonorrhoeae resistance rate (cefixime)	Quantitative	Human Health	Surveillance & Laboratory
Neisseria gonorrhoeae resistance rate (ceftriaxone)	Quantitative	Human Health	Surveillance & Laboratory
Neisseria gonorrhoeae resistance rate (ciprofloxacin)	Quantitative	Human Health	Surveillance & Laboratory
Neisseria gonorrhoeae resistance rate (azithromycin)	Quantitative	Human Health	Surveillance & Laboratory
Urinary tract escherichia coli resistance rate (doripenem)	Quantitative	Human Health	Surveillance & Laboratory
Urinary tract escherichia coli resistance rate (ertapenem)	Quantitative	Human Health	Surveillance & Laboratory
Urinary tract escherichia coli resistance rate (imipenem)	Quantitative	Human Health	Surveillance & Laboratory
Urinary tract escherichia coli resistance rate (meropenem)	Quantitative	Human Health	Surveillance & Laboratory
Urinary tract escherichia coli resistance rate (cefotaxime)	Quantitative	Human Health	Surveillance & Laboratory
Urinary tract escherichia coli resistance rate (ceftazidime)	Quantitative	Human Health	Surveillance & Laboratory
Urinary tract escherichia coli resistance rate (ceftriaxone)	Quantitative	Human Health	Surveillance & Laboratory
Urinary tract escherichia coli resistance rate (cefepime)	Quantitative	Human Health	Surveillance & Laboratory
Urinary tract escherichia coli resistance rate (ciprofloxacin)	Quantitative	Human Health	Surveillance & Laboratory
Urinary tract escherichia coli resistance rate (levofloxacin)	Quantitative	Human Health	Surveillance & Laboratory
Urinary tract escherichia coli resistance rate (ampicillin)	Quantitative	Human Health	Surveillance & Laboratory
Urinary tract escherichia coli resistance rate (colistin)	Quantitative	Human Health	Surveillance & Laboratory
Urinary tract escherichia coli resistance rate (co-trimoxazole)	Quantitative	Human Health	Surveillance & Laboratory
Urinary tract klebsiella pneumoniae resistance rate (doripenem)	Quantitative	Human Health	Surveillance & Laboratory
Urinary tract klebsiella pneumoniae resistance rate (ertapenem)	Quantitative	Human Health	Surveillance & Laboratory

		Urinary tract klebsiella pneumoniae resistance rate (imipenem)	Quantitative	Human Health	Surveillance & Laboratory
		Urinary tract klebsiella pneumoniae resistance rate (meropenem)	Quantitative	Human Health	Surveillance & Laboratory
		Urinary tract klebsiella pneumoniae resistance rate (cefotaxime)	Quantitative	Human Health	Surveillance & Laboratory
		Urinary tract klebsiella pneumoniae resistance rate (ceftazidime)	Quantitative	Human Health	Surveillance & Laboratory
		Urinary tract klebsiella pneumoniae resistance rate (ceftriaxone)	Quantitative	Human Health	Surveillance & Laboratory
		Urinary tract klebsiella pneumoniae resistance rate (cefepime)	Quantitative	Human Health	Surveillance & Laboratory
		Urinary tract klebsiella pneumoniae resistance rate (ciprofloxacin)	Quantitative	Human Health	Surveillance & Laboratory
		Urinary tract klebsiella pneumoniae resistance rate (levofloxacin)	Quantitative	Human Health	Surveillance & Laboratory
		Urinary tract klebsiella pneumoniae resistance rate (colistin)	Quantitative	Human Health	Surveillance & Laboratory
		Urinary tract klebsiella pneumoniae resistance rate (co-trimoxazole)	Quantitative	Human Health	Coordination
5	Monitoring and evaluation for effective management of zoonotic diseases	Direct change in State Party Self-Assessment Annual Reporting indicator levels	Quantitative	Multisectoral	Reporting
		Number/list of policies/legal instruments enabling implementation of multisectoral, One Health coordinated activities	Quantitative	Multisectoral	Coordination
		Proportion of relevant sectors included in coordinated activities or in the MCM	Quantitative	Multisectoral	Sustainability
		Proportion of coordinated activities that are sufficiently resourced	Quantitative	Multisectoral	Coordination
		Number of coordinated activities organized/conducted	Quantitative	Multisectoral	Workforce
		Number of strategies/plans jointly developed/revised/adopted by relevant sectors	Quantitative	Multisectoral	Coordination
		Number of joint training sessions in relevant technical areas	Quantitative	Multisectoral	Coordination
		Number/list of One Health coordination structures and/or governance processes established	Quantitative	Multisectoral	Feedback Mechanisms
		Number of assessments conducted to inform revision or development of coordinated activities	Quantitative	Multisectoral	Coordination

	Number/list of coordinated operational documents developed/implemented	Quantitative	Multisectoral	Strategic Vision
	Proportion of priority zoonotic diseases that have a joint plan	Quantitative	Multisectoral	Workforce
	Demonstrated evidence for the workforce that is competent, maintained and mobilized by relevant sectors for an effective coordinated zoonotic disease management	Qualitative	Multisectoral	Workforce
	Workforce functions and occupations are identified according to the needs	Ordinal	Multisectoral	Workforce
	Number of coordinated activities implemented to train, maintain and mobilize a workforce	Quantitative	Multisectoral	Coordination
	Demonstrated evidence for coordination functions by MCM	Qualitative	Multisectoral	Coordination
	Demonstrated evidence for joint management and operational infrastructure for effective coordinated planning and preparedness to zoonotic disease events	Qualitative	Multisectoral	Feedback Mechanisms
	Proportion of zoonotic disease events for which timeliness metrics were used to drive performance improvement process throughout the investigation and response	Quantitative	Animal Health	Workforce
	Joint (rapid) response team roster is shared and mobilized across sectors	Qualitative	Multisectoral	Feedback Mechanisms
	Proportion of zoonotic disease events that are evaluated using a decision tool/process	Quantitative	Animal Health	Feedback Mechanisms
	Proportion of zoonotic disease events that have joint/coordinated investigation and/or response	Quantitative	Multisectoral	Feedback Mechanisms
	Number of activities implemented for zoonotic diseases based on joint risk assessment recommendations	Quantitative	Multisectoral	Workforce
	Number of professionals trained to conduct joint risk assessment	Quantitative	Multisectoral	Surveillance & Laboratory
	A coordinated surveillance and information sharing systems for zoonotic diseases is established at national level/piloted	Qualitative	Multisectoral	Surveillance & Laboratory
	Proportion of priority zoonotic diseases for which quality surveillance data are shared with relevant sectors	Quantitative	Multisectoral	Community Awareness & Enabling Behaviours

		Number/list of identified and engaged stakeholders from relevant sectors for risk reduction, risk communication and community engagement	Quantitative	Multisectoral	Community Awareness & Enabling Behaviours
		Number of risk reduction/risk communication and community engagement activities jointly developed/implemented	Quantitative	Multisectoral	Community Awareness & Enabling Behaviours
		Proportion of implemented activities that are adopted by communities	Quantitative	Multisectoral	Prevention & Control
		Direct change in units measured resulting from zoonotic disease prevention and control	Quantitative	Multisectoral	Reporting
6	UHC Watch	Out-of-pocket payments as a share of household consumption by consumption quintile	Quantitative	Human Health	Access to Medicines and Health Services
		Breakdown of out-of-pocket payments by type of care	Quantitative	Human Health	Access to Medicines and Health Services
		Breakdown of out-of-pocket payments by type of care and consumption quintile	Quantitative	Human Health	Access to Medicines and Health Services
		Households with out-of-pocket payments by consumption quintile	Quantitative	Human Health	Access to Medicines and Health Services
		Annual out-of-pocket payments by consumption quintile	Quantitative	Human Health	Access to Medicines and Health Services
		Annual out-of-pocket payments per person by type of care	Quantitative	Human Health	Access to Medicines and Health Services
		Out-of-pocket payments as a share of household consumptions	Quantitative	Human Health	Access to Medicines and Health Services
		Households with impoverishing health spending	Quantitative	Human Health	Access to Medicines and Health Services
		Households with catastrophic health spending	Quantitative	Human Health	Access to Medicines and Health Services
		Households at risk of impoverishment after out-of-pocket payments	Quantitative	Human Health	Access to Medicines and Health Services
		Households with catastrophic health spending by consumption quintile	Quantitative	Human Health	Access to Medicines and Health Services
		Breakdown of catastrophic health spending by type of care	Quantitative	Human Health	Access to Medicines and Health Services
		Breakdown of catastrophic health spending by type of care and consumption quintile	Quantitative	Human Health	Access to Medicines and Health Services
		Breakdown of households with catastrophic health spending by risk of impoverishment	Quantitative	Human Health	Access to Medicines and Health Services
		The cost of meeting basic needs, capacity to pay	Quantitative	Human Health	Access to Medicines and Health

	for health care and households living below the basic needs line			Services
	Breakdown of catastrophic health spending by type of care in the poorest quintile	Quantitative	Human Health	Access to Medicines and Health Services
	Breakdown of households with health spending by risk of impoverishment	Quantitative	Human Health	Access to Medicines and Health Services
	Unmet need for health care due to cost, distance and waiting time (poorest quintile, total, richest quintile)	Quantitative	Human Health	Access to Medicines and Health Services
	Unmet need for dental care due to cost, distance and waiting time (poorest quintile, total, richest quintile)	Quantitative	Human Health	Access to Medicines and Health Services
	Unmet need for health care due to cost, distance and waiting time	Quantitative	Human Health	Access to Medicines and Health Services
	Unmet need for dental care due to cost, distance and waiting time	Quantitative	Human Health	Access to Medicines and Health Services
	Unmet need for prescribed medicines due to cost	Quantitative	Human Health	Access to Medicines and Health Services
	Unmet need for prescribed medicines due to cost (poorest quintile, total, richest quintile)	Quantitative	Human Health	Access to Medicines and Health Services
	Current spending on health per person by financing scheme	Quantitative	Human Health	Access to Medicines and Health Services
	Breakdown of current spending on health by financing scheme and type of care	Quantitative	Human Health	Access to Medicines and Health Services
	Out-of-pocket payments as a share of current spending on health	Quantitative	Human Health	Access to Medicines and Health Services
	Voluntary health insurance spending as a share of current spending on health	Quantitative	Human Health	Access to Medicines and Health Services
	Public spending on health as a share of current spending on health	Quantitative	Human Health	Access to Medicines and Health Services
	Public spending on health as a share of government spending	Quantitative	Human Health	Access to Medicines and Health Services
	Public spending on health as a share of GDP	Quantitative	Human Health	Access to Medicines and Health Services
	Households with catastrophic health spending and out-of-pocket payments as a share of current spending on health	Quantitative	Human Health	Access to Medicines and Health Services
	Public spending on health as a share of GDP and out-of-pocket payments as a share of current	Quantitative	Human Health	Access to Medicines and Health Services

		spending on health			
		Public spending on health as a share of GDP and GDP per person	Quantitative	Human Health	Participation
7	Global Coalition on Aging AMR Preparedness Index Progress Report	Existence and creation of NAPs or new funding for NAPs	Ordinal	Multisectoral	Research, Innovation, & Digital Technology
		Government funding for research projects related to AMR	Ordinal	Multisectoral	Coordination
		Existence of collaborations with international research initiatives	Ordinal	Multisectoral	Coordination
		Existence of collaborations among Governments and NGOs or patient groups	Ordinal	Multisectoral	Research, Innovation, & Digital Technology
		Existence and creation of pull incentive programs or pilots	Ordinal	Multisectoral	Research, Innovation, & Digital Technology
		Existence and improvement of funding for basic and therapeutic research projects via push incentives	Ordinal	Multisectoral	Regulations & Legislation
		Existence and creation of policies that consider the value of antibiotics in pricing	Ordinal	Multisectoral	Regulations & Legislation
		Existence and creation of policies to fast-track drug approvals, including for antibiotics	Ordinal	Multisectoral	Research, Innovation, & Digital Technology
		Number of antibiotics commercialized in each country out of all antibiotic new molecular entities developed since 2010	Ordinal	Multisectoral	Access to Medicines and Health Services
		Affordability of healthcare and antibiotics in each country in terms of the proportion of populations spending more than 10% of income on healthcare, and the proportion with catastrophic health expenditure	Ordinal	Human Health	Community Awareness & Enabling Behaviours
		Existence and creation of incentives to promote and monitor public awareness	Ordinal	Multisectoral	Regulations & Legislation
		Existence and creation of policies to promote antimicrobial stewardship teams or practices	Ordinal	Multisectoral	Prevention & Control
		Country-reported adherence to WHO IPC guidelines	Ordinal	Human Health	Reporting / Participation
		Participation in GLASS	Ordinal	Human Health	Surveillance & Laboratory
		Existence or establishment of national reference laboratories involved in AMR surveillance	Ordinal	Multisectoral	Workforce
		Inclusion of AMR within medical personnel	Ordinal	Human Health	Strategic Vision

		training curriculum			
		Existence and creation of sections or pillars dedicated to environmental issues in NAPs	Ordinal	Environment	Sustainability
		Existence of funding for One Health initiatives in government budgets	Ordinal	Multisectoral	Surveillance & Laboratory
		Existence of initiatives to monitor antimicrobial resistant genes in water	Ordinal	Environment	Surveillance & Laboratory
8	Surveillance of health care-associated infections at national and facility levels: practical handbook (WHO)	National strategic plan for surveillance of HAI and related AMR (with a focus on priority infections based on the local context) developed by a multidisciplinary technical group (by 2026) within the context of a broader surveillance system	Y/N	Human Health	Surveillance & Laboratory
		National/subnational surveillance system for HAI and related AMR (including for early warning the ability to detect epidemic- and pandemic-prone pathogens causing HAI) established and supported (including financially) by governmental and national/subnational authorities (by 2028)	Y/N	Human Health	Surveillance & Laboratory
		Proportion of tertiary/secondary health care facilities participating in the national/subnational or international network for surveillance of HAI and related AMR, if existing	Quantitative	Human Health	Surveillance & Laboratory
		Proportion of tertiary/secondary health care facilities having a surveillance system for HAI and related AMR including for early warning the ability to detect epidemic- and pandemic-prone pathogens	Quantitative	Human Health	Strategic Vision
9	Commitment to Development index (Health)	Prevention of disease - AMR - antibiotic consumption in humans (DDD per 1000 population)	Quantitative	Human Health	Stewardship
		Prevention of disease - AMR - Antibiotic consumption in Livestock animals (mg/PCU)	Quantitative	Animal Health	Prevention & Control
		Prevention of disease - vaccination coverage - measles vaccine (MCV2) coverage	Quantitative	Human Health	Prevention & Control
		Prevention of disease - vaccination coverage - DPT3 vaccine coverage	Quantitative	Human Health	Prevention & Control
		Prevention of disease - pandemic preparedness - Completion and publication of Joint External Evaluation mission report	Y/N	Multisectoral	Reporting

		Prevention of disease - pandemic preparedness - Completion of a Performance of Veterinary Services (PVS) evaluation with WOAH in the past 10 years	Y/N	Animal Health	Reporting
		Prevention of disease - pandemic preparedness - Completion of a biological focused IHR exercise with the WHO in the previous 2 calendar years	Y/N	Multisectoral	Prevention & Control
		Prevention of disease - pandemic preparedness - Existence of a public pandemic influenza preparedness plan which has been updated at least since 2011	Y/N	Multisectoral	Coordination
10	WHO Joint External Evaluation - AMR	P4.1 Multisectoral coordination on AMR (graded 1-5)	Ordinal	Multisectoral	Surveillance & Laboratory
		P4.2 Surveillance of AMR (graded 1-5)	Ordinal	Multisectoral	Stewardship
		P4.3 Prevention of multidrug resistant organisms (graded 1-5)	Ordinal	Multisectoral	Prevention & Control/Effectiveness
		P4.4 Optimal use of antimicrobial medicines in human health (graded 1-5)	Ordinal	Human Health	Stewardship
		P4.5 Optimal use of antimicrobial medicines in animal health and agriculture (graded 1-5)	Ordinal	Animal Health	Stewardship
11	WHO benchmarks for strengthening health emergency capacities	Benchmark 4.2: A surveillance system for AMR is in place	Ordinal (scale 1-5)	Multisectoral	Prevention & Control
		Benchmark 4.3 Effective mechanisms are in place to prevent multidrug resistant organisms (MDRO)	Ordinal (scale 1-5)	Multisectoral	Stewardship
		Benchmark 4.4 Optimize use of antimicrobial medicines in human health	Ordinal (scale 1-5)	Human Health	Stewardship
		Benchmark 4.5 Optimize use of antimicrobial medicines in animal health and agriculture	Ordinal (scale 1-5)	Animal Health	Surveillance & Laboratory
		Benchmark 5.1 A multisectoral surveillance system is in place for priority zoonotic diseases/pathogens	Ordinal (scale 1-5)	Animal Health	Surveillance & Laboratory
		Benchmark 5.2 A functional mechanism to respond to priority zoonotic diseases is in place	Ordinal (scale 1-5)	Animal Health	Prevention & Control
		Benchmark 5.3 Safe practices in animal breeding and animal product systems limit the risk of zoonotic diseases	Ordinal (scale 1-5)	Animal Health	Prevention & Control
		Benchmark 6.1 Surveillance systems are in place for the detection and monitoring of foodborne diseases and food contamination	Ordinal (scale 1-5)	Environment	Prevention & Control

	Benchmark 6.2 A functional mechanism is in place for the response and management of food safety emergencies	Ordinal (scale 1-5)	Environment	Prevention & Control
	Benchmark 8.1 Whole-of-government biosafety and biosecurity system is in place for relevant sectors including human, animal (domestic animals and wildlife) and agricultural facilities	Ordinal (scale 1-5)	Multisectoral	Workforce
	Benchmark 8.2 Biosafety and biosecurity training and practices in relevant sectors including human health, animal health (domestic animals and wildlife) and agriculture are in place	Ordinal (scale 1-5)	Multisectoral	Surveillance & Laboratory
	Benchmark 9.1 Specimen referral and transport system is in place for relevant sectors	Ordinal (scale 1-5)	Multisectoral	Surveillance & Laboratory
	Benchmark 9.2 Laboratory quality system is in place	Ordinal (scale 1-5)	Multisectoral	Surveillance & Laboratory
	Benchmark 9.3 Laboratory testing for detection of priority diseases is in place	Ordinal (scale 1-5)	Multisectoral	Surveillance & Laboratory
	Benchmark 9.4 An effective national diagnostic network is in place	Ordinal (scale 1-5)	Human Health	Surveillance & Laboratory
	Benchmark 10.1 Early warning surveillance systems are well established and functional	Ordinal (scale 1-5)	Multisectoral	Surveillance & Laboratory
	Benchmark 10.2 Well functioning event verification and investigation systems are in place	Ordinal (scale 1-5)	Multisectoral	Feedback Mechanisms
	Benchmark 10.3 Surveillance data and information are systematically analysed and shared to inform decision making for action	Ordinal (scale 1-5)	Multisectoral	Workforce
	Benchmark 11.1 An up-to-date multisectoral workforce strategy is in place	Ordinal (scale 1-5)	Multisectoral	Workforce
	Benchmark 11.3 Fit for purpose, competency-based education programmes are available for multisectoral workforce	Ordinal (scale 1-5)	Multisectoral	Workforce
	Benchmark 11.4 Multisectoral workforce surge strategy for health emergencies is well established and functional	Ordinal (scale 1-5)	Multisectoral	Coordination / Prevention & Control
	Benchmark 12A.5 A system is in place for emergency logistics and supply chain management during a health emergency	Ordinal (scale 1-5)	Multisectoral	Research, Innovation, & Digital Technology
	Benchmark 12A.6 Research, development and innovation (RD&I) capacity for emergency	Ordinal (scale 1-5)	Multisectoral	Prevention & Control

		management is in place			
		Benchmark 15.1 National and health facility level infection prevention and control programmes are in place	Ordinal (scale 1-5)	Human Health	Surveillance & Laboratory
		Benchmark 15.2 A functioning health care acquired infection surveillance system is in place for public health decision-making	Ordinal (scale 1-5)	Human Health	Surveillance & Laboratory
		Benchmark 15.3 Provide a safe environment in all healthcare facilities	Ordinal (scale 1-5)	Human Health	Prevention & Control
		Benchmark H1.2 Genomic surveillance systems are in place and functional	Ordinal (scale 1-5)	Multisectoral	Surveillance & Laboratory
		Benchmark H1.3 Integrated, interoperable and standardized data systems and data sharing platforms are established and functional	Ordinal (scale 1-5)	Multisectoral	Surveillance & Laboratory / Coordination
		Benchmark H1.4 Integrated networks are created and functional to support surveillance information sharing and collaboration	Ordinal (scale 1-5)	Multisectoral	Prevention & Control
		Benchmark H2.1: Integrated vector control management systems are in place	Ordinal (scale 1-5)	Multisectoral	Prevention & Control / Social Determinants
		Benchmark H2.2: Community driven water, sanitation and hygiene (WASH) interventions are in place and effective	Ordinal (scale 1-5)	Multisectoral	Research, Innovation, & Digital Technology
		Benchmark H4.1: Standardized platforms for conducting equitable and scalable clinical trials are created and functional	Ordinal (scale 1-5)	Multisectoral	Research, Innovation, & Digital Technology
		Benchmark H4.2: Regulatory and legal frameworks are developed and functional for timely trials, product review and approval	Ordinal (scale 1-5)	Multisectoral	Regulations & Legislation
		Benchmark H4.3 Adaptable manufacturing platforms are established and functional, and supported by pre-negotiated agreements	Ordinal (scale 1-5)	Multisectoral	Research, Innovation, & Digital Technology
		Benchmark H4.4: Manufacturing capabilities are enhanced through ever-ready capabilities for rapid mobilization of medical countermeasure production during health emergencies	Ordinal (scale 1-5)	Multisectoral	Reporting
12	8th Annual Report on Antimicrobial Agents Intended for Use in Animals (ANIMUSE)	Reporting of antimicrobial quantities to ANIMUSE	Y/N	Animal Health	Stewardship
		Use of antimicrobial growth promoters (Use, no use, unknown use)	Qualitative	Animal Health	Regulations & Legislation

Existence of country-level legislation/regulations on the use of antimicrobial growth promoters in animals	Y/N	Animal Health	Regulations & Legislation
Type of growth promotion legislation (some antimicrobial agents banned for use as growth promoters; some antimicrobial agents banned for use as growth promoters + One or more antimicrobial growth promoters are authorized for use; one or more antimicrobial growth promoters are authorized for use)	Qualitative	Animal Health	Stewardship
Differentiation by animal group (overall)	Y/N	Animal Health	Reporting
Differentiation by animal group (terrestrial vs aquatic food producing animals)	Y/N	Animal Health	Reporting
Differentiation by animal group (food-producing vs non-food producing animals)	Y/N	Animal Health	Reporting
Included cattle in quantitative data reporting (terrestrial food-producing animals)	Y/N	Animal Health	Reporting
Included poultry in quantitative data reporting (terrestrial food-producing animals)	Y/N	Animal Health	Reporting
Included small ruminants in quantitative data reporting (terrestrial food-producing animals)	Y/N	Animal Health	Reporting
Included pigs in quantitative data reporting (terrestrial food-producing animals)	Y/N	Animal Health	Reporting
Included equidae in quantitative data reporting (terrestrial food-producing animals)	Y/N	Animal Health	Reporting
Included rabbits in quantitative data reporting (terrestrial food-producing animals)	Y/N	Animal Health	Reporting
Included camelidae in quantitative data reporting (terrestrial food-producing animals)	Y/N	Animal Health	Reporting
Included bees - honey in quantitative data reporting (terrestrial food-producing animals)	Y/N	Animal Health	Reporting
Included cervidae (farmed) in quantitative data reporting (terrestrial food-producing animals)	Y/N	Animal Health	Reporting
Included reptiles in quantitative data reporting (terrestrial food-producing animals)	Y/N	Animal Health	Reporting
Reported use of sulfathiazole as a growth promoter	Y/N	Animal Health	Stewardship
Reported use of sulfaquinoxaline as a growth promoter	Y/N	Animal Health	Stewardship

	Reported use of sulfadimethoxine as a growth promoter	Y/N	Animal Health	Stewardship
	Reported use of sulfadimethoxazole as a growth promoter	Y/N	Animal Health	Stewardship
	Reported use of sulfadiazine as a growth promoter	Y/N	Animal Health	Stewardship
	Reported use of streptomycin as a growth promoter	Y/N	Animal Health	Stewardship
	Reported use of fosfomycin as a growth promoter	Y/N	Animal Health	Stewardship
	Reported use of erythromycin as a growth promoter	Y/N	Animal Health	Stewardship
	Reported use of clindamycin as a growth promoter	Y/N	Animal Health	Stewardship
	Reported use of carbadox as a growth promoter	Y/N	Animal Health	Stewardship
	Reported use of bicozamycin as a growth promoter	Y/N	Animal Health	Stewardship
	Reported use of apramycin as a growth promoter	Y/N	Animal Health	Stewardship
	Reported use of sulfadimidine as a growth promoter	Y/N	Animal Health	Stewardship
	Reported use of olaquindox as a growth promoter	Y/N	Animal Health	Stewardship
	Reported use of nociceptive as a growth promoter	Y/N	Animal Health	Stewardship/Reporting
	Reported use of kitasamycin as a growth promoter	Y/N	Animal Health	Stewardship
	Reported use of florfenicol as a growth promoter	Y/N	Animal Health	Stewardship
	Reported use of amoxicillin as a growth promoter	Y/N	Animal Health	Stewardship
	Reported use of tilmicosin as a growth promoter	Y/N	Animal Health	Stewardship
	Reported use of tetracycline as a growth promoter	Y/N	Animal Health	Stewardship
	Reported use of neomycin as a growth promoter	Y/N	Animal Health	Stewardship
	Reported use of enrofloxacin as a growth promoter	Y/N	Animal Health	Stewardship
	Reported use of colistin sulphate as a growth promoter	Y/N	Animal Health	Stewardship
	Reported use of tiamulin as a growth promoter	Y/N	Animal Health	Stewardship
	Reported use of chlortetracycline as a growth promoter	Y/N	Animal Health	Stewardship
	Reported use of halquinol as a growth promoter	Y/N	Animal Health	Stewardship
	Reported use of oxytetracycline as a growth promoter	Y/N	Animal Health	Stewardship

Reported use of virginiamycin as a growth promoter	Y/N	Animal Health	Stewardship
Reported use of lincomycin as a growth promoter	Y/N	Animal Health	Stewardship
Reported use of avilamycin as a growth promoter	Y/N	Animal Health	Stewardship
Reported use of enramycin as a growth promoter	Y/N	Animal Health	Stewardship
Reported use of bacitracin as a growth promoter	Y/N	Animal Health	Stewardship
Reported use of flavophospholipol as a growth promoter	Y/N	Animal Health	Stewardship
Reported use of tylosin as a growth promoter	Y/N	Animal Health	Reporting
Reporting of sales data for antimicrobial quantities intended for use in animals	Y/N	Animal Health	Reporting
Reporting of import data for antimicrobial quantities intended for use in animals	Y/N	Animal Health	Reporting
Reporting of prescription data for antimicrobial quantities intended for use in animals	Y/N	Animal Health	Reporting
Reporting of purchase data for antimicrobial quantities intended for use in animals	Y/N	Animal Health	Reporting
Reporting of use - farm records data for antimicrobial quantities intended for use in animals	Y/N	Animal Health	Reporting
Reporting of manufacturing data for antimicrobial quantities intended for use in animals	Y/N	Animal Health	Stewardship
Reported quantity of antimicrobial agents intended for use in animals (in tonnes)	Quantitative	Animal Health	Stewardship
Reported quantity of antimicrobial agents intended for use in animals (in tonnes), adjusted by estimated coverage	Quantitative	Animal Health	Stewardship
Proportion of antimicrobial classes reported for use in animals (tetracyclines)	Quantitative	Animal Health	Stewardship
Proportion of antimicrobial classes reported for use in animals (polypeptides)	Quantitative	Animal Health	Stewardship
Proportion of antimicrobial classes reported for use in animals (penicillins)	Quantitative	Animal Health	Stewardship/Reporting
Proportion of antimicrobial classes reported for use in animals (macrolides)	Quantitative	Animal Health	Stewardship
Proportion of antimicrobial classes reported for use in animals (sulfonamides - including trimethoprim)	Quantitative	Animal Health	Stewardship/Reporting
Proportion of antimicrobial classes reported for use	Quantitative	Animal Health	Stewardship

	in animals (amphenicols)			
	Proportion of antimicrobial classes reported for use in animals (aminoglycosides)	Quantitative	Animal Health	Stewardship
	Proportion of antimicrobial classes reported for use in animals (fluoroquinolones)	Quantitative	Animal Health	Stewardship
	Proportion of antimicrobial classes reported for use in animals (pleuromutilins)	Quantitative	Animal Health	Stewardship
	Proportion of antimicrobial classes reported for use in animals (lincosamides)	Quantitative	Animal Health	Stewardship
	Proportion of antimicrobial classes reported for use in animals (aggregated class data)	Quantitative	Animal Health	Stewardship
	Proportion of antimicrobial classes reported for use in animals (orthosomycins)	Quantitative	Animal Health	Stewardship
	Proportion of antimicrobial classes reported for use in animals (cephalosporins - all generations)	Quantitative	Animal Health	Stewardship
	Proportion of antimicrobial classes reported for use in animals (3-4 gen cephalosporins)	Quantitative	Animal Health	Stewardship
	Proportion of antimicrobial classes reported for use in animals (1-2 gen cephalosporins)	Quantitative	Animal Health	Stewardship
	Proportion of antimicrobial classes reported for use in animals (others)	Quantitative	Animal Health	Stewardship
	Proportion of antimicrobial classes reported for use in animals (glycophospholipids)	Quantitative	Animal Health	Stewardship
	Proportion of antimicrobial classes reported for use in animals (streptogramins)	Quantitative	Animal Health	Stewardship
	Proportion of antimicrobial classes reported for use in animals (quinoxalines)	Quantitative	Animal Health	Stewardship
	Proportion of antimicrobial classes reported for use in animals (other quinolones)	Quantitative	Animal Health	Stewardship
	Proportion of antimicrobial classes reported for use in animals (nitrofurans)	Quantitative	Animal Health	Stewardship
	Proportion of antimicrobial classes reported for use in animals (arsenicals)	Quantitative	Animal Health	Stewardship
	Tonnes of antimicrobials allocated to tetracyclines	Quantitative	Animal Health	Stewardship
	Tonnes of antimicrobial allocated to polypeptides	Quantitative	Animal Health	Stewardship
	Use of tetracyclines compared to the total amount reported (% - mean)	Quantitative	Animal Health	Stewardship
	Use of polypeptides compared to the total amount	Quantitative	Animal Health	Stewardship

	reported (% - mean)			
	Proportion of tetracyclines used in food-producing animals	Quantitative	Animal Health	Stewardship
	Proportion of penicillins used in terrestrial food-producing animals	Quantitative	Animal Health	Stewardship
	Proportion of macrolides used in terrestrial food-producing animals	Quantitative	Animal Health	Stewardship
	Proportion of amphenicols used in terrestrial food-producing animals	Quantitative	Animal Health	Stewardship
	Proportion of Aminoglycosides used in terrestrial food-producing animals	Quantitative	Animal Health	Stewardship
	Proportion of Pleuromutilins used in terrestrial food-producing animals	Quantitative	Animal Health	Stewardship
	Proportion of Fluoroquinolones used in terrestrial food-producing animals	Quantitative	Animal Health	Stewardship
	Proportion of Lincosamides used in terrestrial food-producing animals	Quantitative	Animal Health	Stewardship
	Proportion of Polypeptides used in terrestrial food-producing animals	Quantitative	Animal Health	Stewardship
	Proportion of Cephalosporins (all generations) used in terrestrial food-producing animals	Quantitative	Animal Health	Stewardship
	Proportion of Sulfonamides (including trimethoprim) used in terrestrial food-producing animals	Quantitative	Animal Health	Stewardship
	Proportion of 3-4 gen cephalosporins used in terrestrial food-producing animals	Quantitative	Animal Health	Stewardship
	Proportion of 1-2 gen cephalosporins used in terrestrial food-producing animals	Quantitative	Animal Health	Stewardship
	Proportion of other antimicrobial classes used in terrestrial food-producing animals	Quantitative	Animal Health	Stewardship
	Proportion of Orthosomycins used in terrestrial food-producing animals	Quantitative	Animal Health	Stewardship
	Proportion of Quinoxalines used in terrestrial food-producing animals	Quantitative	Animal Health	Stewardship
	Proportion of Glycophospholipids used in terrestrial food-producing animals	Quantitative	Animal Health	Stewardship
	Proportion of Aggregated class data used in terrestrial food-producing animals	Quantitative	Animal Health	Stewardship
	Proportion of Streptogramins used in terrestrial	Quantitative	Animal Health	Stewardship

food-producing animals			
Proportion of Other quinolones used in terrestrial food-producing animals	Quantitative	Animal Health	Stewardship
Proportion of Arsenicals used in terrestrial food-producing animals	Quantitative	Animal Health	Stewardship
Proportion of Nitrofurans used in terrestrial food-producing animals	Quantitative	Animal Health	Reporting
Included fish in quantitative data reporting (aquatic food-producing animals)	Quantitative	Animal Health	Reporting
Included crustaceans in quantitative data reporting (aquatic food-producing animals)	Quantitative	Animal Health	Reporting
Included amphibians in quantitative data reporting (aquatic food-producing animals)	Quantitative	Animal Health	Reporting
Included molluscs in quantitative data reporting (aquatic food-producing animals)	Quantitative	Animal Health	Stewardship
Proportion of amphenicols used by aquatic food-producing animals	Quantitative	Animal Health	Stewardship
Proportion of fluoroquinolones used by aquatic food-producing animals	Quantitative	Animal Health	Stewardship
Proportion of tetracyclines used by aquatic food-producing animals	Quantitative	Animal Health	Stewardship
Proportion of aminoglycosides used by aquatic food-producing animals	Quantitative	Animal Health	Stewardship
Proportion of Pleuromutilins used by aquatic food-producing animals	Quantitative	Animal Health	Stewardship
Proportion of Penicillins used by aquatic food-producing animals	Quantitative	Animal Health	Stewardship
Proportion of Macrolides used by aquatic food-producing animals	Quantitative	Animal Health	Stewardship
Proportion of sulfonamides (including trimethoprim) used by aquatic food-producing animals	Quantitative	Animal Health	Stewardship
Proportion of Other quinolones used by aquatic food-producing animals	Quantitative	Animal Health	Reporting
Included canines in quantitative data reporting (non-food-producing animals)	Y/N	Animal Health	Reporting
Included felines in quantitative data reporting (non-food-producing animals)	Y/N	Animal Health	Reporting
Included ornamental fish in quantitative data	Y/N	Animal Health	Stewardship

	reporting (non-food-producing animals)			
	Proportion of penicillins used in companion animals	Quantitative	Animal Health	Stewardship
	Proportion of fluoroquinolones used in companion animals	Quantitative	Animal Health	Stewardship
	Proportion of tetracyclines used in companion animals	Quantitative	Animal Health	Stewardship
	Proportion of aminoglycosides used in companion animals	Quantitative	Animal Health	Stewardship
	Proportion of polypeptides used in companion animals	Quantitative	Animal Health	Stewardship
	Proportion of lincosamides used in companion animals	Quantitative	Animal Health	Stewardship
	Proportion of pleuromutilins used in companion animals	Quantitative	Animal Health	Stewardship
	Proportion of sulfonamides (including trimethoprim) used in companion animals	Quantitative	Animal Health	Stewardship
	Proportion of amphenicols used in companion animals	Quantitative	Animal Health	Stewardship
	Proportion of cephalosporins (all generations) used in companion animals	Quantitative	Animal Health	Stewardship
	Proportion of 1-2 gen cephalosporins used in companion animals	Quantitative	Animal Health	Stewardship
	Proportion of 3-4 gen cephalosporins used in companion animals	Quantitative	Animal Health	Stewardship
	Proportion of macrolides used in companion animals	Quantitative	Animal Health	Stewardship
	Proportion of other antibiotic classes used in companion animals	Quantitative	Animal Health	Stewardship
	Proportion of nitrofurans used in companion animals	Quantitative	Animal Health	Stewardship
	Proportion of streptogramins used in companion animals	Quantitative	Animal Health	Stewardship
	Proportion of Sulphonamides (including trimethoprim) used in terrestrial food-producing animals	Quantitative	Animal Health	Stewardship
	Proportion of antimicrobial classes reported for use in animals (penicillin)	Quantitative	Animal Health	Stewardship
	Proportion of antimicrobial classes reported for use	Quantitative	Animal Health	Stewardship

in animals (sulphonamides - including trimethoprim)			
Proportion of sulphonamides (including trimethoprim) used by aquatic food-producing animals	Quantitative	Animal Health	Stewardship
Proportion of sulphonamides (including trimethoprim) used in companion animals	Quantitative	Animal Health	Stewardship
Proportional route of administration (oral, injection, other routes) for sulphonamides (including trimethoprim)	Quantitative	Animal Health	Stewardship
Quantities of sulphonamides (including trimethoprim) used, adjusted by animal biomass (mg/kg)	Quantitative	Animal Health	Stewardship
Reported use of Nosiheptide as a growth promoter	Quantitative	Animal Health	Stewardship
Proportional route of administration (oral, injection, other routes) for aminoglycosides	Quantitative	Animal Health	Stewardship
Proportional route of administration (oral, injection, other routes) for amphenicols	Quantitative	Animal Health	Stewardship
Proportional route of administration (oral, injection, other routes) for arsenicals	Quantitative	Animal Health	Stewardship
Proportional route of administration (oral, injection, other routes) for cephalosporins (all generations)	Quantitative	Animal Health	Stewardship
Proportional route of administration (oral, injection, other routes) for cephalosporins (1-2 gen)	Quantitative	Animal Health	Stewardship
Proportional route of administration (oral, injection, other routes) for sulfonamides (including trimethoprim)	Quantitative	Animal Health	Stewardship
Proportional route of administration (oral, injection, other routes) for cephalosporins (3-4 gen)	Quantitative	Animal Health	Stewardship
Proportional route of administration (oral, injection, other routes) for fluoroquinolones	Quantitative	Animal Health	Stewardship
Proportional route of administration (oral, injection, other routes) for glycophospholipids	Quantitative	Animal Health	Stewardship
Proportional route of administration (oral, injection, other routes) for lincosamides	Quantitative	Animal Health	Stewardship

	Proportional route of administration (oral, injection, other routes) for macrolides	Quantitative	Animal Health	Stewardship
	Proportional route of administration (oral, injection, other routes) for nitrofurans	Quantitative	Animal Health	Stewardship
	Proportional route of administration (oral, injection, other routes) for orthosomycins	Quantitative	Animal Health	Stewardship
	Proportional route of administration (oral, injection, other routes) for other quinolones	Quantitative	Animal Health	Stewardship
	Proportional route of administration (oral, injection, other routes) for other antimicrobial classes	Quantitative	Animal Health	Stewardship
	Proportional route of administration (oral, injection, other routes) for penicillins	Quantitative	Animal Health	Stewardship
	Proportional route of administration (oral, injection, other routes) for pleuromutilins	Quantitative	Animal Health	Stewardship
	Proportional route of administration (oral, injection, other routes) for polypeptides	Quantitative	Animal Health	Stewardship
	Proportional route of administration (oral, injection, other routes) for quinoxalines	Quantitative	Animal Health	Stewardship
	Proportional route of administration (oral, injection, other routes) for streptogramins	Quantitative	Animal Health	Stewardship
	Proportional route of administration (oral, injection, other routes) for tetracyclines	Quantitative	Animal Health	Stewardship
	Species composition of animal biomass (bovine) - %	Quantitative	Animal Health	Reporting
	Species composition of animal biomass (swine) - %	Quantitative	Animal Health	Reporting
	Species composition of animal biomass (poultry) - %	Quantitative	Animal Health	Reporting
	Species composition of animal biomass (sheep) - %	Quantitative	Animal Health	Reporting
	Species composition of animal biomass (fish) - %	Quantitative	Animal Health	Reporting
	Species composition of animal biomass (goats) - %	Quantitative	Animal Health	Reporting
	Species composition of animal biomass (equidae) - %	Quantitative	Animal Health	Reporting
	Species composition of animal biomass (molluscs) - %	Quantitative	Animal Health	Reporting
	Species composition of animal biomass	Quantitative	Animal Health	Reporting

	(camelidae) - %			
	Species composition of animal biomass (crustaceans) - %	Quantitative	Animal Health	Reporting
	Quantities of antimicrobial agents intended for use in animals (mg/kg) - not adjusted by reported coverage	Quantitative	Animal Health	Stewardship
	Quantities of antimicrobial agents intended for use in animals (mg/kg) - adjusted by reported coverage	Quantitative	Animal Health	Stewardship
	Quantities of tetracyclines used, adjusted by animal biomass (mg/kg)	Quantitative	Animal Health	Stewardship
	Quantities of penicillins used, adjusted by animal biomass (mg/kg)	Quantitative	Animal Health	Stewardship
	Quantities of macrolides used, adjusted by animal biomass (mg/kg)	Quantitative	Animal Health	Stewardship
	Quantities of polypeptides used, adjusted by animal biomass (mg/kg)	Quantitative	Animal Health	Stewardship
	Quantities of pleuromutilins used, adjusted by animal biomass (mg/kg)	Quantitative	Animal Health	Stewardship
	Quantities of sulfonamides (including trimethoprim) used, adjusted by animal biomass (mg/kg)	Quantitative	Animal Health	Stewardship
	Quantities of amphenicols used, adjusted by animal biomass (mg/kg)	Quantitative	Animal Health	Stewardship
	Quantities of aminoglycosides used, adjusted by animal biomass (mg/kg)	Quantitative	Animal Health	Stewardship
	Quantities of fluoroquinolones used, adjusted by animal biomass (mg/kg)	Quantitative	Animal Health	Stewardship
	Quantities of quinoxalines used, adjusted by animal biomass (mg/kg)	Quantitative	Animal Health	Stewardship
	Quantities of lincosamides used, adjusted by animal biomass (mg/kg)	Quantitative	Animal Health	Stewardship
	Quantities of cephalosporins (all generations) used, adjusted by animal biomass (mg/kg)	Quantitative	Animal Health	Stewardship
	Quantities of cephalosporins (1-2 gen) used, adjusted by animal biomass (mg/kg)	Quantitative	Animal Health	Stewardship
	Quantities of cephalosporins (3-4 gen) used, adjusted by animal biomass (mg/kg)	Quantitative	Animal Health	Stewardship
	Quantities of streptogramins used, adjusted by animal biomass (mg/kg)	Quantitative	Animal Health	Stewardship

		Quantities of other antimicrobial classes used, adjusted by animal biomass (mg/kg)	Quantitative	Animal Health	Stewardship
		Quantities of glycophospholipids used, adjusted by animal biomass (mg/kg)	Quantitative	Animal Health	Stewardship
		Quantities of orthosomycins used, adjusted by animal biomass (mg/kg)	Quantitative	Animal Health	Stewardship
		Quantities of arsenicals used, adjusted by animal biomass (mg/kg)	Quantitative	Animal Health	Stewardship
		Quantities of other quinolones used, adjusted by animal biomass (mg/kg)	Quantitative	Animal Health	Stewardship
		Quantities of nitrofurans used, adjusted by animal biomass (mg/kg)	Quantitative	Animal Health	Stewardship
		Quantities of glycopeptides used, adjusted by animal biomass (mg/kg)	Quantitative	Animal Health	Strategic Vision
13	OECD Embracing a One Health Framework to Fight Antimicrobial Resistance	OECD Long-Term Care Facility survey: NAP integrates LCTFs (mentioned in plan; no mention but in other relevant legislation; next NAP will mention LTCFs, but current does not and no other relevant legislation; other/don't know)	Qualitative	Human Health	Stewardship
		OECD Long-Term Care Facility survey: AMR guidelines and budget (antimicrobial guidelines or restrictive lists for antimicrobials in LCTS and a specific budget dedicated to LCTS; antimicrobial guidelines or restrictive lists for antimicrobials in LCTFs but no budget dedicated to LTCFs; neither guidelines nor restrictive lists for antimicrobials in LCTFs; other/don't know)	Qualitative	Human Health	Prevention & Control
		OECD Long-Term Care Facility survey: IPC programmes and budget (IPC programme and a dedicated IPC budget; IPC programme but no dedicated IPC budget; IPC programme but dedicated budget unknown; no IPC programme)	Qualitative	Human Health	Prevention & Control
		OECD Long-Term Care Facility survey: AMR surveillance in LCTFs (surveillance programmes both for antimicrobial stewardship and IPC; surveillance programmes for IPC but not for antimicrobial stewardship; no surveillance programme for IPC or antimicrobial stewardship)	Qualitative	Human Health	Prevention & Control
		Percentage of population aged 65 and over	Quantitative	Human Health	Sustainability

		vaccinated for influenza			
		% of AMR-related development assistance for health	Quantitative	Multisectoral	Stewardship
		Share of medical records with no clear documentation of indication for antimicrobial prescription, various years	Quantitative	Human Health	Access to Medicines & Health Services
		Occupancy rate of curative (acute) care beds	Quantitative	Human Health	Stewardship
		Sales volume of fungicides and bactericides	Quantitative	Multisectoral	Strategic Vision
		AMR-NAP mentions LTCFs (yes/no/no answer)	Categorical	Human Health	Strategic Vision
		Next AMR-NAP will mention LCTFs (yes/no/no answer)	Categorical	Human Health	Regulations & Legislation
		Legislation, policies and/or programmes to address AMR in LTCFs (yes/no/no answer)	Categorical	Human Health	Surveillance & Laboratory
		Monitoring and evaluation of AMR in LCTFs (yes/no/no answer)	Categorical	Human Health	Feedback Mechanisms
		Quality audits of LTCFs include AMR (yes/no/no answer)	Categorical	Human Health	Prevention & Control
14	Assessment tool of the minimum requirements for infection prevention and control programmes at the national level	An active infection prevention and control programme exists at the national level	Y/N	Multisectoral	Prevention & Control
		An appointed IPC focal point in charge of the programme can be identified	Y/N	Multisectoral	Prevention and Control
		The appointed IPC focal point(s) have undergone training in IPC in the prevention of health care associated infections	Y/N	Human Health	Prevention & Control
		There is an identified, protected and dedicated budget allocated to the IPC programme, according to planned activity	Y/N	Multisectoral	Prevention & Control
		The appointed IPC focal point(s) have dedicated time for the tasks (at least one full-time equivalent)	Y/N	Multisectoral	Prevention & Control
		The national IPC programme has a mandate to produce guidelines for preventing and controlling HAI	Y/N	Human Health	Prevention & Control
		The development of guidelines involves the use of evidence-based scientific knowledge and international/national standards	Y/N	Multisectoral	Prevention & Control
		The guidelines are for national coverage, including all acute health care facilities (both public and private)	Y/N	Human Health	Prevention & Control

The guidelines are reviewed at least once every five years and updated to reflect the current evidence base	Y/N	Multisectoral	Workforce
The national IPC programme provides guidance and recommendations for in-service IPC training at the facility level (for example, frequency, expertise required, requirements for new employee orientation, monitoring and evaluation approaches)	Y/N	Human Health	Workforce
The national IPC programme provides content and support for IPC training of health workers at the facility level	Y/N	Human Health	Workforce
A national IPC curriculum for in-service training of health care workers has been developed in alignment with the national IPC guidelines, approved and endorsed by an appropriate national body	Y/N	Human Health	Workforce
A national system and schedule of monitoring and evaluation is in place to check on the effectiveness of training and education, at least annually	Y/N	Human Health	Workforce
A multidisciplinary technical group for HAI surveillance is established at the national level by the national IPC focal point	Y/N	Human Health	Prevention & Control
A national strategic plan for HAI surveillance (with a focus on priority infections based on the local context) is developed by the multidisciplinary technical group.	Y/N	Human Health	Workforce
The national IPC focal point/team is trained in HAI surveillance concepts and methods	Y/N	Human Health	Workforce
A multidisciplinary technical group for IPC monitoring is established at the national level	Y/N	Multisectoral	Feedback Mechanisms
A strategic plan for IPC monitoring is in place, including an integrated system for collection, analysis and feedback of data	Y/N	Multisectoral	Prevention & Control
A minimal set of core indicators for health care facilities in the country is defined	Y/N	Multisectoral	Workforce
A mechanism to train national and local auditors is in place	Y/N	Multisectoral	Prevention & Control
Hand hygiene compliance monitoring and feedback is identified as a key national indicator, at	Y/N	Human Health	Prevention & Control

		the very least for reference hospitals.			
15	IPCAT 2	An active IPC programme exists at the national level	Y/N	Human Health	Workforce / Prevention & Control
		An appointed infection preventionist(s) in charge of the programme can be identified	Y/N	Human Health	Workforce / Prevention & Control
		The appointed technical team of infection preventionist(s) includes both doctors and nurses	Y/N	Human Health	Workforce / Prevention & Control
		The appointed infection preventionist(s) have undergone training in IPC in the prevention of health care-associated infection (HAI)	Y/N	Human Health	Workforce / Prevention & Control
		The appointed infection preventionist(s) have dedicated time for the tasks (at least one full-time person)	Y/N	Human Health	Accountability / Prevention & Control
		The programme has been granted authority to make decisions that influence field implementation	Y/N	Human Health	Sustainability / Prevention & Control
		There is an identified, protected and dedicated budget allocated according to planned activity	Y/N	Human Health	Workforce / Prevention & Control
		An official multidisciplinary group/committee or equivalent structure is established to support the IPC team at the national level (for example, national IPC committee)	Y/N	Human Health	Prevention & Control
		The scope of IPC responsibilities is defined and includes development of national policies, guidelines and standards for effective, evidence-based practices	Y/N	Human Health	Prevention & Control
		The scope of IPC responsibilities is defined and includes development of a national plan for preventing HAIs relating to endemic pathogens and those with epidemic potential, for example, including national goals, objectives and strategies	Y/N	Human Health	Surveillance & Laboratory / Prevention & Control
		The scope of IPC responsibilities is defined and includes development of national monitoring frameworks to measure implementation with policies, guidelines and standards	Y/N	Human Health	Workforce / Prevention & Control
		The scope of IPC responsibilities is defined and includes development and support of IPC training and educational programmes to support the facility level	Y/N	Human Health	Surveillance & Laboratory / Prevention & Control
		The scope of IPC responsibilities is defined and	Y/N	Human Health	Surveillance & Laboratory /

	includes surveillance and epidemiology of HAI and HAI-related aspects of antimicrobial resistance in collaboration with epidemiologists, data managers and information technology experts			Prevention & Control
	The scope of IPC responsibilities is defined and includes a national plan to support early detection of HAI outbreaks and prompt and effective response	Y/N	Human Health	Prevention & Control / Prevention & Control
	The scope of IPC responsibilities is defined and includes assurance of national procurement of adequate supplies for IPC practices, including access to essential infrastructures, materials and equipment necessary for safe IPC practice	Y/N	Human Health	Coordination / Prevention & Control
	Clear linkages (including routine communications) between IPC and other programmes and professional organizations: other national programmes, for example, AMR, quality and safety, water, sanitation and hygiene, environment, tuberculosis, human immunodeficiency virus, immunization, maternal, child and adolescent health	Y/N	Human Health	Coordination / Prevention & Control
	Clear linkages (including routine communications) between IPC and other programmes and professional organizations: priority public health programmes including integration of IPC with IHR and preparedness relating to public health emergencies	Y/N	Human Health	Coordination / Surveillance & Laboratory / Prevention & Control
	Clear linkages (including routine communications) between IPC and other programmes and professional organizations: national referral laboratories and laboratory biosafety	Y/N	Human Health	Coordination / Prevention & Control
	Clear linkages (including routine communications) between IPC and other programmes and professional organizations: occupational health programmes	Y/N	Human Health	Coordination / Prevention & Control
	Clear linkages (including routine communications) between IPC and other programmes and professional organizations: patient associations/civil society bodies	Y/N	Human Health	Coordination / Workforce / Prevention & Control

	Clear linkages (including routine communications) between IPC and other programmes and professional organizations: scientific professional organizations (for example, IPC professional societies and other relevant medical, nursing and allied health professional societies)	Y/N	Human Health	Coordination / Workforce / Prevention & Control
	Clear linkages (including routine communications) between IPC and other programmes and professional organizations: training establishments and academia	Y/N	Human Health	Coordination / Prevention & Control
	Clear linkages (including routine communications) between IPC and other programmes and professional organizations: relevant sub-national bodies, for example, provisional or district health offices	Y/N	Human Health	Prevention & Control
	The IPC programme has a mandate to produce guidelines for preventing and controlling HAI	Y/N	Human Health	Prevention & Control
	The guidelines are reviewed at least every five years and updated to reflect the current evidence base	Y/N	Human Health	Prevention & Control
	The IPC programme has the necessary expertise to develop national guidelines	Y/N	Human Health	Prevention & Control
	The IPC programme actively addresses guideline adaptation and standardization of effective preventive practices (standard operating procedures) and their implementation to reflect local conditions	Y/N	Human Health	Prevention & Control
	Guideline development involves early engagement of key stakeholders, including involvement of programmes closely linked to IPC	Y/N	Human Health	Strategic Vision / Prevention & Control
	The IPC programme develops multimodal implementation strategies using available national/international implementation support packages	Y/N	Human Health	Prevention & Control
	The IPC programme has the capability to ensure that the infrastructure and supply-related requirements to enable facility-level guideline implementation are in place/being addressed	Y/N	Human Health	Workforce / Prevention & Control
	The IPC programme supports and mandates a	Y/N	Human Health	Workforce / Prevention & Control

programme of health worker education and training on guideline recommendations across all facilities			
The IPC programme supports and mandates a programme of health worker education and training on guideline recommendations at the pre-graduate level	Y/N	Human Health	Workforce / Prevention & Control
The IPC programme supports and mandates a programme of health worker education and training on guideline recommendations at the postgraduate level	Y/N	Human Health	Feedback Mechanisms / Prevention & Control
A national system and schedule of monitoring and evaluation is in place to check on adherence with guideline recommendations, for example, at least annually	Y/N	Human Health	Prevention & Control
National guidelines are based on local priorities, frequency of practices and practices associated with the populations most at risk of HAI	Y/N	Human Health	Prevention & Control
Basic/essential guidelines have been developed based on/adapted from international standards	Y/N	Human Health	Prevention & Control
Specific guidelines to prevent the most prevalent HAIs (catheter-associated urinary tract infection, central line-associated bloodstream infection, surgical site infection, ventilator-associated infection) have been developed, depending on the context and complexity of care required	Y/N	Human Health	Workforce / Prevention & Control
The national IPC programme provides guidance and recommendations for in-service training at the facility level (for example, frequency, expertise required, requirements for new employee orientation, monitoring and evaluation approaches)	Y/N	Human Health	Workforce / Prevention & Control
The national IPC programme provides content and support for IPC training of all health workers at the facility level	Y/N	Human Health	Workforce / Prevention & Control
The national IPC programme provides content and support for other personnel that support health service delivery**	Y/N	Human Health	Workforce / Prevention & Control
The national IPC programme provides content and support for the training of IPC professionals to	Y/N	Human Health	Surveillance & Laboratory / Prevention & Control

support competence development/development of an IPC career pathway			
The national IPC programme provides content and support to undertake national HAI surveillance	Y/N	Human Health	Workforce / Prevention & Control
National IPC curricula, developed (or under development) in collaboration with local academic institutions are available for pre-graduate courses	Y/N	Human Health	Workforce / Prevention & Control
National IPC curricula, developed (or under development) in collaboration with local academic institutions is available for postgraduate courses	Y/N	Human Health	Workforce / Prevention & Control
National curricula are informed by international curricula/networks and adapted to national needs and local resources	Y/N	Human Health	Workforce / Prevention & Control
National curricula are adapted to national needs and local resources	Y/N	Human Health	Workforce / Prevention & Control
IPC training is integrated into continuing medical, nursing and allied health professional education and training	Y/N	Human Health	Workforce / Prevention & Control
A national system and schedule of monitoring and evaluation is in place to check on the effectiveness of training and education, for example, at least annually	Y/N	Human Health	Workforce / Prevention & Control
Standardized training tools in line with national guidelines and international standards to support implementation of curricula are available	Y/N	Human Health	Workforce / Prevention & Control
The national IPC training supports packages to promote the use of participatory and team- and task-based strategies	Y/N	Human Health	Workforce / Prevention & Control
The national IPC training supports packages to promote the use of simulation	Y/N	Human Health	Workforce / Prevention & Control
The national IPC training supports packages to promote the use of multimodal strategies	Y/N	Human Health	Workforce / Prevention & Control
The national IPC training supports packages to promote the integration and embedding of IPC training within clinical practice and the training of other disciplines	Y/N	Human Health	Workforce / Prevention & Control
The national IPC training supports packages to promote the importance of involving patients or family members in facility-level training	Y/N	Human Health	Surveillance & Laboratory / Prevention & Control

	programmes			
	A national HAI surveillance programme and network of facilities is established and supported (including financially) by governments and national authorities	Y/N	Human Health	Workforce / Prevention & Control
	The national IPC team is trained in HAI surveillance concepts and methods	Y/N	Human Health	Workforce / Prevention & Control
	The national IPC programme (or collaborating partner) leads are designated to coordinate the national HAI surveillance programme and network	Y/N	Human Health	Surveillance & Laboratory / Prevention & Control
	The national IPC programme collects a representative sample of data on HAI at the country level or in selected regions according to feasibility, including the use of trained data collectors	Y/N	Human Health	Surveillance & Laboratory / Prevention & Control
	The national HAI surveillance programme links with AMR surveillance systems	Y/N	Human Health	Surveillance & Laboratory / Prevention & Control
	The national HAI surveillance programme links with the national public health bodies responsible for International Health Regulations to ensure timely detection of outbreaks	Y/N	Human Health	Surveillance & Laboratory / Prevention & Control
	National HAI surveillance data are used for benchmarking purposes (for example, establishing baselines for comparison)	Y/N	Human Health	Surveillance & Laboratory / Prevention & Control
	National objectives of surveillance are defined and include: Describing the epidemiology of HAI (that is, incidence and/or prevalence, type, aetiology, severity, burden of disease)	Y/N	Human Health	Surveillance & Laboratory / Prevention & Control
	National objectives of surveillance are defined and include: Identification of risk factors, for example, high-risk populations, procedures and exposures	Y/N	Human Health	Surveillance & Laboratory / Prevention & Control
	National objectives of surveillance are defined and include: Early detection of outbreaks	Y/N	Human Health	Surveillance & Laboratory / Prevention & Control
	National objectives of surveillance are defined and include: Informing policy priorities	Y/N	Human Health	Surveillance & Laboratory / Prevention & Control
	National objectives of surveillance are defined and include: Assessment of the impact of IPC interventions	Y/N	Human Health	Surveillance & Laborator / Prevention & Control
	Prioritized HAIs for surveillance are defined and	Y/N	Human Health	Surveillance & Laboratory /

include: Epidemic-prone infections (for example, norovirus, influenza, severe acute respiratory syndrome)			Prevention & Control
Prioritized HAIs for surveillance are defined and include: Infections in vulnerable populations (for example, neonates, burn patients, intensive care unit patients, immunocompromised hosts)	Y/N	Human Health	Surveillance & Laboratory / Prevention & Control
Prioritized HAIs for surveillance are defined and include: Infections that may cause severe outcomes	Y/N	Human Health	Surveillance & Laboratory / Prevention & Control
Prioritized HAIs for surveillance are defined and include: Infections caused by multidrug-resistant, extensive drug-resistant and pan drug pathogens (for example, WHO priority/Global Antimicrobial Surveillance Systems**)	Y/N	Human Health	Surveillance & Laboratory / Prevention & Control
Prioritized HAIs for surveillance are defined and include: Infections associated with invasive devices or specific procedures (for example, intravascular devices, surgery, etc.)	Y/N	Human Health	Surveillance & Laboratory / Prevention & Control
Prioritized HAIs for surveillance are defined and include: Infections that may affect health care workers in clinical, laboratory and other settings (for example, hepatitis B or C, human immunodeficiency virus, influenza)	Y/N	Human Health	Surveillance & Laboratory / Prevention & Control
Methods of surveillance are defined and include: Standardized active prospective data collection methods	Y/N	Human Health	Surveillance & Laboratory / Prevention & Control
Methods of surveillance are defined and include: Standardized case definitions of infections (including accurate denominators) informed by international standards, careful local expert consultation and validation	Y/N	Human Health	Surveillance & Laboratory / Prevention & Control
Methods of surveillance are defined and include: Systems to regularly assess data quality (for example, review of case report forms, microbiology results, denominator determination) and surveillance programme attributes (for example, sensitivity, specificity, user-acceptability)	Y/N	Human Health	Surveillance & Laboratory / Prevention & Control
The national IPC programme has microbiological support to monitor certain organisms (at least one	Y/N	Human Health	Surveillance & Laboratory / Prevention & Control

	national reference microbiology laboratory)			
	Microbiological data on the etiology and patterns of AMR (at least for prioritized HAIs, for example, most severe infections)	Y/N	Human Health	Reporting / Prevention & Control
	Clear and regular reporting lines from facility to the national level are in place	Y/N	Human Health	Surveillance & Laboratory / Prevention & Control
	National IPC programme has a clear plan for data management and analysis at the national level	Y/N	Human Health	Feedback Mechanisms / Prevention & Control
	National IPC programme provides timely feedback reports to relevant stakeholders on the national situation of HAI and special events	Y/N	Human Health	Feedback Mechanisms / Prevention & Control
	National IPC programme provides timely feedback reports to relevant stakeholders on outbreak management and control	Y/N	Human Health	Feedback Mechanisms / Prevention & Control
	National IPC programme provides timely feedback reports to relevant stakeholders on HAI caused by multidrug-resistant pathogens	Y/N	Human Health	Surveillance & Laboratory / Prevention & Control
	HAI surveillance data are linked with available IPC and water, sanitation and hygiene monitoring data	Y/N	Human Health	Feedback Mechanisms / Prevention & Control
	Feedback reports from the national level to relevant stakeholders contain both analyses and recommendations	Y/N	Human Health	Workforce / Prevention & Control
	National and sub-national coordination in support of local implementation of IPC improvement interventions includes: A trained national IPC team, competent in implementation science and multimodal behaviour change strategies**	Y/N	Human Health	Prevention & Control / Workforce
	National and sub-national coordination in support of local implementation of IPC improvement interventions includes: Promotion of multimodal strategies through the inclusion of the approach in the development of IPC guidelines, education and training	Y/N	Human Health	Prevention & Control
	National and sub-national facilitation in support of local implementation of IPC improvement interventions includes: Promotion of actions to ensure that the infrastructure/necessary supplies for IPC are in place (system change)	Y/N	Human Health	Workforce / Prevention & Control

National and sub-national facilitation in support of local implementation of IPC improvement interventions includes: Promotion of health care worker training and education relevant to IPC interventions is being implemented	Y/N	Human Health	Feedback Mechanisms / Prevention & Control
National and sub-national facilitation in support of local implementation of IPC improvement interventions includes: Promotion of the development of monitoring indicators (process or outcome) reflecting the IPC improvement interventions is being implemented, including provision of feedback data	Y/N	Human Health	Feedback Mechanisms / Prevention & Control
National and sub-national facilitation in support of local implementation of IPC improvement interventions includes: Promotion of the role of communications and reminders/awareness-raising resources relating to the IPC improvements is being implemented	Y/N	Human Health	Feedback Mechanisms / Prevention & Control
National and sub-national facilitation in support of local implementation of IPC improvement interventions includes: Promotion of organizational culture change	Y/N	Human Health	Coordination / Prevention & Control
Programme and accreditation linkages include: Liaison between national IPC programme and quality improvement/quality and safety departments to promote multimodal strategies	Y/N	Human Health	Coordination / Prevention & Control
Programme and accreditation linkages include: Liaison between national IPC programme and accreditation bodies to promote multimodal strategies	Y/N	Human Health	Feedback Mechanisms / Effectiveness
Evaluation of multimodal strategies includes: A system for regular reporting and evaluation on multimodal strategies across health facilities, including feedback	Y/N	Human Health	Prevention & Control
Monitoring/audit and feedback framework for IPC is established at national level, including: A well-defined plan focusing on IPC outcomes, processes and strategies, with clear goals, targets and operational plans	Y/N	Human Health	Feedback Mechanisms / Prevention & Control

Monitoring/audit and feedback framework for IPC is established at national level, including: IPC indicators integrated within national monitoring systems, for example, health management information system	Y/N	Human Health	Feedback Mechanisms / Prevention & Control
Monitoring/audit and feedback framework for IPC is established at national level, including: Development of tools to collect information needed for monitoring/audit and feedback in a systematic way including the WHO hand hygiene self-assessment framework	Y/N	Human Health	Feedback Mechanisms / Coordination / Prevention & Control
Monitoring/audit and feedback framework for IPC is established at national level, including: National monitoring/audit and feedback activities aligned with equivalent activities at the local level (focused on core IPC indicators)	Y/N	Human Health	Workforce / Prevention & Control
Monitoring/audit and feedback framework for IPC is established at national level, including: A mechanism to train national and local auditors is in place	Y/N	Human Health	Feedback Mechanisms / Prevention & Control
Monitoring/audit and feedback framework for IPC is established at national level, including: Mechanisms to link/cross-reference IPC monitoring/audit data with available water, sanitation and hygiene monitoring data	Y/N	Human Health	Prevention & Control
Hand hygiene compliance monitoring and feedback is identified as a key national indicator, at the very least for reference hospitals	Y/N	Human Health	Effectiveness / Prevention & Control
All indicators are linked to the targets established by the national IPC work plan	Y/N	Human Health	Prevention & Control
Core indicators include both process and outcome indicators (for example, focused on structures/infrastructure and the environment as well as practices of health care workers)	Y/N	Human Health	Prevention & Control
Information on the monitoring/audit of national IPC goals and strategies is collected regularly	Y/N	Multisectoral	Feedback Mechanisms / Prevention & Control
Monitoring/audit of IPC activities and structures of health care facilities is conducted regularly	Y/N	Human Health	Effectiveness / Prevention & Control
Information collected is regularly analysed and	Y/N	Human Health	Feedback Mechanisms /

		used to inform national decision making			Prevention & Control
		Evaluation of the performance of local IPC programmes is performed in an improvement-oriented institutional culture	Y/N	Human Health	Feedback Mechanisms / Prevention & Control
		The IPC national programme facilitates facility-level self or peer evaluation against national standards/goals	Y/N	Human Health	Feedback Mechanisms / Prevention & Control
		Regular reports of monitoring/audit results are provided to drive improvement action at the facility level as part of a multimodal strategy	Y/N	Human Health	Stewardship
16	One Health Trust Resistance Map	Antibiotic use: broad spectrum penicillins (DDD / 1000 pop)	Quantitative	Human Health	Stewardship
		Antibiotic use: macrolides (DDD / 1000 pop)	Quantitative	Human Health	Stewardship
		Antibiotic use: cephalosporins (DDD / 1000 pop)	Quantitative	Human Health	Stewardship
		Antibiotic use: fluoroquinolones (DDD / 1000 pop)	Quantitative	Human Health	Stewardship
		Antibiotic use: aminoglycosides (DDD / 1000 pop)	Quantitative	Human Health	Stewardship
		Antibiotic use: carbapenems (DDD / 1000 pop)	Quantitative	Human Health	Stewardship
		Antibiotic use: chloramphenicols (DDD / 1000 pop)	Quantitative	Human Health	Stewardship
		Antibiotic use: glycopeptides (DDD / 1000 pop)	Quantitative	Human Health	Stewardship
		Antibiotic use: glycylcyclines (DDD / 1000 pop)	Quantitative	Human Health	Stewardship
		Antibiotic use: lipopeptides (DDD / 1000 pop)	Quantitative	Human Health	Stewardship
		Antibiotic use: monobactams (DDD / 1000 pop)	Quantitative	Human Health	Stewardship
		Antibiotic use: narrow spectrum penicillins (DDD / 1000 pop)	Quantitative	Human Health	Stewardship
		Antibiotic use: others (DDD / 1000 pop)	Quantitative	Human Health	Stewardship
		Antibiotic use: oxazolidinones (DDD / 1000 pop)	Quantitative	Human Health	Stewardship
		Antibiotic use: phosphonics (DDD / 1000 pop)	Quantitative	Human Health	Stewardship
		Antibiotic use: polymyxins (DDD / 1000 pop)	Quantitative	Human Health	Stewardship
		Antibiotic use: rifampicin (DDD / 1000 pop)	Quantitative	Human Health	Stewardship
		Antibiotic use: tetracyclines (DDD / 1000 pop)	Quantitative	Human Health	Stewardship
		Antibiotic use: trimethoprim (DDD / 1000 pop)	Quantitative	Human Health	Surveillance & Laboratory

	% resistant (invasive isolates): Acinetobacter baumannii / Amikacin	Quantitative	Human Health	Surveillance & Laboratory
	% resistant (invasive isolates): Acinetobacter baumannii / Aminoglycosides	Quantitative	Human Health	Surveillance & Laboratory
	% resistant (invasive isolates): Acinetobacter baumannii / Aminoglycosides (high-level)	Quantitative	Human Health	Surveillance & Laboratory
	% resistant (invasive isolates): Acinetobacter baumannii / Aminopenicillins	Quantitative	Human Health	Surveillance & Laboratory
	% resistant (invasive isolates): Acinetobacter baumannii / Ampicillin-sulbactam	Quantitative	Human Health	Surveillance & Laboratory
	% resistant (invasive isolates): Acinetobacter baumannii / Carbapenems	Quantitative	Human Health	Surveillance & Laboratory
	% resistant (invasive isolates): Acinetobacter baumannii / Ceftazidime	Quantitative	Human Health	Surveillance & Laboratory
	% resistant (invasive isolates): Acinetobacter baumannii / Cephalosporins (3rd gen)	Quantitative	Human Health	Surveillance & Laboratory
	% resistant (invasive isolates): Acinetobacter baumannii / Fluoroquinolones	Quantitative	Human Health	Surveillance & Laboratory
	% resistant (invasive isolates): Acinetobacter baumannii / Glycylcyclines	Quantitative	Human Health	Surveillance & Laboratory
	% resistant (invasive isolates): Acinetobacter baumannii / Piperacillin-tazobactam	Quantitative	Human Health	Surveillance & Laboratory
	% resistant (invasive isolates): Acinetobacter baumannii / Polymyxins	Quantitative	Human Health	Surveillance & Laboratory
	% resistant (invasive isolates): Acinetobacter baumannii / Tetracyclines	Quantitative	Human Health	Surveillance & Laboratory
	% resistant (invasive isolates): Acinetobacter baumannii / Trimethoprim-sulfamethoxazole	Quantitative	Human Health	Surveillance & Laboratory
	% resistant (invasive isolates): Enterobacter aerogenes/cloacae / Aminoglycosides	Quantitative	Human Health	Surveillance & Laboratory
	% resistant (invasive isolates): Enterobacter aerogenes/cloacae / Amoxicillin-clavulanate	Quantitative	Human Health	Surveillance & Laboratory
	% resistant (invasive isolates): Enterobacter aerogenes/cloacae / Carbapenems	Quantitative	Human Health	Surveillance & Laboratory
	% resistant (invasive isolates): Enterobacter aerogenes/cloacae / Cephalosporins (3rd gen)	Quantitative	Human Health	Surveillance & Laboratory
	% resistant (invasive isolates): Enterobacter aerogenes/cloacae / Fluoroquinolones	Quantitative	Human Health	Surveillance & Laboratory
	% resistant (invasive isolates): Enterobacter	Quantitative	Human Health	Surveillance & Laboratory

	aerogenes/cloacae / Glycylcyclines			
	% resistant (invasive isolates): Enterobacter aerogenes/cloacae / Piperacillin-tazobactam	Quantitative	Human Health	Surveillance & Laboratory
	% resistant (invasive isolates): Enterobacter aerogenes/cloacae / Polymyxins	Quantitative	Human Health	Surveillance & Laboratory
	% resistant (invasive isolates): Enterococcus faecalis / Aminoglycosides	Quantitative	Human Health	Surveillance & Laboratory
	% resistant (invasive isolates): Enterococcus faecalis / Aminoglycosides (high-level)	Quantitative	Human Health	Surveillance & Laboratory
	% resistant (invasive isolates): Enterococcus faecalis / Aminopenicillins	Quantitative	Human Health	Surveillance & Laboratory
	% resistant (invasive isolates): Enterococcus faecalis / Fluoroquinolones	Quantitative	Human Health	Surveillance & Laboratory
	% resistant (invasive isolates): Enterococcus faecalis / Linezolid	Quantitative	Human Health	Surveillance & Laboratory
	% resistant (invasive isolates): Enterococcus faecalis / Vancomycin	Quantitative	Human Health	Surveillance & Laboratory
	% resistant (invasive isolates): Enterococcus faecium / Aminoglycosides	Quantitative	Human Health	Surveillance & Laboratory
	% resistant (invasive isolates): Enterococcus faecium / Aminoglycosides (high-level)	Quantitative	Human Health	Surveillance & Laboratory
	% resistant (invasive isolates): Enterococcus faecium / Aminopenicillins	Quantitative	Human Health	Surveillance & Laboratory
	% resistant (invasive isolates): Enterococcus faecium / Fluoroquinolones	Quantitative	Human Health	Surveillance & Laboratory
	% resistant (invasive isolates): Enterococcus faecium / Linezolid	Quantitative	Human Health	Surveillance & Laboratory
	% resistant (invasive isolates): Enterococcus faecium / Penicillins	Quantitative	Human Health	Surveillance & Laboratory
	% resistant (invasive isolates): Enterococcus faecium / Vancomycin	Quantitative	Human Health	Surveillance & Laboratory
	% resistant (invasive isolates): Escherichia coli / Amikacin	Quantitative	Human Health	Surveillance & Laboratory
	% resistant (invasive isolates): Escherichia coli / Aminoglycosides	Quantitative	Human Health	Surveillance & Laboratory
	% resistant (invasive isolates): Escherichia coli / Aminopenicillins	Quantitative	Human Health	Surveillance & Laboratory
	% resistant (invasive isolates): Escherichia coli / Amoxicillin-clavulanate	Quantitative	Human Health	Surveillance & Laboratory

% resistant (invasive isolates): Escherichia coli / Ampicillin-sulbactam	Quantitative	Human Health	Surveillance & Laboratory
% resistant (invasive isolates): Escherichia coli / Carbapenems	Quantitative	Human Health	Surveillance & Laboratory
% resistant (invasive isolates): Escherichia coli / Cephalosporins (3rd Gen)	Quantitative	Human Health	Surveillance & Laboratory
% resistant (invasive isolates): Escherichia coli / Fluoroquinolones	Quantitative	Human Health	Surveillance & Laboratory
% resistant (invasive isolates): Escherichia coli / Glycylcyclines	Quantitative	Human Health	Surveillance & Laboratory
% resistant (invasive isolates): Escherichia coli / Macrolides	Quantitative	Human Health	Surveillance & Laboratory
% resistant (invasive isolates): Escherichia coli / Piperacillin-tazobactam	Quantitative	Human Health	Surveillance & Laboratory
% resistant (invasive isolates): Escherichia coli / Polymyxins	Quantitative	Human Health	Surveillance & Laboratory
% resistant (invasive isolates): Escherichia coli / Trimethoprim-sulfamethoxazole	Quantitative	Human Health	Surveillance & Laboratory
% resistant (invasive isolates): Klebsiella pneumoniae / Aminoglycosides	Quantitative	Human Health	Surveillance & Laboratory
% resistant (invasive isolates): Klebsiella pneumoniae / Aminopenicillins	Quantitative	Human Health	Surveillance & Laboratory
% resistant (invasive isolates): Klebsiella pneumoniae / Amoxicillin-clavulanate	Quantitative	Human Health	Surveillance & Laboratory
% resistant (invasive isolates): Klebsiella pneumoniae / Carbapenems	Quantitative	Human Health	Surveillance & Laboratory
% resistant (invasive isolates): Klebsiella pneumoniae / Cephalosporins (3rd gen)	Quantitative	Human Health	Surveillance & Laboratory
% resistant (invasive isolates): Klebsiella pneumoniae / Fluoroquinolones	Quantitative	Human Health	Surveillance & Laboratory
% resistant (invasive isolates): Klebsiella pneumoniae / Glycylcyclines	Quantitative	Human Health	Surveillance & Laboratory
% resistant (invasive isolates): Klebsiella pneumoniae / Piperacillin-tazobactam	Quantitative	Human Health	Surveillance & Laboratory
% resistant (invasive isolates): Klebsiella pneumoniae / Polymyxins	Quantitative	Human Health	Surveillance & Laboratory
% resistant (invasive isolates): Klebsiella pneumoniae / Trimethoprim-sulfamethoxazole	Quantitative	Human Health	Surveillance & Laboratory
% resistant (invasive isolates): Pseudomonas	Quantitative	Human Health	Surveillance & Laboratory

aeruginosa / Amikacin			
% resistant (invasive isolates): Pseudomonas aeruginosa / Aminoglycosides	Quantitative	Human Health	Surveillance & Laboratory
% resistant (invasive isolates): Pseudomonas aeruginosa / Aminopenicillins	Quantitative	Human Health	Surveillance & Laboratory
% resistant (invasive isolates): Pseudomonas aeruginosa / Carbapenems	Quantitative	Human Health	Surveillance & Laboratory
% resistant (invasive isolates): Pseudomonas aeruginosa / Ceftazidime	Quantitative	Human Health	Surveillance & Laboratory
% resistant (invasive isolates): Pseudomonas aeruginosa / Cephalosporins (3rd gen)	Quantitative	Human Health	Surveillance & Laboratory
% resistant (invasive isolates): Pseudomonas aeruginosa / Fluoroquinolones	Quantitative	Human Health	Surveillance & Laboratory
% resistant (invasive isolates): Pseudomonas aeruginosa / Piperacillin-tazobactam	Quantitative	Human Health	Surveillance & Laboratory
% resistant (invasive isolates): Pseudomonas aeruginosa / Polymyxins	Quantitative	Human Health	Surveillance & Laboratory
% resistant (invasive isolates): Salmonella Paratyphi / Aminoglycosides	Quantitative	Human Health	Surveillance & Laboratory
% resistant (invasive isolates): Salmonella Paratyphi / Aminopenicillins	Quantitative	Human Health	Surveillance & Laboratory
% resistant (invasive isolates): Salmonella Paratyphi / Amoxicillin-clavulanate	Quantitative	Human Health	Surveillance & Laboratory
% resistant (invasive isolates): Salmonella Paratyphi / Carbapenems	Quantitative	Human Health	Surveillance & Laboratory
% resistant (invasive isolates): Salmonella Paratyphi / Cephalosporins (3rd gen)	Quantitative	Human Health	Surveillance & Laboratory
% resistant (invasive isolates): Salmonella Paratyphi / Fluoroquinolones	Quantitative	Human Health	Surveillance & Laboratory
% resistant (invasive isolates): Salmonella Paratyphi / Macrolides	Quantitative	Human Health	Surveillance & Laboratory
% resistant (invasive isolates): Salmonella Paratyphi / Piperacillin-tazobactam	Quantitative	Human Health	Surveillance & Laboratory
% resistant (invasive isolates): Salmonella Paratyphi / Polymyxins	Quantitative	Human Health	Surveillance & Laboratory
% resistant (invasive isolates): Salmonella Paratyphi / Trimethoprim-sulfamethoxazole	Quantitative	Human Health	Surveillance & Laboratory
% resistant (invasive isolates): Salmonella Typhi / Aminoglycosides	Quantitative	Human Health	Surveillance & Laboratory

	% resistant (invasive isolates): Salmonella Typhi / Aminopenicillins	Quantitative	Human Health	Surveillance & Laboratory
	% resistant (invasive isolates): Salmonella Typhi / Amoxicillin-clavulanate	Quantitative	Human Health	Surveillance & Laboratory
	% resistant (invasive isolates): Salmonella Typhi / Carbapenems	Quantitative	Human Health	Surveillance & Laboratory
	% resistant (invasive isolates): Salmonella Typhi / Cephalosporins (3rd gen)	Quantitative	Human Health	Surveillance & Laboratory
	% resistant (invasive isolates): Salmonella Typhi / Fluoroquinolones	Quantitative	Human Health	Surveillance & Laboratory
	% resistant (invasive isolates): Salmonella Typhi / Macrolides	Quantitative	Human Health	Surveillance & Laboratory
	% resistant (invasive isolates): Salmonella Typhi / Piperacillin-tazobactam	Quantitative	Human Health	Surveillance & Laboratory
	% resistant (invasive isolates): Salmonella Typhi / Polymyxins	Quantitative	Human Health	Surveillance & Laboratory
	% resistant (invasive isolates): Salmonella Typhi / Tetracyclines	Quantitative	Human Health	Surveillance & Laboratory
	% resistant (invasive isolates): Salmonella Typhi / Trimethoprim-sulfamethoxazole	Quantitative	Human Health	Surveillance & Laboratory
	% resistant (invasive isolates): Staphylococcus aureus / Aminoglycosides	Quantitative	Human Health	Surveillance & Laboratory
	% resistant (invasive isolates): Staphylococcus aureus / Aminopenicillins	Quantitative	Human Health	Surveillance & Laboratory
	% resistant (invasive isolates): Staphylococcus aureus / Amoxicillin-clavulanate	Quantitative	Human Health	Surveillance & Laboratory
	% resistant (invasive isolates): Staphylococcus aureus / Carbapenems	Quantitative	Human Health	Surveillance & Laboratory
	% resistant (invasive isolates): Staphylococcus aureus / Cephalosporins (3rd gen)	Quantitative	Human Health	Surveillance & Laboratory
	% resistant (invasive isolates): Staphylococcus aureus / Fluoroquinolones	Quantitative	Human Health	Surveillance & Laboratory
	% resistant (invasive isolates): Staphylococcus aureus / Glycylcyclines	Quantitative	Human Health	Surveillance & Laboratory
	% resistant (invasive isolates): Staphylococcus aureus / Linezolid	Quantitative	Human Health	Surveillance & Laboratory
	% resistant (invasive isolates): Staphylococcus aureus / Macrolides	Quantitative	Human Health	Surveillance & Laboratory
	% resistant (invasive isolates): Staphylococcus	Quantitative	Human Health	Surveillance & Laboratory

		aureus / Oxacillin (MRSA)			
		% resistant (invasive isolates): Staphylococcus aureus / Piperacillin-tazobactam	Quantitative	Human Health	Surveillance & Laboratory
		% resistant (invasive isolates): Staphylococcus aureus / Rifampicin	Quantitative	Human Health	Surveillance & Laboratory
		% resistant (invasive isolates): Staphylococcus aureus / Trimethoprim-sulfamethoxazole	Quantitative	Human Health	Surveillance & Laboratory
		% resistant (invasive isolates): Staphylococcus aureus / Vancomycin	Quantitative	Human Health	Surveillance & Laboratory
		% resistant (invasive isolates): Streptococcus pneumoniae / Aminoglycosides	Quantitative	Human Health	Surveillance & Laboratory
		% resistant (invasive isolates): Streptococcus pneumoniae / Cephalosporins (3rd gen)	Quantitative	Human Health	Surveillance & Laboratory
		% resistant (invasive isolates): Streptococcus pneumoniae / Fluoroquinolones	Quantitative	Human Health	Surveillance & Laboratory
		% resistant (invasive isolates): Streptococcus pneumoniae / Linezolid	Quantitative	Human Health	Surveillance & Laboratory
		% resistant (invasive isolates): Streptococcus pneumoniae / Macrolides	Quantitative	Human Health	Surveillance & Laboratory
		% resistant (invasive isolates): Streptococcus pneumoniae / Penicillins	Quantitative	Human Health	Surveillance & Laboratory
		% resistant (invasive isolates): Streptococcus pneumoniae / Tetracyclines	Quantitative	Human Health	Surveillance & Laboratory
		% resistant (invasive isolates): Streptococcus pneumoniae / Trimethoprim-sulfamethoxazole	Quantitative	Human Health	Surveillance & Laboratory
		% resistant (invasive isolates): Streptococcus pneumoniae / Vancomycin	Quantitative	Human Health	Community Awareness & Enabling Behaviors
17	InFARM surveillance components and implementation questionnaire	Is there a national AMR surveillance strategy/programme/plan integrating components and implementation of activities across different AMR surveillance programmes in animals and food (e.g. surveillance of healthy animals and food at processing and/or point of sale is conducted in an integrated manner at various steps of the food chain, including primary production, processing, and distribution)? (Yes, no, don't know)	Qualitative	Animal Health	Surveillance & Laboratory
		What is the status of development and implementation of this integrated strategy/programme/plan? (under development,	Qualitative	Animal Health	Surveillance & Laboratory

	finalized but waiting for final endorsement, approved by government but no funds allocated, approved by government with allocated funds, and under implementation, don't know)			
	Which specific surveillance programmes under the InFARM framework are covered by this integrated strategy/programme/plan? Multiple answers are possible (healthy terrestrial animals (potentially expanded to cover their production environment) / diseased terrestrial animals / healthy aquatic animals (potentially expanded to cover their production environment) / diseased aquatic animals / food at processing and/or point of sale)	Qualitative	Animal Health	Surveillance & Laboratory
	What are the requirements for peripheral laboratories to participate in the integrated national surveillance network in animals and food? Multiple answers are possible. (Participation in EQA/PT for bacterial isolation and/or AST / Shipment of bacterial isolates for confirmation or additional characterization / Commitment on data quality of results (methods, format for data sharing) / Implementation of quality assurance and/or accreditation of the laboratory/ No specific requirements, all interested laboratories can participate)	Qualitative	Animal Health	Surveillance & Laboratory
	Is surveillance and monitoring of antimicrobial use (AMU) in agri-food systems, and/or AMR/AMU surveillance and monitoring in other sectors (human and/or environment) also covered by this integrated strategy/programme/plan? Multiple answers are possible (Yes, integrated with surveillance and monitoring of AMU in terrestrial animals / Yes, integrated with surveillance and monitoring of AMU in aquatic animals / Yes, integrated with surveillance and monitoring of AMU in plant production and protection / Yes, integrated with surveillance and monitoring of AMU in humans / Yes, integrated with surveillance and monitoring of AMR in humans / Yes,	Qualitative	Animal Health	Surveillance & Laboratory

	integrated with surveillance and monitoring of AMR in the wider environment / Yes, integrated with surveillance and monitoring of AMR/AMU in others, please specify / No / Don't know			
	Is there any specific and independent national AMR surveillance strategy/programme/plan in any of the AMR surveillance programmes under the InFARM framework (i.e. components and implementation of surveillance in at least one of the programmes is compartmentalized and not integrated with the rest of programmes)? Select only one answer (Yes / no / don't know)	Qualitative	Animal Health	Surveillance & Laboratory
	Which specific surveillance programmes under the InFARM framework are compartmentalized and not integrated under a specific and independent national AMR surveillance strategy/programme/plan? Multiple answers are possible. (Healthy terrestrial animals (potentially expanded to cover their production environment) / diseased terrestrial animals / healthy aquatic animals (potentially expanded to cover their production environment) / diseased aquatic animals / food at processing and/or point of sale)	Qualitative	Animal Health	Surveillance & Laboratory
	Are there any additional AMR surveillance activities conducted in any of the specific surveillance programmes under the InFARM framework but without a strategy/programme/plan? Select only one answer (yes / no / don't know)	Qualitative	Animal Health	Surveillance & Laboratory
	Which specific surveillance programmes under the InFARM framework conducted AMR surveillance activities without a strategy/programme/plan? Multiple answers are possible. (Healthy terrestrial animals (potentially expanded to cover their production environment) / diseased terrestrial animals / healthy aquatic animals (potentially expanded to cover their production environment / diseased aquatic animals / food at processing and/or point of sale)	Qualitative	Animal Health	Surveillance & Laboratory

	Has a national network of peripheral laboratories supporting integrated AMR surveillance in animals and food been established?3 Select only one answer (yes / no / don't know)	Qualitative	Animal Health	Surveillance & Laboratory
	Are these peripheral laboratories supporting the integration of components and implementation of activities across different AMR surveillance programmes under the InFARM framework (e.g. laboratories receiving samples from healthy animals and food taken at various steps of the food chain, including primary production, processing, and distribution)? Select only one answer (yes / no / don't know)	Qualitative	Animal Health	Surveillance & Laboratory
	Please provide the number of laboratories included in the network	Quantitative	Animal Health	Surveillance & Laboratory
	What are the responsibilities of the peripheral laboratories supporting integrated AMR surveillance in animals and food? Multiple answers are possible. (Submission of bacterial isolates to the National Reference Laboratory for antimicrobial susceptibility testing / Submission of bacterial isolates to NRL for further molecular characterization / Perform AST and submission of bacterial isolates to NRL for confirmation of results / Submission of written verification of performance and quality control procedures to NRL / Provision of details of their performance in external quality assurance or proficiency testing (EQA/PT) schemes for bacterial isolation and/or AST / other, please specify)	Qualitative	Animal Health	Surveillance & Laboratory
	Has a National Reference Laboratory (NRL) been designated to support integrated AMR surveillance in animals and food? Select only one answer (Yes / no / don't know)	Qualitative	Animal Health	Coordination / Surveillance & Laboratory
	Is the NRL supporting the integration across different AMR surveillance programmes in animals and food (e.g. supporting peripheral laboratories receiving samples from healthy animals and food taken at various steps of the food	Qualitative	Animal Health	Surveillance & Laboratory

	chain, including primary production, processing, and distribution)? Select only one answer (yes / no / don't know)			
	Please indicate the responsibilities for the national reference laboratory (NRL) supporting integrated AMR surveillance in animals and food. Multiple answers are possible (coordinate the national AMR laboratory network including peripheral laboratories / confirm results from peripheral laboratories (e.g. specific resistance phenotypes, molecular characterization) / validate appropriate methods for defined surveillance programmes / provide guidance and reference material (e.g. protocols, reference strains) / provide external quality assurance/proficiency testing for antimicrobial susceptibility testing to national AMR laboratory network / collaborate and provide support to the NCC / collaborate and provide support to the designated unit/person to analyse AMR surveillance data / provide advice and expertise to risk managers and decision/policy makers / develop research programmes and international scientific collaborations on AMR / other)	Qualitative	Animal Health	Feedback Mechanisms / Surveillance & Laboratory
	Does the NRL supporting integrated AMR surveillance in animals and food participate in External Quality Assurance (EQA)/Proficiency Testing (PT) scheme for Antimicrobial Susceptibility Testing (AST)? (yes / no / don't know)	Qualitative	Animal Health	Feedback Mechanisms
	How often in months is the frequency of EQA/PT	Quantitative	Animal Health	Feedback Mechanisms
	What are the microorganisms assessed? (approx. 50 different to select from)	Qualitative	Animal Health	Feedback Mechanisms
	Are corrective measures taken after EQA/PT? (yes / no / don't know)	Qualitative	Animal Health	Feedback Mechanisms
	Is there any institutional body that substitutes the function or having equal function as NRL supporting integrated AMR surveillance in animals	Qualitative	Animal Health	Surveillance & Laboratory

		and food? (yes no)			
		Has a national coordinating centre (NCC) or a centralized surveillance team overseeing integrated AMR surveillance and/or monitoring in animals and food been established? Select only one answer (yes / no / don't know)	Qualitative	Animal Health	Surveillance & Laboratory
		Is the NCC supporting the integration of components and implementation of surveillance activities across different AMR surveillance programmes under the InFARM framework (e.g. surveillance of healthy animals and food at processing and/or point of sale is conducted in an integrated manner at various steps of the food chain, including primary production, processing, and distribution) Select only one answer (yes / no / don't know)	Qualitative	Animal Health	Surveillance & Laboratory
		What microorganisms are included in your country for integrated AMR surveillance in animals and food? Multiple answers are possible? (many different listed microorganisms)	Qualitative	Animal Health	Surveillance & Laboratory
		Have specific list(s) or panels of antibiotics been defined to be tested based on bacterial genus/species for integrated AMR surveillance in animals and food? (yes / no / don't know)	Qualitative	Animal Health	Surveillance & Laboratory
		List the classes of antibiotics represented by the antibiotic panel(s) (many different antibiotics listed to select)	Qualitative	Animal Health	Surveillance & Laboratory
18	CAESAR External Quality Assessment	Methods and guidelines used by participating laboratories examining the EQA specimens (CLSI, EUCAST)	Quantitative	Human Health	Surveillance & Laboratory
		Number of laboratories and type of susceptibility testing method per country or area (Disk diffusion (CLSI) / Semi-automated system (CLSI) / Disk diffusion (EUCAST) / Semi-automated system (EUCAST))	Quantitative	Human Health	Surveillance & Laboratory
		Correct identification % among participating laboratories (disaggregated by 6 different organisms)	Quantitative	Human Health	Surveillance & Laboratory
		Percentage of laboratories giving the correct result:	Quantitative	Human Health	Surveillance & Laboratory

		identification (for 6 different organisms)			
		Percentage of laboratories giving the correct result: E. Coli (for 17 different antimicrobial agents)	Quantitative	Human Health	Surveillance & Laboratory
		Percentage of laboratories giving the correct result: S. enterica (for 17 different antimicrobial agents)	Quantitative	Human Health	Surveillance & Laboratory
		Percentage of laboratories giving the correct result: A. baumannii complex (for 8 different antimicrobial agents)	Quantitative	Human Health	Surveillance & Laboratory
		Percentage of laboratories giving the correct result: S. aureus (for 13 different antimicrobial agents)	Quantitative	Human Health	Surveillance & Laboratory
		Percentage of laboratories giving the correct result: S. pneumoniae (for 14 different antimicrobial agents)	Quantitative	Human Health	Surveillance & Laboratory
		Percentage of laboratories giving the correct result: E. faecalis (for 5 different antimicrobial agents)	Quantitative	Human Health	Coordination
19	WHO implementation handbook for NAPs on AMR: guidance for the human health sector (annex 7)	Establish or improve national/subnational coordination and governance: fully functional decision-making body with operational working groups established	Qualitative	Multisectoral	Feedback Mechanisms / Transparency
		Establish or improve national/subnational coordination and governance: Annual reports from all sectors on NAP progress shared with the multisectoral governance mechanism for decision-making and revision	Qualitative	Multisectoral	Feedback Mechanisms / Coordination
		Establish or improve national/subnational coordination and governance: Budget lines for different activities in all sectors finalized and agreed	Qualitative	Multisectoral	Sustainability
		Establish or improve national/subnational coordination and governance: TORs for national focal points, multisectoral coordination mechanism(s) and TWGs(s) written and approved	Qualitative	Multisectoral	Strategic Vision
		Establish or improve national/subnational coordination and governance: independent M&E working group on NAP implementation established	Qualitative	Multisectoral	Strategic Vision
		Establish or improve national/subnational coordination and governance: national focal points for all sectors nominated	Qualitative	Multisectoral	Strategic Vision

Is there regular audit, review and dissemination of AMR treatment guidelines? Is the audit report published?	Y/N	Multisectoral	Feedback Mechanisms / Transparency
GAP objective 1: improve awareness and understanding of AMR through effective communication, education and training: WAAW activities conducted	Qualitative	Multisectoral	Reporting
GAP objective 2: strengthen the knowledge and evidence base through surveillance and research: data on AMR and AMU in humans - reporting to GLASS on AMR in humans and AMU in humans	Qualitative	Human Health	Surveillance & Laboratory
GAP objective 3: reduce the incidence of infection through effective sanitation and hygiene and infection prevention measures: Overall vaccination coverage of >95% in human population achieved	Qualitative	Human Health	Stewardship
GAP objective 4: optimize the use of antimicrobial medicines in human health: a national plan on policy on AMS or optimization of AMU in the human sector in place	Qualitative	Human Health	Stewardship
GAP objective 4: optimize the use of antimicrobial medicines in human health: sales and import data to estimate the consumption of antibiotics in human health collected and analysed	Qualitative	Human Health	Stewardship
GAP objective 4: optimize the use of antimicrobial medicines in human health: AWaRE classification included in the national EML	Qualitative	Human Health	Stewardship /Feedback Mechanisms
GAP objective 4: optimize the use of antimicrobial medicines in human health: Regular audit, review and dissemination of treatment guidelines and audit report published	Qualitative	Human Health	Stewardship
GAP objective 4: optimize the use of antimicrobial medicines in human health: pilot point prevalence surveys on AMU in selected health care facilities conducted	Qualitative	Human Health	Stewardship
GAP objective 4: optimize the use of antimicrobial medicines in human health: a national/subnational AMS TWG and AMS committees in health care facilities established	Qualitative	Human Health	Regulations & Legislation
GAP objective 4: optimize the use of antimicrobial	Qualitative	Human Health	Regulations & Legislation

	medicines in human health: legislative framework to limit or prohibit over-the-counter sale of antibiotics without a prescription developed and implemented			
	GAP objective 4: optimize the use of antimicrobial medicines in human health: legislative framework for regulation of antibiotic and active pharmaceutical ingredient production developed and implemented	Qualitative	Human Health	Feedback Mechanisms
	GAP objective 4: optimize the use of antimicrobial medicines in human health: assessment of adherence legislation on strict rules of sales of antimicrobials to animals (and use the WHO list of critically important antimicrobials assessed	Qualitative	Animal Health	Stewardship
	GAP objective 4: optimize the use of antimicrobial medicines in human health: adoption of AWaRe classification of antibiotics in the national EML and AMS strategies	Qualitative	Human Health	Stewardship
	GAP objective 4: optimize the use of antimicrobial medicines in human health: Guidelines on optimizing antibiotic use are implemented for all major syndromes and data on use is systematically fed back to the prescriber	Qualitative	Human Health	Stewardship
	GAP objective 4: optimize the use of antimicrobial medicines in human health: use of antimicrobials in humans - total human consumption of antibiotics for systemic use (anatomical therapeutic chemical classification code J01) in Defined Daily Doses (DDDs) per 1000 population (or inhabitants) per day	Quantitative	Human Health	Stewardship
	GAP objective 4: optimize the use of antimicrobial medicines in human health: appropriate use of antimicrobials - percentage of inpatient surgical procedures with appropriate timing and duration of surgical antibiotic prophylaxis	Quantitative	Human Health	Stewardship
	GAP objective 4: optimize the use of antimicrobial medicines in human health: use of antimicrobials in humans - proportion of Access antibiotics for systemic use, relative to total antibiotic	Quantitative	Human Health	Stewardship

		consumption in DDDs			
		GAP objective 4: optimize the use of antimicrobial medicines in human health: Relative proportion of AWaRe antibiotics for paediatric formulations	Quantitative	Human Health	Stewardship
		GAP objective 4: optimize the use of antimicrobial medicines in human health: use of antimicrobials in humans - percentage of adult and paediatric hospital patients receiving an antibiotic according to AWaRe categories	Quantitative	Human Health	Stewardship
		GAP objective 4: optimize the use of antimicrobial medicines in human health: access of antibiotics - percentage of health facilities that have a core set of relevant antibiotics available and affordable on a sustainable basis	Quantitative	Human Health	Regulations & Legislation
		GAP objective 4: optimize the use of antimicrobial medicines in human health: optimized AMU and regulation - legislation or regulation that requires antimicrobials for human use to be dispensed only with a prescription from an authorized health worker	Qualitative	Human Health	Regulations & Legislation
20	Methodology to analyze AMR-relevant legislation in the food and agriculture sector	Regulate the authorization of Veterinary medical products (VMP) and, to this purpose, establish and implement efficient statutory registration procedures that evaluate the quality, safety and efficacy of VMP	Target	Animal Health	Research, Innovation, & Digital Technology
		Grant marketing authorization on the basis of the data submitted by the pharmaceutical industry or applicant and only if the criteria of safety, quality and efficacy are met. Within the framework above, if new VMPs are necessary for specific needs related to the treatment of animal diseases, expedite the authorization processes.	Target	Animal Health	Research, Innovation, & Digital Technology
		Countries without the resources to implement an authorization procedure and whose supply of VMPs mostly depend on imports from foreign countries, should: a. ensure the efficacy of administrative controls on importation of VMPs; b. seek and validate information on the status of authorizations valid in other countries; c. develop	Target	Multisectoral	Coordination / Regulations & Legislation

		technical cooperation with experienced authorities to check the quality of imported VMPs as well as the validity of the recommended conditions of use.			
		Is there any regulatory instrument setting up a coordination mechanism across ministries and other entities for AMR governance? (horizontal level)	Y/N	Multisectoral	Coordination/ Participation / Transparency
		How are the composition, mandate and decision-making powers of the AMR coordination mechanism defined? Is there a reference to its funding? Does this mechanism include also representatives from the decentralized level and the private sector? If it includes private sector representatives: is there any safeguard against potential conflicts of interest?	Qualitative	Multisectoral	Coordination
		How are national competences shared between the central and the decentralized level for those areas relevant for AMR (health, agriculture, environment…)?	Qualitative	Multisectoral	Community Awareness & Enabling Behaviors
21	People-centred approach to addressing antimicrobial resistance in human health: WHO core package of interventions to support national action plans	Develop and implement a communication strategy to improve awareness and understanding of AMR among policy-makers, health workers and communities.	Target	Multisectoral	Feedback Mechanisms
		Use the collected data to inform action and guidelines, and monitor the burden and distribution of AMR	Target	Human Health	Feedback Mechanisms
		Collect, collate, analyse and interpret data on infections (syndromic and AMR) as part of AMR/HAI surveillance to guide local empirical treatment.	Target	Human Health	Surveillance & Laboratory
		Conduct routine surveillance of AMC at national and subnational levels.	Target	Multisectoral	Stewardship
		Raise political awareness by advocating for AMR, and secure political and financial commitment, and accountability from the government.	Target	Multisectoral	Strategic Vision
		Ensure that AMC/U data collected at all levels are analysed, reported and shared with relevant stakeholders to signal possible under- and overuse, and inform corrective actions.	Target	Multisectoral	Research, Innovation, & Digital Technology

Develop a national AMR research agenda adapted from global and regional AMR agendas according to local research priorities, including behavioural and implementation research.	Target	Multisectoral	Research, Innovation, & Digital Technology
Build capacity for implementation of AMR research priorities in collaboration with academia, the private sector and civil society.	Target	Multisectoral	Research, Innovation, & Digital Technology
Participate in clinical trial networks and surveillance platforms, share AMR and AMC/U data, and foster public and private collaboration to ensure that vaccine, diagnostic and antimicrobial development pipelines meet unmet public health needs.	Target	Multisectoral	Research, Innovation, & Digital Technology
Identify sustainable financing and incentives and strengthen local capacity for the development and/or production of novel and/or existing vaccines, diagnostics, and antimicrobials including appropriate paediatric and oral formulations that target priority pathogens.	Target	Multisectoral	Access to Medicines & Health Services
Ensure equitable access to and timely national and local adoption of new and existing vaccines, diagnostics and antimicrobials, including ensuring timely inclusion of products into policies, guidelines, procurement and reimbursement schemes.	Target	Human Health	Access to Medicines & Health Services
Ensure that access to diagnosis, treatment and care of (drug-resistant) infections is not barred by financial constraints by including the services in pooled financing schemes, such as UHC health benefit packages, while ensuring rational use.	Target	Human Health	Access to Medicines & Health Services
Ensure the affordability of quality-assured essential vaccines, diagnostics and antimicrobials for all including key vulnerable groups through appropriate financing and pricing policies.	Target	Human Health	Community Awareness & Enabling Behaviors
Improve public awareness of the coverage of health services and products related to the prevention, diagnosis and treatment of (drug-resistant) infections in health benefits packages.	Target	Human Health	Access to Medicines & Health Services
Ensure the availability and affordability of	Target	Human Health	Access to Medicines & Health

	preventive testing and counselling services for common infections (e.g. sexually transmitted infections), with engagement of the community.			Services
	Ensure adequate forecasting, procurement and distribution of essential diagnostics, reagents, antibiotics, and other health products for management of (drug-resistant) infections	Target	Human Health	Regulations & Legislation
	Implement policies and procedures to prevent, detect and respond to substandard and falsified medical products (vaccines, diagnostics and antibiotics) to manage (drug-resistant) infections at all levels of health care	Target	Human Health	Community Awareness & Enabling Behaviors
	Increase community engagement in the prevention, detection and reporting of supply issues of medical products, including substandard and falsified medical products, and enhance community awareness on their impact on AMR.	Target	Multisectoral	Surveillance & Laboratory
	Establish or strengthen a national quality-assured bacteriology (and mycology) laboratory system, including a national bacteriology reference laboratory which supports capacity building at all levels and external quality control.	Target	Multisectoral	Workforce
	Develop national guidelines for diagnostic stewardship and integrate them into pre- and in-service training for health workers at all levels of health care.	Target	Human Health	Stewardship
	Develop or revise and implement up-to-date national treatment guidelines based on AMS principles, the WHO AWaRe antibiotic book, evidence and epidemiology, and monitor compliance.	Target	Human Health	Stewardship
	Integrate the AWaRe classification into the national essential medicines list and formulary to promote appropriate selection and use of antimicrobials.	Target	Human Health	Regulations & Legislation
	Develop, implement and enforce legislation and regulation to restrict (OTC) sales of antimicrobials (including internet sales) to prescriptions from a qualified health-care professional.	Target	Human Health	Stewardship
	Restrict the inappropriate promotion and	Target	Human Health	Stewardship

		advertisement of antimicrobials, and design local solutions to increase accountability in sales of antimicrobials.			
		Antibiotic residue in wastewater shall be quantified and assessed against environmental protection criteria to measure risk. Risk shall be measured by the risk quotient (RQ), which is a comparison of the predicted environmental concentration (PEC) of an API in the environment resulting from a site's wastewater discharge to the predicted no-effect concentration (PNEC).	Target	Environment	Stewardship
		The user shall reduce the antibiotics discharged to the environment to an RQ less than 1 (RQ <1) by employing good management practices and by applying a hierarchy of control.	Target	Environment	Stewardship
		Ensure active participation of the human health sector in the national multisectoral AMR coordination mechanism.	Target	Multisectoral	Participation
		Engage civil society and communities in the multisectoral AMR coordination mechanism and in development, implementation and monitoring of the One Health NAP on AMR.		Multisectoral	Participation
		Develop or adapt and implement national integrated AMS policy, standards and tools, and provide education materials and in- and pre-service training for health workers.		Multisectoral	Stewardship
22	Priorities of the Global Leaders Group on AMR	The user shall assess wastewater discharges containing antibiotic to determine the concentration of antibiotic(s). This predicted environmental concentration (PEC) shall be less than the concentration believed to result in increased selection pressure on bacteria in the environment, known as the predicted no-effect concentration (PNEC). Where necessary the user shall apply controls or treatment to achieve the PNEC, where: PEC / PNEC = Risk Quotient (RQ) RQ<1	Target	Environment	Surveillance & Laboratory / Prevention & Control
		Environmental AMR containment efforts are included in national action plans on AMR in all countries.	Target	Environment	Regulations & Legislation / Prevention & Control
23	Point prevalence survey of	Antimicrobial stewardship elements present in the	Percent of countries	Human Health	Stewardship

healthcare-associated infections and antimicrobial use in European long-term care facilities	included LTCFs, by country	with: Antimicrobial committee, training on appropriate prescribing, written guidelines for antimicrobial use, data on annual antimicrobial consumption, reminder of importance samples, local antimicrobial resistance profiles, permission for prescribing restricted antimicrobials, advice from a pharmacist, therapeutic formulary, feedback to GPs on antimicrobial consumption, None of these elements		
	Available written therapeutic antimicrobial guidelines and surveillance programmes in the included LTCFs, by country	Percent of Countries with RTI, UTI, Wound and SSTI	Human Health	Surveillance & Laboratory
	Available written surveillance programmes in the included LTCFs, by country	Percent of Countries with HAIs, Antimicrobial Consumption, Resistant Microorganisms	Human Health	Reporting
	Age and gender of the LTCF residents presenting an HAI and of the LTCF residents receiving an antimicrobial, by country, HALT-2, 2013	Quantitative	Human Health	Reporting
	Length of stay, recent hospitalisation, care load indicators and risk factors among LTCF residents with an HAI and among LTCF residents receiving an antimicrobial, by country,	Quantitative	Human Health	Prevention & Control
	Number and prevalence of LTCF residents with at least one HAI on the day of survey, by country,	Quantitative	Human Health	Prevention & Control

	Distribution of types of HAI (number and relative frequency) in the included LTCFs, by country,	Quantitative: UTIs (Probable, confirmed); RTIs (Colds, flu, pna, other lower RTIs); SSTI; Herpes Simplex or Zoster infections; GI infections (Gastroenteritis, CDI); ENT (Conjunctivitis, ear infections, sinusitis, oral candidiasis)	Human Health	Stewardship
	Number and prevalence of eligible LTCF residents receiving at least one antimicrobial agent on the day of the PPS, by country,	Quantitative	Human Health	Stewardship
	Indication for antimicrobial use, by country,	Percent prophylactic vs therapeutic	Human Health	Stewardship
	Site of diagnosis for antimicrobial use, by country,	Percent Respiratory tract, urinary tract, skin, GI, ENT, systemic, Unexplained fever, other	Human Health	Stewardship
	Distribution of use of antibacterials for systemic use	Beta-lactams, PNC; Other; Quinolones; Other beta-lactams; Sulfonamides & Trimethoprim; Macrolides, streptogramins, lincosamides; Tetracyclines, Other J01 classes	Human Health	Stewardship
	Number of courses of antimicrobials for treatment or prophylaxis, with a microbiological sample taken and with culture results in the included LTCFs, by country,	Quantitative	Human Health	Surveillance & Laboratory
	Antimicrobial resistance markers in selected microorganisms,	Quantitative: Staph aureus (Oxacillin S/R); Enterococcus	Human Health	Stewardship

			(Glycopeptide S/NS); Enterobacteriaceae (3rd gen Cephalosporin and Carbapenem S/S OR NS/s OR NS/NS); Pseudomonas aeruginosa (Carbapenem S/NS); Acinetobacter baumannii (Carbapenem S/NS)		
24	Muscat Ministerial Manifesto on AMR	Target 1: Reduce the total amount of antimicrobials used in the agri-food system at least by 30-50% by 2030 from the current level	Target	Environment	Stewardship
		Target 2: Zero Use of medically important antimicrobials for human medicine in animals for non-veterinary purposes or in crop production and agri-food systems for non-phytosanitary purposes	Target	Animal Health	Stewardship
		Target 3: Ensuring that ACCESS group antibiotics comprise at least 60% of overall antibiotic consumption in humans by 2030	Target	Human Health	Community Awareness & Enabling Behaviors
25	Antimicrobial resistance: global report on surveillance	Total no. of reports with data sets based on 30+ isolates by WHO region for: (E. coli / 3rd gen cephalosporins; E. coli / fluoroquinolones; K. pneumoniae / 3rd gen cephalosporins; K. pneumoniae / carbapenems; MRSA; S. pneumoniae non-susceptible or resistant to penicillin; nontyphoidal salmonella / fluoroquinolones; shigella species / fluoroquinolones; N. gonorrhoeae / 3rd gen cephalosporins; total no. of reports with data sets based on 30+ tested isolates)	Quantitative	Human Health	Surveillance & Laboratory
		Fluconazole drug resistance, by Candida, species and country	Quantitative	Human Health	Sustainability
26	From reacting to preventing pandemics: building animal health and wildlife systems for One Health in East Asia	Institutional capacities for managing EIDs linked to livestock, zoonoses, and food-borne infections	Quantitative	Animal Health	Feedback Mechanisms
		Assessment of animal health, food safety, and AMR programs	Scale 0-4	Animal Health	Feedback Mechanisms

	and Pacific	Assessment of country performance capacities: governance, capacity, budgets, livestock, wildlife (disaggregate by income level)	Scale 0-5	Multisectoral	Sustainability
		Operating budget for veterinary services (disaggregate by income level)	Quantitative	Animal Health	Sustainability
		Available annual budgets for livestock and animal health services	Quantitative	Animal Health	Stewardship
27	Antimicrobial consumption and resistance in bacteria from humans and food-producing animals	Population-weighted mean of the total consumption of animals in humans	Quantitative	Animal Health	Stewardship
		Population-weighted mean of the total consumption of animals in food producing animals	Quantitative	Animal Health	Surveillance & Laboratory
		Clinical breakpoints for I "susceptible, increased exposure" and R "resistant" categories combined and epidemiological cut-off values used to interpret MIC data reported for E. Coli from humans and food-producing animals (Ampicillin and Amoxicillin, ceftriaxone, cefotaxime, ceftazidime, ciprofloxacin, ofloxacin, levofloxacin, imipenem, meropenem) in Mg/L	Quantitative	Human Health	Surveillance & Laboratory
		Clinical breakpoints for I "susceptible, increased exposure" and R "resistant" categories combined and epidemiological cut-off values used to interpret MIC data reported for Salmonella from humans and food-producing animals (Ampicillin, Cefotaxime, Ciprofloxacin, Nalidixic acid, tetracyclines) in mg/L	Quantitative	Human Health	Surveillance & Laboratory
		Clinical breakpoints for I "susceptible, increased exposure" and R "resistant" categories combined and epidemiological cut-off values used to interpret MIC data reported for Campylobacter spp. from humans and food-producing animals (ciprofloxacin, erythromycin, tetracyclines) in mg/L	Quantitative	Human Health	Stewardship
		Amount of active antimicrobial substance (tonnes) in humans, food producing animals, and total	Quantitative	Multisectoral	Stewardship
		Estimated biomass (1000 tonnes) of humans and food-producing animals	Quantitative	Multisectoral	Stewardship
		Antimicrobial consumption (mg/kg estimated biomass) in humans and food-producing animals	Quantitative	Multisectoral	Stewardship

		Antimicrobial consumption (mg/kg estimated biomass) in humans and food-producing animals, disaggregated by antimicrobial group (3rd and 5th gen cephalosporins, fluoroquinolones and other quinolones, polymyxins, aminopenicillins, macrolides, tetracyclines)	Quantitative	Multisectoral	Surveillance & Laboratory
28	Averting the AMR Crisis: What are the Avenues for policy action for countries in Europe?	Estimates of the burden of infections with selected antibiotic-resistant bacteria of public health importance in DALYs per 100 000 population, EU/EAA\	Qualitative (categorical)	Human Health	Surveillance & Laboratory
		% resistant (invasive isolates) of K. pneumoniae and E. coli	Quantitative	Human Health	Stewardship
		Consumption of antibiotics for systemic use (ATC group J01) in EU/EAA countries, in DDDs per 1000 inhabitants per day (in community and in the hospital sector)	Quantitative	Human Health	Stewardship
29	How can the EU support sustainable innovation and access to effective antibiotics?	Number of antibiotics available	Quantitative	Multisectoral	Access to Medicines & Healthcare Services
		Globally exported API volumes for antibiotics, in kilotonnes	Quantitative	Multisectoral	Stewardship
30	Third joint inter-agency report on integrated analysis of consumption of antimicrobial agents and occurrence of antimicrobial resistance in bacteria from humans and food-producing animals in the EU/EEA (with disaggregation by antimicrobial class)	Amount of antimicrobial active substance, estimated biomass and antimicrobial consumption in humans and food producing animals	Quantitative	Multisectoral	Stewardship
		Consumption of carbapenems in humans expressed as DDD per 1000 inhabitants per day, by country	Quantitative	Human Health	Stewardship
		Biomass-corrected consumption of 3rd and 4th gen cephalosporins in humans and food-producing animals	Quantitative	Human Health	Stewardship
		Consumption of 3rd and 4th generation cephalosporins in humans, in DDD per 1000 inhabitants per day	Quantitative	Human Health	Stewardship
		Consumption of 3rd and 4th generation cephalosporins in food-producing animals, in mg per kg of estimated biomass	Quantitative	Animal Health	Stewardship
		Population-corrected consumption of fluoroquinolones and other quinolones in humans and food-producing animals	Quantitative	Human Health	Surveillance & Laboratory
		Consumption of fluoroquinolones and other quinolones in humans, expressed as DDD per 1000	Quantitative	Human Health	Stewardship

		inhabitants per day			
		Population-corrected consumption of polymyxins in humans and food-producing animals	Quantitative	Human Health	Stewardship
		Biomass-corrected consumption of aminopenicillins in humans and food-producing animals	Quantitative	Human Health	Stewardship
		Population-corrected consumption of macrolides for humans and food producing animals	Quantitative	Human Health	Surveillance & Laboratory
		Population-corrected consumption of tetracyclines for humans and food-producing animals	Quantitative	Human Health	Stewardship
		AMC humans	Quantitative	Human Health	Surveillance & Laboratory
		AMC animals	Quantitative	Animal Health	Surveillance & Laboratory
		%3GCR EC Humans	Quantitative	Human Health	Surveillance & Laboratory
		% Complete S EC animals	Quantitative	Animal Health	Surveillance & Laboratory
		% MRSA humans	Quantitative	Human Health	Effectiveness
31	Building the investment case for action against antimicrobial resistance (Annex to the GLG report)	Loss of life expectancy attributable to AMR	Quantitative	Human Health	Effectiveness
		Excess health expenditure due to AMR	Quantitative	Human Health	Effectiveness
		Depressed economic activity through reduced participation in the workforce from AMR	Quantitative	Multisectoral	Coordination
32	FAO-ATLASS "SET AMR" (Surveillance Evaluation Tool for AMR)	Governance: existence of an operational structure representative of the stakeholders involved in AMR surveillance under One Health approach (multi-sectoral working group(s) or coordination committee on AMR)	Semi-quantitative (1-5 scale)	Multisectoral	Strategic Vision
		Governance: Development of a National Action Plan on AMR involving the food and agriculture sectors	Semi-quantitative (1-5 scale)	Multisectoral	Surveillance & Laboratory
		Governance: Relevance of AMR surveillance objectives and AMR indicators in food and agriculture sectors	Semi-quantitative (1-5 scale)	Environment	Regulations & Legislation
		Governance: regulations on AMR surveillance organization in the food and agriculture sectors	Semi-quantitative (1-5 scale)	Environment	Surveillance & Laboratory
		Data collection and analysis: Existence of an operational management structure (central epidemiology unit) in food and agriculture sectors	Semi-quantitative (1-5 scale)	Environment	Surveillance & Laboratory
		Data collection and analysis: Representativeness of	Semi-quantitative (1-5	Environment	Workforce

	the surveillance sampling scheme in food and agriculture sectors including environment	scale)		
	Data collection and analysis: Adequate skill level in AMR epidemiology of members of the central unit	Semi-quantitative (1-5 scale)	Multisectoral	Surveillance & Laboratory
	Data collection and analysis: Data input interval in accordance with the objectives and use of AMR surveillance system results	Semi-quantitative (1-5 scale)	Multisectoral	Surveillance & Laboratory
	Data collection and analysis: AMR data verification and validation procedures formalized and operational	Semi-quantitative (1-5 scale)	Multisectoral	Surveillance & Laboratory
	Data collection and analysis: Analysis of AMR data against system requirements	Semi-quantitative (1-5 scale)	Multisectoral	Surveillance & Laboratory
	Data collection and analysis: Adequacy of the data management system for the needs of the AMR surveillance system results	Semi-quantitative (1-5 scale)	Multisectoral	Surveillance & Laboratory
	Data production network: Effective integration of component laboratories in the AMR surveillance system	Semi-quantitative (1-5 scale)	Multisectoral	Surveillance & Laboratory
	Data production network: Level of the standardization of work between different laboratories involved in the AMR surveillance system	Semi-quantitative (1-5 scale)	Multisectoral	Surveillance & Laboratory
	Data production network: Technical level of AMR data management of the laboratory network	Semi-quantitative (1-5 scale)	Multisectoral	Transparency
	Communication: External policy for communication with decision makers and other stakeholders	Semi-quantitative (1-5 scale)	Multisectoral	Transparency
	Communication: Identification and coverage of key stakeholders' expectations about the results of the surveillance system	Semi-quantitative (1-5 scale)	Multisectoral	Community Awareness & Enabling Behaviors
	Communication: Existence of awareness building AMR programs for surveillance actors	Semi-quantitative (1-5 scale)	Multisectoral	Transparency
	Communication: Communication of risk assessment outcomes to relevant parties	Semi-quantitative (1-5 scale)	Multisectoral	Transparency
	Communication: Regular release of reports on AMR surveillance results	Semi-quantitative (1-5 scale)	Multisectoral	Feedback Mechanisms
	Communication: Systematic distribution of AMR surveillance results to field actors (outside of a	Semi-quantitative (1-5 scale)	Multisectoral	Feedback Mechanisms

		report)			
		Communication: Presence of a communication system organized between field actors (mail, websites, telephone...)	Semi-quantitative (1-5 scale)	Multisectoral	Sustainability
		Sustainability: Adequacy of material and financial resources for the multi-sectoral working groups(s) or coordination committee on AMR	Semi-quantitative (1-5 scale)	Multisectoral	Sustainability
		Sustainability: Adequacy of financial resources for the implementation of the National AMR action plan	Semi-quantitative (1-5 scale)	Multisectoral	Sustainability
		Sustainability: Adequacy of human, material, and financial resources for AMR data production (laboratory network) needs	Semi-quantitative (1-5 scale)	Multisectoral	Sustainability
		Sustainability: Adequacy of human, material, and financial resources for AMR data production and analysis (epidemiology) needs	Semi-quantitative (1-5 scale)	Multisectoral	Workforce
		Sustainability: Adequacy of human, material, and financial resources for communication needs	Semi-quantitative (1-5 scale)	Multisectoral	Coordination
		Sustainability: Regular advanced training for actions of the surveillance	Semi-quantitative (1-5 scale)	Multisectoral	Sustainability
		Sustainability: Adequacy of material and financial resources for training	Semi-quantitative (1-5 scale)	Multisectoral	Feedback Mechanisms
		Sustainability: Regular measurement, interpretation, and dissemination of performance indicators	Semi-quantitative (1-5 scale)	Multisectoral	Stewardship
33	The costs and risks of AMR water pollution	Hospital and community use of antibiotics (DDD/1m)	Quantitative	Human Health	Stewardship
		Agricultural use of antibiotics (tonnes/100ht)	Quantitative	Environment	Social Determinants
		Sanitation services access gap (%)	Quantitative	Environment	Social Determinants
		Country pollution score (0-100): determined by clinical use, agricultural use, manufacturing, and mediated by whether wastewater treatment systems are in place	Semi-quantitative	Environment	Social Determinants / Prevention & Control
		Sanitation score (0-100) (based on the worst-performing sanitation indicator from: hand washing-prevalence of open defecation, access to basic sanitation, and access to clean water)	Semi-quantitative	Environment	Effectiveness
		Overall risk of AMR incidence (risk index 0-100)	Semi-quantitative	Multisectoral	Surveillance & Laboratory

34	Antimicrobial resistance in the EU/EAA (EARS-Net): Annual Epidemiological Report for 2023	Blood culture rate (blood culture sets/1000 patient-days)	Quantitative	Human Health	Surveillance & Laboratory
		Estimated incidence of isolates from bloodstream infections with resistance phenotype (n per 100 000 population): E. Coli (assessed for resistance to aminopenicillins; 3rd gen cephalosporins; carbapenems; fluoroquinolones; aminoglycosides; combined resistance)	Quantitative	Human Health	Surveillance & Laboratory
		Estimated incidence of isolates from bloodstream infections with resistance phenotype (n per 100 000 population): K. Pneumoniae (assessed for resistance to 3rd gen cephalosporins; carbapenems; fluoroquinolones; aminoglycosides; combined resistance)	Quantitative	Human Health	Surveillance & Laboratory
		Estimated incidence of isolates from bloodstream infections with resistance phenotype (n per 100 000 population): P. Aeruginosa (assessed for resistance to Piperacillin-tazobactam; ceftazidime; carbapenems; fluoroquinolones; aminoglycosides; combined resistance)	Quantitative	Human Health	Surveillance & Laboratory
		Estimated incidence of isolates from bloodstream infections with resistance phenotype (n per 100 000 population): Acinetobacter species (assessed for resistance to Carbapenems; Fluoroquinolones)	Quantitative	Human Health	Surveillance & Laboratory
		Estimated incidence of isolates from bloodstream infections with resistance phenotype (n per 100 000 population): Staphylococcus aureus (assessed for resistance to Aminoglycosides; combined resistance to carbapenems, fluoroquinolones and aminoglycosides; MRSA)	Quantitative	Human Health	Surveillance & Laboratory
		Estimated incidence of isolates from bloodstream infections with resistance phenotype (n per 100 000 population): Streptococcus pneumoniae (assessed for resistance to penicillin non-wild type; macrolides; combined penicillin non-wild-type and resistance to macrolides)	Quantitative	Human Health	Surveillance & Laboratory
		Estimated incidence of isolates from bloodstream infections with resistance phenotype (n per 100 000 population): Enterococcus faecalis (assessed	Quantitative	Human Health	Surveillance & Laboratory

for resistance to high-level gentamicin)			
Estimated incidence of isolates from bloodstream infections with resistance phenotype (n per 100 000 population): Enterococcus faecium (assessed for resistance to vancomycin)	Quantitative	Human Health	Surveillance & Laboratory
Total number of invasive isolates tested (n) and percentage of isolates with AMR phenotype (%): E. Coli (for 6 different antimicrobial groups)	Quantitative	Human Health	Surveillance & Laboratory
Total number of invasive isolates tested (n) and percentage of isolates with AMR phenotype (%): K. Pneumoniae (for 5 different antimicrobial groups)	Quantitative	Human Health	Surveillance & Laboratory
Total number of invasive isolates tested (n) and percentage of isolates with AMR phenotype (%): Pseudomonas aeruginosa (for 6 different antimicrobial groups)	Quantitative	Human Health	Surveillance & Laboratory
Total number of invasive isolates tested (n) and percentage of isolates with AMR phenotype (%): Acinetobacter species (for 4 different antimicrobial groups)	Quantitative	Human Health	Surveillance & Laboratory
Total number of invasive isolates tested (n) and percentage of isolates with AMR phenotype (%): Staphylococcus (MRSA)	Quantitative	Human Health	Surveillance & Laboratory
Total number of invasive isolates tested (n) and percentage of isolates with AMR phenotype (%): Streptococcus pneumoniae (for 3 different antimicrobial groups)	Quantitative	Human Health	Surveillance & Laboratory
Total number of invasive isolates tested (n) and percentage of isolates with AMR phenotype (%): Enterococcus faecalis (for high-level gentamicin)	Quantitative	Human Health	Surveillance & Laboratory
Total number of invasive isolates tested (n) and percentage of isolates with AMR phenotype (%): Enterococcus faecium (for vancomycin)	Quantitative	Human Health	
Percentage of WHO 'Access' group antibiotics consumed out of total AWaRe consumption (community and hospital sectors combined)	Quantitative	Human Health	Stewardship
Community consumption of antibacterials for systemic use (ATC group J01) by subgroup (ATC level 3)	Quantitative	Human Health	Stewardship

		Community consumption of antibacterials for systemic use (ATC group J01)	Quantitative	Human Health	Stewardship
		Ratio of consumption of broad-spectrum penicillins, cephalosporins, macrolides (except erythromycin) and fluoroquinolones to consumption of narrow-spectrum penicillins, cephalosporins and erythromycin in the community	Quantitative	Human Health	Stewardship
		Hospital sector consumption of antibacterials for systemic use (ATC group J01) by subgroup (ATC level 3)	Quantitative	Human Health	Stewardship
		Hospital sector consumption of antibacterials for systemic use (ATC group J01)	Quantitative	Human Health	Stewardship
		Percentage of glycopeptides, 3rd and 4th generation cephalosporins, monobactams, carbapenems, fluoroquinolones, polymyxins, piperacillin and enzyme inhibitor, linezolid, tedizolid and daptomycin out of total hospital sector consumption of antibacterials for systemic use	Quantitative	Human Health	Stewardship
		Percentage of WHO 'Reserve' group antibiotics consumed out of hospital sector AWaRe consumption	Quantitative	Human Health	Stewardship
		Total consumption (community and hospital sectors combined) of antimycotics and antifungals for systemic use (ATC group J01 & D01B)	Quantitative	Human Health	Stewardship
35	Evaluation of the Performance of Veterinary Services: PVS Tool (Terrestrial 2019)	Planning, sustainability and management of policies and programmes: the capability of the VS leadership and organization to develop, document and sustain strategic policies and programmes, and also to report on, review and evolve them, as appropriate over time	Qualitative (1-5 scale, each with different criteria)	Animal Health	Sustainability
		Coordination capability of the veterinary services: external coordination (including the One Health approach)	Qualitative (1-5 scale, each with different criteria)	Animal Health	Coordination
		Physical resources and capital investment: access of the VS to functional and well-maintained physical resources including buildings, transport, IT, cold chain, and other necessary equipment or	Qualitative (1-5 scale, each with different criteria)	Animal Health	Sustainability

	structures.			
	Regulation of the profession by the veterinary statutory body: the authority and capability of the VSB to effectively and independently maintain educational and professional standards for veterinarians and veterinary professionals	Qualitative (1-5 scale, each with different criteria)	Animal Health	Sustainability
	Operational funding: the ability of VS to access operational resources adequate for their planned and continued activities	Qualitative (1-5 scale, each with different criteria)	Animal Health	Sustainability
	Emergency funding: the capability of VS to access extraordinary financial resources in order to respond to emergency situations or newly emerging issues, as measured by the ease with which contingency and related funding	Qualitative (1-5 scale, each with different criteria)	Animal Health	Sustainability
	Veterinary laboratory diagnosis: access to veterinary laboratory diagnosis	Qualitative (1-5 scale, each with different criteria)	Animal Health	Access to Medicines & Health Services
	Veterinary laboratory diagnosis: sustainability of the national laboratory system	Qualitative (1-5 scale, each with different criteria)	Animal Health	Sustainability
	Veterinary laboratory diagnosis: laboratory quality management systems (QMS)	Qualitative (1-5 scale, each with different criteria)	Animal Health	Feedback Mechanisms
	Risk analysis and epidemiology: authority and capability of the VS to base its risk management and risk communication measures on risk assessment, incorporating sound epidemiological principles	Qualitative (1-5 scale, each with different criteria)	Animal Health	Feedback Mechanisms
	Quarantine and border security: authority and capability of the VS to operate and prevent the entry of diseases and other hazards of animals and animal and veterinary products into their country	Qualitative (1-5 scale, each with different criteria)	Animal Health	Prevention & Control
	Emergency preparedness and response: authority and capacity of VS to be prepared and respond rapidly to a sanitary emergency threat	Qualitative (1-5 scale, each with different criteria)	Multisectoral	Prevention & Control
	Animal production food safety: regulation, inspection (including audits), authorization and supervision of establishments for production and processing of food of animal origin	Qualitative (1-5 scale, each with different criteria)	Animal Health	Prevention & Control

	Animal production food safety: ante- and post-mortem inspection at slaughter facilities and associated premises	Qualitative (1-5 scale, each with different criteria)	Animal Health	Prevention & Control
	Veterinary medicines and biologicals: the authority and capability of the VS to regulate veterinary medicines, and biologicals, in order to ensure their quality and safety, as well as their responsible and prudent use, including as medicated feed	Qualitative (1-5 scale, each with different criteria)	Animal Health	Regulations & Legislation
	Antimicrobial resistance and antimicrobial use: the authority and capability of the VS to manage AMR and AMU, and to undertake surveillance and control of the development and spread of AMR pathogens in animal production and animal origin food products, via a One Health approach	Qualitative (1-5 scale, each with different criteria)	Multisectoral	Accountability
	Residue testing, monitoring, and management: the capability of the VS to undertake residue testing and monitoring programmes, for veterinary medicines, chemicals, pesticides, radionuclides, heavy metals, etc. and respond appropriately to adverse findings	Qualitative (1-5 scale, each with different criteria)	Animal Health	Surveillance & Laboratory
	Animal feed safety: the authority and capability of the VS to regulate animal feed safety	Qualitative (1-5 scale, each with different criteria)	Animal Health	Regulations & Legislation
	Identification, traceability and movement control: premises, herd, batch and animal identification, tracing and movement control	Qualitative (1-5 scale, each with different criteria)	Animal Health	Prevention & Control
	Identification, traceability and movement control: identification, traceability and control of products of animal origin	Qualitative (1-5 scale, each with different criteria)	Animal Health	Prevention & Control
	Animal welfare: the authority and capability of the VS to legislate and implement the animal welfare standards of WOAH as published in the Terrestrial Code	Qualitative (1-5 scale, each with different criteria)	Animal Health	Regulations & Legislation
	Communicate: The capability of the VS to keep non-government stakeholders aware and informed, in a transparent and effective and timely manner, of VS activities and programmes, and of developments in animal health, animal welfare and veterinary public health	Qualitative (1-5 scale, each with different criteria)	Animal Health	Transparency

Consultation with stakeholders: Capability of the VS to consult effectively with non-government stakeholders on VS policies and programmes and on developments in animal health and food safety	Qualitative (1-5 scale, each with different criteria)	Animal Health	Coordination
Official representation and international collaboration: capability of the VS to regularly and actively participate, coordinate and provide follow-up on relevant meetings and activities of regional and international organizations	Qualitative (1-5 scale, each with different criteria)	Animal Health	Coordination/ Participation
Accreditation/authorization/delegation: The authority and capability of the public sector of the VS to accredit/authorize/delegate to private sector or NGO expertise to carry out official tasks on their behalf, usually via formal agreement	Qualitative (1-5 scale, each with different criteria)	Animal Health	Accountability
Participation of producers and other stakeholders in joint programmes: the capability of the VS to develop joint programs with producers and non-government stakeholders to deliver animal health, veterinary public health, food safety and/or animal welfare outcomes	Qualitative (1-5 scale, each with different criteria)	Animal Health	Participation
International harmonization: the authority and capability of the VS to be active in the harmonization of national veterinary legislation and sanitary measures to ensure they take into account international standards, and/or related regional directives or guidelines	Qualitative (1-5 scale, each with different criteria)	Animal Health	Regulations & Legislation
International certification: authority and capability of the VS to reliably certify animals and animal products, and related services and processes under their mandate, for export, in accordance with national veterinary legislation, international standards and importing country requirements	Qualitative (1-5 scale, each with different criteria)	Animal Health	Regulations & Legislation
Equivalence and other types of sanitary agreements: the authority and capability of the VS to apply flexibility in negotiating, implementing and maintaining equivalence and other types of sanitary agreements with trading partners	Qualitative (1-5 scale, each with different criteria)	Animal Health	Regulations & Legislation
Transparency: the authority and capability of the VS to notify WOAH, WTO, trading partners and	Qualitative (1-5 scale, each with different	Animal Health	Transparency

		other relevant organizations of its disease status, regulations and sanitary measures and systems, in accordance with established procedures, as applicable to international trade	criteria)		
		Zoning: the authority and capability of the VS to establish and maintain disease free zones, as necessary and in accordance with the criteria established by WOAH	Qualitative (1-5 scale, each with different criteria)	Animal Health	Prevention & Control
		Compartmentalization: the authority and capability of the VS to establish and maintain disease free compartments in accordance with the criteria established by WOAH	Qualitative (1-5 scale, each with different criteria)	Animal Health	Prevention & Control
36	Antimicrobial stewardship programmes in health-care facilities in LMICs: a WHO practical toolkit	Appropriate use of antimicrobials: percentage of inpatient surgical procedures with appropriate timing and duration of surgical antibiotic prophylaxis	Quantitative	Human Health	Stewardship
		Antimicrobial use: DDD per admission	Quantitative	Human Health	Stewardship
		Antimicrobial use: DOT per 1000 patient-days	Quantitative	Human Health	Stewardship
37	Monitoring and evaluation of the global action plan on antimicrobial resistance: framework and recommended indicators	Impact of infectious diseases: burden of infectious disease in disability-adjusted life-years per 100 000 population	Quantitative	Human Health	Effectiveness
		Patterns and trends in resistance in human health: prevalence of bloodstream infections caused by methicillin-resistant Staphylococcus aureus and ESBL in E. Coli - third-generation cephalosporin resistance as a proxy	Quantitative	Human Health	Surveillance & Laboratory
		Patterns and trends in resistance for indicator E. Coli from priority food producing species: Resistance in commensal E. Coli from key food producing animals as follows: percentage of E. Coli isolates showing resistance to 3rd generation cephalosporins; patterns of resistance in E. Coli to a defined panel of antimicrobials	Quantitative	Animal Health	Surveillance & Laboratory
		Percentage of stakeholders (e.g. human and animal health workers, prescribers, farmers, food processing workers) who have knowledge of AMR and the implications for AMU and infection prevention	Quantitative	Multisectoral	Workforce

	Incidence of surgical site infections - inpatient surgical procedures	Quantitative	Human Health	Prevention & Control
	Percentage of the target population that has received the last recommended dose of the basic series for each of the following vaccines: pneumococcal conjugate vaccine; rotavirus vaccine; measles-containing vaccine, either alone, or in a measles-rubella or measles-mumps-rubella combination; haemophilus influenzae type b containing vaccine (Hib)	Quantitative	Human Health	Prevention & Control
	Proportion of population using safely managed drinking-water services	Quantitative	Environment	Social Determinants / Prevention & Control
	Proportion of population using safely managed sanitation services	Quantitative	Environment	Social Determinants / Prevention & Control
	Number of state parties to international multilateral environmental agreements on hazardous waste and other chemicals that meet their commitments and obligations in transmitting information as required by each relevant agreement	Quantitative	Environment	Transparency / Accountability
	Hazardous waste generated per capita and proportion of hazardous waste treated, by type of treatment	Quantitative	Environment	Social Determinants
	Total consumption of antibiotics for systemic use (Anatomical Therapeutic Chemical classification code J01) in DDD per 1000 population per day	Quantitative	Human Health	Stewardship
	Proportion of Access antibiotics for systemic use, relative to total antibiotics for systemic use, relative to total antibiotic consumption in DDDs	Quantitative	Human Health	Stewardship
	Relative proportion of AWaRe antibiotics for paediatric formulations	Quantitative	Human Health	Stewardship
	Percentage of adult and paediatric hospital patients receiving an antibiotic according to AWaRe categories	Quantitative	Human Health	Stewardship
	Percentage of health facilities that have a core set of relevant antibiotics available and affordable on a sustainable basis	Quantitative	Human Health	Stewardship
	Percentage of inpatient surgical procedures with appropriate timing and duration of surgical antibiotic prophylaxis	Quantitative	Human Health	Stewardship

	Percentage of veterinary AMs authorized/used for non-veterinary medical use (e.g. for growth promotion)	Quantitative	Animal Health	Stewardship
	Total volume of sales/imports (or use) in mg/kg biomass, in food producing animals	Quantitative	Animal Health	Stewardship
	Percentage of total sales/imports (or use) classified as WHO Highest Priority Critically Important Antimicrobial agents	Quantitative	Human Health	Stewardship
	Total amount of pesticide (active substance) intended to repel, destroy or control bacterial or fungal disease (tonnes)	Quantitative	Environment	Stewardship
	Percentage of the above total composed of each of the following antimicrobial classes: aminoglycosides, tetracyclines, triazoles, oxolinic acid	Quantitative	Environment	Stewardship
	Legislation or regulation that requires antimicrobials for human use to be dispensed only with a prescription from an authorized health worker	Qualitative	Human Health	Regulations & Legislation
	Countries that have a regulatory framework for veterinary medical products (including medicated feed) that covers all stages of the cycle (manufacture, supply, sale, use, disposal) and meets other requirements in the OIE and Codex standards	Quantitative	Animal Health	Regulations & Legislation
	Countries that have a regulatory framework for the discharge of antimicrobials and waste potentially contaminated with antimicrobials into the environment	Quantitative	Environment	Regulations & Legislation
	Number of new medicines in the R&D pipeline targeting products on the WHO global priority pathogens list (antimicrobials and alternative treatments)	Quantitative	Multisectoral	Research, Innovation, & Digital Technology
	Number of new diagnostic products in the R&D pipeline responding to the essential diagnostics list (forthcoming)	Quantitative	Multisectoral	Research, Innovation, & Digital Technology
	Number of new Vaccines registered according to prioritization (OIE reports on prioritization of diseases for which vaccines could reduce	Quantitative	Animal Health	Prevention & Control

antimicrobial use in pig, poultry and fish, 2015, and in cattle, sheep, and goats, 2018)			
Nationwide, government-supported AMR awareness campaign priority stakeholder groups in the following sectors: human health, animal health, planet health, food production, food safety, environment	Qualitative	Multisectoral	Community Awareness & Enabling Behaviors
Countries that report to GLASS on: AMR in humans, AMU in humans	Quantitative	Human Health	Reporting
Countries that report information on total quantities of antimicrobial agents sold for/imported for/used in food producing animals	Quantitative	Animal Health	Reporting
Countries that have systems to collect and report information on the quantity of pesticides used to control bacteria or fungal diseases in plant production	Quantitative	Environment	Reporting
Percentage of laboratories included in the national AMR surveillance system in the food and agricultural sectors with capacity to perform antimicrobial susceptibility testing and/or bacterial isolation and identification according to international standards	Quantitative	Animal Health	Surveillance & Laboratory
Robustness of the national AMR laboratory network included in the AMR surveillance system for the veterinary, food and agricultural sectors	Quantitative	Animal Health	Surveillance & Laboratory
Countries that collect and report AMR surveillance data for: food producing animals and food	Quantitative	Animal Health	Surveillance & Laboratory
Countries that measure the prevalence of ESBL-producing indicator commensal E.Coli in key food producing species (terrestrial) in accordance with the OIE Terrestrial Animal Health Code and the OIE Manual of Diagnostic Tests and Vaccines for Terrestrial Animals	Quantitative	Animal Health	Surveillance & Laboratory
National bodies that review information from national AMR surveillance programs, and make and implement recommendations accordingly	Quantitative	Multisectoral	Feedback Mechanisms
Countries that achieve level III or more on PVS Critical competency II-9: the authority and capability of the veterinary services to manage	Quantitative	Animal Health	Surveillance & Laboratory

		AMU and AMR, and to undertake surveillance and control of the development and spread of AMR pathogens in animal production and animal origin food products, via a One Health approach			
		Level of access to veterinary advice and care within a country (e.g. number of qualified veterinarians and/or veterinary paraprofessionals per animal population)	Quantitative	Animal Health	Workforce
		Countries that implement minimum requirements for infection prevention for food animal production, in accordance with OIE standards	Quantitative	Animal Health	Prevention & Control
		Percentage of healthcare facilities where the main source of water is from an improved source, located on the premises	Quantitative	Human Health	Prevention & Control / Social Determinants
		Proportion of health care facilities with improved and usable sanitation facilities, with at least one toilet dedicated for staff, at least one sex-separated toiled with menstrual hygiene facilities and at least one toilet accessible for users with limited mobility	Quantitative	Human Health	Prevention & Control
		Countries that have a regulatory framework for pesticides that considers all stages of the antimicrobial life cycle (production, supply, sale, use, disposal) and meets the other requirements in the reference international standards	Quantitative	Environment	Regulations & Legislation
		Countries that have laws or regulations that prohibit use of antibiotics for growth promotion in the absence of risk analysis	Quantitative	Animal Health	Regulations & Legislation
		Countries that have adopted food safety standards consistent with the Codex Alimentarius	Quantitative	Environment	Prevention & Control
		Mechanisms and investments for R&D: list of mechanisms and funding for R&D to prevent, diagnose and treat priority pathogens	Qualitative	Human Health	Research, Innovation, & Digital Technology
		Mechanisms and investments for R&D: list of mechanisms, commitments and expenditures for R&D targeting priority pathogens	Qualitative	Multisectoral	Research, Innovation, & Digital Technology
38	Worldwide country situation analysis: response to antimicrobial resistance	Percentages of member states that had conducted a public information campaign about use of antimicrobial medicines in the previous 2 years	Qualitative	Multisectoral	Community Awareness & Enabling Behaviours
		Percentages of Member states that had an infection	Qualitative	Human Health	Prevention & Control

		prevention and control programme and in which all tertiary hospitals had such a programme, all regions			
39	Eurostat	Consumption of antibiotics in the community and hospital sectors - defined daily doses (DDD) per day	Quantitative	Human Health	Stewardship
40	Monitoring Framework for the WHO Strategic and Operational priorities to address drug-resistant bacterial infections	I.1.a Number of global deaths from bacterial infections in the human health sector (total)	Quantitative	Human Health	Surveillance & Laboratory
		I.1.b Number of global deaths associated with drug-resistant bacterial infections in the human health sector	Quantitative	Human Health	Surveillance & Laboratory
		OC.1 SDG 3.d.2 Percentage of infections due to two selected bacterial pathogens that are resistant to specific antibiotics	Quantitative	Human Health	Surveillance & Laboratory
		S.1.1.a Proportion of population using safely managed sanitation services	Quantitative	Human Health	Prevention & Control
		S.1.1.b. Proportion of population using a hand-washing facility with soap and water	Quantitative	Human Health	Prevention & Control
		S.1.2.a Percentage of health care facilities with access to basic water services	Quantitative	Human Health	Prevention & Control
		S.1.2.b Percentage of health care facilities with access to basic water services	Quantitative	Human Health	Prevention & Control
		S.1.3.a Percentage of target population that received Pneumococcal conjugate vaccine	Quantitative	Human Health	Prevention & Control
		S.1.3.c Percentage of target population that received Haemophilus influenzae type b vaccine	Quantitative	Human Health	Prevention & Control
		Percentage of target population that received diphtheria-tetanus-pertussis vaccine	Quantitative	Human Health	Prevention & Control
		S.2.1 SDG 3.8.1 Universal Health Coverage of essential health services - Index of 14 tracer indicators	Quantitative	Human Health	Access to Medicines & Health Services
		S.2.3 Antibiotic use in defined daily dose per 1000 population per day; overall and by AWaRe classification	Quantitative	Human Health	Stewardship
		S.3.4.a Number of vaccines in R&D pipeline based on Bacterial Priority Pathogens List	Quantitative	Human Health	Research, Innovation, & Digital Technology
		S.3.4.c Number of antibiotics/antibacterials in clinical development in R&D pipeline based on Bacterial Priority Pathogens List	Quantitative	Human Health	Research, Innovation, & Digital Technology

		O.2.3.a Financial investments/incentives for R&D: Total value of R&D investment/push incentives	Quantitative	Human Health	Research, Innovation, & Digital Technology
		O.2.3.b Financial investments/incentives for R&D: Number of countries implementing pull incentives	Quantitative	Human Health	Research, Innovation, & Digital Technology
		O.3.5 Proportion of research priorities in WHO's human health research agenda that are being addressed	Quantitative	Human Health	Strategic Vision
41	International Health Regulations States Parties Self-assessment annual reporting tool	C1.1. Policy, legal and normative instruments: A) The country has not conducted a mapping of relevant legal and normative instruments and policies for IHR implementation. B) The country has conducted a legal analysis (e.g., a legal mapping and assessment) of relevant legal and normative instruments and policies for IHR implementation at the national and subnational levels and documented, where applicable. C) The country has identified and reviewed gaps in the health sector and developed and/or revised the necessary legal and normative instruments and policies for IHR implementation at the national and subnational levels, where applicable. D) The country has identified and reviewed gaps in all sectors and across government levels and developed and/or revised the necessary legal and normative instruments and policies for IHR implementation at the national and subnational levels, where applicable. E) The country has identified and reviewed gaps in all sectors and across government levels and developed and/ or revised the necessary legal and normative instruments and policies for IHR implementation in all sectors and across government levels, which are regularly evaluated and improved based on lessons learned from real-world events and exercises (as applicable).	Multiple Choice	Multisectoral	Prevention & Control / Regulations & Legislation
		C1.2. Gender Equality in health emergencies: A) No systematic assessment of gender gaps in any of the IHR capacities has been conducted. B) Systematic assessment of gender gaps has been	Multiple Choice	Human Health	Equity

	conducted in at least one IHR capacity. C) An action plan to address identified high priority gender gaps in at least one IHR capacity is developed and incorporated in annual workplans. D) The developed action plan(s) to address at least one IHR capacity is funded and being implemented, with mechanisms in place for monitoring, evaluation and reporting. E) Systematic gender analysis of IHR capacities is conducted, and action plans to address gender gaps and inequalities are developed, funded and operationalized in at least three IHR capacities, with mechanisms in place for monitoring, evaluation and reporting.			
	C2.1 National IHR Focal point functions: Multiple choice. A) The terms of reference describing the roles and responsibilities of the established IHR National Focal Point are not in place or under development and represented by one individual who is entirely familiar with the mandatory National Focal Point functions under the IHR but lacks the authority, capacity and resources to effectively carry out these functions, including the around-the-clock accessibility. B) National IHR Focal Point is a designated centre and has a duty officer system to ensure accessibility at all times for urgent communications with WHO but legal, normative and institutional instruments and arrangements, including terms of reference describing the roles and responsibilities, are insufficient to communicate effectively with all levels and relevant sectors of the State Party's administration. C) National IHR Focal Point is a designated centre and has a clear legal and governmental mandate, with terms of reference describing the roles and responsibilities, is sufficiently organized, resourced and accessible at all times to communicate with WHO, but intersectoral collaboration and communication are	Multiple Choice	Multisectoral	Prevention & Control

inadequate to consolidate surveillance information or to obtain clearance from decision-makers in other domestic sectors. D) National IHR Focal Point is a centre sufficiently organized, resourced and positioned within the government with levels of authority and institutional arrangements and instruments to access the relevant information sources and decision-making level within the national surveillance and response system. E) National IHR Focal Point is a centre appropriately organized, positioned, trained and equipped with adequate levels of authority, efficient communication channels as well as administrative, human, technological, and financial resources to meaningfully engage with all relevant sectors and carry out the function as by IHR provisions and its functioning is exercised, reviewed, evaluated and updated on a regular basis and actions have been taken to strengthen and maintain its capacities.			
C2.2 Multisectoral coordination mechanisms: A) Multisectoral coordination mechanisms for IHR implementation are not in place or under development. Multisectoral coordination activities occur in ad hoc basis. B) Multisectoral coordination mechanisms for IHR implementation are developed but not disseminated. Multisectoral coordination activities occur in ad hoc basis. C) Multisectoral coordination mechanisms for IHR implementation are in place, disseminated and are being implemented at national level. D) Multisectoral coordination mechanisms for IHR implementation are in place, disseminated and are being implemented at national and intermediate levels. E) Multisectoral coordination mechanisms for IHR implementation are being implemented at all levels, and are exercised, reviewed, evaluated and updated on a regular basis.	Multiple Choice	Multisectoral	Coordination / Prevention & Control
C2.3 Advocacy for IHR implementation: A) Advocacy mechanisms for IHR implementation	Multiple Choice	Multisectoral	Sustainability / Prevention & Control

are not in place or under development. Advocacy activities are conducted on ad hoc basis. B) The advocacy mechanisms are developed but not disseminated. Advocacy activities are conducted on ad hoc basis. C) The advocacy mechanisms are in place, disseminated and being implemented at the national level. D) The advocacy mechanisms are in place, disseminated and being implemented at the national and intermediate levels. E) The advocacy mechanisms are implemented at all levels in a multisectoral and whole of society approach. Mechanisms are exercised, reviewed, evaluated and updated on a regular basis at national, intermediate and local levels based on best practices and lessons learned.			
C3.1 Financing for IHR implementation: A) There is no financial planning, budget line or budgetary allocation available to finance IHR implementation, and is handled through extrabudgetary means. B) Financial planning is limited with a budgetary allocation or substantial external financing made for some of the relevant sectors and their respective ministries to support the IHR implementation at the national level. C) Financial planning based on identified gaps and estimated resource needs with a budgetary allocation and/or substantial external financing made for relevant sectors is available to support IHR implementation at national level and some monitoring and accountability mechanisms are in place. D) Financial planning based on identified gaps and estimated resource needs with sufficient budgetary allocation for IHR implementation, that may include external financing. The budget is predictable, flexible, and distributed in a timely manner at the national and intermediate levels in all relevant ministries or sectors, with monitoring and accountability mechanisms in place to measure implementation and effectiveness. E) Financial	Multiple Choice	Multisectoral	Sustainability / Prevention & Control

	planning with sufficient budgetary allocation for IHR implementation, that may include external financing is available at national, intermediate and local levels and all sectors; with predictable and flexible budget, distributed in a timely manner. The country is able to collaborate and provide financial support to other countries considering regional priorities, needs and global threats The budget is monitored against objectives, and accountability mechanisms are in place at each level for transparent and effective use of funds.			
	C3.2 Financing for public health emergency response: A) Public financing for responding to public health emergencies is not identified and funds are allocated and distributed in an ad hoc manner. B) Public financing exists that allows for structured reception, rapid distribution and use of funds for responding to public health emergencies. C) Public financing for responding to public health emergencies is identified for immediate mobilization when needed, at the national level for all the relevant sectors in advance of a public health emergency. D) Public financing for responding to public health emergencies is in place at national and intermediate levels and allows for the timely execution of funds by all relevant sectors during a public health emergency. E) Public financing for responding to public health emergencies in place, with an appropriate emergency contingency, at national intermediate and local levels, that allows for the timely execution of funds by all relevant sectors during a public health emergency. The country is able to collaborate and provide financial support to other countries during a public health emergency.	Multiple Choice	Multisectoral	Sustainability / Prevention & Control
	C4.1 Specimen referral and transport system: A) No system in place for transporting specimens from intermediate levels/districts to national laboratories; only ad hoc transportation is	Multiple Choice	Multisectoral	Surveillance & Laboratory

	available. B) Referral and transport of specimens is organized for some priority diseases but may be restricted within districts or at the intermediate and national level. C) Referral and transport of specimens is organized for diagnostics and/or confirmation of most priority diseases from subnational to national level. D) Referral and transport of specimens is organized systematically for diagnostics and/or confirmation of all priority diseases at all levels. E) Sustainable referral and transport systems, that are exercised (as appropriate) reviewed, evaluated and updated on a regular basis, are in place for all specimen types and requests for the diagnosis, confirmation, characterization of all specimens with complete coverage at all levels.			
	C4.2. Implementation of a laboratory and biosecurity regime: A) National laboratory biosafety and biosecurity guidelines and/or regulations are under development. B) National laboratory biosafety and biosecurity guidelines and/or regulations are in place and implemented by some laboratories at the national level. C) National laboratory biosafety and biosecurity guidelines and/or regulations are in place and implemented by all laboratories at the national level. D) National laboratory biosafety and biosecurity guidelines and/or regulations are implemented by all laboratories at national, intermediate and local levels. E) National laboratory biosafety and biosecurity guidelines and/or regulations are exercised, reviewed, evaluated and updated on a regular basis, as applicable and a system for oversight of the regulation is in place.	Multiple Choice	Multisectoral	Surveillance & Laboratory
	C4.3 Laboratory quality system: A) National laboratory quality standards are not available or under development. B) National quality standards have been developed but not implemented. C) National quality standards have been developed	Multiple Choice	Multisectoral	Surveillance & Laboratory

	and implemented at national level. Activities include licensing of laboratories in conformity with national quality standards. D) National quality standards have been developed and are being implemented at national and subnational levels. Activities include mandatory licensing of laboratories in line with basic quality requirements or national laboratory standards. E) National quality standards are implemented at all levels including mandatory licensing of all laboratories in conformity with international quality standards and exercised, reviewed, evaluated and updated on a regular basis, as applicable			
	C4.4 Laboratory testing capacity modalities: A) Laboratory system can support one or two testing modalities such as rapid diagnostic testing (antigen and antibody) and microscopy services for pathogen detection. B) Laboratory system can support testing modalities including serological tests (i.e., antigen and antibody enzyme immunoassays) and quality assurance process is in place. C) Laboratory system can perform nucleic acid amplification testing, bacterial culture with antimicrobial sensitivity testing with quality assurance process in place and have access to (or has) sequencing capacity. D) Laboratory system can perform nucleic acid amplification testing, bacterial culture with antimicrobial sensitivity testing with quality assurance process in place and has some basic sequencing capacity and country has ability to test for all its endemic diseases and its priority diseases. E) Laboratory system can perform in all capacities including access to whole genome sequencing; identification of unknown and high consequence pathogens and has access to viral culture. Laboratory networks configured to support all diagnostic services30 that are integrated, sustainable, with maximum population coverage, and exercised, reviewed, evaluated and	Multiple Choice	Multisectoral	Surveillance & Laboratory

updated on a regular basis as applicable			
C4.5 Effective national diagnostic network: A) Tier-specific diagnostic testing strategies are not available or under development. B) Tier-specific diagnostic testing strategies are developed. C) Tier-specific diagnostic testing strategies exist, but not fully implemented. D) Tier-specific diagnostic testing strategies are being implemented at national level. E) Tier-specific diagnostic testing strategies are being implemented at national, intermediate and local levels, and exercised, reviewed, evaluated, and updated on a regular basis, as applicable.	Multiple Choice	Multisectoral	Surveillance & Laboratory
C5.1. Early warning surveillance function: A) National guidelines and/or SOPs for surveillance are not available or under development. B) National guidelines and/or SOPs for surveillance have been developed but not implemented. The surveillance system is functioning but lacks systematic immediate reporting or weekly reporting of events and/or data. C) National guidelines and/or SOPs for surveillance have been developed and are being implemented at the national level and provide immediate and weekly reporting of events and/or data. D) National guidelines and/or SOPs for surveillance have been developed and are being implemented at the national and intermediate levels and provide immediate and weekly reporting of events and/or data. E) National guidelines and/or SOPs for surveillance have been developed and implemented at national, intermediate and local levels; and the system is exercised (as applicable), reviewed, evaluated and updated on a regular basis, with improvement at all levels in the country.	Multiple Choice	Multisectoral	Surveillance & Laboratory
C5.2. Event management (i.e., verification, investigation, analysis, and dissemination of information): A) Process or mechanism for	Multiple Choice	Multisectoral	Prevention & Control

	managing detected events is not available or under development. B) Process or mechanism for managing detected events has been developed but not implemented. C) Process or mechanism for managing detected events has been developed and is being implemented at the national level. D) Process or mechanism for managing detected events has been developed and is being implemented at the national and intermediate levels. E) Process or mechanism for managing detected events is being implemented at national, intermediate and local levels, and exercised (as applicable), reviewed, evaluated and updated on a regular basis.			
	C6.1. Human resources for implementation of IHR: A) Country does not have appropriate human resources capacity in relevant sectors required, to detect, assess, notify, report and respond to events according to IHR provision. B) Appropriate human resources are available in relevant sectors at national level, to detect, assess, notify, report and respond to events according to IHR provisions. C) Appropriate human resources are available in all relevant sectors at national and intermediate levels, to detect, assess, notify, report and respond to events according to IHR provisions. D) Human resources are available as required in all relevant sectors at the national, intermediate and local levels, to detect, assess, notify, report and respond to events according to IHR provisions. E) Country has documented policies or procedures for sustainable appropriate human resources in all relevant sectors to detect, assess, notify, report and respond to events according to IHR provisions, that are exercised (as applicable), reviewed, evaluated and updated on a regular basis and country may assist other countries in planning and developing human resources for IHR implementation, to the extent possible.	Multiple Choice	Multisectoral	Workforce

C6.2. Workforce surge during a public health event: A) A national multisectoral workforce surge strategic plan in emergencies is not available or is under development. B) A national multisectoral workforce surge strategic plan in emergencies is developed to carry out the functions attributed at the national level, including the government and nongovernmental partners workforce as applicable. C) A national multisectoral workforce surge strategic plan in emergencies is implemented to carry out the functions attributed at the national level, with procedures and limited capacity to send and receive multidisciplinary personnel within the country (shifting resources), including the government and nongovernmental partners workforce as applicable. D) A national multisectoral workforce surge strategic plan in emergencies is implemented to carry out the functions at national and intermediate levels, with procedures and adequate capacity to send and receive multidisciplinary personnel within the country (shifting resources), including the government and nongovernmental partners workforce as applicable. E) A national multisectoral workforce surge strategic plan in emergencies is implemented to carry out the functions attributed at national, intermediate and local levels, with procedures and adequate capacity to send and receive multidisciplinary personnel within the country (shifting resources), including the government and nongovernmental partners workforce, as applicable, and exercised, reviewed, evaluated and updated annually, and may well provide international collaboration for assisting emergency response.	Multiple Choice	Multisectoral	Workforce
C7.1. Planning for health emergencies: A) All-hazard risk informed41 health emergency plan is not available or under development. B) All-hazard risk informed health emergency plan is developed	Multiple Choice	Multisectoral	Prevention & Control

but not being implemented. C) All-hazard risk informed health emergency plan is developed and being implemented at the national level. D) All-hazard risk informed health emergency plan is developed and being implemented at the national and intermediate levels. E) All-hazard risk informed health emergency plan is developed and being implemented at national, intermediate and local levels and exercised, reviewed, evaluated and updated, with improvements based on SimE and lessons learned from real-world events, e.g., IARs or AARs.			
C7.2. Management of health emergency response: A) An incident management system integrated with a national public health emergency operations centre or equivalent structure is not available or under development. B) An incident management system integrated with a national public health emergency operations centre, or equivalent structure is developed but not operational. C) An incident management system integrated with a national public health emergency operations centre, or equivalent structure is in place and operational at the national level. D) An incident management system integrated with a national public health emergency operations centre, or equivalent structure is in place and operational at the national level and able to support intermediate levels. E) An incident management system integrated with a national levels public health emergency operations centre, or equivalent structure is in place and operational at national level and is able to support national, intermediate and local levels, and is exercised, reviewed, evaluated and updated, with improvements based on SimEx and lessons learned from real-world events, e.g. IARs or AARs.	Multiple Choice	Multisectoral	Prevention & Control
C7.3. Emergency logistic and supply chain management: A) Emergency logistics and supply	Multiple Choice	Multisectoral	Prevention & Control

chain management system/mechanism is under development and/or not able to provide adequate support for health emergencies. B) Emergency logistics and supply chain management system/mechanism is developed but not able to provide adequate support for health emergencies. C) Emergency logistics and supply chain management system/mechanism is developed and is able to provide adequate support for health emergencies at national level. D) Emergency logistics and supply chain management system/mechanism is developed and is able to provide adequate support for health emergencies at national and intermediate levels. E) Emergency logistics and supply chain management system/mechanism is implemented at national, intermediate and local levels, and is exercised (as appropriate), reviewed, evaluated and updated on a regular basis.			
C8.1. Case management: A) National clinical case management guidelines for priority health events are not available or under development. B) National clinical case management guidelines for priority health events are developed but not being implemented. C) National clinical case management guidelines for priority health events are developed and being implemented at national level. D) National clinical case management guidelines for priority health events are developed and being implemented at national and subnational levels. E) National clinical case management guidelines for priority health events are implemented at all levels and are exercised (as applicable), reviewed, evaluated and updated on regular basis.	Multiple Choice	Multisectoral	Prevention & Control
C8.2. Trusted and utilized health services: A) Very low levels of outpatient service utilization* (number of outpatient contacts per person per year < 1.00 visit/person/ year; in both urban and rural	Multiple Choice	Human Health	Access to Medicines & Health Services

	areas). B) Low levels of outpatient service utilization (number of outpatient department visits per person per year 1.0 ≤ X < 2.0 visit/person/year; in both urban and rural areas). C) Satisfactory levels of outpatient service utilization (number of outpatient contacts per person per year ≥ 2.0 visit/person/year, in both urban and rural areas). D) Strong levels of outpatient service utilization (number of outpatient contacts per person per year ≥ 3.0 visit/person/ year, in both urban and rural areas). E) Strong levels of outpatient service utilization (number of outpatient contacts per person per year ≥ 3.0 visit/person/ year, in both urban and rural areas) AND Information on service utilization and from patient experience (or satisfaction) surveys is reviewed on an annual basis and used to improve access and quality of services.			
	C8.3. Continuity of essential health services (EHS): A) A package of EHS is not defined and there are no plans or guidelines for continuity EHS during emergency. B) A package of EHS is defined but plans/guidelines on continuity of EHS in emergencies is not developed. C) A package of EHS and plans/guidelines on continuity of EHS in emergencies are developed and mechanism for monitoring service continuity during emergency is in place at national level. D) A package of EHS and plans/guidelines on continuity of EHS in emergencies are developed and mechanism for monitoring service continuity during emergency is in place at national and intermediate levels. E) A package of EHS, plans/guidelines on continuity of EHS in emergencies, and mechanisms for monitoring service continuity based on existing guidelines are defined and functional at national, intermediate and local levels and exercised, reviewed, evaluated and updated, with improvements based on SimEx and lessons learned	Multiple Choice	Human Health	Access to Medicines & Health Services

	from real- world events, e.g. IARs or AARs.			
	C9.1. IPC programmes: A) An active national IPC programme or operational plan according to the WHO minimum requirements is not available or is under development. B) An active national IPC programme or operational plan according to WHO minimum requirements exists but is not fully implemented. National IPC guidelines/ standards exist but are not fully implemented. C) An active national IPC programme exists, and a national IPC operational plan according to the WHO minimum requirements is available. National guidelines/standards for IPC in health care are available and disseminated. Selected health facilities are implementing guidelines using multimodal strategies, including health workers' training and monitoring and feedback. D) An active national IPC programme is available according to WHO IPC core components guidelines and is leading implementation of the national IPC operational plan and guidelines nationwide using multimodal strategies, including health workers' training and monitoring and feedback in place. More than 75% of health care facilities meet WHO minimum requirements for IPC programmes, guidelines, training, and monitoring/feedback. E) IPC programmes are in place and functioning at national and health facility levels according to the WHO IPC core components and their compliance and effectiveness are exercised (as applicable), reviewed, evaluated and published. Plans and guidance are regularly updated in response to monitoring and feedback	Multiple Choice	Human Health	Prevention & Control
	C9.2. Health care-associated infections (HCAI) surveillance: A) No national HCAI surveillance programme or national strategic plan for HCAI surveillance, including pathogens that are antimicrobial resistant and/or prone to outbreaks is	Multiple Choice	Human Health	Prevention & Control

	available or under development. B) A national strategic plan for HCAI surveillance (including antimicrobial resistant pathogens that are antimicrobial resistant and/or prone to outbreaks) is available but not implemented. C) A national strategic plan for HCAI surveillance (including antimicrobial resistant pathogens that are antimicrobial resistant and/or prone to outbreaks) is available and implemented through a national system. Selected secondary and tertiary health care facilities are conducting HCAI surveillance (as specified above) and provide timely and regular feedback to senior management and health workers. D) A national strategic plan for HCAI surveillance (including antimicrobial resistant pathogens that are antimicrobial resistant and/or prone to outbreaks) is available and implemented nationwide in all health care facilities through a national system according to the WHO recommendations on IPC core components. Regular reports are available for providing feedback. E) A national strategic plan for HCAI surveillance (including antimicrobial resistant pathogens that are antimicrobial resistant and/or prone to outbreaks) is available and implemented nationwide in health care facilities through a national system according to the WHO recommendations on IPC core components. Data are shared and being used continuously and in a timely manner to inform prevention efforts. The quality and impact of the system are regularly evaluated, and improvement actions are taken accordingly.			
	C9.3. Safe environment in health facilities: A) National standards and resources for safe built environment, e.g., water, sanitation and hygiene (WASH) in health care facilities, including appropriate infrastructure, materials and equipment for IPC; as well as standards for reduction of	Multiple Choice	Human Health	Prevention & Control

overcrowding and for optimization of staffing levels in health care facilities are not available or under development. B) National standards and resources for safe built environment e.g., WASH in health care facilities, including appropriate infrastructure, materials and equipment for IPC; as well as standards for reduction of overcrowding and optimization of staffing levels in health care facilities, according to WHO minimum requirements, exist but they are not fully implemented through a national plan. C) National standards and resources for safe built environment, e.g., WASH in health care facilities, including appropriate infrastructure, materials and equipment fort IPC; as well as standards for reduction of overcrowding and optimization of staffing levels in health care facilities, according to WHO minimum requirements, exist and are implemented in health care facilities at national level through a national plan. D) National standards and resources for safe built environment, e.g., WASH in health care facilities, including appropriate infrastructure, materials and equipment for IPC; as well as standards for reduction of overcrowding and optimization of staffing levels in health care facilities, according to WHO minimum requirements, are implemented at national and intermediate levels according to a national plan. E) National standards and resources for safe built environment, e.g., WASH in health care facilities, including appropriate infrastructure, materials and equipment for IPC; as well as standards for reduction of overcrowding and for optimization of staffing levels in health care facilities, according to WHO minimum requirements, are implemented at national and subnational levels according to a national plan, and are regularly exercised (as applicable) and monitored and improvement actions are taken accordingly.

C10.1 Risk communication and community engagement for emergencies: A) Mechanisms for coordination of RCCE functions and resources are under development, or coordination of RCCE functions and resources is conducted on an ad hoc basis. B) Mechanisms for coordination of RCCE functions and resources, including plans, SOPs and formal government arrangements are developed. C) Mechanisms for coordination of RCCE functions and resources, including plans, SOPs and formal government arrangements are developed and being implemented at the national level. D) Mechanisms for coordination of RCCE functions and resources, including plans, SOPs and formal government arrangements are developed and being implemented at the national and intermediate levels. E) Mechanisms for coordination of RCCE functions and resources are implemented at the national, intermediate and local levels; are fully integrated into emergency response systems; and are exercised, reviewed, evaluated and updated on a regular basis.	Multiple Choice	Multisectoral	Community Awareness & Enabling Behaviours
C10.2. Risk communication: A) Mechanisms for public communication and/or media relations, including infodemics, are under development or implemented on an ad hoc basis. B) Mechanisms for public communication and/or media relations, including infodemics, are developed but not fully implemented with significant gaps. C) Mechanisms for public communication and/or media relations, including infodemics, are developed and activities are being implemented at the national level. D) Mechanisms for public communication and/or media relations, including infodemics, are developed and activities are being implemented and coordinated across all sectors at national and intermediate levels. E) Mechanisms for public communication and/or media relations, including infodemics, are developed and activities	Multiple Choice	Multisectoral	Community Awareness & Enabling Behaviours

are being implemented and coordinated across sectors at national, intermediate and local levels, and information is shared in a timely manner. The mechanisms and related activities are exercised (as applicable),reviewed, evaluated and updated on a regular basis.			
C10.3. Community engagement: A) Mechanisms for systematic community engagement in public health emergencies, including guidelines and/or SOPs, are under development or community engagement activites75 are implemented on an ad hoc basis. B) Mechanisms for systematic community engagement in public health emergencies, including guidelines and/or SOPs, have been developed but not implemented. C) Mechanisms for systematic community engagement in public health emergencies, including guidelines and/or SOPs, have been developed, disseminated and community engagement activities are being implemented and supported at the national level. D) Mechanisms for systematic community engagement in public health emergencies, including guidelines and/or SOPs, have been developed, disseminated and community engagement activities are being implemented and supported at national and intermediate levels. E) Mechanisms for systematic community engagement in public health emergencies, including guidelines and/or SOPs, have been developed, disseminated, and community engagement activities are being implemented and supported at the national, intermediate and local levels. Qualitative and quantitative socio-behavioural research is conducted;77 and mechanisms and activities for community engagement are exercised (as applicable), reviewed, evaluated and updated on a regular basis.	Multiple Choice	Multisectoral	Community Awareness & Enabling Behaviours
C11.1 Core capacity requirements at all times for	Multiple Choice	Multisectoral	Prevention & Control

PoEs (airports, ports and ground crossings): A) Strategic risk assessment for individual PoE as an integral part of a national risk assessment has not been completed. B) Some designated PoE are implementing routine core capacities based on a completed associated strategic risk assessment. C) Some designated PoE are implementing routine core capacities AND These are integrated into the national surveillance system for biological hazards/all hazards (e.g., event-based and early warning surveillance). D) All designated PoE are implementing routine core capacities with an all-hazard and multisectoral approach integrated into the national surveillance system. E) Routine core capacities implemented at all designated PoE are exercised (as appropriate), reviewed, evaluated, updated and actions are taken to improve capacity on a regular basis.			
C11.2. Public health response at points of entry: A) PoE designated based on a strategic risk assessment are in the process of developing a PoE public health emergency contingency plan. B) Some designated PoE have developed a PoE public health emergency contingency plan for events caused by biological hazards. C) All designated PoE have developed PoE public health emergency contingency plans for events caused by biological hazards and integrated into national emergency response plans. D) All designated PoE have developed PoE public health emergency contingency plans for events caused by all hazards and integrated into national emergency response plans. E) All PoE public health emergency contingency plans for events caused by all hazards all designated PoE are exercised (as appropriate), reviewed, evaluated and updated on a regular basis.	Multiple Choice	Multisectoral	Prevention & Control
C11.3. Risk-based approach to international travel-related measures: A) The animal, human,	Multiple Choice	Multisectoral	Prevention & Control

and environment health sectors work together on zoonoses on an ad hoc basis. B) The animal, human and environment health sectors have jointly mapped existing and areas of collaboration and agreed on prioritized zoonoses for coordinated prevention and control activities. C) The animal, human and environment health sectors collaborate regularly and coordinate their activities at national level to prevent, detect assess/investigate and respond to one or more prioritized zoonoses(s). Their ability to detect new or emerging zoonotic diseases has been demonstrated in some occasions. D) The animal, human and environment health sectors collaborate regularly and coordinate their activities at national and intermediate level to prevent, detect assess/investigate and to respond to prioritized zoonoses, and have appropriate procedures to jointly react in case of emergency, including in case of new or emerging zoonotic diseases. E) One Health multisectoral capacities to prevent, detect, assess/investigate and respond to zoonotic events (endemic and emerging) are exercised (as applicable, reviewed, evaluated, updated on a regular basis and improvements are implemented accordingly).			
C12.1. One Health collaborative efforts across sectors on activities to address zoonoses: A) The animal, human, and environment health sectors work together on zoonoses on an ad hoc basis. B) The animal, human and environment health sectors have jointly mapped existing and areas of collaboration and agreed on prioritized zoonoses for coordinated prevention and control activities. C) The animal, human and environment health sectors collaborate regularly and coordinate their activities at national level to prevent, detect assess/investigate and respond to one or more prioritized zoonoses(s). Their ability to detect new or emerging zoonotic diseases has been	Multiple Choice	Multisectoral	Coordination / Prevention & Control

	demonstrated in some occasions. D) The animal, human and environment health sectors collaborate regularly and coordinate their activities at national and intermediate level to prevent, detect assess/investigate and to respond to prioritized zoonoses, and have appropriate procedures to jointly react in case of emergency, including in case of new or emerging zoonotic diseases. E) One Health multisectoral capacities to prevent, detect, assess/investigate and respond to zoonotic events (endemic and emerging) are exercised (as applicable, reviewed, evaluated, updated on a regular basis and improvements are implemented accordingly).			
	C13.1. Multisectoral collaboration mechanism for food safety events: A) A multisectoral collaboration mechanism that includes an INFOSAN Emergency Contact Point is under development, activated on an ad hoc basis. B) A multisectoral collaboration mechanism that includes the INFOSAN Emergency Contact Point is in place at the national level AND Communication channels between the INFOSAN Emergency Contact Point, the National IHR Focal Point and all relevant sectors for food safety events, including for emergencies, have been established at the national level. C) A multisectoral collaboration mechanism and communication channels that includes the INFOSAN Emergency Contact Point is in place at the national, intermediate and local levels, if appropriate, to the structure and governance of the country. D) A multisectoral collaboration mechanism and communication channels between the INFOSAN Emergency Contact Point, the National IHR Focal Point and all relevant sectors for food safety events including emergencies, at the international level have been established. E) The multisectoral collaboration mechanism related	Multiple Choice	Multisectoral	Coordination

		to food safety events and Communication channels between the INFOSAN emergency contact, the National IHR Focal Point, and other relevant sectors for food safety events including emergencies at national and international level have been exercised (as applicable), reviewed, evaluated and updated as appropriate.			
42	European Medicines Agency Science Medicines Health	Report antimicrobial use by AMEG categorisation [relative use % of Category A (Avoid) antibiotics; relative use % of Category B (Restrict) antibiotics; relative use % of Category C (Caution) antibiotics; relative use % of Category D (Prudence) antibiotics]:	Quantitative	Animal Health	Stewardship
43	Minimizing risk of developing antibiotic resistance and aquatic ecotoxicity in the environment resulting from the manufacturing of human antibiotics	The user shall minimize the release of non-routine antibiotic discharges to the environment, including spills, application of treated wastewater to land for irrigation and firewater run-off containment,	Target	Environment	Stewardship
		The user shall demonstrate and check controls are in place for effective and safe handling, movement, storage, recycling, reuse and disposal of antibiotic waste.	Target	Environment	Stewardship
		The user shall have systems in place to prevent and mitigate accidental spills and releases to the environment. In the case of unpermitted or accidental release of antibiotic waste in the environment, remedial measures shall be in place to prevent reoccurrence and address associated environmental impacts.	Target	Environment	Stewardship
		The user shall incinerate or dispose of solid waste containing antibiotic residue to a secure landfill site. The user shall require the operator to confirm the landfill site is secure, designed and operated to prevent release into the environment.	Target	Environment	Stewardship
44	Addressing gender inequalities in national action plans on AMR	Promote research to strengthen the evidence base on the intersections between gender and AMR.	Target	Human Health	Equity
		Use context-specific messages, language and images in AMR awareness and education materials that actively address harmful gender norms and promote gender equality.	Target	Human Health	Equity

	Promote equal participation of women, men and other vulnerable groups and/or groups facing discrimination in the multisectoral AMR coordination mechanism and technical working groups.	Target	Human Health	Equity
	Include representation from gender experts in the multisectoral AMR coordination mechanism	Target	Human Health	Equity
	Report on patients' sex, age and, where feasible, other social stratifiers as part of routine surveillance systems on AMR and antimicrobial use.	Target	Human Health	Equity
	Invest in new diagnostics for infections that disproportionately affect women such as (drug resistant) UTIs.	Target	Human Health	Equity
	Improve WASH and waste management infrastructure in health facilities and community settings to ensure infrastructure is available, accessible and safe for all genders, and does not perpetuate stigma and discrimination.	Target	Human Health	Equity
	Identify and address gender inequalities in the risk of exposure to (drug-resistant) infections among health care workers and in community settings.	Target	Human Health	Equity
	Deliver culturally sensitive and gender-responsive health services for the prevention, diagnosis and treatment of (drug-resistant) infections.	Target	Human Health	Equity
	Identify and address gender inequalities in access to quality-assured medicines including antimicrobials, focusing on specific groups of women or men who might be at a higher risk of purchasing substandard or falsified antimicrobials.	Target	Human Health	Equity
	Ensure health insurance and/or health benefit packages cover access to health services, diagnostics and antimicrobials for the treatment of (drug-resistant) infections without leaving behind vulnerable populations.	Target	Human Health	Equity
	Update and implement standards on the forecasting and procurement of medicines including antimicrobials by undertaking an assessment of the local epidemiology of infections based on sex to	Target	Human Health	Equity

		ensure all relevant antimicrobials are included.			
		Conduct retrospective reviews of diagnostic services for different (drug-resistant) infections to identify and address any gender inequalities.	Target	Human Health	Equity
		Apply a gender analysis in regular retrospective prescription audits to identify unconscious gender biases or inequalities in prescribing practices.	Target	Human Health	Equity
		Conduct a gender assessment of the unintended effect of policies or regulations that aim to reduce over-the-counter sale of antimicrobials on access to essential antimicrobials.	Target	Human Health	Equity
		Capture and disaggregate data on AMR and surveillance of antimicrobial use and other relevant data by, at minimum, sex and age and, where feasible, other social stratifiers.	Target	Human Health	Equity
45	TrACSS 2023	2.1 Multi-sector and One Health collaboration/coordination	Ordinal	Multisectoral	Coordination
		2.2 Which sectors are actively involved in the multisector coordination mechanism?	Multiple choice	Multisectoral	Coordination
		2.3 Country progress with development of a national action plan on AMR	Ordinal	Multisectoral	Strategic Vision
		2.3.a If the duration of national AMR plan has/is about to expire, is the country in the process of revising the national AMR action plan or developing a new one?	Y/N	Multisectoral	Accountability
		2.3.b Does the country have a monitoring and evaluation plan for the national AMR action plan?	Y/N	Multisectoral	Effectiveness/Feedback Mechanisms
		2.3.c Is there a focal point or specific working group responsible for the monitoring and evaluation of the implementation of the national AMR action plan?	Y/N	Multisectoral	Strategic Vision, Accountability
		2.3.d Regular collection of monitoring data across sectors	Y/N	Multisectoral	Reporting
		2.3.e Do you have adequate technical capacity, resources and established systems in the country to collect data across all relevant sectors?	Y/N	Multisectoral	Sustainability
		2.3.f Are relevant data disaggregated by sex, geographic location, income, etc.?	Y/N	Multisectoral	Equity / Reporting
		2.3.g Is the data analysed and used by the AMR	Y/N	Multisectoral	Feedback Mechanisms

	multisector coordination mechanism for decision making across all relevant sectors and to advocate for policy changes and allocation of adequate resources?			
	2.4.a Does the multisector coordination mechanism meet to discuss the response to the TrACSS questionnaire before submission?	Y/N	Multisectoral	Coordination
	2.4.b Does the multisector coordination mechanism review current and previous year data from TrACSS as a way to monitor progress of AMR national action plan implementation?	Y/N	Multisectoral	Feedback Mechanisms
	2.5 Status update on development/delivery of NAP	Open response	Multisectoral	Reporting
	2.6 Is your country's national planning on AMR integrated with other existing action plans, or strategies?	Y/N - if yes, select which other plans integrated	Multisectoral	Coordination
	2.7 How has COVID-19 pandemic and its national response, either positively or negatively, impacted the AMR National Action Plan implementation?	Open response	Multisectoral	Effectiveness
	2.8.1 Country has laws or regulations on prescription and sale of antimicrobials, for human use	Y/N	Human Health	Regulations & Legislation
	2.8.2 Country has laws or regulations on prescription and sale of antimicrobials for terrestrial animal use	Y/N	Animal Health	Regulations & Legislation
	2.8.3 Country has laws or regulations on prescription and sale of antimicrobials for aquatic animals	Y/N	Animal Health	Regulations & Legislation
	2.8.4 Country has laws or regulations on prescription and sale of medicated feed	Y/N	Animal Health	Regulations & Legislation
	2.8.5 Country has laws or regulations that prohibit the use of antibiotics for growth promotion in terrestrial animals in the absence of risk analysis	Y/N	Animal Health	Regulations & Legislation
	2.8.6 Country has legislation on the registration and use of applicable pesticides with antimicrobial effects, such as bactericides and fungicides used in plant production	Y/N	Environment	Regulations & Legislation
	2.9 Raising awareness and understanding of AMR risks and response	Ordinal	Multisectoral	Community Awareness & Enabling Behaviours

	2.9.1 For the level selected above, please indicate the extent of involvement of the sectors below: human health, terrestrial animal health, aquatic animal health, plant health, food production, food safety, environment	Ordinal	Multisectoral	Community Awareness & Enabling Behaviours
	2.10 Youth education on AMR	Y/N	Multisectoral	Community Awareness & Enabling Behaviours
	2.11 Is the country using relevant antimicrobial consumption/use data to inform operational decision making and amend policies?	Y/N - if yes, indicate which sectors	Multisectoral	Feedback Mechanisms
	2.12 Is the country using relevant antimicrobial resistance surveillance data to inform operational decision making and policies?	Y/N - if yes, indicate which sectors	Multisectoral	Feedback Mechanisms
	2.13 Has the country established or started the implementation of an Integrated Surveillance System for Antimicrobial Resistance?	Y/N - if yes, indicate which sectors	Multisectoral	Surveillance & Laboratory
	3.1 Training and professional education on AMR in the human health sector	Ordinal	Human Health	Workforce
	3.2 National monitoring system for consumption and rational use of antimicrobials in human health	Ordinal	Human Health	Stewardship
	3.3 National surveillance system for antimicrobial resistance in humans	Ordinal	Human Health	Surveillance & Laboratory
	3.4.1 Coverage of bacteriology lab services	Quantitative	Multisectoral	Surveillance & Laboratory
	3.4.2 Capacity to perform AST for critically important bacteria	Ordinal	Multisectoral	Surveillance & Laboratory
	3.4.3 Capacity to perform AST for critically important fungi	Yes (Candida), yes (aspergillus), yes (both), no, unknown	Multisectoral	Surveillance & Laboratory
	3.4.4 Continuity of services for National Reference Bacteriology/AST Laboratory	Quantitative	Multisectoral	Surveillance & Laboratory
	3.4.5 Continuity of services for clinical bacteriology labs: mechanism for reporting stock-outs	Yes (local level), yes (national level), no, unknown	Multisectoral	Surveillance & Laboratory
	3.4.6 Standardized AST guidelines	Multiple choice	Multisectoral	Surveillance & Laboratory
	3.4.7 External quality-assured bacteriology laboratory services: does the country have an external quality assurance EQA programme and to what extent is it implemented?	Multiple choice	Multisectoral	Surveillance & Laboratory

3.4.8 Does the country have developed a national list of essential in-vitro diagnostics that includes all essential AMR diagnostics?	Multiple choice	Multisectoral	Surveillance & Laboratory
3.5 Infection prevention and control in human health care	Ordinal	Human Health	Prevention & Control
3.6 Optimizing antimicrobial use in human health	Ordinal	Human Health	Stewardship
3.7 Adoption of "AWaRe" classification of antibiotics in the National Essential Medicines List	Multiple choice	Human Health	Stewardship
4.1 Training and professional education on AMR in the veterinary sector	Ordinal	Animal Health	Workforce
4.2 Training and education on AMR in the aquatic animal health sector	Ordinal	Animal Health	Workforce
4.3 Progress with strengthening veterinary services	Ordinal	Animal Health	Access to Medicines and Health Services
4.4 Progress with strengthening aquatic animal health services	Ordinal	Animal Health	Access to Medicines and Health Services
4.5.a Do you have a national plan or system in place for monitoring sales/use of antimicrobials in animals?	Yes (terrestrial), Yes (aquatic), Yes (both), no	Animal Health	Stewardship
4.5.b Do you submit AMU data to the WOAH database on antimicrobial agents intended for use in animals?	Y/N/unknown	Animal Health	Reporting
4.6 WOAH reporting options for the antimicrobial use database	Multiple choice	Animal Health	Reporting
4.7 National surveillance system for antimicrobial resistance in live terrestrial animals	Multiple choice	Animal Health	Surveillance & Laboratory
4.7.1 AMR surveillance is routinely undertaken in live terrestrial animals for the following categories: Terrestrial animal isolates linked to animal disease, Zoonotic pathogenic bacteria (e.g. Salmonella spp. in terrestrial animals), Commensal isolates (e.g. E. coli for terrestrial animals), Specific resistance phenotypes (e.g. ESBL producing indicator E. coli obtained from healthy animals in key food producing species).	Multiple choice	Animal Health	Surveillance & Laboratory
4.8 National surveillance system for antimicrobial resistance in live aquatic animals	Multiple choice	Animal Health	Surveillance & Laboratory
4.8.1 AMR surveillance is routinely undertaken in	Y/N	Animal Health	Surveillance & Laboratory

	live aquatic animals for the following categories: Aquatic animal isolates linked to animal disease (e.g. Aeromonas spp.), Zoonotic pathogenic bacteria (e.g. Vibrio parahaemolyticus), Commensal isolates (e.g. non-pathogenic Aeromonas hydrophila).			
	4.9 Biosecurity and good animal husbandry practices to reduce the use of antimicrobials and minimize development and transmission of AMR in terrestrial animal production	Ordinal	Animal Health	Stewardship
	4.10 Biosecurity and good animal husbandry practices to reduce the use of antimicrobials and minimize development and transmission of AMR in aquatic animal production	Ordinal	Animal Health	Stewardship
	4.11 Optimizing antimicrobial use in terrestrial animal health	Ordinal	Animal Health	Stewardship
	4.12 Optimizing antimicrobial use in aquatic animal health	Ordinal	Animal Health	Stewardship
	5.1 Training and professional education on AMR provided to the agriculture (animal and plant), food production, food safety and environment sectors	Multiple choice	Multisectoral	Workforce
	5.2 National monitoring system for antimicrobial-pesticide use in plant production including bactericides and fungicides	Multiple choice	Environment	Surveillance & Laboratory
	5.3 National surveillance system for antimicrobial resistance in food (terrestrial and aquatic animal and plant origin)	Multiple choice	Multisectoral	Surveillance & Laboratory
	5.3.1 AMR surveillance is systematically undertaken in food (terrestrial and aquatic animal and plant origin) in the following categories	Y/N by sector and by bacteria variety	Animal Health	Surveillance & Laboratory
	5.4.a Effective integration of laboratories in the AMR surveillance in the animal health and food safety sectors	Multiple choice	Animal Health	Surveillance & Laboratory
	5.4.b Level of the standardization and harmonization of procedures among laboratories included in the AMR surveillance system in the animal health and food safety sectors	Multiple choice	Animal Health	Surveillance & Laboratory
	5.4.c Relevance of diagnostic (bacteriology) techniques used by laboratories included in the	Multiple choice	Animal Health	Surveillance & Laboratory

AMR surveillance system in the animal health and food safety sectors			
5.4.d Technical level of data management of the laboratory network in the AMR surveillance system in the animal health and food safety sectors	Multiple choice	Animal Health	Surveillance & Laboratory
5.5 Good manufacturing and hygiene practices to reduce the development and transmission of AMR in food processing	Ordinal	Environment	Prevention & Control
5.6 Optimizing antimicrobial pesticide such as bactericides and fungicides use in plant production	Multiple choice	Environment	Stewardship
6.1 Has a national assessment of risks for residues of antimicrobial compounds and antimicrobial resistant pathogens in the environment been conducted?	Y/N	Environment	Prevention & Control
6.1.1 Conducted risk assessment for AMR spread in the environment: human sewage treatment quality	Y/N	Environment	Prevention & Control
6.1.2 Conducted risk assessment for AMR spread in the environment: wastewater discharges from health facilities	Y/N	Environment	Prevention & Control
6.1.3 Conducted risk assessment for AMR spread in the environment: Management of solid clinical waste from health facilities destroyed by incineration	Y/N	Environment	Prevention & Control
6.1.4 Conducted risk assessment for AMR spread in the environment: Disposal of medicines, antimicrobial agents for human use	Y/N	Environment	Prevention & Control
6.1.5 Conducted risk assessment for AMR spread in the environment: Disposal of medicines, antimicrobial agents for animal use	Y/N	Environment	Prevention & Control
6.1.6 Conducted risk assessment for AMR spread in the environment: Discharges from intensive terrestrial animal production	Y/N	Environment	Prevention & Control
6.1.7 Conducted risk assessment for AMR spread in the environment: Discharges from intensive aquatic animal production	Y/N	Environment	Prevention & Control
6.1.8 Conducted risk assessment for AMR spread in the environment: Wastewater discharges from manufacturing sites for antimicrobial agents	Y/N	Environment	Prevention & Control

6.1.9 Conducted risk assessment for AMR spread in the environment: Disposal of food, plant or animal products contaminated with antimicrobial residues over the MRL	Y/N	Environment	Prevention & Control
6.1.10 Conducted risk assessment for AMR spread in the environment: Runoff and solid waste from slaughterhouses, traditional markets and food processing plants	Y/N	Environment	Prevention & Control
6.1.11 Conducted risk assessment for AMR spread in the environment: Liquid and solid waste from intensive terrestrial animal production prior to use in agriculture	Y/N	Environment	Prevention & Control
6.1.12 Conducted risk assessment for AMR spread in the environment: Liquid and solid waste from intensive aquatic animal production prior to use in agriculture	Y/N	Environment	Prevention & Control
6.1.13 Conducted risk assessment for AMR spread in the environment: Human solid waste that may be used for agricultural purposes	Y/N	Environment	Prevention & Control
6.1.14 Conducted risk assessment for AMR spread in the environment: Overspray, drift and leaching following pesticide applications	Y/N	Environment	Prevention & Control
6.1.15 Conducted risk assessment for AMR spread in the environment: Dust, drift and leaching following fertilizer applications	Y/N	Environment	Prevention & Control
6.1.16 Conducted risk assessment for AMR spread in the environment: Transnational and intercontinental transport and movement of food, goods, live animals and people	Y/N	Environment	Prevention & Control
6.1.17 Conducted risk assessment for AMR spread in the environment: Storm runoff, wastewater treatment plant overflow or failure	Y/N	Environment	Prevention & Control
6.2 Country has legislation and/or regulations to prevent contamination of the environment with antimicrobials - antimicrobial compounds and their metabolites discharged to the environment	Y/N	Environment	Regulations & Legislation
6.2.1 Legislation/regulation to mitigate risks: human sewage treatment quality	Y/N	Environment	Regulations & Legislation
6.2.2 Legislation/regulation to mitigate risks:	Y/N	Environment	Regulations & Legislation

wastewater discharges from health facilities			
6.2.3 Legislation/regulation to mitigate risks: Management of solid clinical waste from health facilities destroyed by incineration	Y/N	Environment	Regulations & Legislation
6.2.4 Legislation/regulation to mitigate risks: Disposal of medicines, antimicrobial agents for human use	Y/N	Environment	Regulations & Legislation
6.2.5 Legislation/regulation to mitigate risks: Disposal of medicines, antimicrobial agents for animal use	Y/N	Environment	Regulations & Legislation
6.2.6 Legislation/regulation to mitigate risks: Discharges from intensive terrestrial animal production	Y/N	Environment	Regulations & Legislation
6.2.7 Legislation/regulation to mitigate risks: Discharges from intensive aquatic animal production	Y/N	Environment	Regulations & Legislation
6.2.8 Legislation/regulation to mitigate risks: Wastewater discharges from manufacturing sites for antimicrobial agents	Y/N	Environment	Regulations & Legislation
6.2.9 Legislation/regulation to mitigate risks: Disposal of food, plant or animal products contaminated with antimicrobial residues over the MRL	Y/N	Environment	Regulations & Legislation
6.2.10 Legislation/regulation to mitigate risks: Runoff and solid waste from slaughterhouses, traditional markets and food processing plants	Y/N	Environment	Regulations & Legislation
6.2.11 Legislation/regulation to mitigate risks: Liquid and solid waste from intensive terrestrial animal production prior to use in agriculture	Y/N	Environment	Regulations & Legislation
6.2.12 Legislation/regulation to mitigate risks: Liquid and solid waste from intensive aquatic animal production prior to use in agriculture	Y/N	Environment	Regulations & Legislation
6.2.13 Legislation/regulation to mitigate risks: Human solid waste that may be used for agricultural purposes	Y/N	Environment	Regulations & Legislation
6.2.14 Legislation/regulation to mitigate risks: Overspray, drift and leaching following pesticide applications	Y/N	Environment	Regulations & Legislation
6.2.15 Legislation/regulation to mitigate risks:	Y/N	Environment	Regulations & Legislation

		Dust, drift and leaching following fertilizer applications			
		6.2.16 Legislation/regulation to mitigate risks: Transnational and intercontinental transport and movement of food, goods, live animals and people	Y/N	Environment	Regulations & Legislation
		6.2.17 Legislation/regulation to mitigate risks: Storm runoff, wastewater treatment plant overflow or failure	Y/N	Environment	Regulations & Legislation
		6.3 Is there a system for regular monitoring of antimicrobial compounds and their metabolites and resistant bacteria or antimicrobial resistance genes in water quality	Y/N (if yes, check waters that apply)	Environment	Surveillance & Laboratory
46	Operational approach to antimicrobial stewardship in the WHO Eastern Mediterranean Region	Country is formally part of the Member State Mechanism to address the issue of tackling substandard and falsified medical products and implements its work plan	Qualitative	Multisectoral	Accountability/Stewardship
47	EU Council recommendation on stepping up EU actions to combat AMR in a One Health approach	Take appropriate national measures aimed at ensuring that, by 2030, at least 65 % of the total consumption of antibiotics in humans belongs to the Access group of antibiotics as defined in the AWaRe classification of the WHO	Target	Human Health	Stewardship
		Take appropriate national measures aimed at ensuring that, by 2030, the total consumption of antibiotics in humans (in Defined Daily Dose (DDD) per 1 000 inhabitants per day), in the community and hospital sectors combined, including in long-term care facilities and in home-care settings, is reduced by 20 % in the Union compared with the baseline year 2019	Target	Human Health	Stewardship
		Take appropriate national measures aimed at ensuring that, by 2030, the total incidence of bloodstream infections with carbapenem-resistant Klebsiella pneumoniae (number per 100 000 population) is reduced by 5 % in the EU, compared to the baseline year 2019.	Target	Human Health	Prevention & Control
		Take appropriate national measures aimed at ensuring that, by 2030, the total incidence of bloodstream infections with methicillin-resistant Staphylococcus aureus (MRSA) (number per 100	Target	Human Health	Prevention & Control

	000 population) is reduced by 15 % in the EU, compared to the baseline year 2019			
	Take appropriate national measures aimed at ensuring that, by 2030, the total incidence of bloodstream infections with third generation cephalosporins-resistant Escherichia coli (number per 100 000 population) is reduced by 10 % in the EU, compared to the baseline year 2019.	Target	Human Health	Prevention & Control
	Put in place appropriate measures to contribute to the achievement of the Farm to Fork Strategy and Zero Pollution Action Plan target of 50 % reduction of the overall EU sales of antimicrobials used for farmed animals and in aquaculture by 2030	Target	Animal Health	Stewardship
	Ensure that the minimum requirements for national infection prevention and control programmes in healthcare facilities are in place to provide adequate protection and safety for patients, health workers and visitors, through, inter alia, implementation of WHO's global strategy on infection prevention and control (2023), the Immunization Agenda 2030, the WHO water, sanitation and hygiene strategy 2018-2025, and the WHO global patient safety action plan 2021-2030, with the goal of achieving their respective targets, such as 100 per cent of countries having basic water, sanitation and hygiene and waste services in all health care facilities and 90 per cent of countries meeting all WHO's minimum requirements for infection prevention and control programs at the national level by 2030	Target	Human Health	Prevention & Control
	Improve access to diagnosis and care, so at least 80 per cent of countries can test resistance in all bacterial and fungal GLASS pathogens by 2030	Target	Human Health	Access to Medicines & Healthcare Services
	Improve monitoring and evaluation of the implementation of multisectoral national action plans on antimicrobial resistance by building country-level technical capacity and ensure that 95 per cent of countries participate in TrACSS by	Target	Multisectoral	Sustainability

		2030			
48	G7 Compliance Report on Antimicrobial Resistance, 2021-2023	Overall country-level compliance score	3-point scale	Multisectoral	Sustainability
